# Revealing the pathogenic changes of PAH based on multiomics characteristics

**DOI:** 10.1186/s12967-019-1981-5

**Published:** 2019-07-22

**Authors:** Li Zhang, Shaokun Chen, Xixi Zeng, Dacen Lin, Yumei Li, Longxin Gui, Mo-jun Lin

**Affiliations:** 1 Department of Physiology & Pathophysiology, Fujian Medical University, Fuzhou, China; 2 The Key Laboratory of Fujian Province University on Ion Channel and Signal Transduction in Cardiovascular Disease, School of Basic Medical Sciences, Fujian Medical University, Fuzhou, China; 3 Fujian Center for Safety Evaluation of New Drug, Fujian Medical University, Fuzhou, China

**Keywords:** Pulmonary artery hypertension, Pulmonary arteries, Multiomics, Proteomic, Transcriptomic, DNA methylation

## Abstract

**Background:**

Pulmonary artery hypertension (PAH), which is characterized by an increase in pulmonary circulation blood pressure, is a fatal disease, and its pathogenesis remains unclear.

**Methods:**

In this study, RNA sequencing (RNA-seq), tandem mass tags (TMT) and reduced representation bisulfite sequencing (RRBS) were performed to detect the levels of mRNA, protein, and DNA methylation in pulmonary arteries (PAs), respectively. To screen the possible pathways and proteins related to PAH, pathway enrichment analysis and protein–protein interaction (PPI) network analysis were performed. For selected genes, differential expression levels were confirmed at both the transcriptional and translational levels by real-time PCR and Western blot analyses, respectively.

**Results:**

A total of 362 differentially expressed genes (|Fold-change| > 1.5 and *p *< 0.05), 811 differentially expressed proteins (|Fold-change| > 1.2 and *p *< 0.05) and 76,562 differentially methylated regions (1000 bp slide windows, 500 bp overlap, *p *< 0.05, and |Fold-change| > 1.2) were identified when the PAH group (n = 15) was compared with the control group (n = 15). Through an integrated analysis of the characteristics of the three omic analyses, a multiomics table was constructed. Additionally, pathway enrichment analysis showed that the differentially expressed proteins were significantly enriched in five Kyoto Encyclopedia of Genes and Genomes (KEGG) biological pathways and ten Gene Ontology (GO) terms for the PAH group compared with the control group. Moreover, protein–protein interaction (PPI) networks were constructed to identify hub genes. Finally, according to the genes identified in the PPI and the protein expression fold-change, nine key genes and their associated proteins were verified by real-time PCR and Western blot analyses, including Col4a1, Itga5, Col2a1, Gstt1, Gstm3, Thbd, Mgst2, Kng1 and Fgg.

**Conclusions:**

This study conducted multiomic characteristic profiling to identify genes that contribute to the hypoxia-induced PAH model, identifying new avenues for basic PAH research.

**Electronic supplementary material:**

The online version of this article (10.1186/s12967-019-1981-5) contains supplementary material, which is available to authorized users.

## Background

Pulmonary arterial hypertension (PAH) is clinically defined as the elevation of mean pulmonary artery pressure (mPAP) > 25 mmHg at rest [1]. It is pathologically characterized by the proliferation, migration, anti-apoptosis, or phenotype switching of pulmonary arterial endothelial cells, pulmonary arterial smooth muscle cells, and fibroblasts. Despite recent achievements in the treatment of PAH, most current therapies only improve the symptoms, rather than cure them. Thus, there is an urgent need to explore the genes, pathways or epigenetic factors that drive PAH to identify potential therapeutic targets.

A number of previous transcriptomic and proteomic studies have revealed the pathological mechanisms underlying pulmonary hypertension in different samples; most of the studies were performed in lung homogenate samples [2–7] and circulating cells (peripheral blood) [14, 15], although some studies have been performed in pulmonary arterial samples [8–11] and specific cell types (isolated primary cells) [12, 13]. The studies examined multiple different species, including *Homo sapiens*, *Rattus norvegicus* and *Mus musculus*. However, previous studies were primarily based on single-omics analyses, especially transcriptome analyses, and rarely included epigenetic analyses. DNA methylation is one of the most stable epigenetic modifications and is traditionally regarded as the major mediator of epigenetic regulation. Recent studies have demonstrated the role of epigenetic modifications in the pathogenesis of PAH [16–18], suggesting that DNA methylation may be associated with the etiology of PAH. Although genome-wide analyses have contributed greatly to our understanding of the genetic basis of PAH, the deeper and more comprehensive mechanisms of PAH must still be explained.

Chronic hypoxia causes a decrease in blood oxygen saturation, leading to the persistent constriction of pulmonary arterioles and pathological changes in blood vessels. These changes are clinically relevant, as hypoxia is one of the most common inducers of PAH worldwide [19–21]. The expression of vascular-specific genes can be masked in lung homogenates because intrapulmonary arteries represent only a small percentage of total lung tissues [22]. Therefore, based on previous studies and considering the pros and cons of different materials, pulmonary arteries (PAs) were chosen for this experiment, with the aim of identifying vascular-specific genes.

In this study, we aimed to identify gene expression patterns, potential signaling pathways and the epigenetic characteristics of PAs in rats suffering from hypoxia-induced PAH. PAs were isolated from connective tissues and cleaned. RNA sequencing (RNA-seq), tandem mass tags (TMT) and reduced representation bisulfite sequencing (RRBS) were performed to screen for differentially expressed genes (DEGs), differentially expressed proteins (DEPs) and differentially methylated regions (DMRs), respectively. Then, for the first time, we combined the information from these three omics analyses to identify the pathogenic characteristics of hypoxic-induced PAH in PAs. The integrated analysis of these multiomics sequencing data contributed to a detailed understanding of the mechanisms underlying PAH, which may play an important role in the development of new and more effective treatment targets and contribute to the development of new therapeutic drugs for PAH.

## Methods

### PAH models and sample collection

Experiments were performed on male Sprague–Dawley (SD) rats (200–250 g), supplied by the animal center of the Fujian Medical University. All procedures were approved by the Animal Care and Use Committee of Fujian Medical University. Hypoxia-induced PAH was produced using an established method [23, 24]. Rats were placed in a hypoxic chamber and exposed to 10% O_2_ for 3 weeks. Meanwhile, the control group was placed in normoxic conditions for 3 weeks. Right ventricle systolic pressure (RVSP) was measured by accessing the right ventricle through the jugular vein using a polyethylene catheter connected to a pressure transducer (YPJ01; Chengyi, China). Pressure signals were displayed continuously on an RM6240 polygraph (Chengyi, China). The right ventricular mass index (RVMI) was calculated by determining the right ventricle/(left ventricle + ventricular septal) [RV/(LV + S)] mass ratio.

Rats were injected with heparin and anesthetized with urethane. The lungs were removed and transferred to a petri dish filled with cold, oxygenated modified Krebs solution containing the following (in mM): 118 NaCl, 4.7 KCl, 1.2 MgSO_4_, 1.18 KH_2_PO_4_, 25 NaHCO_3_, 10 glucose, and 2 CaCl_2_. Under a dissecting microscope, PAs were isolated from connective tissue and cleaned. In this experiment, the PAs were taken from hypoxia-induced PAH rats (n = 15) and normoxia control rats (n = 15). However, due to the necessary sample sizes for the multiomics analysis, we pooled the PAs from five animals from the same group into one sample. Therefore, both the PAH group and the control group contained three pooled samples each. Meanwhile, lung tissues were collected for hematoxylin–eosin staining.

### Transcriptomic analysis

#### RNA isolation, purification, and quantification

Total RNA was extracted from rats PAs using TRIzol (Invitrogen, Carlsbad, CA, USA) following the manufacturer’s instructions. The quantity and purity of total RNA were assessed using a Bioanalyzer 2100 and the RNA 6000 Nano LabChip Kit (Agilent, CA, USA), with an RNA integrity number > 7.0.

#### cDNA library construction and sequencing

Poly(A) mRNA was isolated from total RNA with poly-T oligo-attached magnetic beads (Invitrogen, Waltham, US). Following purification, the mRNA was fragmented into small pieces using divalent cations and incubated with Fragmentation Buffer (Illumina) in a preheated tube for 5 min at 94 °C. Then, the cleaved RNA fragments were reverse-transcribed to create cDNA, which was used to synthesize U-labeled second-stranded DNAs using *E. coli* DNA polymerase I, RNase H and dUTPs. An A-base was then added to the blunt ends of each strand, preparing them for ligation with the indexed adapters. Each adapter contained a T-base overhang to ligate the adapter with the A-tailed fragmented DNA. Single-or dual-index adapters were ligated with the fragments, and size selection was performed using AMPureXP beads. After the heat-labile uracil-DNA glycosylase enzyme treatment of the U-labeled second-stranded DNAs, the ligated products were amplified using PCR under the following conditions: initial denaturation at 95 °C for 3 min; 8 cycles of denaturation at 98 °C for 15 s, annealing at 60 °C for 15 s, and extension at 72 °C for 30 s; and then a final extension at 72 °C for 5 min (in accordance with the protocol provided with the mRNA-Seq Sample Preparation Kit [Illumina, San Diego, USA]). The average insert size for the paired-end libraries was 300 bp (± 50 bp). Finally, we performed 150 bp paired-end sequencing on an Illumina Hiseq 4000 (LC Bio, China), following the vendor’s recommended protocol.

#### Data analysis

First, Cutadapt [25] and in-house Perl scripts were used to remove the reads that contained adapter contamination, low-quality bases and undetermined bases. Hisat software (version 2.0) was used to compare the sequencing data with the reference genome, and the transcript was assembled using the results of the alignments. After the final transcriptome was generated, StringTie (version 1.3.0) [26] and Ballgown [27] were used to estimate the expression levels of all transcripts. StringTie was used to analyze the expression levels of mRNAs by calculating the FPKM. The DEGs were determined to be those genes with |Fold-change| > 1.5 and *p *< 0.05.

### Tandem mass tags analysis

#### Sample preparation, protein digestion and TMT labeling

Pulmonary artery samples were ground into powder in liquid nitrogen and extracted with lysis buffer. Two milliliters of lysis buffer (7 M urea, 4% SDS, 1× Protease Inhibitor Cocktail [Roche Ltd. Basel, Switzerland]) was added to each sample, followed by sonication on ice and centrifugation at 13,000 rpm for 10 min at 4 °C. The protein concentration of the supernatant was determined using the BCA protein assay, 100 µg of protein per condition was transferred into new tubes, and the final volume was adjusted to 100 µl with 100 mM TEAB. To each sample, 5 µl of 200 mM DTT was added, and samples were incubated at 50 °C for 1 h. Then, 5 µl of 375 mM iodoacetamide was added, and the samples were incubated for 30 min at room temperature, protected from light. For each sample, proteins were precipitated with ice-cold acetone and were then redissolved in 100 µl of 100 mM TEAB. Then, the proteins were digested with sequence-grade modified trypsin (Promega, Madison, WI), and the resultant peptide mixture was labeled using chemicals from the 10-plex TMT Reagent Kit (Thermo Scientific, USA). The labeled samples were combined and desalted using a C18 SPE column (Sep-Pak C18, Waters, Milford, MA).

#### High pH reverse phase separation

The peptide mixture was redissolved in buffer A (buffer A: 10 mM ammonium formate in water, pH 10.0, adjusted with ammonium hydroxide) and then fractionated by high pH separation using an Aquity UPLC system (Waters Corporation, Milford, MA) connected to a reverse phase column (BEH C18 column, 2.1 mm × 150 mm, 1.7 µm, 300 Å, Waters Corporation, Milford, MA). High pH separation was performed using a linear gradient, increasing from 0% B to 45% B in 35 min (B: 10 mM ammonium formate in 90% ACN, pH 10.0, adjusted with ammonium hydroxide). The column flow rate was maintained at 250 µl/min, and the column temperature was maintained at 45 °C. Twelve fractions were collected, and each fraction was dried in a vacuum concentrator for the next step.

#### Low pH nano-HPLC–MS/MS analysis

Each fraction was resuspended with 32 µl of solvent C (C: water with 0.1% formic acid; D: ACN with 0.1% formic acid), separated by nano liquid chromatography (LC) and analyzed by online electrospray tandem mass spectrometry (MS/MS). The experiments were performed on an EASY-nLC 1000 system (Thermo Fisher Scientific, Waltham, MA), connected to an Orbitrap Fusion mass spectrometer (Thermo Fisher Scientific, San Jose, CA), and equipped with an online nanoelectrospray ion source. For each sample, 4 µl of peptide was loaded onto the trap column (Thermo Scientific Acclaim PepMap C18, 100 µm × 2 cm), with a flow rate of 10 µl/min for 3 min, and subsequently separated on the analytical column (Acclaim PepMap C18, 75 µm × 25 cm), with a linear gradient from 5% D to 30% D over 95 min. The column was re-equilibrated at initial conditions for 15 min. The column flow rate was maintained at 300 nl/min, and the column temperature was maintained at 45 °C. An electrospray voltage of 2.0 kV versus the inlet of the mass spectrometer was used. The Orbitrap Fusion mass spectrometer was operated in the data-dependent mode to switch automatically between mass spectrometry (MS) and MS/MS acquisition modes. Survey full-scan MS spectra (m/z 400–1600) were acquired in Orbitrap with a mass resolution of 60,000 at m/z 200. The automatic gain control (AGC) target was set to 500,000, and the maximum injection time was 50 ms. MS/MS acquisition was performed in Orbitrap with a 3 s cycle time and a resolution of 15,000 at m/z 200. The intensity threshold was 50,000, and the maximum injection time was 150 ms. The AGC target was set to 150,000, and the isolation window was 2 m/z. Ions with charge states of 2+, 3+ and 4+ were sequentially fragmented by higher energy collisional dissociation, with a normalized collision energy of 37%. In all cases, one microscan was recorded using dynamic exclusion of 30 s. The MS/MS fixed first mass was set at 110.

#### Database searching and quantitative data analysis

Tandem mass spectra were extracted by Proteome Discoverer software (Thermo Fisher Scientific, version 1.4.0.288). Charge state deconvolution and deisotoping were not performed. All MS/MS samples were analyzed using Mascot (Matrix Science, London, UK; version 2.3). Mascot was configured to search the UniProt database (Taxonomy: Rattus norvegicus, 36,076 entries), assuming the digestion enzyme trypsin. For protein identification, mass tolerances of 0.05 Da for intact peptide masses and 0.1 Da for fragmented masses were permitted, with an allowance for one missed cleavage upon trypsin digest. Several parameters in Mascot were configured for peptide searching, including Gln → pyro-Glu (N-term Q), oxidation (M) and deamidated (NQ), as potential variable modifications, and carbamidomethyl (C), TMT 10-plex (N-term) and TMT 10-plex (K), as fixed modifications. The percolator algorithm was used to maintain the peptide-level false discovery rate (FDR) [28] below 1%. A protein containing at least one unique peptide was required for quantitation. The quantitative protein ratios were weighted and normalized by the median ratio in Mascot. Proteins with an absolute fold-change greater than 1.2 and a *p*-value less than 0.05 were considered to be significant DEPs.

#### Bioinformatics and annotations

To determine the biological and functional properties of all of the identified proteins, we employed the hypergeometric test to perform Gene Ontology (GO) enrichment analysis and Kyoto Encyclopedia of Genes and Genomes (KEGG) pathway enrichment analysis using the DAVID system (https://david.ncifcrf.gov/, version 6.8). The default parameters of the DAVID system were used for calculation and analysis. Finally, the statistical significance test FDR < 0.05 was used as a threshold for identifying significantly enriched KEGG pathways and GO terms associated with DEPs.

### Reduced representation bisulfite sequencing

#### DNA sample preparation

Total DNA was extracted using the QIAamp Fast DNA Tissue Kit (Qiagen, Dusseldorf, Germany), following the manufacturer’s instructions. DNA quantity was measured by reading A260/280 ratios using a spectrophotometer. When the A260/280 ratios were located in the 1.8 to 2.0 range, DNA was available.

#### DNA fragmentation and bisulfite conversion

The DNA samples were fragmented using sonication and were then subjected to bisulfite conversion with the EZ DNA Methylation-Gold™ Kit (Zymo Research, USA) following the manufacturer’s instructions. Briefly, add 900 µl water, 50 µl of M-Dissolving Buffer and 300 µl of M-Dilution Buffer to one tube of CT Conversion Reagent and mix for 10 min firstly. Secondly, add 130 µl of the prepared CT Conversion Reagent to 20 µl of DNA sample, performed the following temperature steps: 98 °C for 10 min, 64 °C for 2.5 h, and then hold at 4 °C. Thirdly, add 600 µl of M-Binding Buffer to a Zymo-spin IC Column. After centrifuged at full speed, add 100 µl of M-Wash Buffer to the column, spin 30 s. Fourthly, add 200 µl of M-Desulphonation Buffer to the column and wait for 20 min and spin at full speed for 30 s. And then, add 200 µl of M-Wash Buffer to the column, spin 30 s, repeat this wash step one more time. Finally, add 10 µl of M-Elution Buffer directly to the column matrix, place into a 1.5 ml tube and spin briefly to elute the DNA. According to the steps of this kit, the transformed products were obtained and applied to the ssDNA quantification of nanodrop, and the cycle number of library amplification was determined. Bisulfite-treated ssDNA fragments were used for library construction.

#### Library construction and sequencing

The Accel-NGS Methyl-Seq DNA Library Kit (Swift, MI, USA) was utilized to attach adapters to single-stranded DNA fragments. Briefly, as in the protocol shown below, the adaptase step was a highly efficient, proprietary reaction that simultaneously performed end repair, the tailing of 3′ ends, and the ligation of the first truncated adapter complement with 3′ ends. The extension step was used to incorporate a truncated adapter 1 via a primer extension reaction. The ligation step was used to add the second truncated adapter to the bottom strand only. The indexing PCR step increased the yield and incorporated full-length adapters. Bead-based solid phase reversible immobilization clean-ups were used to remove both oligo nucleotides and small fragments, as well as to change the enzymatic buffer composition. Finally, we performed pair-end 2 × 150 bp sequencing on an Illumina Hiseq 4000 platform housed in the LC Sciences.

#### Bioinformatics analysis

First, Cutadapt and in-house Perl scripts were used to remove reads that contained adapter contamination, low-quality bases and undetermined bases. Then, sequence quality was verified using FASTQC (http://www.bioinformatics.babraham.ac.uk/projects/fastqc/, version 0.10.1). Reads that passed quality control were mapped to the reference genome using WALT [29]. After alignment, reads were further deduplicated using SAM tools [30]. For each cytosine (or guanine corresponding to a cytosine on the opposite strand) in the reference genome sequence, the DNA methylation level was determined as the ratio of the number of reads supporting C (methylated) to the number of total reads (methylated and unmethylated) using in-house Perl scripts and MethPipe [31]. DMRs were calculated using the R package-Methyl Kit [32] with default parameters (1000 bp slide windows, 500 bp overlap, *p*-value < 0.05) and |Fold-change| > 1.2.

### Integrated analysis of the multiomics results

There is a translational relationship between mRNA and protein. Protein, as the translation product of mRNA, performs specific functions. The UniProt database (https://www.uniprot.org/) was used to obtain relationships between mRNAs and proteins to establish an association database. In addition, by integrating DNA methylation data, the mRNA data was annotated with methylated region information. Thus, a table containing three types of omics information was constructed. The tool we used for this integrative analysis was ACGT101-COR (Version 1.1). ACGT101-COR is an in-house pipeline script (LC Sciences, Houston, TX, USA).

### Protein–protein interaction (PPI) networks

Protein–protein interaction information for the DEPs in each selected KEGG pathways (FDR < 0.05) was acquired using the Search Tool for the Retrieval of Interacting Genes (STRING) database (http://www.stringdb.org/). Then, Cytoscape software (version 3.5.1) was used to construct the PPI networks. PPI networks could help us to identify the key genes involved in PAH development, based on interaction relationships.

### Real-time PCR analysis

Total RNA was extracted from PAs using TRIzol. cDNA was synthesized with the Transcriptor First Strand cDNA Synthesis Kit (Roche, Basel, Switzerland), using random oligo (dT) primers. To determine the expression levels of key genes, real-time PCR experiments were performed using the Fast Start DNA Master SYBR Green I Kit and Light Cycler 2.0 (Roche, Basel, Switzerland). Serially diluted solutions of β-actin cDNA-containing plasmids with known copy numbers were used during each PCR experiment to create a linear regression for the analysis of the β-actin standard. All expression levels were normalized to the levels of internal β-actin.

### Western blot analysis

Protein samples were separated on standard 12% SDS-polyacrylamide gels and transferred to a PVDF membrane. After blocking with 5% skim milk in PBS for 2 h, membranes were incubated with antibodies against the proteins, including Col4a1, Itga5, Col2a1, Gstt1, Gstm3 and β-actin, purchased from BOSTER (Wuhan, China), and Thbd, purchased from BIOSS (Beijing, China), overnight at 4 °C, followed by incubation with secondary antibodies, diluted with secondary antibody dilution buffer, at room temperature for 2 h. Immunoreactive bands were detected using enhanced chemiluminescence (ECL) reagent (Thermo, Carlsbad, CA, USA) to visualize the bands, and the optical density of each blot was normalized to that of β-actin and presented as the relative optical density.

### Statistical analysis

Statistical analysis was performed in GraphPad Prism 5 (GraphPad Software Inc., La Jolla, CA) software. Mean responses by group were compared by Student *t*-tests. Comparisons with *p* values less than 0.05 are indicated by asterisks in the diagrams (*p* values less than 0.01 are indicated by two asterisks).

## Results

### Verification of hypoxia-induced PAH models

**Fig. 1:**
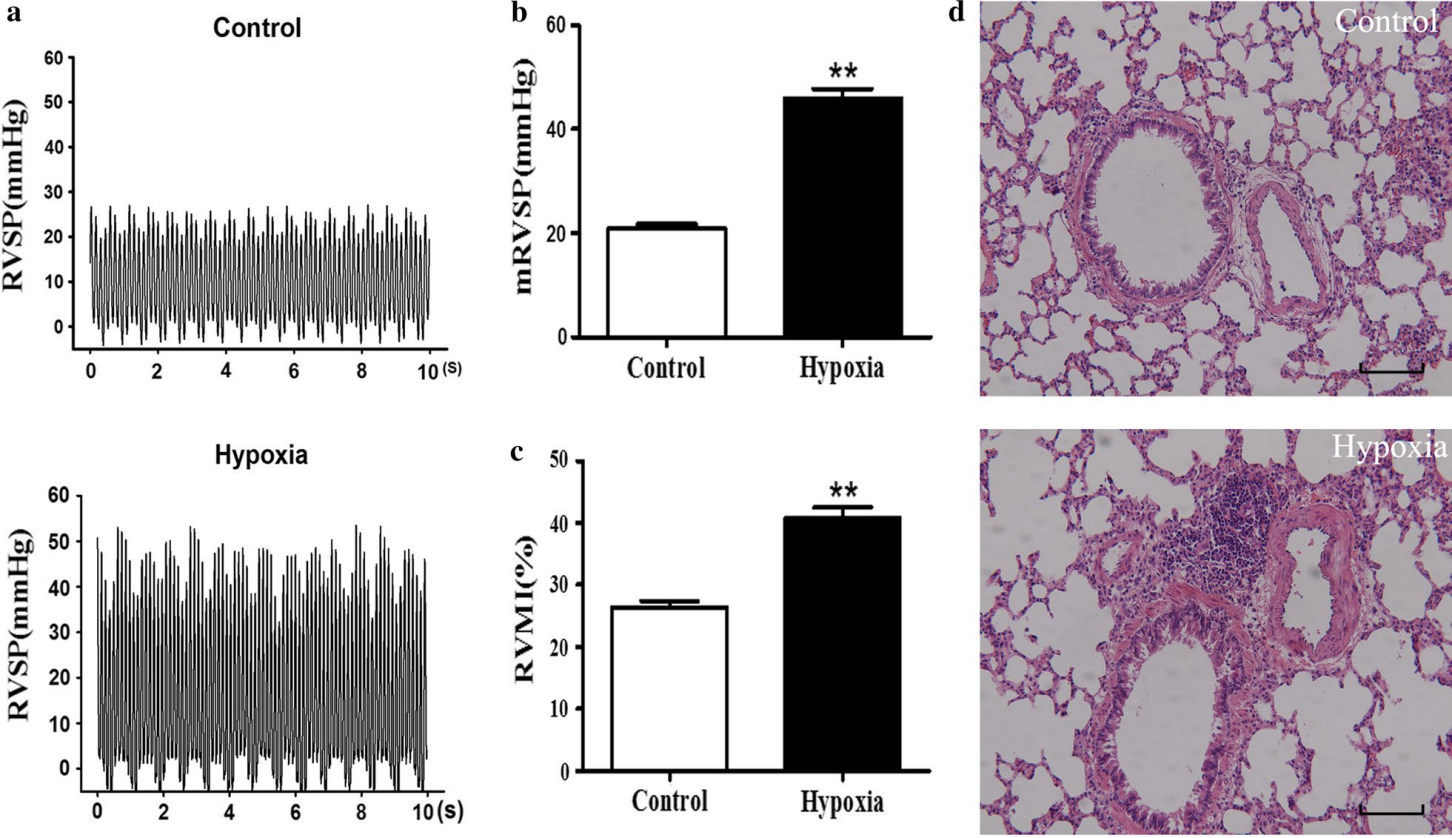
Validation of pulmonary artery hypertension in hypoxia-treated rats. **a**, **b** Representative right ventricular pressure (RVSP) recorded from control and hypoxic rats. **c** Right ventricular mass index (RVMI) showed right ventricular hypertrophy, as indexed by the right ventricle/(left ventricle + ventricular septal) [RV/(LV + S)] mass ratio. **d** Comparison of the PAs from the hypoxic group and with those from the normoxic control group, as assessed by the hematoxylin–eosin staining of paraffin sections

Hypoxic rat models were established and confirmed in this study. RVSP was significant increased from 21.03 ± 0.6966 mmHg (control) to 45.97 ± 1.842 mmHg in the hypoxia-induced rats (Fig. 1a, b). RVMI was approximately 50% higher in hypoxic rats (40.84 ± 1.667%) than in normoxic controls (26.47 ± 0.9474%) (Fig. 1c). Hematoxylin and eosin staining showed that the PAs were thickened, and remodeling was pronounced in hypoxic rats compared with normoxic controls (Fig. 1d). These results indicated that the generation of hypoxia-induced PAH rat model was successful.

### Screening the differential characteristics in PAH

**Fig. 2:**
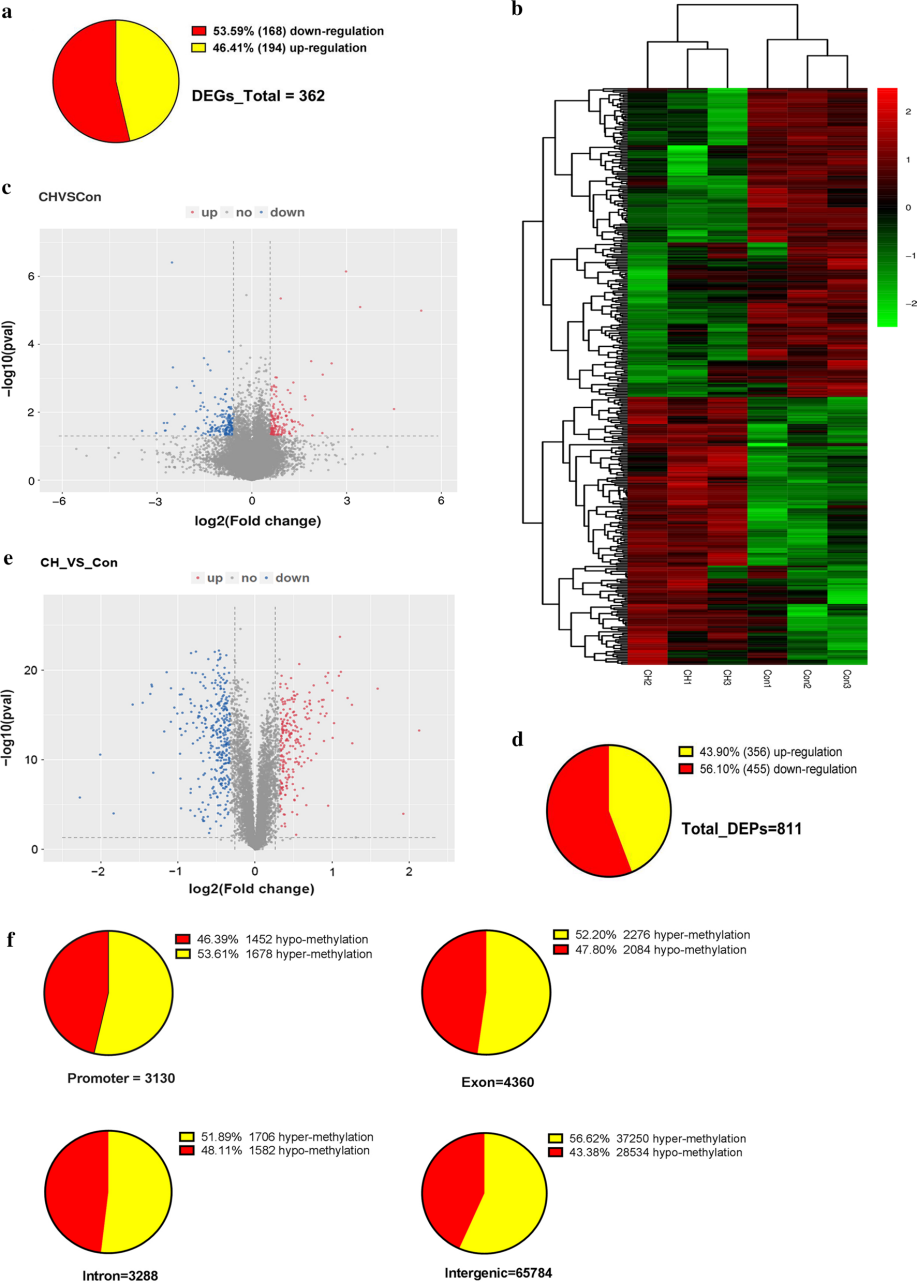
Different characteristics between the hypoxic group and the control group. **a** The pie chart shows the DEGs. **b** Gene expression heatmap of hypoxia versus control groups: unsupervised hierarchical clustering analysis of the significantly dysregulated genes. Red: upregulated genes; green: downregulated genes. **c** Volcano plots for the transcriptome comparisons between the hypoxic group and the control group (Fold change > 1.5 and *p *< 0.05). **d** The pie chart shows the DEPs. **e** Volcano plots for the proteome comparisons between the hypoxic group and the control group (Fold change > 1.25 and *p *< 0.05). **f** The pie chart shows the DMRs, including promoter, exon, intron, and intergenic regions. The pie chart shows the number and proportion of upregulated and downregulated

The PA samples from PAH and normal rats were subjected to high-throughput sequencing, including RNA-seq, TMT and RRBS to identify gene expression, protein expression and epigenetic changes, respectively. The quality control of processed RNA-seq data was performed and shown in Additional file 1: Table S1. DEGs between the hypoxic and control groups were identified by the R-package Ballgown. After filtering (> 1.5-fold change, *p *< 0.05), 362 genes were detected as being significantly differentially expressed (168 up- and 194 downregulated) in the hypoxic group compared with the control group (Fig. 2a). As shown in Fig. 2b, a heatmap of the significantly dysregulated genes depicts a clustered gene expression pattern between the hypoxic and control groups. Volcano plots showed significant differences (as negative log *p* values) in the differential expression means between the PAH and controls (Fig. 2c). Because proteins represent the actual functional molecules, TMT was conducted to investigate the differences in proteomic levels between the two groups. After data filtering (> 1.2-fold change, *p *< 0.05), 811 differentially expressed proteins (356 up- and 455 downregulated) were observed (Fig. 2d). Volcano plots show the differential expression of proteins in Fig. 2e. Meanwhile, the R package-methyl Kit was used to analyze the DMRs with the standard screening criteria (1000 bp windows, 500 bp overlap, *p *< 0.05 and > 1.2-fold change). Figure 2f shows that a total of 76,562 DMRs were identified among promoter (1678 hyper- and 1452 hypomethylated regions), exon (2276 hyper- and 2084 hypomethylated regions), intron (1706 hyper- and 1582 hypomethylated regions) and intergenic (37,250 hyper- and 28,534 hypomethylated regions) areas. Overall, different characteristics between the two groups could be observed through multiomics sequencing.

### Integrated analysis of multiomics characteristics

Based on the information regarding the 811 DEPs, 362 DEGs and 76,562 DMRs, the UniProt database was used to integrate and establish a correlation relationship among the multiple omics analyses. A total of 3991 genes were correlated among the three types of omics information, with *p* values for the difference between PAH and control groups being less than 0.05 for at least one of the omics analyses (the integrated omics analysis ensured that changes in DNA methylation met the standard of fold change > 1.2 and *p *< 0.05). After sorting the fold-changes in protein expression, 10 of the top up- and downregulated proteins in the PAH group are displayed in Table 1. And three omics correlation table was shown in the Additional file 2: Table S2. Taken together, these findings constitute a multiomics library for further study in PAH.

**Table 1:** Top regulated proteins: integrate analysis of three-omics

Correlation			Methylation information							
Gene symbol	mRNA_ID	Protein full name	FC	dir	*p*	FDR	Chr	Info1	Info2	Info3
S100a9	ENSRNOT00000015351	Protein S100-A9	5.8	Up	1.2E−04	1.9E−02	chr2	Intron	First intron	NA
Ttc29	ENSRNOT00000089569	Tetratricopeptide repeat protein 29	0.3	Down	7.9E−05	8.8E−03	chr19	Exon	Internal exon	NA
Bicc1	MSTRG.12240.6	BicC family RNA-binding protein 1	5.2	Up	5.0E−04	4.5E−02	chr20	Intron	Internal intron	NA
Tnc	ENSRNOT00000084563	Tenascin C	0.0	Down	7.1E−07	1.6E−04	chr5	Intron	Internal intron	NA
Runx1	MSTRG.4485.1	Runt-related transcription factor 1	0.6	Down	5.4E−05	8.5E−03	chr11	Intron	First intron	NA
Picalm	ENSRNOT00000092945	Clathrin-assembly lymphoid myeloid leukemia protein	8.7	Up	2.1E−13	1.4E−10	chr1	Intron	Internal intron	NA
Cdyl2	ENSRNOT00000068440	Chromodomain Y-like 2	0.3	Down	1.4E−04	1.4E−02	chr19	Intron	Internal intron	NA
Gfpt2	ENSRNOT00000003770	Glutamine-fructose-6-phosphate aminotransferase 2	0.8	Down	3.9E−05	5.1E−03	chr10	Intron	Internal intron	NA
Folr2	MSTRG.1446.1	Folate receptor 2	0.2	Down	8.7E−05	1.2E−02	chr1	Promoter	Distal	LCP
Slc43a2	MSTRG.3515.3	Solute carrier family 43 member 2	17.7	Up	2.2E−19	3.0E−16	chr10	Intron	Internal intron	NA
Myh6	ENSRNOT00000023302	Myosin-6	2.8	Up	4.8E−05	8.6E−03	chr15	Intron	Internal intron	NA
RGD1563354	ENSRNOT00000019453	Similar to hypothetical protein D630003M21	0.2	Down	5.6E−06	1.1E−03	chr3	Promoter	Distal	LCP
Hist2h2ac	ENSRNOT00000051917	Histone H2A	0.2	down	9.0E−08	3.0E−05	chr2	Promoter	Intermediate	ICP
Map4k2	ENSRNOT00000064798	Mitogen-activated protein kinase	4.9	up	3.4E−06	6.8E−04	chr1	Intron	Internal intron	NA
Gsta3	ENSRNOT00000088416	Glutathione *S*-transferase alpha-3	2.7	up	2.3E−04	9.2E−06	chr9	Exon	Last exon	NA
Pdk4	ENSRNOT00000012760	Pyruvate dehydrogenase kinase, isoenzyme 4	0.2	down	7.9E−09	2.7E−06	chr4	Promoter	Proximal	LCP
Med12 l	ENSRNOT00000084020	Mediator complex subunit 12-like	6.5	Up	1.3E−08	5.0E−06	chr2	Promoter	Distal	LCP
Mapk10	ENSRNOT00000065965	Mitogen-activated protein kinase	2.6	Up	3.6E−06	8.0E−04	chr14	Intron	First intron	NA
Cd63	ENSRNOT00000090381	Ad1-antigen	0.5	Down	3.6E−06	7.0E−04	chr7	Promoter	Intermediate	ICP
Krt1	ENSRNOT00000034450	Keratin, type II cytoskeletal 1	21.8	Up	3.6E−04	3.9E−02	chr7	Promoter	Distal	LCP

Correlation			mRNA information				Protein information			
Gene symbol	mRNA_ID	Protein full name	FC	dir	*p*	FDR	FC	dir	*p*	FDR
S100a9	ENSRNOT00000015351	Protein S100-A9	2.4	Up	0.05	0.82	1.6	Up	6.6E−11	4.5E−10
Ttc29	ENSRNOT00000089569	Tetratricopeptide repeat protein 29	1.0	Down	0.22	0.84	1.5	Up	1.9E−10	1.2E−09
Bicc1	MSTRG.12240.6	BicC family RNA-binding protein 1	0.8	Down	0.38	0.84	1.5	Up	1.2E−05	2.8E−05
Tnc	ENSRNOT00000084563	Tenascin C	2.2	Up	0.36	0.84	1.5	Up	1.5E−12	1.5E−11
Runx1	MSTRG.4485.1	Runt-related transcription factor 1	1.5	Up	0.3	0.84	1.5	Up	3.2E−10	1.9E−09
Picalm	ENSRNOT00000092945	Clathrin-assembly lymphoid myeloid leukemia protein	0.6	Down	0.04	0.82	1.5	Up	1.6E−10	1.0E−09
Cdyl2	ENSRNOT00000068440	Chromodomain Y-like 2	1.1	Up	0.24	0.84	1.4	Up	9.9E−16	2.7E−14
Gfpt2	ENSRNOT00000003770	Glutamine-fructose-6-phosphate aminotransferase 2	1.5	Up	0.1	0.82	1.4	Up	1.7E−17	9.4E−16
Folr2	MSTRG.1446.1	Folate receptor 2	1.4	Up	0.15	0.84	1.4	Up	6.4E−18	4.3E−16
Slc43a2	MSTRG.3515.3	Solute carrier family 43 member 2	1.7	Up	0.35	0.84	1.4	Up	5.1E−08	1.9E−07
Myh6	ENSRNOT00000023302	Myosin-6	1.0	Down	0.31	0.84	0.7	Down	6.8E−16	2.0E−14
RGD1563354	ENSRNOT00000019453	Similar to hypothetical protein D630003M21	1.7	Up	0.06	0.82	0.7	Down	1.6E−11	1.2E−10
Hist2h2ac	ENSRNOT00000051917	Histone H2A	1.1	Up	0.51	0.84	0.7	Down	8.6E−07	2.6E−06
Map4k2	ENSRNOT00000064798	Mitogen-activated protein kinase	1.3	Up	0.08	0.82	0.6	Down	2.4E−14	4.2E−13
Gsta3	ENSRNOT00000088416	Glutathione *S*-transferase alpha-3	0.8	Down	0.31	0.84	0.6	Down	2.9E−15	6.6E−14
Pdk4	ENSRNOT00000012760	Pyruvate dehydrogenase kinase, isoenzyme 4	0.3	Down	0.05	0.82	0.6	Down	5.5E−19	5.8E−17
Med12 l	ENSRNOT00000084020	Mediator complex subunit 12-like	1.0	Down	0.02	0.82	0.6	Down	2.2E−04	4.4E−04
Mapk10	ENSRNOT00000065965	Mitogen-activated protein kinase	1.0	Down	0.41	0.84	0.6	Down	6.7E−04	1.2E−03
Cd63	ENSRNOT00000090381	Ad1-antigen	1.3	Up	0.47	0.84	0.6	Down	4.8E−03	7.7E−03
Krt1	ENSRNOT00000034450	Keratin, type II cytoskeletal 1	0.7	Down	0.02	0.82	0.4	Down	4.2E−17	1.9E−15

Top 10 upregulated and downregulated proteins identified among the three omics analyses. These three omics are correlated in the table, including methylation information, mRNA information and protein information. The fold change (FC), the direction (dir), *p* value (*p*) and FDR of three omics information are shown. Meanwhile, the chromosomal localization (Chr) and methylated positions (Info1–3) are also shown. Info1–3 shows the concrete methylated locations

### Dysregulated biological functions in PAH

**Fig. 3:**
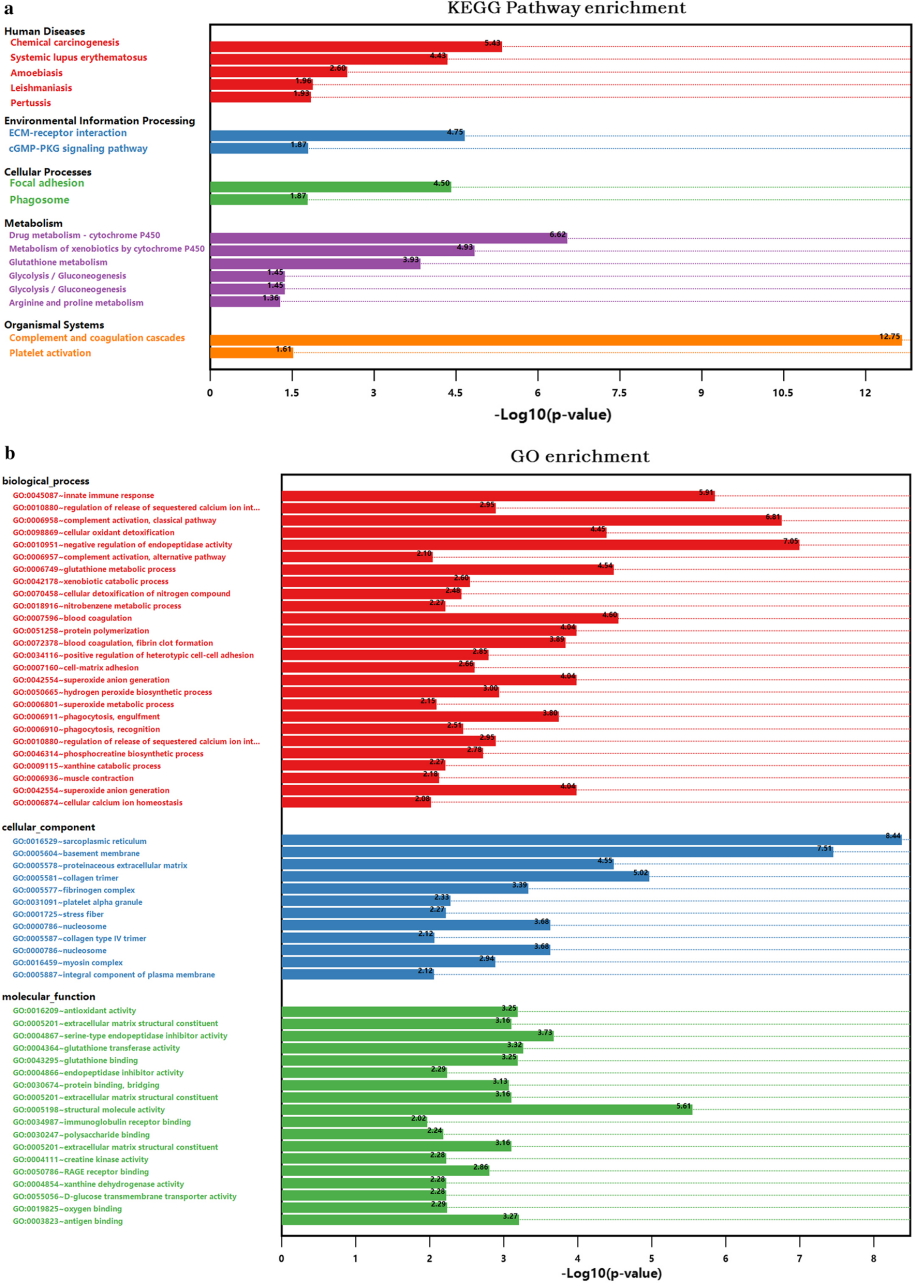

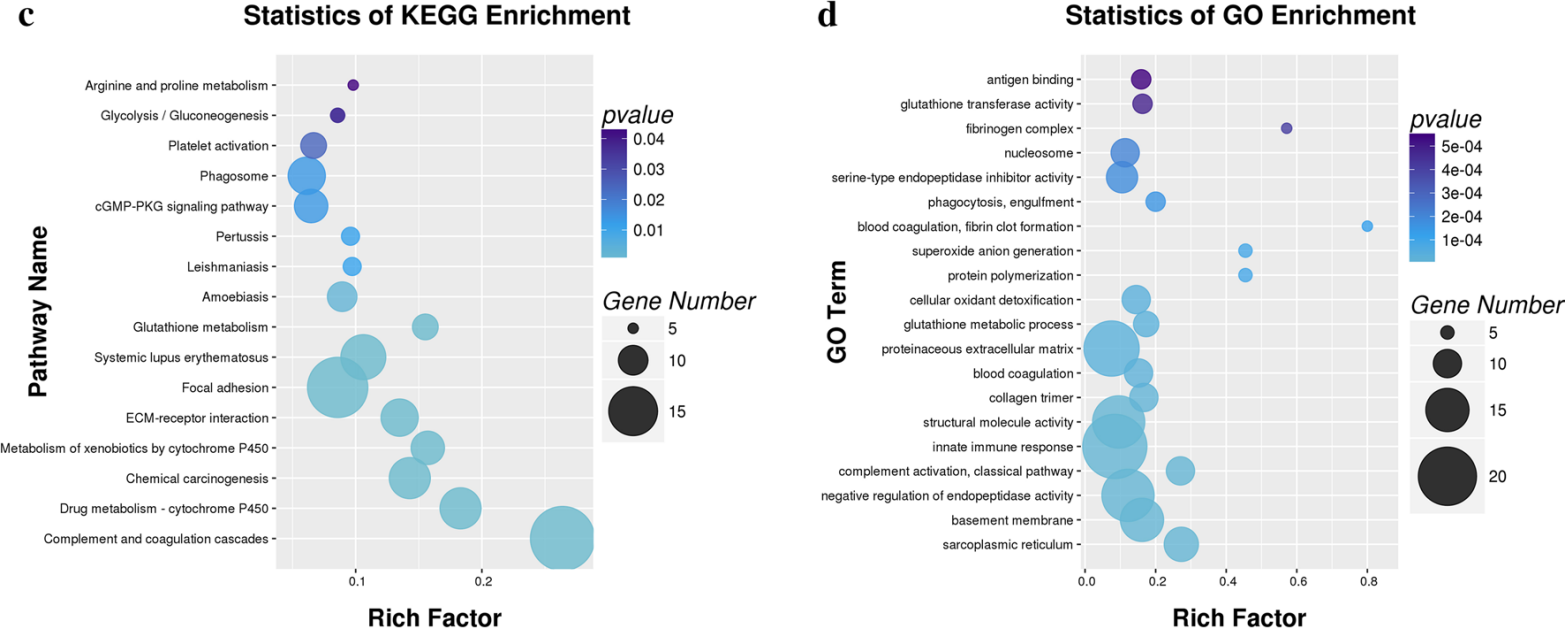
Functional classifications based on pathway enrichment analysis of differentially expressed proteins in the hypoxic group compared with the control group. **a** The KEGG pathway classification of DEPs (*p *< 0.05). **b** GO functional classification of DEPs (*p *< 0.01). **c**, **d** Rich factor of significant KEGG pathways and GO terms

**Fig. 4:**
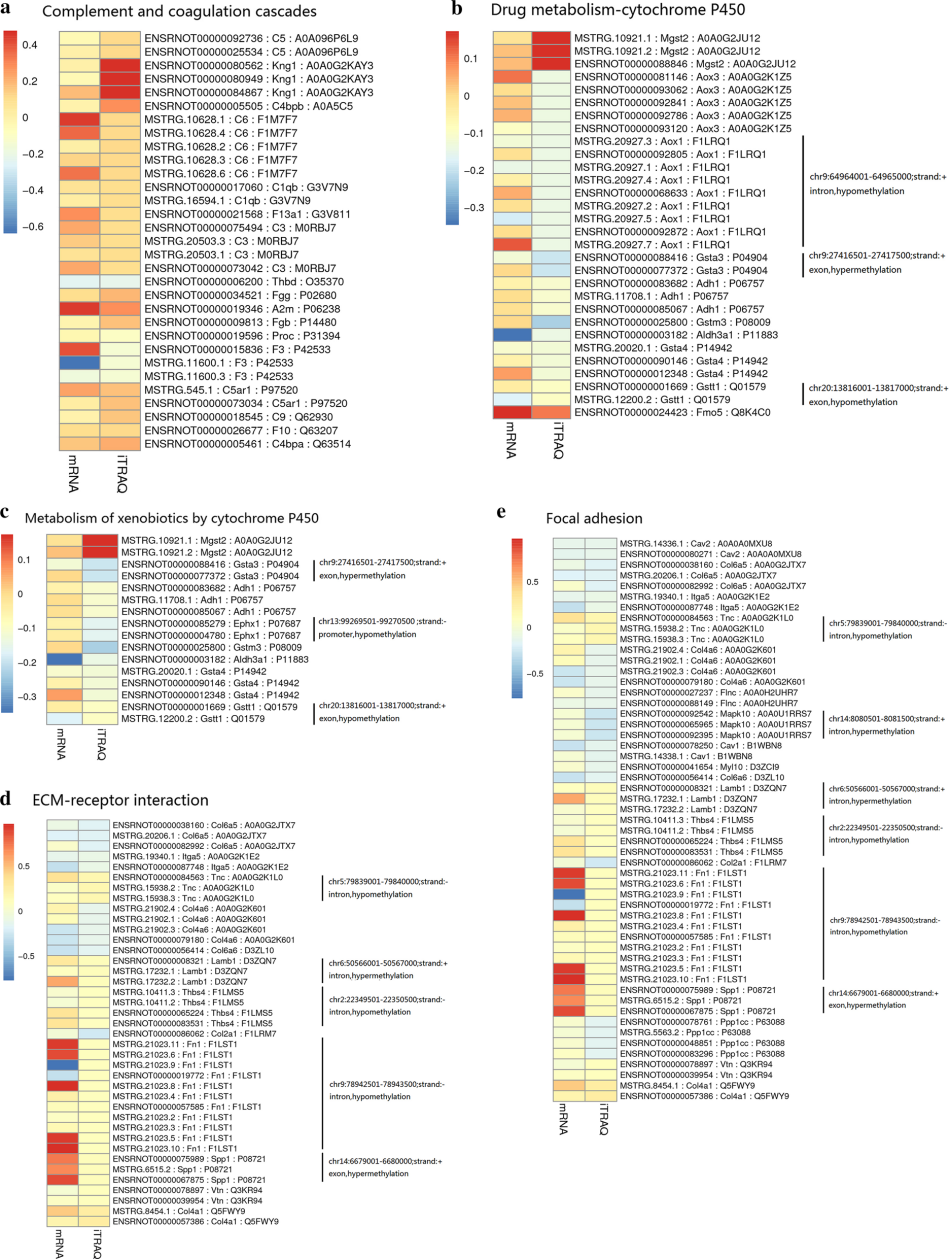
The multiomics information of the selected KEGG pathways. The means of the gene and protein expression levels from the hypoxic and control groups were compared. The data ≥ 0 in the heatmap (slant yellow and red) were upregulated, whereas data < 0 in the heatmap (partial red) were downregulated. Meanwhile, the methylation information, including methylation position, positive or negative chain, and the regulation of methylation are shown to the right of each graph. **a**–**e** represent complement and coagulation cascades, drug metabolism-cytochrome P450, metabolism of xenobiotics by cytochrome P450, ECM-receptor interaction and focal adhesion, respectively

To further identify the novel biological pathways associated with PAH, KEGG and GO analysis were applied. Based on the selected 811 DEPs, 17 KEGG pathways were selected using *p *< 0.05 as the standard (Fig. 3a). Based on a 5% FDR, the 7 KEGG pathways with the lowest *p* values were screened for further analyses, including complement and coagulation cascades (rno04610, *p *= 1.78E−13, FDR = 2.25E−10), drug metabolism-cytochrome P450 (rno00982, *p *= 2.38E−07, FDR = 0.0003), metabolism of xenobiotics by cytochrome P450 (rno00980, *p *= 1.18E−05, FDR = 0.015), extracellular matrix (ECM)-receptor interaction (rno04512, *p *= 1.79E−05, FDR = 0.0227), focal adhesion (rno04510, *p *= 3.18E−05, FDR = 0.0403), chemical carcinogenesis (rno05204, *p *= 3.72E−06, FDR = 0.0047) and systemic lupus erythematosus (rno05322, *p *= 0.0000371824599510135, FDR = 0.047). The chemical carcinogenesis and systemic lupus erythematosus pathways were not included in subsequent analyses, as they are associated with specific human disease pathways. Similarly, GO pathway enrichment analysis was conducted to identify the functional damage terms, and 90 terms were identified using the filter *p *< 0.05 (shown in Additional file 3: Table S3). By raising the screen standard to *p *< 0.01, 56 terms were identified (Fig. 3b). Among these, the top ten terms had FDR < 0.05. The three ontology nodes with the lowest *p* values were sarcoplasmic reticulum (cellular component, GO: 0016529, *p *= 3.67E−09, FDR = 5.04E−06), basement membrane (cellular component, GO: 0005604, *p *= 3.08E−08, FDR = 0.00004) and negative regulation of endopeptidase activity (biologic process, GO: 0010951, *p *= 8.83E−08, FDR = 0.00015). Furthermore, rich factors showed the ratio of the number of DEPs to the number of total proteins in each pathway identified by the KEGG and GO analyses (Fig. 3c, d). More importantly, heatmap analysis was performed for the significant KEGG pathways to show the DEG and DEP information for each pathway. The methylated or unmethylated genes are marked in Fig. 4. This analysis allows the visualization of the multiomics information for the selected key KEGG pathways. Meanwhile, the detailed multiomics information of Fig. 4 is shown in Additional file 4: Table S4.

### PPI network identification of hub node genes

**Fig. 5:**
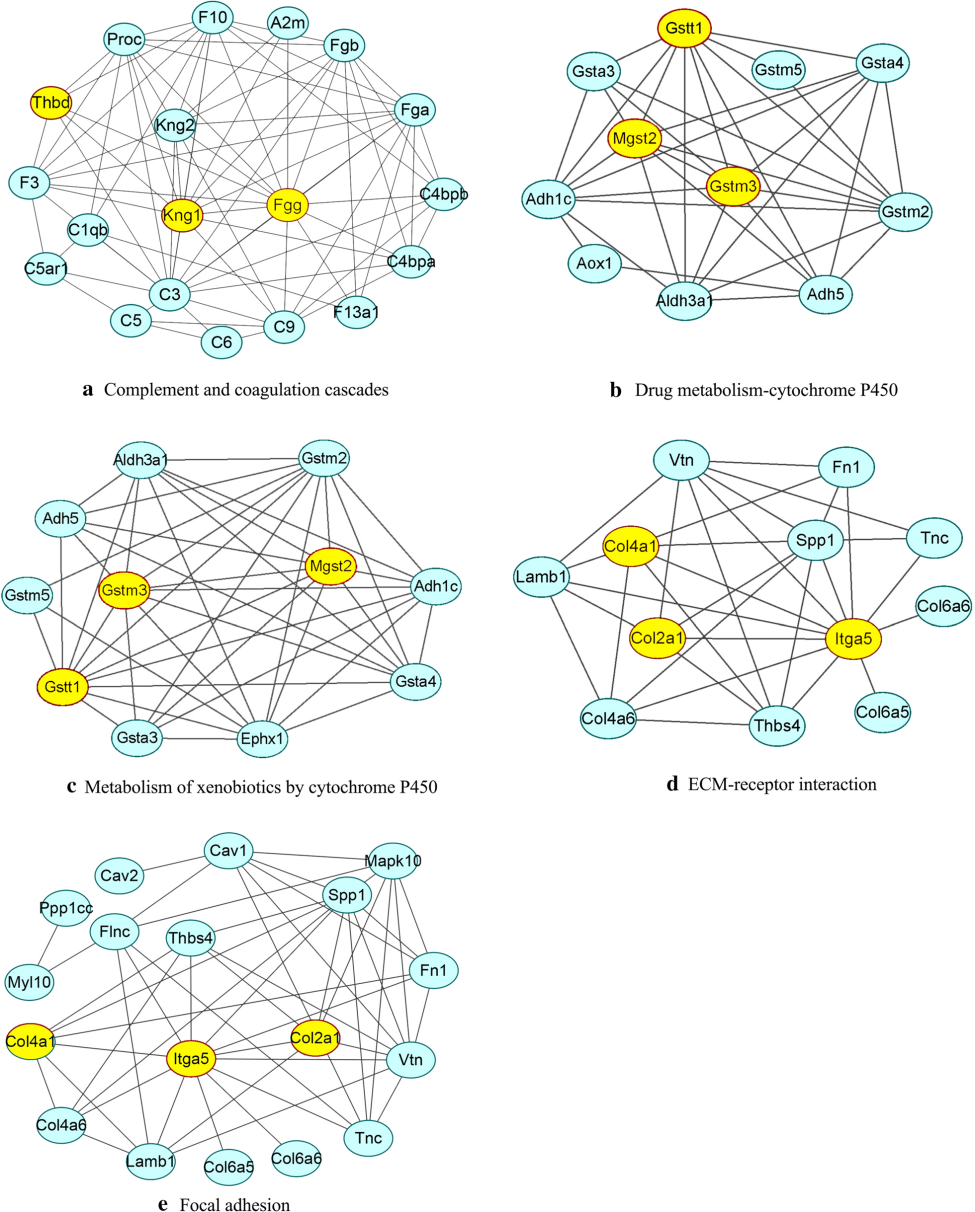
Protein–protein interaction networks. The networks were constructed using the STRING database. Supporting evidence comes from text mining, experiments, databases, coexpression, neighborhood, gene fusion and cooccurrence. **a**–**e** Represent complement and coagulation cascades, drug metabolism-cytochrome P450, metabolism of xenobiotics by cytochrome P450, ECM-receptor interaction and focal adhesion, respectively. The hub node genes validated subsequently, are labeled in yellow

As shown in Fig. 5, the genes of the five KEGG pathways identified above were used to construct protein–protein interaction networks using the STRING database, respectively. The genes with more joint-edges have more important biological functions [33]. Therefore, genes those have more interaction relationships with other genes may play important roles in the process of PAH. In our study, the degree of the genes, which is the number of joint-edges, was calculated for each selected KEGG pathways (Table 2). Then, the hub genes were labeled yellow in the Fig. 5, which had higher degrees were selected for further verification.

**Table 2:** The degrees of gene and the expression fold change of proteins in each significant KEGG pathway (pathway enriched FDR < 0.05)

KEGG-pathway	Gene symbol	Name	Degree	FC
ECM-receptor interaction	*Itga5*	*Integrin subunit alpha 5*	*11*	*0.79*
	Spp1	Secreted phosphoprotein 1	8	1.32
	Vtn	Vitronectin	7	1.28
	Thbs4	Thrombospondin 4	6	1.26
	*Col4a1*	*Collagen, type IV, alpha 1*	*6*	*1.88*
	Lamb1	Laminin, beta 1	5	1.31
	Col4a6	Collagen, type IV, alpha 6	5	0.78
	*Col2a1*	*Collagen, type II, alpha 1*	*5*	*0.58*
	Fn1	Fibronectin 1	4	1.27
	Tnc	Tenascin C	3	1.49
	Col6a6	Collagen, type VI, alpha 6	1	0.72
	Col6a5	Collagen, type VI, alpha 5	1	0.69
Focal adhesion	*Itga5*	*Integrin subunit alpha 5*	*12*	*0.79*
	Spp1	Secreted phosphoprotein 1	10	1.32
	Vtn	Vitronectin	9	1.28
	Cav1	Caveolin 1	7	0.73
	Mapk10	Mitogen activated protein kinase 10	7	0.63
	*Col2a1*	*Collagen, type II, alpha 1*	*6*	*0.58*
	*Col4a1*	*Collagen, type IV, alpha 1*	*6*	*1.88*
	Flnc	Filamin C, gamma	6	0.75
	Fn1	Fibronectin 1	6	1.27
	Lamb1	Laminin, beta 1	6	1.31
	Thbs4	Thrombospondin 4	6	1.26
	Tnc	Tenascin C	6	1.49
	Col4a6	Collagen, type IV, alpha 6	5	0.78
	Myl10	Myosin, light chain 10, regulatory	2	0.77
	Cav2	Caveolin 2	1	0.76
	Col6a5	Collagen, type VI, alpha 5	1	0.69
	Col6a6	Collagen, type VI, alpha 6	1	0.72
	Ppp1cc	Protein phosphatase 1 catalytic subunit gamma	1	0.72
Drug metabolism-cytochrome P450	Gstm2	Glutathione *S*-transferase mu 2	9	0.73
	*Gstt1*	*Glutathione S-transferase theta 1*	*9*	*0.80*
	Adh1c	Alcohol dehydrogenase 1C (class I)	8	0.78
	*Gstm3*	*Glutathione S-transferase mu 3*	*8*	*0.59*
	*Mgst2*	*Microsomal glutathione S-transferase 2*	*8*	*1.50*
	Adh5	Alcohol dehydrogenase 5 (class III)	7	0.78
	Aldh3a1	Aldehyde dehydrogenase 3 family, member A1	7	0.72
	Aox3	Aldehyde oxidase 3	0	0.73
	Gsta4	Glutathione *S*-transferase alpha 4	7	0.76
	Gsta3	Glutathione *S*-transferase alpha 3	5	0.64
	Aox1	Aldehyde oxidase 1	2	0.73
	Gstm5	Glutathione *S*-transferase, mu 5	2	0.67
	Fmo5	Flavin containing monooxygenase 5	0	1.27
Metabolism of xenobiotics by cytochrome P450	Gstm2	Glutathione *S*-transferase mu 2	10	0.73
	Gstt1	Glutathione *S*-transferase theta 1	10	0.80
	Ephx1	Epoxide hydrolase 1	9	0.69
	*Gstm3*	*Glutathione S-transferase mu 3*	*9*	*0.59*
	*Mgst2*	*Microsomal glutathione S-transferase 2*	*9*	*1.50*
	Adh1c	Alcohol dehydrogenase 1C (class I)	8	0.78
	Aldh3a1	Aldehyde dehydrogenase 3 family, member A1	8	0.72
	Gsta4	Glutathione *S*-transferase alpha 4	8	0.76
	Adh5	Alcohol dehydrogenase 5 (class III)	6	0.78
	Gsta3	Glutathione *S*-transferase alpha 3	6	0.64
	Gstm5	Glutathione *S*-transferase, mu 5	3	0.67
Complement and coagulation cascades	C3	Complement C3	16	1.26
	*Fgg*	*Fibrinogen gamma chain*	*14*	*1.57*
	Fga	Fibrinogen alpha chain	12	1.60
	*Kng1*	*Kininogen 1*	*12*	*3.01*
	F10	Coagulation factor X	11	1.32
	Fgb	Fibrinogen beta chain	10	1.49
	Kng2	Kininogen 2	10	1.80
	Proc	Protein C	10	0.79
	C9	Complement component 9	9	1.44
	F3	Coagulation factor III	9	0.71
	C4bpa	Complement component 4 binding protein, alpha	7	1.64
	C4bpb	Complement component 4 binding protein, beta	7	1.90
	*Thbd*	*Thrombomodulin*	*6*	*0.62*
	C1qb	Complement C1q B chain	5	1.25
	C5	Complement component 5	4	1.29
	C5ar1	Complement C5a receptor 1	4	1.47
	F13a1	Coagulation factor XIII A1 chain	4	1.27
	A2m	Alpha-2-macroglobulin	3	1.93
	C6	Complement component 6	3	1.31
	Kng2l1	Kininogen 2-like 1	0	1.55

The KEGG pathway name, gene symbol, gene name, the degree of gene in the PPI network and the FC of proteins are shown. These genes validated subsequently, are highlighted in italic

### Verification of the key genes

**Fig. 6:**
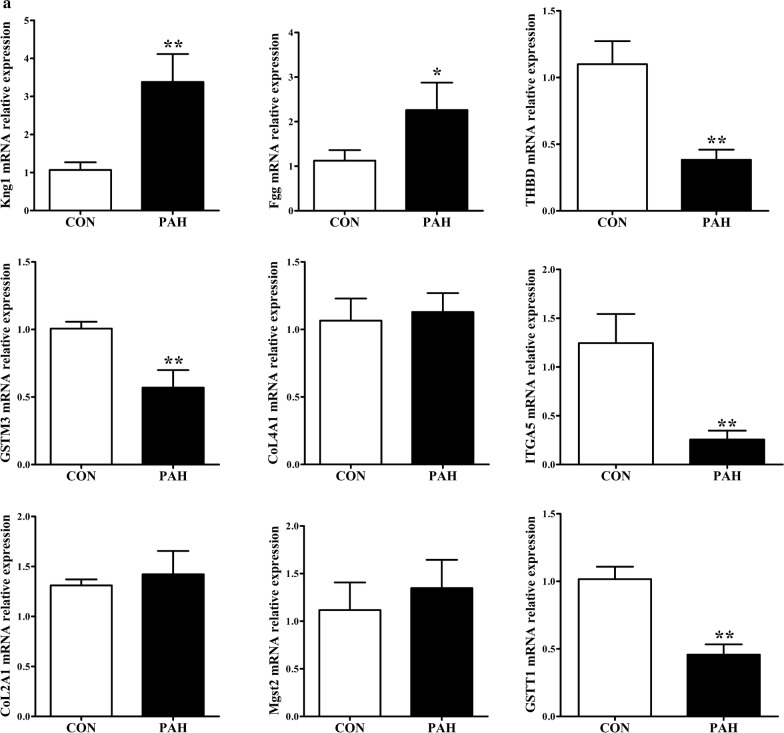

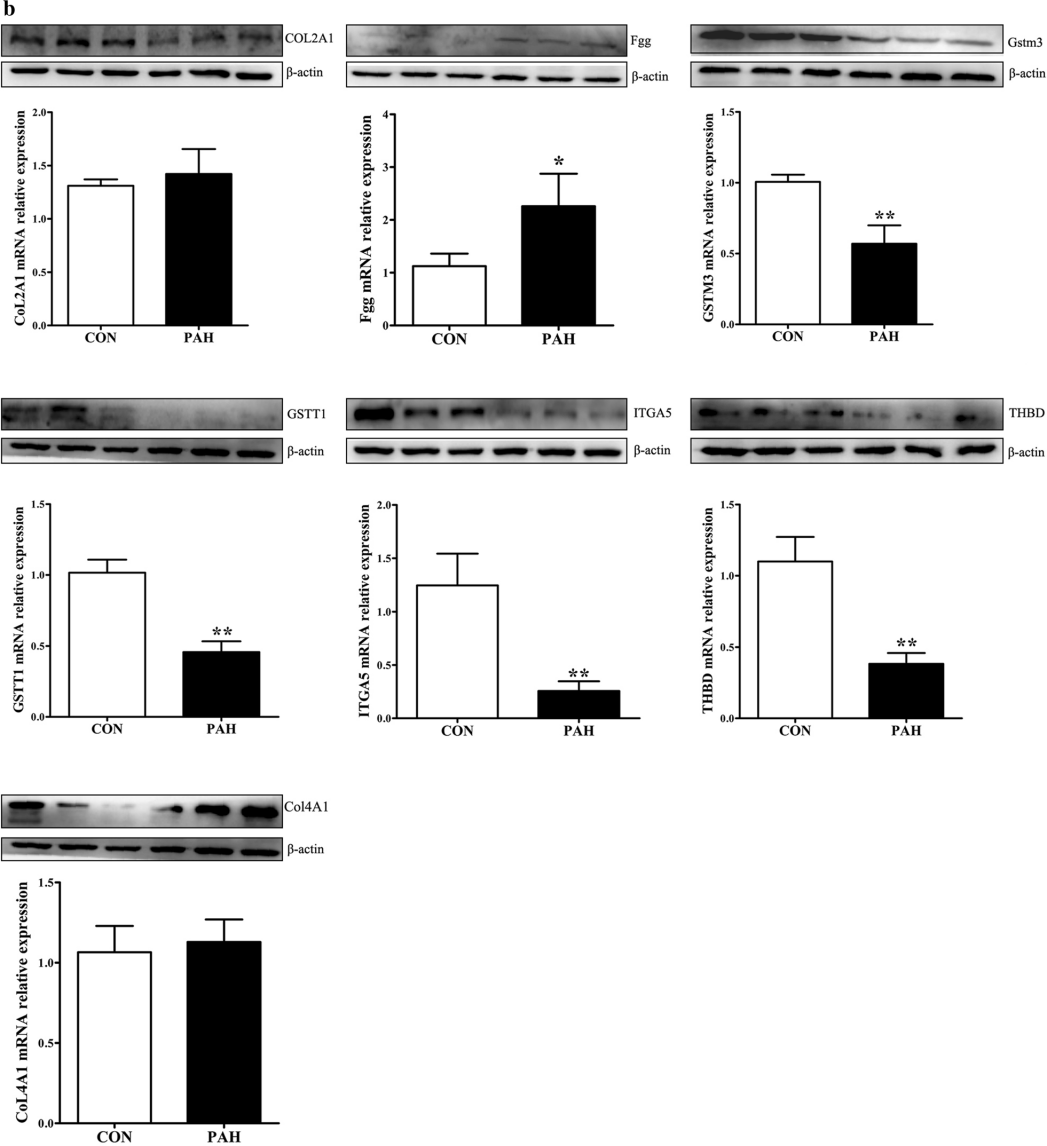
Validation of candidate gene expression with real-time PCR and Western blot analyses. Data were normalized to β-actin and are presented as means with confidence intervals, *p < 0.05, **p < 0.01. **a** The relative mRNA expression levels of Col2a1, Col4a1, Fgg, Gstm3, Gstt1, Itga5, Kng1, Mgst2 and Thbd. **b** Validation of the protein expression levels with Western blot analysis

According to the degree of the genes in the PPI and the expression fold-change of their associated proteins, nine key genes were verified by real-time PCR analyses. Mgst2, Kng1, Fgg, Col4a1 and Col2a1 were upregulated, whereas Itga5, Gstt1, Gstm3 and Thbd were downregulated in PAs from the PAH group compared with those from the control group. The mRNA levels of these genes tended to be consistent with the sequencing results, except for Col2a1. Itga5, Gstm3, Thbd and Gstt1 were significantly downregulated (*p *< 0.01), and Kng1 was significantly upregulated (*p *< 0.01) in PAs from the PAH group compared with those from the control group (Fig. 6a). Then, we performed Western blot analyses of these genes to identify protein level changes. As expected, the protein expression levels were consistent with the sequencing results. Fgg and Col4a1 were significantly upregulated, while Itga5, Gstt1, Gstm3, Col2a1 and Thbd were significantly downregulated in PAs from the PAH group compared with those from the control group (all *p *< 0.01) (Fig. 6b). Together, verification using real-time PCR and Western blot further indicated that the sequencing results were reliable.

## Discussion

In this study, we identified novel dysregulated genes and pathways in hypoxia-induced PAH. Using high-throughput sequencing and the integrated analysis of the mRNA expression, protein expression and DNA methylation data, we established DNA methylation, mRNA and protein profiles of the same samples to investigate the complexity of gene dysregulation in PAH. To date and to the best of our knowledge, only three studies have previously investigated compartment-specific gene expression patterns in the PAs from PAH models using high-throughput screening [8, 10, 11]. In our study, PAs were used for the experiments. RNA-seq, TMT and RRBS were combined to screen the different characteristics of the PAH group compared with the control group. This is the first study to integrate multiomics characteristics to create PA profiles for hypoxia-induced PAH models.

In our study, seven KEGG pathways and ten GO terms were selected by KEGG pathway enrichment and GO enrichment analyses, respectively. Complement and coagulation cascades was the top significantly regulated KEGG pathway identified in the PAH group. A previous study exploited an iTRAQ-based proteomic method to investigate the therapeutic actions and associated mechanisms of osthole in rats with PAH induced by experimental monocrotaline (MCT). This study reported that the complement and coagulation cascade pathway was significantly disordered in PAH lungs [4]. In our sequencing data, within this pathway, the Kng1 and Thbd genes were the most significantly upregulated and downregulated genes, respectively. Interestingly, the changes in Kng1 and Thbd expression were consistently validated by real-time PCR and Western blotting analyses in our study. According to previous studies, Kng1 can influence the activated partial thromboplastin time and the risk of thrombosis [34] as well as key components of the renin–angiotensin–aldosterone system, which is critical for the regulation of blood pressure and fluid balance and influences cardiovascular remodeling [35]. In this regard, Thbd might slightly delay the progression of MCT-induced PAH [36]. PAs were utilized in compartment-specific gene expression patterns in three previous studies. Some of these results were consistent with our data. Hoffmann et al. reported gene expression data from patients with chronic obstructive pulmonary disease and idiopathic pulmonary fibrosis (IPF) with pulmonary hypertension. They found that the retinol metabolism and ECM receptor interaction were the most perturbed processes [8]. In our results, the *p*-values for these two pathways were 0.043 and 1.79E−05, respectively. In addition, several genes, including Tnc (upregulated), Thbs2 (upregulated) and Vwf (downregulated), were reported to be involved in ECM receptor interaction, in agreement with our data. Laumanns et al. [11] performed transcriptome-wide expression profiling of laser microdissected PA resistance vessels derived from explanted idiopathic PAH and nontransplanted donor lung tissues. Consistent with our data, the focal adhesion and ECM-receptor interaction pathways were found to be significant pathways. In our results, the top regulated genes from the focal adhesion and ECM-receptor interaction pathways were Col4a1 and Col2a1. In addition, Itga5 was identified as hub node for the PPI networks of these two pathways. Functionally, Col2a1 has been reported to be involved in the arterial tortuosity syndrome, which is often associated with PA stenosis and pulmonary hypertension [37]. Col4a1 was associated with several vascular defects, including arterial stiffness and myocardial infarction [38, 39]. Similarly, Itga5 has been shown to enhance cell adhesion, cell viability, cell migration and nitric oxide production [40]. Patel et al. [10] compared pulmonary arteriole gene expression from 16 IPF patients, including eight with PAH (PAH-IPF) and eight without PAH (NPAH-IPF), and seven controls. They found that the expression levels of Stat1 and Smoc2, a transcription factor and an extracellular protein, respectively, associated with vascular proliferation, were increased in IPF arteriole, which was validated with immunohistochemical staining. Meanwhile, in our data, STAT1 and SMOC2 were significantly upregulated at both the mRNA and protein levels. The above three studies of PAs were conducted on human beings. Interestingly, there was a great deal of consistency between their results and ours. Therefore, our results are more persuasive and have a certain clinical significance. Two other significantly regulated pathways were drug metabolism-cytochrome P450 and metabolism of xenobiotics by cytochrome P450. Xet et al. indicated that the activation of the drug metabolism-cytochrome P450 pathway in cardiomyocytes through derived endothelial cell crosstalk could be crucial for cardioprotection under oxidative stress conditions [41]. Cytochrome P450 has been shown to be prevalent in inflammation-related disorders, including cardiovascular disease [42]. Overall, these results indicate that the genes related to our selected pathways might play important roles in the formation processes involved in hypoxia-induced PAH.

Genes in the selected KEGG pathways contained methylation information, included Tnc, Fn1, Lamb1, Thbs4, Spp1, Mapk10, Aox1, Gsta3, Gstt1 and Ephx1. The methylated genes were associated with proliferation or apoptosis and the regulation of a range of cardiovascular diseases. Tnc promotes glioblastoma invasion and negatively regulates tumor proliferation [43]. Meanwhile, Tnc has been associated with worse left ventricular remodeling and long-term outcomes in dilated cardiomyopathy cases [44]. Wang et al. [45] demonstrated an essential role for the localized synthesis of Fn1 during cardiovascular development and the spatial regulation of Notch signaling. Overall, these methylated genes were generally indirectly correlated with the development of PAH, and there have been few studies on these genes during PAH. Epigenetic factors also play important roles in the formation and development of PAH, and these methylated genes may serve as guides for further research into the pathogenesis of PAH.

One limitation of this study is that the experiments were performed only at the animal level. These results have not been validated by the examination of PAs from human case samples. However, previous studies on human samples have shown a great deal of consistency with our results, confirming our results and demonstrating that our data have clinical significance. An integrated analysis of mRNA and protein levels can provide more comprehensive information to better understand gene regulation [46]. Moreover, we integrated methylation information to achieve a more profound understanding of hypoxia-induced PAH and to reveal the pathogenic mechanisms of PAH. In the future, the use of omics and systems biology approaches should be extended to fully identify the more detailed pathogenic mechanisms of key genes and pathways. Another limitation of this study is that sex differences were not considered. The basic blood pressure of male and female rats is different, as is the incidence of disease. In this study, we used male rats to establish PAH models which were agreed with previous publications [47–49].

## Conclusions

In summary, we have identified key genes and potential pathways associated with the development of PAH through pathway enrichment and PPI analyses, which may play important roles during pulmonary vascular remodeling and pulmonary hypertension. Our findings provide a general overview that integrates the dysfunctional characteristics associated with PAH as determined by three omics analyses. This study has contributed to the understanding of the injury mechanisms underlying PAH and to the possible development of new drugs and therapeutic targets for PAH.

## Additional files

**Additional file 1: Table S1.** The table summary of RNA-seq quality control data.

**Additional file 2: Table S2.** Integrated analysis of three omics characteristics.

**Additional file 3: Table S3.** GO enrichment analysis ( *p*-value < 0.05).

**Additional file 4: Table S4.** The detailed information of the significant KEGG pathways.

## Data Availability

The data generated or analyzed during this study are available from the corresponding author upon reasonable request.

## Abbreviations

PAH: pulmonary artery hypertension
mPAP: mean pulmonary artery pressure
PAs: pulmonary arteries
SD: Sprague–Dawley
RNA-seq: RNA sequencing
TMT: tandem mass tags
RRBS: reduced representation bisulfite sequencing
DEGs: differentially expressed genes
DEPs: differentially expressed proteins
DMRs: differentially methylated regions
RVSP: right ventricle systolic pressure
RVMI: right ventricular mass index
HPLC: high performance liquid chromatography
MS/MS: tandem mass spectrometry
LC: liquid chromatography
MS: mass spectrometry
AGC: automatic gain control
FDR: false discovery rate
GO: Gene Ontology
KEGG: Kyoto Encyclopedia of Genes and Genomes
PPI: protein–protein interaction
STRING: Search Tool for the Retrieval of Interacting Genes
MCT: monocrotaline
IPF: idiopathic pulmonary fibrosis
