# Supplementary material for: Revealing the pathogenic changes of PAH based on multiomics characteristics
Source: J Transl Med. 2019 Jul 22;17:231. doi: 10.1186/s12967-019-1981-5 (PMC6647123; doi:10.1186/s12967-019-1981-5)
Supplement: Supplementary file 1 — Additional file 1: Table S1. The table summary of RNA-seq quality control data. [file 12967_2019_1981_MOESM1_ESM.docx]

**Table S1.** The table summary of RNA-seq quality control data. The sequencing data and reference genome are compared with reads statistics, including valid reads, mapped reads, unique mapped reads, muliti mapped reads, PE mapped reads, reads map to sense stand, reads map to antisense stand, non-splice reads, splice reads. The parameters are described in the table.

**Table S1. The table summary of RNA-seq quality control data**

| **Sample** | **Valid reads** | **Mapped reads** | **Unique Mapped reads** | **Multi Mapped reads** | **PE Mapped reads** | **Reads map to sense strand** | **Reads map to antisense strand** | **Non-splice reads** | **Splice reads** |
| --- | --- | --- | --- | --- | --- | --- | --- | --- | --- |
| Con1 | 4.8E+07 | 45167761(94.22%) | 30984315(64.63%) | 14183446(29.59%) | 42655810(88.98%) | 20913850(43.62%) | 21025282(43.86%) | 26773926(55.85%) | 15165206(31.63%) |
| Con2 | 5.3E+07 | 50269963(95.52%) | 37277011(70.83%) | 12992952(24.69%) | 48075464(91.35%) | 23294886(44.26%) | 23405779(44.47%) | 30520252(57.99%) | 16180413(30.74%) |
| Con3 | 3.9E+07 | 37195062(95.84%) | 27682252(71.33%) | 9512810(24.51%) | 35494004(91.45%) | 17228417(44.39%) | 17305216(44.59%) | 22419491(57.77%) | 12114142(31.21%) |
| CH1 | 5.4E+07 | 51501357(95.60%) | 38320728(71.13%) | 13180629(24.47%) | 49059572(91.06%) | 23844306(44.26%) | 23936701(44.43%) | 30569220(56.74%) | 17211787(31.95%) |
| CH2 | 4.8E+07 | 45976558(94.97%) | 33908748(70.04%) | 12067810(24.93%) | 43762876(90.40%) | 21403100(44.21%) | 21486592(44.38%) | 27349801(56.49%) | 15539891(32.10%) |
| CH3 | 5.3E+07 | 50279527(95.65%) | 37377514(71.11%) | 12902013(24.54%) | 48137980(91.58%) | 23361875(44.44%) | 23438342(44.59%) | 29200222(55.55%) | 17599995(33.48%) |

**Table parameter description:**

| **Term** | **Meaning** |
| --- | --- |
| Sample | Name of sequencing library |
| Valid reads | Data after quality control |
| Mapped reads | The number of Reads that can be matched to the genome |
| Unique Mapped reads | The number of reads that can be uniquely matched to one location in the genome |
| Multi mapped reads | The number of reads that can be aligned to multiple locations in the genome |
| PE Mapped reads | Pairwise localization of Pair-end sequencing reads to reads on the genome |
| Reads map to sense strand | Reads map to sense strand |
| Reads map to antisense strand | Reads map to antisense strand |
| Non-splice reads | Read is capable of end-to-end alignment to genomic regions |
| Splice reads | Read does not end-to-end alignment to the genome region |
