# Supplementary material for: Revealing the pathogenic changes of PAH based on multiomics characteristics
Source: J Transl Med. 2019 Jul 22;17:231. doi: 10.1186/s12967-019-1981-5 (PMC6647123; doi:10.1186/s12967-019-1981-5)
Supplement: Supplementary file 2 — Additional file 2: Table S2. Integrated analysis of three omics characteristics. [file 12967_2019_1981_MOESM2_ESM.pdf]

Table S2. Integrated analysis of three omics characteristics. Three omics correlated information, including methylation, mRNA and protein information. The fold change (FC), the significance of FC (sig-FC), the direction (dir), p value (*p*) and the significance of p value (sig-*p*) of three omics information are shown. Meanwhile, The chromosomal localization (Chr) and methylated positions (Info1-3) are also shown in methylation information. Info1-3 shows the concrete methylated locations.

Table S2. Integrated analysis of three omics characteristics

Correlation	Methylation information									mRNA information					protein information				
**mRNA : Gene : Protein**	**FC**	**sig_FC**	**dir**	***p***	**sig_p**	**chr**	**Info1**	**Info2**	**Info3**	**FC**	**sig_FC**	**dir**	***p***	**sig_p**	**FC**	**Sig_FC**	**dir**	***p***	**sig_p**
ENSRNOT00000001916 : Serpine1 : F1LM16	2.28	yes	up	2.6E-04	yes	chr12	exon	last exon	NA	0.43	yes	down	0.33	no	1.00	no	down	7.7E-01	no
ENSRNOT00000084660 : Ctnna3 : F1M4I1	0	yes	down	1.3E-06	yes	chr20	promoter	Distal	LCP	1.09	no	up	0.69	no	0.92	no	down	3.6E-04	yes
ENSRNOT00000076259 : Dopey2 : A0A0G2JXD9	3.39	yes	up	1.3E-40	yes	chr11	intron	internal intron	NA	0.97	no	down	0.80	no	0.98	no	down	3.1E-01	no
MSTRG.17118.5 : Atad2b : A0A096MKA5	3.51	yes	up	9.0E-07	yes	chr6	intron	internal intron	NA	0.98	no	down	0.28	no	1.15	no	up	9.1E-06	yes
ENSRNOT00000039273 : Zc3h18 : Q6TQE1	0.03	yes	down	1.2E-11	yes	chr19	promoter	Intermediate	LCP	1.15	no	up	0.10	no	1.02	no	up	2.3E-01	no
MSTRG.22038.4 : Fmr1 : Q80WE1	inf	yes	up	3.0E-04	yes	chrX	intron	internal intron	NA	1.35	no	up	0.40	no	1.09	no	up	7.3E-06	yes
ENSRNOT00000048140 : Dhx8 : A0A0G2K283	0.28	yes	down	3.7E-05	yes	chr10	intron	internal intron	NA	0.99	no	down	0.59	no	0.94	no	down	1.2E-01	no
ENSRNOT00000030065 : Tmem62 : D3ZIW4	0.44	yes	down	9.9E-05	yes	chr3	exon	last exon	NA	2.42	yes	up	0.22	no	1.03	no	up	1.0E-01	no
MSTRG.2596.6 : Gbf1 : A0A0G2K3N1	0.28	yes	down	1.1E-05	yes	chr1	intron	internal intron	NA	1.00	no	down	0.40	no	1.05	no	up	6.1E-10	yes
MSTRG.21909.1 : Acsl4 : O35547	0.19	yes	down	1.2E-04	yes	chrX	intron	internal intron	NA	1.08	no	up	0.77	no	1.22	yes	up	2.0E-10	yes
MSTRG.3645.2 : Slfn13 : A0A096MKD0	2.27	yes	up	1.5E-04	yes	chr10	promoter	Intermediate	LCP	1.29	no	up	0.60	no	0.97	no	down	2.7E-02	yes
MSTRG.21491.5 : Phf8 : D4AD31	0	yes	down	2.1E-07	yes	chrX	exon	first exon	NA	1.53	no	up	0.44	no	1.13	no	up	3.6E-02	yes
MSTRG.7561.2 : Phf11b : M0RB46	3.03	yes	up	2.9E-04	yes	chr15	promoter	Intermediate	LCP	2.06	yes	up	0.22	no	1.15	no	up	2.3E-06	yes
MSTRG.8345.6 : Fgfr1 : F1LM54	0.22	yes	down	3.0E-05	yes	chr16	exon	last exon	NA	1.19	no	up	0.69	no	1.05	no	up	1.3E-02	yes
ENSRNOT00000023418 : Lrba : A0A0G2JYI0	10.95	yes	up	4.8E-12	yes	chr2	intron	internal intron	NA	1.00	no	down	0.99	no	0.94	no	down	4.5E-07	yes
MSTRG.16232.1 : Hectd3 : F1LVZ9	3.28	yes	up	3.1E-04	yes	chr5	intron	internal intron	NA	0.91	no	down	0.89	no	0.99	no	down	6.3E-01	no
MSTRG.19735.1 : Atm : A0A0G2K3I0	0.65	yes	down	2.4E-04	yes	chr8	intron	internal intron	NA	1.01	no	up	0.48	no	1.01	no	up	4.6E-01	no
ENSRNOT00000032248 : Tek : D3ZCD0	inf	yes	up	4.6E-07	yes	chr5	intron	internal intron	NA	0.69	no	down	0.11	no	0.98	no	down	7.9E-02	no
MSTRG.10517.3 : Erbin : M0R9T2	3.86	yes	up	2.1E-05	yes	chr2	intron	last intron	NA	2.20	yes	up	0.02	yes	0.94	no	down	3.9E-05	yes
MSTRG.18046.3 : Pan2 : R9PXX6	2.14	yes	up	4.2E-04	yes	chr7	exon	internal exon	NA	1.17	no	up	0.56	no	0.84	no	down	1.2E-03	yes
ENSRNOT00000077020 : Synm : G3V9G5	1.68	yes	up	3.0E-04	yes	chr1	exon	last exon	NA	0.99	no	down	0.50	no	0.82	yes	down	8.8E-13	yes
MSTRG.18723.2 : Deptor : F1M8Y4	2.15	yes	up	9.9E-15	yes	chr7	intron	internal intron	NA	0.69	no	down	0.58	no	0.97	no	down	2.0E-01	no
MSTRG.12668.4 : Nup188 : F1LRC6	0.22	yes	down	2.3E-05	yes	chr3	exon	internal exon	NA	0.68	no	down	0.25	no	1.02	no	up	1.9E-01	no
MSTRG.8278.26 : Rbpms : F2Z3S5	0.02	yes	down	2.6E-05	yes	chr16	promoter	Proximal	ICP	1.12	no	up	0.41	no	1.06	no	up	4.9E-05	yes
MSTRG.16640.7 : Arhgef10l : M0R7W2	2.87	yes	up	9.9E-07	yes	chr5	intron	internal intron	NA	0.50	yes	down	0.51	no	1.13	no	up	8.4E-04	yes
ENSRNOT00000055875 : Anapc1 : F1M801	0.25	yes	down	6.5E-11	yes	chr3	intron	internal intron	NA	2.17	yes	up	0.36	no	1.00	no	down	5.4E-01	no
MSTRG.20743.1 : Dst : D3ZC56	0.35	yes	down	1.3E-05	yes	chr9	intron	internal intron	NA	1.01	no	up	0.91	no	1.03	no	up	7.3E-04	yes
MSTRG.14342.2 : ST7 : A0A0G2KBB5	4.88	yes	up	1.5E-16	yes	chr4	promoter	Distal	LCP	0.90	no	down	0.52	no	1.14	no	up	1.4E-03	yes
MSTRG.20927.3 : Aox1 : F1LRQ1	0.36	yes	down	1.5E-07	yes	chr9	intron	internal intron	NA	0.76	no	down	0.15	no	0.73	yes	down	3.5E-17	yes
ENSRNOT00000093546 : Mycbp2 : D4A2D3	0.31	yes	down	9.2E-06	yes	chr15	intron	internal intron	NA	1.00	no	down	0.48	no	1.00	no	up	6.8E-01	no
MSTRG.21039.3 : Tns1 : F1LN42	0.09	yes	down	2.0E-04	yes	chr9	intron	last intron	NA	2.78	yes	up	0.33	no	0.86	no	down	7.8E-19	yes
MSTRG.18368.1 : Nt5dc3 : D3ZAI6	1.55	yes	up	4.2E-07	yes	chr7	intron	internal intron	NA	0.61	no	down	0.49	no	1.05	no	up	2.5E-05	yes
MSTRG.6191.4 : Ddr2 : B1WC09	0.49	yes	down	3.3E-05	yes	chr13	exon	internal exon	NA	1.63	no	up	0.60	no	0.95	no	down	3.2E-01	no
ENSRNOT00000093263 : Atp6v1a : D4A133	0.36	yes	down	7.3E-06	yes	chr11	intron	internal intron	NA	1.06	no	up	0.48	no	1.08	no	up	1.1E-08	yes
MSTRG.10847.3 : Gnb4 : O35353	2.44	yes	up	1.4E-05	yes	chr2	intron	last intron	NA	1.01	no	up	0.97	no	0.91	no	down	2.4E-06	yes
MSTRG.20352.15 : Ubp1 : D4A030	0.44	yes	down	2.3E-04	yes	chr8	intron	internal intron	NA	0.87	no	down	0.40	no	0.98	no	down	1.9E-01	no
MSTRG.7964.1 : Bmpr1a : Q78EA7	0.29	yes	down	2.1E-04	yes	chr16	intron	internal intron	NA	1.12	no	up	0.67	no	0.90	no	down	9.8E-10	yes
ENSRNOT00000012829 : Tfe3 : D3ZAW6	0.07	yes	down	2.9E-11	yes	chrX	exon	last exon	NA	0.93	no	down	0.71	no	1.04	no	up	1.6E-03	yes
ENSRNOT00000044158 : Casc4 : D3ZW58	0.14	yes	down	1.2E-10	yes	chr3	intron	first intron	NA	0.59	no	down	0.10	no	1.03	no	up	1.3E-02	yes
MSTRG.6480.4 : Evi5 : D3ZJN9	4.04	yes	up	4.0E-04	yes	chr14	intron	internal intron	NA	2.31	yes	up	0.12	no	0.98	no	down	2.5E-01	no
ENSRNOT00000028592 : Men1 : Q9WVR8	4.89	yes	up	3.4E-06	yes	chr1	exon	last exon	NA	0.62	no	down	0.11	no	1.06	no	up	2.0E-04	yes
ENSRNOT00000091264 : Snx17 : Q6AYS6	0.27	yes	down	1.2E-11	yes	chr6	intron	internal intron	NA	0.87	no	down	0.26	no	0.99	no	down	1.5E-01	no
MSTRG.5025.2 : Pds5b : D3ZU56	0.26	yes	down	2.1E-04	yes	chr12	promoter	Intermediate	LCP	1.27	no	up	0.09	no	0.96	no	down	4.7E-06	yes
MSTRG.18206.5 : Cirbp : P60825	0.73	yes	down	1.3E-04	yes	chr7	exon	first exon	NA	1.51	no	up	0.11	no	0.85	no	down	3.4E-16	yes
ENSRNOT00000060868 : Bmp2k : Q3SYQ0	11.81	yes	up	5.6E-07	yes	chr14	intron	internal intron	NA	0.91	no	down	0.28	no	1.19	no	up	6.4E-09	yes
MSTRG.7470.17 : Acin1 : E9PST5	0.30	yes	down	1.1E-04	yes	chr15	promoter	Distal	LCP	0.69	no	down	0.18	no	0.98	no	down	5.1E-04	yes
MSTRG.9465.2 : Dmxl1 : D4AA13	0.19	yes	down	1.3E-04	yes	chr18	intron	internal intron	NA	0.85	no	down	0.76	no	0.95	no	down	3.8E-03	yes
MSTRG.611.3 : Apoe : P02650	0.23	yes	down	3.2E-04	yes	chr1	exon	last exon	NA	0.43	yes	down	0.48	no	0.86	no	down	6.2E-12	yes
MSTRG.6248.1 : Ncstn : Q8CGU6	0.30	yes	down	6.8E-05	yes	chr13	intron	first intron	NA	2.28	yes	up	0.10	no	1.02	no	up	4.0E-02	yes
MSTRG.8278.17 : Rbpms : F2Z3S5	0.02	yes	down	2.6E-05	yes	chr16	promoter	Proximal	ICP	0.87	no	down	0.42	no	1.06	no	up	4.9E-05	yes
MSTRG.19956.1 : Myo1e : Q63356	3.15	yes	up	9.4E-05	yes	chr8	intron	first intron	NA	1.29	no	up	0.13	no	1.03	no	up	1.7E-02	yes
MSTRG.19895.3 : Hacd3 : D4ABI7	0.11	yes	down	1.7E-06	yes	chr8	intron	internal intron	NA	0.62	no	down	0.50	no	1.02	no	up	6.2E-05	yes
MSTRG.7186.2 : Sptbn1 : A0A0G2K8W9	5.72	yes	up	4.0E-04	yes	chr14	intron	internal intron	NA	0.40	yes	down	0.34	no	0.87	no	down	1.1E-18	yes
ENSRNOT00000092102 : Slc4a1 : Q5U329	0.42	yes	down	3.2E-08	yes	chr10	intron	internal intron	NA	0.89	no	down	0.11	no	0.87	no	down	1.3E-09	yes
ENSRNOT00000089569 : Ttc29 : Q6AYP3	0.28	yes	down	7.9E-05	yes	chr19	exon	internal exon	NA	0.95	no	down	0.22	no	1.55	yes	up	1.9E-10	yes
ENSRNOT00000080082 : Dnajb5 : D3ZB76	0.05	yes	down	2.8E-05	yes	chr5	promoter	Distal	ICP	0.44	yes	down	0.06	no	1.02	no	up	2.3E-01	no
MSTRG.5686.1 : Ube3b : A0A0G2K0E7	0.36	yes	down	3.1E-06	yes	chr12	intron	internal intron	NA	1.32	no	up	0.53	no	1.15	no	up	1.8E-12	yes
MSTRG.19173.8 : Ano6 : A0A0G2K1M7	3.87	yes	up	9.8E-09	yes	chr7	intron	internal intron	NA	0.90	no	down	0.74	no	0.91	no	down	2.1E-09	yes
MSTRG.2211.1 : Stx5 : Q08851	0	yes	down	9.9E-06	yes	chr1	exon	first exon	NA	1.29	no	up	0.44	no	1.01	no	up	5.9E-01	no
MSTRG.4866.4 : Vps8 : D4A5F7	7.39	yes	up	3.4E-05	yes	chr11	intron	internal intron	NA	1.01	no	up	0.89	no	0.95	no	down	3.2E-03	yes
MSTRG.3572.5 : RGD1307929 : F1LSX1	7.34	yes	up	1.1E-12	yes	chr10	intron	internal intron	NA	0.79	no	down	0.43	no	0.97	no	down	1.8E-04	yes
MSTRG.110.6 : Lama2 : F1M614	0.44	yes	down	4.4E-04	yes	chr1	intron	internal intron	NA	0.89	no	down	0.49	no	1.06	no	up	1.3E-07	yes
ENSRNOT00000019180 : Map4k4 : A0A0G2K7W4	0.36	yes	down	7.1E-05	yes	chr9	intron	internal intron	NA	1.00	no	down	0.63	no	1.10	no	up	7.1E-06	yes
ENSRNOT00000058560 : Uap1 : D3ZF39	0.41	yes	down	2.7E-06	yes	chr13	intron	internal intron	NA	1.51	no	up	0.20	no	1.13	no	up	4.6E-12	yes
MSTRG.13766.1 : Ncoa6 : G3V8C9	7.63	yes	up	1.0E-10	yes	chr3	exon	internal exon	NA	3.78	yes	up	0.15	no	1.03	no	up	4.4E-03	yes
ENSRNOT00000013496 : Ldlr : G3V7A5	0.13	yes	down	6.1E-05	yes	chr8	exon	internal exon	NA	2.15	yes	up	0.12	no	1.13	no	up	9.7E-05	yes
MSTRG.5280.9 : Taf6 : Q498R0	0.74	yes	down	2.2E-06	yes	chr12	intron	internal intron	NA	1.03	no	up	0.96	no	0.91	no	down	1.8E-03	yes
MSTRG.2881.5 : Pkd1 : Q9ERV0	inf	yes	up	6.6E-05	yes	chr10	intron	internal intron	NA	1.43	no	up	0.02	yes	1.19	no	up	9.8E-07	yes
MSTRG.10325.2 : Cast : F1LPH1	0.36	yes	down	1.6E-06	yes	chr2	intron	internal intron	NA	0.90	no	down	0.73	no	0.95	no	down	2.4E-10	yes
ENSRNOT00000093270 : Nup214 : M0RBV9	0.13	yes	down	4.7E-04	yes	chr3	intron	internal intron	NA	0.99	no	down	0.21	no	1.01	no	up	5.4E-01	no
MSTRG.7197.2 : Kcnma1 : A0A0G2K104	4.06	yes	up	2.6E-13	yes	chr15	intron	internal intron	NA	1.30	no	up	0.24	no	0.89	no	down	7.4E-06	yes
MSTRG.12137.6 : Pknox1 : Q5BJP1	0.26	yes	down	3.4E-07	yes	chr20	promoter	Proximal	LCP	0.61	no	down	0.37	no	0.89	no	down	7.5E-04	yes
MSTRG.20920.2 : Maip1 : Q6AY04	27.81	yes	up	1.4E-21	yes	chr9	promoter	Intermediate	ICP	2.11	yes	up	0.20	no	0.87	no	down	5.5E-04	yes
MSTRG.2579.4 : Sfxn3 : Q6P6T0	0.39	yes	down	7.8E-05	yes	chr1	intron	internal intron	NA	0.59	no	down	0.29	no	1.12	no	up	6.4E-07	yes
MSTRG.12896.5 : Rabgap1 : D3ZX42	3.82	yes	up	8.1E-06	yes	chr3	intron	internal intron	NA	0.99	no	down	0.98	no	1.01	no	up	1.9E-01	no
ENSRNOT00000004716 : Spta1 : D4A678	10.78	yes	up	3.5E-09	yes	chr13	intron	internal intron	NA	1.00	no	down	0.58	no	0.93	no	down	7.8E-08	yes
MSTRG.20698.5 : Tram2 : F1LUA2	3	yes	up	2.1E-05	yes	chr9	intron	first intron	NA	0.71	no	down	0.47	no	1.02	no	up	1.2E-01	no
MSTRG.20157.1 : Dzip1l : Q5XIA0	0.67	yes	down	4.1E-05	yes	chr8	intron	internal intron	NA	1.87	no	up	0.42	no	1.09	no	up	1.8E-03	yes
ENSRNOT00000023853 : Ctcf : Q9R1D1	2.29	yes	up	1.4E-04	yes	chr19	intron	internal intron	NA	0.57	no	down	0.11	no	0.94	no	down	4.5E-07	yes
MSTRG.2596.2 : Gbf1 : A0A0G2K3N1	0.28	yes	down	1.1E-05	yes	chr1	intron	internal intron	NA	0.99	no	down	0.88	no	1.05	no	up	6.1E-10	yes
ENSRNOT00000011864 : Phip : F1M3B3	4.05	yes	up	4.4E-04	yes	chr8	intron	internal intron	NA	1.02	no	up	0.96	no	0.95	no	down	6.3E-02	no
MSTRG.1326.2 : Picalm : A0A1B0GWW9	8.74	yes	up	2.1E-13	yes	chr1	intron	internal intron	NA	1.00	no	down	0.99	no	1.04	no	up	1.0E-01	no
ENSRNOT00000092805 : Aox1 : F1LRQ1	0.36	yes	down	1.5E-07	yes	chr9	intron	internal intron	NA	0.98	no	down	0.50	no	0.73	yes	down	3.5E-17	yes
ENSRNOT00000019874 : Pi4k2a : Q99M64	0.48	yes	down	2.9E-06	yes	chr1	intron	internal intron	NA	1.06	no	up	0.83	no	1.02	no	up	1.6E-01	no
MSTRG.5613.2 : Tbx3 : A0A0G2K8D7	2.83	yes	up	2.0E-10	yes	chr12	intron	internal intron	NA	1.55	no	up	0.34	no	0.99	no	down	7.3E-01	no
ENSRNOT00000092689 : Washc2c : F1LPG9	0.05	yes	down	2.7E-19	yes	chr4	exon	internal exon	NA	1.17	no	up	0.38	no	1.05	no	up	2.1E-05	yes
MSTRG.8278.1 : Rbpms : F2Z3S5	0.02	yes	down	2.6E-05	yes	chr16	promoter	Proximal	ICP	2.79	yes	up	0.25	no	1.06	no	up	4.9E-05	yes
ENSRNOT00000083659 : Nfrkb : D4A421	0.58	yes	down	1.3E-05	yes	chr8	intron	internal intron	NA	1.00	no	down	0.50	no	0.89	no	down	2.5E-03	yes
ENSRNOT00000082271 : Plec : Q6S3A0	0.33	yes	down	4.2E-06	yes	chr7	exon	last exon	NA	1.04	no	up	0.53	no	1.11	no	up	6.0E-08	yes
MSTRG.18046.1 : Pan2 : R9PXX6	2.14	yes	up	4.2E-04	yes	chr7	exon	internal exon	NA	0.90	no	down	0.63	no	0.84	no	down	1.2E-03	yes

MSTRG.11985.3 : Vars : Q04462	4.99	yes	up	5.5E-05	yes	chr20	intron	internal intron	NA	1.71	no	up	0.09	no	1.01	no	up	2.1E-04	yes
ENSRNOT00000083955 : Fkbp11 : G3V7V5	inf	yes	up	4.7E-06	yes	chr7	intron	internal intron	NA	0.93	no	down	0.83	no	1.06	no	up	1.6E-07	yes
MSTRG.1427.1 : Arhgef17 : A0A0G2JXT9	0.45	yes	down	6.0E-05	yes	chr1	exon	last exon	NA	1.32	no	up	0.41	no	0.93	no	down	3.1E-10	yes
MSTRG.6733.1 : Limch1 : F1M392	1.09	no	up	3.3E-04	yes	chr14	intron	last intron	NA	0.81	no	down	0.48	no	0.85	no	down	1.1E-13	yes
MSTRG.4038.2 : Plcd3 : D4A978	inf	yes	up	1.4E-05	yes	chr10	promoter	Distal	LCP	0.48	yes	down	0.40	no	0.86	no	down	2.9E-08	yes
ENSRNOT00000092021 : Myo1g : A0A0G2K6E3	0.66	yes	down	3.2E-12	yes	chr14	intron	internal intron	NA	1.50	no	up	0.28	no	1.04	no	up	3.4E-02	yes
ENSRNOT00000048418 : Ubtf : P25977	1.48	yes	up	1.5E-06	yes	chr10	intron	internal intron	NA	1.62	no	up	0.20	no	0.96	no	down	1.4E-06	yes
MSTRG.13558.1 : Siglec1 : A0A0G2K320	0.24	yes	down	6.2E-06	yes	chr3	promoter	Proximal	LCP	1.85	no	up	0.01	yes	1.25	yes	up	7.1E-13	yes
MSTRG.5467.1 : Stx2 : Q7TS57	4.45	yes	up	6.0E-04	yes	chr12	exon	last exon	NA	1.36	no	up	0.03	yes	0.91	no	down	2.6E-04	yes
MSTRG.18481.1 : Zdhhc17 : E9PTT0	5.08	yes	up	4.0E-04	yes	chr7	intron	internal intron	NA	0.48	yes	down	0.09	no	0.94	no	down	1.6E-01	no
MSTRG.7561.1 : Phf11b : M0RB46	3.03	yes	up	2.9E-04	yes	chr15	promoter	Intermediate	LCP	0.99	no	down	0.97	no	1.15	no	up	2.3E-06	yes
ENSRNOT00000093318 : Cln5 : A0A1W2Q672	0.33	yes	down	1.1E-04	yes	chr15	intron	first intron	NA	1.01	no	up	0.95	no	1.04	no	up	3.7E-01	no
MSTRG.8717.2 : Mcur1 : D3ZEJ2	0.10	yes	down	2.2E-05	yes	chr17	promoter	Distal	LCP	1.42	no	up	0.14	no	0.92	no	down	1.0E-11	yes
ENSRNOT00000030825 : Ncbp1 : Q56A27	0.31	yes	down	1.1E-07	yes	chr5	intron	internal intron	NA	1.09	no	up	0.50	no	1.00	no	up	9.4E-01	no
MSTRG.15089.5 : Csgalnact2 : D4A5Z0	2.74	yes	up	6.9E-07	yes	chr4	intron	internal intron	NA	1.01	no	up	0.94	no	0.94	no	down	1.2E-02	yes
MSTRG.2579.2 : Sfxn3 : Q9JHY2	0.39	yes	down	7.8E-05	yes	chr1	intron	internal intron	NA	0.37	yes	down	0.20	no	0.98	no	down	7.5E-03	yes
ENSRNOT00000039836 : Psmd3 : Q5U2S7	0.29	yes	down	4.3E-04	yes	chr10	intron	internal intron	NA	0.98	no	down	0.33	no	0.96	no	down	1.8E-06	yes
MSTRG.21273.1 : Fer : A0A140TAC4	3.31	yes	up	2.7E-04	yes	chr9	promoter	Proximal	LCP	0.72	no	down	0.04	yes	0.92	no	down	7.3E-07	yes
ENSRNOT00000076602 : Aco2 : Q9ER34	0	yes	down	2.0E-04	yes	chr7	promoter	Distal	ICP	1.07	no	up	0.35	no	0.90	no	down	5.3E-13	yes
MSTRG.5482.13 : Ncor2 : A0A0G2JU91	2.16	yes	up	1.6E-06	yes	chr12	intron	internal intron	NA	1.05	no	up	0.93	no	1.01	no	up	7.1E-01	no
MSTRG.8033.5 : Slc27a1 : Q6GMM8	0.26	yes	down	5.6E-15	yes	chr16	intron	internal intron	NA	0.65	no	down	0.03	yes	0.99	no	down	2.5E-01	no
ENSRNOT00000093052 : Picalm : Q66WT9	8.74	yes	up	2.1E-13	yes	chr1	intron	internal intron	NA	1.05	no	up	0.84	no	1.45	yes	up	1.6E-10	yes
ENSRNOT00000077125 : Sbf2 : B5DEJ9	4.77	yes	up	8.9E-06	yes	chr1	intron	internal intron	NA	1.01	no	up	0.23	no	1.03	no	up	9.5E-02	no
MSTRG.21175.2 : Inpp5d : F1M981	0.08	yes	down	9.9E-05	yes	chr9	intron	internal intron	NA	0.65	no	down	0.05	yes	1.08	no	up	3.1E-05	yes
MSTRG.9278.2 : Ercc3 : Q4G005	0.04	yes	down	1.2E-04	yes	chr18	promoter	Intermediate	ICP	1.45	no	up	0.32	no	0.97	no	down	5.7E-03	yes
ENSRNOT00000082723 : Itpr1 : A0A0A0MY31	3.16	yes	up	1.1E-05	yes	chr4	intron	internal intron	NA	0.87	no	down	0.43	no	0.87	no	down	1.1E-14	yes
ENSRNOT00000017211 : Tm9sf2 : Q66HG5	0.39	yes	down	9.3E-05	yes	chr15	intron	last intron	NA	0.74	no	down	0.47	no	1.03	no	up	6.1E-04	yes
ENSRNOT00000028793 : Txnip : Q5M7W1	6.38	yes	up	4.0E-07	yes	chr2	exon	internal exon	NA	0.57	no	down	0.00	yes	0.85	no	down	1.0E-06	yes
MSTRG.7186.5 : Sptbn1 : A0A0G2K8W9	5.72	yes	up	4.0E-04	yes	chr14	intron	internal intron	NA	0.21	yes	down	0.40	no	0.87	no	down	1.1E-18	yes
MSTRG.15715.2 : Dnajb5 : D3ZB76	0.05	yes	down	2.8E-05	yes	chr5	promoter	Distal	ICP	1.12	no	up	0.76	no	1.02	no	up	2.3E-01	no
MSTRG.877.5 : Lsm14a : A0A0G2JUK2	0.31	yes	down	3.8E-04	yes	chr1	intron	last intron	NA	2.42	yes	up	0.11	no	1.02	no	up	2.8E-01	no
MSTRG.6818.2 : Rbpj : M0R7Q3	0.34	yes	down	7.0E-05	yes	chr14	intron	last intron	NA	1.93	no	up	0.32	no	1.08	no	up	1.1E-04	yes
MSTRG.12910.1 : Golga1 : D4A6K4	0.04	yes	down	1.1E-04	yes	chr3	intron	internal intron	NA	1.00	no	down	0.99	no	1.12	no	up	8.2E-05	yes
ENSRNOT00000011330 : Decr1 : G3V734	0.34	yes	down	2.6E-06	yes	chr5	exon	internal exon	NA	0.90	no	down	0.48	no	0.84	no	down	1.9E-13	yes
MSTRG.1446.1 : Folr2 : D4A4S5	0.21	yes	down	8.7E-05	yes	chr1	promoter	Distal	LCP	1.43	no	up	0.15	no	1.42	yes	up	6.4E-18	yes
MSTRG.13734.2 : Bpifb1 : A0JPN3	1.80	yes	up	3.8E-07	yes	chr3	intron	internal intron	NA	0.42	yes	down	0.32	no	0.76	yes	down	2.7E-18	yes
MSTRG.10153.7 : Cmip : A0A0G2JYB9	0.58	yes	down	9.9E-05	yes	chr19	intron	internal intron	NA	0.49	yes	down	0.26	no	0.89	no	down	2.1E-04	yes
ENSRNOT00000081110 : Adam17 : G3V711	0.27	yes	down	3.8E-13	yes	chr6	intron	internal intron	NA	1.20	no	up	0.07	no	1.00	no	down	9.2E-01	no
MSTRG.3866.1 : Srcin1 : Q9QXY2	3.06	yes	up	2.8E-04	yes	chr10	intron	last intron	NA	1.31	no	up	0.12	no	1.05	no	up	1.2E-02	yes
MSTRG.1731.4 : Tbc1d10b : D3ZSY8	1.59	yes	up	2.7E-04	yes	chr1	intron	internal intron	NA	1.33	no	up	0.65	no	0.99	no	down	3.1E-01	no
ENSRNOT00000092837 : Trim21 : D4ACF2	0.20	yes	down	5.7E-10	yes	chr1	promoter	Distal	LCP	1.27	no	up	0.11	no	0.96	no	down	1.3E-04	yes
MSTRG.7350.12 : Ktn1 : D4A4Z9	2.49	yes	up	1.2E-04	yes	chr15	intron	internal intron	NA	1.02	no	up	0.71	no	1.04	no	up	2.2E-11	yes
MSTRG.9334.1 : Tmem173 : F1M391	inf	yes	up	7.6E-06	yes	chr18	intron	last intron	NA	0.95	no	down	0.76	no	1.18	no	up	2.5E-10	yes
ENSRNOT00000061513 : Clasp1 : F1LNR1	0.36	yes	down	6.7E-07	yes	chr13	intron	internal intron	NA	0.98	no	down	0.14	no	1.00	no	down	7.0E-01	no
MSTRG.20352.17 : Ubp1 : D4A030	0.44	yes	down	2.3E-04	yes	chr8	intron	internal intron	NA	1.38	no	up	0.07	no	0.98	no	down	1.9E-01	no
ENSRNOT00000076823 : Aco2 : Q9ER34	0	yes	down	2.0E-04	yes	chr7	promoter	Distal	ICP	0.98	no	down	0.90	no	0.90	no	down	5.3E-13	yes
ENSRNOT00000077044 : Asph : A0A096MKE0	2.56	yes	up	9.8E-06	yes	chr5	intron	internal intron	NA	1.16	no	up	0.10	no	0.99	no	down	1.3E-01	no
ENSRNOT00000058685 : Sin3a : A0A0G2K3H5	0.53	yes	down	4.1E-06	yes	chr8	intron	last intron	NA	0.98	no	down	0.47	no	0.96	no	down	6.2E-05	yes
MSTRG.8278.35 : Rbpms : F2Z3S5	0.02	yes	down	2.6E-05	yes	chr16	promoter	Proximal	ICP	1.14	no	up	0.14	no	1.06	no	up	4.9E-05	yes
MSTRG.10411.3 : Thbs4 : F1LMS5	0.15	yes	down	4.0E-04	yes	chr2	intron	internal intron	NA	1.07	no	up	0.47	no	1.25	yes	up	3.7E-10	yes
MSTRG.18670.3 : Ubr5 : H9KVE3	4.26	yes	up	2.2E-04	yes	chr7	intron	first intron	NA	0.87	no	down	0.46	no	0.94	no	down	3.0E-02	yes
ENSRNOT00000003314 : Serpinb10 : G3V6B2	0.43	yes	down	3.2E-05	yes	chr13	promoter	Distal	LCP	0.84	no	down	0.17	no	1.30	yes	up	1.6E-14	yes
MSTRG.9650.1 : Hdhd2 : Q6QI86	0.20	yes	down	5.1E-06	yes	chr18	intron	first intron	NA	0.79	no	down	0.55	no	1.04	no	up	3.3E-03	yes
MSTRG.18670.1 : Ubr5 : H9KVE3	4.26	yes	up	2.2E-04	yes	chr7	intron	first intron	NA	2.54	yes	up	0.25	no	0.94	no	down	3.0E-02	yes
ENSRNOT00000020775 : Ces1f : M0R7R1	0.26	yes	down	2.8E-08	yes	chr19	intron	internal intron	NA	1.00	no	down	0.75	no	0.86	no	down	3.2E-11	yes
MSTRG.1443.13 : Arap1 : F1LM60	2.71	yes	up	3.3E-04	yes	chr1	intron	first intron	NA	1.06	no	up	0.85	no	0.99	no	down	2.5E-02	yes
MSTRG.14960.3 : Eogt : Q5NDL0	6.71	yes	up	1.7E-04	yes	chr4	intron	internal intron	NA	1.53	no	up	0.32	no	1.10	no	up	2.0E-05	yes
MSTRG.3645.10 : Slfn13 : A0A096MKD0	2.27	yes	up	1.5E-04	yes	chr10	promoter	Intermediate	LCP	1.50	no	up	0.49	no	0.97	no	down	2.7E-02	yes
ENSRNOT00000081150 : Slc35a1 : D3ZZ48	0.47	yes	down	4.9E-04	yes	chr5	exon	internal exon	NA	1.05	no	up	0.67	no	1.00	no	up	9.2E-01	no
MSTRG.5482.7 : Ncor2 : A0A0G2JU91	2.16	yes	up	1.6E-06	yes	chr12	intron	internal intron	NA	1.57	no	up	0.48	no	1.01	no	up	7.1E-01	no
MSTRG.5398.1 : Eln : Q99372	3.55	yes	up	3.0E-04	yes	chr12	intron	internal intron	NA	0.52	no	down	0.17	no	0.82	yes	down	3.9E-08	yes
ENSRNOT00000014552 : Thbs2 : D4A2G6	1.43	yes	up	1.4E-19	yes	chr1	intron	internal intron	NA	1.80	no	up	0.06	no	1.17	no	up	2.2E-05	yes
MSTRG.19895.7 : Hacd3 : D4ABI7	0.11	yes	down	1.7E-06	yes	chr8	intron	internal intron	NA	1.06	no	up	0.47	no	1.02	no	up	6.2E-05	yes
MSTRG.877.2 : Lsm14a : A0A0G2JUK2	0.31	yes	down	3.8E-04	yes	chr1	intron	last intron	NA	0.48	yes	down	0.22	no	1.02	no	up	2.8E-01	no
MSTRG.5417.5 : Auts2 : F1M388	0.04	yes	down	4.8E-14	yes	chr12	intron	internal intron	NA	1.17	no	up	0.51	no	1.01	no	up	5.6E-01	no
ENSRNOT00000027685 : Stim1 : P84903	2.61	yes	up	1.3E-04	yes	chr1	exon	last exon	NA	0.93	no	down	0.66	no	0.92	no	down	3.4E-08	yes
ENSRNOT00000061054 : Lims2 : A0A0G2KAE1	0.67	yes	down	6.6E-13	yes	chr18	intron	internal intron	NA	0.58	no	down	0.10	no	0.82	yes	down	4.4E-10	yes
MSTRG.6713.9 : Fryl : D3ZQY4	2.76	yes	up	1.7E-04	yes	chr14	intron	internal intron	NA	0.65	no	down	0.18	no	1.00	no	down	4.6E-01	no
ENSRNOT00000003992 : Tsr1 : D3ZEM8	45.59	yes	up	3.4E-04	yes	chr10	intron	internal intron	NA	1.35	no	up	0.20	no	1.00	no	down	9.7E-01	no
ENSRNOT00000037115 : Golim4 : Q5BJK8	0.34	yes	down	3.2E-04	yes	chr2	intron	internal intron	NA	0.88	no	down	0.60	no	1.06	no	up	1.6E-03	yes
MSTRG.3781.1 : Luc7l3 : D3ZFB2	0.31	yes	down	4.3E-04	yes	chr10	intron	internal intron	NA	1.07	no	up	0.48	no	0.98	no	down	9.6E-04	yes
MSTRG.5906.4 : Atp2b4 : Q64542	2.31	yes	up	1.3E-05	yes	chr13	intron	internal intron	NA	0.74	no	down	0.04	yes	0.68	yes	down	2.6E-19	yes
MSTRG.8278.16 : Rbpms : F2Z3S5	0.02	yes	down	2.6E-05	yes	chr16	promoter	Proximal	ICP	1.15	no	up	0.66	no	1.06	no	up	4.9E-05	yes
ENSRNOT00000006682 : Agr2 : D3ZIA7	0.34	yes	down	2.0E-08	yes	chr6	intron	first intron	NA	0.95	no	down	0.79	no	0.85	no	down	2.5E-12	yes
MSTRG.3538.3 : Abr : A0A0G2JZZ7	inf	yes	up	3.7E-04	yes	chr10	intron	internal intron	NA	0.77	no	down	0.50	no	1.05	no	up	1.0E-03	yes
MSTRG.6211.7 : Fcgr2b : A3RLA8	0.44	yes	down	2.8E-04	yes	chr13	promoter	Distal	LCP	1.14	no	up	0.88	no	1.04	no	up	4.3E-03	yes
ENSRNOT00000004942 : Tmed7 : D3ZTX0	4.37	yes	up	3.1E-04	yes	chr18	promoter	Distal	LCP	1.32	no	up	0.05	yes	0.98	no	down	4.8E-02	yes
MSTRG.22091.2 : Arhgef6 : Q5XXR3	0.21	yes	down	2.1E-04	yes	chrX	intron	internal intron	NA	1.42	no	up	0.09	no	0.93	no	down	2.3E-08	yes
MSTRG.2665.2 : Acsl5 : O88813	6.59	yes	up	1.3E-04	yes	chr1	intron	last intron	NA	1.22	no	up	0.27	no	1.08	no	up	2.2E-08	yes
ENSRNOT00000028143 : Pten : O54857	0.23	yes	down	5.0E-05	yes	chr1	intron	internal intron	NA	1.15	no	up	0.32	no	0.94	no	down	2.3E-06	yes
MSTRG.20284.4 : Qrich1 : F1M4M7	3.68	yes	up	3.4E-07	yes	chr8	exon	internal exon	NA	1.29	no	up	0.15	no	1.03	no	up	2.0E-01	no
MSTRG.13559.2 : Hspa12b : D3ZVM5	0.65	yes	down	3.5E-04	yes	chr3	intron	last intron	NA	0.91	no	down	0.79	no	0.86	no	down	5.9E-13	yes
MSTRG.5613.1 : Tbx3 : A0A0G2K8D7	2.83	yes	up	2.0E-10	yes	chr12	intron	internal intron	NA	0.91	no	down	0.78	no	0.99	no	down	7.3E-01	no
ENSRNOT00000066509 : Spta1 : D4A678	10.78	yes	up	3.5E-09	yes	chr13	intron	internal intron	NA	0.98	no	down	0.29	no	0.93	no	down	7.8E-08	yes
ENSRNOT00000079688 : Epb41l2 : D3ZM69	0.25	yes	down	1.1E-12	yes	chr1	intron	internal intron	NA	0.98	no	down	0.34	no	0.91	no	down	8.9E-11	yes
ENSRNOT00000083933 : Mapk8 : A0A0G2KA63	0.22	yes	down	1.8E-04	yes	chr16	intron	internal intron	NA	1.11	no	up	0.66	no	1.06	no	up	2.4E-05	yes
MSTRG.12337.3 : Sgpl1 : Q8CHN6	0.20	yes	down	6.8E-07	yes	chr20	intron	internal intron	NA	1.01	no	up	0.98	no	1.12	no	up	5.6E-16	yes
MSTRG.479.5 : Epn1 : O88339	2.09	yes	up	1.9E-04	yes	chr1	intron	internal intron	NA	0.91	no	down	0.68	no	0.99	no	down	3.7E-01	no
ENSRNOT00000057734 : Nebl : F1LVX3	0	yes	down	3.8E-04	yes	chr17	intron	first intron	NA	0.98	no	down	0.95	no	1.02	no	up	2.0E-01	no
ENSRNOT00000075983 : Nebl : F1LVX3	0	yes	down	3.8E-04	yes	chr17	intron	first intron	NA	0.92	no	down	0.13	no	1.02	no	up	2.0E-01	no
MSTRG.7470.10 : Acin1 : E9PST5	0.30	yes	down	1.1E-04	yes	chr15	promoter	Distal	LCP	1.08	no	up	0.52	no	0.98	no	down	5.1E-04	yes
MSTRG.20752.4 : Ptpn18 : Q4KM54	0	yes	down	1.9E-04	yes	chr9	intron	first intron	NA	0.91	no	down	0.76	no	1.20	no	up	1.2E-08	yes
MSTRG.7503.1 : Gmpr2 : A0A0G2JX25	0.33	yes	down	2.2E-06	yes	chr15	promoter	Distal	LCP	1.18	no	up	0.44	no	0.95	no	down	1.0E-01	no
ENSRNOT00000037848 : Magi1 : M0R8T1	0.38	yes	down	2.0E-04	yes	chr4	intron	internal intron	NA	1.08	no	up	0.09	no	0.99	no	down	6.0E-01	no
ENSRNOT00000032299 : Tmem245 : D3ZXD8	5.92	yes	up	3.2E-05	yes	chr5	intron	internal intron	NA	0.91	no	down	0.45	no	0.88	no	down	4.0E-07	yes
MSTRG.3895.2 : Cdk12 : A0A0G2K5U7	5.94	yes	up	1.1E-04	yes	chr10	exon	first exon	NA	1.03	no	up	0.86	no	0.97	no	down	5.5E-04	yes
ENSRNOT00000070909 : Tatdn1 : M0R6T1	0.49	yes	down	2.8E-04	yes	chr7	promoter	Distal	LCP	0.87	no	down	0.49	no	0.91	no	down	4.5E-09	yes
MSTRG.2431.2 : Pten : O54857	0.23	yes	down	5.0E-05	yes	chr1	intron	internal intron	NA	1.04	no	up	0.94	no	0.94	no	down	2.3E-06	yes
MSTRG.16339.2 : Fhl3 : D3ZPF0	0.20	yes	down	2.5E-09	yes	chr5	exon	last exon	NA	0.73	no	down	0.19	no	1.28	yes	up	1.8E-14	yes
MSTRG.1201.5 : Ntrk3 : Q68G04	7.03	yes	up	1.5E-06	yes	chr1	intron	internal intron	NA	2.27	yes	up	0.49	no	0.87	no	down	1.8E-07	yes

MSTRG.6635.5 : Rufy3 : A0A0G2K6A9	2.37	yes	up	3.9E-04	yes	chr14	promoter	Distal	LCP	1.34	no	up	0.20	no	1.11	no	up	1.3E-05	yes
ENSRNOT00000078820 : Gas7 : M0R4R4	4.60	yes	up	4.0E-11	yes	chr10	intron	first intron	NA	0.49	yes	down	0.22	no	1.22	yes	up	2.5E-15	yes
ENSRNOT00000092029 : Hdac7 : A0A0G2K6B1	1.57	yes	up	6.8E-05	yes	chr7	intron	internal intron	NA	1.11	no	up	0.70	no	0.99	no	down	6.7E-01	no
ENSRNOT00000077878 : Mon2 : D3ZCG3	0.07	yes	down	1.5E-08	yes	chr7	intron	internal intron	NA	1.01	no	up	0.47	no	1.05	no	up	7.1E-07	yes
MSTRG.659.3 : Cic : D4A853	4.32	yes	up	3.8E-07	yes	chr1	exon	internal exon	NA	1.06	no	up	0.83	no	1.06	no	up	4.3E-02	yes
MSTRG.10411.2 : Thbs4 : F1LMS5	0.15	yes	down	4.0E-04	yes	chr2	intron	internal intron	NA	0.99	no	down	0.45	no	1.25	yes	up	3.7E-10	yes
ENSRNOT00000064932 : Dnmt1 : D3ZS06	0.16	yes	down	6.3E-05	yes	chr8	intron	internal intron	NA	1.11	no	up	0.45	no	1.00	no	down	4.1E-01	no
ENSRNOT00000049967 : Atr : D3Z822	0.06	yes	down	2.6E-04	yes	chr8	intron	internal intron	NA	1.11	no	up	0.39	no	1.05	no	up	1.7E-08	yes
MSTRG.22064.7 : Arhgap4 : A0A0G2JVF0	0.34	yes	down	1.4E-05	yes	chrX	intron	internal intron	NA	0.93	no	down	0.88	no	1.07	no	up	7.1E-04	yes
ENSRNOT00000075989 : Spp1 : P08721	29.54	yes	up	1.8E-44	yes	chr14	exon	last exon	NA	5.34	yes	up	0.17	no	1.32	yes	up	5.2E-14	yes
MSTRG.5025.6 : Pds5b : D3ZU56	0.26	yes	down	2.1E-04	yes	chr12	promoter	Intermediate	LCP	0.73	no	down	0.31	no	0.96	no	down	4.7E-06	yes
MSTRG.16606.3 : Alpl : P08289	0.52	yes	down	2.4E-04	yes	chr5	promoter	Intermediate	LCP	5.15	yes	up	0.02	yes	1.00	no	down	6.2E-01	no
MSTRG.18043.4 : Stat2 : Q5XI26	0.06	yes	down	1.0E-04	yes	chr7	promoter	Distal	LCP	0.78	no	down	0.53	no	1.14	no	up	1.9E-09	yes
MSTRG.16523.1 : Arid1a : D4A3E3	0.22	yes	down	1.1E-04	yes	chr5	intron	first intron	NA	1.83	no	up	0.50	no	1.00	no	down	2.1E-01	no
ENSRNOT00000000142 : Dnajb5 : D3ZB76	0.05	yes	down	2.8E-05	yes	chr5	promoter	Distal	ICP	1.01	no	up	0.24	no	1.02	no	up	2.3E-01	no
MSTRG.11091.3 : Gucy1a3 : P19686	0.26	yes	down	3.9E-06	yes	chr2	intron	internal intron	NA	0.63	no	down	0.50	no	0.85	no	down	1.4E-12	yes
MSTRG.8815.1 : Dcdc2 : D3ZR10	5.48	yes	up	1.6E-11	yes	chr17	exon	last exon	NA	1.03	no	up	0.92	no	0.87	no	down	5.6E-02	no
MSTRG.16317.3 : Cap1 : Q08163	0.21	yes	down	1.6E-07	yes	chr5	intron	internal intron	NA	2.19	yes	up	0.01	yes	0.94	no	down	1.0E-08	yes
MSTRG.4725.3 : Kalrn : P97924	1.54	yes	up	9.1E-07	yes	chr11	exon	last exon	NA	1.36	no	up	0.15	no	1.04	no	up	1.6E-04	yes
MSTRG.5467.3 : Stx2 : Q7TS57	4.45	yes	up	6.0E-04	yes	chr12	exon	last exon	NA	1.00	no	down	0.99	no	0.91	no	down	2.6E-04	yes
ENSRNOT00000076676 : Parp4 : A0A096MK99	1.23	yes	up	1.1E-06	yes	chr15	intron	last intron	NA	1.05	no	up	0.37	no	0.94	no	down	4.1E-06	yes
MSTRG.5398.1 : Eln : A0A0G2JST5	3.55	yes	up	3.0E-04	yes	chr12	intron	internal intron	NA	0.52	no	down	0.17	no	0.91	no	down	1.2E-01	no
MSTRG.18268.17 : Akap8 : Q63014	0.08	yes	down	2.7E-04	yes	chr7	intron	internal intron	NA	0.93	no	down	0.09	no	0.99	no	down	2.8E-01	no
MSTRG.6855.7 : Prom1 : Q9JI49	8.32	yes	up	2.4E-09	yes	chr14	intron	internal intron	NA	0.62	no	down	0.11	no	0.87	no	down	1.5E-05	yes
ENSRNOT00000091470 : Picalm : Q66SY1	8.74	yes	up	2.1E-13	yes	chr1	intron	internal intron	NA	1.31	no	up	0.64	no	1.13	no	up	1.1E-06	yes
MSTRG.1443.14 : Arap1 : F1LM60	2.71	yes	up	3.3E-04	yes	chr1	intron	first intron	NA	0.59	no	down	0.34	no	0.99	no	down	2.5E-02	yes
MSTRG.3895.1 : Cdk12 : A0A0G2K5U7	5.94	yes	up	1.1E-04	yes	chr10	exon	first exon	NA	1.30	no	up	0.27	no	0.97	no	down	5.5E-04	yes
MSTRG.18268.10 : Akap8 : Q63014	0.08	yes	down	2.7E-04	yes	chr7	intron	internal intron	NA	1.38	no	up	0.35	no	0.99	no	down	2.8E-01	no
MSTRG.15366.1 : Cmas : Q5M963	5.02	yes	up	1.4E-04	yes	chr4	intron	internal intron	NA	0.97	no	down	0.31	no	0.86	no	down	3.1E-13	yes
MSTRG.19945.1 : Tpm1 : Q923Z2	9.41	yes	up	5.1E-14	yes	chr8	intron	internal intron	NA	0.98	no	down	0.92	no	0.75	yes	down	6.0E-18	yes
MSTRG.18812.1 : Trappc9 : B1H266	4.58	yes	up	1.4E-09	yes	chr7	intron	internal intron	NA	0.36	yes	down	0.14	no	0.94	no	down	3.8E-02	yes
MSTRG.19220.12 : Kmt2d : A0A0G2JVD6	6.53	yes	up	2.1E-09	yes	chr7	exon	last exon	NA	1.62	no	up	0.14	no	1.03	no	up	3.5E-02	yes
ENSRNOT00000089560 : Tle1 : A0A0G2K324	0.11	yes	down	4.0E-05	yes	chr5	exon	last exon	NA	0.85	no	down	0.51	no	0.95	no	down	1.3E-05	yes
MSTRG.15479.3 : Prex2 : A0A0G2KA11	5.57	yes	up	1.6E-04	yes	chr5	intron	internal intron	NA	0.64	no	down	0.31	no	1.02	no	up	2.3E-01	no
ENSRNOT00000090032 : Naa35 : Q6DKG0	inf	yes	up	1.7E-04	yes	chr17	promoter	Proximal	LCP	3.38	yes	up	0.03	yes	1.04	no	up	3.3E-02	yes
ENSRNOT00000051338 : Inpp5d : F1M981	0.08	yes	down	9.9E-05	yes	chr9	intron	internal intron	NA	1.53	no	up	0.10	no	1.08	no	up	3.1E-05	yes
ENSRNOT00000068553 : Nebl : F1LVX3	0	yes	down	3.8E-04	yes	chr17	intron	first intron	NA	1.16	no	up	0.10	no	1.02	no	up	2.0E-01	no
ENSRNOT00000091357 : Cic : D4A853	4.32	yes	up	3.8E-07	yes	chr1	exon	internal exon	NA	1.06	no	up	0.73	no	1.06	no	up	4.3E-02	yes
MSTRG.10830.4 : Phc3 : D3ZS50	0.36	yes	down	4.0E-04	yes	chr2	intron	internal intron	NA	0.58	no	down	0.61	no	0.86	no	down	4.2E-04	yes
MSTRG.276.8 : Arid1b : F1LNP1	1.53	yes	up	6.5E-08	yes	chr1	promoter	Proximal	LCP	1.18	no	up	0.83	no	0.98	no	down	1.8E-01	no
MSTRG.13195.10 : Arhgap1 : D4A6C5	3.73	yes	up	1.8E-11	yes	chr3	intron	internal intron	NA	0.71	no	down	0.46	no	1.04	no	up	1.7E-04	yes
MSTRG.19657.1 : Ube4a : F1M9N5	4.51	yes	up	1.3E-04	yes	chr8	intron	internal intron	NA	0.69	no	down	0.37	no	0.97	no	down	2.4E-03	yes
MSTRG.11813.2 : Wls : Q6P689	0.38	yes	down	6.5E-05	yes	chr2	exon	last exon	NA	0.94	no	down	0.88	no	1.11	no	up	1.3E-07	yes
ENSRNOT00000065542 : Kcnma1 : A0A0G2K104	4.06	yes	up	2.6E-13	yes	chr15	intron	internal intron	NA	0.93	no	down	0.42	no	0.89	no	down	7.4E-06	yes
ENSRNOT00000093656 : Phf8 : D4AD31	0	yes	down	2.1E-07	yes	chrX	exon	first exon	NA	1.05	no	up	0.47	no	1.13	no	up	3.6E-02	yes
MSTRG.12337.4 : Sgpl1 : Q8CHN6	0.20	yes	down	6.8E-07	yes	chr20	intron	internal intron	NA	1.06	no	up	0.92	no	1.12	no	up	5.6E-16	yes
MSTRG.16317.7 : Cap1 : Q08163	0.21	yes	down	1.6E-07	yes	chr5	intron	internal intron	NA	3.81	yes	up	0.05	yes	0.94	no	down	1.0E-08	yes
ENSRNOT00000079709 : Mtch2 : A0A0G2K459	2.54	yes	up	4.3E-04	yes	chr3	intron	internal intron	NA	0.93	no	down	0.70	no	1.00	no	up	8.3E-01	no
ENSRNOT00000010689 : Plscr1 : A0A0G2K7Q1	0.33	yes	down	1.3E-05	yes	chr8	promoter	Distal	LCP	1.04	no	up	0.91	no	1.08	no	up	5.2E-06	yes
ENSRNOT00000008321 : Lamb1 : D3ZQN7	3.86	yes	up	8.9E-05	yes	chr6	intron	internal intron	NA	2.02	yes	up	0.49	no	1.31	yes	up	5.4E-17	yes
MSTRG.16007.4 : Dennd4c : F1LTD7	0.31	yes	down	2.0E-04	yes	chr5	intron	internal intron	NA	0.54	no	down	0.54	no	0.97	no	down	9.0E-02	no
ENSRNOT00000023033 : Klf6 : G3V880	14.58	yes	up	4.1E-04	yes	chr17	intron	internal intron	NA	1.05	no	up	0.64	no	0.81	yes	down	4.1E-06	yes
MSTRG.16327.10 : Macf1 : A0A0G2K9T4	0.36	yes	down	1.3E-05	yes	chr5	exon	last exon	NA	2.21	yes	up	0.09	no	0.97	no	down	9.0E-11	yes
MSTRG.8511.1 : Dapk1 : F1LNN8	0.35	yes	down	1.5E-04	yes	chr17	intron	internal intron	NA	0.93	no	down	0.89	no	0.96	no	down	1.0E-01	no
MSTRG.12174.1 : Col18a1 : F1LR02	0.22	yes	down	4.1E-11	yes	chr20	exon	internal exon	NA	2.39	yes	up	0.61	no	1.03	no	up	3.6E-07	yes
MSTRG.21202.6 : Lrrfip1 : Q66HF9	6.49	yes	up	4.9E-10	yes	chr9	exon	last exon	NA	0.81	no	down	0.14	no	1.15	no	up	2.1E-11	yes
MSTRG.21023.11 : Fn1 : F1LST1	0.11	yes	down	7.7E-11	yes	chr9	intron	internal intron	NA	8.43	yes	up	0.01	yes	1.27	yes	up	4.1E-20	yes
MSTRG.4716.1 : Adcy5 : G3V9G1	0.16	yes	down	2.3E-10	yes	chr11	intron	internal intron	NA	0.39	yes	down	0.45	no	1.00	no	down	7.6E-01	no
MSTRG.14948.7 : Magi1 : M0R8T1	0.38	yes	down	2.0E-04	yes	chr4	intron	internal intron	NA	1.24	no	up	0.13	no	0.99	no	down	6.0E-01	no
MSTRG.2561.1 : Chuk : B5DF32	24.13	yes	up	2.0E-05	yes	chr1	intron	internal intron	NA	1.14	no	up	0.67	no	0.90	no	down	8.1E-05	yes
MSTRG.10570.2 : Skiv2l2 : D4AE49	5.78	yes	up	1.8E-10	yes	chr2	intron	internal intron	NA	1.20	no	up	0.09	no	0.99	no	down	1.0E-01	no
MSTRG.256.2 : Syne1 : Q8VHJ9	0.04	yes	down	1.5E-19	yes	chr1	exon	first exon	NA	1.79	no	up	0.47	no	0.89	no	down	8.9E-14	yes
MSTRG.6713.4 : Fryl : D3ZQY4	2.76	yes	up	1.7E-04	yes	chr14	intron	internal intron	NA	0.75	no	down	0.49	no	1.00	no	down	4.6E-01	no
ENSRNOT00000016418 : Prkcb : A0A0G2K5Q0	3.82	yes	up	4.6E-04	yes	chr1	intron	internal intron	NA	1.01	no	up	0.47	no	1.10	no	up	1.7E-07	yes
ENSRNOT00000087874 : Filip1 : F1LM79	4.15	yes	up	2.1E-10	yes	chr8	exon	first exon	NA	1.00	no	down	0.45	no	1.01	no	up	3.0E-01	no
MSTRG.19963.3 : Sltm : A0A0G2K904	0.34	yes	down	6.4E-06	yes	chr8	intron	internal intron	NA	1.45	no	up	0.30	no	0.94	no	down	2.8E-02	yes
ENSRNOT00000075279 : Mut : D3ZKG1	0.31	yes	down	1.0E-06	yes	chr9	exon	last exon	NA	1.22	no	up	0.30	no	1.07	no	up	3.0E-06	yes
MSTRG.4030.13 : Eftud2 : F1LM66	4.66	yes	up	6.0E-04	yes	chr10	exon	internal exon	NA	0.91	no	down	0.48	no	1.02	no	up	4.9E-02	yes
MSTRG.479.3 : Epn1 : O88339	2.09	yes	up	1.9E-04	yes	chr1	intron	internal intron	NA	1.16	no	up	0.22	no	0.99	no	down	3.7E-01	no
ENSRNOT00000012760 : Pdk4 : G3V778	0.20	yes	down	7.9E-09	yes	chr4	promoter	Proximal	LCP	0.28	yes	down	0.05	yes	0.63	yes	down	5.5E-19	yes
MSTRG.21176.9 : Inpp5d : F1M981	0.08	yes	down	9.9E-05	yes	chr9	intron	internal intron	NA	0.65	no	down	0.06	no	1.08	no	up	3.1E-05	yes
ENSRNOT00000001695 : Col6a2 : F1LNH3	7.44	yes	up	1.2E-13	yes	chr20	intron	internal intron	NA	1.00	no	down	0.98	no	0.93	no	down	1.6E-17	yes
MSTRG.6706.1 : Spata18 : Q6AYL6	1.61	yes	up	6.3E-07	yes	chr14	intron	internal intron	NA	0.45	yes	down	0.23	no	0.76	yes	down	1.3E-15	yes
MSTRG.19148.10 : Shank3 : A0A0U1RS13	3.23	yes	up	4.3E-05	yes	chr7	exon	first exon	NA	0.81	no	down	0.04	yes	0.98	no	down	1.0E-02	yes
ENSRNOT00000075886 : Becn1 : Q91XJ1	0.15	yes	down	1.2E-04	yes	chr10	exon	first exon	NA	1.13	no	up	0.26	no	0.97	no	down	9.2E-02	no
MSTRG.12973.1 : Rbms1 : A0A0G2K4R7	0	yes	down	3.3E-05	yes	chr3	intron	internal intron	NA	1.03	no	up	0.47	no	0.97	no	down	1.2E-01	no
MSTRG.4992.9 : Top3b : D4A9Z2	11.41	yes	up	1.2E-04	yes	chr11	intron	last intron	NA	0.70	no	down	0.10	no	0.99	no	down	6.1E-01	no
MSTRG.13963.3 : Ptgis : Q62969	0.55	yes	down	6.0E-05	yes	chr3	intron	internal intron	NA	3.00	yes	up	0.10	no	0.99	no	down	7.4E-02	no
MSTRG.17520.6 : Srsf5 : Q09167	0.10	yes	down	3.0E-09	yes	chr6	promoter	Proximal	ICP	2.05	yes	up	0.51	no	1.03	no	up	4.2E-02	yes
MSTRG.986.1 : Akt1s1 : D3ZH75	2.93	yes	up	2.4E-05	yes	chr1	intron	internal intron	NA	0.89	no	down	0.43	no	0.97	no	down	7.2E-01	no
MSTRG.18876.2 : Puf60 : A0A0H2UHZ6	0.25	yes	down	1.3E-05	yes	chr7	exon	internal exon	NA	1.26	no	up	0.66	no	1.00	no	down	7.4E-02	no
ENSRNOT00000037296 : Stx2 : Q7TS57	4.45	yes	up	6.0E-04	yes	chr12	exon	last exon	NA	1.00	no	down	0.68	no	0.91	no	down	2.6E-04	yes
MSTRG.7804.4 : Mycbp2 : A0A1W2Q6I3	0.31	yes	down	9.2E-06	yes	chr15	intron	internal intron	NA	1.12	no	up	0.47	no	1.00	no	up	8.8E-01	no
MSTRG.16610.1 : Ece1 : Q6IN10	0.58	yes	down	9.3E-05	yes	chr5	intron	internal intron	NA	1.00	no	down	0.50	no	1.14	no	up	8.3E-15	yes
ENSRNOT00000085332 : Cab39 : A0A0G2JZH0	39.86	yes	up	1.2E-27	yes	chr9	promoter	Intermediate	LCP	1.11	no	up	0.81	no	0.99	no	down	5.6E-01	no
MSTRG.18072.6 : Dgka : P51556	0.72	yes	down	1.5E-04	yes	chr7	exon	internal exon	NA	1.25	no	up	0.36	no	0.95	no	down	8.8E-04	yes
ENSRNOT00000090470 : Exoc1 : Q4V8H2	inf	yes	up	6.1E-05	yes	chr14	intron	first intron	NA	1.00	no	down	0.98	no	0.91	no	down	3.2E-04	yes
ENSRNOT00000024900 : Pde4dip : A0A0G2JW66	6.94	yes	up	5.0E-08	yes	chr2	exon	internal exon	NA	1.00	no	down	0.38	no	1.06	no	up	5.1E-04	yes
MSTRG.11866.1 : RT1-S3 : Q9R0V1	0.16	yes	down	4.2E-07	yes	chr20	exon	last exon	NA	1.21	no	up	0.83	no	1.05	no	up	2.0E-01	no
ENSRNOT00000073429 : Rpn1 : Q6P7A7	3.98	yes	up	2.7E-19	yes	chr4	intron	last intron	NA	1.16	no	up	0.05	yes	1.01	no	up	7.6E-02	no
ENSRNOT00000057218 : Naglt1 : A0A0G2JSX3	0.35	yes	down	3.9E-04	yes	chr20	intron	first intron	NA	0.99	no	down	0.31	no	0.95	no	down	1.5E-03	yes
MSTRG.10475.4 : Tnpo1 : F1LQP9	24.87	yes	up	1.6E-05	yes	chr2	exon	internal exon	NA	1.00	no	down	0.50	no	0.98	no	down	5.8E-02	no
MSTRG.6624.1 : Ankrd17 : D4A0B4	3.39	yes	up	8.9E-05	yes	chr14	intron	internal intron	NA	0.87	no	down	0.65	no	1.11	no	up	1.6E-06	yes
MSTRG.19329.6 : Atf7 : B0BMY0	0.38	yes	down	7.2E-06	yes	chr7	intron	internal intron	NA	0.81	no	down	0.47	no	1.00	no	down	9.7E-01	no
MSTRG.10871.6 : Dcun1d1 : D3ZRV0	1.60	yes	up	3.7E-04	yes	chr2	exon	internal exon	NA	1.03	no	up	0.92	no	0.97	no	down	2.6E-02	yes
ENSRNOT00000093052 : Picalm : A0A1B0GWW9	8.74	yes	up	2.1E-13	yes	chr1	intron	internal intron	NA	1.05	no	up	0.84	no	1.04	no	up	1.0E-01	no
MSTRG.4117.2 : Nol11 : A0A0G2K1A9	0.21	yes	down	9.6E-05	yes	chr10	intron	internal intron	NA	1.14	no	up	0.64	no	1.06	no	up	1.9E-03	yes
ENSRNOT00000007144 : Slc12a6 : A0A0G2K6T0	0.22	yes	down	4.0E-04	yes	chr3	intron	internal intron	NA	1.65	no	up	0.04	yes	1.10	no	up	9.9E-02	no
ENSRNOT00000092277 : Washc2c : F1LPG9	0.05	yes	down	2.7E-19	yes	chr4	exon	internal exon	NA	1.36	no	up	0.06	no	1.05	no	up	2.1E-05	yes
ENSRNOT00000046512 : Serpinc1 : Q5M7T5	11.92	yes	up	5.6E-26	yes	chr13	intron	first intron	NA	0.99	no	down	0.77	no	0.89	no	down	1.2E-15	yes

MSTRG.16636.5 : Ubr4 : A0A0G2JU89	2.84	yes	up	3.7E-05	yes	chr5	intron	internal intron	NA	0.55	no	down	0.50	no	1.02	no	up	4.6E-05	yes
ENSRNOT00000090299 : Dzip1l : Q5XIA0	0.67	yes	down	4.1E-05	yes	chr8	intron	internal intron	NA	0.59	no	down	0.31	no	1.09	no	up	1.8E-03	yes
MSTRG.5972.2 : Ptprc : P04157	2.98	yes	up	6.9E-14	yes	chr13	intron	internal intron	NA	1.11	no	up	0.80	no	1.12	no	up	6.1E-15	yes
ENSRNOT00000091541 : Fcgr2b : A3RLA8	0.44	yes	down	2.8E-04	yes	chr13	promoter	Distal	LCP	0.99	no	down	0.88	no	1.04	no	up	4.3E-03	yes
MSTRG.18268.22 : Akap8 : Q63014	0.08	yes	down	2.7E-04	yes	chr7	intron	internal intron	NA	1.36	no	up	0.52	no	0.99	no	down	2.8E-01	no
MSTRG.3895.5 : Cdk12 : A0A0G2K5U7	5.94	yes	up	1.1E-04	yes	chr10	exon	first exon	NA	1.21	no	up	0.21	no	0.97	no	down	5.5E-04	yes
MSTRG.15096.3 : Cacna1c : F1MA84	4.75	yes	up	4.3E-04	yes	chr4	intron	internal intron	NA	1.85	no	up	0.08	no	0.97	no	down	2.7E-02	yes
ENSRNOT00000081533 : Myef2 : A0A0G2K402	2.63	yes	up	2.7E-04	yes	chr3	promoter	Intermediate	LCP	1.18	no	up	0.41	no	0.93	no	down	4.5E-09	yes
MSTRG.1675.3 : Arhgap17 : D4AAV2	0.83	yes	down	2.6E-07	yes	chr1	intron	internal intron	NA	1.02	no	up	0.81	no	1.01	no	up	3.1E-01	no
ENSRNOT00000088042 : Fbln1 : D3ZQ25	0.23	yes	down	7.2E-09	yes	chr7	intron	internal intron	NA	0.60	no	down	0.20	no	1.06	no	up	1.7E-09	yes
MSTRG.13130.1 : Calcrl : Q63118	2.81	yes	up	1.2E-04	yes	chr3	intron	internal intron	NA	0.69	no	down	0.43	no	1.09	no	up	1.2E-04	yes
ENSRNOT00000086542 : Ankrd17 : D4A0B4	3.39	yes	up	8.9E-05	yes	chr14	intron	internal intron	NA	1.64	no	up	0.58	no	1.11	no	up	1.6E-06	yes
MSTRG.15963.1 : Ptprd : M0RB22	3.73	yes	up	1.3E-08	yes	chr5	exon	internal exon	NA	0.39	yes	down	0.11	no	1.01	no	up	5.3E-01	no
MSTRG.18670.2 : Ubr5 : H9KVE3	4.26	yes	up	2.2E-04	yes	chr7	intron	first intron	NA	0.25	yes	down	0.19	no	0.94	no	down	3.0E-02	yes
ENSRNOT00000086641 : Clasp1 : F1LNR1	0.36	yes	down	6.7E-07	yes	chr13	intron	internal intron	NA	1.00	no	down	0.65	no	1.00	no	down	7.0E-01	no
MSTRG.12277.3 : Ctnna3 : F1M4I1	0	yes	down	1.3E-06	yes	chr20	promoter	Distal	LCP	0.95	no	down	0.84	no	0.92	no	down	3.6E-04	yes
MSTRG.1907.3 : Ptdss2 : B2GV22	0.17	yes	down	6.9E-07	yes	chr1	intron	internal intron	NA	0.91	no	down	0.68	no	0.94	no	down	7.1E-04	yes
ENSRNOT00000030399 : Afg3l1 : B5DEY1	0.32	yes	down	4.6E-04	yes	chr19	exon	internal exon	NA	0.98	no	down	0.86	no	0.98	no	down	3.4E-01	no
MSTRG.11091.2 : Gucy1a3 : P19686	0.26	yes	down	3.9E-06	yes	chr2	intron	internal intron	NA	1.81	no	up	0.37	no	0.85	no	down	1.4E-12	yes
MSTRG.16821.2 : Prdm16 : M0RDL0	0.37	yes	down	2.7E-04	yes	chr5	exon	internal exon	NA	0.77	no	down	0.45	no	0.83	yes	down	1.1E-06	yes
ENSRNOT00000050555 : Atm : A0A0G2K3I0	0.65	yes	down	2.4E-04	yes	chr8	intron	internal intron	NA	1.00	no	down	0.50	no	1.01	no	up	4.6E-01	no
MSTRG.12059.1 : Srsf3 : A0A0U1RRV7	4.40	yes	up	1.5E-08	yes	chr20	intron	internal intron	NA	0.90	no	down	0.30	no	0.94	no	down	5.5E-08	yes
ENSRNOT00000089336 : Herc1 : A0A0G2JTT6	10.78	yes	up	2.7E-04	yes	chr8	intron	internal intron	NA	1.27	no	up	0.46	no	1.04	no	up	5.4E-02	no
MSTRG.2161.3 : Men1 : Q9WVR8	4.89	yes	up	3.4E-06	yes	chr1	exon	last exon	NA	0.94	no	down	0.81	no	1.06	no	up	2.0E-04	yes
ENSRNOT00000009207 : Mrps16 : D4A7X1	1.05	no	up	1.6E-04	yes	chr15	exon	internal exon	NA	0.49	yes	down	0.49	no	0.94	no	down	1.4E-01	no
MSTRG.13952.2 : Stau1 : Q9ESY8	0.08	yes	down	6.3E-05	yes	chr3	intron	internal intron	NA	0.99	no	down	0.92	no	1.05	no	up	2.2E-05	yes
MSTRG.2161.4 : Map4k2 : D3ZXB1	4.89	yes	up	3.4E-06	yes	chr1	intron	internal intron	NA	1.84	no	up	0.38	no	0.65	yes	down	2.4E-14	yes
ENSRNOT00000083271 : Magi1 : M0R8T1	0.38	yes	down	2.0E-04	yes	chr4	intron	internal intron	NA	1.00	no	down	0.48	no	0.99	no	down	6.0E-01	no
ENSRNOT00000093046 : Trim21 : D4ACF2	0.20	yes	down	5.7E-10	yes	chr1	promoter	Distal	LCP	0.93	no	down	0.58	no	0.96	no	down	1.3E-04	yes
ENSRNOT00000017883 : Rbpms : F2Z3S5	0.02	yes	down	2.6E-05	yes	chr16	promoter	Proximal	ICP	1.57	no	up	0.06	no	1.06	no	up	4.9E-05	yes
ENSRNOT00000006492 : Ylpm1 : A0A0G2K678	0.14	yes	down	2.3E-07	yes	chr6	intron	internal intron	NA	1.00	no	down	0.55	no	1.02	no	up	1.8E-03	yes
MSTRG.11953.15 : RT1-CE7 : D3ZLE6	inf	yes	up	7.3E-07	yes	chr20	exon	internal exon	NA	1.38	no	up	0.34	no	1.22	yes	up	7.3E-16	yes
ENSRNOT00000015172 : Mcc : F1LZS6	43.12	yes	up	1.3E-14	yes	chr1	exon	last exon	NA	0.78	no	down	0.34	no	0.85	no	down	3.7E-04	yes
MSTRG.12945.2 : Fmnl2 : A0A0G2K132	0.84	no	down	6.1E-07	yes	chr3	intron	internal intron	NA	1.58	no	up	0.23	no	1.00	no	down	9.6E-01	no
MSTRG.5906.1 : Atp2b4 : Q64542	2.31	yes	up	1.3E-05	yes	chr13	intron	internal intron	NA	0.56	no	down	0.20	no	0.68	yes	down	2.6E-19	yes
MSTRG.20817.2 : Map4k4 : A0A0G2K7W4	0.36	yes	down	7.1E-05	yes	chr9	intron	internal intron	NA	1.15	no	up	0.14	no	1.10	no	up	7.1E-06	yes
MSTRG.12184.1 : Pcnt : D3ZMY8	0.14	yes	down	8.5E-07	yes	chr20	promoter	Distal	ICP	1.15	no	up	0.72	no	0.84	no	down	9.2E-06	yes
ENSRNOT00000024609 : Uqcrfs1 : P20788	0.51	yes	down	3.6E-04	yes	chr17	exon	last exon	NA	0.73	no	down	0.23	no	0.96	no	down	2.1E-06	yes
MSTRG.16557.7 : Srrm1 : B2RYB3	2.05	yes	up	2.0E-04	yes	chr5	intron	internal intron	NA	1.92	no	up	0.16	no	1.10	no	up	2.8E-06	yes
ENSRNOT00000017254 : Rhoc : B2RYP0	0.09	yes	down	2.2E-12	yes	chr2	intron	first intron	NA	0.93	no	down	0.42	no	0.96	no	down	1.7E-03	yes
MSTRG.19538.4 : Nfrkb : D4A421	0.58	yes	down	1.3E-05	yes	chr8	intron	internal intron	NA	0.90	no	down	0.81	no	0.89	no	down	2.5E-03	yes
MSTRG.11265.27 : Ubap2l : E9PTR4	6.62	yes	up	4.0E-04	yes	chr2	intron	internal intron	NA	1.42	no	up	0.20	no	1.13	no	up	3.8E-12	yes
ENSRNOT00000027165 : Chpf : Q5XIQ8	1.40	yes	up	4.1E-04	yes	chr9	intron	internal intron	NA	1.79	no	up	0.06	no	1.00	no	down	8.4E-01	no
MSTRG.323.8 : LOC108348175 : A0A0G2JUS0	0.05	yes	down	4.0E-19	yes	chr1	promoter	Intermediate	LCP	1.35	no	up	0.67	no	1.01	no	up	4.0E-01	no
ENSRNOT00000008041 : Ctnna1 : Q5U302	2.58	yes	up	1.7E-05	yes	chr18	intron	internal intron	NA	0.93	no	down	0.53	no	0.96	no	down	3.1E-09	yes
MSTRG.8278.21 : Rbpms : F2Z3S5	0.02	yes	down	2.6E-05	yes	chr16	promoter	Proximal	ICP	1.21	no	up	0.36	no	1.06	no	up	4.9E-05	yes
ENSRNOT00000060683 : Stk11 : A0A0H2UI02	0.18	yes	down	9.1E-05	yes	chr7	exon	last exon	NA	1.18	no	up	0.18	no	0.88	no	down	1.8E-03	yes
ENSRNOT00000004140 : Plcd3 : D4A978	inf	yes	up	1.4E-05	yes	chr10	promoter	Distal	LCP	0.87	no	down	0.53	no	0.86	no	down	2.9E-08	yes
ENSRNOT00000088378 : Arhgap5 : Q6TUE6	4.16	yes	up	3.9E-08	yes	chr6	exon	first exon	NA	1.03	no	up	0.77	no	1.03	no	up	4.9E-02	yes
MSTRG.15397.3 : Stk38l : A4GW50	0.61	yes	down	1.5E-06	yes	chr4	intron	internal intron	NA	0.13	yes	down	0.15	no	1.16	no	up	1.1E-11	yes
MSTRG.3572.7 : RGD1307929 : F1LSX1	7.34	yes	up	1.1E-12	yes	chr10	intron	internal intron	NA	0.77	no	down	0.42	no	0.97	no	down	1.8E-04	yes
MSTRG.16523.5 : Arid1a : D4A3E3	0.22	yes	down	1.1E-04	yes	chr5	intron	first intron	NA	0.57	no	down	0.22	no	1.00	no	down	2.1E-01	no
ENSRNOT00000029076 : Tpm3 : Q63610	0.37	yes	down	8.0E-05	yes	chr2	intron	internal intron	NA	1.07	no	up	0.55	no	1.11	no	up	3.2E-12	yes
MSTRG.7186.1 : Sptbn1 : A0A0G2K8W9	5.72	yes	up	4.0E-04	yes	chr14	intron	internal intron	NA	3.11	yes	up	0.44	no	0.87	no	down	1.1E-18	yes
MSTRG.7804.5 : Mycbp2 : D4A2D3	0.31	yes	down	9.2E-06	yes	chr15	intron	internal intron	NA	1.10	no	up	0.47	no	1.00	no	up	6.8E-01	no
MSTRG.16606.5 : Alpl : P08289	0.52	yes	down	2.4E-04	yes	chr5	promoter	Intermediate	LCP	0.19	yes	down	0.03	yes	1.00	no	down	6.2E-01	no
MSTRG.6919.2 : Cpz : A0A0G2JSJ7	3.01	yes	up	1.1E-05	yes	chr14	intron	internal intron	NA	0.56	no	down	0.52	no	1.08	no	up	5.1E-02	no
MSTRG.5410.3 : Gtf2i : Q5U2Y1	5.31	yes	up	4.9E-06	yes	chr12	promoter	Distal	LCP	0.82	no	down	0.61	no	0.95	no	down	1.5E-07	yes
ENSRNOT00000021758 : Fer : A0A140TAC4	3.31	yes	up	2.7E-04	yes	chr9	promoter	Proximal	LCP	1.17	no	up	0.50	no	0.92	no	down	7.3E-07	yes
ENSRNOT00000006087 : Egfr : E7CXR8	0.25	yes	down	9.1E-09	yes	chr14	exon	internal exon	NA	1.33	no	up	0.08	no	0.99	no	down	2.7E-01	no
MSTRG.20742.1 : Dst : D3ZC56	0.35	yes	down	1.3E-05	yes	chr9	intron	internal intron	NA	0.64	no	down	0.18	no	1.03	no	up	7.3E-04	yes
ENSRNOT00000013375 : Eif2s1 : P68101	2.64	yes	up	8.2E-05	yes	chr6	promoter	Distal	ICP	1.19	no	up	0.19	no	0.99	no	down	3.4E-01	no
MSTRG.12337.5 : Sgpl1 : Q8CHN6	0.20	yes	down	6.8E-07	yes	chr20	intron	internal intron	NA	1.51	no	up	0.04	yes	1.12	no	up	5.6E-16	yes
MSTRG.10830.5 : Phc3 : D3ZS50	0.36	yes	down	4.0E-04	yes	chr2	intron	internal intron	NA	1.25	no	up	0.47	no	0.86	no	down	4.2E-04	yes
MSTRG.2854.1 : Abca3 : A0A0G2K1Q8	0.49	yes	down	3.5E-07	yes	chr10	intron	internal intron	NA	2.32	yes	up	0.33	no	1.05	no	up	1.5E-04	yes
ENSRNOT00000010633 : Stau1 : Q9ESY8	0.08	yes	down	6.3E-05	yes	chr3	intron	internal intron	NA	1.00	no	down	0.94	no	1.05	no	up	2.2E-05	yes
MSTRG.4263.4 : Tnrc6c : D3ZRA6	0.39	yes	down	2.3E-06	yes	chr10	intron	internal intron	NA	1.44	no	up	0.47	no	1.07	no	up	1.9E-01	no
MSTRG.18268.6 : Akap8 : Q63014	0.08	yes	down	2.7E-04	yes	chr7	intron	internal intron	NA	0.74	no	down	0.33	no	0.99	no	down	2.8E-01	no
MSTRG.17007.1 : Fam98a : Q5FWT1	0.57	yes	down	6.5E-05	yes	chr6	exon	last exon	NA	0.77	no	down	0.69	no	0.98	no	down	9.6E-02	no
MSTRG.5417.11 : Auts2 : F1M388	0.04	yes	down	4.8E-14	yes	chr12	intron	internal intron	NA	0.73	no	down	0.16	no	1.01	no	up	5.6E-01	no
MSTRG.19188.5 : Rpap3 : Q68FQ7	0.29	yes	down	4.9E-05	yes	chr7	intron	internal intron	NA	1.05	no	up	0.85	no	1.04	no	up	2.9E-04	yes
ENSRNOT00000055342 : Bpifb1 : A0JPN3	1.80	yes	up	3.8E-07	yes	chr3	intron	internal intron	NA	0.22	yes	down	0.28	no	0.76	yes	down	2.7E-18	yes
ENSRNOT00000077502 : Kmt2d : A0A0G2JVD6	6.53	yes	up	2.1E-09	yes	chr7	exon	last exon	NA	0.98	no	down	0.95	no	1.03	no	up	3.5E-02	yes
ENSRNOT00000054925 : Ogfod3 : Q5M843	3.46	yes	up	5.1E-05	yes	chr10	intron	internal intron	NA	1.14	no	up	0.56	no	1.07	no	up	1.8E-03	yes
MSTRG.123.2 : Epb41l2 : D3ZM69	0.25	yes	down	1.1E-12	yes	chr1	intron	internal intron	NA	0.95	no	down	0.86	no	0.91	no	down	8.9E-11	yes
ENSRNOT00000059814 : Exoc1 : Q4V8H2	inf	yes	up	6.1E-05	yes	chr14	intron	first intron	NA	1.04	no	up	0.48	no	0.91	no	down	3.2E-04	yes
MSTRG.11285.1 : Npr1 : P18910	0.37	yes	down	1.2E-04	yes	chr2	intron	internal intron	NA	1.98	no	up	0.43	no	1.02	no	up	1.6E-02	yes
ENSRNOT00000018246 : Cdh6 : F1LQP8	3.77	yes	up	4.7E-05	yes	chr2	intron	internal intron	NA	0.94	no	down	0.73	no	1.08	no	up	1.9E-09	yes
ENSRNOT00000066007 : Dnm2 : A0A0A0MY48	0.11	yes	down	8.9E-11	yes	chr8	intron	first intron	NA	0.86	no	down	0.52	no	1.05	no	up	2.0E-08	yes
ENSRNOT00000018734 : Cmas : Q5M963	5.02	yes	up	1.4E-04	yes	chr4	intron	internal intron	NA	1.00	no	down	0.95	no	0.86	no	down	3.1E-13	yes
ENSRNOT00000087928 : Aldoa : P05065	0.17	yes	down	1.4E-04	yes	chr1	exon	last exon	NA	1.23	no	up	0.74	no	0.98	no	down	4.2E-08	yes
ENSRNOT00000090481 : Inpp5k : A0A0G2K2Z2	8.85	yes	up	4.8E-08	yes	chr10	promoter	Distal	ICP	1.09	no	up	0.61	no	0.94	no	down	3.3E-03	yes
MSTRG.8278.37 : Rbpms : F2Z3S5	0.02	yes	down	2.6E-05	yes	chr16	promoter	Proximal	ICP	0.58	no	down	0.46	no	1.06	no	up	4.9E-05	yes
MSTRG.5399.2 : Eln : Q99372	3.55	yes	up	3.0E-04	yes	chr12	intron	internal intron	NA	0.75	no	down	0.06	no	0.82	yes	down	3.9E-08	yes
MSTRG.7350.9 : Ktn1 : D4A4Z9	2.49	yes	up	1.2E-04	yes	chr15	intron	internal intron	NA	1.13	no	up	0.35	no	1.04	no	up	2.2E-11	yes
ENSRNOT00000040629 : Tnrc6c : D3ZRA6	0.39	yes	down	2.3E-06	yes	chr10	intron	internal intron	NA	0.56	no	down	0.50	no	1.07	no	up	1.9E-01	no
MSTRG.19020.3 : Tnrc6b : A0A0G2K6R0	2.14	yes	up	4.3E-13	yes	chr7	exon	last exon	NA	1.40	no	up	0.22	no	1.01	no	up	7.4E-01	no
MSTRG.15715.4 : Dnajb5 : D3ZB76	0.05	yes	down	2.8E-05	yes	chr5	promoter	Distal	ICP	0.89	no	down	0.67	no	1.02	no	up	2.3E-01	no
MSTRG.3468.2 : Pafah1b1 : P63004	0.19	yes	down	5.9E-04	yes	chr10	intron	internal intron	NA	1.77	no	up	0.61	no	0.96	no	down	1.4E-04	yes
ENSRNOT00000083582 : Aamp : B0K024	0.09	yes	down	2.6E-04	yes	chr9	promoter	Intermediate	LCP	1.02	no	up	0.82	no	1.06	no	up	1.5E-05	yes
MSTRG.6037.1 : Npl : Q66H59	2.35	yes	up	1.9E-05	yes	chr13	intron	internal intron	NA	1.59	no	up	0.21	no	1.08	no	up	5.3E-06	yes
MSTRG.4717.1 : Adcy5 : G3V9G1	0.16	yes	down	2.3E-10	yes	chr11	intron	internal intron	NA	0.98	no	down	0.95	no	1.00	no	down	7.6E-01	no
ENSRNOT00000023628 : Hspa4 : O88600	0.10	yes	down	2.8E-04	yes	chr10	intron	internal intron	NA	1.15	no	up	0.14	no	1.19	no	up	3.6E-08	yes
ENSRNOT00000014871 : Gnb4 : O35353	2.44	yes	up	1.4E-05	yes	chr2	intron	last intron	NA	0.86	no	down	0.76	no	0.91	no	down	2.4E-06	yes
ENSRNOT00000055245 : Wls : Q6P689	0.38	yes	down	6.5E-05	yes	chr2	exon	last exon	NA	1.11	no	up	0.43	no	1.11	no	up	1.3E-07	yes
ENSRNOT00000082668 : Cdk12 : A0A0G2K5U7	5.94	yes	up	1.1E-04	yes	chr10	exon	first exon	NA	0.97	no	down	0.18	no	0.97	no	down	5.5E-04	yes
ENSRNOT00000064965 : Gigyf2 : A0A096MJI4	0.59	yes	down	1.9E-05	yes	chr9	intron	internal intron	NA	0.98	no	down	0.32	no	1.31	yes	up	2.3E-10	yes
MSTRG.18080.2 : Cd63 : Q9QZV0	0.47	yes	down	3.6E-06	yes	chr7	promoter	Intermediate	ICP	0.95	no	down	0.96	no	0.56	yes	down	4.8E-03	yes
MSTRG.3521.4 : Inpp5k : A0A0G2K2Z2	8.85	yes	up	4.8E-08	yes	chr10	promoter	Distal	ICP	1.13	no	up	0.38	no	0.94	no	down	3.3E-03	yes
MSTRG.6818.5 : Rbpj : M0R7Q3	0.34	yes	down	7.0E-05	yes	chr14	intron	last intron	NA	0.60	no	down	0.43	no	1.08	no	up	1.1E-04	yes

MSTRG.20817.14 : Map4k4 : A0A0G2K7W4	0.36	yes	down	7.1E-05	yes	chr9	intron	internal intron	NA	0.72	no	down	0.50	no	1.10	no	up	7.1E-06	yes
ENSRNOT00000055992 : Kdm1a : A0A0G2K736	0.26	yes	down	3.7E-04	yes	chr5	intron	internal intron	NA	1.09	no	up	0.05	yes	1.01	no	up	5.0E-01	no
MSTRG.16317.8 : Cap1 : Q08163	0.21	yes	down	1.6E-07	yes	chr5	intron	internal intron	NA	0.53	no	down	0.61	no	0.94	no	down	1.0E-08	yes
MSTRG.18206.3 : Cirbp : P60825	0.73	yes	down	1.3E-04	yes	chr7	exon	first exon	NA	0.80	no	down	0.38	no	0.85	no	down	3.4E-16	yes
MSTRG.9868.3 : Cc2d1a : Q66HA5	0.21	yes	down	1.7E-05	yes	chr19	promoter	Distal	LCP	0.80	no	down	0.38	no	0.96	no	down	4.1E-02	yes
ENSRNOT00000004094 : Bst1 : F1LSX3	13.53	yes	up	3.3E-04	yes	chr14	exon	last exon	NA	1.14	no	up	0.61	no	1.29	yes	up	2.4E-16	yes
ENSRNOT00000011155 : Vps39 : E9PT04	inf	yes	up	4.3E-04	yes	chr3	intron	internal intron	NA	1.10	no	up	0.41	no	1.08	no	up	2.7E-04	yes
MSTRG.9278.1 : Ercc3 : Q4G005	0.04	yes	down	1.2E-04	yes	chr18	promoter	Intermediate	ICP	1.61	no	up	0.59	no	0.97	no	down	5.7E-03	yes
MSTRG.15855.5 : RGD1306148 : F1M446	0.22	yes	down	4.0E-05	yes	chr5	intron	internal intron	NA	1.00	no	down	0.47	no	1.01	no	up	7.4E-02	no
ENSRNOT00000080604 : C1qtnf7 : B2RYB7	3.60	yes	up	9.8E-06	yes	chr14	exon	last exon	NA	0.75	no	down	0.20	no	0.87	no	down	2.3E-09	yes
MSTRG.14680.2 : Thnsl2 : Q5M7T9	3.56	yes	up	3.5E-12	yes	chr4	intron	internal intron	NA	0.98	no	down	0.93	no	0.97	no	down	4.8E-01	no
ENSRNOT00000017110 : Mfsd5 : A0A0G2KAK9	0.14	yes	down	6.3E-05	yes	chr7	intron	first intron	NA	1.97	no	up	0.43	no	1.12	no	up	3.3E-09	yes
ENSRNOT00000092172 : Rufy3 : A0A0G2K6A9	2.37	yes	up	3.9E-04	yes	chr14	promoter	Distal	LCP	1.25	no	up	0.46	no	1.11	no	up	1.3E-05	yes
ENSRNOT00000091488 : Ankmy2 : D3ZC34	2.16	yes	up	2.9E-04	yes	chr6	promoter	Distal	ICP	1.11	no	up	0.56	no	0.93	no	down	2.5E-09	yes
ENSRNOT00000028751 : Vps45 : O08700	3.11	yes	up	8.5E-06	yes	chr2	intron	internal intron	NA	1.04	no	up	0.60	no	0.99	no	down	3.7E-01	no
ENSRNOT00000011767 : Fggy : Q5FVC3	18.59	yes	up	3.3E-04	yes	chr5	intron	internal intron	NA	0.68	no	down	0.26	no	0.84	no	down	2.3E-07	yes
MSTRG.1201.4 : Ntrk3 : Q68G04	7.03	yes	up	1.5E-06	yes	chr1	intron	internal intron	NA	0.57	no	down	0.09	no	0.87	no	down	1.8E-07	yes
MSTRG.4443.2 : Ltn1 : F1M9Q3	0.38	yes	down	4.2E-04	yes	chr11	intron	internal intron	NA	0.78	no	down	0.35	no	1.08	no	up	1.4E-11	yes
ENSRNOT00000065216 : Mapk8 : A0A0G2KA63	0.22	yes	down	1.8E-04	yes	chr16	intron	internal intron	NA	0.97	no	down	0.87	no	1.06	no	up	2.4E-05	yes
MSTRG.19655.1 : Kmt2a : F1M0L3	0.53	yes	down	1.7E-05	yes	chr8	exon	internal exon	NA	0.50	yes	down	0.46	no	0.99	no	down	9.3E-01	no
ENSRNOT00000076030 : Parp4 : A0A096MK99	1.23	yes	up	1.1E-06	yes	chr15	intron	last intron	NA	0.62	no	down	0.14	no	0.94	no	down	4.1E-06	yes
MSTRG.15915.1 : Prpf4 : D4A7J8	0.23	yes	down	2.8E-04	yes	chr5	exon	last exon	NA	1.26	no	up	0.19	no	1.03	no	up	3.4E-04	yes
MSTRG.7835.2 : Tm9sf2 : Q66HG5	0.39	yes	down	9.3E-05	yes	chr15	intron	last intron	NA	6.01	yes	up	0.01	yes	1.03	no	up	6.1E-04	yes
MSTRG.20875.1 : Nab1 : Q62722	3.78	yes	up	5.6E-07	yes	chr9	exon	internal exon	NA	0.96	no	down	0.90	no	0.97	no	down	6.0E-01	no
MSTRG.15479.2 : Prex2 : A0A0G2KA11	5.57	yes	up	1.6E-04	yes	chr5	intron	internal intron	NA	0.31	yes	down	0.01	yes	1.02	no	up	2.3E-01	no
ENSRNOT00000092331 : Tecpr1 : Q3ZBA0	0.43	yes	down	3.1E-04	yes	chr12	promoter	Intermediate	LCP	1.06	no	up	0.09	no	1.17	no	up	7.7E-08	yes
MSTRG.18880.4 : Plec : Q6S3A0	0.33	yes	down	4.2E-06	yes	chr7	exon	last exon	NA	1.31	no	up	0.08	no	1.11	no	up	6.0E-08	yes
MSTRG.14930.6 : Grip2 : A0A0H2UHH8	2.18	yes	up	2.2E-05	yes	chr4	intron	internal intron	NA	0.88	no	down	0.67	no	1.01	no	up	5.2E-01	no
MSTRG.10569.5 : Plpp1 : O08564	0.47	yes	down	8.3E-05	yes	chr2	intron	internal intron	NA	0.53	no	down	0.17	no	1.04	no	up	2.9E-03	yes
MSTRG.13356.1 : Vps18 : B5DFJ4	0.55	yes	down	3.0E-04	yes	chr3	exon	internal exon	NA	0.86	no	down	0.52	no	1.01	no	up	4.9E-01	no
ENSRNOT00000079862 : Dkc1 : P40615	0.28	yes	down	2.4E-05	yes	chrX	intron	internal intron	NA	0.87	no	down	0.56	no	0.99	no	down	3.6E-01	no
MSTRG.12240.5 : Bicc1 : A0A0G2K0Y0	5.19	yes	up	5.0E-04	yes	chr20	intron	internal intron	NA	0.72	no	down	0.68	no	1.52	yes	up	1.2E-05	yes
MSTRG.12137.4 : Pknox1 : Q5BJP1	0.26	yes	down	3.4E-07	yes	chr20	promoter	Proximal	LCP	0.77	no	down	0.50	no	0.89	no	down	7.5E-04	yes
ENSRNOT00000084134 : Runx1 : Q63046	0.62	yes	down	5.4E-05	yes	chr11	intron	first intron	NA	1.11	no	up	0.48	no	1.47	yes	up	3.2E-10	yes
MSTRG.9433.5 : Rbm27 : F1M1R4	0.45	yes	down	2.5E-04	yes	chr18	intron	internal intron	NA	1.24	no	up	0.39	no	1.01	no	up	5.2E-01	no
MSTRG.13954.4 : Znfx1 : F1LMA9	20.79	yes	up	3.0E-04	yes	chr3	intron	internal intron	NA	1.58	no	up	0.10	no	0.92	no	down	1.0E-03	yes
MSTRG.20284.6 : Qrich1 : F1M4M7	3.68	yes	up	3.4E-07	yes	chr8	exon	internal exon	NA	0.67	no	down	0.07	no	1.03	no	up	2.0E-01	no
MSTRG.16517.3 : Slc9a1 : Q8R4H8	0.61	yes	down	7.2E-05	yes	chr5	intron	internal intron	NA	1.23	no	up	0.24	no	1.05	no	up	2.8E-01	no
ENSRNOT00000067928 : Ptpn18 : Q4KM54	0	yes	down	1.9E-04	yes	chr9	intron	first intron	NA	0.59	no	down	0.11	no	1.20	no	up	1.2E-08	yes
ENSRNOT00000049857 : Snrpb : B0BN51	0.11	yes	down	3.8E-04	yes	chr3	intron	last intron	NA	1.00	no	down	1.00	no	0.98	no	down	4.0E-02	yes
MSTRG.18230.1 : Arhgap45 : D4AAI2	5.87	yes	up	2.5E-04	yes	chr7	exon	internal exon	NA	1.19	no	up	0.29	no	1.20	no	up	4.5E-05	yes
ENSRNOT00000011654 : Nsfl1c : O35987	16.74	yes	up	1.8E-04	yes	chr3	intron	first intron	NA	1.01	no	up	0.94	no	0.98	no	down	1.6E-03	yes
MSTRG.16771.1 : H6pd : D4A7D7	0.22	yes	down	2.6E-06	yes	chr5	intron	last intron	NA	1.40	no	up	0.65	no	1.14	no	up	9.6E-15	yes
MSTRG.4725.9 : Kalrn : P97924	1.54	yes	up	9.1E-07	yes	chr11	exon	last exon	NA	1.14	no	up	0.09	no	1.04	no	up	1.6E-04	yes
MSTRG.2839.12 : Srrm2 : A0A0G2K2M9	1.72	yes	up	9.0E-05	yes	chr10	exon	internal exon	NA	1.00	no	down	0.50	no	1.00	no	up	9.0E-01	no
MSTRG.10426.1 : Scamp1 : A0A0G2K1I6	11.34	yes	up	2.9E-04	yes	chr2	intron	first intron	NA	1.61	no	up	0.09	no	1.05	no	up	3.2E-06	yes
MSTRG.11953.27 : RT1-CE7 : D3ZLE6	inf	yes	up	7.3E-07	yes	chr20	exon	internal exon	NA	0.65	no	down	0.32	no	1.22	yes	up	7.3E-16	yes
MSTRG.9728.4 : Cnot1 : G3V7M0	0.08	yes	down	2.6E-04	yes	chr19	intron	internal intron	NA	0.75	no	down	0.67	no	1.01	no	up	1.2E-01	no
MSTRG.19329.3 : Atf7 : B0BMY0	0.38	yes	down	7.2E-06	yes	chr7	intron	internal intron	NA	1.19	no	up	0.09	no	1.00	no	down	9.7E-01	no
MSTRG.21170.2 : Gigyf2 : A0A096MJI4	0.59	yes	down	1.9E-05	yes	chr9	intron	internal intron	NA	0.96	no	down	0.71	no	1.31	yes	up	2.3E-10	yes
MSTRG.13838.2 : Ctnnbl1 : Q4V8K2	5.63	yes	up	4.6E-05	yes	chr3	intron	first intron	NA	1.04	no	up	0.92	no	1.01	no	up	3.8E-01	no
ENSRNOT00000083108 : Cmtr1 : Q5U2Z5	4.09	yes	up	4.3E-15	yes	chr20	intron	internal intron	NA	0.99	no	down	0.88	no	1.00	no	down	9.7E-01	no
ENSRNOT00000091063 : Atl2 : F1LQ09	2.32	yes	up	2.9E-04	yes	chr6	intron	last intron	NA	1.00	no	down	0.97	no	0.95	no	down	8.9E-05	yes
MSTRG.20998.3 : Pikfyve : D3ZYT8	0.33	yes	down	2.0E-04	yes	chr9	intron	internal intron	NA	2.44	yes	up	0.09	no	1.22	yes	up	6.5E-08	yes
MSTRG.4508.4 : Dyrk1a : Q63470	0.26	yes	down	2.2E-05	yes	chr11	intron	internal intron	NA	0.56	no	down	0.60	no	1.02	no	up	1.6E-01	no
MSTRG.10517.5 : Erbin : M0R9T2	3.86	yes	up	2.1E-05	yes	chr2	intron	last intron	NA	0.64	no	down	0.28	no	0.94	no	down	3.9E-05	yes
MSTRG.6818.4 : Rbpj : M0R7Q3	0.34	yes	down	7.0E-05	yes	chr14	intron	last intron	NA	0.53	no	down	0.32	no	1.08	no	up	1.1E-04	yes
ENSRNOT00000078645 : Calcrl : Q63118	2.81	yes	up	1.2E-04	yes	chr3	intron	internal intron	NA	0.90	no	down	0.75	no	1.09	no	up	1.2E-04	yes
ENSRNOT00000051000 : Tarsl2 : Q5XI17	0.35	yes	down	1.8E-06	yes	chr1	intron	internal intron	NA	1.00	no	down	0.87	no	1.01	no	up	6.4E-01	no
ENSRNOT00000001669 : Gstt1 : Q01579	0.44	yes	down	5.4E-04	yes	chr20	exon	last exon	NA	0.92	no	down	0.80	no	0.80	yes	down	1.4E-15	yes
MSTRG.5972.1 : Ptprc : P04157	2.98	yes	up	6.9E-14	yes	chr13	intron	internal intron	NA	1.23	no	up	0.83	no	1.12	no	up	6.1E-15	yes
MSTRG.19969.4 : Myzap : Q5EB94	0.16	yes	down	3.6E-06	yes	chr8	exon	last exon	NA	0.72	no	down	0.16	no	0.84	no	down	4.4E-10	yes
MSTRG.20742.4 : Dst : D3ZC56	0.35	yes	down	1.3E-05	yes	chr9	intron	internal intron	NA	2.19	yes	up	0.15	no	1.03	no	up	7.3E-04	yes
MSTRG.7804.7 : Mycbp2 : D4A2D3	0.31	yes	down	9.2E-06	yes	chr15	intron	internal intron	NA	0.48	yes	down	0.23	no	1.00	no	up	6.8E-01	no
MSTRG.17628.1 : Ahsa1 : B0BN63	0.11	yes	down	3.3E-06	yes	chr6	exon	last exon	NA	1.27	no	up	0.20	no	1.00	no	up	4.2E-01	no
ENSRNOT00000091980 : Clasp2 : A0A0G2JZM8	0.25	yes	down	1.3E-05	yes	chr8	intron	internal intron	NA	0.94	no	down	0.50	no	0.98	no	down	1.8E-01	no
ENSRNOT00000091840 : Plec : F7F9U6	0.33	yes	down	4.2E-06	yes	chr7	exon	last exon	NA	1.01	no	up	0.04	yes	0.90	no	down	2.5E-03	yes
MSTRG.20218.4 : Rpl29 : P25886	3.86	yes	up	3.0E-04	yes	chr8	intron	first intron	NA	5.06	yes	up	0.20	no	0.98	no	down	6.0E-01	no
MSTRG.10007.3 : Ctcf : Q9R1D1	2.29	yes	up	1.4E-04	yes	chr19	intron	internal intron	NA	0.77	no	down	0.40	no	0.94	no	down	4.5E-07	yes
ENSRNOT00000093182 : Eftud2 : F1LM66	4.66	yes	up	6.0E-04	yes	chr10	exon	internal exon	NA	0.95	no	down	0.61	no	1.02	no	up	4.9E-02	yes
MSTRG.8278.43 : Rbpms : F2Z3S5	0.02	yes	down	2.6E-05	yes	chr16	promoter	Proximal	ICP	0.50	yes	down	0.12	no	1.06	no	up	4.9E-05	yes
MSTRG.18557.13 : Mon2 : D3ZCG3	0.07	yes	down	1.5E-08	yes	chr7	intron	internal intron	NA	1.53	no	up	0.09	no	1.05	no	up	7.1E-07	yes
MSTRG.20875.2 : Nab1 : Q62722	3.78	yes	up	5.6E-07	yes	chr9	exon	internal exon	NA	1.00	no	down	0.48	no	0.97	no	down	6.0E-01	no
ENSRNOT00000055963 : Slfn13 : A0A096MKD0	2.27	yes	up	1.5E-04	yes	chr10	promoter	Intermediate	LCP	2.32	yes	up	0.27	no	0.97	no	down	2.7E-02	yes
MSTRG.16007.1 : Dennd4c : F1LTD7	0.31	yes	down	2.0E-04	yes	chr5	intron	internal intron	NA	1.34	no	up	0.62	no	0.97	no	down	9.0E-02	no
ENSRNOT00000090725 : Tspan9 : D4AAV9	0.41	yes	down	1.4E-09	yes	chr4	exon	last exon	NA	0.95	no	down	0.77	no	0.96	no	down	2.4E-05	yes
ENSRNOT00000087548 : Triobp : A2TIS7	0.29	yes	down	2.0E-06	yes	chr7	intron	internal intron	NA	0.96	no	down	0.46	no	0.97	no	down	5.1E-06	yes
MSTRG.10716.1 : Trio : F1M0Z1	0.35	yes	down	1.6E-06	yes	chr2	exon	first exon	NA	0.66	no	down	0.31	no	1.02	no	up	2.1E-02	yes
MSTRG.7804.1 : Mycbp2 : D4A2D3	0.31	yes	down	9.2E-06	yes	chr15	intron	internal intron	NA	1.03	no	up	0.49	no	1.00	no	up	6.8E-01	no
MSTRG.17738.1 : Vrk1 : Q6AYA2	0.22	yes	down	2.5E-07	yes	chr6	intron	internal intron	NA	0.71	no	down	0.44	no	1.01	no	up	2.7E-01	no
MSTRG.9047.2 : Upf2 : D3ZT03	0.21	yes	down	9.8E-07	yes	chr17	intron	internal intron	NA	1.08	no	up	0.41	no	1.03	no	up	3.0E-03	yes
ENSRNOT00000015452 : Mtmr9 : Q8K3W5	2.60	yes	up	2.7E-05	yes	chr15	exon	last exon	NA	1.12	no	up	0.10	no	0.93	no	down	1.2E-04	yes
MSTRG.15715.5 : Dnajb5 : D3ZB76	0.05	yes	down	2.8E-05	yes	chr5	promoter	Distal	ICP	0.55	no	down	0.02	yes	1.02	no	up	2.3E-01	no
MSTRG.2839.10 : Srrm2 : A0A0G2K2M9	1.72	yes	up	9.0E-05	yes	chr10	exon	internal exon	NA	0.75	no	down	0.63	no	1.00	no	up	9.0E-01	no
MSTRG.19220.4 : Kmt2d : A0A0G2JVD6	6.53	yes	up	2.1E-09	yes	chr7	exon	last exon	NA	1.58	no	up	0.48	no	1.03	no	up	3.5E-02	yes
MSTRG.17183.2 : Rock2 : F1LQT3	0.10	yes	down	2.0E-24	yes	chr6	intron	internal intron	NA	0.69	no	down	0.50	no	0.97	no	down	1.9E-04	yes
ENSRNOT00000079700 : Abca3 : A0A0G2K1Q8	0.49	yes	down	3.5E-07	yes	chr10	intron	internal intron	NA	0.50	yes	down	0.18	no	1.05	no	up	1.5E-04	yes
MSTRG.19042.3 : Aco2 : Q9ER34	0	yes	down	2.0E-04	yes	chr7	promoter	Distal	ICP	0.80	no	down	0.49	no	0.90	no	down	5.3E-13	yes
MSTRG.8278.12 : Rbpms : F2Z3S5	0.02	yes	down	2.6E-05	yes	chr16	promoter	Proximal	ICP	1.29	no	up	0.31	no	1.06	no	up	4.9E-05	yes
MSTRG.13952.1 : Stau1 : Q9ESY8	0.08	yes	down	6.3E-05	yes	chr3	intron	internal intron	NA	1.13	no	up	0.61	no	1.05	no	up	2.2E-05	yes
MSTRG.5400.1 : Eln : A0A0G2JST5	3.55	yes	up	3.0E-04	yes	chr12	intron	internal intron	NA	0.18	yes	down	0.17	no	0.91	no	down	1.2E-01	no
MSTRG.4038.3 : Plcd3 : D4A978	inf	yes	up	1.4E-05	yes	chr10	promoter	Distal	LCP	0.84	no	down	0.05	yes	0.86	no	down	2.9E-08	yes
MSTRG.16339.3 : Fhl3 : D3ZPF0	0.20	yes	down	2.5E-09	yes	chr5	exon	last exon	NA	0.81	no	down	0.18	no	1.28	yes	up	1.8E-14	yes
ENSRNOT00000001968 : Pom121 : G3V659	0.10	yes	down	1.6E-23	yes	chr12	intron	first intron	NA	0.91	no	down	0.55	no	1.02	no	up	1.8E-01	no
ENSRNOT00000024617 : Tpm1 : Q923Z2	9.41	yes	up	5.1E-14	yes	chr8	intron	internal intron	NA	0.67	no	down	0.44	no	0.75	yes	down	6.0E-18	yes
ENSRNOT00000009994 : Rac2 : Q5U1Y2	6.06	yes	up	4.2E-04	yes	chr7	intron	internal intron	NA	1.27	no	up	0.38	no	1.09	no	up	2.6E-04	yes
MSTRG.15963.3 : Ptprd : M0RB22	3.73	yes	up	1.3E-08	yes	chr5	exon	internal exon	NA	1.12	no	up	0.59	no	1.01	no	up	5.3E-01	no
ENSRNOT00000092353 : Lamp1 : P14562	0.27	yes	down	5.9E-08	yes	chr16	promoter	Distal	LCP	1.13	no	up	0.70	no	1.10	no	up	5.4E-06	yes
ENSRNOT00000091840 : Plec : Q6S3A0	0.33	yes	down	4.2E-06	yes	chr7	exon	last exon	NA	1.01	no	up	0.04	yes	1.11	no	up	6.0E-08	yes
MSTRG.19148.1 : Shank3 : A0A0U1RS13	3.23	yes	up	4.3E-05	yes	chr7	exon	first exon	NA	0.49	yes	down	0.12	no	0.98	no	down	1.0E-02	yes

MSTRG.18268.12 : Akap8 : Q63014	0.08	yes	down	2.7E-04	yes	chr7	intron	internal intron	NA	1.20	no	up	0.33	no	0.99	no	down	2.8E-01	no
MSTRG.19777.5 : Sin3a : A0A0G2K3H5	0.53	yes	down	4.1E-06	yes	chr8	intron	last intron	NA	0.68	no	down	0.45	no	0.96	no	down	6.2E-05	yes
MSTRG.1334.1 : Dlg2 : F1M907	2.35	yes	up	1.4E-05	yes	chr1	intron	internal intron	NA	1.75	no	up	0.27	no	0.92	no	down	2.8E-06	yes
MSTRG.14075.3 : Dnajc5 : A0A0G2JX56	0.30	yes	down	1.1E-06	yes	chr3	promoter	Intermediate	HCP	0.76	no	down	0.11	no	0.89	no	down	4.0E-08	yes
MSTRG.1443.2 : Arap1 : F1LM60	2.71	yes	up	3.3E-04	yes	chr1	intron	first intron	NA	1.37	no	up	0.34	no	0.99	no	down	2.5E-02	yes
ENSRNOT00000064965 : Gigyf2 : A0A096MKC0	0.59	yes	down	1.9E-05	yes	chr9	intron	internal intron	NA	0.98	no	down	0.32	no	1.01	no	up	7.0E-01	no
MSTRG.2768.1 : Usp7 : F1LM09	inf	yes	up	5.0E-04	yes	chr10	intron	internal intron	NA	1.00	no	down	0.45	no	1.00	no	down	7.8E-01	no
MSTRG.4105.1 : Smurf2 : F1M3F2	3.77	yes	up	9.3E-06	yes	chr10	intron	internal intron	NA	1.43	no	up	0.03	yes	1.03	no	up	6.3E-02	no
MSTRG.21600.1 : Acot9 : Q5U2X8	0.09	yes	down	5.0E-06	yes	chrX	promoter	Distal	LCP	1.21	no	up	0.57	no	0.99	no	down	5.1E-02	no
ENSRNOT00000016948 : Bag2 : B0BN74	0.18	yes	down	4.5E-04	yes	chr9	promoter	Distal	ICP	0.91	no	down	0.61	no	1.05	no	up	1.1E-05	yes
MSTRG.4890.1 : Dvl3 : D4ADV8	0.50	yes	down	1.6E-05	yes	chr11	intron	first intron	NA	0.73	no	down	0.40	no	0.98	no	down	2.0E-01	no
MSTRG.2161.2 : Men1 : Q9WVR8	4.89	yes	up	3.4E-06	yes	chr1	exon	last exon	NA	1.01	no	up	0.97	no	1.06	no	up	2.0E-04	yes
MSTRG.19173.1 : Ano6 : A0A0G2K1M7	3.87	yes	up	9.8E-09	yes	chr7	intron	internal intron	NA	0.87	no	down	0.27	no	0.91	no	down	2.1E-09	yes
ENSRNOT00000001829 : Taf6 : Q498R0	0.74	yes	down	2.2E-06	yes	chr12	intron	internal intron	NA	1.20	no	up	0.53	no	0.91	no	down	1.8E-03	yes
ENSRNOT00000021816 : Ankfy1 : D4A1J6	0.33	yes	down	7.9E-05	yes	chr10	intron	internal intron	NA	0.82	no	down	0.74	no	0.97	no	down	1.1E-04	yes
MSTRG.8278.47 : Rbpms : F2Z3S5	0.02	yes	down	2.6E-05	yes	chr16	promoter	Proximal	ICP	1.11	no	up	0.35	no	1.06	no	up	4.9E-05	yes
MSTRG.12922.3 : Zeb2 : A0A0G2K8T6	0.34	yes	down	8.0E-09	yes	chr3	intron	internal intron	NA	1.49	no	up	0.46	no	1.01	no	up	2.4E-01	no
ENSRNOT00000055120 : Pik3c2a : D3ZTF6	0.07	yes	down	5.5E-05	yes	chr1	intron	internal intron	NA	0.87	no	down	0.64	no	1.06	no	up	1.3E-06	yes
ENSRNOT00000049657 : Grip2 : A0A0H2UHH8	2.18	yes	up	2.2E-05	yes	chr4	intron	internal intron	NA	1.52	no	up	0.48	no	1.01	no	up	5.2E-01	no
ENSRNOT00000027512 : Pdcd11 : D3ZNI3	0.34	yes	down	5.3E-07	yes	chr1	exon	internal exon	NA	1.01	no	up	0.93	no	1.08	no	up	5.5E-06	yes
ENSRNOT00000032843 : Pde3a : Q62865	0.30	yes	down	3.1E-07	yes	chr4	intron	internal intron	NA	1.31	no	up	0.32	no	1.00	no	up	9.7E-01	no
ENSRNOT00000028328 : Bax : Q9JKL3	8.53	yes	up	7.6E-07	yes	chr1	promoter	Distal	LCP	1.04	no	up	0.85	no	1.07	no	up	5.8E-08	yes
MSTRG.123.5 : Epb41l2 : D3ZM69	0.25	yes	down	1.1E-12	yes	chr1	intron	internal intron	NA	0.98	no	down	0.95	no	0.91	no	down	8.9E-11	yes
MSTRG.2433.3 : Rnls : Q5U2W9	0.31	yes	down	2.4E-05	yes	chr1	promoter	Distal	LCP	1.43	no	up	0.07	no	1.02	no	up	3.0E-01	no
MSTRG.20817.10 : Map4k4 : A0A0G2K7W4	0.36	yes	down	7.1E-05	yes	chr9	intron	internal intron	NA	1.13	no	up	0.47	no	1.10	no	up	7.1E-06	yes
MSTRG.10163.3 : Cdh13 : Q8R490	2.07	yes	up	3.3E-06	yes	chr19	promoter	Intermediate	LCP	1.08	no	up	0.77	no	0.83	yes	down	2.3E-16	yes
ENSRNOT00000062054 : Cast : F1LPH1	0.36	yes	down	1.6E-06	yes	chr2	intron	internal intron	NA	1.24	no	up	0.08	no	0.95	no	down	2.4E-10	yes
MSTRG.11263.4 : Atp8b2 : D4A509	0.14	yes	down	3.1E-04	yes	chr2	exon	first exon	NA	1.27	no	up	0.47	no	0.92	no	down	5.0E-03	yes
MSTRG.16640.9 : Arhgef10l : M0R7W2	2.87	yes	up	9.9E-07	yes	chr5	intron	internal intron	NA	0.59	no	down	0.45	no	1.13	no	up	8.4E-04	yes
ENSRNOT00000039186 : Mettl9 : G0Z7P9	inf	yes	up	1.6E-04	yes	chr1	intron	first intron	NA	1.19	no	up	0.50	no	0.99	no	down	7.9E-01	no
MSTRG.21202.5 : Lrrfip1 : Q66HF9	6.49	yes	up	4.9E-10	yes	chr9	exon	last exon	NA	0.93	no	down	0.42	no	1.15	no	up	2.1E-11	yes
ENSRNOT00000032535 : Rasip1 : B5DF05	6.95	yes	up	1.3E-05	yes	chr1	intron	first intron	NA	1.12	no	up	0.74	no	0.96	no	down	1.3E-02	yes
MSTRG.4890.3 : Dvl3 : D4ADV8	0.50	yes	down	1.6E-05	yes	chr11	intron	first intron	NA	1.83	no	up	0.09	no	0.98	no	down	2.0E-01	no
ENSRNOT00000092464 : Tsr1 : D3ZEM8	45.59	yes	up	3.4E-04	yes	chr10	intron	internal intron	NA	1.18	no	up	0.52	no	1.00	no	down	9.7E-01	no
ENSRNOT00000029885 : Ddx46 : Q62780	0.56	yes	down	3.4E-04	yes	chr17	intron	internal intron	NA	1.35	no	up	0.14	no	0.98	no	down	1.3E-01	no
MSTRG.5417.2 : Auts2 : F1M388	0.04	yes	down	4.8E-14	yes	chr12	intron	internal intron	NA	1.18	no	up	0.32	no	1.01	no	up	5.6E-01	no
MSTRG.9749.1 : Ccdc102a : D3ZSR7	0.89	no	down	6.8E-05	yes	chr19	intron	internal intron	NA	0.84	no	down	0.01	yes	0.95	no	down	3.2E-05	yes
MSTRG.16606.8 : Alpl : P08289	0.52	yes	down	2.4E-04	yes	chr5	promoter	Intermediate	LCP	1.00	no	down	0.50	no	1.00	no	down	6.2E-01	no
MSTRG.11964.3 : Ddx39b : Q63413	0.02	yes	down	4.7E-11	yes	chr20	exon	last exon	NA	0.52	no	down	0.27	no	1.01	no	up	2.0E-01	no
ENSRNOT00000026617 : Gnpat : Q9ES71	inf	yes	up	4.2E-12	yes	chr19	exon	last exon	NA	1.14	no	up	0.11	no	1.10	no	up	6.9E-05	yes
ENSRNOT00000086892 : Dhx8 : A0A0G2K283	0.28	yes	down	3.7E-05	yes	chr10	intron	internal intron	NA	1.01	no	up	0.94	no	0.94	no	down	1.2E-01	no
ENSRNOT00000014470 : Med12l : A0A0G2JV69	6.48	yes	up	1.3E-08	yes	chr2	promoter	Distal	LCP	1.00	no	down	0.83	no	0.61	yes	down	2.2E-04	yes
MSTRG.6732.3 : Limch1 : F1M392	1.09	no	up	3.3E-04	yes	chr14	intron	last intron	NA	1.56	no	up	0.56	no	0.85	no	down	1.1E-13	yes
ENSRNOT00000025925 : St13 : P50503	0.44	yes	down	2.0E-04	yes	chr7	intron	internal intron	NA	1.01	no	up	0.94	no	0.92	no	down	6.9E-08	yes
MSTRG.21083.4 : Speg : Q63638	0.17	yes	down	2.6E-06	yes	chr9	intron	internal intron	NA	1.12	no	up	0.55	no	0.88	no	down	2.3E-07	yes
MSTRG.4725.6 : Kalrn : P97924	1.54	yes	up	9.1E-07	yes	chr11	exon	last exon	NA	0.97	no	down	0.85	no	1.04	no	up	1.6E-04	yes
MSTRG.6127.1 : Fmo2 : G3V6F6	2.66	yes	up	2.1E-11	yes	chr13	intron	internal intron	NA	1.17	no	up	0.48	no	1.21	yes	up	2.2E-15	yes
MSTRG.5482.21 : Ncor2 : A0A0G2JU91	2.16	yes	up	1.6E-06	yes	chr12	intron	internal intron	NA	0.87	no	down	0.66	no	1.01	no	up	7.1E-01	no
MSTRG.13843.2 : RGD1563354 : F1M4P5	0.18	yes	down	5.6E-06	yes	chr3	promoter	Distal	LCP	0.70	no	down	0.11	no	0.66	yes	down	1.6E-11	yes
ENSRNOT00000041354 : Atp2b1 : P11505	4.09	yes	up	4.6E-11	yes	chr7	exon	last exon	NA	1.18	no	up	0.40	no	1.04	no	up	4.5E-06	yes
MSTRG.19895.2 : Hacd3 : D4ABI7	0.11	yes	down	1.7E-06	yes	chr8	intron	internal intron	NA	1.00	no	down	0.05	yes	1.02	no	up	6.2E-05	yes
ENSRNOT00000018622 : Rbp1 : P02696	0.28	yes	down	1.7E-12	yes	chr8	exon	internal exon	NA	1.06	no	up	0.79	no	1.03	no	up	1.2E-01	no
MSTRG.19220.3 : Kmt2d : A0A0G2JVD6	6.53	yes	up	2.1E-09	yes	chr7	exon	last exon	NA	0.87	no	down	0.73	no	1.03	no	up	3.5E-02	yes
MSTRG.2010.5 : Ppp6r3 : D3ZBT9	3.21	yes	up	8.1E-07	yes	chr1	intron	internal intron	NA	0.76	no	down	0.41	no	0.98	no	down	7.0E-03	yes
MSTRG.14680.3 : Thnsl2 : Q5M7T9	3.56	yes	up	3.5E-12	yes	chr4	intron	internal intron	NA	1.09	no	up	0.70	no	0.97	no	down	4.8E-01	no
MSTRG.7082.2 : Grb10 : D3ZEA3	0.35	yes	down	3.4E-07	yes	chr14	intron	internal intron	NA	1.24	no	up	0.61	no	1.09	no	up	4.5E-06	yes
MSTRG.4871.1 : Ephb3 : D3ZH39	1.63	yes	up	6.2E-06	yes	chr11	intron	internal intron	NA	1.42	no	up	0.21	no	0.86	no	down	1.2E-07	yes
MSTRG.10569.1 : Plpp1 : O08564	0.47	yes	down	8.3E-05	yes	chr2	intron	internal intron	NA	0.94	no	down	0.40	no	1.04	no	up	2.9E-03	yes
MSTRG.3447.6 : Atp2a3 : G3V9U7	inf	yes	up	2.1E-05	yes	chr10	intron	internal intron	NA	1.22	no	up	0.12	no	0.77	yes	down	1.7E-16	yes
ENSRNOT00000081021 : Plec : Q6S395	0.33	yes	down	4.2E-06	yes	chr7	exon	last exon	NA	0.97	no	down	0.44	no	1.03	no	up	2.2E-01	no
ENSRNOT00000010603.1 : Nnt : Q5BJZ3	3.00	yes	up	1.8E-04	yes	chr2	intron	internal intron	NA	0.90	no	down	0.70	no	0.96	no	down	1.2E-07	yes
MSTRG.18523.1 : Slc35e3 : B2GUZ8	8.31	yes	up	7.9E-07	yes	chr7	promoter	Intermediate	LCP	1.40	no	up	0.62	no	1.18	no	up	2.7E-09	yes
ENSRNOT00000078562 : Man2b2 : A0A0G2K8F6	9.92	yes	up	3.6E-07	yes	chr14	intron	internal intron	NA	0.54	no	down	0.03	yes	1.08	no	up	4.2E-05	yes
MSTRG.16327.7 : Macf1 : A0A0G2K9T4	0.36	yes	down	1.3E-05	yes	chr5	exon	last exon	NA	0.88	no	down	0.84	no	0.97	no	down	9.0E-11	yes
MSTRG.4525.8 : Mx1 : Q499S4	3.65	yes	up	1.1E-13	yes	chr11	intron	internal intron	NA	1.09	no	up	0.91	no	1.36	yes	up	1.2E-13	yes
ENSRNOT00000016369 : Rab27b : Q99P74	0	yes	down	1.4E-04	yes	chr18	promoter	Distal	LCP	0.89	no	down	0.22	no	1.04	no	up	7.7E-02	no
MSTRG.5417.8 : Auts2 : F1M388	0.04	yes	down	4.8E-14	yes	chr12	intron	internal intron	NA	0.84	no	down	0.62	no	1.01	no	up	5.6E-01	no
MSTRG.13910.1 : Pigt : D4A604	29.04	yes	up	1.4E-06	yes	chr3	intron	internal intron	NA	1.22	no	up	0.11	no	1.02	no	up	5.9E-02	no
MSTRG.9728.3 : Cnot1 : G3V7M0	0.08	yes	down	2.6E-04	yes	chr19	intron	internal intron	NA	0.94	no	down	0.93	no	1.01	no	up	1.2E-01	no
MSTRG.12922.4 : Zeb2 : A0A0G2K8T6	0.34	yes	down	8.0E-09	yes	chr3	intron	internal intron	NA	0.73	no	down	0.71	no	1.01	no	up	2.4E-01	no
ENSRNOT00000038612 : Aco2 : Q9ER34	0	yes	down	2.0E-04	yes	chr7	promoter	Distal	ICP	1.07	no	up	0.49	no	0.90	no	down	5.3E-13	yes
MSTRG.8514.2 : Zcchc6 : D3ZKR9	0.14	yes	down	1.2E-08	yes	chr17	exon	internal exon	NA	1.11	no	up	0.68	no	1.12	no	up	2.3E-06	yes
MSTRG.9846.2 : Pkn1 : Q63433	1.53	yes	up	6.1E-04	yes	chr19	intron	internal intron	NA	1.40	no	up	0.40	no	1.01	no	up	5.4E-01	no
ENSRNOT00000079667 : Mcc : F1LZS6	43.12	yes	up	1.3E-14	yes	chr1	exon	last exon	NA	0.91	no	down	0.40	no	0.85	no	down	3.7E-04	yes
MSTRG.5399.3 : Eln : Q99372	3.55	yes	up	3.0E-04	yes	chr12	intron	internal intron	NA	0.82	no	down	0.64	no	0.82	yes	down	3.9E-08	yes
MSTRG.9429.1 : Mcc : F1LZS6	43.12	yes	up	1.3E-14	yes	chr1	exon	last exon	NA	1.24	no	up	0.56	no	0.85	no	down	3.7E-04	yes
MSTRG.15046.1 : Sec13 : Q5XFW8	0.17	yes	down	8.7E-10	yes	chr4	intron	internal intron	NA	0.57	no	down	0.43	no	1.04	no	up	9.2E-06	yes
ENSRNOT00000018649 : Dsp : F1LMV6	inf	yes	up	5.3E-04	yes	chr17	intron	internal intron	NA	0.72	no	down	0.26	no	1.01	no	up	5.1E-01	no
ENSRNOT00000025784 : Slc7a5 : Q63016	0.19	yes	down	8.7E-05	yes	chr19	exon	last exon	NA	0.50	yes	down	0.24	no	1.01	no	up	6.5E-01	no
ENSRNOT00000031988 : Tln2 : D3ZA84	3.91	yes	up	1.0E-11	yes	chr8	intron	internal intron	NA	1.01	no	up	0.17	no	0.87	no	down	7.8E-17	yes
MSTRG.6211.1 : Fcgr2b : A3RLA8	0.44	yes	down	2.8E-04	yes	chr13	promoter	Distal	LCP	1.24	no	up	0.50	no	1.04	no	up	4.3E-03	yes
MSTRG.15089.1 : Csgalnact2 : D4A5Z0	2.74	yes	up	6.9E-07	yes	chr4	intron	internal intron	NA	0.79	no	down	0.53	no	0.94	no	down	1.2E-02	yes
MSTRG.18451.3 : Atp2b1 : P11505	4.09	yes	up	4.6E-11	yes	chr7	exon	last exon	NA	0.34	yes	down	0.01	yes	1.04	no	up	4.5E-06	yes
MSTRG.10475.2 : Tnpo1 : F1LQP9	24.87	yes	up	1.6E-05	yes	chr2	exon	internal exon	NA	1.72	no	up	0.65	no	0.98	no	down	5.8E-02	no
ENSRNOT00000021096 : Pabpn1 : G3V7Z8	0.52	yes	down	3.9E-04	yes	chr15	intron	last intron	NA	0.99	no	down	0.90	no	1.02	no	up	3.4E-02	yes
ENSRNOT00000001546 : Coq5 : Q4G064	1.40	yes	up	5.3E-04	yes	chr12	intron	internal intron	NA	1.03	no	up	0.75	no	1.01	no	up	6.8E-01	no
MSTRG.21083.2 : Speg : Q63638	0.17	yes	down	2.6E-06	yes	chr9	intron	internal intron	NA	0.89	no	down	0.79	no	0.88	no	down	2.3E-07	yes
MSTRG.16557.2 : Srrm1 : B2RYB3	2.05	yes	up	2.0E-04	yes	chr5	intron	internal intron	NA	1.31	no	up	0.42	no	1.10	no	up	2.8E-06	yes
ENSRNOT00000039876 : Mx1 : Q499S4	3.65	yes	up	1.1E-13	yes	chr11	intron	internal intron	NA	0.94	no	down	0.29	no	1.36	yes	up	1.2E-13	yes
MSTRG.6685.1 : Exoc1 : Q4V8H2	inf	yes	up	6.1E-05	yes	chr14	intron	first intron	NA	1.28	no	up	0.02	yes	0.91	no	down	3.2E-04	yes
MSTRG.20879.1 : Stat1 : F1M9D6	0.50	yes	down	1.5E-10	yes	chr9	intron	first intron	NA	1.56	no	up	0.05	yes	1.10	no	up	1.4E-13	yes
ENSRNOT00000073860 : Larp7 : M0R7D1	0.06	yes	down	3.4E-04	yes	chr2	exon	last exon	NA	1.00	no	down	0.40	no	1.07	no	up	2.0E-06	yes
ENSRNOT00000038649 : Hadha : Q64428	0.24	yes	down	2.1E-04	yes	chr6	intron	internal intron	NA	1.04	no	up	0.61	no	0.94	no	down	2.3E-10	yes
ENSRNOT00000041839 : Ntrk3 : Q68G04	7.03	yes	up	1.5E-06	yes	chr1	intron	internal intron	NA	0.92	no	down	0.62	no	0.87	no	down	1.8E-07	yes
ENSRNOT00000076426 : Asph : A0A096MKE0	2.56	yes	up	9.8E-06	yes	chr5	intron	internal intron	NA	0.70	no	down	0.24	no	0.99	no	down	1.3E-01	no
ENSRNOT00000081997 : Zswim8 : A0A0G2K9R0	0.41	yes	down	1.3E-06	yes	chr15	intron	last intron	NA	1.03	no	up	0.87	no	0.92	no	down	1.2E-05	yes
ENSRNOT00000082164 : Plin1 : F1LSF5	2.99	yes	up	9.0E-13	yes	chr1	intron	internal intron	NA	0.95	no	down	0.64	no	1.08	no	up	8.1E-05	yes
ENSRNOT00000082010 : Becn1 : Q91XJ1	0.15	yes	down	1.2E-04	yes	chr10	exon	first exon	NA	1.40	no	up	0.21	no	0.97	no	down	9.2E-02	no
MSTRG.20350.3 : Clasp2 : A0A0G2JZM8	0.25	yes	down	1.3E-05	yes	chr8	intron	internal intron	NA	0.56	no	down	0.33	no	0.98	no	down	1.8E-01	no
MSTRG.22038.8 : Fmr1 : Q80WE1	inf	yes	up	3.0E-04	yes	chrX	intron	internal intron	NA	0.99	no	down	0.50	no	1.09	no	up	7.3E-06	yes

ENSRNOT00000011223 : Nfrkb : D4A421	0.58	yes	down	1.3E-05	yes	chr8	intron	internal intron	NA	0.97	no	down	0.50	no	0.89	no	down	2.5E-03	yes
ENSRNOT00000091493 : Siglec1 : A0A0G2K320	0.24	yes	down	6.2E-06	yes	chr3	promoter	Proximal	LCP	1.42	no	up	0.03	yes	1.25	yes	up	7.1E-13	yes
MSTRG.9900.1 : Rnaseh2a : Q5U209	14.16	yes	up	1.1E-05	yes	chr19	intron	last intron	NA	0.83	no	down	0.62	no	1.08	no	up	2.6E-05	yes
MSTRG.7350.3 : Ktn1 : D4A4Z9	2.49	yes	up	1.2E-04	yes	chr15	intron	internal intron	NA	0.93	no	down	0.40	no	1.04	no	up	2.2E-11	yes
MSTRG.15096.1 : Cacna1c : F1MA84	4.75	yes	up	4.3E-04	yes	chr4	intron	internal intron	NA	0.53	no	down	0.00	yes	0.97	no	down	2.7E-02	yes
MSTRG.4030.12 : Eftud2 : F1LM66	4.66	yes	up	6.0E-04	yes	chr10	exon	internal exon	NA	0.63	no	down	0.32	no	1.02	no	up	4.9E-02	yes
ENSRNOT00000059921 : Serpinb6b : Q68FX2	5.33	yes	up	5.8E-08	yes	chr17	promoter	Distal	LCP	1.10	no	up	0.37	no	0.95	no	down	6.3E-06	yes
ENSRNOT00000080305 : Wls : Q6P689	0.38	yes	down	6.5E-05	yes	chr2	exon	last exon	NA	0.97	no	down	0.64	no	1.11	no	up	1.3E-07	yes
MSTRG.5482.4 : Ncor2 : A0A0G2JU91	2.16	yes	up	1.6E-06	yes	chr12	intron	internal intron	NA	0.68	no	down	0.57	no	1.01	no	up	7.1E-01	no
MSTRG.11265.2 : Ubap2l : E9PTR4	6.62	yes	up	4.0E-04	yes	chr2	intron	internal intron	NA	1.14	no	up	0.58	no	1.13	no	up	3.8E-12	yes
ENSRNOT00000082985 : Prkar2b : P12369	0.61	yes	down	1.6E-04	yes	chr6	intron	internal intron	NA	1.09	no	up	0.09	no	0.71	yes	down	2.8E-15	yes
ENSRNOT00000044805 : Dnajb12 : Q5FVC4	32.15	yes	up	1.5E-05	yes	chr20	exon	internal exon	NA	0.95	no	down	0.76	no	1.07	no	up	2.0E-04	yes
MSTRG.20284.2 : Qrich1 : F1M4M7	3.68	yes	up	3.4E-07	yes	chr8	exon	internal exon	NA	0.64	no	down	0.45	no	1.03	no	up	2.0E-01	no
ENSRNOT00000076146 : Becn1 : Q91XJ1	0.15	yes	down	1.2E-04	yes	chr10	exon	first exon	NA	0.85	no	down	0.31	no	0.97	no	down	9.2E-02	no
ENSRNOT00000008788 : Tbc1d10a : Q587K3	inf	yes	up	1.7E-04	yes	chr14	intron	first intron	NA	0.96	no	down	0.47	no	0.99	no	down	5.8E-01	no
ENSRNOT00000064705 : Dock9 : F1LSM8	0.10	yes	down	2.7E-05	yes	chr15	intron	internal intron	NA	0.97	no	down	0.91	no	0.97	no	down	2.3E-04	yes
ENSRNOT00000091789 : Myo5a : A0A0G2K9S4	1.92	yes	up	1.3E-04	yes	chr8	exon	internal exon	NA	1.20	no	up	0.62	no	1.12	no	up	2.4E-10	yes
ENSRNOT00000045628 : Tf : A0A0G2QC06	5.10	yes	up	5.5E-06	yes	chr8	intron	first intron	NA	1.34	no	up	0.46	no	1.08	no	up	1.0E-13	yes
MSTRG.4443.1 : Ltn1 : F1M9Q3	0.38	yes	down	4.2E-04	yes	chr11	intron	internal intron	NA	0.66	no	down	0.48	no	1.08	no	up	1.4E-11	yes
ENSRNOT00000084268 : Picalm : Q66WT9	8.74	yes	up	2.1E-13	yes	chr1	intron	internal intron	NA	0.50	yes	down	0.04	yes	1.45	yes	up	1.6E-10	yes
MSTRG.11263.1 : Atp8b2 : D4A509	0.14	yes	down	3.1E-04	yes	chr2	exon	first exon	NA	1.59	no	up	0.62	no	0.92	no	down	5.0E-03	yes
MSTRG.9846.3 : Pkn1 : Q63433	1.53	yes	up	6.1E-04	yes	chr19	intron	internal intron	NA	1.44	no	up	0.40	no	1.01	no	up	5.4E-01	no
ENSRNOT00000089229 : Golim4 : Q5BJK8	0.34	yes	down	3.2E-04	yes	chr2	intron	internal intron	NA	1.07	no	up	0.67	no	1.06	no	up	1.6E-03	yes
MSTRG.7229.4 : Usp54 : Q6IE24	0.36	yes	down	1.9E-04	yes	chr15	intron	internal intron	NA	1.25	no	up	0.49	no	0.83	yes	down	7.0E-09	yes
ENSRNOT00000084274 : Prom1 : Q9JI49	8.32	yes	up	2.4E-09	yes	chr14	intron	internal intron	NA	0.76	no	down	0.05	yes	0.87	no	down	1.5E-05	yes
MSTRG.1201.6 : Ntrk3 : Q68G04	7.03	yes	up	1.5E-06	yes	chr1	intron	internal intron	NA	1.65	no	up	0.43	no	0.87	no	down	1.8E-07	yes
ENSRNOT00000090060 : Cwc27 : A0A0G2JXR7	2.71	yes	up	6.8E-05	yes	chr2	intron	internal intron	NA	0.96	no	down	0.29	no	1.19	no	up	1.4E-06	yes
ENSRNOT00000085735 : Ywhah : P68511	0.09	yes	down	3.0E-04	yes	chr14	intron	last intron	NA	0.98	no	down	0.80	no	1.00	no	down	5.7E-01	no
ENSRNOT00000018464 : Tinagl1 : Q4V8N0	0.10	yes	down	7.3E-25	yes	chr5	promoter	Distal	LCP	0.57	no	down	0.12	no	0.89	no	down	4.1E-08	yes
ENSRNOT00000086660 : Cand2 : G3V7E8	0.39	yes	down	2.9E-04	yes	chr4	exon	internal exon	NA	1.01	no	up	0.87	no	0.97	no	down	1.3E-05	yes
ENSRNOT00000045165 : Ntrk3 : Q68G04	7.03	yes	up	1.5E-06	yes	chr1	intron	internal intron	NA	1.22	no	up	0.06	no	0.87	no	down	1.8E-07	yes
MSTRG.20089.1 : Morf4l1 : Q6AYU1	0.22	yes	down	4.4E-05	yes	chr8	intron	internal intron	NA	4.45	yes	up	0.04	yes	1.07	no	up	1.5E-07	yes
ENSRNOT00000019304 : Arhgap17 : D4AAV2	0.83	yes	down	2.6E-07	yes	chr1	intron	internal intron	NA	1.20	no	up	0.49	no	1.01	no	up	3.1E-01	no
MSTRG.21682.2 : Yipf6 : A0A096MJG6	inf	yes	up	3.8E-06	yes	chrX	intron	first intron	NA	1.97	no	up	0.09	no	1.03	no	up	1.9E-01	no
MSTRG.13130.4 : Calcrl : Q63118	2.81	yes	up	1.2E-04	yes	chr3	intron	internal intron	NA	3.78	yes	up	0.17	no	1.09	no	up	1.2E-04	yes
MSTRG.8278.34 : Rbpms : F2Z3S5	0.02	yes	down	2.6E-05	yes	chr16	promoter	Proximal	ICP	1.21	no	up	0.14	no	1.06	no	up	4.9E-05	yes
MSTRG.3713.1 : Cltc : F1M779	0.27	yes	down	1.4E-04	yes	chr10	intron	internal intron	NA	0.55	no	down	0.50	no	1.04	no	up	1.3E-11	yes
MSTRG.3280.2 : Arhgap44 : A0A0H2UHC0	7.94	yes	up	4.2E-19	yes	chr10	intron	internal intron	NA	0.63	no	down	0.09	no	1.04	no	up	6.6E-02	no
ENSRNOT00000092475 : Tsr1 : D3ZEM8	45.59	yes	up	3.4E-04	yes	chr10	intron	internal intron	NA	1.23	no	up	0.12	no	1.00	no	down	9.7E-01	no
MSTRG.13195.9 : Arhgap1 : D4A6C5	3.73	yes	up	1.8E-11	yes	chr3	intron	internal intron	NA	0.65	no	down	0.47	no	1.04	no	up	1.7E-04	yes
ENSRNOT00000032780 : Eln : Q99372	3.55	yes	up	3.0E-04	yes	chr12	intron	internal intron	NA	0.89	no	down	0.81	no	0.82	yes	down	3.9E-08	yes
MSTRG.16640.1 : Arhgef10l : M0R7W2	2.87	yes	up	9.9E-07	yes	chr5	intron	internal intron	NA	1.03	no	up	0.67	no	1.13	no	up	8.4E-04	yes
MSTRG.9728.6 : Cnot1 : G3V7M0	0.08	yes	down	2.6E-04	yes	chr19	intron	internal intron	NA	1.17	no	up	0.68	no	1.01	no	up	1.2E-01	no
MSTRG.1683.13 : Il4r : Q63257	9.80	yes	up	2.9E-08	yes	chr1	intron	internal intron	NA	1.00	no	down	0.64	no	1.11	no	up	2.1E-05	yes
MSTRG.11797.1 : Cryz : Q6AYT0	3.72	yes	up	1.8E-04	yes	chr2	intron	first intron	NA	0.86	no	down	0.71	no	0.91	no	down	9.1E-09	yes
ENSRNOT00000046181 : Rnaseh2a : Q5U209	14.16	yes	up	1.1E-05	yes	chr19	intron	last intron	NA	1.10	no	up	0.70	no	1.08	no	up	2.6E-05	yes
MSTRG.17241.2 : Prkar2b : P12369	0.61	yes	down	1.6E-04	yes	chr6	intron	internal intron	NA	1.81	no	up	0.02	yes	0.71	yes	down	2.8E-15	yes
ENSRNOT00000090538 : Med12l : A0A0G2JV69	6.48	yes	up	1.3E-08	yes	chr2	promoter	Distal	LCP	1.01	no	up	0.62	no	0.61	yes	down	2.2E-04	yes
MSTRG.9598.4 : Cep192 : D4A3X0	4.22	yes	up	1.3E-12	yes	chr18	intron	internal intron	NA	1.45	no	up	0.32	no	0.95	no	down	9.1E-04	yes
MSTRG.9846.1 : Pkn1 : Q63433	1.53	yes	up	6.1E-04	yes	chr19	intron	internal intron	NA	1.23	no	up	0.40	no	1.01	no	up	5.4E-01	no
MSTRG.20817.12 : Map4k4 : A0A0G2K7W4	0.36	yes	down	7.1E-05	yes	chr9	intron	internal intron	NA	0.97	no	down	0.93	no	1.10	no	up	7.1E-06	yes
MSTRG.7470.15 : Acin1 : E9PST5	0.30	yes	down	1.1E-04	yes	chr15	promoter	Distal	LCP	0.71	no	down	0.33	no	0.98	no	down	5.1E-04	yes
MSTRG.162.1 : Tpd52l1 : Q499Q2	2.69	yes	up	6.5E-28	yes	chr1	promoter	Distal	LCP	0.38	yes	down	0.01	yes	0.97	no	down	5.0E-01	no
MSTRG.479.4 : Epn1 : O88339	2.09	yes	up	1.9E-04	yes	chr1	intron	internal intron	NA	0.87	no	down	0.69	no	0.99	no	down	3.7E-01	no
MSTRG.19956.1 : Myo1e : A0A0G2K9E8	3.15	yes	up	9.4E-05	yes	chr8	intron	first intron	NA	1.29	no	up	0.13	no	0.76	yes	down	2.6E-20	yes
MSTRG.2881.4 : Pkd1 : Q9ERV0	inf	yes	up	6.6E-05	yes	chr10	intron	internal intron	NA	0.76	no	down	0.47	no	1.19	no	up	9.8E-07	yes
ENSRNOT00000066447 : Kif13a : D3ZM20	0	yes	down	7.8E-05	yes	chr17	intron	internal intron	NA	1.13	no	up	0.66	no	1.00	no	up	9.5E-01	no
MSTRG.8278.41 : Rbpms : F2Z3S5	0.02	yes	down	2.6E-05	yes	chr16	promoter	Proximal	ICP	0.38	yes	down	0.05	yes	1.06	no	up	4.9E-05	yes
ENSRNOT00000085801 : Zeb2 : A0A0G2K8T6	0.34	yes	down	8.0E-09	yes	chr3	intron	internal intron	NA	1.37	no	up	0.34	no	1.01	no	up	2.4E-01	no
ENSRNOT00000054862 : Nap1l4 : Q5U2Z3	0.36	yes	down	7.2E-05	yes	chr1	intron	internal intron	NA	0.96	no	down	0.22	no	0.98	no	down	3.1E-02	yes
ENSRNOT00000001564 : Ndufv3 : Q6PCU8	0	yes	down	1.9E-04	yes	chr20	intron	first intron	NA	1.04	no	up	0.75	no	0.93	no	down	3.2E-03	yes
MSTRG.21008.1 : Myl1 : P02600	2.29	yes	up	4.3E-05	yes	chr9	promoter	Distal	LCP	1.11	no	up	0.07	no	1.33	yes	up	6.1E-13	yes
ENSRNOT00000040255 : Itpr1 : A0A0A0MY31	3.16	yes	up	1.1E-05	yes	chr4	intron	internal intron	NA	1.00	no	down	0.50	no	0.87	no	down	1.1E-14	yes
MSTRG.9435.7 : Tcerg1 : B5DEZ4	0.26	yes	down	2.8E-04	yes	chr18	promoter	Intermediate	ICP	1.53	no	up	0.00	yes	1.02	no	up	4.4E-02	yes
MSTRG.5778.2 : Pign : E9PTA5	0.23	yes	down	2.2E-04	yes	chr13	intron	internal intron	NA	1.00	no	down	0.75	no	1.05	no	up	3.6E-03	yes
MSTRG.185.3 : Pdcd6 : G3V7W1	15.41	yes	up	1.1E-04	yes	chr1	promoter	Distal	LCP	1.31	no	up	0.20	no	0.95	no	down	4.9E-04	yes
MSTRG.1683.1 : Il4r : Q63257	9.80	yes	up	2.9E-08	yes	chr1	intron	internal intron	NA	0.29	yes	down	0.40	no	1.11	no	up	2.1E-05	yes
MSTRG.5482.14 : Ncor2 : A0A0G2JU91	2.16	yes	up	1.6E-06	yes	chr12	intron	internal intron	NA	1.31	no	up	0.51	no	1.01	no	up	7.1E-01	no
MSTRG.9889.1 : Nf1x : F2Z3R4	0.58	yes	down	1.9E-06	yes	chr19	intron	internal intron	NA	0.96	no	down	0.82	no	0.77	yes	down	2.4E-14	yes
MSTRG.21170.1 : Gigyf2 : A0A096MJI4	0.59	yes	down	1.9E-05	yes	chr9	intron	internal intron	NA	0.88	no	down	0.38	no	1.31	yes	up	2.3E-10	yes
ENSRNOT00000090643 : Opcml : F1M2I5	2.41	yes	up	1.6E-04	yes	chr8	intron	last intron	NA	0.99	no	down	0.63	no	0.91	no	down	2.1E-15	yes
MSTRG.5467.4 : Stx2 : Q7TS57	4.45	yes	up	6.0E-04	yes	chr12	exon	last exon	NA	0.61	no	down	0.10	no	0.91	no	down	2.6E-04	yes
ENSRNOT00000076742 : Dync1li2 : Q5D023	0.17	yes	down	2.9E-04	yes	chr19	intron	last intron	NA	0.96	no	down	0.83	no	0.99	no	down	1.1E-01	no
ENSRNOT00000085894 : Tpm1 : Q923Z2	9.41	yes	up	5.1E-14	yes	chr8	intron	internal intron	NA	0.26	yes	down	0.16	no	0.75	yes	down	6.0E-18	yes
ENSRNOT00000004941 : Lrrc59 : Q5RJR8	4.07	yes	up	4.6E-04	yes	chr10	exon	internal exon	NA	1.08	no	up	0.73	no	1.13	no	up	5.6E-13	yes
MSTRG.9561.1 : Wdr7 : Q9ERH3	0.16	yes	down	4.0E-04	yes	chr18	promoter	Distal	LCP	1.33	no	up	0.29	no	0.99	no	down	6.5E-01	no
ENSRNOT00000084303 : Hist1h2bd : D3ZWM5	0.90	no	down	5.8E-05	yes	chr17	promoter	Distal	LCP	0.96	no	down	0.45	no	0.74	yes	down	5.5E-11	yes
ENSRNOT00000036195 : Pak1ip1 : Q32PZ0	0	yes	down	6.0E-07	yes	chr17	exon	last exon	NA	1.06	no	up	0.78	no	1.04	no	up	7.5E-02	no
MSTRG.6191.1 : Ddr2 : B1WC09	0.49	yes	down	3.3E-05	yes	chr13	exon	internal exon	NA	0.36	yes	down	0.39	no	0.95	no	down	3.2E-01	no
MSTRG.3441.3 : Ankfy1 : D4A1J6	0.33	yes	down	7.9E-05	yes	chr10	intron	internal intron	NA	1.01	no	up	0.99	no	0.97	no	down	1.1E-04	yes
MSTRG.4699.2 : Kpna1 : P83953	32	yes	up	7.2E-05	yes	chr11	promoter	Distal	LCP	1.52	no	up	0.58	no	1.11	no	up	5.7E-08	yes
ENSRNOT00000006131 : Atg2b : Q5EBA2	0.42	yes	down	4.5E-04	yes	chr6	exon	internal exon	NA	0.55	no	down	0.16	no	1.03	no	up	1.2E-01	no
ENSRNOT00000078705 : Prkcb : A0A0G2K5Q0	3.82	yes	up	4.6E-04	yes	chr1	intron	internal intron	NA	1.14	no	up	0.26	no	1.10	no	up	1.7E-07	yes
ENSRNOT00000088170 : Calcrl : Q63118	2.81	yes	up	1.2E-04	yes	chr3	intron	internal intron	NA	1.00	no	down	0.45	no	1.09	no	up	1.2E-04	yes
ENSRNOT00000001334 : Ncor2 : A0A0G2JU91	2.16	yes	up	1.6E-06	yes	chr12	intron	internal intron	NA	0.94	no	down	0.26	no	1.01	no	up	7.1E-01	no
MSTRG.11953.63 : RT1-CE7 : D3ZLE6	inf	yes	up	7.3E-07	yes	chr20	exon	internal exon	NA	1.46	no	up	0.69	no	1.22	yes	up	7.3E-16	yes
MSTRG.15231.7 : Tspan9 : D4AAV9	0.41	yes	down	1.4E-09	yes	chr4	exon	last exon	NA	1.11	no	up	0.80	no	0.96	no	down	2.4E-05	yes
MSTRG.19107.1 : Fbln1 : B1WC21	0.23	yes	down	7.2E-09	yes	chr7	intron	internal intron	NA	2.17	yes	up	0.17	no	1.15	no	up	5.3E-06	yes
ENSRNOT00000089108 : Dlg4 : P31016	2.15	yes	up	8.6E-07	yes	chr10	intron	last intron	NA	0.99	no	down	0.72	no	0.97	no	down	9.1E-02	no
ENSRNOT00000057641 : Tpm1 : A0A0G2JSQ4	9.41	yes	up	5.1E-14	yes	chr8	intron	internal intron	NA	0.59	no	down	0.35	no	0.81	yes	down	2.5E-10	yes
ENSRNOT00000093097 : Tie1 : B5DFD6	1.35	yes	up	2.1E-04	yes	chr5	intron	first intron	NA	0.45	yes	down	0.23	no	0.76	yes	down	4.3E-08	yes
MSTRG.5035.1 : Arhgef18 : F1LT94	0.62	yes	down	4.6E-05	yes	chr12	exon	internal exon	NA	0.70	no	down	0.10	no	0.93	no	down	5.9E-05	yes
MSTRG.16268.1 : Tie1 : B5DFD6	1.35	yes	up	2.1E-04	yes	chr5	intron	first intron	NA	0.39	yes	down	0.38	no	0.76	yes	down	4.3E-08	yes
MSTRG.6624.2 : Ankrd17 : D4A0B4	3.39	yes	up	8.9E-05	yes	chr14	intron	internal intron	NA	0.46	yes	down	0.40	no	1.11	no	up	1.6E-06	yes
MSTRG.18056.2 : Smarcc2 : D4A510	6.20	yes	up	2.1E-04	yes	chr7	intron	last intron	NA	1.03	no	up	0.70	no	0.96	no	down	6.4E-05	yes
MSTRG.14995.10 : Itpr1 : A0A0A0MY31	3.16	yes	up	1.1E-05	yes	chr4	intron	internal intron	NA	1.24	no	up	0.08	no	0.87	no	down	1.1E-14	yes
ENSRNOT00000037050 : Rsf1 : D3ZGQ8	2.47	yes	up	3.7E-29	yes	chr1	exon	last exon	NA	0.99	no	down	0.50	no	0.95	no	down	6.9E-05	yes
MSTRG.5723.2 : Ttc28 : D3ZXP1	4.41	yes	up	1.9E-04	yes	chr12	promoter	Intermediate	LCP	0.42	yes	down	0.07	no	0.86	no	down	6.9E-05	yes
ENSRNOT00000026734 : Tm9sf1 : Q66HF2	5.15	yes	up	5.3E-11	yes	chr15	intron	last intron	NA	1.12	no	up	0.37	no	1.02	no	up	4.7E-02	yes
ENSRNOT00000082474 : Ptgis : Q62969	0.55	yes	down	6.0E-05	yes	chr3	intron	internal intron	NA	0.71	no	down	0.37	no	0.99	no	down	7.4E-02	no

ENSRNOT00000016220 : Mapre1 : Q66HR2	5.59	yes	up	4.6E-04	yes	chr3	intron	internal intron	NA	1.16	no	up	0.61	no	1.03	no	up	2.1E-03	yes
ENSRNOT00000055127 : Znfx1 : F1LMA9	20.79	yes	up	3.0E-04	yes	chr3	intron	internal intron	NA	0.83	no	down	0.75	no	0.92	no	down	1.0E-03	yes
ENSRNOT00000013569 : Bpgm : Q6P6G4	0.17	yes	down	4.7E-04	yes	chr4	intron	first intron	NA	1.69	no	up	0.00	yes	0.88	no	down	2.8E-09	yes
MSTRG.8692.1 : Pak1ip1 : Q32PZ0	0	yes	down	6.0E-07	yes	chr17	exon	last exon	NA	1.17	no	up	0.63	no	1.04	no	up	7.5E-02	no
ENSRNOT00000089723 : Mef2a : A0A0G2JSZ4	0.04	yes	down	2.4E-04	yes	chr1	exon	last exon	NA	1.00	no	down	0.92	no	0.78	yes	down	4.1E-07	yes
ENSRNOT00000055194 : Dusp3 : B5DFF7	0.16	yes	down	1.6E-06	yes	chr10	exon	last exon	NA	0.84	no	down	0.87	no	0.90	no	down	6.2E-11	yes
ENSRNOT00000018005 : Psmb5 : G3V7Q6	2.13	yes	up	3.6E-06	yes	chr15	exon	last exon	NA	0.86	no	down	0.49	no	0.99	no	down	3.7E-01	no
MSTRG.3447.3 : Atp2a3 : G3V9U7	inf	yes	up	2.1E-05	yes	chr10	intron	internal intron	NA	0.99	no	down	0.40	no	0.77	yes	down	1.7E-16	yes
ENSRNOT00000081195 : Smarcd1 : D3ZBS9	0.24	yes	down	1.4E-04	yes	chr7	intron	internal intron	NA	1.00	no	down	1.00	no	0.95	no	down	2.9E-07	yes
MSTRG.13870.3 : Plcg1 : G3V845	0.24	yes	down	1.1E-04	yes	chr3	intron	first intron	NA	0.71	no	down	0.35	no	0.94	no	down	1.8E-09	yes
ENSRNOT00000048848 : Rbms2 : Q4QR81	0.04	yes	down	2.6E-23	yes	chr7	exon	last exon	NA	0.43	yes	down	0.48	no	1.14	no	up	2.7E-10	yes
ENSRNOT00000088945 : Plec : Q6S395	0.33	yes	down	4.2E-06	yes	chr7	exon	last exon	NA	1.11	no	up	0.50	no	1.03	no	up	2.2E-01	no
MSTRG.17715.2 : Syne3 : D3ZD24	0.24	yes	down	2.1E-14	yes	chr6	intron	internal intron	NA	1.19	no	up	0.79	no	1.11	no	up	3.8E-04	yes
MSTRG.15665.2 : Aco1 : G3V6S2	4.49	yes	up	2.7E-06	yes	chr5	intron	internal intron	NA	0.74	no	down	0.64	no	0.93	no	down	1.1E-11	yes
MSTRG.7804.5 : Mycbp2 : A0A1W2Q6I3	0.31	yes	down	9.2E-06	yes	chr15	intron	internal intron	NA	1.10	no	up	0.47	no	1.00	no	up	8.8E-01	no
MSTRG.6515.2 : Spp1 : P08721	29.54	yes	up	1.8E-44	yes	chr14	exon	last exon	NA	4.80	yes	up	0.11	no	1.32	yes	up	5.2E-14	yes
ENSRNOT00000060111 : Cfh : F1M983	0.20	yes	down	2.4E-04	yes	chr13	intron	internal intron	NA	2.07	yes	up	0.04	yes	1.20	no	up	2.7E-15	yes
MSTRG.3296.5 : Gas7 : M0R4R4	4.60	yes	up	4.0E-11	yes	chr10	intron	first intron	NA	7.06	yes	up	0.03	yes	1.22	yes	up	2.5E-15	yes
MSTRG.6812.2 : Pcdh7 : Q68HB8	0	yes	down	4.2E-04	yes	chr14	exon	last exon	NA	0.89	no	down	0.71	no	0.94	no	down	1.0E-07	yes
ENSRNOT00000013392 : Slc33a1 : Q6AYY8	0.10	yes	down	8.7E-08	yes	chr2	exon	last exon	NA	0.76	no	down	0.19	no	1.05	no	up	1.4E-05	yes
MSTRG.16757.2 : Ube4b : F1M8V2	0.02	yes	down	2.8E-12	yes	chr5	exon	last exon	NA	1.08	no	up	0.54	no	1.06	no	up	1.6E-06	yes
MSTRG.21039.9 : Tns1 : F1LN42	0.09	yes	down	2.0E-04	yes	chr9	intron	last intron	NA	0.70	no	down	0.83	no	0.86	no	down	7.8E-19	yes
MSTRG.4335.4 : Pcyt2 : O88637	4.82	yes	up	3.0E-05	yes	chr10	intron	last intron	NA	1.57	no	up	0.01	yes	0.94	no	down	1.8E-03	yes
MSTRG.20817.1 : Map4k4 : A0A0G2K7W4	0.36	yes	down	7.1E-05	yes	chr9	intron	internal intron	NA	1.31	no	up	0.50	no	1.10	no	up	7.1E-06	yes
ENSRNOT00000092413 : Shank3 : A0A0U1RS13	3.23	yes	up	4.3E-05	yes	chr7	exon	first exon	NA	1.19	no	up	0.62	no	0.98	no	down	1.0E-02	yes
MSTRG.12626.1 : Col5a1 : G3V763	0.33	yes	down	4.5E-04	yes	chr3	intron	internal intron	NA	1.00	no	down	1.00	no	1.08	no	up	2.2E-06	yes
ENSRNOT00000038589 : Iqgap3 : D3ZCS4	6.61	yes	up	2.3E-04	yes	chr2	intron	internal intron	NA	1.09	no	up	0.67	no	1.06	no	up	2.0E-01	no
MSTRG.16771.5 : H6pd : D4A7D7	0.22	yes	down	2.6E-06	yes	chr5	intron	last intron	NA	1.24	no	up	0.59	no	1.14	no	up	9.6E-15	yes
ENSRNOT00000056600 : Cops9 : B5DFN0	inf	yes	up	5.8E-06	yes	chr9	promoter	Distal	LCP	0.70	no	down	0.40	no	0.77	yes	down	2.8E-02	yes
ENSRNOT00000092945 : Picalm : Q66WT9	8.74	yes	up	2.1E-13	yes	chr1	intron	internal intron	NA	0.63	no	down	0.04	yes	1.45	yes	up	1.6E-10	yes
MSTRG.18880.4 : Plec : F7F9U6	0.33	yes	down	4.2E-06	yes	chr7	exon	last exon	NA	1.31	no	up	0.08	no	0.90	no	down	2.5E-03	yes
ENSRNOT00000021309 : Agfg1 : F1M9N7	3.59	yes	up	1.8E-08	yes	chr9	exon	internal exon	NA	0.81	no	down	0.70	no	1.16	no	up	6.0E-06	yes
ENSRNOT00000074286 : Mxra7 : F1M1U0	inf	yes	up	4.6E-04	yes	chr10	intron	internal intron	NA	1.03	no	up	0.89	no	1.16	no	up	7.7E-10	yes
MSTRG.19503.2 : Anln : M0RDG0	0.78	yes	down	4.3E-04	yes	chr8	intron	internal intron	NA	1.14	no	up	0.90	no	0.97	no	down	9.1E-01	no
ENSRNOT00000003052 : Scarb2 : P27615	6.44	yes	up	3.7E-05	yes	chr14	intron	internal intron	NA	1.11	no	up	0.67	no	0.99	no	down	3.4E-01	no
MSTRG.18056.3 : Smarcc2 : D4A510	6.20	yes	up	2.1E-04	yes	chr7	intron	last intron	NA	0.65	no	down	0.37	no	0.96	no	down	6.4E-05	yes
MSTRG.3296.1 : Gas7 : M0R4R4	4.60	yes	up	4.0E-11	yes	chr10	intron	first intron	NA	1.02	no	up	0.95	no	1.22	yes	up	2.5E-15	yes
MSTRG.7057.2 : Myo1g : A0A0G2K6E3	0.66	yes	down	3.2E-12	yes	chr14	intron	internal intron	NA	1.51	no	up	0.35	no	1.04	no	up	3.4E-02	yes
MSTRG.17007.2 : Fam98a : Q5FWT1	0.57	yes	down	6.5E-05	yes	chr6	exon	last exon	NA	0.80	no	down	0.50	no	0.98	no	down	9.6E-02	no
MSTRG.2348.2 : Smc5 : D4A9F0	4.35	yes	up	2.4E-09	yes	chr1	promoter	Distal	LCP	1.15	no	up	0.18	no	0.86	no	down	3.1E-03	yes
MSTRG.3781.5 : Luc7l3 : D3ZFB2	0.31	yes	down	4.3E-04	yes	chr10	intron	internal intron	NA	1.15	no	up	0.22	no	0.98	no	down	9.6E-04	yes
MSTRG.256.5 : Syne1 : Q8VHJ9	0.04	yes	down	1.5E-19	yes	chr1	exon	first exon	NA	1.47	no	up	0.68	no	0.89	no	down	8.9E-14	yes
ENSRNOT00000025096 : Naa35 : Q6DKG0	inf	yes	up	1.7E-04	yes	chr17	promoter	Proximal	LCP	0.73	no	down	0.18	no	1.04	no	up	3.3E-02	yes
MSTRG.8325.6 : Hook3 : Q7TQ77	0.22	yes	down	2.1E-04	yes	chr16	intron	internal intron	NA	0.73	no	down	0.45	no	1.01	no	up	5.0E-03	yes
MSTRG.20817.6 : Map4k4 : A0A0G2K7W4	0.36	yes	down	7.1E-05	yes	chr9	intron	internal intron	NA	0.83	no	down	0.18	no	1.10	no	up	7.1E-06	yes
MSTRG.7500.5 : Tm9sf1 : Q66HF2	5.15	yes	up	5.3E-11	yes	chr15	intron	last intron	NA	1.07	no	up	0.56	no	1.02	no	up	4.7E-02	yes
MSTRG.12973.12 : Rbms1 : A0A0G2K4R7	0	yes	down	3.3E-05	yes	chr3	intron	internal intron	NA	1.20	no	up	0.41	no	0.97	no	down	1.2E-01	no
MSTRG.3421.4 : Nup88 : O08658	0.25	yes	down	1.2E-04	yes	chr10	intron	internal intron	NA	1.72	no	up	0.16	no	0.95	no	down	3.4E-05	yes
ENSRNOT00000021153 : Slc2a13 : Q921A2	0.21	yes	down	3.6E-06	yes	chr7	exon	last exon	NA	1.22	no	up	0.07	no	0.92	no	down	4.1E-06	yes
MSTRG.6248.4 : Ncstn : Q8CGU6	0.30	yes	down	6.8E-05	yes	chr13	intron	first intron	NA	1.11	no	up	0.68	no	1.02	no	up	4.0E-02	yes
ENSRNOT00000092932 : Sema4a : A0A1B0GWV9	3.09	yes	up	1.9E-04	yes	chr2	promoter	Intermediate	LCP	1.00	no	down	0.80	no	1.07	no	up	2.4E-05	yes
MSTRG.6812.4 : Pcdh7 : Q68HB8	0	yes	down	4.2E-04	yes	chr14	exon	last exon	NA	1.00	no	down	0.99	no	0.94	no	down	1.0E-07	yes
ENSRNOT00000076121 : Smndc1 : Q4QQU6	inf	yes	up	1.3E-04	yes	chr1	promoter	Distal	LCP	0.81	no	down	0.56	no	1.06	no	up	2.2E-04	yes
MSTRG.123.1 : Epb41l2 : D3ZM69	0.25	yes	down	1.1E-12	yes	chr1	intron	internal intron	NA	0.72	no	down	0.09	no	0.91	no	down	8.9E-11	yes
ENSRNOT00000084540 : Kpna1 : P83953	32	yes	up	7.2E-05	yes	chr11	promoter	Distal	LCP	1.30	no	up	0.37	no	1.11	no	up	5.7E-08	yes
MSTRG.6983.1 : Morc2 : D4A2C4	6.36	yes	up	1.5E-04	yes	chr14	promoter	Proximal	HCP	0.85	no	down	0.48	no	0.97	no	down	3.2E-01	no
MSTRG.11285.6 : Npr1 : P18910	0.37	yes	down	1.2E-04	yes	chr2	intron	internal intron	NA	1.41	no	up	0.38	no	1.02	no	up	1.6E-02	yes
MSTRG.18661.1 : Ywhaz : P63102	3.68	yes	up	2.7E-04	yes	chr7	intron	last intron	NA	2.06	yes	up	0.60	no	0.87	no	down	1.2E-13	yes
ENSRNOT00000084268 : Picalm : Q66SY1	8.74	yes	up	2.1E-13	yes	chr1	intron	internal intron	NA	0.50	yes	down	0.04	yes	1.13	no	up	1.1E-06	yes
MSTRG.20284.3 : Qrich1 : F1M4M7	3.68	yes	up	3.4E-07	yes	chr8	exon	internal exon	NA	0.59	no	down	0.54	no	1.03	no	up	2.0E-01	no
ENSRNOT00000083312 : Eci1 : P23965	6.21	yes	up	1.7E-05	yes	chr10	intron	internal intron	NA	1.00	no	down	0.50	no	0.85	no	down	2.2E-15	yes
ENSRNOT00000006961 : Itgav : F1LZX9	24.74	yes	up	1.9E-17	yes	chr3	intron	first intron	NA	1.28	no	up	0.26	no	0.92	no	down	1.7E-15	yes
MSTRG.15356.5 : Pde3a : Q62865	0.30	yes	down	3.1E-07	yes	chr4	intron	internal intron	NA	0.75	no	down	0.40	no	1.00	no	up	9.7E-01	no
MSTRG.14960.2 : Eogt : Q5NDL0	6.71	yes	up	1.7E-04	yes	chr4	intron	internal intron	NA	1.06	no	up	0.89	no	1.10	no	up	2.0E-05	yes
MSTRG.20878.7 : Gls : A0A0G2KAN7	0.05	yes	down	1.5E-13	yes	chr9	intron	first intron	NA	1.00	no	down	0.99	no	1.09	no	up	8.3E-03	yes
ENSRNOT00000091230 : Exoc1 : Q4V8H2	inf	yes	up	6.1E-05	yes	chr14	intron	first intron	NA	0.83	no	down	0.23	no	0.91	no	down	3.2E-04	yes
MSTRG.19777.10 : Sin3a : A0A0G2K3H5	0.53	yes	down	4.1E-06	yes	chr8	intron	last intron	NA	2.55	yes	up	0.09	no	0.96	no	down	6.2E-05	yes
MSTRG.4675.1 : Gsk3b : A0A0G2JSH4	2.36	yes	up	1.7E-04	yes	chr11	intron	internal intron	NA	1.03	no	up	0.97	no	0.98	no	down	1.1E-02	yes
ENSRNOT00000036156 : Gbf1 : A0A0G2K3N1	0.28	yes	down	1.1E-05	yes	chr1	intron	internal intron	NA	1.03	no	up	0.90	no	1.05	no	up	6.1E-10	yes
MSTRG.11636.2 : Larp7 : M0R7D1	0.06	yes	down	3.4E-04	yes	chr2	exon	last exon	NA	1.33	no	up	0.64	no	1.07	no	up	2.0E-06	yes
MSTRG.21168.1 : Gigyf2 : A0A096MKC0	0.59	yes	down	1.9E-05	yes	chr9	intron	internal intron	NA	1.30	no	up	0.36	no	1.01	no	up	7.0E-01	no
MSTRG.13870.9 : Plcg1 : G3V845	0.24	yes	down	1.1E-04	yes	chr3	intron	first intron	NA	1.02	no	up	0.92	no	0.94	no	down	1.8E-09	yes
MSTRG.8278.25 : Rbpms : F2Z3S5	0.02	yes	down	2.6E-05	yes	chr16	promoter	Proximal	ICP	0.93	no	down	0.77	no	1.06	no	up	4.9E-05	yes
ENSRNOT00000081248 : Papss1 : A0A0G2K0L0	15.84	yes	up	7.3E-05	yes	chr2	promoter	Intermediate	LCP	0.90	no	down	0.45	no	1.04	no	up	5.2E-04	yes
ENSRNOT00000077543 : Atl2 : F1LQ09	2.32	yes	up	2.9E-04	yes	chr6	intron	last intron	NA	1.02	no	up	0.72	no	0.95	no	down	8.9E-05	yes
MSTRG.11000.1 : Slc33a1 : Q6AYY8	0.10	yes	down	8.7E-08	yes	chr2	exon	last exon	NA	0.91	no	down	0.68	no	1.05	no	up	1.4E-05	yes
MSTRG.5417.12 : Auts2 : F1M388	0.04	yes	down	4.8E-14	yes	chr12	intron	internal intron	NA	0.99	no	down	0.99	no	1.01	no	up	5.6E-01	no
MSTRG.16327.3 : Macf1 : A0A0G2K9T4	0.36	yes	down	1.3E-05	yes	chr5	exon	last exon	NA	3.43	yes	up	0.22	no	0.97	no	down	9.0E-11	yes
ENSRNOT00000007676 : Skp1 : Q6PEC4	0.41	yes	down	2.1E-04	yes	chr10	exon	last exon	NA	1.06	no	up	0.48	no	0.93	no	down	2.8E-11	yes
MSTRG.17520.2 : Srsf5 : Q09167	0.10	yes	down	3.0E-09	yes	chr6	promoter	Proximal	ICP	1.61	no	up	0.48	no	1.03	no	up	4.2E-02	yes
ENSRNOT00000065076 : Speg : Q63638	0.17	yes	down	2.6E-06	yes	chr9	intron	internal intron	NA	0.63	no	down	0.30	no	0.88	no	down	2.3E-07	yes
ENSRNOT00000009015 : Nup88 : O08658	0.25	yes	down	1.2E-04	yes	chr10	intron	internal intron	NA	0.59	no	down	0.43	no	0.95	no	down	3.4E-05	yes
MSTRG.13267.1 : Tcp11l1 : F1M9Y7	3.13	yes	up	1.2E-06	yes	chr3	intron	last intron	NA	1.17	no	up	0.54	no	0.99	no	down	8.5E-01	no
MSTRG.5972.3 : Ptprc : P04157	2.98	yes	up	6.9E-14	yes	chr13	intron	internal intron	NA	4.20	yes	up	0.19	no	1.12	no	up	6.1E-15	yes
ENSRNOT00000083017 : Herc4 : Q5PQN1	2.70	yes	up	6.2E-07	yes	chr20	intron	internal intron	NA	1.85	no	up	0.05	yes	1.00	no	down	9.1E-01	no
ENSRNOT00000001752 : P2rx4 : P51577	2.45	yes	up	6.7E-18	yes	chr12	intron	internal intron	NA	0.99	no	down	0.95	no	1.15	no	up	7.4E-08	yes
MSTRG.14918.1 : Nup210 : P11654	0.11	yes	down	6.6E-10	yes	chr4	intron	internal intron	NA	0.71	no	down	0.50	no	1.08	no	up	1.7E-07	yes
ENSRNOT00000082596 : Dennd4c : F1LTD7	0.31	yes	down	2.0E-04	yes	chr5	intron	internal intron	NA	1.08	no	up	0.10	no	0.97	no	down	9.0E-02	no
MSTRG.18214.2 : Arhgap45 : D4AAI2	5.87	yes	up	2.5E-04	yes	chr7	exon	internal exon	NA	1.26	no	up	0.56	no	1.20	no	up	4.5E-05	yes
ENSRNOT00000049437 : Ctnna3 : B2RYN9	0	yes	down	1.3E-06	yes	chr20	promoter	Distal	LCP	0.92	no	down	0.55	no	1.03	no	up	1.7E-01	no
ENSRNOT00000033627 : Nnt : Q5BJZ3	3.00	yes	up	1.8E-04	yes	chr2	intron	internal intron	NA	1.61	no	up	0.09	no	0.96	no	down	1.2E-07	yes
MSTRG.13413.3 : Map1a : P34926	3.41	yes	up	1.8E-05	yes	chr3	intron	last intron	NA	1.64	no	up	0.44	no	1.03	no	up	8.9E-11	yes
ENSRNOT00000024347 : Atp2a2 : P11507	0.21	yes	down	5.6E-04	yes	chr12	intron	internal intron	NA	1.03	no	up	0.87	no	0.89	no	down	1.5E-13	yes
MSTRG.2366.1 : Pgm5 : D3ZVR9	2.44	yes	up	4.5E-04	yes	chr1	intron	last intron	NA	0.37	yes	down	0.41	no	1.01	no	up	3.2E-01	no
ENSRNOT00000077597 : Cln5 : A0A1W2Q672	0.33	yes	down	1.1E-04	yes	chr15	intron	first intron	NA	1.17	no	up	0.29	no	1.04	no	up	3.7E-01	no
MSTRG.7082.1 : Grb10 : D3ZEA3	0.35	yes	down	3.4E-07	yes	chr14	intron	internal intron	NA	1.10	no	up	0.52	no	1.09	no	up	4.5E-06	yes
ENSRNOT00000078830 : Kif1b : A0A0G2KA12	3.65	yes	up	7.4E-05	yes	chr5	intron	internal intron	NA	1.01	no	up	0.40	no	1.05	no	up	6.5E-02	no
ENSRNOT00000083193 : Tifa : Q5XIB9	0.42	yes	down	9.5E-05	yes	chr2	exon	internal exon	NA	0.98	no	down	0.62	no	1.16	no	up	2.8E-04	yes
ENSRNOT00000000092 : Antxr2 : Q00IM8	1.10	no	up	9.8E-05	yes	chr14	intron	internal intron	NA	1.02	no	up	0.88	no	1.01	no	up	3.8E-01	no
ENSRNOT00000064283 : Ubtf : P25977	1.48	yes	up	1.5E-06	yes	chr10	intron	internal intron	NA	1.41	no	up	0.32	no	0.96	no	down	1.4E-06	yes

MSTRG.16592.3 : Kdm1a : A0A0G2K736	0.26	yes	down	3.7E-04	yes	chr5	intron	internal intron	NA	0.59	no	down	0.39	no	1.01	no	up	5.0E-01	no
ENSRNOT00000079719 : Prkar2b : P12369	0.61	yes	down	1.6E-04	yes	chr6	intron	internal intron	NA	0.94	no	down	0.50	no	0.71	yes	down	2.8E-15	yes
MSTRG.14053.3 : Dido1 : D3ZWL9	2.83	yes	up	7.0E-07	yes	chr3	intron	internal intron	NA	0.93	no	down	0.88	no	1.01	no	up	2.0E-01	no
ENSRNOT00000068742 : Ano6 : A0A0G2K1M7	3.87	yes	up	9.8E-09	yes	chr7	intron	internal intron	NA	0.90	no	down	0.58	no	0.91	no	down	2.1E-09	yes
ENSRNOT00000002313 : Runx1 : Q63046	0.62	yes	down	5.4E-05	yes	chr11	intron	first intron	NA	0.88	no	down	0.66	no	1.47	yes	up	3.2E-10	yes
MSTRG.8278.40 : Rbpms : F2Z3S5	0.02	yes	down	2.6E-05	yes	chr16	promoter	Proximal	ICP	0.53	no	down	0.07	no	1.06	no	up	4.9E-05	yes
MSTRG.14342.4 : ST7 : A0A0G2KBB5	4.88	yes	up	1.5E-16	yes	chr4	promoter	Distal	LCP	1.20	no	up	0.30	no	1.14	no	up	1.4E-03	yes
ENSRNOT00000089624 : Plscr1 : A0A0G2K7Q1	0.33	yes	down	1.3E-05	yes	chr8	promoter	Distal	LCP	0.89	no	down	0.23	no	1.08	no	up	5.2E-06	yes
MSTRG.1026.1 : Bax : Q9JKL3	8.53	yes	up	7.6E-07	yes	chr1	promoter	Distal	LCP	0.99	no	down	0.96	no	1.07	no	up	5.8E-08	yes
ENSRNOT00000034567 : Tmx4 : G3V912	7.04	yes	up	3.9E-04	yes	chr3	promoter	Distal	LCP	0.39	yes	down	0.20	no	0.97	no	down	6.5E-02	no
ENSRNOT00000063772 : Rad50 : G3V9X6	3.28	yes	up	4.1E-06	yes	chr10	intron	internal intron	NA	1.12	no	up	0.30	no	0.95	no	down	2.3E-11	yes
MSTRG.6320.2 : Cdc42bpa : G3V6C9	8.92	yes	up	2.1E-05	yes	chr13	intron	internal intron	NA	0.82	no	down	0.46	no	0.97	no	down	2.5E-04	yes
ENSRNOT00000016558 : Ttc29 : Q6AYP3	0.28	yes	down	7.9E-05	yes	chr19	exon	internal exon	NA	0.64	no	down	0.22	no	1.55	yes	up	1.9E-10	yes
ENSRNOT00000075967 : Gigyf2 : A0A096MKC0	0.59	yes	down	1.9E-05	yes	chr9	intron	internal intron	NA	0.93	no	down	0.78	no	1.01	no	up	7.0E-01	no
ENSRNOT00000002323 : Dvl3 : D4ADV8	0.50	yes	down	1.6E-05	yes	chr11	intron	first intron	NA	1.31	no	up	0.09	no	0.98	no	down	2.0E-01	no
MSTRG.8055.1 : Lsm4 : D4A2C6	0.48	yes	down	3.2E-04	yes	chr16	exon	last exon	NA	1.72	no	up	0.06	no	0.96	no	down	2.1E-01	no
MSTRG.13558.4 : Siglec1 : A0A0G2K320	0.24	yes	down	6.2E-06	yes	chr3	promoter	Proximal	LCP	1.10	no	up	0.76	no	1.25	yes	up	7.1E-13	yes
ENSRNOT00000074058 : Rrm1 : Q5U2Q5	8.63	yes	up	2.6E-22	yes	chr1	intron	last intron	NA	1.29	no	up	0.15	no	1.04	no	up	2.0E-04	yes
ENSRNOT00000029577 : Cnn1 : Q08290	3.38	yes	up	1.8E-04	yes	chr8	intron	internal intron	NA	0.74	no	down	0.07	no	1.04	no	up	1.3E-06	yes
MSTRG.13466.5 : Myef2 : A0A0G2K402	2.63	yes	up	2.7E-04	yes	chr3	promoter	Intermediate	LCP	1.08	no	up	0.49	no	0.93	no	down	4.5E-09	yes
MSTRG.7965.1 : Bmpr1a : Q78EA7	0.29	yes	down	2.1E-04	yes	chr16	intron	internal intron	NA	4.37	yes	up	0.17	no	0.90	no	down	9.8E-10	yes
MSTRG.20839.6 : Kdelc1 : B5DFA5	5.54	yes	up	1.1E-06	yes	chr9	exon	last exon	NA	0.78	no	down	0.55	no	0.94	no	down	8.4E-02	no
MSTRG.4497.1 : Dopey2 : A0A0G2JXD9	3.39	yes	up	1.3E-40	yes	chr11	intron	internal intron	NA	0.90	no	down	0.65	no	0.98	no	down	3.1E-01	no
MSTRG.1326.1 : Picalm : A0A1B0GWW9	8.74	yes	up	2.1E-13	yes	chr1	intron	internal intron	NA	3.98	yes	up	0.01	yes	1.04	no	up	1.0E-01	no
MSTRG.4716.8 : Adcy5 : G3V9G1	0.16	yes	down	2.3E-10	yes	chr11	intron	internal intron	NA	0.81	no	down	0.35	no	1.00	no	down	7.6E-01	no
MSTRG.17689.1 : Golga5 : G3V6Z7	4.67	yes	up	4.2E-17	yes	chr6	intron	last intron	NA	1.03	no	up	0.72	no	1.07	no	up	2.3E-12	yes
ENSRNOT00000028680 : Nomo1 : D3ZSA9	4.00	yes	up	3.3E-06	yes	chr1	intron	internal intron	NA	1.09	no	up	0.47	no	1.08	no	up	1.0E-11	yes
ENSRNOT00000092542 : Mapk10 : A0A0U1RRS7	2.61	yes	up	3.6E-06	yes	chr14	intron	first intron	NA	1.00	no	down	0.40	no	0.56	yes	down	6.7E-04	yes
ENSRNOT00000086769 : Ywhah : P68511	0.09	yes	down	3.0E-04	yes	chr14	intron	last intron	NA	0.76	no	down	0.03	yes	1.00	no	down	5.7E-01	no
MSTRG.97.3 : Bclaf1 : B1WC16	0.67	yes	down	1.3E-07	yes	chr1	intron	internal intron	NA	0.81	no	down	0.35	no	1.03	no	up	6.8E-02	no
MSTRG.5439.3 : Gusb : F1LQQ8	29.04	yes	up	6.5E-05	yes	chr12	intron	internal intron	NA	1.57	no	up	0.06	no	0.97	no	down	1.8E-03	yes
MSTRG.18206.6 : Cirbp : P60825	0.73	yes	down	1.3E-04	yes	chr7	exon	first exon	NA	0.67	no	down	0.05	yes	0.85	no	down	3.4E-16	yes
ENSRNOT00000073950 : Dync1h1 : M0R9X8	0.19	yes	down	1.5E-06	yes	chr6	intron	internal intron	NA	1.01	no	up	0.48	no	0.99	no	down	3.4E-03	yes
MSTRG.9714.4 : Dync1li2 : Q5D023	0.17	yes	down	2.9E-04	yes	chr19	intron	last intron	NA	0.60	no	down	0.10	no	0.99	no	down	1.1E-01	no
ENSRNOT00000012725 : Tf : P12346	5.10	yes	up	5.5E-06	yes	chr8	intron	first intron	NA	1.07	no	up	0.37	no	1.33	yes	up	1.6E-08	yes
MSTRG.1683.2 : Il4r : Q63257	9.80	yes	up	2.9E-08	yes	chr1	intron	internal intron	NA	2.39	yes	up	0.10	no	1.11	no	up	2.1E-05	yes
MSTRG.7562.1 : Phf11b : M0RB46	3.03	yes	up	2.9E-04	yes	chr15	promoter	Intermediate	LCP	1.44	no	up	0.21	no	1.15	no	up	2.3E-06	yes
ENSRNOT00000008238 : Ppm1a : P20650	2.35	yes	up	3.9E-04	yes	chr6	intron	last intron	NA	2.96	yes	up	0.09	no	0.96	no	down	5.3E-04	yes
MSTRG.17804.1 : Cdc42bpb : A0A0G2KB58	0.39	yes	down	3.8E-04	yes	chr6	intron	internal intron	NA	0.93	no	down	0.61	no	0.97	no	down	3.6E-07	yes
MSTRG.5482.9 : Ncor2 : A0A0G2JU91	2.16	yes	up	1.6E-06	yes	chr12	intron	internal intron	NA	1.00	no	down	0.90	no	1.01	no	up	7.1E-01	no
MSTRG.21367.3 : Uba1 : Q5U300	0	yes	down	1.9E-08	yes	chrX	exon	last exon	NA	1.11	no	up	0.71	no	0.93	no	down	2.8E-14	yes
ENSRNOT00000006709 : Dock4 : M0R6K4	0.43	yes	down	2.9E-04	yes	chr6	exon	last exon	NA	1.03	no	up	0.76	no	0.94	no	down	8.4E-03	yes
ENSRNOT00000088025 : Bclaf1 : B1WC16	0.67	yes	down	1.3E-07	yes	chr1	intron	internal intron	NA	0.95	no	down	0.48	no	1.03	no	up	6.8E-02	no
ENSRNOT00000084758 : Arih1 : D3ZXL1	0.23	yes	down	8.6E-06	yes	chr8	intron	first intron	NA	1.09	no	up	0.09	no	0.93	no	down	1.4E-04	yes
MSTRG.1974.4 : Nap1l4 : Q5U2Z3	0.36	yes	down	7.2E-05	yes	chr1	intron	internal intron	NA	0.66	no	down	0.03	yes	0.98	no	down	3.1E-02	yes
ENSRNOT00000020539 : Smndc1 : Q4QQU6	inf	yes	up	1.3E-04	yes	chr1	promoter	Distal	LCP	0.90	no	down	0.77	no	1.06	no	up	2.2E-04	yes
MSTRG.14882.1 : Aplf : F6Q5G6	0.12	yes	down	8.1E-06	yes	chr4	exon	last exon	NA	0.95	no	down	0.88	no	0.99	no	down	2.8E-01	no
ENSRNOT00000022126 : Acsl5 : O88813	6.59	yes	up	1.3E-04	yes	chr1	intron	last intron	NA	1.49	no	up	0.06	no	1.08	no	up	2.2E-08	yes
ENSRNOT00000010676 : Rcor1 : F1LXS5	2.75	yes	up	3.6E-04	yes	chr6	intron	internal intron	NA	1.33	no	up	0.65	no	1.11	no	up	2.6E-06	yes
MSTRG.8325.1 : Hook3 : Q7TQ77	0.22	yes	down	2.1E-04	yes	chr16	intron	internal intron	NA	1.28	no	up	0.33	no	1.01	no	up	5.0E-03	yes
ENSRNOT00000081021 : Plec : F7F9U6	0.33	yes	down	4.2E-06	yes	chr7	exon	last exon	NA	0.97	no	down	0.44	no	0.90	no	down	2.5E-03	yes
ENSRNOT00000065028 : Dnm2 : A0A0A0MY48	0.11	yes	down	8.9E-11	yes	chr8	intron	first intron	NA	1.07	no	up	0.46	no	1.05	no	up	2.0E-08	yes
ENSRNOT00000014849 : Rpl29 : P25886	3.86	yes	up	3.0E-04	yes	chr8	intron	first intron	NA	0.83	no	down	0.84	no	0.98	no	down	6.0E-01	no
ENSRNOT00000064212 : Yeats2 : D3ZBV9	4.61	yes	up	5.1E-05	yes	chr11	intron	internal intron	NA	1.53	no	up	0.02	yes	1.11	no	up	2.4E-05	yes
MSTRG.19963.7 : Sltm : A0A0G2K904	0.34	yes	down	6.4E-06	yes	chr8	intron	internal intron	NA	0.88	no	down	0.09	no	0.94	no	down	2.8E-02	yes
MSTRG.4992.1 : Top3b : D4A9Z2	11.41	yes	up	1.2E-04	yes	chr11	intron	last intron	NA	0.67	no	down	0.54	no	0.99	no	down	6.1E-01	no
ENSRNOT00000086441 : LOC108348175 : A0A0G2	0.05	yes	down	4.0E-19	yes	chr1	promoter	Intermediate	LCP	0.41	yes	down	0.02	yes	1.01	no	up	4.0E-01	no
ENSRNOT00000081395 : Ints8 : A0A0G2K0V1	0.11	yes	down	2.1E-05	yes	chr5	intron	last intron	NA	1.09	no	up	0.32	no	1.07	no	up	1.6E-05	yes
MSTRG.9900.3 : Rnaseh2a : Q5U209	14.16	yes	up	1.1E-05	yes	chr19	intron	last intron	NA	1.32	no	up	0.43	no	1.08	no	up	2.6E-05	yes
MSTRG.2788.2 : Hmox2 : P23711	inf	yes	up	5.3E-04	yes	chr10	exon	last exon	NA	1.28	no	up	0.67	no	1.09	no	up	4.1E-06	yes
ENSRNOT00000065224 : Thbs4 : F1LMS5	0.15	yes	down	4.0E-04	yes	chr2	intron	internal intron	NA	2.16	yes	up	0.08	no	1.25	yes	up	3.7E-10	yes
MSTRG.20752.1 : Ptpn18 : Q4KM54	0	yes	down	1.9E-04	yes	chr9	intron	first intron	NA	1.13	no	up	0.54	no	1.20	no	up	1.2E-08	yes
MSTRG.18734.1 : Derl1 : F7FNS3	0.48	yes	down	3.9E-05	yes	chr7	exon	last exon	NA	1.35	no	up	0.48	no	1.04	no	up	2.4E-03	yes
MSTRG.12226.1 : Bcr : F1LXF1	2.12	yes	up	1.3E-04	yes	chr20	intron	first intron	NA	0.86	no	down	0.25	no	0.96	no	down	8.7E-05	yes
MSTRG.10218.1 : Zc3h18 : Q6TQE1	0.03	yes	down	1.2E-11	yes	chr19	promoter	Intermediate	LCP	0.79	no	down	0.10	no	1.02	no	up	2.3E-01	no
MSTRG.2631.1 : Calhm2 : Q5RJQ8	0.13	yes	down	1.1E-04	yes	chr1	intron	first intron	NA	0.90	no	down	0.51	no	1.02	no	up	9.8E-02	no
MSTRG.17811.3 : Mark3 : F1M836	0.30	yes	down	2.6E-04	yes	chr6	exon	last exon	NA	1.03	no	up	0.93	no	1.10	no	up	4.1E-07	yes
MSTRG.11953.3 : RT1-CE7 : D3ZLE6	inf	yes	up	7.3E-07	yes	chr20	exon	internal exon	NA	1.20	no	up	0.79	no	1.22	yes	up	7.3E-16	yes
MSTRG.6560.4 : Hnrnpdl : A0A0G2KAZ7	inf	yes	up	7.6E-05	yes	chr14	intron	first intron	NA	0.92	no	down	0.65	no	0.99	no	down	4.6E-01	no
ENSRNOT00000019320 : Map1a : P34926	3.41	yes	up	1.8E-05	yes	chr3	intron	last intron	NA	1.00	no	down	0.99	no	1.03	no	up	8.9E-11	yes
MSTRG.7804.2 : Mycbp2 : D4A2D3	0.31	yes	down	9.2E-06	yes	chr15	intron	internal intron	NA	1.07	no	up	0.82	no	1.00	no	up	6.8E-01	no
MSTRG.3249.9 : Specc1 : A0A0G2K5D7	5.53	yes	up	1.9E-10	yes	chr10	exon	last exon	NA	0.85	no	down	0.48	no	0.93	no	down	4.4E-09	yes
MSTRG.1144.1 : Tarsl2 : Q5XI17	0.35	yes	down	1.8E-06	yes	chr1	intron	internal intron	NA	1.20	no	up	0.43	no	1.01	no	up	6.4E-01	no
MSTRG.8278.19 : Rbpms : F2Z3S5	0.02	yes	down	2.6E-05	yes	chr16	promoter	Proximal	ICP	0.95	no	down	0.80	no	1.06	no	up	4.9E-05	yes
ENSRNOT00000085654 : Atp6v1h : A0A0G2K9J2	0.31	yes	down	9.6E-05	yes	chr5	intron	internal intron	NA	1.15	no	up	0.25	no	1.09	no	up	2.6E-06	yes
MSTRG.13195.8 : Arhgap1 : D4A6C5	3.73	yes	up	1.8E-11	yes	chr3	intron	internal intron	NA	1.14	no	up	0.84	no	1.04	no	up	1.7E-04	yes
ENSRNOT00000084236 : Ncor2 : A0A0G2JU91	2.16	yes	up	1.6E-06	yes	chr12	intron	internal intron	NA	0.93	no	down	0.89	no	1.01	no	up	7.1E-01	no
ENSRNOT00000056392 : Rtf1 : D3ZLH8	0.17	yes	down	2.6E-07	yes	chr3	intron	first intron	NA	1.15	no	up	0.60	no	1.04	no	up	2.7E-04	yes
MSTRG.14918.3 : Nup210 : P11654	0.11	yes	down	6.6E-10	yes	chr4	intron	internal intron	NA	1.49	no	up	0.09	no	1.08	no	up	1.7E-07	yes
MSTRG.22091.3 : Arhgef6 : Q5XXR3	0.21	yes	down	2.1E-04	yes	chrX	intron	internal intron	NA	0.81	no	down	0.27	no	0.93	no	down	2.3E-08	yes
MSTRG.19329.2 : Atf7 : B0BMY0	0.38	yes	down	7.2E-06	yes	chr7	intron	internal intron	NA	1.06	no	up	0.80	no	1.00	no	down	9.7E-01	no
ENSRNOT00000035826 : Syne3 : D3ZD24	0.24	yes	down	2.1E-14	yes	chr6	intron	internal intron	NA	0.82	no	down	0.37	no	1.11	no	up	3.8E-04	yes
MSTRG.1684.1 : Il4r : Q63257	9.80	yes	up	2.9E-08	yes	chr1	intron	internal intron	NA	1.25	no	up	0.35	no	1.11	no	up	2.1E-05	yes
MSTRG.15948.5 : Cdk5rap2 : F1M4B7	0	yes	down	1.4E-04	yes	chr5	intron	internal intron	NA	1.07	no	up	0.76	no	0.98	no	down	2.6E-01	no
ENSRNOT00000076330 : Smndc1 : Q4QQU6	inf	yes	up	1.3E-04	yes	chr1	promoter	Distal	LCP	1.06	no	up	0.45	no	1.06	no	up	2.2E-04	yes
ENSRNOT00000019624 : Ndufb10 : D4A0T0	0.14	yes	down	1.1E-05	yes	chr10	promoter	Distal	ICP	0.85	no	down	0.32	no	0.97	no	down	8.5E-03	yes
ENSRNOT00000041330 : Adam9 : E9PTA4	0.15	yes	down	9.7E-05	yes	chr16	intron	internal intron	NA	0.97	no	down	0.77	no	1.06	no	up	3.4E-02	yes
MSTRG.20094.6 : Plscr1 : A0A0G2K7Q1	0.33	yes	down	1.3E-05	yes	chr8	promoter	Distal	LCP	0.90	no	down	0.42	no	1.08	no	up	5.2E-06	yes
ENSRNOT00000003867 : Gsk3b : A0A0G2JSH4	2.36	yes	up	1.7E-04	yes	chr11	intron	internal intron	NA	1.25	no	up	0.43	no	0.98	no	down	1.1E-02	yes
ENSRNOT00000024206 : Mcur1 : D3ZEJ2	0.10	yes	down	2.2E-05	yes	chr17	promoter	Distal	LCP	1.21	no	up	0.54	no	0.92	no	down	1.0E-11	yes
MSTRG.16461.11 : Pum1 : D3Z8L5	0.44	yes	down	3.1E-04	yes	chr5	intron	internal intron	NA	0.55	no	down	0.13	no	1.03	no	up	7.0E-02	no
MSTRG.12137.1 : Pknox1 : Q5BJP1	0.26	yes	down	3.4E-07	yes	chr20	promoter	Proximal	LCP	1.27	no	up	0.64	no	0.89	no	down	7.5E-04	yes
MSTRG.9435.6 : Tcerg1 : B5DEZ4	0.26	yes	down	2.8E-04	yes	chr18	promoter	Intermediate	ICP	1.13	no	up	0.41	no	1.02	no	up	4.4E-02	yes
MSTRG.16640.8 : Arhgef10l : M0R7W2	2.87	yes	up	9.9E-07	yes	chr5	intron	internal intron	NA	0.90	no	down	0.34	no	1.13	no	up	8.4E-04	yes
MSTRG.19125.2 : Brd1 : D3ZUW8	7.17	yes	up	3.3E-04	yes	chr7	exon	internal exon	NA	0.68	no	down	0.70	no	1.21	yes	up	6.4E-02	no
ENSRNOT00000083012 : Bclaf1 : B1WC16	0.67	yes	down	1.3E-07	yes	chr1	intron	internal intron	NA	0.97	no	down	0.57	no	1.03	no	up	6.8E-02	no
MSTRG.3895.9 : Cdk12 : A0A0G2K5U7	5.94	yes	up	1.1E-04	yes	chr10	exon	first exon	NA	1.00	no	down	0.99	no	0.97	no	down	5.5E-04	yes
MSTRG.2510.2 : Tm9sf3 : D3ZUD8	4.98	yes	up	1.2E-05	yes	chr1	promoter	Intermediate	LCP	0.39	yes	down	0.30	no	1.06	no	up	1.3E-07	yes
MSTRG.13433.1 : Casc4 : D3ZW58	0.14	yes	down	1.2E-10	yes	chr3	intron	first intron	NA	0.91	no	down	0.57	no	1.03	no	up	1.3E-02	yes
MSTRG.12279.1 : Ctnna3 : B2RYN9	0	yes	down	1.3E-06	yes	chr20	promoter	Distal	LCP	0.68	no	down	0.54	no	1.03	no	up	1.7E-01	no

MSTRG.276.1 : Arid1b : F1LNP1	1.53	yes	up	6.5E-08	yes	chr1	promoter	Proximal	LCP	1.15	no	up	0.87	no	0.98	no	down	1.8E-01	no
MSTRG.2839.15 : Srrm2 : A0A0G2K2M9	1.72	yes	up	9.0E-05	yes	chr10	exon	internal exon	NA	1.10	no	up	0.82	no	1.00	no	up	9.0E-01	no
ENSRNOT00000085132 : Vps39 : E9PT04	inf	yes	up	4.3E-04	yes	chr3	intron	internal intron	NA	0.83	no	down	0.68	no	1.08	no	up	2.7E-04	yes
MSTRG.14603.4 : Tax1bp1 : Q66HA4	8.48	yes	up	1.1E-06	yes	chr4	exon	last exon	NA	0.57	no	down	0.09	no	1.04	no	up	2.0E-04	yes
MSTRG.20746.1 : Bag2 : B0BN74	0.18	yes	down	4.5E-04	yes	chr9	promoter	Distal	ICP	0.89	no	down	0.34	no	1.05	no	up	1.1E-05	yes
ENSRNOT00000089557 : Sin3a : A0A0G2K3H5	0.53	yes	down	4.1E-06	yes	chr8	intron	last intron	NA	0.93	no	down	0.86	no	0.96	no	down	6.2E-05	yes
MSTRG.11405.2 : Pde4dip : A0A0G2JW66	6.94	yes	up	5.0E-08	yes	chr2	exon	internal exon	NA	1.39	no	up	0.02	yes	1.06	no	up	5.1E-04	yes
ENSRNOT00000024364 : Ubap2l : E9PTR4	6.62	yes	up	4.0E-04	yes	chr2	intron	internal intron	NA	0.97	no	down	0.49	no	1.13	no	up	3.8E-12	yes
MSTRG.123.6 : Epb41l2 : D3ZM69	0.25	yes	down	1.1E-12	yes	chr1	intron	internal intron	NA	0.71	no	down	0.12	no	0.91	no	down	8.9E-11	yes
MSTRG.9435.5 : Tcergl : B5DEZ4	0.26	yes	down	2.8E-04	yes	chr18	promoter	Intermediate	ICP	0.85	no	down	0.40	no	1.02	no	up	4.4E-02	yes
ENSRNOT00000088201 : Stat2 : Q5XI26	0.06	yes	down	1.0E-04	yes	chr7	promoter	Distal	LCP	1.01	no	up	0.78	no	1.14	no	up	1.9E-09	yes
ENSRNOT00000075474 : Ptprd : M0RB22	3.73	yes	up	1.3E-08	yes	chr5	exon	internal exon	NA	1.00	no	down	0.89	no	1.01	no	up	5.3E-01	no
ENSRNOT00000019781 : Smurf2 : F1M3F2	3.77	yes	up	9.3E-06	yes	chr10	intron	internal intron	NA	1.98	no	up	0.10	no	1.03	no	up	6.3E-02	no
MSTRG.6732.2 : Limch1 : F1M392	1.09	no	up	3.3E-04	yes	chr14	intron	last intron	NA	0.78	no	down	0.48	no	0.85	no	down	1.1E-13	yes
ENSRNOT00000023605 : Tpm3 : A0A140TAF0	0.37	yes	down	8.0E-05	yes	chr2	intron	internal intron	NA	0.91	no	down	0.63	no	1.03	no	up	4.4E-04	yes
MSTRG.5482.17 : Ncor2 : A0A0G2JU91	2.16	yes	up	1.6E-06	yes	chr12	intron	internal intron	NA	1.01	no	up	0.94	no	1.01	no	up	7.1E-01	no
ENSRNOT00000025086 : Nup93 : Q66HC5	4.51	yes	up	2.5E-04	yes	chr19	exon	internal exon	NA	1.32	no	up	0.10	no	0.97	no	down	1.0E-07	yes
ENSRNOT00000020670 : Atp5d : G3V7Y3	0.12	yes	down	5.2E-08	yes	chr7	promoter	Distal	LCP	3.48	yes	up	0.36	no	0.90	no	down	7.4E-12	yes
ENSRNOT00000007427 : Ybx3 : D4A0L4	0.20	yes	down	8.8E-05	yes	chr4	intron	internal intron	NA	0.97	no	down	0.91	no	1.24	yes	up	8.2E-11	yes
ENSRNOT00000093684 : Nup214 : M0RBV9	0.13	yes	down	4.7E-04	yes	chr3	intron	internal intron	NA	1.05	no	up	0.50	no	1.01	no	up	5.4E-01	no
MSTRG.13080.3 : Lnpk : A0JN29	3.42	yes	up	1.2E-11	yes	chr3	intron	internal intron	NA	0.87	no	down	0.50	no	0.96	no	down	2.6E-04	yes
MSTRG.7561.8 : Phf11b : M0RB46	3.03	yes	up	2.9E-04	yes	chr15	promoter	Intermediate	LCP	1.50	no	up	0.12	no	1.15	no	up	2.3E-06	yes
MSTRG.2579.4 : Sfxn3 : Q9JHY2	0.39	yes	down	7.8E-05	yes	chr1	intron	internal intron	NA	0.59	no	down	0.29	no	0.98	no	down	7.5E-03	yes
MSTRG.7561.11 : Phf11b : M0RB46	3.03	yes	up	2.9E-04	yes	chr15	promoter	Intermediate	LCP	1.43	no	up	0.34	no	1.15	no	up	2.3E-06	yes
ENSRNOT00000005031 : Hmox2 : P23711	inf	yes	up	5.3E-04	yes	chr10	exon	last exon	NA	0.95	no	down	0.57	no	1.09	no	up	4.1E-06	yes
MSTRG.18475.1 : Pawr : G3V6S1	4.33	yes	up	2.3E-06	yes	chr7	intron	last intron	NA	0.88	no	down	0.60	no	1.06	no	up	9.6E-07	yes
MSTRG.12337.6 : Sgpl1 : Q8CHN6	0.20	yes	down	6.8E-07	yes	chr20	intron	internal intron	NA	1.20	no	up	0.46	no	1.12	no	up	5.6E-16	yes
MSTRG.17785.1 : Dync1h1 : M0R9X8	0.19	yes	down	1.5E-06	yes	chr6	intron	internal intron	NA	0.63	no	down	0.53	no	0.99	no	down	3.4E-03	yes
MSTRG.21023.6 : Fn1 : F1LST1	0.11	yes	down	7.7E-11	yes	chr9	intron	internal intron	NA	7.56	yes	up	0.49	no	1.27	yes	up	4.1E-20	yes
ENSRNOT00000065111 : Bcar3 : D3ZAZ5	3.50	yes	up	1.2E-08	yes	chr2	intron	internal intron	NA	1.23	no	up	0.59	no	1.06	no	up	1.2E-02	yes
MSTRG.15546.1 : Asph : A0A096MKE0	2.56	yes	up	9.8E-06	yes	chr5	intron	internal intron	NA	1.07	no	up	0.83	no	0.99	no	down	1.3E-01	no
MSTRG.5482.24 : Ncor2 : A0A0G2JU91	2.16	yes	up	1.6E-06	yes	chr12	intron	internal intron	NA	1.20	no	up	0.54	no	1.01	no	up	7.1E-01	no
MSTRG.13963.2 : Ptgis : Q62969	0.55	yes	down	6.0E-05	yes	chr3	intron	internal intron	NA	0.98	no	down	0.98	no	0.99	no	down	7.4E-02	no
ENSRNOT00000072698 : Mest : M0R830	0.59	yes	down	9.4E-06	yes	chr4	intron	first intron	NA	0.64	no	down	0.17	no	0.86	no	down	1.4E-06	yes
MSTRG.10288.1 : Gnpat : Q9ES71	inf	yes	up	4.2E-12	yes	chr19	exon	last exon	NA	0.95	no	down	0.52	no	1.10	no	up	6.9E-05	yes
MSTRG.8033.3 : Slc27a1 : Q6GMM8	0.26	yes	down	5.6E-15	yes	chr16	intron	internal intron	NA	1.20	no	up	0.68	no	0.99	no	down	2.5E-01	no
MSTRG.5548.1 : P2rx4 : P51577	2.45	yes	up	6.7E-18	yes	chr12	intron	internal intron	NA	0.79	no	down	0.60	no	1.15	no	up	7.4E-08	yes
ENSRNOT00000066950 : Dapk1 : F1LNN8	0.35	yes	down	1.5E-04	yes	chr17	intron	internal intron	NA	0.78	no	down	0.42	no	0.96	no	down	1.0E-01	no
MSTRG.15231.9 : Tspan9 : D4AAV9	0.41	yes	down	1.4E-09	yes	chr4	exon	last exon	NA	1.45	no	up	0.22	no	0.96	no	down	2.4E-05	yes
ENSRNOT00000023628 : Hspa4 : F1LRV4	0.10	yes	down	2.8E-04	yes	chr10	intron	internal intron	NA	1.15	no	up	0.14	no	0.69	yes	down	5.3E-07	yes
MSTRG.15231.11 : Tspan9 : D4AAV9	0.41	yes	down	1.4E-09	yes	chr4	exon	last exon	NA	0.46	yes	down	0.45	no	0.96	no	down	2.4E-05	yes
MSTRG.9900.5 : Rnaseh2a : Q5U209	14.16	yes	up	1.1E-05	yes	chr19	intron	last intron	NA	1.42	no	up	0.06	no	1.08	no	up	2.6E-05	yes
MSTRG.17422.5 : Ppm1a : P20650	2.35	yes	up	3.9E-04	yes	chr6	intron	last intron	NA	0.90	no	down	0.88	no	0.96	no	down	5.3E-04	yes
ENSRNOT00000007300 : Snx6 : B5DEY8	21.45	yes	up	3.0E-04	yes	chr6	exon	internal exon	NA	0.97	no	down	0.82	no	0.97	no	down	1.8E-09	yes
MSTRG.20878.6 : Gls : A0A0G2KAN7	0.05	yes	down	1.5E-13	yes	chr9	intron	first intron	NA	1.14	no	up	0.89	no	1.09	no	up	8.3E-03	yes
MSTRG.19538.13 : Nfrkb : D4A421	0.58	yes	down	1.3E-05	yes	chr8	intron	internal intron	NA	1.20	no	up	0.25	no	0.89	no	down	2.5E-03	yes
MSTRG.8278.18 : Rbpms : F2Z3S5	0.02	yes	down	2.6E-05	yes	chr16	promoter	Proximal	ICP	1.27	no	up	0.54	no	1.06	no	up	4.9E-05	yes
ENSRNOT00000004524 : Rbfox3 : A0A0G2K2J4	0.25	yes	down	5.8E-04	yes	chr10	exon	internal exon	NA	1.00	no	down	0.47	no	0.94	no	down	8.2E-05	yes
ENSRNOT00000028246 : Atp8b2 : D4A509	0.14	yes	down	3.1E-04	yes	chr2	exon	first exon	NA	0.90	no	down	0.76	no	0.92	no	down	5.0E-03	yes
MSTRG.5704.2 : RGD1306556 : A0A0G2JZ88	0.24	yes	down	1.7E-04	yes	chr12	exon	internal exon	NA	0.58	no	down	0.40	no	1.28	yes	up	1.1E-07	yes
ENSRNOT00000093370 : Mycbp2 : A0A1W2Q6I3	0.31	yes	down	9.2E-06	yes	chr15	intron	internal intron	NA	0.94	no	down	0.81	no	1.00	no	up	8.8E-01	no
MSTRG.20927.1 : Aox1 : F1LRQ1	0.36	yes	down	1.5E-07	yes	chr9	intron	internal intron	NA	0.71	no	down	0.43	no	0.73	yes	down	3.5E-17	yes
ENSRNOT00000092223 : Atad2b : A0A096MKA5	3.51	yes	up	9.0E-07	yes	chr6	intron	internal intron	NA	0.96	no	down	0.45	no	1.15	no	up	9.1E-06	yes
ENSRNOT00000078991 : Ptcd3 : D3ZGM1	3.88	yes	up	4.1E-35	yes	chr4	intron	internal intron	NA	0.97	no	down	0.96	no	0.98	no	down	3.1E-01	no
MSTRG.13963.6 : Ptgis : Q62969	0.55	yes	down	6.0E-05	yes	chr3	intron	internal intron	NA	2.35	yes	up	0.48	no	0.99	no	down	7.4E-02	no
MSTRG.6919.4 : Cpz : A0A0G2JSJ7	3.01	yes	up	1.1E-05	yes	chr14	intron	internal intron	NA	1.45	no	up	0.49	no	1.08	no	up	5.1E-02	no
ENSRNOT00000092086 : Picalm : Q66WT9	8.74	yes	up	2.1E-13	yes	chr1	intron	internal intron	NA	0.60	no	down	0.06	no	1.45	yes	up	1.6E-10	yes
MSTRG.11265.1 : Ubap2l : E9PTR4	6.62	yes	up	4.0E-04	yes	chr2	intron	internal intron	NA	2.12	yes	up	0.19	no	1.13	no	up	3.8E-12	yes
MSTRG.1731.7 : Tbc1d10b : D3ZSY8	1.59	yes	up	2.7E-04	yes	chr1	intron	internal intron	NA	1.08	no	up	0.89	no	0.99	no	down	3.1E-01	no
ENSRNOT00000022746 : Gramd4 : F1LYJ8	0.57	yes	down	9.4E-05	yes	chr7	intron	internal intron	NA	0.78	no	down	0.53	no	1.32	yes	up	2.0E-09	yes
MSTRG.12403.2 : Msl3l2 : Q6AYG1	0.29	yes	down	7.4E-06	yes	chr20	exon	first exon	NA	0.75	no	down	0.44	no	0.89	no	down	2.3E-05	yes
MSTRG.19194.6 : Hdac7 : A0A0G2K6B1	1.57	yes	up	6.8E-05	yes	chr7	intron	internal intron	NA	1.20	no	up	0.14	no	0.99	no	down	6.7E-01	no
MSTRG.19194.7 : Hdac7 : A0A0G2K6B1	1.57	yes	up	6.8E-05	yes	chr7	intron	internal intron	NA	0.90	no	down	0.61	no	0.99	no	down	6.7E-01	no
ENSRNOT00000011808 : Tmem97 : Q5U3Y7	0.12	yes	down	2.2E-04	yes	chr10	promoter	Distal	LCP	1.86	no	up	0.41	no	0.91	no	down	2.5E-11	yes
MSTRG.21039.6 : Tns1 : F1LN42	0.09	yes	down	2.0E-04	yes	chr9	intron	last intron	NA	0.77	no	down	0.74	no	0.86	no	down	7.8E-19	yes
MSTRG.611.2 : Tomm40 : G3V8F5	0.23	yes	down	3.2E-04	yes	chr1	promoter	Distal	LCP	0.57	no	down	0.59	no	1.06	no	up	1.1E-05	yes
MSTRG.16755.3 : Kif1b : A0A0G2KA12	3.65	yes	up	7.4E-05	yes	chr5	intron	internal intron	NA	1.13	no	up	0.03	yes	1.05	no	up	6.5E-02	no
MSTRG.97.8 : Bclaf1 : B1WC16	0.67	yes	down	1.3E-07	yes	chr1	intron	internal intron	NA	1.52	no	up	0.16	no	1.03	no	up	6.8E-02	no
MSTRG.18210.1 : Stk11 : A0A0H2UI02	0.18	yes	down	9.1E-05	yes	chr7	exon	last exon	NA	0.93	no	down	0.62	no	0.88	no	down	1.8E-03	yes
ENSRNOT00000046273 : Atp2b4 : Q64542	2.31	yes	up	1.3E-05	yes	chr13	intron	internal intron	NA	1.01	no	up	0.89	no	0.68	yes	down	2.6E-19	yes
MSTRG.9417.1 : Nr3c1 : P06536	0.27	yes	down	1.2E-04	yes	chr18	exon	first exon	NA	0.66	no	down	0.66	no	0.91	no	down	1.6E-10	yes
ENSRNOT00000042307 : Eml4 : A0A0G2K2Z0	0.79	yes	down	2.3E-04	yes	chr6	exon	internal exon	NA	0.96	no	down	0.64	no	1.03	no	up	6.8E-04	yes
ENSRNOT00000014377 : Sec13 : Q5XFW8	0.17	yes	down	8.7E-10	yes	chr4	exon	internal exon	NA	1.45	no	up	0.28	no	1.04	no	up	9.2E-06	yes
ENSRNOT00000072059 : Prdm16 : M0RDL0	0.37	yes	down	2.7E-04	yes	chr5	exon	internal exon	NA	1.14	no	up	0.17	no	0.83	yes	down	1.1E-06	yes
ENSRNOT00000032162 : Gart : G3V918	0.39	yes	down	2.0E-05	yes	chr11	intron	internal intron	NA	1.20	no	up	0.13	no	0.96	no	down	6.3E-04	yes
MSTRG.20352.4 : Ubp1 : D4A030	0.44	yes	down	2.3E-04	yes	chr8	intron	internal intron	NA	1.11	no	up	0.31	no	0.98	no	down	1.9E-01	no
ENSRNOT00000068484 : Ankh : F1LN34	3.44	yes	up	9.7E-06	yes	chr2	intron	first intron	NA	1.35	no	up	0.11	no	1.20	yes	up	7.4E-12	yes
MSTRG.3895.10 : Cdk12 : A0A0G2K5U7	5.94	yes	up	1.1E-04	yes	chr10	exon	first exon	NA	1.04	no	up	0.81	no	0.97	no	down	5.5E-04	yes
ENSRNOT00000092988 : Gtf2i : Q5U2Y1	5.31	yes	up	4.9E-06	yes	chr12	promoter	Distal	LCP	1.03	no	up	0.89	no	0.95	no	down	1.5E-07	yes
MSTRG.13870.4 : Plcg1 : G3V845	0.24	yes	down	1.1E-04	yes	chr3	intron	first intron	NA	0.95	no	down	0.84	no	0.94	no	down	1.8E-09	yes
MSTRG.10847.1 : Gnb4 : O35353	2.44	yes	up	1.4E-05	yes	chr2	intron	last intron	NA	1.21	no	up	0.58	no	0.91	no	down	2.4E-06	yes
ENSRNOT00000020853 : Prmt3 : O70467	1.85	yes	up	1.1E-04	yes	chr1	intron	internal intron	NA	1.69	no	up	0.04	yes	1.06	no	up	2.2E-04	yes
MSTRG.15720.1 : Pigo : D3ZTP8	0.61	yes	down	2.7E-05	yes	chr5	exon	internal exon	NA	2.87	yes	up	0.01	yes	0.80	yes	down	9.0E-03	yes
ENSRNOT00000032454 : Ss18 : Q5XI66	6.27	yes	up	1.6E-10	yes	chr18	exon	internal exon	NA	1.84	no	up	0.20	no	1.04	no	up	6.9E-02	no
MSTRG.18557.5 : Mon2 : D3ZCG3	0.07	yes	down	1.5E-08	yes	chr7	intron	internal intron	NA	0.80	no	down	0.47	no	1.05	no	up	7.1E-07	yes
ENSRNOT00000034472 : Dync1li2 : Q5D023	0.17	yes	down	2.9E-04	yes	chr19	intron	last intron	NA	1.60	no	up	0.36	no	0.99	no	down	1.1E-01	no
MSTRG.18268.21 : Akap8 : Q63014	0.08	yes	down	2.7E-04	yes	chr7	intron	internal intron	NA	2.16	yes	up	0.06	no	0.99	no	down	2.8E-01	no
MSTRG.12911.1 : Scai : F1M3P6	0.24	yes	down	1.2E-04	yes	chr3	intron	internal intron	NA	1.01	no	up	0.86	no	0.86	no	down	1.0E-11	yes
ENSRNOT00000002487 : Rfc4 : B4F778	43.52	yes	up	4.1E-16	yes	chr11	promoter	Proximal	ICP	1.10	no	up	0.58	no	0.99	no	down	5.5E-01	no
MSTRG.19088.3 : Scube1 : F1M987	2.13	yes	up	1.3E-04	yes	chr7	intron	internal intron	NA	0.66	no	down	0.18	no	0.80	yes	down	1.2E-14	yes
ENSRNOT00000071386 : Ranbp2 : M0R3M4	0.30	yes	down	3.3E-11	yes	chr20	intron	internal intron	NA	1.09	no	up	0.20	no	1.00	no	down	6.2E-01	no
MSTRG.11953.55 : RT1-CE7 : D3ZLE6	inf	yes	up	7.3E-07	yes	chr20	exon	internal exon	NA	0.51	no	down	0.40	no	1.22	yes	up	7.3E-16	yes
MSTRG.4248.2 : Mxra7 : F1M1U0	inf	yes	up	4.6E-04	yes	chr10	intron	internal intron	NA	0.83	no	down	0.74	no	1.16	no	up	7.7E-10	yes
ENSRNOT00000018796 : Pgrmc2 : Q5XIU9	9.90	yes	up	5.8E-08	yes	chr2	intron	first intron	NA	0.94	no	down	0.72	no	0.97	no	down	1.4E-04	yes
MSTRG.6635.4 : Rufy3 : A0A0G2K6A9	2.37	yes	up	3.9E-04	yes	chr14	promoter	Distal	LCP	1.14	no	up	0.70	no	1.11	no	up	1.3E-05	yes
ENSRNOT00000019778 : Csgalnact2 : D4A5Z0	2.74	yes	up	6.9E-07	yes	chr4	intron	internal intron	NA	1.14	no	up	0.32	no	0.94	no	down	1.2E-02	yes
ENSRNOT00000083467 : Usp54 : Q6IE24	0.36	yes	down	1.9E-04	yes	chr15	intron	internal intron	NA	0.80	no	down	0.68	no	0.83	yes	down	7.0E-09	yes
ENSRNOT00000077999 : Myzap : Q5EB94	0.16	yes	down	3.6E-06	yes	chr8	exon	last exon	NA	0.75	no	down	0.27	no	0.84	no	down	4.4E-10	yes
ENSRNOT00000080651 : Usp9x : D3ZC84	21.85	yes	up	4.1E-05	yes	chrX	exon	last exon	NA	1.04	no	up	0.55	no	1.00	no	up	7.5E-01	no
ENSRNOT00000093137 : Gpx4 : A0A0G2K398	0.42	yes	down	9.7E-05	yes	chr7	intron	internal intron	NA	0.91	no	down	0.80	no	0.91	no	down	1.6E-05	yes

ENSRNOT00000033719 : Trex1 : Q5BK16	0.35	yes	down	1.1E-04	yes	chr8	promoter	Distal	LCP	0.44	yes	down	0.16	no	1.21	yes	up	5.1E-11	yes
MSTRG.12174.5 : Col18a1 : F1LR02	0.22	yes	down	4.1E-11	yes	chr20	exon	internal exon	NA	0.80	no	down	0.57	no	1.03	no	up	3.6E-07	yes
MSTRG.20920.4 : Maip1 : Q6AY04	27.81	yes	up	1.4E-21	yes	chr9	promoter	Intermediate	ICP	0.70	no	down	0.36	no	0.87	no	down	5.5E-04	yes
MSTRG.13195.3 : Arhgap1 : D4A6C5	3.73	yes	up	1.8E-11	yes	chr3	intron	internal intron	NA	1.24	no	up	0.67	no	1.04	no	up	1.7E-04	yes
ENSRNOT00000092976 : Arid1b : F1LNP1	1.53	yes	up	6.5E-08	yes	chr1	promoter	Proximal	LCP	1.84	no	up	0.23	no	0.98	no	down	1.8E-01	no
MSTRG.4525.9 : Mx1 : Q499S4	3.65	yes	up	1.1E-13	yes	chr11	intron	internal intron	NA	2.43	yes	up	0.33	no	1.36	yes	up	1.2E-13	yes
MSTRG.21176.3 : Inpp5d : F1M981	0.08	yes	down	9.9E-05	yes	chr9	intron	internal intron	NA	0.71	no	down	0.20	no	1.08	no	up	3.1E-05	yes
MSTRG.8278.10 : Rbpms : F2Z3S5	0.02	yes	down	2.6E-05	yes	chr16	promoter	Proximal	ICP	0.82	no	down	0.64	no	1.06	no	up	4.9E-05	yes
MSTRG.9425.3 : Nr3c1 : P06536	0.27	yes	down	1.2E-04	yes	chr18	exon	first exon	NA	0.95	no	down	0.72	no	0.91	no	down	1.6E-10	yes
ENSRNOT00000049292 : Kalrn : P97924	1.54	yes	up	9.1E-07	yes	chr11	exon	last exon	NA	0.93	no	down	0.53	no	1.04	no	up	1.6E-04	yes
ENSRNOT00000006403 : Rock2 : F1LQT3	0.10	yes	down	2.0E-24	yes	chr6	intron	internal intron	NA	1.11	no	up	0.85	no	0.97	no	down	1.9E-04	yes
ENSRNOT00000092645 : Srsf3 : A0A0U1RRV7	4.40	yes	up	1.5E-08	yes	chr20	intron	internal intron	NA	0.98	no	down	0.86	no	0.94	no	down	5.5E-08	yes
ENSRNOT00000003437 : Pds5a : A4L9P7	1.26	yes	up	1.3E-13	yes	chr14	exon	last exon	NA	1.17	no	up	0.04	yes	1.01	no	up	2.7E-01	no
ENSRNOT00000067875 : Spp1 : P08721	29.54	yes	up	1.8E-44	yes	chr14	exon	last exon	NA	7.36	yes	up	0.06	no	1.32	yes	up	5.2E-14	yes
MSTRG.2881.12 : Pkd1 : Q9ERV0	inf	yes	up	6.6E-05	yes	chr10	intron	internal intron	NA	1.15	no	up	0.62	no	1.19	no	up	9.8E-07	yes
ENSRNOT00000089305 : Actn4 : Q9QXQ0	0.43	yes	down	2.1E-04	yes	chr1	intron	internal intron	NA	1.00	no	down	0.99	no	1.01	no	up	3.9E-03	yes
MSTRG.1443.11 : Arap1 : F1LM60	2.71	yes	up	3.3E-04	yes	chr1	intron	first intron	NA	1.36	no	up	0.09	no	0.99	no	down	2.5E-02	yes
ENSRNOT00000076879 : Selp : A0A096MK10	inf	yes	up	7.4E-05	yes	chr13	promoter	Distal	LCP	2.15	yes	up	0.27	no	0.93	no	down	6.5E-03	yes
MSTRG.9435.8 : Tcerg1 : B5DEZ4	0.26	yes	down	2.8E-04	yes	chr18	promoter	Intermediate	ICP	1.30	no	up	0.09	no	1.02	no	up	4.4E-02	yes
MSTRG.21427.2 : Rbm3 : Q925G0	5.50	yes	up	1.2E-04	yes	chrX	intron	first intron	NA	0.88	no	down	0.75	no	1.28	yes	up	1.7E-10	yes
ENSRNOT00000024187 : Ces1f : M0R7R1	0.26	yes	down	2.8E-08	yes	chr19	intron	internal intron	NA	0.95	no	down	0.74	no	0.86	no	down	3.2E-11	yes
MSTRG.15096.5 : Cacna1c : F1MA84	4.75	yes	up	4.3E-04	yes	chr4	intron	internal intron	NA	0.81	no	down	0.46	no	0.97	no	down	2.7E-02	yes
ENSRNOT00000077443 : Slc27a6 : D4A2B8	0.27	yes	down	1.3E-05	yes	chr18	intron	internal intron	NA	1.00	no	down	0.99	no	1.07	no	up	2.7E-02	yes
ENSRNOT00000080038 : Ylpm1 : A0A0G2K678	0.14	yes	down	2.3E-07	yes	chr6	intron	internal intron	NA	0.93	no	down	0.63	no	1.02	no	up	1.8E-03	yes
MSTRG.19503.1 : Anln : M0RDG0	0.78	yes	down	4.3E-04	yes	chr8	intron	internal intron	NA	1.00	no	down	1.00	no	0.97	no	down	9.1E-01	no
MSTRG.1683.6 : Il4r : Q63257	9.80	yes	up	2.9E-08	yes	chr1	intron	internal intron	NA	1.55	no	up	0.43	no	1.11	no	up	2.1E-05	yes
MSTRG.10589.5 : Itga1 : P18614	0.09	yes	down	9.6E-08	yes	chr2	intron	internal intron	NA	1.73	no	up	0.05	yes	0.81	yes	down	3.6E-17	yes
MSTRG.12973.3 : Rbms1 : A0A0G2K4R7	0	yes	down	3.3E-05	yes	chr3	intron	internal intron	NA	1.36	no	up	0.44	no	0.97	no	down	1.2E-01	no
ENSRNOT00000024452 : Lactb : D3ZFJ6	4.28	yes	up	2.7E-14	yes	chr8	exon	last exon	NA	0.97	no	down	0.74	no	1.12	no	up	4.3E-10	yes
ENSRNOT00000056862 : Sema4a : A0A1B0GWV9	3.09	yes	up	1.9E-04	yes	chr2	promoter	Intermediate	LCP	1.54	no	up	0.03	yes	1.07	no	up	2.4E-05	yes
ENSRNOT00000067085 : Cd59 : P27274	5.87	yes	up	3.8E-04	yes	chr3	exon	last exon	NA	1.38	no	up	0.02	yes	0.93	no	down	5.0E-05	yes
MSTRG.13466.6 : Myef2 : A0A0G2K402	2.63	yes	up	2.7E-04	yes	chr3	promoter	Intermediate	LCP	0.78	no	down	0.43	no	0.93	no	down	4.5E-09	yes
ENSRNOT00000028188 : Ddb1 : G3V8T4	inf	yes	up	1.9E-05	yes	chr1	intron	internal intron	NA	1.10	no	up	0.45	no	0.97	no	down	4.7E-09	yes
ENSRNOT00000068711 : Klhl22 : D3ZZC3	2.85	yes	up	3.5E-06	yes	chr11	intron	internal intron	NA	1.61	no	up	0.08	no	0.98	no	down	1.1E-01	no
ENSRNOT00000018114 : Ddx41 : B2RYL8	9.95	yes	up	1.6E-09	yes	chr17	exon	last exon	NA	1.04	no	up	0.66	no	1.02	no	up	2.7E-01	no
ENSRNOT00000001564 : Ndufv3 : G3V644	0	yes	down	1.9E-04	yes	chr20	intron	first intron	NA	1.04	no	up	0.75	no	1.00	no	up	9.0E-01	no
ENSRNOT00000023145 : Cep192 : D4A3X0	4.22	yes	up	1.3E-12	yes	chr18	intron	internal intron	NA	1.00	no	down	0.48	no	0.95	no	down	9.1E-04	yes
MSTRG.14075.4 : Dnajc5 : A0A0G2JX56	0.30	yes	down	1.1E-06	yes	chr3	promoter	Intermediate	HCP	0.52	no	down	0.05	yes	0.89	no	down	4.0E-08	yes
ENSRNOT00000079796 : Dpt : B2RZ77	6.14	yes	up	2.8E-04	yes	chr13	exon	last exon	NA	0.52	no	down	0.20	no	0.85	no	down	3.5E-10	yes
MSTRG.20878.3 : Gls : A0A0G2KAN7	0.05	yes	down	1.5E-13	yes	chr9	intron	first intron	NA	1.28	no	up	0.55	no	1.09	no	up	8.3E-03	yes
ENSRNOT00000025664 : Stx5 : Q08851	0	yes	down	9.9E-06	yes	chr1	exon	first exon	NA	1.00	no	down	1.00	no	1.01	no	up	5.9E-01	no
MSTRG.19221.1 : Kmt2d : A0A0G2JVD6	6.53	yes	up	2.1E-09	yes	chr7	exon	last exon	NA	0.74	no	down	0.45	no	1.03	no	up	3.5E-02	yes
ENSRNOT00000089229 : Golim4 : D3ZM57	0.34	yes	down	3.2E-04	yes	chr2	intron	internal intron	NA	1.07	no	up	0.67	no	0.55	yes	down	1.1E-01	no
ENSRNOT00000064951 : Pds5b : D3ZU56	0.26	yes	down	2.1E-04	yes	chr12	promoter	Intermediate	LCP	1.00	no	down	0.84	no	0.96	no	down	4.7E-06	yes
MSTRG.11582.3 : Palmd : Q4KM62	0.16	yes	down	7.2E-05	yes	chr2	promoter	Distal	LCP	0.79	no	down	0.08	no	0.77	yes	down	4.3E-06	yes
ENSRNOT00000001207 : Alox5ap : P20291	3.07	yes	up	3.3E-04	yes	chr12	intron	first intron	NA	1.73	no	up	0.25	no	0.96	no	down	8.4E-03	yes
ENSRNOT00000086114 : Itga1 : P18614	0.09	yes	down	9.6E-08	yes	chr2	intron	internal intron	NA	0.88	no	down	0.48	no	0.81	yes	down	3.6E-17	yes
MSTRG.14711.1 : Mat2a : F1LRB8	0.10	yes	down	1.4E-04	yes	chr4	exon	last exon	NA	0.82	no	down	0.60	no	0.99	no	down	1.5E-01	no
ENSRNOT00000083701 : Prom1 : Q9JI49	8.32	yes	up	2.4E-09	yes	chr14	intron	internal intron	NA	0.82	no	down	0.50	no	0.87	no	down	1.5E-05	yes
ENSRNOT00000012622 : Ptcd3 : D3ZGM1	3.88	yes	up	4.1E-35	yes	chr4	intron	internal intron	NA	0.78	no	down	0.54	no	0.98	no	down	3.1E-01	no
MSTRG.19956.4 : Myo1e : Q63356	3.15	yes	up	9.4E-05	yes	chr8	intron	first intron	NA	1.64	no	up	0.48	no	1.03	no	up	1.7E-02	yes
MSTRG.15231.1 : Tspan9 : D4AAV9	0.41	yes	down	1.4E-09	yes	chr4	exon	last exon	NA	1.20	no	up	0.63	no	0.96	no	down	2.4E-05	yes
ENSRNOT00000075967 : Gigyf2 : A0A096MJI4	0.59	yes	down	1.9E-05	yes	chr9	intron	internal intron	NA	0.93	no	down	0.78	no	1.31	yes	up	2.3E-10	yes
MSTRG.18880.6 : Plec : Q6S395	0.33	yes	down	4.2E-06	yes	chr7	exon	last exon	NA	0.59	no	down	0.48	no	1.03	no	up	2.2E-01	no
MSTRG.10163.1 : Cdh13 : Q8R490	2.07	yes	up	3.3E-06	yes	chr19	promoter	Intermediate	LCP	1.24	no	up	0.55	no	0.83	yes	down	2.3E-16	yes
MSTRG.15469.3 : Tram1 : Q5XI41	3.29	yes	up	2.9E-05	yes	chr5	exon	last exon	NA	0.52	no	down	0.45	no	1.13	no	up	1.2E-09	yes
MSTRG.20742.5 : Dst : D3ZC56	0.35	yes	down	1.3E-05	yes	chr9	intron	internal intron	NA	1.35	no	up	0.47	no	1.03	no	up	7.3E-04	yes
ENSRNOT00000065181 : Atp2a3 : G3V9U7	inf	yes	up	2.1E-05	yes	chr10	intron	internal intron	NA	0.98	no	down	0.59	no	0.77	yes	down	1.7E-16	yes
ENSRNOT00000018711 : Cap1 : Q08163	0.21	yes	down	1.6E-07	yes	chr5	intron	internal intron	NA	2.33	yes	up	0.00	yes	0.94	no	down	1.0E-08	yes
MSTRG.19329.4 : Atf7 : B0BMY0	0.38	yes	down	7.2E-06	yes	chr7	intron	internal intron	NA	0.34	yes	down	0.17	no	1.00	no	down	9.7E-01	no
MSTRG.6862.1 : C1qtnf7 : B2RYB7	3.60	yes	up	9.8E-06	yes	chr14	exon	last exon	NA	0.87	no	down	0.36	no	0.87	no	down	2.3E-09	yes
ENSRNOT00000075996 : Dpt : B2RZ77	6.14	yes	up	2.8E-04	yes	chr13	exon	last exon	NA	0.52	no	down	0.02	yes	0.85	no	down	3.5E-10	yes
MSTRG.2561.2 : Chuk : B5DF32	24.13	yes	up	2.0E-05	yes	chr1	intron	internal intron	NA	1.09	no	up	0.55	no	0.90	no	down	8.1E-05	yes
MSTRG.1783.8 : Tgfb1i1 : Q99PD6	3.08	yes	up	7.2E-05	yes	chr1	intron	internal intron	NA	0.85	no	down	0.53	no	1.06	no	up	1.6E-04	yes
ENSRNOT00000089683 : Ylpm1 : A0A0G2K678	0.14	yes	down	2.3E-07	yes	chr6	intron	internal intron	NA	0.93	no	down	0.60	no	1.02	no	up	1.8E-03	yes
ENSRNOT00000020402 : Sae1 : Q6AXQ0	6.24	yes	up	9.5E-08	yes	chr1	intron	internal intron	NA	0.99	no	down	0.83	no	0.99	no	down	1.2E-02	yes
ENSRNOT00000088619 : Rbms2 : Q4QR81	0.04	yes	down	2.6E-23	yes	chr7	exon	last exon	NA	0.84	no	down	0.82	no	1.14	no	up	2.7E-10	yes
ENSRNOT00000056592 : Pabpc4 : G3V9N0	15.58	yes	up	1.8E-05	yes	chr5	intron	first intron	NA	1.18	no	up	0.27	no	1.10	no	up	6.8E-12	yes
ENSRNOT00000008705 : Yipf6 : A0A096MJG6	inf	yes	up	3.8E-06	yes	chrX	intron	first intron	NA	1.11	no	up	0.62	no	1.03	no	up	1.9E-01	no
ENSRNOT00000088138 : Acaca : P11497	0.15	yes	down	3.0E-04	yes	chr10	intron	internal intron	NA	0.93	no	down	0.77	no	0.97	no	down	3.1E-04	yes
ENSRNOT00000020581 : Dnajc5 : A0A0G2JX56	0.30	yes	down	1.1E-06	yes	chr3	promoter	Intermediate	HCP	0.99	no	down	0.50	no	0.89	no	down	4.0E-08	yes
MSTRG.7350.2 : Ktn1 : D4A4Z9	2.49	yes	up	1.2E-04	yes	chr15	intron	internal intron	NA	1.43	no	up	0.02	yes	1.04	no	up	2.2E-11	yes
MSTRG.18971.4 : Triobp : A2TIS7	0.29	yes	down	2.0E-06	yes	chr7	intron	internal intron	NA	0.68	no	down	0.00	yes	0.97	no	down	5.1E-06	yes
ENSRNOT00000009806 : Cc2d1a : Q66HA5	0.21	yes	down	1.7E-05	yes	chr19	promoter	Distal	LCP	1.08	no	up	0.22	no	0.96	no	down	4.1E-02	yes
ENSRNOT00000014887 : Dnase2 : Q9QZK8	0.04	yes	down	1.6E-06	yes	chr19	exon	last exon	NA	1.13	no	up	0.38	no	1.04	no	up	8.4E-05	yes
MSTRG.6855.2 : Prom1 : Q9JI49	8.32	yes	up	2.4E-09	yes	chr14	intron	internal intron	NA	0.47	yes	down	0.20	no	0.87	no	down	1.5E-05	yes
MSTRG.9334.3 : Tmem173 : F1M391	inf	yes	up	7.6E-06	yes	chr18	intron	last intron	NA	0.97	no	down	0.92	no	1.18	no	up	2.5E-10	yes
MSTRG.12626.2 : Col5a1 : A0A0G2JX47	0.33	yes	down	4.5E-04	yes	chr3	intron	internal intron	NA	0.83	no	down	0.80	no	1.01	no	up	4.0E-01	no
MSTRG.16927.3 : Eml4 : A0A0G2K2Z0	0.79	yes	down	2.3E-04	yes	chr6	exon	internal exon	NA	1.10	no	up	0.83	no	1.03	no	up	6.8E-04	yes
ENSRNOT00000078123 : Ptcd3 : D3ZGM1	3.88	yes	up	4.1E-35	yes	chr4	intron	internal intron	NA	0.89	no	down	0.21	no	0.98	no	down	3.1E-01	no
MSTRG.14695.5 : Ptcd3 : D3ZGM1	3.88	yes	up	4.1E-35	yes	chr4	intron	internal intron	NA	1.06	no	up	0.86	no	0.98	no	down	3.1E-01	no
MSTRG.11953.16 : RT1-CE7 : D3ZLE6	inf	yes	up	7.3E-07	yes	chr20	exon	internal exon	NA	2.15	yes	up	0.12	no	1.22	yes	up	7.3E-16	yes
MSTRG.16317.2 : Cap1 : Q08163	0.21	yes	down	1.6E-07	yes	chr5	intron	internal intron	NA	0.94	no	down	0.90	no	0.94	no	down	1.0E-08	yes
MSTRG.17800.1 : Rcor1 : F1LXS5	2.75	yes	up	3.6E-04	yes	chr6	intron	internal intron	NA	0.97	no	down	0.97	no	1.11	no	up	2.6E-06	yes
ENSRNOT00000084595 : Sptbn1 : A0A0G2K8W9	5.72	yes	up	4.0E-04	yes	chr14	intron	internal intron	NA	1.03	no	up	0.19	no	0.87	no	down	1.1E-18	yes
ENSRNOT00000068386 : Tmem173 : F1M391	inf	yes	up	7.6E-06	yes	chr18	intron	last intron	NA	1.46	no	up	0.03	yes	1.18	no	up	2.5E-10	yes
ENSRNOT00000011960 : Dnajc11 : B1WBY5	2.59	yes	up	1.9E-14	yes	chr5	promoter	Intermediate	ICP	0.86	no	down	0.54	no	0.88	no	down	4.2E-12	yes
ENSRNOT00000054913 : Tbcd : F1M1D5	10.07	yes	up	4.7E-25	yes	chr10	intron	internal intron	NA	1.03	no	up	0.45	no	0.94	no	down	2.3E-10	yes
ENSRNOT00000011784 : Eci1 : P23965	6.21	yes	up	1.7E-05	yes	chr10	intron	internal intron	NA	0.91	no	down	0.37	no	0.85	no	down	2.2E-15	yes
MSTRG.20561.5 : Rftn1 : G3V7I2	3.70	yes	up	2.3E-04	yes	chr9	intron	internal intron	NA	1.27	no	up	0.48	no	1.12	no	up	1.5E-09	yes
MSTRG.10161.1 : Cdh13 : Q8R490	2.07	yes	up	3.3E-06	yes	chr19	promoter	Intermediate	LCP	2.85	yes	up	0.31	no	0.83	yes	down	2.3E-16	yes
ENSRNOT00000024992 : Trim21 : D4ACF2	0.20	yes	down	5.7E-10	yes	chr1	promoter	Distal	LCP	0.82	no	down	0.49	no	0.96	no	down	1.3E-04	yes
ENSRNOT00000011371 : Tax1bp1 : Q66HA4	8.48	yes	up	1.1E-06	yes	chr4	exon	last exon	NA	0.96	no	down	0.77	no	1.04	no	up	2.0E-04	yes
ENSRNOT00000091994 : Nf1x : F2Z3R4	0.58	yes	down	1.9E-06	yes	chr19	intron	internal intron	NA	1.18	no	up	0.11	no	0.77	yes	down	2.4E-14	yes
MSTRG.14930.5 : Grip2 : A0A0H2UHH8	2.18	yes	up	2.2E-05	yes	chr4	intron	internal intron	NA	0.74	no	down	0.07	no	1.01	no	up	5.2E-01	no
MSTRG.11813.1 : Wls : Q6P689	0.38	yes	down	6.5E-05	yes	chr2	exon	last exon	NA	1.03	no	up	0.50	no	1.11	no	up	1.3E-07	yes
ENSRNOT00000019554 : Anxa5 : P14668	3.34	yes	up	1.2E-08	yes	chr2	promoter	Distal	LCP	0.89	no	down	0.81	no	0.88	no	down	2.2E-14	yes
MSTRG.185.1 : Pdcd6 : G3V7W1	15.41	yes	up	1.1E-04	yes	chr1	promoter	Distal	LCP	1.05	no	up	0.46	no	0.95	no	down	4.9E-04	yes
MSTRG.11285.3 : Npr1 : P18910	0.37	yes	down	1.2E-04	yes	chr2	intron	internal intron	NA	0.49	yes	down	0.39	no	1.02	no	up	1.6E-02	yes
MSTRG.6691.1 : Pdgfra : G3V6A0	0.22	yes	down	1.6E-04	yes	chr14	intron	last intron	NA	2.29	yes	up	0.05	yes	1.02	no	up	4.1E-02	yes

ENSRNOT00000027264 : Cdc5l : O08837	4.40	yes	up	1.7E-04	yes	chr9	intron	internal intron	NA	1.04	no	up	0.57	no	1.05	no	up	3.5E-05	yes
MSTRG.16609.5 : Ece1 : Q6IN10	0.58	yes	down	9.3E-05	yes	chr5	intron	internal intron	NA	0.21	yes	down	0.45	no	1.14	no	up	8.3E-15	yes
MSTRG.21023.9 : Fn1 : F1LST1	0.11	yes	down	7.7E-11	yes	chr9	intron	internal intron	NA	0.17	yes	down	0.09	no	1.27	yes	up	4.1E-20	yes
MSTRG.1545.1 : Sbf2 : B5DEJ9	4.77	yes	up	8.9E-06	yes	chr1	intron	internal intron	NA	1.67	no	up	0.56	no	1.03	no	up	9.5E-02	no
MSTRG.11223.8 : Fdps : F1LND7	inf	yes	up	2.2E-05	yes	chr2	exon	last exon	NA	1.59	no	up	0.16	no	1.07	no	up	1.2E-03	yes
MSTRG.15096.8 : Cacna1c : F1MA84	4.75	yes	up	4.3E-04	yes	chr4	intron	internal intron	NA	1.18	no	up	0.19	no	0.97	no	down	2.7E-02	yes
ENSRNOT00000089199 : Tspan9 : D4AAV9	0.41	yes	down	1.4E-09	yes	chr4	exon	last exon	NA	0.90	no	down	0.58	no	0.96	no	down	2.4E-05	yes
ENSRNOT00000086352 : Col5a1 : A0A0G2JX47	0.33	yes	down	4.5E-04	yes	chr3	intron	internal intron	NA	0.97	no	down	0.76	no	1.01	no	up	4.0E-01	no
MSTRG.2768.8 : Usp7 : F1LM09	inf	yes	up	5.0E-04	yes	chr10	intron	internal intron	NA	6.20	yes	up	0.08	no	1.00	no	down	7.8E-01	no
ENSRNOT00000084563 : Tnc : A0A0G2K1L0	0	yes	down	7.1E-07	yes	chr5	intron	internal intron	NA	2.22	yes	up	0.36	no	1.49	yes	up	1.5E-12	yes
ENSRNOT00000036931 : Dnttip2 : D3ZHM7	5.36	yes	up	4.0E-04	yes	chr2	promoter	Proximal	ICP	1.07	no	up	0.60	no	1.05	no	up	1.0E-04	yes
ENSRNOT00000011114 : Crocc : D4AD05	2.69	yes	up	2.9E-05	yes	chr5	intron	internal intron	NA	1.10	no	up	0.22	no	0.87	no	down	9.6E-04	yes
MSTRG.3849.1 : Pnpo : O88794	2.62	yes	up	1.2E-06	yes	chr10	intron	internal intron	NA	1.47	no	up	0.10	no	0.99	no	down	6.7E-01	no
MSTRG.19777.2 : Sin3a : A0A0G2K3H5	0.53	yes	down	4.1E-06	yes	chr8	intron	last intron	NA	0.97	no	down	0.40	no	0.96	no	down	6.2E-05	yes
MSTRG.3481.1 : Tsr1 : D3ZEM8	45.59	yes	up	3.4E-04	yes	chr10	intron	internal intron	NA	0.73	no	down	0.13	no	1.00	no	down	9.7E-01	no
ENSRNOT00000092431 : Shank3 : A0A0U1RS13	3.23	yes	up	4.3E-05	yes	chr7	exon	first exon	NA	1.00	no	down	0.09	no	0.98	no	down	1.0E-02	yes
MSTRG.7803.1 : Mycbp2 : A0A1W2Q6I3	0.31	yes	down	9.2E-06	yes	chr15	intron	internal intron	NA	0.93	no	down	0.50	no	1.00	no	up	8.8E-01	no
MSTRG.18268.2 : Akap8 : Q63014	0.08	yes	down	2.7E-04	yes	chr7	intron	internal intron	NA	1.04	no	up	0.92	no	0.99	no	down	2.8E-01	no
ENSRNOT00000019772 : Fn1 : F1LST1	0.11	yes	down	7.7E-11	yes	chr9	intron	internal intron	NA	0.52	no	down	0.62	no	1.27	yes	up	4.1E-20	yes
MSTRG.21176.8 : Inpp5d : F1M981	0.08	yes	down	9.9E-05	yes	chr9	intron	internal intron	NA	0.75	no	down	0.22	no	1.08	no	up	3.1E-05	yes
ENSRNOT00000092157 : Mpp5 : B4F7E7	0.37	yes	down	1.0E-04	yes	chr6	exon	internal exon	NA	1.02	no	up	0.93	no	0.99	no	down	5.9E-01	no
MSTRG.7731.1 : Tsc22d1 : P62501	3.00	yes	up	3.0E-04	yes	chr15	promoter	Intermediate	ICP	2.16	yes	up	0.15	no	0.77	yes	down	5.9E-09	yes
MSTRG.16461.1 : Pum1 : D3Z8L5	0.44	yes	down	3.1E-04	yes	chr5	intron	internal intron	NA	1.15	no	up	0.43	no	1.03	no	up	7.0E-02	no
MSTRG.3781.3 : Luc7l3 : D3ZFB2	0.31	yes	down	4.3E-04	yes	chr10	intron	internal intron	NA	1.57	no	up	0.26	no	0.98	no	down	9.6E-04	yes
ENSRNOT00000044287 : Nr3c1 : P06536	0.27	yes	down	1.2E-04	yes	chr18	exon	first exon	NA	1.30	no	up	0.24	no	0.91	no	down	1.6E-10	yes
ENSRNOT00000086572 : Cfh : F1M983	0.20	yes	down	2.4E-04	yes	chr13	intron	internal intron	NA	1.49	no	up	0.19	no	1.20	no	up	2.7E-15	yes
ENSRNOT00000083500 : Ces1f : M0R7R1	0.26	yes	down	2.8E-08	yes	chr19	intron	internal intron	NA	1.02	no	up	0.23	no	0.86	no	down	3.2E-11	yes
MSTRG.8948.2 : Mtpap : D3ZPN5	1.99	yes	up	1.4E-05	yes	chr17	intron	internal intron	NA	1.40	no	up	0.07	no	1.03	no	up	1.2E-01	no
MSTRG.5399.1 : Eln : A0A0G2JST5	3.55	yes	up	3.0E-04	yes	chr12	intron	internal intron	NA	0.60	no	down	0.09	no	0.91	no	down	1.2E-01	no
MSTRG.16327.15 : Macf1 : A0A0G2K9T4	0.36	yes	down	1.3E-05	yes	chr5	exon	last exon	NA	1.22	no	up	0.06	no	0.97	no	down	9.0E-11	yes
MSTRG.19220.8 : Kmt2d : A0A0G2JVD6	6.53	yes	up	2.1E-09	yes	chr7	exon	last exon	NA	1.05	no	up	0.94	no	1.03	no	up	3.5E-02	yes
ENSRNOT00000065402 : Atp6v1h : A0A0G2K9J2	0.31	yes	down	9.6E-05	yes	chr5	intron	internal intron	NA	1.12	no	up	0.28	no	1.09	no	up	2.6E-06	yes
MSTRG.21023.8 : Fn1 : F1LST1	0.11	yes	down	7.7E-11	yes	chr9	intron	internal intron	NA	9.19	yes	up	0.42	no	1.27	yes	up	4.1E-20	yes
ENSRNOT00000093546 : Mycbp2 : A0A1W2Q6I3	0.31	yes	down	9.2E-06	yes	chr15	intron	internal intron	NA	1.00	no	down	0.48	no	1.00	no	up	8.8E-01	no
ENSRNOT00000089133 : Tbrg4 : Q5M9G9	0.25	yes	down	2.7E-04	yes	chr14	promoter	Distal	LCP	0.84	no	down	0.64	no	0.99	no	down	1.9E-01	no
ENSRNOT00000024707 : Sfxn1 : Q63965	0.56	yes	down	3.6E-04	yes	chr17	intron	internal intron	NA	1.20	no	up	0.78	no	1.22	yes	up	2.3E-08	yes
ENSRNOT00000004776 : Rufy3 : A0A0G2K6A9	2.37	yes	up	3.9E-04	yes	chr14	promoter	Distal	LCP	0.98	no	down	0.76	no	1.11	no	up	1.3E-05	yes
MSTRG.18268.9 : Akap8 : Q63014	0.08	yes	down	2.7E-04	yes	chr7	intron	internal intron	NA	1.42	no	up	0.38	no	0.99	no	down	2.8E-01	no
MSTRG.18670.4 : Ubr5 : H9KVE3	4.26	yes	up	2.2E-04	yes	chr7	intron	first intron	NA	0.70	no	down	0.20	no	0.94	no	down	3.0E-02	yes
ENSRNOT00000080778 : Qrich1 : F1M4M7	3.68	yes	up	3.4E-07	yes	chr8	exon	internal exon	NA	1.11	no	up	0.82	no	1.03	no	up	2.0E-01	no
MSTRG.4978.2 : Pi4ka : A0A140TAJ5	7.84	yes	up	1.4E-07	yes	chr11	intron	internal intron	NA	0.74	no	down	0.64	no	0.97	no	down	1.2E-03	yes
MSTRG.18268.31 : Akap8 : Q63014	0.08	yes	down	2.7E-04	yes	chr7	intron	internal intron	NA	0.61	no	down	0.49	no	0.99	no	down	2.8E-01	no
ENSRNOT00000029549 : Acap2 : Q5FVC7	0.15	yes	down	2.4E-05	yes	chr11	promoter	Intermediate	ICP	1.17	no	up	0.60	no	0.96	no	down	5.5E-04	yes
MSTRG.9111.4 : Bmi1 : B4F7B6	2.46	yes	up	2.3E-04	yes	chr17	exon	last exon	NA	1.02	no	up	0.08	no	0.98	no	down	3.6E-01	no
MSTRG.16653.2 : Crocc : D4AD05	2.69	yes	up	2.9E-05	yes	chr5	intron	internal intron	NA	1.05	no	up	0.81	no	0.87	no	down	9.6E-04	yes
ENSRNOT00000080379 : Ptpra : Q03348	0.34	yes	down	1.6E-04	yes	chr3	exon	internal exon	NA	0.98	no	down	0.84	no	0.89	no	down	6.4E-12	yes
ENSRNOT00000087143 : Ptdss1 : Q5PQL5	4.36	yes	up	3.7E-04	yes	chr7	exon	first exon	NA	1.25	no	up	0.01	yes	1.08	no	up	1.2E-04	yes
MSTRG.7731.2 : Tsc22d1 : P62501	3.00	yes	up	3.0E-04	yes	chr15	promoter	Intermediate	ICP	0.89	no	down	0.76	no	0.77	yes	down	5.9E-09	yes
MSTRG.659.4 : Cic : D4A853	4.32	yes	up	3.8E-07	yes	chr1	exon	internal exon	NA	0.96	no	down	0.70	no	1.06	no	up	4.3E-02	yes
MSTRG.16416.1 : Ak2 : A0A0G2JSG6	inf	yes	up	6.9E-05	yes	chr5	exon	first exon	NA	2.16	yes	up	0.22	no	0.98	no	down	1.2E-04	yes
ENSRNOT00000013581 : Arih1 : D3ZXL1	0.23	yes	down	8.6E-06	yes	chr8	intron	first intron	NA	1.23	no	up	0.12	no	0.93	no	down	1.4E-04	yes
ENSRNOT00000076676 : Parp4 : A0A096MJR6	1.23	yes	up	1.1E-06	yes	chr15	intron	last intron	NA	1.05	no	up	0.37	no	0.95	no	down	6.6E-05	yes
ENSRNOT00000086396 : Slc8a2 : A0A0G2JZK7	5.43	yes	up	2.7E-05	yes	chr1	exon	last exon	NA	1.45	no	up	0.26	no	0.78	yes	down	9.3E-16	yes
MSTRG.7223.1 : Zswim8 : A0A0G2K9R0	0.41	yes	down	1.3E-06	yes	chr15	intron	last intron	NA	0.95	no	down	0.53	no	0.92	no	down	1.2E-05	yes
MSTRG.21202.7 : Lrrfip1 : Q66HF9	6.49	yes	up	4.9E-10	yes	chr9	exon	last exon	NA	0.83	no	down	0.05	yes	1.15	no	up	2.1E-11	yes
MSTRG.8552.3 : Ddx46 : Q62780	0.56	yes	down	3.4E-04	yes	chr17	intron	internal intron	NA	0.96	no	down	0.81	no	0.98	no	down	1.3E-01	no
ENSRNOT00000031871 : Nt5dc3 : D3ZAI6	1.55	yes	up	4.2E-07	yes	chr7	intron	internal intron	NA	0.76	no	down	0.50	no	1.05	no	up	2.5E-05	yes
MSTRG.1463.2 : Stim1 : P84903	2.61	yes	up	1.3E-04	yes	chr1	exon	last exon	NA	1.14	no	up	0.12	no	0.92	no	down	3.4E-08	yes
MSTRG.21604.5 : Tbl1x : B2RZA6	0	yes	down	6.0E-05	yes	chrX	intron	internal intron	NA	1.11	no	up	0.47	no	1.12	no	up	7.8E-09	yes
MSTRG.18557.12 : Mon2 : D3ZCG3	0.07	yes	down	1.5E-08	yes	chr7	intron	internal intron	NA	1.00	no	down	0.45	no	1.05	no	up	7.1E-07	yes
MSTRG.21083.18 : Speg : Q63638	0.17	yes	down	2.6E-06	yes	chr9	intron	internal intron	NA	1.38	no	up	0.27	no	0.88	no	down	2.3E-07	yes
MSTRG.11091.4 : Gucy1a3 : P19686	0.26	yes	down	3.9E-06	yes	chr2	intron	internal intron	NA	0.43	yes	down	0.47	no	0.85	no	down	1.4E-12	yes
MSTRG.21039.2 : Tns1 : F1LN42	0.09	yes	down	2.0E-04	yes	chr9	intron	last intron	NA	1.02	no	up	0.97	no	0.86	no	down	7.8E-19	yes
MSTRG.13383.1 : Vps39 : E9PT04	inf	yes	up	4.3E-04	yes	chr3	intron	internal intron	NA	2.28	yes	up	0.02	yes	1.08	no	up	2.7E-04	yes
MSTRG.17200.1 : Adam17 : G3V711	0.27	yes	down	3.8E-13	yes	chr6	intron	internal intron	NA	0.89	no	down	0.90	no	1.00	no	down	9.2E-01	no
MSTRG.19329.1 : Atf7 : B0BMY0	0.38	yes	down	7.2E-06	yes	chr7	intron	internal intron	NA	1.38	no	up	0.49	no	1.00	no	down	9.7E-01	no
ENSRNOT00000020630 : Gle1 : Q4KLN4	0.16	yes	down	1.6E-04	yes	chr3	promoter	Proximal	LCP	1.20	no	up	0.06	no	0.95	no	down	1.3E-02	yes
MSTRG.3249.3 : Specc1 : A0A0G2K5D7	5.53	yes	up	1.9E-10	yes	chr10	exon	last exon	NA	1.27	no	up	0.61	no	0.93	no	down	4.4E-09	yes
MSTRG.14995.4 : Itpr1 : A0A0A0MY31	3.16	yes	up	1.1E-05	yes	chr4	intron	internal intron	NA	0.89	no	down	0.88	no	0.87	no	down	1.1E-14	yes
MSTRG.7951.1 : Mapk8 : A0A0G2KA63	0.22	yes	down	1.8E-04	yes	chr16	intron	internal intron	NA	0.95	no	down	0.78	no	1.06	no	up	2.4E-05	yes
ENSRNOT00000092982 : Gtf2i : Q5U2Y1	5.31	yes	up	4.9E-06	yes	chr12	promoter	Distal	LCP	0.95	no	down	0.21	no	0.95	no	down	1.5E-07	yes
ENSRNOT00000055839 : Crocc : D4AD05	2.69	yes	up	2.9E-05	yes	chr5	intron	internal intron	NA	1.03	no	up	0.81	no	0.87	no	down	9.6E-04	yes
MSTRG.5482.3 : Ncor2 : A0A0G2JU91	2.16	yes	up	1.6E-06	yes	chr12	intron	internal intron	NA	0.92	no	down	0.71	no	1.01	no	up	7.1E-01	no
MSTRG.7350.10 : Ktn1 : D4A4Z9	2.49	yes	up	1.2E-04	yes	chr15	intron	internal intron	NA	0.94	no	down	0.81	no	1.04	no	up	2.2E-11	yes
ENSRNOT00000083991 : Cmip : A0A0G2JYB9	0.58	yes	down	9.9E-05	yes	chr19	intron	internal intron	NA	0.58	no	down	0.09	no	0.89	no	down	2.1E-04	yes
ENSRNOT00000005770 : Pkn1 : Q63433	1.53	yes	up	6.1E-04	yes	chr19	intron	internal intron	NA	0.83	no	down	0.38	no	1.01	no	up	5.4E-01	no
ENSRNOT00000008256 : Cdk12 : A0A0G2K5U7	5.94	yes	up	1.1E-04	yes	chr10	exon	first exon	NA	0.64	no	down	0.16	no	0.97	no	down	5.5E-04	yes
MSTRG.12200.2 : Gstt1 : Q01579	0.44	yes	down	5.4E-04	yes	chr20	exon	last exon	NA	0.67	no	down	0.23	no	0.80	yes	down	1.4E-15	yes
ENSRNOT00000078203 : Ahsg : P24090	5.21	yes	up	1.0E-04	yes	chr11	exon	internal exon	NA	1.00	no	down	0.50	no	0.89	no	down	3.2E-06	yes
ENSRNOT00000045657 : Zc3h18 : Q6TQE1	0.03	yes	down	1.2E-11	yes	chr19	promoter	Intermediate	LCP	1.20	no	up	0.39	no	1.02	no	up	2.3E-01	no
ENSRNOT00000079535 : Myo5a : A0A0G2K9S4	1.92	yes	up	1.3E-04	yes	chr8	exon	internal exon	NA	1.08	no	up	0.77	no	1.12	no	up	2.4E-10	yes
ENSRNOT00000008888 : Nup210 : P11654	0.11	yes	down	6.6E-10	yes	chr4	intron	internal intron	NA	1.61	no	up	0.37	no	1.08	no	up	1.7E-07	yes
MSTRG.8552.7 : Ddx46 : Q62780	0.56	yes	down	3.4E-04	yes	chr17	intron	internal intron	NA	0.87	no	down	0.38	no	0.98	no	down	1.3E-01	no
MSTRG.13171.1 : Mtch2 : A0A0G2K459	2.54	yes	up	4.3E-04	yes	chr3	intron	internal intron	NA	0.99	no	down	0.90	no	1.00	no	up	8.3E-01	no
MSTRG.9889.2 : Nf1x : F2Z3R4	0.58	yes	down	1.9E-06	yes	chr19	intron	internal intron	NA	1.31	no	up	0.18	no	0.77	yes	down	2.4E-14	yes
ENSRNOT00000093439 : Mcc : F1LZS6	43.12	yes	up	1.3E-14	yes	chr1	exon	last exon	NA	1.02	no	up	0.48	no	0.85	no	down	3.7E-04	yes
MSTRG.10326.1 : Cast : F1LPH1	0.36	yes	down	1.6E-06	yes	chr2	intron	internal intron	NA	2.87	yes	up	0.05	yes	0.95	no	down	2.4E-10	yes
ENSRNOT00000047234 : Fbln1 : D3ZQ25	0.23	yes	down	7.2E-09	yes	chr7	intron	internal intron	NA	1.22	no	up	0.68	no	1.06	no	up	1.7E-09	yes
MSTRG.2768.5 : Usp7 : F1LM09	inf	yes	up	5.0E-04	yes	chr10	intron	internal intron	NA	0.40	yes	down	0.30	no	1.00	no	down	7.8E-01	no
MSTRG.17260.2 : Agr2 : D3ZIA7	0.34	yes	down	2.0E-08	yes	chr6	intron	first intron	NA	0.24	yes	down	0.06	no	0.85	no	down	2.5E-12	yes
ENSRNOT00000018717 : Vps18 : B5DFJ4	0.55	yes	down	3.0E-04	yes	chr3	exon	internal exon	NA	0.80	no	down	0.46	no	1.01	no	up	4.9E-01	no
MSTRG.21427.2 : Rbm3 : G3V6P6	5.50	yes	up	1.2E-04	yes	chrX	intron	first intron	NA	0.88	no	down	0.75	no	0.93	no	down	8.6E-07	yes
MSTRG.3949.5 : Dhx58 : D3ZD46	5.00	yes	up	2.1E-04	yes	chr10	exon	internal exon	NA	0.76	no	down	0.03	yes	1.22	yes	up	6.3E-08	yes
ENSRNOT00000035277 : Tns1 : F1LN42	0.09	yes	down	2.0E-04	yes	chr9	intron	last intron	NA	1.29	no	up	0.75	no	0.86	no	down	7.8E-19	yes
MSTRG.19219.5 : Fkbp11 : G3V7V5	inf	yes	up	4.7E-06	yes	chr7	intron	internal intron	NA	1.48	no	up	0.24	no	1.06	no	up	1.6E-07	yes
ENSRNOT00000068440 : Cdyl2 : F1LT42	0.32	yes	down	1.4E-04	yes	chr19	intron	internal intron	NA	1.11	no	up	0.24	no	1.45	yes	up	9.9E-16	yes
MSTRG.17673.3 : Ppp4r3a : D4ABC4	3.37	yes	up	4.5E-04	yes	chr6	intron	internal intron	NA	1.79	no	up	0.27	no	1.03	no	up	5.4E-03	yes
ENSRNOT00000071595 : Arhgap44 : A0A0H2UHC07.94		yes	up	4.2E-19	yes	chr10	intron	internal intron	NA	0.96	no	down	0.69	no	1.04	no	up	6.6E-02	no
MSTRG.1683.7 : Il4r : Q63257	9.80	yes	up	2.9E-08	yes	chr1	intron	internal intron	NA	0.54	no	down	0.17	no	1.11	no	up	2.1E-05	yes
ENSRNOT00000084322 : Prom1 : Q9JI49	8.32	yes	up	2.4E-09	yes	chr14	intron	internal intron	NA	1.00	no	down	0.14	no	0.87	no	down	1.5E-05	yes

MSTRG.2010.1 : Ppp6r3 : D3ZBT9	3.21	yes	up	8.1E-07	yes	chr1	intron	internal intron	NA	1.13	no	up	0.44	no	0.98	no	down	7.0E-03	yes
MSTRG.549.2 : Zc3h4 : D3ZVW3	2.19	yes	up	2.6E-04	yes	chr1	intron	internal intron	NA	1.21	no	up	0.79	no	0.98	no	down	3.2E-01	no
ENSRNOT00000020543 : Tnpo1 : F1LQP9	24.87	yes	up	1.6E-05	yes	chr2	exon	internal exon	NA	0.89	no	down	0.91	no	0.98	no	down	5.8E-02	no
ENSRNOT00000074718 : Gas7 : M0R4R4	4.60	yes	up	4.0E-11	yes	chr10	intron	first intron	NA	0.90	no	down	0.71	no	1.22	yes	up	2.5E-15	yes
MSTRG.19173.3 : Ano6 : A0A0G2K1M7	3.87	yes	up	9.8E-09	yes	chr7	intron	internal intron	NA	0.71	no	down	0.39	no	0.91	no	down	2.1E-09	yes
ENSRNOT00000019309 : Arhgap17 : D4AAV2	0.83	yes	down	2.6E-07	yes	chr1	intron	internal intron	NA	1.08	no	up	0.78	no	1.01	no	up	3.1E-01	no
MSTRG.13466.1 : Myef2 : A0A0G2K402	2.63	yes	up	2.7E-04	yes	chr3	promoter	Intermediate	LCP	2.59	yes	up	0.17	no	0.93	no	down	4.5E-09	yes
MSTRG.20831.1 : Tgfbrap1 : D3ZXT8	0.12	yes	down	2.5E-04	yes	chr9	intron	internal intron	NA	0.50	yes	down	0.37	no	0.98	no	down	2.9E-01	no
ENSRNOT00000026373 : Gnao1 : P59215	0.45	yes	down	1.5E-04	yes	chr19	exon	internal exon	NA	0.81	no	down	0.10	no	0.81	yes	down	3.6E-12	yes
ENSRNOT00000084020 : Med12l : A0A0G2JV69	6.48	yes	up	1.3E-08	yes	chr2	promoter	Distal	LCP	0.95	no	down	0.02	yes	0.61	yes	down	2.2E-04	yes
MSTRG.4937.1 : Gp1bb : Q9JJM7	0	yes	down	2.2E-04	yes	chr11	intron	internal intron	NA	0.62	no	down	0.21	no	0.93	no	down	7.7E-08	yes
MSTRG.599.1 : Bloc1s3 : D4A3V6	3.64	yes	up	1.9E-05	yes	chr1	exon	first exon	NA	1.41	no	up	0.40	no	0.73	yes	down	2.9E-09	yes
ENSRNOT00000020044 : Nr2f1 : A0A1W2Q6N8	1.84	yes	up	2.6E-07	yes	chr2	intron	last intron	NA	1.14	no	up	0.41	no	1.00	no	up	7.8E-01	no
MSTRG.19119.2 : Gramd4 : F1LYJ8	0.57	yes	down	9.4E-05	yes	chr7	intron	internal intron	NA	2.49	yes	up	0.09	no	1.32	yes	up	2.0E-09	yes
MSTRG.13405.3 : Tmem62 : D3ZIW4	0.44	yes	down	9.9E-05	yes	chr3	exon	last exon	NA	1.45	no	up	0.44	no	1.03	no	up	1.0E-01	no
ENSRNOT00000090026 : Gls : A0A0G2KAN7	0.05	yes	down	1.5E-13	yes	chr9	intron	first intron	NA	0.99	no	down	0.99	no	1.09	no	up	8.3E-03	yes
MSTRG.18268.30 : Akap8 : Q63014	0.08	yes	down	2.7E-04	yes	chr7	intron	internal intron	NA	0.68	no	down	0.44	no	0.99	no	down	2.8E-01	no
MSTRG.6191.3 : Ddr2 : B1WC09	0.49	yes	down	3.3E-05	yes	chr13	exon	internal exon	NA	1.88	no	up	0.48	no	0.95	no	down	3.2E-01	no
ENSRNOT00000080676 : Ywhaz : P63102	3.68	yes	up	2.7E-04	yes	chr7	intron	last intron	NA	1.18	no	up	0.47	no	0.87	no	down	1.2E-13	yes
ENSRNOT00000091270 : Tnrc6b : A0A0G2K6R0	2.14	yes	up	4.3E-13	yes	chr7	exon	last exon	NA	1.34	no	up	0.02	yes	1.01	no	up	7.4E-01	no
ENSRNOT00000077612 : Gsk3b : A0A0G2JSH4	2.36	yes	up	1.7E-04	yes	chr11	intron	internal intron	NA	1.01	no	up	0.85	no	0.98	no	down	1.1E-02	yes
MSTRG.10517.1 : Erbin : M0R9T2	3.86	yes	up	2.1E-05	yes	chr2	intron	last intron	NA	0.78	no	down	0.52	no	0.94	no	down	3.9E-05	yes
ENSRNOT00000076859 : Fyn : Q62844	0.38	yes	down	2.4E-05	yes	chr20	exon	last exon	NA	1.25	no	up	0.09	no	1.01	no	up	5.2E-01	no
ENSRNOT00000019057 : Lhfp : Q5BJS2	2.11	yes	up	5.8E-06	yes	chr2	intron	internal intron	NA	1.04	no	up	0.70	no	0.95	no	down	4.1E-02	yes
ENSRNOT00000024196 : Gpld1 : G3V8B1	6.56	yes	up	2.9E-12	yes	chr17	intron	internal intron	NA	0.88	no	down	0.34	no	1.02	no	up	3.6E-01	no
MSTRG.8713.3 : Sirt5 : Q68FX9	6.16	yes	up	1.6E-04	yes	chr17	intron	internal intron	NA	0.93	no	down	0.61	no	1.01	no	up	5.1E-01	no
MSTRG.8278.29 : Rbpms : F2Z3S5	0.02	yes	down	2.6E-05	yes	chr16	promoter	Proximal	ICP	1.01	no	up	0.09	no	1.06	no	up	4.9E-05	yes
MSTRG.19496.1 : Cnn1 : Q08290	3.38	yes	up	1.8E-04	yes	chr8	intron	internal intron	NA	0.89	no	down	0.52	no	1.04	no	up	1.3E-06	yes
MSTRG.17688.1 : Golga5 : G3V6Z7	4.67	yes	up	4.2E-17	yes	chr6	intron	last intron	NA	0.89	no	down	0.45	no	1.07	no	up	2.3E-12	yes
MSTRG.14349.2 : Cped1 : A0A0G2QC10	1.61	yes	up	4.5E-04	yes	chr4	intron	internal intron	NA	1.88	no	up	0.42	no	1.07	no	up	1.5E-03	yes
ENSRNOT00000077334 : Med12l : A0A0G2JV69	6.48	yes	up	1.3E-08	yes	chr2	promoter	Distal	LCP	0.99	no	down	0.45	no	0.61	yes	down	2.2E-04	yes
MSTRG.19472.6 : Dnm2 : A0A0A0MY48	0.11	yes	down	8.9E-11	yes	chr8	intron	first intron	NA	1.07	no	up	0.21	no	1.05	no	up	2.0E-08	yes
MSTRG.15479.4 : Prex2 : A0A0G2KA11	5.57	yes	up	1.6E-04	yes	chr5	intron	internal intron	NA	1.59	no	up	0.48	no	1.02	no	up	2.3E-01	no
MSTRG.11953.28 : RT1-CE7 : D3ZLE6	inf	yes	up	7.3E-07	yes	chr20	exon	internal exon	NA	0.99	no	down	0.97	no	1.22	yes	up	7.3E-16	yes
MSTRG.11265.8 : Ubap2l : E9PTR4	6.62	yes	up	4.0E-04	yes	chr2	intron	internal intron	NA	0.82	no	down	0.44	no	1.13	no	up	3.8E-12	yes
ENSRNOT00000058887 : Dock5 : F1LVA9	0.40	yes	down	5.4E-05	yes	chr15	intron	internal intron	NA	1.26	no	up	0.11	no	1.14	no	up	7.1E-05	yes
ENSRNOT00000084535 : Cpd : A0A0G2JY30	0.15	yes	down	4.3E-04	yes	chr10	intron	internal intron	NA	1.00	no	down	0.42	no	1.13	no	up	1.1E-08	yes
ENSRNOT00000067318 : Arhgap1 : D4A6C5	3.73	yes	up	1.8E-11	yes	chr3	intron	internal intron	NA	0.42	yes	down	0.35	no	1.04	no	up	1.7E-04	yes
MSTRG.17738.3 : Vrk1 : Q6AYA2	0.22	yes	down	2.5E-07	yes	chr6	intron	internal intron	NA	1.10	no	up	0.82	no	1.01	no	up	2.7E-01	no
ENSRNOT00000080888 : Kif1b : A0A0G2KA12	3.65	yes	up	7.4E-05	yes	chr5	intron	internal intron	NA	0.80	no	down	0.45	no	1.05	no	up	6.5E-02	no
MSTRG.3353.1 : Zbtb4 : D4A8X0	inf	yes	up	2.4E-05	yes	chr10	intron	last intron	NA	1.61	no	up	0.40	no	0.97	no	down	6.9E-02	no
ENSRNOT00000022359 : Lpcat2 : F1LN03	0.55	yes	down	2.2E-04	yes	chr19	exon	internal exon	NA	1.29	no	up	0.05	yes	1.16	no	up	3.1E-07	yes
MSTRG.540.4 : Slc8a2 : A0A0G2JZK7	5.43	yes	up	2.7E-05	yes	chr1	exon	last exon	NA	1.12	no	up	0.64	no	0.78	yes	down	9.3E-16	yes
MSTRG.22091.1 : Arhgef6 : Q5XXR3	0.21	yes	down	2.1E-04	yes	chrX	intron	internal intron	NA	0.98	no	down	0.96	no	0.93	no	down	2.3E-08	yes
MSTRG.2579.1 : Sfxn3 : Q6P6T0	0.39	yes	down	7.8E-05	yes	chr1	intron	internal intron	NA	2.21	yes	up	0.05	yes	1.12	no	up	6.4E-07	yes
ENSRNOT00000091318 : Kcnma1 : A0A0G2K104	4.06	yes	up	2.6E-13	yes	chr15	intron	internal intron	NA	1.01	no	up	0.66	no	0.89	no	down	7.4E-06	yes
MSTRG.1783.6 : Tgfb1i1 : Q99PD6	3.08	yes	up	7.2E-05	yes	chr1	intron	internal intron	NA	0.80	no	down	0.63	no	1.06	no	up	1.6E-04	yes
ENSRNOT00000001198 : Kctd7 : B1WC97	1.68	yes	up	8.9E-05	yes	chr12	intron	internal intron	NA	0.89	no	down	0.43	no	1.10	no	up	2.1E-04	yes
MSTRG.6685.3 : Exoc1 : A0A0G2K2V5	inf	yes	up	6.1E-05	yes	chr14	intron	first intron	NA	0.68	no	down	0.42	no	0.90	no	down	2.2E-04	yes
MSTRG.8552.5 : Ddx46 : Q62780	0.56	yes	down	3.4E-04	yes	chr17	intron	internal intron	NA	0.77	no	down	0.10	no	0.98	no	down	1.3E-01	no
MSTRG.21008.3 : Myl1 : P02600	2.29	yes	up	4.3E-05	yes	chr9	promoter	Distal	LCP	0.80	no	down	0.38	no	1.33	yes	up	6.1E-13	yes
ENSRNOT00000079917 : Prex2 : A0A0G2KA11	5.57	yes	up	1.6E-04	yes	chr5	intron	internal intron	NA	1.00	no	down	0.98	no	1.02	no	up	2.3E-01	no
ENSRNOT00000037517 : C1qbp : O35796	19.02	yes	up	1.7E-11	yes	chr10	intron	internal intron	NA	0.94	no	down	0.65	no	0.95	no	down	2.1E-05	yes
MSTRG.21122.1 : Agfg1 : F1M9N7	3.59	yes	up	1.8E-08	yes	chr9	exon	internal exon	NA	1.74	no	up	0.47	no	1.16	no	up	6.0E-06	yes
ENSRNOT00000001347 : Tecpr1 : Q3ZBA0	0.43	yes	down	3.1E-04	yes	chr12	promoter	Intermediate	LCP	1.06	no	up	0.48	no	1.17	no	up	7.7E-08	yes
ENSRNOT00000008698 : Thnsl2 : Q5M7T9	3.56	yes	up	3.5E-12	yes	chr4	intron	internal intron	NA	1.02	no	up	0.84	no	0.97	no	down	4.8E-01	no
ENSRNOT00000021475 : Dcp1a : D4AE80	inf	yes	up	1.3E-05	yes	chr16	exon	last exon	NA	0.95	no	down	0.44	no	0.95	no	down	3.8E-02	yes
MSTRG.18264.2 : Notch3 : F1LQX7	0.61	yes	down	2.1E-04	yes	chr7	exon	internal exon	NA	1.06	no	up	0.89	no	0.96	no	down	9.5E-04	yes
ENSRNOT00000001743 : S100b : P04631	0.27	yes	down	2.6E-04	yes	chr20	exon	last exon	NA	0.83	no	down	0.71	no	0.76	yes	down	2.0E-16	yes
ENSRNOT00000057798 : Specc1 : A0A0G2K5D7	5.53	yes	up	1.9E-10	yes	chr10	exon	last exon	NA	0.58	no	down	0.16	no	0.93	no	down	4.4E-09	yes
MSTRG.19777.9 : Sin3a : A0A0G2K3H5	0.53	yes	down	4.1E-06	yes	chr8	intron	last intron	NA	2.72	yes	up	0.07	no	0.96	no	down	6.2E-05	yes
MSTRG.6713.6 : Fryl : D3ZQY4	2.76	yes	up	1.7E-04	yes	chr14	intron	internal intron	NA	2.21	yes	up	0.09	no	1.00	no	down	4.6E-01	no
MSTRG.18880.3 : Plec : F7F9U6	0.33	yes	down	4.2E-06	yes	chr7	exon	last exon	NA	1.02	no	up	0.96	no	0.90	no	down	2.5E-03	yes
ENSRNOT00000079138 : Lama2 : F1M614	0.44	yes	down	4.4E-04	yes	chr1	intron	internal intron	NA	1.03	no	up	0.73	no	1.06	no	up	1.3E-07	yes
MSTRG.21168.3 : Gigyf2 : A0A096MJI4	0.59	yes	down	1.9E-05	yes	chr9	intron	internal intron	NA	1.54	no	up	0.06	no	1.31	yes	up	2.3E-10	yes
MSTRG.20963.4 : Nbeal1 : F1M6V0	4.79	yes	up	6.8E-08	yes	chr9	intron	internal intron	NA	1.32	no	up	0.75	no	1.00	no	up	5.1E-01	no
MSTRG.98.1 : Bclaf1 : B1WC16	0.67	yes	down	1.3E-07	yes	chr1	intron	internal intron	NA	0.78	no	down	0.75	no	1.03	no	up	6.8E-02	no
ENSRNOT00000064263 : Limch1 : F1M392	1.09	no	up	3.3E-04	yes	chr14	intron	last intron	NA	0.99	no	down	0.99	no	0.85	no	down	1.1E-13	yes
ENSRNOT00000001605 : Pwp2 : D3ZV54	0.63	yes	down	1.1E-04	yes	chr20	promoter	Intermediate	ICP	0.99	no	down	0.87	no	1.05	no	up	1.3E-02	yes
MSTRG.3353.2 : Zbtb4 : D4A8X0	inf	yes	up	2.4E-05	yes	chr10	intron	last intron	NA	1.28	no	up	0.61	no	0.97	no	down	6.9E-02	no
ENSRNOT00000084496 : Inpp5k : A0A0G2K2Z2	8.85	yes	up	4.8E-08	yes	chr10	promoter	Distal	ICP	2.82	yes	up	0.27	no	0.94	no	down	3.3E-03	yes
MSTRG.21604.4 : Tbl1x : B2RZA6	0	yes	down	6.0E-05	yes	chrX	intron	internal intron	NA	0.78	no	down	0.50	no	1.12	no	up	7.8E-09	yes
MSTRG.2768.4 : Usp7 : F1LM09	inf	yes	up	5.0E-04	yes	chr10	intron	internal intron	NA	1.28	no	up	0.20	no	1.00	no	down	7.8E-01	no
MSTRG.5280.8 : Taf6 : Q498R0	0.74	yes	down	2.2E-06	yes	chr12	intron	internal intron	NA	1.09	no	up	0.91	no	0.91	no	down	1.8E-03	yes
ENSRNOT00000080351 : Cenpb : A0A0G2KAW9	1.54	yes	up	2.1E-56	yes	chr3	exon	first exon	NA	0.28	yes	down	0.14	no	1.09	no	up	2.5E-02	yes
ENSRNOT00000001305 : Camsap3 : A0A0G2K5C0	0.05	yes	down	5.3E-04	yes	chr12	intron	first intron	NA	0.88	no	down	0.14	no	0.86	no	down	3.4E-07	yes
MSTRG.276.2 : Arid1b : F1LNP1	1.53	yes	up	6.5E-08	yes	chr1	promoter	Proximal	LCP	1.23	no	up	0.40	no	0.98	no	down	1.8E-01	no
ENSRNOT00000093122 : Tie1 : B5DFD6	1.35	yes	up	2.1E-04	yes	chr5	intron	first intron	NA	0.73	no	down	0.28	no	0.76	yes	down	4.3E-08	yes
MSTRG.5778.1 : Pign : E9PTA5	0.23	yes	down	2.2E-04	yes	chr13	intron	internal intron	NA	0.62	no	down	0.37	no	1.05	no	up	3.6E-03	yes
MSTRG.22089.2 : Htatsf1 : D4A997	6.87	yes	up	2.7E-04	yes	chrX	promoter	Intermediate	HCP	1.02	no	up	0.94	no	0.95	no	down	9.0E-07	yes
ENSRNOT00000078851 : Alpl : P08289	0.52	yes	down	2.4E-04	yes	chr5	promoter	Intermediate	LCP	1.00	no	down	0.97	no	1.00	no	down	6.2E-01	no
MSTRG.9568.5 : Nedd4l : F1LRN8	2.03	yes	up	3.6E-05	yes	chr18	intron	internal intron	NA	0.62	no	down	0.45	no	0.95	no	down	3.9E-02	yes
MSTRG.18072.5 : Dgka : P51556	0.72	yes	down	1.5E-04	yes	chr7	exon	internal exon	NA	0.83	no	down	0.66	no	0.95	no	down	8.8E-04	yes
ENSRNOT00000081637 : Syne1 : Q8VHJ9	0.04	yes	down	1.5E-19	yes	chr1	exon	first exon	NA	0.97	no	down	0.36	no	0.89	no	down	8.9E-14	yes
ENSRNOT00000085273 : Phf10 : A0A0G2K3M7	0.43	yes	down	3.7E-05	yes	chr1	intron	internal intron	NA	1.07	no	up	0.18	no	1.03	no	up	2.1E-01	no
ENSRNOT00000085901 : Aamp : B0K024	0.09	yes	down	2.6E-04	yes	chr9	promoter	Intermediate	LCP	0.72	no	down	0.02	yes	1.06	no	up	1.5E-05	yes
MSTRG.13544.1 : Ptpra : Q03348	0.34	yes	down	1.6E-04	yes	chr3	exon	internal exon	NA	0.60	no	down	0.50	no	0.89	no	down	6.4E-12	yes
MSTRG.13080.2 : Lnpk : A0JN29	3.42	yes	up	1.2E-11	yes	chr3	intron	internal intron	NA	1.11	no	up	0.59	no	0.96	no	down	2.6E-04	yes
ENSRNOT00000077961 : ST7 : A0A0G2KBB5	4.88	yes	up	1.5E-16	yes	chr4	promoter	Distal	LCP	0.87	no	down	0.48	no	1.14	no	up	1.4E-03	yes
ENSRNOT00000014063 : Pus7 : D4ADZ9	0.24	yes	down	4.9E-09	yes	chr4	intron	internal intron	NA	1.08	no	up	0.64	no	1.01	no	up	6.7E-01	no
MSTRG.6572.2 : Bmp2k : Q3SYQ0	11.81	yes	up	5.6E-07	yes	chr14	intron	internal intron	NA	1.19	no	up	0.47	no	1.19	no	up	6.4E-09	yes
MSTRG.13954.5 : Znfx1 : F1LMA9	20.79	yes	up	3.0E-04	yes	chr3	intron	internal intron	NA	0.82	no	down	0.65	no	0.92	no	down	1.0E-03	yes
MSTRG.2862.1 : Eci1 : P23965	6.21	yes	up	1.7E-05	yes	chr10	intron	internal intron	NA	0.98	no	down	0.36	no	0.85	no	down	2.2E-15	yes
ENSRNOT00000088042 : Fbln1 : B1WC21	0.23	yes	down	7.2E-09	yes	chr7	intron	internal intron	NA	0.60	no	down	0.20	no	1.15	no	up	5.3E-06	yes
MSTRG.18206.2 : Cirbp : P60825	0.73	yes	down	1.3E-04	yes	chr7	exon	first exon	NA	0.54	no	down	0.06	no	0.85	no	down	3.4E-16	yes
MSTRG.18880.2 : Plec : F7F9U6	0.33	yes	down	4.2E-06	yes	chr7	exon	last exon	NA	1.39	no	up	0.52	no	0.90	no	down	2.5E-03	yes
ENSRNOT00000084391 : Sgpl1 : Q8CHN6	0.20	yes	down	6.8E-07	yes	chr20	intron	internal intron	NA	0.67	no	down	0.72	no	1.12	no	up	5.6E-16	yes
MSTRG.1078.1 : Prmt3 : O70467	1.85	yes	up	1.1E-04	yes	chr1	intron	internal intron	NA	0.88	no	down	0.59	no	1.06	no	up	2.2E-04	yes
MSTRG.12922.1 : Zeb2 : A0A0G2K8T6	0.34	yes	down	8.0E-09	yes	chr3	intron	internal intron	NA	2.01	yes	up	0.08	no	1.01	no	up	2.4E-01	no
ENSRNOT00000041215 : Eml2 : Q6P6T4	0.17	yes	down	4.6E-06	yes	chr1	intron	internal intron	NA	1.06	no	up	0.65	no	0.81	yes	down	6.9E-13	yes

MSTRG.22064.5 : Arhgap4 : A0A0G2JVF0	0.34	yes	down	1.4E-05	yes	chrX	intron	internal intron	NA	1.17	no	up	0.47	no	1.07	no	up	7.1E-04	yes
ENSRNOT00000064774 : Prex2 : A0A0G2KA11	5.57	yes	up	1.6E-04	yes	chr5	intron	internal intron	NA	1.01	no	up	0.52	no	1.02	no	up	2.3E-01	no
MSTRG.12280.2 : Herc4 : Q5PQN1	2.70	yes	up	6.2E-07	yes	chr20	intron	internal intron	NA	1.05	no	up	0.79	no	1.00	no	down	9.1E-01	no
MSTRG.3296.2 : Gas7 : M0R4R4	4.60	yes	up	4.0E-11	yes	chr10	intron	first intron	NA	0.72	no	down	0.56	no	1.22	yes	up	2.5E-15	yes
ENSRNOT00000078255 : ST7 : A0A0G2KBB5	4.88	yes	up	1.5E-16	yes	chr4	promoter	Distal	LCP	0.98	no	down	0.40	no	1.14	no	up	1.4E-03	yes
MSTRG.21446.1 : Tfe3 : D3ZAW6	0.07	yes	down	2.9E-11	yes	chrX	exon	last exon	NA	0.54	no	down	0.07	no	1.04	no	up	1.6E-03	yes
MSTRG.9198.1 : Osbpl1a : A0A0G2K327	0.28	yes	down	2.2E-05	yes	chr18	intron	internal intron	NA	1.15	no	up	0.34	no	0.94	no	down	2.8E-02	yes
ENSRNOT00000080185 : Fdps : F1LND7	inf	yes	up	2.2E-05	yes	chr2	exon	last exon	NA	1.43	no	up	0.43	no	1.07	no	up	1.2E-03	yes
ENSRNOT00000001158 : Arhgef6 : Q5XXR3	0.21	yes	down	2.1E-04	yes	chrX	intron	internal intron	NA	1.59	no	up	0.31	no	0.93	no	down	2.3E-08	yes
ENSRNOT00000085727 : Myo1e : A0A0G2K9E8	3.15	yes	up	9.4E-05	yes	chr8	intron	first intron	NA	1.00	no	down	0.48	no	0.76	yes	down	2.6E-20	yes
ENSRNOT00000092552 : Lamp1 : P14562	0.27	yes	down	5.9E-08	yes	chr16	promoter	Distal	LCP	1.00	no	down	0.09	no	1.10	no	up	5.4E-06	yes
ENSRNOT00000042082 : Ube2f : Q5U203	7.18	yes	up	7.2E-05	yes	chr9	intron	internal intron	NA	1.09	no	up	0.65	no	1.01	no	up	5.6E-01	no
ENSRNOT00000005144 : Slc25a11 : G3V6H5	0.46	yes	down	2.3E-04	yes	chr10	intron	internal intron	NA	0.99	no	down	0.94	no	0.93	no	down	1.9E-12	yes
ENSRNOT00000091389 : Fmo2 : G3V6F6	2.66	yes	up	2.1E-11	yes	chr13	intron	internal intron	NA	0.85	no	down	0.67	no	1.21	yes	up	2.2E-15	yes
MSTRG.12770.2 : Hspa5 : P06761	0.56	yes	down	2.4E-04	yes	chr3	intron	internal intron	NA	0.62	no	down	0.55	no	1.06	no	up	6.4E-09	yes
ENSRNOT00000077048 : Becn1 : Q91XJ1	0.15	yes	down	1.2E-04	yes	chr10	exon	first exon	NA	1.45	no	up	0.02	yes	0.97	no	down	9.2E-02	no
ENSRNOT00000085191 : Shank3 : A0A0U1RS13	3.23	yes	up	4.3E-05	yes	chr7	exon	first exon	NA	1.00	no	down	0.09	no	0.98	no	down	1.0E-02	yes
MSTRG.9900.2 : Rnaseh2a : Q5U209	14.16	yes	up	1.1E-05	yes	chr19	intron	last intron	NA	0.86	no	down	0.21	no	1.08	no	up	2.6E-05	yes
ENSRNOT00000001215 : Gusb : F1LQQ8	29.04	yes	up	6.5E-05	yes	chr12	intron	internal intron	NA	0.99	no	down	0.96	no	0.97	no	down	1.8E-03	yes
MSTRG.1265.1 : Zfp592 : D3ZJG8	0.22	yes	down	8.1E-08	yes	chr1	exon	internal exon	NA	1.08	no	up	0.36	no	1.06	no	up	1.8E-05	yes
ENSRNOT00000021689 : Fam129b : B4F7E8	25.76	yes	up	9.8E-05	yes	chr3	intron	internal intron	NA	0.97	no	down	0.93	no	0.90	no	down	7.0E-10	yes
MSTRG.17586.5 : Ylpm1 : A0A0G2K678	0.14	yes	down	2.3E-07	yes	chr6	intron	internal intron	NA	1.76	no	up	0.26	no	1.02	no	up	1.8E-03	yes
MSTRG.15065.1 : Plxnd1 : D4AA77	0.14	yes	down	1.5E-08	yes	chr4	intron	internal intron	NA	0.88	no	down	0.43	no	0.94	no	down	1.4E-01	no
MSTRG.9803.1 : Rbl2 : G3V7P7	0.41	yes	down	2.1E-05	yes	chr19	intron	internal intron	NA	0.83	no	down	0.53	no	0.89	no	down	1.5E-05	yes
MSTRG.16821.1 : Prdm16 : M0RDL0	0.37	yes	down	2.7E-04	yes	chr5	exon	internal exon	NA	0.97	no	down	0.92	no	0.83	yes	down	1.1E-06	yes
ENSRNOT00000018167 : Pex14 : Q642G4	2.11	yes	up	2.4E-04	yes	chr5	intron	internal intron	NA	0.86	no	down	0.16	no	1.04	no	up	2.1E-03	yes
ENSRNOT00000093260 : Mycbp2 : A0A1W2Q6I3	0.31	yes	down	9.2E-06	yes	chr15	intron	internal intron	NA	0.97	no	down	0.34	no	1.00	no	up	8.8E-01	no
ENSRNOT00000016613 : Casp8 : Q9JHX4	11.45	yes	up	1.6E-14	yes	chr9	intron	first intron	NA	1.42	no	up	0.28	no	1.03	no	up	4.4E-02	yes
ENSRNOT00000018788 : Klhl13 : F1LM44	0.22	yes	down	7.1E-07	yes	chrX	intron	last intron	NA	1.12	no	up	0.73	no	1.03	no	up	6.8E-03	yes
MSTRG.8514.8 : Zcchc6 : D3ZKR9	0.14	yes	down	1.2E-08	yes	chr17	exon	internal exon	NA	1.59	no	up	0.11	no	1.12	no	up	2.3E-06	yes
ENSRNOT00000024493 : Tpm1 : A0A0G2JSQ4	9.41	yes	up	5.1E-14	yes	chr8	intron	internal intron	NA	0.72	no	down	0.44	no	0.81	yes	down	2.5E-10	yes
MSTRG.5723.4 : Ttc28 : D3ZXP1	4.41	yes	up	1.9E-04	yes	chr12	promoter	Intermediate	LCP	1.19	no	up	0.52	no	0.86	no	down	6.9E-05	yes
ENSRNOT00000018843 : Arhgap26 : A0A0G2K5D5	0.31	yes	down	2.9E-05	yes	chr18	intron	internal intron	NA	1.03	no	up	0.48	no	1.14	no	up	8.4E-08	yes
MSTRG.6746.2 : Pds5a : A4L9P7	1.26	yes	up	1.3E-13	yes	chr14	exon	last exon	NA	1.11	no	up	0.47	no	1.01	no	up	2.7E-01	no
ENSRNOT00000036373 : Dgka : P51556	0.72	yes	down	1.5E-04	yes	chr7	exon	internal exon	NA	1.21	no	up	0.40	no	0.95	no	down	8.8E-04	yes
MSTRG.2160.2 : Rasgrp2 : P0C643	0.26	yes	down	2.6E-04	yes	chr1	exon	internal exon	NA	1.00	no	down	1.00	no	1.04	no	up	7.0E-02	no
MSTRG.20817.3 : Map4k4 : A0A0G2K7W4	0.36	yes	down	7.1E-05	yes	chr9	intron	internal intron	NA	1.12	no	up	0.33	no	1.10	no	up	7.1E-06	yes
MSTRG.4525.7 : Mx1 : Q499S4	3.65	yes	up	1.1E-13	yes	chr11	intron	internal intron	NA	0.28	yes	down	0.41	no	1.36	yes	up	1.2E-13	yes
ENSRNOT00000004829 : Arhgap44 : A0A0H2UHC	7.94	yes	up	4.2E-19	yes	chr10	intron	internal intron	NA	0.91	no	down	0.01	yes	1.04	no	up	6.6E-02	no
MSTRG.3225.1 : Shmt1 : Q6TXG7	0.30	yes	down	2.1E-05	yes	chr10	intron	internal intron	NA	1.11	no	up	0.66	no	0.90	no	down	1.3E-08	yes
ENSRNOT00000093205 : Timm50 : D3ZJX5	0.54	yes	down	3.3E-07	yes	chr1	exon	last exon	NA	1.06	no	up	0.48	no	0.98	no	down	4.2E-02	yes
MSTRG.97.11 : Bclaf1 : B1WC16	0.67	yes	down	1.3E-07	yes	chr1	intron	internal intron	NA	1.05	no	up	0.78	no	1.03	no	up	6.8E-02	no
MSTRG.18206.4 : Cirbp : P60825	0.73	yes	down	1.3E-04	yes	chr7	exon	first exon	NA	1.24	no	up	0.69	no	0.85	no	down	3.4E-16	yes
ENSRNOT00000090211 : Nsfl1c : O35987	16.74	yes	up	1.8E-04	yes	chr3	intron	first intron	NA	0.94	no	down	0.21	no	0.98	no	down	1.6E-03	yes
MSTRG.5399.1 : Eln : Q99372	3.55	yes	up	3.0E-04	yes	chr12	intron	internal intron	NA	0.60	no	down	0.09	no	0.82	yes	down	3.9E-08	yes
ENSRNOT00000004206 : Mpc2 : P38718	0.25	yes	down	5.7E-05	yes	chr13	promoter	Intermediate	ICP	0.87	no	down	0.12	no	0.90	no	down	1.6E-06	yes
MSTRG.9106.1 : Nebl : F1LVX3	0	yes	down	3.8E-04	yes	chr17	intron	first intron	NA	0.83	no	down	0.24	no	1.02	no	up	2.0E-01	no
MSTRG.8094.6 : Atp13a1 : B5DEX7	3.83	yes	up	1.1E-13	yes	chr16	intron	internal intron	NA	1.41	no	up	0.52	no	1.09	no	up	4.2E-12	yes
ENSRNOT00000061169 : LOC361016 : A0A0G2JXI	0.05	yes	down	2.2E-18	yes	chr15	intron	first intron	NA	0.76	no	down	0.23	no	1.05	no	up	2.5E-01	no
ENSRNOT00000086605 : Chtopl1 : M0RDD7	0.48	yes	down	9.7E-06	yes	chr4	exon	last exon	NA	0.57	no	down	0.03	yes	1.25	yes	up	3.9E-09	yes
MSTRG.9694.1 : Cndp1 : Q66HG3	4.65	yes	up	2.7E-05	yes	chr18	intron	last intron	NA	1.00	no	down	0.97	no	1.01	no	up	5.4E-01	no
MSTRG.9721.6 : Cdh11 : F1MAH6	1.94	yes	up	2.2E-05	yes	chr19	exon	last exon	NA	1.10	no	up	0.09	no	0.91	no	down	6.5E-11	yes
MSTRG.5804.8 : Clasp1 : F1LNR1	0.36	yes	down	6.7E-07	yes	chr13	intron	internal intron	NA	4.32	yes	up	0.04	yes	1.00	no	down	7.0E-01	no
ENSRNOT00000077484 : Tcp11l1 : F1M9Y7	3.13	yes	up	1.2E-06	yes	chr3	intron	last intron	NA	0.94	no	down	0.69	no	0.99	no	down	8.5E-01	no
MSTRG.19463.2 : Tyk2 : D3ZD03	0.17	yes	down	1.5E-06	yes	chr8	intron	internal intron	NA	1.04	no	up	0.75	no	1.01	no	up	4.7E-01	no
MSTRG.8345.1 : Fgfr1 : F1LM54	0.22	yes	down	3.0E-05	yes	chr16	exon	last exon	NA	1.24	no	up	0.85	no	1.05	no	up	1.3E-02	yes
MSTRG.19963.4 : Sltm : A0A0G2K904	0.34	yes	down	6.4E-06	yes	chr8	intron	internal intron	NA	1.22	no	up	0.44	no	0.94	no	down	2.8E-02	yes
ENSRNOT00000076477 : Slfn13 : A0A096MKD0	2.27	yes	up	1.5E-04	yes	chr10	promoter	Intermediate	LCP	1.03	no	up	0.17	no	0.97	no	down	2.7E-02	yes
MSTRG.10688.1 : Drosha : E9PTR3	0.31	yes	down	1.4E-04	yes	chr2	intron	internal intron	NA	1.54	no	up	0.35	no	1.00	no	up	8.3E-01	no
MSTRG.3850.1 : Pnpo : O88794	2.62	yes	up	1.2E-06	yes	chr10	intron	internal intron	NA	1.22	no	up	0.54	no	0.99	no	down	6.7E-01	no
MSTRG.13870.2 : Plcg1 : G3V845	0.24	yes	down	1.1E-04	yes	chr3	intron	first intron	NA	1.22	no	up	0.69	no	0.94	no	down	1.8E-09	yes
ENSRNOT00000001738 : Atp2a2 : P11507	0.21	yes	down	5.6E-04	yes	chr12	intron	internal intron	NA	1.11	no	up	0.38	no	0.89	no	down	1.5E-13	yes
MSTRG.6713.3 : Fryl : D3ZQY4	2.76	yes	up	1.7E-04	yes	chr14	intron	internal intron	NA	0.68	no	down	0.36	no	1.00	no	down	4.6E-01	no
ENSRNOT00000024575 : Tpm1 : Q923Z2	9.41	yes	up	5.1E-14	yes	chr8	intron	internal intron	NA	0.69	no	down	0.37	no	0.75	yes	down	6.0E-18	yes
MSTRG.10603.5 : Nnt : Q5BJZ3	3.00	yes	up	1.8E-04	yes	chr2	intron	internal intron	NA	1.00	no	down	0.99	no	0.96	no	down	1.2E-07	yes
MSTRG.16317.5 : Cap1 : Q08163	0.21	yes	down	1.6E-07	yes	chr5	intron	internal intron	NA	1.24	no	up	0.29	no	0.94	no	down	1.0E-08	yes
MSTRG.18037.2 : Rbms2 : Q4QR81	0.04	yes	down	2.6E-23	yes	chr7	exon	last exon	NA	1.03	no	up	0.09	no	1.14	no	up	2.7E-10	yes
MSTRG.13731.3 : Mapre1 : Q66HR2	5.59	yes	up	4.6E-04	yes	chr3	intron	internal intron	NA	0.54	no	down	0.32	no	1.03	no	up	2.1E-03	yes
MSTRG.16461.7 : Pum1 : D3Z8L5	0.44	yes	down	3.1E-04	yes	chr5	intron	internal intron	NA	1.08	no	up	0.69	no	1.03	no	up	7.0E-02	no
MSTRG.8278.15 : Rbpms : F2Z3S5	0.02	yes	down	2.6E-05	yes	chr16	promoter	Proximal	ICP	1.23	no	up	0.15	no	1.06	no	up	4.9E-05	yes
MSTRG.1683.11 : Il4r : Q63257	9.80	yes	up	2.9E-08	yes	chr1	intron	internal intron	NA	0.62	no	down	0.05	yes	1.11	no	up	2.1E-05	yes
MSTRG.4736.1 : Osbpl11 : B5DF74	0.37	yes	down	2.7E-06	yes	chr11	exon	first exon	NA	1.12	no	up	0.47	no	1.07	no	up	2.3E-05	yes
MSTRG.16557.5 : Srrm1 : B2RYB3	2.05	yes	up	2.0E-04	yes	chr5	intron	internal intron	NA	0.95	no	down	0.87	no	1.10	no	up	2.8E-06	yes
MSTRG.19173.4 : Ano6 : A0A0G2K1M7	3.87	yes	up	9.8E-09	yes	chr7	intron	internal intron	NA	1.18	no	up	0.40	no	0.91	no	down	2.1E-09	yes
MSTRG.256.8 : Syne1 : Q8VHJ9	0.04	yes	down	1.5E-19	yes	chr1	exon	first exon	NA	1.04	no	up	0.78	no	0.89	no	down	8.9E-14	yes
MSTRG.1229.3 : Anpep : G3V7W7	0.25	yes	down	1.9E-09	yes	chr1	intron	internal intron	NA	0.72	no	down	0.36	no	1.05	no	up	3.0E-08	yes
MSTRG.10517.4 : Erbin : M0R9T2	3.86	yes	up	2.1E-05	yes	chr2	intron	last intron	NA	1.32	no	up	0.60	no	0.94	no	down	3.9E-05	yes
MSTRG.11268.1 : Tpm3 : Q63610	0.37	yes	down	8.0E-05	yes	chr2	intron	internal intron	NA	0.85	no	down	0.26	no	1.11	no	up	3.2E-12	yes
MSTRG.18880.1 : Plec : F7F9U6	0.33	yes	down	4.2E-06	yes	chr7	exon	last exon	NA	0.61	no	down	0.10	no	0.90	no	down	2.5E-03	yes
MSTRG.15075.1 : Alox5 : F1LMM5	0.38	yes	down	1.3E-04	yes	chr4	intron	internal intron	NA	1.16	no	up	0.66	no	0.96	no	down	9.7E-06	yes
MSTRG.2580.1 : Sfxn3 : Q6P6T0	0.39	yes	down	7.8E-05	yes	chr1	intron	internal intron	NA	1.28	no	up	0.08	no	1.12	no	up	6.4E-07	yes
MSTRG.16897.1 : Atl2 : F1LQ09	2.32	yes	up	2.9E-04	yes	chr6	intron	last intron	NA	1.22	no	up	0.18	no	0.95	no	down	8.9E-05	yes
ENSRNOT00000064822 : Ankmy2 : D3ZC34	2.16	yes	up	2.9E-04	yes	chr6	promoter	Distal	ICP	0.97	no	down	0.56	no	0.93	no	down	2.5E-09	yes
ENSRNOT00000080229 : Fam126a : A0A0G2K3C7	0.38	yes	down	2.9E-04	yes	chr4	promoter	Intermediate	LCP	1.02	no	up	0.54	no	1.07	no	up	1.8E-06	yes
ENSRNOT00000060168 : Pls3 : F1LPK7	0	yes	down	1.5E-04	yes	chrX	intron	internal intron	NA	0.95	no	down	0.51	no	1.11	no	up	9.8E-14	yes
MSTRG.3379.8 : Dlg4 : P31016	2.15	yes	up	8.6E-07	yes	chr10	intron	last intron	NA	0.98	no	down	0.89	no	0.97	no	down	9.1E-02	no
MSTRG.1229.4 : Anpep : G3V7W7	0.25	yes	down	1.9E-09	yes	chr1	intron	internal intron	NA	1.51	no	up	0.48	no	1.05	no	up	3.0E-08	yes
MSTRG.12318.2 : Dnajb12 : Q5FVC4	32.15	yes	up	1.5E-05	yes	chr20	exon	internal exon	NA	0.99	no	down	0.95	no	1.07	no	up	2.0E-04	yes
MSTRG.6919.1 : Cpz : A0A0G2JSJ7	3.01	yes	up	1.1E-05	yes	chr14	intron	internal intron	NA	1.49	no	up	0.48	no	1.08	no	up	5.1E-02	no
MSTRG.8278.11 : Rbpms : F2Z3S5	0.02	yes	down	2.6E-05	yes	chr16	promoter	Proximal	ICP	0.99	no	down	0.96	no	1.06	no	up	4.9E-05	yes
ENSRNOT00000079554 : Bmpr1a : Q78EA7	0.29	yes	down	2.1E-04	yes	chr16	intron	internal intron	NA	0.95	no	down	0.77	no	0.90	no	down	9.8E-10	yes
MSTRG.4866.2 : Vps8 : D4A5F7	7.39	yes	up	3.4E-05	yes	chr11	intron	internal intron	NA	1.06	no	up	0.60	no	0.95	no	down	3.2E-03	yes
MSTRG.17171.2 : Nbas : F1M0U5	5.28	yes	up	7.6E-38	yes	chr6	intron	internal intron	NA	0.95	no	down	0.62	no	1.02	no	up	1.1E-03	yes
MSTRG.11962.1 : Ddx39b : Q63413	0.02	yes	down	4.7E-11	yes	chr20	exon	last exon	NA	0.88	no	down	0.32	no	1.01	no	up	2.0E-01	no
MSTRG.13954.6 : Znfx1 : F1LMA9	20.79	yes	up	3.0E-04	yes	chr3	intron	internal intron	NA	1.12	no	up	0.85	no	0.92	no	down	1.0E-03	yes
ENSRNOT00000061114 : RT1-CE7 : D3ZLE6	inf	yes	up	7.3E-07	yes	chr20	exon	internal exon	NA	1.01	no	up	0.48	no	1.22	yes	up	7.3E-16	yes
MSTRG.8094.1 : Atp13a1 : B5DEX7	3.83	yes	up	1.1E-13	yes	chr16	intron	internal intron	NA	1.22	no	up	0.03	yes	1.09	no	up	4.2E-12	yes
MSTRG.17738.4 : Vrk1 : Q6AYA2	0.22	yes	down	2.5E-07	yes	chr6	intron	internal intron	NA	1.40	no	up	0.43	no	1.01	no	up	2.7E-01	no
MSTRG.14053.5 : Dido1 : D3ZWL9	2.83	yes	up	7.0E-07	yes	chr3	intron	internal intron	NA	0.94	no	down	0.76	no	1.01	no	up	2.0E-01	no
ENSRNOT00000016070 : Srcin1 : Q9QXY2	3.06	yes	up	2.8E-04	yes	chr10	intron	last intron	NA	1.06	no	up	0.23	no	1.05	no	up	1.2E-02	yes

MSTRG.7470.20 : Acin1 : E9PST5	0.30	yes	down	1.1E-04	yes	chr15	promoter	Distal	LCP	1.06	no	up	0.91	no	0.98	no	down	5.1E-04	yes
MSTRG.11953.24 : RT1-CE7 : D3ZLE6	inf	yes	up	7.3E-07	yes	chr20	exon	internal exon	NA	0.80	no	down	0.12	no	1.22	yes	up	7.3E-16	yes
ENSRNOT00000014464 : Anxa6 : Q6IMZ3	2.83	yes	up	1.2E-05	yes	chr10	intron	internal intron	NA	0.97	no	down	0.56	no	0.84	no	down	6.6E-16	yes
MSTRG.4992.3 : Top3b : D4A9Z2	11.41	yes	up	1.2E-04	yes	chr11	intron	last intron	NA	3.24	yes	up	0.01	yes	0.99	no	down	6.1E-01	no
MSTRG.15110.6 : Wnk1 : Q9JIH7	18.77	yes	up	1.6E-38	yes	chr4	exon	internal exon	NA	0.98	no	down	0.97	no	1.04	no	up	1.7E-03	yes
ENSRNOT00000019465 : Stat1 : F1M9D6	0.50	yes	down	1.5E-10	yes	chr9	intron	first intron	NA	1.03	no	up	0.95	no	1.10	no	up	1.4E-13	yes
MSTRG.19107.1 : Fbln1 : D3ZQ25	0.23	yes	down	7.2E-09	yes	chr7	intron	internal intron	NA	2.17	yes	up	0.17	no	1.06	no	up	1.7E-09	yes
ENSRNOT00000004764 : Efemp1 : Q6AXN2	0.06	yes	down	2.6E-05	yes	chr14	exon	last exon	NA	0.44	yes	down	0.22	no	0.94	no	down	1.1E-08	yes
MSTRG.9568.4 : Nedd4l : F1LRN8	2.03	yes	up	3.6E-05	yes	chr18	intron	internal intron	NA	1.14	no	up	0.25	no	0.95	no	down	3.9E-02	yes
MSTRG.19946.2 : Tln2 : D3ZA84	3.91	yes	up	1.0E-11	yes	chr8	intron	internal intron	NA	0.99	no	down	0.96	no	0.87	no	down	7.8E-17	yes
MSTRG.17738.5 : Vrk1 : Q6AYA2	0.22	yes	down	2.5E-07	yes	chr6	intron	internal intron	NA	0.79	no	down	0.50	no	1.01	no	up	2.7E-01	no
ENSRNOT00000086550 : Plec : Q6S395	0.33	yes	down	4.2E-06	yes	chr7	exon	last exon	NA	1.00	no	down	0.70	no	1.03	no	up	2.2E-01	no
MSTRG.20284.8 : Qrich1 : F1M4M7	3.68	yes	up	3.4E-07	yes	chr8	exon	internal exon	NA	1.08	no	up	0.73	no	1.03	no	up	2.0E-01	no
MSTRG.19655.2 : Kmt2a : F1M0L3	0.53	yes	down	1.7E-05	yes	chr8	exon	internal exon	NA	1.64	no	up	0.63	no	0.99	no	down	9.3E-01	no
MSTRG.10688.2 : Drosha : E9PTR3	0.31	yes	down	1.4E-04	yes	chr2	intron	internal intron	NA	1.07	no	up	0.84	no	1.00	no	up	8.3E-01	no
MSTRG.9106.3 : Nebl : F1LVX3	0	yes	down	3.8E-04	yes	chr17	intron	first intron	NA	1.01	no	up	0.09	no	1.02	no	up	2.0E-01	no
MSTRG.5983.1 : Cfh : F1M983	0.20	yes	down	2.4E-04	yes	chr13	intron	internal intron	NA	1.33	no	up	0.36	no	1.20	no	up	2.7E-15	yes
MSTRG.10711.2 : Ankh : F1LN34	3.44	yes	up	9.7E-06	yes	chr2	intron	first intron	NA	1.74	no	up	0.06	no	1.20	yes	up	7.4E-12	yes
MSTRG.21491.1 : Phf8 : D4AD31	0	yes	down	2.1E-07	yes	chrX	exon	first exon	NA	0.50	yes	down	0.45	no	1.13	no	up	3.6E-02	yes
MSTRG.11636.1 : Larp7 : M0R7D1	0.06	yes	down	3.4E-04	yes	chr2	exon	last exon	NA	0.77	no	down	0.22	no	1.07	no	up	2.0E-06	yes
ENSRNOT00000092075 : Lims1 : C0KUC5	12.34	yes	up	5.9E-05	yes	chr20	intron	internal intron	NA	1.11	no	up	0.58	no	0.77	yes	down	1.9E-12	yes
MSTRG.4725.15 : Kalrn : P97924	1.54	yes	up	9.1E-07	yes	chr11	exon	last exon	NA	0.93	no	down	0.56	no	1.04	no	up	1.6E-04	yes
MSTRG.5482.20 : Ncor2 : A0A0G2JU91	2.16	yes	up	1.6E-06	yes	chr12	intron	internal intron	NA	0.94	no	down	0.43	no	1.01	no	up	7.1E-01	no
ENSRNOT00000044209 : Lrp1b : F1M443	0.05	yes	down	8.3E-06	yes	chr3	intron	internal intron	NA	1.02	no	up	0.48	no	0.81	yes	down	1.7E-02	yes
MSTRG.18268.23 : Akap8 : Q63014	0.08	yes	down	2.7E-04	yes	chr7	intron	internal intron	NA	1.69	no	up	0.13	no	0.99	no	down	2.8E-01	no
MSTRG.9752.2 : Coq9 : Q68FT1	0.32	yes	down	5.1E-11	yes	chr19	promoter	Distal	LCP	0.99	no	down	0.99	no	0.93	no	down	1.8E-05	yes
ENSRNOT00000086812 : Atm : A0A0G2K3I0	0.65	yes	down	2.4E-04	yes	chr8	intron	internal intron	NA	1.67	no	up	0.29	no	1.01	no	up	4.6E-01	no
MSTRG.1551.1 : Ctr9 : G3V897	12.42	yes	up	1.5E-04	yes	chr1	intron	internal intron	NA	0.57	no	down	0.51	no	1.02	no	up	9.9E-02	no
ENSRNOT00000083959 : Ptdss1 : Q5PQL5	4.36	yes	up	3.7E-04	yes	chr7	exon	first exon	NA	1.11	no	up	0.49	no	1.08	no	up	1.2E-04	yes
MSTRG.5410.4 : Gtf2i : Q5U2Y1	5.31	yes	up	4.9E-06	yes	chr12	promoter	Distal	LCP	1.51	no	up	0.10	no	0.95	no	down	1.5E-07	yes
ENSRNOT00000092693 : Upf2 : D3ZT03	0.21	yes	down	9.8E-07	yes	chr17	intron	internal intron	NA	1.05	no	up	0.61	no	1.03	no	up	3.0E-03	yes
MSTRG.4547.2 : Arl6 : B1WC73	5.24	yes	up	2.5E-08	yes	chr11	exon	first exon	NA	1.27	no	up	0.00	yes	0.98	no	down	1.6E-01	no
MSTRG.8552.11 : Ddx46 : Q62780	0.56	yes	down	3.4E-04	yes	chr17	intron	internal intron	NA	0.79	no	down	0.55	no	0.98	no	down	1.3E-01	no
ENSRNOT00000001466 : Foxk1 : D3ZU55	4.61	yes	up	3.7E-14	yes	chr12	intron	internal intron	NA	1.11	no	up	0.76	no	0.99	no	down	5.1E-01	no
ENSRNOT00000014217 : Cand2 : G3V7E8	0.39	yes	down	2.9E-04	yes	chr4	exon	internal exon	NA	1.36	no	up	0.15	no	0.97	no	down	1.3E-05	yes
MSTRG.11223.5 : Fdps : F1LND7	inf	yes	up	2.2E-05	yes	chr2	exon	last exon	NA	0.74	no	down	0.31	no	1.07	no	up	1.2E-03	yes
MSTRG.6320.3 : Cdc42bpa : G3V6C9	8.92	yes	up	2.1E-05	yes	chr13	intron	internal intron	NA	1.67	no	up	0.48	no	0.97	no	down	2.5E-04	yes
MSTRG.3793.2 : Lrrc59 : Q5RJR8	4.07	yes	up	4.6E-04	yes	chr10	exon	internal exon	NA	1.80	no	up	0.04	yes	1.13	no	up	5.6E-13	yes
MSTRG.7226.4 : Sec24c : A0A0G2JZF0	3.50	yes	up	1.3E-09	yes	chr15	intron	internal intron	NA	1.10	no	up	0.79	no	1.01	no	up	2.6E-01	no
ENSRNOT00000030229 : Ccdc102a : D3ZSR7	0.89	no	down	6.8E-05	yes	chr19	intron	internal intron	NA	1.11	no	up	0.43	no	0.95	no	down	3.2E-05	yes
MSTRG.14960.1 : Eogt : Q5NDL0	6.71	yes	up	1.7E-04	yes	chr4	intron	internal intron	NA	1.23	no	up	0.53	no	1.10	no	up	2.0E-05	yes
ENSRNOT00000039354 : Nf1x : F2Z3R4	0.58	yes	down	1.9E-06	yes	chr19	intron	internal intron	NA	0.83	no	down	0.47	no	0.77	yes	down	2.4E-14	yes
MSTRG.3379.7 : Dlg4 : P31016	2.15	yes	up	8.6E-07	yes	chr10	intron	last intron	NA	1.09	no	up	0.60	no	0.97	no	down	9.1E-02	no
MSTRG.9868.1 : Cc2d1a : Q66HA5	0.21	yes	down	1.7E-05	yes	chr19	promoter	Distal	LCP	1.10	no	up	0.50	no	0.96	no	down	4.1E-02	yes
MSTRG.12646.3 : Rapgef1 : F1M8L9	inf	yes	up	4.7E-05	yes	chr3	intron	internal intron	NA	1.07	no	up	0.16	no	0.88	no	down	4.1E-04	yes
ENSRNOT00000015092 : Pkd1 : Q9ERV0	inf	yes	up	6.6E-05	yes	chr10	intron	internal intron	NA	1.01	no	up	0.28	no	1.19	no	up	9.8E-07	yes
ENSRNOT00000001766 : Bcr : F1LXF1	2.12	yes	up	1.3E-04	yes	chr20	intron	first intron	NA	0.90	no	down	0.56	no	0.96	no	down	8.7E-05	yes
MSTRG.13196.1 : Arhgap1 : D4A6C5	3.73	yes	up	1.8E-11	yes	chr3	intron	internal intron	NA	0.92	no	down	0.47	no	1.04	no	up	1.7E-04	yes
MSTRG.4481.2 : Gart : G3V918	0.39	yes	down	2.0E-05	yes	chr11	intron	internal intron	NA	0.79	no	down	0.36	no	0.96	no	down	6.3E-04	yes
MSTRG.5159.2 : Tecpr1 : Q3ZBA0	0.43	yes	down	3.1E-04	yes	chr12	promoter	Intermediate	LCP	1.45	no	up	0.33	no	1.17	no	up	7.7E-08	yes
MSTRG.21938.1 : Klhl13 : F1LM44	0.22	yes	down	7.1E-07	yes	chrX	intron	last intron	NA	1.06	no	up	0.74	no	1.03	no	up	6.8E-03	yes
MSTRG.11380.1 : Txnip : Q5M7W1	6.38	yes	up	4.0E-07	yes	chr2	exon	internal exon	NA	0.74	no	down	0.40	no	0.85	no	down	1.0E-06	yes
MSTRG.1443.1 : Arap1 : F1LM60	2.71	yes	up	3.3E-04	yes	chr1	intron	first intron	NA	1.33	no	up	0.30	no	0.99	no	down	2.5E-02	yes
MSTRG.2907.2 : Unkl : D4A3S7	2.08	yes	up	2.1E-04	yes	chr10	intron	internal intron	NA	1.16	no	up	0.44	no	0.84	no	down	1.1E-15	yes
MSTRG.6982.1 : Morc2 : D4A2C4	6.36	yes	up	1.5E-04	yes	chr14	promoter	Proximal	HCP	0.67	no	down	0.49	no	0.97	no	down	3.2E-01	no
MSTRG.19088.2 : Scube1 : F1M987	2.13	yes	up	1.3E-04	yes	chr7	intron	internal intron	NA	1.20	no	up	0.69	no	0.80	yes	down	1.2E-14	yes
MSTRG.17733.4 : Atg2b : Q5EBA2	0.42	yes	down	4.5E-04	yes	chr6	exon	internal exon	NA	0.79	no	down	0.54	no	1.03	no	up	1.2E-01	no
MSTRG.8517.5 : Naa35 : Q6DKG0	inf	yes	up	1.7E-04	yes	chr17	promoter	Proximal	LCP	0.95	no	down	0.84	no	1.04	no	up	3.3E-02	yes
ENSRNOT00000015946 : Psma1 : P18420	11.23	yes	up	1.1E-12	yes	chr1	exon	last exon	NA	1.24	no	up	0.01	yes	0.92	no	down	1.8E-10	yes
MSTRG.97.7 : Bclaf1 : B1WC16	0.67	yes	down	1.3E-07	yes	chr1	intron	internal intron	NA	0.96	no	down	0.87	no	1.03	no	up	6.8E-02	no
MSTRG.3353.4 : Zbtb4 : D4A8X0	inf	yes	up	2.4E-05	yes	chr10	intron	last intron	NA	0.86	no	down	0.82	no	0.97	no	down	6.9E-02	no
MSTRG.11953.31 : RT1-CE7 : D3ZLE6	inf	yes	up	7.3E-07	yes	chr20	exon	internal exon	NA	0.96	no	down	0.90	no	1.22	yes	up	7.3E-16	yes
MSTRG.8278.22 : Rbpms : F2Z3S5	0.02	yes	down	2.6E-05	yes	chr16	promoter	Proximal	ICP	1.08	no	up	0.63	no	1.06	no	up	4.9E-05	yes
ENSRNOT00000013353 : Puf60 : A0A0H2UHZ6	0.25	yes	down	1.3E-05	yes	chr7	exon	internal exon	NA	0.84	no	down	0.46	no	1.00	no	down	7.4E-02	no
MSTRG.110.3 : Lama2 : F1M614	0.44	yes	down	4.4E-04	yes	chr1	intron	internal intron	NA	1.05	no	up	0.53	no	1.06	no	up	1.3E-07	yes
MSTRG.3783.4 : Acsf2 : Q499N5	0.31	yes	down	5.4E-07	yes	chr10	intron	internal intron	NA	1.00	no	down	0.99	no	0.87	no	down	2.4E-13	yes
MSTRG.7470.16 : Acin1 : E9PST5	0.30	yes	down	1.1E-04	yes	chr15	promoter	Distal	LCP	0.47	yes	down	0.27	no	0.98	no	down	5.1E-04	yes
MSTRG.3645.11 : Slfn13 : A0A096MKD0	2.27	yes	up	1.5E-04	yes	chr10	promoter	Intermediate	LCP	2.61	yes	up	0.12	no	0.97	no	down	2.7E-02	yes
MSTRG.17688.2 : Golga5 : G3V6Z7	4.67	yes	up	4.2E-17	yes	chr6	intron	last intron	NA	1.84	no	up	0.17	no	1.07	no	up	2.3E-12	yes
MSTRG.3895.8 : Cdk12 : A0A0G2K5U7	5.94	yes	up	1.1E-04	yes	chr10	exon	first exon	NA	0.75	no	down	0.29	no	0.97	no	down	5.5E-04	yes
ENSRNOT00000080623 : Cdc42bpb : A0A0G2KB58	0.39	yes	down	3.8E-04	yes	chr6	intron	internal intron	NA	0.94	no	down	0.83	no	0.97	no	down	3.6E-07	yes
ENSRNOT00000092739 : Sema4a : A0A1B0GWV9	3.09	yes	up	1.9E-04	yes	chr2	promoter	Intermediate	LCP	1.47	no	up	0.08	no	1.07	no	up	2.4E-05	yes
MSTRG.21144.3 : Cab39 : A0A0G2JZH0	39.86	yes	up	1.2E-27	yes	chr9	promoter	Intermediate	LCP	0.44	yes	down	0.15	no	0.99	no	down	5.6E-01	no
MSTRG.3448.1 : P2rx1 : B7U2F3	5.09	yes	up	1.5E-10	yes	chr10	promoter	Intermediate	LCP	1.16	no	up	0.38	no	0.82	yes	down	1.7E-12	yes
MSTRG.9803.2 : Rbl2 : G3V7P7	0.41	yes	down	2.1E-05	yes	chr19	intron	internal intron	NA	1.10	no	up	0.10	no	0.89	no	down	1.5E-05	yes
MSTRG.7082.4 : Grb10 : D3ZEA3	0.35	yes	down	3.4E-07	yes	chr14	intron	internal intron	NA	1.41	no	up	0.43	no	1.09	no	up	4.5E-06	yes
ENSRNOT00000092882 : Stim1 : P84903	2.61	yes	up	1.3E-04	yes	chr1	exon	last exon	NA	1.00	no	down	0.98	no	0.92	no	down	3.4E-08	yes
ENSRNOT00000019735 : Pdcd6 : G3V7W1	15.41	yes	up	1.1E-04	yes	chr1	promoter	Distal	LCP	1.16	no	up	0.66	no	0.95	no	down	4.9E-04	yes
ENSRNOT00000091612 : Clu : G3V836	0.38	yes	down	6.6E-05	yes	chr15	intron	first intron	NA	0.47	yes	down	0.16	no	1.26	yes	up	5.8E-16	yes
ENSRNOT00000021840 : Igf2r : Q63002	0.32	yes	down	2.3E-06	yes	chr1	promoter	Distal	ICP	1.43	no	up	0.23	no	1.15	no	up	2.6E-11	yes
MSTRG.18268.29 : Akap8 : Q63014	0.08	yes	down	2.7E-04	yes	chr7	intron	internal intron	NA	0.60	no	down	0.42	no	0.99	no	down	2.8E-01	no
MSTRG.21083.16 : Speg : Q63638	0.17	yes	down	2.6E-06	yes	chr9	intron	internal intron	NA	0.57	no	down	0.51	no	0.88	no	down	2.3E-07	yes
ENSRNOT00000023392 : Mark4 : A0A0G2JTD8	0.28	yes	down	3.4E-05	yes	chr1	intron	internal intron	NA	0.81	no	down	0.08	no	0.99	no	down	7.5E-01	no
ENSRNOT00000011096 : Ddx47 : G3V727	0.37	yes	down	4.0E-04	yes	chr4	intron	internal intron	NA	1.03	no	up	0.67	no	1.05	no	up	9.1E-04	yes
MSTRG.19963.2 : Sltm : A0A0G2K904	0.34	yes	down	6.4E-06	yes	chr8	intron	internal intron	NA	1.00	no	down	0.99	no	0.94	no	down	2.8E-02	yes
ENSRNOT00000002091 : Gap43 : P07936	inf	yes	up	3.1E-17	yes	chr11	intron	first intron	NA	0.84	no	down	0.05	yes	0.89	no	down	9.3E-06	yes
ENSRNOT00000072851 : Vps26b : B1WBS4	3.89	yes	up	3.2E-04	yes	chr8	intron	internal intron	NA	1.20	no	up	0.11	no	0.94	no	down	1.6E-09	yes
MSTRG.9649.1 : Hdhd2 : Q6QI86	0.20	yes	down	5.1E-06	yes	chr18	intron	first intron	NA	0.96	no	down	0.18	no	1.04	no	up	3.3E-03	yes
ENSRNOT00000088712 : Elmsan1 : D4ACA6	0.27	yes	down	2.9E-04	yes	chr6	exon	last exon	NA	1.00	no	down	1.00	no	0.96	no	down	1.9E-01	no
MSTRG.12423.1 : Fyn : Q62844	0.38	yes	down	2.4E-05	yes	chr20	exon	last exon	NA	0.97	no	down	0.87	no	1.01	no	up	5.2E-01	no
MSTRG.7830.2 : Dock9 : F1LSM8	0.10	yes	down	2.7E-05	yes	chr15	intron	internal intron	NA	0.58	no	down	0.14	no	0.97	no	down	2.3E-04	yes
MSTRG.19777.3 : Sin3a : A0A0G2K3H5	0.53	yes	down	4.1E-06	yes	chr8	intron	last intron	NA	0.68	no	down	0.44	no	0.96	no	down	6.2E-05	yes
ENSRNOT00000023706 : Tbc1d10b : D3ZSY8	1.59	yes	up	2.7E-04	yes	chr1	intron	internal intron	NA	1.06	no	up	0.47	no	0.99	no	down	3.1E-01	no
ENSRNOT00000048026 : Drosha : E9PTR3	0.31	yes	down	1.4E-04	yes	chr2	intron	internal intron	NA	0.75	no	down	0.38	no	1.00	no	up	8.3E-01	no
MSTRG.3781.4 : Luc7l3 : D3ZFB2	0.31	yes	down	4.3E-04	yes	chr10	intron	internal intron	NA	1.07	no	up	0.66	no	0.98	no	down	9.6E-04	yes
MSTRG.15938.2 : Tnc : A0A0G2K1L0	0	yes	down	7.1E-07	yes	chr5	intron	internal intron	NA	1.15	no	up	0.84	no	1.49	yes	up	1.5E-12	yes
MSTRG.7804.6 : Mycbp2 : A0A1W2Q6I3	0.31	yes	down	9.2E-06	yes	chr15	intron	internal intron	NA	2.39	yes	up	0.06	no	1.00	no	up	8.8E-01	no
MSTRG.11955.1 : RT1-CE7 : D3ZLE6	inf	yes	up	7.3E-07	yes	chr20	exon	internal exon	NA	0.36	yes	down	0.11	no	1.22	yes	up	7.3E-16	yes
MSTRG.18268.14 : Akap8 : Q63014	0.08	yes	down	2.7E-04	yes	chr7	intron	internal intron	NA	1.07	no	up	0.62	no	0.99	no	down	2.8E-01	no
ENSRNOT00000024622 : Ces1f : M0R7R1	0.26	yes	down	2.8E-08	yes	chr19	intron	internal intron	NA	1.02	no	up	0.27	no	0.86	no	down	3.2E-11	yes

MSTRG.21399.1 : Ddx3x : A0A0G2K719	3.18	yes	up	1.4E-05	yes	chrX	exon	first exon	NA	1.52	no	up	0.52	no	1.11	no	up	3.1E-07	yes
MSTRG.7395.1 : Supt16h : D4A4J0	3.93	yes	up	5.1E-06	yes	chr15	intron	internal intron	NA	1.37	no	up	0.04	yes	1.01	no	up	1.6E-03	yes
MSTRG.16461.9 : Pum1 : D3Z8L5	0.44	yes	down	3.1E-04	yes	chr5	intron	internal intron	NA	0.85	no	down	0.35	no	1.03	no	up	7.0E-02	no
ENSRNOT00000007092 : Shmt1 : Q6TXG7	0.30	yes	down	2.1E-05	yes	chr10	intron	internal intron	NA	1.01	no	up	0.74	no	0.90	no	down	1.3E-08	yes
ENSRNOT00000092890 : Trim21 : D4ACF2	0.20	yes	down	5.7E-10	yes	chr1	promoter	Distal	LCP	0.97	no	down	0.51	no	0.96	no	down	1.3E-04	yes
MSTRG.7171.4 : Efemp1 : Q6AXN2	0.06	yes	down	2.6E-05	yes	chr14	exon	last exon	NA	1.01	no	up	0.13	no	0.94	no	down	1.1E-08	yes
ENSRNOT00000037115 : Golim4 : D3ZM57	0.34	yes	down	3.2E-04	yes	chr2	intron	internal intron	NA	0.88	no	down	0.60	no	0.55	yes	down	1.1E-01	no
MSTRG.13382.1 : Vps39 : E9PT04	inf	yes	up	4.3E-04	yes	chr3	intron	internal intron	NA	1.01	no	up	0.97	no	1.08	no	up	2.7E-04	yes
MSTRG.11285.2 : Npr1 : P18910	0.37	yes	down	1.2E-04	yes	chr2	intron	internal intron	NA	1.27	no	up	0.71	no	1.02	no	up	1.6E-02	yes
ENSRNOT00000093119 : Dnajc11 : B1WBY5	2.59	yes	up	1.9E-14	yes	chr5	promoter	Intermediate	ICP	1.05	no	up	0.66	no	0.88	no	down	4.2E-12	yes
MSTRG.20941.2 : Casp8 : Q9JHX4	11.45	yes	up	1.6E-14	yes	chr9	intron	first intron	NA	1.02	no	up	0.97	no	1.03	no	up	4.4E-02	yes
ENSRNOT00000076618 : Slfn13 : A0A096MKD0	2.27	yes	up	1.5E-04	yes	chr10	promoter	Intermediate	LCP	1.38	no	up	0.09	no	0.97	no	down	2.7E-02	yes
MSTRG.8552.2 : Ddx46 : Q62780	0.56	yes	down	3.4E-04	yes	chr17	intron	internal intron	NA	0.76	no	down	0.32	no	0.98	no	down	1.3E-01	no
MSTRG.4716.4 : Adcy5 : G3V9G1	0.16	yes	down	2.3E-10	yes	chr11	intron	internal intron	NA	1.10	no	up	0.84	no	1.00	no	down	7.6E-01	no
MSTRG.1731.5 : Tbc1d10b : D3ZSY8	1.59	yes	up	2.7E-04	yes	chr1	intron	internal intron	NA	0.75	no	down	0.43	no	0.99	no	down	3.1E-01	no
ENSRNOT00000018170 : Mat2a : F1LRB8	0.10	yes	down	1.4E-04	yes	chr4	exon	last exon	NA	0.96	no	down	0.59	no	0.99	no	down	1.5E-01	no
MSTRG.9995.1 : Fhod1 : A0A0G2JZ38	0.32	yes	down	5.3E-04	yes	chr19	exon	internal exon	NA	1.22	no	up	0.15	no	1.02	no	up	1.8E-02	yes
MSTRG.9560.2 : Wdr7 : Q9ERH3	0.16	yes	down	4.0E-04	yes	chr18	promoter	Distal	LCP	0.78	no	down	0.66	no	0.99	no	down	6.5E-01	no
MSTRG.9721.2 : Cdh11 : F1MAH6	1.94	yes	up	2.2E-05	yes	chr19	exon	last exon	NA	1.88	no	up	0.57	no	0.91	no	down	6.5E-11	yes
ENSRNOT00000081866 : Smc5 : D4A9F0	4.35	yes	up	2.4E-09	yes	chr1	promoter	Distal	LCP	0.91	no	down	0.63	no	0.86	no	down	3.1E-03	yes
ENSRNOT00000092442 : Upf2 : D3ZT03	0.21	yes	down	9.8E-07	yes	chr17	intron	internal intron	NA	1.09	no	up	0.89	no	1.03	no	up	3.0E-03	yes
MSTRG.20798.1 : Mitd1 : Q5I0J5	0.26	yes	down	9.9E-05	yes	chr9	promoter	Intermediate	LCP	0.81	no	down	0.54	no	1.14	no	up	9.0E-08	yes
MSTRG.323.9 : LOC108348175 : A0A0G2JUS0	0.05	yes	down	4.0E-19	yes	chr1	promoter	Intermediate	LCP	1.01	no	up	0.93	no	1.01	no	up	4.0E-01	no
ENSRNOT00000051680 : Gp1bb : Q9JJM7	0	yes	down	2.2E-04	yes	chr11	intron	internal intron	NA	1.32	no	up	0.33	no	0.93	no	down	7.7E-08	yes
MSTRG.1326.2 : Picalm : Q66WT9	8.74	yes	up	2.1E-13	yes	chr1	intron	internal intron	NA	1.00	no	down	0.99	no	1.45	yes	up	1.6E-10	yes
ENSRNOT00000015831 : Pum1 : D3Z8L5	0.44	yes	down	3.1E-04	yes	chr5	intron	internal intron	NA	1.01	no	up	0.92	no	1.03	no	up	7.0E-02	no
MSTRG.7179.2 : Ccdc88a : D3ZYD7	6.58	yes	up	8.2E-05	yes	chr14	promoter	Distal	LCP	2.27	yes	up	0.14	no	1.03	no	up	5.3E-03	yes
ENSRNOT00000076967 : Lsm14a : A0A0G2JUK2	0.31	yes	down	3.8E-04	yes	chr1	intron	last intron	NA	0.94	no	down	0.65	no	1.02	no	up	2.8E-01	no
MSTRG.5482.15 : Ncor2 : A0A0G2JU91	2.16	yes	up	1.6E-06	yes	chr12	intron	internal intron	NA	1.31	no	up	0.24	no	1.01	no	up	7.1E-01	no
ENSRNOT00000089354 : Aamp : B0K024	0.09	yes	down	2.6E-04	yes	chr9	promoter	Intermediate	LCP	0.98	no	down	0.94	no	1.06	no	up	1.5E-05	yes
ENSRNOT00000084554 : Gmpr2 : A0A0G2JX25	0.33	yes	down	2.2E-06	yes	chr15	promoter	Distal	LCP	0.92	no	down	0.11	no	0.95	no	down	1.0E-01	no
MSTRG.5613.4 : Tbx3 : A0A0G2K8D7	2.83	yes	up	2.0E-10	yes	chr12	intron	internal intron	NA	1.86	no	up	0.06	no	0.99	no	down	7.3E-01	no
MSTRG.18037.3 : Rbms2 : Q4QR81	0.04	yes	down	2.6E-23	yes	chr7	exon	last exon	NA	2.93	yes	up	0.08	no	1.14	no	up	2.7E-10	yes
ENSRNOT00000020527 : Atf7 : B0BMY0	0.38	yes	down	7.2E-06	yes	chr7	intron	internal intron	NA	1.04	no	up	0.09	no	1.00	no	down	9.7E-01	no
ENSRNOT00000047408 : Efl1 : D3ZXJ5	2.28	yes	up	1.6E-04	yes	chr1	intron	internal intron	NA	1.14	no	up	0.15	no	0.94	no	down	7.5E-05	yes
MSTRG.18206.9 : Cirbp : P60825	0.73	yes	down	1.3E-04	yes	chr7	exon	first exon	NA	1.12	no	up	0.43	no	0.85	no	down	3.4E-16	yes
MSTRG.13391.2 : Lrrc57 : Q5FVI3	0.39	yes	down	6.1E-06	yes	chr3	intron	internal intron	NA	0.88	no	down	0.73	no	0.91	no	down	5.9E-09	yes
ENSRNOT00000045708 : Phf8 : D4AD31	0	yes	down	2.1E-07	yes	chrX	exon	first exon	NA	3.41	yes	up	0.17	no	1.13	no	up	3.6E-02	yes
ENSRNOT00000008210 : Sptbn1 : A0A0G2K8W9	5.72	yes	up	4.0E-04	yes	chr14	intron	internal intron	NA	1.00	no	down	0.45	no	0.87	no	down	1.1E-18	yes
MSTRG.15798.1 : Tex10 : D4A401	5.97	yes	up	4.0E-04	yes	chr5	intron	internal intron	NA	1.67	no	up	0.09	no	0.97	no	down	1.1E-01	no
ENSRNOT00000020032 : Stat1 : F1M9D6	0.50	yes	down	1.5E-10	yes	chr9	intron	first intron	NA	1.45	no	up	0.23	no	1.10	no	up	1.4E-13	yes
MSTRG.15215.2 : Vwf : F5XVC1	0.45	yes	down	3.0E-05	yes	chr4	intron	internal intron	NA	0.31	yes	down	0.02	yes	0.74	yes	down	1.0E-18	yes
MSTRG.4525.2 : Mx1 : Q499S4	3.65	yes	up	1.1E-13	yes	chr11	intron	internal intron	NA	1.84	no	up	0.64	no	1.36	yes	up	1.2E-13	yes
MSTRG.13195.4 : Arhgap1 : D4A6C5	3.73	yes	up	1.8E-11	yes	chr3	intron	internal intron	NA	0.91	no	down	0.73	no	1.04	no	up	1.7E-04	yes
ENSRNOT00000073809 : Afmid : M0RC77	4.72	yes	up	9.0E-06	yes	chr10	promoter	Intermediate	LCP	0.80	no	down	0.18	no	0.81	yes	down	7.4E-05	yes
ENSRNOT00000002814 : Fras1 : F1M3H3	2.21	yes	up	1.3E-04	yes	chr14	intron	internal intron	NA	1.00	no	down	0.87	no	0.81	yes	down	2.2E-05	yes
MSTRG.6812.3 : Pcdh7 : Q68HB8	0	yes	down	4.2E-04	yes	chr14	exon	last exon	NA	0.62	no	down	0.17	no	0.94	no	down	1.0E-07	yes
ENSRNOT00000088945 : Plec : F7F9U6	0.33	yes	down	4.2E-06	yes	chr7	exon	last exon	NA	1.11	no	up	0.50	no	0.90	no	down	2.5E-03	yes
ENSRNOT00000080880 : ST7 : A0A0G2KBB5	4.88	yes	up	1.5E-16	yes	chr4	promoter	Distal	LCP	0.99	no	down	0.91	no	1.14	no	up	1.4E-03	yes
MSTRG.20218.2 : Rpl29 : P25886	3.86	yes	up	3.0E-04	yes	chr8	intron	first intron	NA	2.60	yes	up	0.43	no	0.98	no	down	6.0E-01	no
ENSRNOT00000076266 : Parp4 : A0A096MJR6	1.23	yes	up	1.1E-06	yes	chr15	intron	last intron	NA	0.97	no	down	0.67	no	0.95	no	down	6.6E-05	yes
MSTRG.16461.8 : Pum1 : D3Z8L5	0.44	yes	down	3.1E-04	yes	chr5	intron	internal intron	NA	0.91	no	down	0.75	no	1.03	no	up	7.0E-02	no
MSTRG.8514.7 : Zcchc6 : D3ZKR9	0.14	yes	down	1.2E-08	yes	chr17	exon	internal exon	NA	0.92	no	down	0.86	no	1.12	no	up	2.3E-06	yes
MSTRG.1427.2 : Arhgef17 : A0A0G2JXT9	0.45	yes	down	6.0E-05	yes	chr1	exon	last exon	NA	1.17	no	up	0.06	no	0.93	no	down	3.1E-10	yes
ENSRNOT00000082233 : Ddx39b : Q63413	0.02	yes	down	4.7E-11	yes	chr20	exon	last exon	NA	1.00	no	down	0.48	no	1.01	no	up	2.0E-01	no
ENSRNOT00000002403 : Pcyt1a : P19836	0.29	yes	down	1.2E-05	yes	chr11	exon	first exon	NA	0.84	no	down	0.65	no	0.94	no	down	3.3E-07	yes
ENSRNOT00000074738 : Phf11b : M0RB46	3.03	yes	up	2.9E-04	yes	chr15	promoter	Intermediate	LCP	1.59	no	up	0.29	no	1.15	no	up	2.3E-06	yes
MSTRG.2021.4 : Unc93b1 : D3ZDJ4	3.27	yes	up	6.0E-05	yes	chr1	intron	internal intron	NA	0.58	no	down	0.48	no	1.19	no	up	9.2E-05	yes
MSTRG.5942.1 : Ppp1r12b : D3ZIC4	2.69	yes	up	3.9E-04	yes	chr13	intron	internal intron	NA	0.86	no	down	0.06	no	0.95	no	down	7.9E-06	yes
ENSRNOT00000067325 : Dnmt1 : D3ZS06	0.16	yes	down	6.3E-05	yes	chr8	intron	internal intron	NA	1.13	no	up	0.42	no	1.00	no	down	4.1E-01	no
MSTRG.12655.1 : Gle1 : Q4KLN4	0.16	yes	down	1.6E-04	yes	chr3	promoter	Proximal	LCP	0.65	no	down	0.30	no	0.95	no	down	1.3E-02	yes
ENSRNOT00000028244 : Smarcc2 : D4A510	6.20	yes	up	2.1E-04	yes	chr7	intron	last intron	NA	1.37	no	up	0.39	no	0.96	no	down	6.4E-05	yes
ENSRNOT00000020335 : Ptgr1 : P97584	12.89	yes	up	1.1E-33	yes	chr5	intron	internal intron	NA	1.08	no	up	0.35	no	0.97	no	down	6.9E-02	no
MSTRG.21083.12 : Speg : Q63638	0.17	yes	down	2.6E-06	yes	chr9	intron	internal intron	NA	0.90	no	down	0.77	no	0.88	no	down	2.3E-07	yes
MSTRG.6746.7 : Pds5a : A4L9P7	1.26	yes	up	1.3E-13	yes	chr14	exon	last exon	NA	1.29	no	up	0.06	no	1.01	no	up	2.7E-01	no
MSTRG.4248.3 : Mxra7 : F1M1U0	inf	yes	up	4.6E-04	yes	chr10	intron	internal intron	NA	0.60	no	down	0.34	no	1.16	no	up	7.7E-10	yes
ENSRNOT00000032240 : Trappc10 : F1MAQ4	0.63	yes	down	1.1E-04	yes	chr20	exon	last exon	NA	1.33	no	up	0.59	no	0.99	no	down	5.9E-01	no
MSTRG.21926.2 : Pls3 : F1LPK7	0	yes	down	1.5E-04	yes	chrX	intron	internal intron	NA	1.24	no	up	0.08	no	1.11	no	up	9.8E-14	yes
ENSRNOT00000036613 : Rbm27 : F1M1R4	0.45	yes	down	2.5E-04	yes	chr18	intron	internal intron	NA	0.96	no	down	0.94	no	1.01	no	up	5.2E-01	no
ENSRNOT00000024714 : Ncoa6 : G3V8C9	7.63	yes	up	1.0E-10	yes	chr3	exon	internal exon	NA	0.73	no	down	0.71	no	1.03	no	up	4.4E-03	yes
ENSRNOT00000010277 : Rbpj : M0R7Q3	0.34	yes	down	7.0E-05	yes	chr14	intron	last intron	NA	1.02	no	up	0.23	no	1.08	no	up	1.1E-04	yes
MSTRG.10218.4 : Zc3h18 : Q6TQE1	0.03	yes	down	1.2E-11	yes	chr19	promoter	Intermediate	LCP	1.01	no	up	0.98	no	1.02	no	up	2.3E-01	no
ENSRNOT00000085279 : Ephx1 : P07687	0.19	yes	down	2.9E-07	yes	chr13	promoter	Intermediate	LCP	0.89	no	down	0.77	no	0.69	yes	down	5.2E-14	yes
ENSRNOT00000041988 : Tax1bp1 : Q66HA4	8.48	yes	up	1.1E-06	yes	chr4	exon	last exon	NA	0.97	no	down	0.31	no	1.04	no	up	2.0E-04	yes
MSTRG.20095.1 : Plscr1 : A0A0G2K7Q1	0.33	yes	down	1.3E-05	yes	chr8	promoter	Distal	LCP	0.95	no	down	0.84	no	1.08	no	up	5.2E-06	yes
ENSRNOT00000090638 : Syne1 : Q8VHJ9	0.04	yes	down	1.5E-19	yes	chr1	exon	first exon	NA	0.89	no	down	0.73	no	0.89	no	down	8.9E-14	yes
MSTRG.2010.2 : Ppp6r3 : D3ZBT9	3.21	yes	up	8.1E-07	yes	chr1	intron	internal intron	NA	0.78	no	down	0.38	no	0.98	no	down	7.0E-03	yes
MSTRG.15096.7 : Cacna1c : F1MA84	4.75	yes	up	4.3E-04	yes	chr4	intron	internal intron	NA	1.37	no	up	0.59	no	0.97	no	down	2.7E-02	yes
MSTRG.16461.3 : Pum1 : D3Z8L5	0.44	yes	down	3.1E-04	yes	chr5	intron	internal intron	NA	1.04	no	up	0.33	no	1.03	no	up	7.0E-02	no
MSTRG.12934.3 : Nmi : Q498S7	0.14	yes	down	8.2E-08	yes	chr3	exon	last exon	NA	0.81	no	down	0.37	no	1.02	no	up	4.1E-02	yes
MSTRG.11953.22 : RT1-CE7 : D3ZLE6	inf	yes	up	7.3E-07	yes	chr20	exon	internal exon	NA	1.15	no	up	0.56	no	1.22	yes	up	7.3E-16	yes
ENSRNOT00000029493 : Slc35e3 : B2GUZ8	8.31	yes	up	7.9E-07	yes	chr7	promoter	Intermediate	LCP	1.08	no	up	0.73	no	1.18	no	up	2.7E-09	yes
ENSRNOT00000013213 : Pigo : D3ZTP8	0.61	yes	down	2.7E-05	yes	chr5	exon	internal exon	NA	0.67	no	down	0.31	no	0.80	yes	down	9.0E-03	yes
MSTRG.15096.4 : Cacna1c : F1MA84	4.75	yes	up	4.3E-04	yes	chr4	intron	internal intron	NA	0.61	no	down	0.22	no	0.97	no	down	2.7E-02	yes
MSTRG.18880.3 : Plec : Q6S395	0.33	yes	down	4.2E-06	yes	chr7	exon	last exon	NA	1.02	no	up	0.96	no	1.03	no	up	2.2E-01	no
MSTRG.97.5 : Bclaf1 : B1WC16	0.67	yes	down	1.3E-07	yes	chr1	intron	internal intron	NA	0.78	no	down	0.39	no	1.03	no	up	6.8E-02	no
MSTRG.13963.1 : Ptgis : Q62969	0.55	yes	down	6.0E-05	yes	chr3	intron	internal intron	NA	1.20	no	up	0.89	no	0.99	no	down	7.4E-02	no
MSTRG.19257.1 : Lima1 : F1LR10	0.35	yes	down	4.4E-04	yes	chr7	intron	internal intron	NA	1.18	no	up	0.07	no	1.16	no	up	2.2E-10	yes
MSTRG.12655.2 : Gle1 : Q4KLN4	0.16	yes	down	1.6E-04	yes	chr3	promoter	Proximal	LCP	1.53	no	up	0.05	yes	0.95	no	down	1.3E-02	yes
ENSRNOT00000019298 : Cdh13 : Q8R490	2.07	yes	up	3.3E-06	yes	chr19	promoter	Intermediate	LCP	1.14	no	up	0.34	no	0.83	yes	down	2.3E-16	yes
ENSRNOT00000056105 : Srrm1 : B2RYB3	2.05	yes	up	2.0E-04	yes	chr5	intron	internal intron	NA	0.90	no	down	0.83	no	1.10	no	up	2.8E-06	yes
MSTRG.6191.2 : Ddr2 : B1WC09	0.49	yes	down	3.3E-05	yes	chr13	exon	internal exon	NA	0.64	no	down	0.23	no	0.95	no	down	3.2E-01	no
MSTRG.8552.1 : Ddx46 : Q62780	0.56	yes	down	3.4E-04	yes	chr17	intron	internal intron	NA	1.19	no	up	0.55	no	0.98	no	down	1.3E-01	no
MSTRG.3249.7 : Specc1 : A0A0G2K5D7	5.53	yes	up	1.9E-10	yes	chr10	exon	last exon	NA	1.81	no	up	0.30	no	0.93	no	down	4.4E-09	yes
MSTRG.19194.4 : Hdac7 : A0A0G2K6B1	1.57	yes	up	6.8E-05	yes	chr7	intron	internal intron	NA	1.03	no	up	0.20	no	0.99	no	down	6.7E-01	no
MSTRG.8561.1 : Dok3 : B2RYG7	9.95	yes	up	1.6E-09	yes	chr17	promoter	Distal	LCP	1.58	no	up	0.23	no	1.15	no	up	1.1E-05	yes
MSTRG.4717.2 : Adcy5 : G3V9G1	0.16	yes	down	2.3E-10	yes	chr11	intron	internal intron	NA	0.89	no	down	0.70	no	1.00	no	down	7.6E-01	no
MSTRG.6855.11 : Prom1 : Q9JI49	8.32	yes	up	2.4E-09	yes	chr14	intron	internal intron	NA	0.94	no	down	0.46	no	0.87	no	down	1.5E-05	yes
MSTRG.20752.7 : Ptpn18 : Q4KM54	0	yes	down	1.9E-04	yes	chr9	intron	first intron	NA	1.66	no	up	0.33	no	1.20	no	up	1.2E-08	yes
MSTRG.659.2 : Cic : D4A853	4.32	yes	up	3.8E-07	yes	chr1	exon	internal exon	NA	1.08	no	up	0.74	no	1.06	no	up	4.3E-02	yes

ENSRNOT00000024659 : Slc27a1 : Q6GMM8	0.26	yes	down	5.6E-15	yes	chr16	intron	internal intron	NA	0.60	no	down	0.18	no	0.99	no	down	2.5E-01	no
ENSRNOT00000029284 : Fgfr1 : F1LM54	0.22	yes	down	3.0E-05	yes	chr16	exon	last exon	NA	1.07	no	up	0.76	no	1.05	no	up	1.3E-02	yes
ENSRNOT00000076266 : Parp4 : A0A096MK99	1.23	yes	up	1.1E-06	yes	chr15	intron	last intron	NA	0.97	no	down	0.67	no	0.94	no	down	4.1E-06	yes
ENSRNOT00000005611 : Aldh9a1 : A0A0G2JSI1	0	yes	down	6.8E-05	yes	chr13	promoter	Distal	LCP	1.11	no	up	0.19	no	0.91	no	down	3.9E-10	yes
ENSRNOT00000057096 : Timm50 : D3ZJX5	0.54	yes	down	3.3E-07	yes	chr1	exon	last exon	NA	0.82	no	down	0.03	yes	0.98	no	down	4.2E-02	yes
MSTRG.2433.4 : Rnls : Q5U2W9	0.31	yes	down	2.4E-05	yes	chr1	promoter	Distal	LCP	1.13	no	up	0.09	no	1.02	no	up	3.0E-01	no
MSTRG.20752.2 : Ptpn18 : Q4KM54	0	yes	down	1.9E-04	yes	chr9	intron	first intron	NA	0.86	no	down	0.63	no	1.20	no	up	1.2E-08	yes
ENSRNOT00000038546 : Car13 : B5DFG6	3.23	yes	up	1.2E-05	yes	chr2	exon	last exon	NA	1.19	no	up	0.02	yes	1.15	no	up	5.8E-10	yes
MSTRG.20878.2 : Gls : A0A0G2KAN7	0.05	yes	down	1.5E-13	yes	chr9	intron	first intron	NA	0.96	no	down	0.95	no	1.09	no	up	8.3E-03	yes
MSTRG.21446.2 : Tfe3 : D3ZAW6	0.07	yes	down	2.9E-11	yes	chrX	exon	last exon	NA	0.73	no	down	0.18	no	1.04	no	up	1.6E-03	yes
MSTRG.19538.19 : Nfrkb : D4A421	0.58	yes	down	1.3E-05	yes	chr8	intron	internal intron	NA	1.01	no	up	0.98	no	0.89	no	down	2.5E-03	yes
ENSRNOT00000049756 : Lrrc57 : Q5FVI3	0.39	yes	down	6.1E-06	yes	chr3	intron	internal intron	NA	1.13	no	up	0.26	no	0.91	no	down	5.9E-09	yes
MSTRG.20350.7 : Clasp2 : A0A0G2JZM8	0.25	yes	down	1.3E-05	yes	chr8	intron	internal intron	NA	1.00	no	down	1.00	no	0.98	no	down	1.8E-01	no
ENSRNOT00000090717 : Bpgm : Q6P6G4	0.17	yes	down	4.7E-04	yes	chr4	intron	first intron	NA	1.00	no	down	0.96	no	0.88	no	down	2.8E-09	yes
MSTRG.6812.6 : Pcdh7 : Q68HB8	0	yes	down	4.2E-04	yes	chr14	exon	last exon	NA	1.63	no	up	0.09	no	0.94	no	down	1.0E-07	yes
ENSRNOT00000092730 : Tecpr1 : Q3ZBA0	0.43	yes	down	3.1E-04	yes	chr12	promoter	Intermediate	LCP	1.03	no	up	0.86	no	1.17	no	up	7.7E-08	yes
MSTRG.6572.1 : Bmp2k : F1M7M4	11.81	yes	up	5.6E-07	yes	chr14	intron	internal intron	NA	1.04	no	up	0.49	no	1.09	no	up	3.4E-07	yes
MSTRG.9889.8 : Nf1x : F2Z3R4	0.58	yes	down	1.9E-06	yes	chr19	intron	internal intron	NA	1.73	no	up	0.21	no	0.77	yes	down	2.4E-14	yes
MSTRG.2881.11 : Pkd1 : Q9ERV0	inf	yes	up	6.6E-05	yes	chr10	intron	internal intron	NA	1.70	no	up	0.03	yes	1.19	no	up	9.8E-07	yes
ENSRNOT00000028632 : Lgi4 : Q6P2A4	inf	yes	up	6.8E-05	yes	chr1	promoter	Proximal	LCP	0.75	no	down	0.08	no	0.77	yes	down	1.0E-07	yes
MSTRG.7964.2 : Bmpr1a : Q78EA7	0.29	yes	down	2.1E-04	yes	chr16	intron	internal intron	NA	0.73	no	down	0.50	no	0.90	no	down	9.8E-10	yes
ENSRNOT00000084587 : Pitpnm2 : A0A0G2JW50	2.70	yes	up	3.9E-04	yes	chr12	promoter	Distal	LCP	0.86	no	down	0.29	no	0.89	no	down	1.9E-08	yes
ENSRNOT00000055801 : Ptpra : Q03348	0.34	yes	down	1.6E-04	yes	chr3	exon	internal exon	NA	0.84	no	down	0.91	no	0.89	no	down	6.4E-12	yes
ENSRNOT00000041155 : Insr : F1LPL6	0.36	yes	down	9.6E-05	yes	chr12	intron	internal intron	NA	1.10	no	up	0.51	no	1.09	no	up	1.4E-04	yes
MSTRG.13963.4 : Ptgis : Q62969	0.55	yes	down	6.0E-05	yes	chr3	intron	internal intron	NA	2.36	yes	up	0.39	no	0.99	no	down	7.4E-02	no
MSTRG.4276.2 : Timp2 : P30121	3.20	yes	up	1.5E-04	yes	chr10	exon	first exon	NA	1.06	no	up	0.96	no	1.01	no	up	5.4E-01	no
MSTRG.11866.3 : RT1-S3 : Q9R0V1	0.16	yes	down	4.2E-07	yes	chr20	exon	last exon	NA	0.78	no	down	0.85	no	1.05	no	up	2.0E-01	no
MSTRG.7350.8 : Ktn1 : D4A4Z9	2.49	yes	up	1.2E-04	yes	chr15	intron	internal intron	NA	0.55	no	down	0.12	no	1.04	no	up	2.2E-11	yes
MSTRG.14722.1 : Dnah6 : Q63169	0.16	yes	down	4.2E-04	yes	chr4	intron	internal intron	NA	0.55	no	down	0.22	no	0.98	no	down	1.5E-01	no
MSTRG.7470.5 : Acin1 : E9PST5	0.30	yes	down	1.1E-04	yes	chr15	promoter	Distal	LCP	1.49	no	up	0.54	no	0.98	no	down	5.1E-04	yes
ENSRNOT00000020509 : Zc3h4 : D3ZVW3	2.19	yes	up	2.6E-04	yes	chr1	intron	internal intron	NA	0.56	no	down	0.19	no	0.98	no	down	3.2E-01	no
ENSRNOT00000093426 : Gpx4 : A0A0G2K398	0.42	yes	down	9.7E-05	yes	chr7	intron	internal intron	NA	0.76	no	down	0.30	no	0.91	no	down	1.6E-05	yes
ENSRNOT00000059814 : Exoc1 : A0A0G2K2V5	inf	yes	up	6.1E-05	yes	chr14	intron	first intron	NA	1.04	no	up	0.48	no	0.90	no	down	2.2E-04	yes
MSTRG.10475.3 : Tnpo1 : F1LQP9	24.87	yes	up	1.6E-05	yes	chr2	exon	internal exon	NA	1.76	no	up	0.47	no	0.98	no	down	5.8E-02	no
ENSRNOT00000091053 : Mfsd5 : A0A0G2KAK9	0.14	yes	down	6.3E-05	yes	chr7	intron	first intron	NA	1.00	no	down	0.97	no	1.12	no	up	3.3E-09	yes
MSTRG.21168.3 : Gigyf2 : A0A096MKC0	0.59	yes	down	1.9E-05	yes	chr9	intron	internal intron	NA	1.54	no	up	0.06	no	1.01	no	up	7.0E-01	no
MSTRG.2881.7 : Pkd1 : Q9ERV0	inf	yes	up	6.6E-05	yes	chr10	intron	internal intron	NA	0.70	no	down	0.76	no	1.19	no	up	9.8E-07	yes
MSTRG.4030.2 : Eftud2 : F1LM66	4.66	yes	up	6.0E-04	yes	chr10	exon	internal exon	NA	0.82	no	down	0.62	no	1.02	no	up	4.9E-02	yes
ENSRNOT00000021533 : Maip1 : Q6AY04	27.81	yes	up	1.4E-21	yes	chr9	promoter	Intermediate	ICP	1.10	no	up	0.58	no	0.87	no	down	5.5E-04	yes
ENSRNOT00000000754 : Bicc1 : A0A0G2K0Y0	5.19	yes	up	5.0E-04	yes	chr20	intron	internal intron	NA	0.95	no	down	0.82	no	1.52	yes	up	1.2E-05	yes
ENSRNOT00000002524 : Top3b : D4A9Z2	11.41	yes	up	1.2E-04	yes	chr11	intron	last intron	NA	1.19	no	up	0.54	no	0.99	no	down	6.1E-01	no
ENSRNOT00000081021 : Plec : Q6S3A0	0.33	yes	down	4.2E-06	yes	chr7	exon	last exon	NA	0.97	no	down	0.44	no	1.11	no	up	6.0E-08	yes
MSTRG.12303.2 : Lims1 : C0KUC5	12.34	yes	up	5.9E-05	yes	chr20	intron	internal intron	NA	1.21	no	up	0.26	no	0.77	yes	down	1.9E-12	yes
MSTRG.9465.3 : Dmxl1 : D4AA13	0.19	yes	down	1.3E-04	yes	chr18	intron	internal intron	NA	2.75	yes	up	0.01	yes	0.95	no	down	3.8E-03	yes
MSTRG.16935.1 : Thada : D3ZVT2	inf	yes	up	4.7E-04	yes	chr6	intron	internal intron	NA	1.00	no	down	0.39	no	0.88	no	down	1.3E-03	yes
MSTRG.7229.8 : Usp54 : Q6IE24	0.36	yes	down	1.9E-04	yes	chr15	intron	internal intron	NA	0.95	no	down	0.95	no	0.83	yes	down	7.0E-09	yes
MSTRG.13544.2 : Ptpra : Q03348	0.34	yes	down	1.6E-04	yes	chr3	exon	internal exon	NA	1.81	no	up	0.47	no	0.89	no	down	6.4E-12	yes
MSTRG.97.13 : Bclaf1 : B1WC16	0.67	yes	down	1.3E-07	yes	chr1	intron	internal intron	NA	0.96	no	down	0.89	no	1.03	no	up	6.8E-02	no
ENSRNOT00000080834 : Acap2 : Q5FVC7	0.15	yes	down	2.4E-05	yes	chr11	promoter	Intermediate	ICP	1.01	no	up	0.12	no	0.96	no	down	5.5E-04	yes
ENSRNOT00000070861 : Abcc2 : Q63120	9.17	yes	up	9.1E-13	yes	chr1	intron	internal intron	NA	1.01	no	up	0.45	no	0.90	no	down	1.2E-01	no
ENSRNOT00000092594 : Washc2c : F1LPG9	0.05	yes	down	2.7E-19	yes	chr4	exon	internal exon	NA	1.03	no	up	0.23	no	1.05	no	up	2.1E-05	yes
ENSRNOT00000067892 : Chd5 : D3ZR50	0.12	yes	down	1.1E-04	yes	chr5	intron	internal intron	NA	1.00	no	down	0.24	no	0.87	no	down	3.8E-03	yes
MSTRG.15557.5 : Ints8 : A0A0G2K0V1	0.11	yes	down	2.1E-05	yes	chr5	intron	last intron	NA	1.21	no	up	0.20	no	1.07	no	up	1.6E-05	yes
MSTRG.19963.9 : Sltm : A0A0G2K904	0.34	yes	down	6.4E-06	yes	chr8	intron	internal intron	NA	0.96	no	down	0.93	no	0.94	no	down	2.8E-02	yes
MSTRG.13684.1 : Nsfl1c : O35987	16.74	yes	up	1.8E-04	yes	chr3	intron	first intron	NA	1.14	no	up	0.23	no	0.98	no	down	1.6E-03	yes
MSTRG.1683.4 : Il4r : Q63257	9.80	yes	up	2.9E-08	yes	chr1	intron	internal intron	NA	0.80	no	down	0.26	no	1.11	no	up	2.1E-05	yes
MSTRG.13195.1 : Arhgap1 : D4A6C5	3.73	yes	up	1.8E-11	yes	chr3	intron	internal intron	NA	0.88	no	down	0.83	no	1.04	no	up	1.7E-04	yes
MSTRG.6525.1 : Aff1 : D3ZBU5	1.49	yes	up	9.8E-06	yes	chr14	intron	internal intron	NA	0.64	no	down	0.04	yes	1.07	no	up	4.0E-02	yes
ENSRNOT00000012109 : Cpz : A0A0G2JSJ7	3.01	yes	up	1.1E-05	yes	chr14	intron	internal intron	NA	0.58	no	down	0.02	yes	1.08	no	up	5.1E-02	no
MSTRG.7171.1 : Efemp1 : Q6AXN2	0.06	yes	down	2.6E-05	yes	chr14	exon	last exon	NA	0.76	no	down	0.56	no	0.94	no	down	1.1E-08	yes
ENSRNOT00000018097 : Myl1 : P02600	2.29	yes	up	4.3E-05	yes	chr9	promoter	Distal	LCP	1.59	no	up	0.09	no	1.33	yes	up	6.1E-13	yes
MSTRG.18809.3 : Trappc9 : B1H266	4.58	yes	up	1.4E-09	yes	chr7	intron	internal intron	NA	1.16	no	up	0.17	no	0.94	no	down	3.8E-02	yes
ENSRNOT00000087594 : Vars : Q04462	4.99	yes	up	5.5E-05	yes	chr20	intron	internal intron	NA	0.80	no	down	0.40	no	1.01	no	up	2.1E-04	yes
ENSRNOT00000090268 : Sltm : A0A0G2K904	0.34	yes	down	6.4E-06	yes	chr8	intron	internal intron	NA	0.88	no	down	0.29	no	0.94	no	down	2.8E-02	yes
ENSRNOT00000084906 : Camsap3 : A0A0G2K5C0	0.05	yes	down	5.3E-04	yes	chr12	intron	first intron	NA	0.98	no	down	0.50	no	0.86	no	down	3.4E-07	yes
MSTRG.6151.1 : Selp : A0A096MK10	inf	yes	up	7.4E-05	yes	chr13	promoter	Distal	LCP	1.23	no	up	0.27	no	0.93	no	down	6.5E-03	yes
MSTRG.16606.4 : Alpl : P08289	0.52	yes	down	2.4E-04	yes	chr5	promoter	Intermediate	LCP	0.84	no	down	0.86	no	1.00	no	down	6.2E-01	no
ENSRNOT00000082998 : RGD1306556 : A0A0G2JZ	0.24	yes	down	1.7E-04	yes	chr12	exon	internal exon	NA	0.81	no	down	0.66	no	1.28	yes	up	1.1E-07	yes
MSTRG.12896.6 : Rabgap1 : D3ZX42	3.82	yes	up	8.1E-06	yes	chr3	intron	internal intron	NA	0.82	no	down	0.87	no	1.01	no	up	1.9E-01	no
ENSRNOT00000090352 : Sec24c : A0A0G2JZF0	3.50	yes	up	1.3E-09	yes	chr15	intron	internal intron	NA	6.40	yes	up	0.13	no	1.01	no	up	2.6E-01	no
MSTRG.19257.4 : Lima1 : F1LR10	0.35	yes	down	4.4E-04	yes	chr7	intron	internal intron	NA	0.92	no	down	0.73	no	1.16	no	up	2.2E-10	yes
MSTRG.19173.2 : Ano6 : A0A0G2K1M7	3.87	yes	up	9.8E-09	yes	chr7	intron	internal intron	NA	0.87	no	down	0.47	no	0.91	no	down	2.1E-09	yes
MSTRG.3249.10 : Specc1 : A0A0G2K5D7	5.53	yes	up	1.9E-10	yes	chr10	exon	last exon	NA	1.16	no	up	0.48	no	0.93	no	down	4.4E-09	yes
MSTRG.20878.1 : Gls : A0A0G2KAN7	0.05	yes	down	1.5E-13	yes	chr9	intron	first intron	NA	2.11	yes	up	0.05	yes	1.09	no	up	8.3E-03	yes
MSTRG.21083.8 : Speg : Q63638	0.17	yes	down	2.6E-06	yes	chr9	intron	internal intron	NA	0.89	no	down	0.81	no	0.88	no	down	2.3E-07	yes
MSTRG.9087.1 : Pter : Q63530	5.96	yes	up	4.9E-04	yes	chr17	promoter	Distal	LCP	1.73	no	up	0.27	no	1.15	no	up	4.7E-10	yes
ENSRNOT00000027897 : Col5a3 : Q9JI04	0.25	yes	down	2.0E-05	yes	chr8	intron	internal intron	NA	0.60	no	down	0.03	yes	0.86	no	down	7.3E-07	yes
ENSRNOT00000087033 : Rbm3 : Q925G0	5.50	yes	up	1.2E-04	yes	chrX	intron	first intron	NA	0.84	no	down	0.23	no	1.28	yes	up	1.7E-10	yes
MSTRG.20154.3 : Armc8 : B4F7A2	0.25	yes	down	7.8E-05	yes	chr8	exon	first exon	NA	0.91	no	down	0.50	no	0.97	no	down	2.3E-03	yes
MSTRG.19220.13 : Kmt2d : A0A0G2JVD6	6.53	yes	up	2.1E-09	yes	chr7	exon	last exon	NA	1.10	no	up	0.83	no	1.03	no	up	3.5E-02	yes
MSTRG.12240.7 : Bicc1 : A0A0G2K0Y0	5.19	yes	up	5.0E-04	yes	chr20	intron	internal intron	NA	1.11	no	up	0.87	no	1.52	yes	up	1.2E-05	yes
ENSRNOT00000074000 : Ube3b : A0A0G2K0E7	0.36	yes	down	3.1E-06	yes	chr12	intron	internal intron	NA	0.84	no	down	0.44	no	1.15	no	up	1.8E-12	yes
MSTRG.6624.6 : Ankrd17 : D4A0B4	3.39	yes	up	8.9E-05	yes	chr14	intron	internal intron	NA	2.28	yes	up	0.10	no	1.11	no	up	1.6E-06	yes
MSTRG.12934.1 : Nmi : Q498S7	0.14	yes	down	8.2E-08	yes	chr3	exon	last exon	NA	1.00	no	down	0.97	no	1.02	no	up	4.1E-02	yes
ENSRNOT00000076167 : Asph : A0A096MKE0	2.56	yes	up	9.8E-06	yes	chr5	intron	internal intron	NA	0.99	no	down	0.91	no	0.99	no	down	1.3E-01	no
MSTRG.8345.7 : Fgfr1 : F1LM54	0.22	yes	down	3.0E-05	yes	chr16	exon	last exon	NA	2.36	yes	up	0.03	yes	1.05	no	up	1.3E-02	yes
ENSRNOT00000090402 : Actn4 : Q9QXQ0	0.43	yes	down	2.1E-04	yes	chr1	intron	internal intron	NA	0.83	no	down	0.63	no	1.01	no	up	3.9E-03	yes
ENSRNOT00000004078 : Atp2b4 : Q64542	2.31	yes	up	1.3E-05	yes	chr13	intron	internal intron	NA	1.44	no	up	0.21	no	0.68	yes	down	2.6E-19	yes
MSTRG.10589.1 : Itga1 : P18614	0.09	yes	down	9.6E-08	yes	chr2	intron	internal intron	NA	0.98	no	down	0.98	no	0.81	yes	down	3.6E-17	yes
MSTRG.17314.1 : Snx6 : B5DEY8	21.45	yes	up	3.0E-04	yes	chr6	exon	internal exon	NA	2.47	yes	up	0.06	no	0.97	no	down	1.8E-09	yes
ENSRNOT00000019959 : RGD1306148 : F1M446	0.22	yes	down	4.0E-05	yes	chr5	intron	internal intron	NA	1.81	no	up	0.61	no	1.01	no	up	7.4E-02	no
MSTRG.16324.1 : Pabpc4 : G3V9N0	15.58	yes	up	1.8E-05	yes	chr5	intron	first intron	NA	1.51	no	up	0.11	no	1.10	no	up	6.8E-12	yes
MSTRG.581.1 : Qpctl : B5DFI7	20.24	yes	up	4.4E-10	yes	chr1	intron	first intron	NA	1.54	no	up	0.56	no	1.00	no	down	6.3E-01	no
MSTRG.9775.3 : Gnao1 : P59215	0.45	yes	down	1.5E-04	yes	chr19	exon	internal exon	NA	1.05	no	up	0.75	no	0.81	yes	down	3.6E-12	yes
ENSRNOT00000018190 : Rala : P63322	0.27	yes	down	4.5E-04	yes	chr17	promoter	Intermediate	LCP	1.64	no	up	0.39	no	0.88	no	down	5.2E-09	yes
MSTRG.21122.2 : Agfg1 : F1M9N7	3.59	yes	up	1.8E-08	yes	chr9	exon	internal exon	NA	0.73	no	down	0.50	no	1.16	no	up	6.0E-06	yes
MSTRG.1683.8 : Il4r : Q63257	9.80	yes	up	2.9E-08	yes	chr1	intron	internal intron	NA	0.56	no	down	0.41	no	1.11	no	up	2.1E-05	yes
MSTRG.4038.1 : Plcd3 : D4A978	inf	yes	up	1.4E-05	yes	chr10	promoter	Distal	LCP	1.10	no	up	0.62	no	0.86	no	down	2.9E-08	yes
MSTRG.8345.14 : Fgfr1 : F1LM54	0.22	yes	down	3.0E-05	yes	chr16	exon	last exon	NA	0.64	no	down	0.29	no	1.05	no	up	1.3E-02	yes
MSTRG.20352.6 : Ubp1 : D4A030	0.44	yes	down	2.3E-04	yes	chr8	intron	internal intron	NA	0.90	no	down	0.72	no	0.98	no	down	1.9E-01	no

ENSRNOT00000005078 : Zdhhc17 : E9PTT0	5.08	yes	up	4.0E-04	yes	chr7	intron	internal intron	NA	1.04	no	up	0.58	no	0.94	no	down	1.6E-01	no
MSTRG.17738.2 : Vrk1 : Q6AYA2	0.22	yes	down	2.5E-07	yes	chr6	intron	internal intron	NA	1.44	no	up	0.51	no	1.01	no	up	2.7E-01	no
MSTRG.12137.5 : Pknox1 : Q5BJP1	0.26	yes	down	3.4E-07	yes	chr20	promoter	Proximal	LCP	0.80	no	down	0.52	no	0.89	no	down	7.5E-04	yes
MSTRG.21491.2 : Phf8 : D4AD31	0	yes	down	2.1E-07	yes	chrX	exon	first exon	NA	0.88	no	down	0.91	no	1.13	no	up	3.6E-02	yes
MSTRG.19777.6 : Sin3a : A0A0G2K3H5	0.53	yes	down	4.1E-06	yes	chr8	intron	last intron	NA	0.78	no	down	0.45	no	0.96	no	down	6.2E-05	yes
MSTRG.21175.4 : Inpp5d : F1M981	0.08	yes	down	9.9E-05	yes	chr9	intron	internal intron	NA	0.58	no	down	0.02	yes	1.08	no	up	3.1E-05	yes
ENSRNOT00000005155 : Sod3 : Q08420	0.92	no	down	2.3E-05	yes	chr14	exon	last exon	NA	6.71	yes	up	0.35	no	1.18	no	up	8.5E-11	yes
MSTRG.19956.5 : Myo1e : Q63356	3.15	yes	up	9.4E-05	yes	chr8	intron	first intron	NA	0.37	yes	down	0.20	no	1.03	no	up	1.7E-02	yes
ENSRNOT00000029878 : Ptprc : P04157	2.98	yes	up	6.9E-14	yes	chr13	intron	internal intron	NA	4.37	yes	up	0.08	no	1.12	no	up	6.1E-15	yes
MSTRG.17673.1 : Ppp4r3a : D4ABC4	3.37	yes	up	4.5E-04	yes	chr6	intron	internal intron	NA	0.72	no	down	0.06	no	1.03	no	up	5.4E-03	yes
ENSRNOT00000025795 : Syne1 : Q8VHJ9	0.04	yes	down	1.5E-19	yes	chr1	exon	first exon	NA	1.02	no	up	0.78	no	0.89	no	down	8.9E-14	yes
ENSRNOT00000017361 : Rbl2 : G3V7P7	0.41	yes	down	2.1E-05	yes	chr19	intron	internal intron	NA	1.21	no	up	0.38	no	0.89	no	down	1.5E-05	yes
ENSRNOT00000012376 : Kpnb1 : F2Z3Q8	17.89	yes	up	1.8E-04	yes	chr10	intron	internal intron	NA	1.60	no	up	0.26	no	0.97	no	down	1.9E-07	yes
MSTRG.1326.1 : Picalm : Q66WT9	8.74	yes	up	2.1E-13	yes	chr1	intron	internal intron	NA	3.98	yes	up	0.01	yes	1.45	yes	up	1.6E-10	yes
MSTRG.8514.4 : Zcchc6 : D3ZKR9	0.14	yes	down	1.2E-08	yes	chr17	exon	internal exon	NA	0.81	no	down	0.03	yes	1.12	no	up	2.3E-06	yes
MSTRG.5704.3 : RGD1306556 : A0A0G2JZ88	0.24	yes	down	1.7E-04	yes	chr12	exon	internal exon	NA	3.82	yes	up	0.05	yes	1.28	yes	up	1.1E-07	yes
MSTRG.5906.10 : Atp2b4 : Q64542	2.31	yes	up	1.3E-05	yes	chr13	intron	internal intron	NA	1.10	no	up	0.69	no	0.68	yes	down	2.6E-19	yes
ENSRNOT00000025325 : Wdr7 : Q9ERH3	0.16	yes	down	4.0E-04	yes	chr18	promoter	Distal	LCP	1.10	no	up	0.83	no	0.99	no	down	6.5E-01	no
ENSRNOT00000086365 : Recql : Q6AYJ1	0.74	yes	down	1.2E-04	yes	chr4	exon	last exon	NA	1.28	no	up	0.16	no	0.97	no	down	6.1E-03	yes
MSTRG.13928.1 : Pltp : E9PSP1	0.31	yes	down	3.6E-05	yes	chr3	intron	internal intron	NA	2.30	yes	up	0.26	no	0.93	no	down	1.9E-11	yes
MSTRG.11953.35 : RT1-CE7 : D3ZLE6	inf	yes	up	7.3E-07	yes	chr20	exon	internal exon	NA	1.10	no	up	0.76	no	1.22	yes	up	7.3E-16	yes
MSTRG.15413.1 : Ipo8 : A0A0G2K6J6	3.66	yes	up	4.4E-04	yes	chr4	intron	internal intron	NA	1.24	no	up	0.23	no	1.07	no	up	4.0E-07	yes
ENSRNOT00000052017 : Cacna1c : F1MA84	4.75	yes	up	4.3E-04	yes	chr4	intron	internal intron	NA	1.30	no	up	0.51	no	0.97	no	down	2.7E-02	yes
MSTRG.479.1 : Epn1 : O88339	2.09	yes	up	1.9E-04	yes	chr1	intron	internal intron	NA	1.11	no	up	0.64	no	0.99	no	down	3.7E-01	no
MSTRG.9433.1 : Rbm27 : F1M1R4	0.45	yes	down	2.5E-04	yes	chr18	intron	internal intron	NA	1.21	no	up	0.43	no	1.01	no	up	5.2E-01	no
ENSRNOT00000000155 : Plxdc2 : B5DEZ8	3.07	yes	up	1.1E-04	yes	chr17	intron	internal intron	NA	0.89	no	down	0.25	no	0.90	no	down	1.5E-10	yes
ENSRNOT00000017633 : Alox5 : F1LMM5	0.38	yes	down	1.3E-04	yes	chr4	intron	internal intron	NA	1.13	no	up	0.40	no	0.96	no	down	9.7E-06	yes
ENSRNOT00000084217 : Dusp3 : B5DFF7	0.16	yes	down	1.6E-06	yes	chr10	exon	last exon	NA	1.39	no	up	0.13	no	0.90	no	down	6.2E-11	yes
ENSRNOT00000080784 : Hdhd2 : Q6QI86	0.20	yes	down	5.1E-06	yes	chr18	intron	first intron	NA	1.39	no	up	0.49	no	1.04	no	up	3.3E-03	yes
MSTRG.10007.1 : Ctcf : Q9R1D1	2.29	yes	up	1.4E-04	yes	chr19	intron	internal intron	NA	1.16	no	up	0.08	no	0.94	no	down	4.5E-07	yes
ENSRNOT00000067591 : Ccdc88a : D3ZYD7	6.58	yes	up	8.2E-05	yes	chr14	promoter	Distal	LCP	0.63	no	down	0.17	no	1.03	no	up	5.3E-03	yes
MSTRG.15546.9 : Asph : A0A096MKE0	2.56	yes	up	9.8E-06	yes	chr5	intron	internal intron	NA	1.00	no	down	0.98	no	0.99	no	down	1.3E-01	no
ENSRNOT00000026925 : Folr2 : D4A4S5	0.21	yes	down	8.7E-05	yes	chr1	promoter	Distal	LCP	0.91	no	down	0.91	no	1.42	yes	up	6.4E-18	yes
ENSRNOT00000050342 : Dyrk1a : Q63470	0.26	yes	down	2.2E-05	yes	chr11	intron	internal intron	NA	1.23	no	up	0.47	no	1.02	no	up	1.6E-01	no
MSTRG.18880.6 : Plec : Q6S3A0	0.33	yes	down	4.2E-06	yes	chr7	exon	last exon	NA	0.59	no	down	0.48	no	1.11	no	up	6.0E-08	yes
MSTRG.3895.3 : Cdk12 : A0A0G2K5U7	5.94	yes	up	1.1E-04	yes	chr10	exon	first exon	NA	1.32	no	up	0.25	no	0.97	no	down	5.5E-04	yes
MSTRG.8094.4 : Atp13a1 : B5DEX7	3.83	yes	up	1.1E-13	yes	chr16	intron	internal intron	NA	1.13	no	up	0.75	no	1.09	no	up	4.2E-12	yes
ENSRNOT00000076099 : Gigyf2 : A0A096MJI4	0.59	yes	down	1.9E-05	yes	chr9	intron	internal intron	NA	0.83	no	down	0.06	no	1.31	yes	up	2.3E-10	yes
ENSRNOT00000066119 : Dpt : B2RZ77	6.14	yes	up	2.8E-04	yes	chr13	exon	last exon	NA	0.79	no	down	0.24	no	0.85	no	down	3.5E-10	yes
MSTRG.14075.2 : Dnajc5 : A0A0G2JX56	0.30	yes	down	1.1E-06	yes	chr3	promoter	Intermediate	HCP	1.20	no	up	0.71	no	0.89	no	down	4.0E-08	yes
MSTRG.21168.1 : Gigyf2 : A0A096MJI4	0.59	yes	down	1.9E-05	yes	chr9	intron	internal intron	NA	1.30	no	up	0.36	no	1.31	yes	up	2.3E-10	yes
ENSRNOT00000080999 : Ctnna1 : Q5U302	2.58	yes	up	1.7E-05	yes	chr18	intron	internal intron	NA	1.02	no	up	0.89	no	0.96	no	down	3.1E-09	yes
MSTRG.2839.13 : Srrm2 : A0A0G2K2M9	1.72	yes	up	9.0E-05	yes	chr10	exon	internal exon	NA	0.81	no	down	0.69	no	1.00	no	up	9.0E-01	no
MSTRG.13841.1 : Rprd1b : B5DEK0	0.05	yes	down	1.1E-04	yes	chr3	intron	last intron	NA	0.68	no	down	0.44	no	0.98	no	down	1.8E-03	yes
MSTRG.323.10 : LOC108348175 : A0A0G2JUS0	0.05	yes	down	4.0E-19	yes	chr1	promoter	Intermediate	LCP	0.99	no	down	0.97	no	1.01	no	up	4.0E-01	no
MSTRG.12240.4 : Bicc1 : A0A0G2K0Y0	5.19	yes	up	5.0E-04	yes	chr20	intron	internal intron	NA	0.61	no	down	0.50	no	1.52	yes	up	1.2E-05	yes
ENSRNOT00000052317 : RGD1310507 : A0A0G2K	10.49	yes	up	4.2E-04	yes	chr8	intron	internal intron	NA	0.98	no	down	0.49	no	1.03	no	up	1.5E-02	yes
MSTRG.22038.2 : Fmr1 : Q80WE1	inf	yes	up	3.0E-04	yes	chrX	intron	internal intron	NA	0.97	no	down	0.95	no	1.09	no	up	7.3E-06	yes
ENSRNOT00000072628 : Nup214 : D4ACK1	0.13	yes	down	4.7E-04	yes	chr3	intron	internal intron	NA	0.98	no	down	0.86	no	0.99	no	down	6.9E-01	no
MSTRG.10128.2 : Terf2ip : Q5EAN7	6.13	yes	up	7.3E-09	yes	chr19	intron	first intron	NA	0.20	yes	down	0.23	no	1.01	no	up	4.8E-01	no
ENSRNOT00000092535 : Shank3 : A0A0U1RS13	3.23	yes	up	4.3E-05	yes	chr7	exon	first exon	NA	0.67	no	down	0.09	no	0.98	no	down	1.0E-02	yes
MSTRG.21040.2 : Tns1 : F1LN42	0.09	yes	down	2.0E-04	yes	chr9	intron	last intron	NA	0.80	no	down	0.48	no	0.86	no	down	7.8E-19	yes
MSTRG.10163.2 : Cdh13 : Q8R490	2.07	yes	up	3.3E-06	yes	chr19	promoter	Intermediate	LCP	0.54	no	down	0.40	no	0.83	yes	down	2.3E-16	yes
ENSRNOT00000070994 : Rbm15 : M0R3Z8	4.02	yes	up	1.9E-06	yes	chr2	exon	last exon	NA	0.98	no	down	0.87	no	0.94	no	down	2.6E-06	yes
MSTRG.20831.4 : Tgfbrap1 : D3ZXT8	0.12	yes	down	2.5E-04	yes	chr9	intron	internal intron	NA	1.10	no	up	0.78	no	0.98	no	down	2.9E-01	no
ENSRNOT00000040808 : Tpm1 : A0A0G2JSQ4	9.41	yes	up	5.1E-14	yes	chr8	intron	internal intron	NA	0.56	no	down	0.42	no	0.81	yes	down	2.5E-10	yes
MSTRG.2839.2 : Srrm2 : A0A0G2K2M9	1.72	yes	up	9.0E-05	yes	chr10	exon	internal exon	NA	1.53	no	up	0.65	no	1.00	no	up	9.0E-01	no
MSTRG.7561.4 : Phf11b : M0RB46	3.03	yes	up	2.9E-04	yes	chr15	promoter	Intermediate	LCP	1.25	no	up	0.68	no	1.15	no	up	2.3E-06	yes
MSTRG.10569.7 : Plpp1 : O08564	0.47	yes	down	8.3E-05	yes	chr2	intron	internal intron	NA	1.71	no	up	0.05	yes	1.04	no	up	2.9E-03	yes
MSTRG.15855.7 : RGD1306148 : F1M446	0.22	yes	down	4.0E-05	yes	chr5	intron	internal intron	NA	1.61	no	up	0.47	no	1.01	no	up	7.4E-02	no
MSTRG.17118.1 : Atad2b : A0A096MKA5	3.51	yes	up	9.0E-07	yes	chr6	intron	internal intron	NA	1.13	no	up	0.67	no	1.15	no	up	9.1E-06	yes
MSTRG.11265.15 : Ubap2l : E9PTR4	6.62	yes	up	4.0E-04	yes	chr2	intron	internal intron	NA	0.91	no	down	0.76	no	1.13	no	up	3.8E-12	yes
MSTRG.19764.1 : Pstpip1 : B0BNK4	0.47	yes	down	3.6E-05	yes	chr8	intron	internal intron	NA	1.12	no	up	0.67	no	1.20	yes	up	1.1E-06	yes
MSTRG.19194.1 : Hdac7 : A0A0G2K6B1	1.57	yes	up	6.8E-05	yes	chr7	intron	internal intron	NA	0.47	yes	down	0.06	no	0.99	no	down	6.7E-01	no
MSTRG.16461.6 : Pum1 : D3Z8L5	0.44	yes	down	3.1E-04	yes	chr5	intron	internal intron	NA	1.01	no	up	0.96	no	1.03	no	up	7.0E-02	no
MSTRG.16324.2 : Pabpc4 : G3V9N0	15.58	yes	up	1.8E-05	yes	chr5	intron	first intron	NA	1.27	no	up	0.23	no	1.10	no	up	6.8E-12	yes
ENSRNOT00000004762 : Fmo2 : G3V6F6	2.66	yes	up	2.1E-11	yes	chr13	intron	internal intron	NA	0.84	no	down	0.64	no	1.21	yes	up	2.2E-15	yes
ENSRNOT00000090026 : Gls : P13264	0.05	yes	down	1.5E-13	yes	chr9	intron	first intron	NA	0.99	no	down	0.99	no	1.08	no	up	4.1E-02	yes
ENSRNOT00000004780 : Ephx1 : P07687	0.19	yes	down	2.9E-07	yes	chr13	promoter	Intermediate	LCP	0.92	no	down	0.69	no	0.69	yes	down	5.2E-14	yes
ENSRNOT00000011682 : Rbms1 : A0A0G2K4R7	0	yes	down	3.3E-05	yes	chr3	intron	internal intron	NA	0.81	no	down	0.38	no	0.97	no	down	1.2E-01	no
MSTRG.2510.3 : Tm9sf3 : D3ZUD8	4.98	yes	up	1.2E-05	yes	chr1	promoter	Intermediate	LCP	0.47	yes	down	0.50	no	1.06	no	up	1.3E-07	yes
MSTRG.21122.6 : Agfg1 : F1M9N7	3.59	yes	up	1.8E-08	yes	chr9	exon	internal exon	NA	1.29	no	up	0.55	no	1.16	no	up	6.0E-06	yes
ENSRNOT00000082999 : Ppp1r18 : A0A0G2K8B3	0.32	yes	down	2.0E-04	yes	chr20	promoter	Distal	LCP	0.42	yes	down	0.50	no	1.10	no	up	1.1E-05	yes
MSTRG.6818.1 : Rbpj : M0R7Q3	0.34	yes	down	7.0E-05	yes	chr14	intron	last intron	NA	1.13	no	up	0.87	no	1.08	no	up	1.1E-04	yes
MSTRG.16416.2 : Ak2 : A0A0G2JSG6	inf	yes	up	6.9E-05	yes	chr5	exon	first exon	NA	1.12	no	up	0.84	no	0.98	no	down	1.2E-04	yes
MSTRG.16327.1 : Macf1 : A0A0G2K9T4	0.36	yes	down	1.3E-05	yes	chr5	exon	last exon	NA	1.37	no	up	0.70	no	0.97	no	down	9.0E-11	yes
MSTRG.5417.4 : Auts2 : F1M388	0.04	yes	down	4.8E-14	yes	chr12	intron	internal intron	NA	1.28	no	up	0.35	no	1.01	no	up	5.6E-01	no
ENSRNOT00000035770 : Rbbp6 : G3V953	inf	yes	up	4.6E-05	yes	chr1	intron	internal intron	NA	1.56	no	up	0.06	no	1.09	no	up	2.1E-04	yes
MSTRG.11223.1 : Fdps : F1LND7	inf	yes	up	2.2E-05	yes	chr2	exon	last exon	NA	2.56	yes	up	0.03	yes	1.07	no	up	1.2E-03	yes
MSTRG.18557.3 : Mon2 : D3ZCG3	0.07	yes	down	1.5E-08	yes	chr7	intron	internal intron	NA	0.95	no	down	0.87	no	1.05	no	up	7.1E-07	yes
MSTRG.9721.3 : Cdh11 : F1MAH6	1.94	yes	up	2.2E-05	yes	chr19	exon	last exon	NA	1.80	no	up	0.46	no	0.91	no	down	6.5E-11	yes
MSTRG.20352.8 : Ubp1 : D4A030	0.44	yes	down	2.3E-04	yes	chr8	intron	internal intron	NA	0.86	no	down	0.36	no	0.98	no	down	1.9E-01	no
ENSRNOT00000068021 : Dcdc2 : D3ZR10	5.48	yes	up	1.6E-11	yes	chr17	exon	last exon	NA	0.97	no	down	0.20	no	0.87	no	down	5.6E-02	no
MSTRG.4725.4 : Kalrn : P97924	1.54	yes	up	9.1E-07	yes	chr11	exon	last exon	NA	1.21	no	up	0.48	no	1.04	no	up	1.6E-04	yes
ENSRNOT00000002530 : Ppm1f : Q9WVR7	1.80	yes	up	3.8E-11	yes	chr11	intron	internal intron	NA	2.49	yes	up	0.06	no	0.91	no	down	2.5E-06	yes
MSTRG.16636.7 : Ubr4 : A0A0G2JU89	2.84	yes	up	3.7E-05	yes	chr5	intron	internal intron	NA	2.26	yes	up	0.41	no	1.02	no	up	4.6E-05	yes
MSTRG.11265.24 : Ubap2l : E9PTR4	6.62	yes	up	4.0E-04	yes	chr2	intron	internal intron	NA	0.95	no	down	0.64	no	1.13	no	up	3.8E-12	yes
MSTRG.6812.8 : Pcdh7 : Q68HB8	0	yes	down	4.2E-04	yes	chr14	exon	last exon	NA	0.81	no	down	0.07	no	0.94	no	down	1.0E-07	yes
MSTRG.2160.1 : Rasgrp2 : P0C643	0.26	yes	down	2.6E-04	yes	chr1	exon	internal exon	NA	1.32	no	up	0.68	no	1.04	no	up	7.0E-02	no
MSTRG.3136.3 : Anxa6 : Q6IMZ3	2.83	yes	up	1.2E-05	yes	chr10	intron	internal intron	NA	0.93	no	down	0.83	no	0.84	no	down	6.6E-16	yes
ENSRNOT00000043517 : Chchd4 : Q5BJN5	1.88	yes	up	1.1E-04	yes	chr1	exon	first exon	NA	1.58	no	up	0.09	no	1.04	no	up	1.2E-04	yes
MSTRG.4751.3 : Pcyt1a : P19836	0.29	yes	down	1.2E-05	yes	chr11	exon	first exon	NA	0.70	no	down	0.40	no	0.94	no	down	3.3E-07	yes
MSTRG.8345.3 : Fgfr1 : F1LM54	0.22	yes	down	3.0E-05	yes	chr16	exon	last exon	NA	1.17	no	up	0.81	no	1.05	no	up	1.3E-02	yes
MSTRG.19089.1 : Scube1 : F1M987	2.13	yes	up	1.3E-04	yes	chr7	intron	internal intron	NA	0.80	no	down	0.76	no	0.80	yes	down	1.2E-14	yes
MSTRG.17487.1 : Mpp5 : B4F7E7	0.37	yes	down	1.0E-04	yes	chr6	exon	internal exon	NA	0.80	no	down	0.21	no	0.99	no	down	5.9E-01	no
ENSRNOT00000084044 : Scamp1 : A0A0G2K1I6	11.34	yes	up	2.9E-04	yes	chr2	intron	first intron	NA	0.66	no	down	0.15	no	1.05	no	up	3.2E-06	yes
MSTRG.11642.2 : Tifa : Q5XIB9	0.42	yes	down	9.5E-05	yes	chr2	exon	internal exon	NA	1.32	no	up	0.30	no	1.16	no	up	2.8E-04	yes
MSTRG.4699.1 : Kpna1 : P83953	32	yes	up	7.2E-05	yes	chr11	promoter	Distal	LCP	0.80	no	down	0.83	no	1.11	no	up	5.7E-08	yes
MSTRG.3572.1 : RGD1307929 : F1LSX1	7.34	yes	up	1.1E-12	yes	chr10	intron	internal intron	NA	1.22	no	up	0.06	no	0.97	no	down	1.8E-04	yes
ENSRNOT00000064798 : Map4k2 : D3ZXB1	4.89	yes	up	3.4E-06	yes	chr1	intron	internal intron	NA	1.29	no	up	0.08	no	0.65	yes	down	2.4E-14	yes

MSTRG.18268.25 : Akap8 : Q63014	0.08	yes	down	2.7E-04	yes	chr7	intron	internal intron	NA	1.12	no	up	0.21	no	0.99	no	down	2.8E-01	no
MSTRG.21039.11 : Tns1 : F1LN42	0.09	yes	down	2.0E-04	yes	chr9	intron	last intron	NA	0.45	yes	down	0.43	no	0.86	no	down	7.8E-19	yes
MSTRG.7514.1 : Nfatc4 : A0A0G2K0L1	0	yes	down	3.3E-04	yes	chr15	intron	internal intron	NA	0.97	no	down	0.72	no	1.13	no	up	1.3E-11	yes
MSTRG.16339.1 : Fhl3 : D3ZPF0	0.20	yes	down	2.5E-09	yes	chr5	exon	last exon	NA	1.15	no	up	0.57	no	1.28	yes	up	1.8E-14	yes
MSTRG.20764.2 : Uggt1 : Q9JLA3	0.22	yes	down	3.9E-04	yes	chr9	intron	internal intron	NA	1.02	no	up	0.98	no	1.11	no	up	9.8E-15	yes
MSTRG.15110.2 : Wnk1 : Q9JIH7	18.77	yes	up	1.6E-38	yes	chr4	exon	internal exon	NA	0.77	no	down	0.36	no	1.04	no	up	1.7E-03	yes
MSTRG.12945.5 : Fmnl2 : A0A0G2K132	0.84	no	down	6.1E-07	yes	chr3	intron	internal intron	NA	1.02	no	up	0.94	no	1.00	no	down	9.6E-01	no
MSTRG.14075.6 : Dnajc5 : A0A0G2JX56	0.30	yes	down	1.1E-06	yes	chr3	promoter	Intermediate	HCP	1.01	no	up	0.96	no	0.89	no	down	4.0E-08	yes
ENSRNOT00000064025 : Itpr1 : A0A0A0MY31	3.16	yes	up	1.1E-05	yes	chr4	intron	internal intron	NA	0.39	yes	down	0.40	no	0.87	no	down	1.1E-14	yes
MSTRG.4508.1 : Dyrk1a : Q63470	0.26	yes	down	2.2E-05	yes	chr11	intron	internal intron	NA	2.02	yes	up	0.09	no	1.02	no	up	1.6E-01	no
MSTRG.7830.8 : Dock9 : F1LSM8	0.10	yes	down	2.7E-05	yes	chr15	intron	internal intron	NA	0.56	no	down	0.22	no	0.97	no	down	2.3E-04	yes
ENSRNOT00000066440 : Cab39 : A0A0G2JZH0	39.86	yes	up	1.2E-27	yes	chr9	promoter	Intermediate	LCP	0.67	no	down	0.22	no	0.99	no	down	5.6E-01	no
ENSRNOT00000083159 : Rnaseh2a : Q5U209	14.16	yes	up	1.1E-05	yes	chr19	intron	last intron	NA	0.56	no	down	0.03	yes	1.08	no	up	2.6E-05	yes
MSTRG.11223.6 : Fdps : F1LND7	inf	yes	up	2.2E-05	yes	chr2	exon	last exon	NA	0.71	no	down	0.47	no	1.07	no	up	1.2E-03	yes
ENSRNOT00000076560 : Dync1li2 : Q5D023	0.17	yes	down	2.9E-04	yes	chr19	intron	last intron	NA	0.91	no	down	0.61	no	0.99	no	down	1.1E-01	no
MSTRG.5482.23 : Ncor2 : A0A0G2JU91	2.16	yes	up	1.6E-06	yes	chr12	intron	internal intron	NA	1.24	no	up	0.60	no	1.01	no	up	7.1E-01	no
ENSRNOT00000080682 : Specc1 : A0A0G2K5D7	5.53	yes	up	1.9E-10	yes	chr10	exon	last exon	NA	0.58	no	down	0.19	no	0.93	no	down	4.4E-09	yes
MSTRG.4992.7 : Top3b : D4A9Z2	11.41	yes	up	1.2E-04	yes	chr11	intron	last intron	NA	0.61	no	down	0.45	no	0.99	no	down	6.1E-01	no
ENSRNOT00000080846 : Bicc1 : A0A0G2K0Y0	5.19	yes	up	5.0E-04	yes	chr20	intron	internal intron	NA	0.82	no	down	0.81	no	1.52	yes	up	1.2E-05	yes
ENSRNOT00000088807 : Lrba : A0A0G2JYI0	10.95	yes	up	4.8E-12	yes	chr2	intron	internal intron	NA	0.90	no	down	0.78	no	0.94	no	down	4.5E-07	yes
MSTRG.15469.4 : Tram1 : Q5XI41	3.29	yes	up	2.9E-05	yes	chr5	exon	last exon	NA	0.52	no	down	0.50	no	1.13	no	up	1.2E-09	yes
MSTRG.7561.3 : Phf11b : M0RB46	3.03	yes	up	2.9E-04	yes	chr15	promoter	Intermediate	LCP	0.84	no	down	0.18	no	1.15	no	up	2.3E-06	yes
MSTRG.4525.5 : Mx1 : Q499S4	3.65	yes	up	1.1E-13	yes	chr11	intron	internal intron	NA	4.56	yes	up	0.03	yes	1.36	yes	up	1.2E-13	yes
MSTRG.97.4 : Bclaf1 : B1WC16	0.67	yes	down	1.3E-07	yes	chr1	intron	internal intron	NA	1.40	no	up	0.12	no	1.03	no	up	6.8E-02	no
MSTRG.18046.6 : Pan2 : R9PXX6	2.14	yes	up	4.2E-04	yes	chr7	exon	internal exon	NA	0.62	no	down	0.28	no	0.84	no	down	1.2E-03	yes
MSTRG.10325.1 : Cast : F1LPH1	0.36	yes	down	1.6E-06	yes	chr2	intron	internal intron	NA	1.07	no	up	0.84	no	0.95	no	down	2.4E-10	yes
ENSRNOT00000073955 : Apoo : M0R7G4	22.85	yes	up	2.9E-05	yes	chrX	intron	first intron	NA	0.85	no	down	0.74	no	0.95	no	down	2.3E-02	yes
MSTRG.8514.10 : Zcchc6 : D3ZKR9	0.14	yes	down	1.2E-08	yes	chr17	exon	internal exon	NA	1.25	no	up	0.48	no	1.12	no	up	2.3E-06	yes
ENSRNOT00000064785 : Ptprc : P04157	2.98	yes	up	6.9E-14	yes	chr13	intron	internal intron	NA	1.20	no	up	0.75	no	1.12	no	up	6.1E-15	yes
MSTRG.9775.1 : Gnao1 : P59215	0.45	yes	down	1.5E-04	yes	chr19	exon	internal exon	NA	0.69	no	down	0.16	no	0.81	yes	down	3.6E-12	yes
MSTRG.20927.4 : Aox1 : F1LRQ1	0.36	yes	down	1.5E-07	yes	chr9	intron	internal intron	NA	0.90	no	down	0.81	no	0.73	yes	down	3.5E-17	yes
MSTRG.7830.6 : Dock9 : F1LSM8	0.10	yes	down	2.7E-05	yes	chr15	intron	internal intron	NA	0.82	no	down	0.68	no	0.97	no	down	2.3E-04	yes
MSTRG.6685.3 : Exoc1 : Q4V8H2	inf	yes	up	6.1E-05	yes	chr14	intron	first intron	NA	0.68	no	down	0.42	no	0.91	no	down	3.2E-04	yes
MSTRG.12973.9 : Rbms1 : A0A0G2K4R7	0	yes	down	3.3E-05	yes	chr3	intron	internal intron	NA	0.84	no	down	0.52	no	0.97	no	down	1.2E-01	no
MSTRG.13391.3 : Lrrc57 : Q5FVI3	0.39	yes	down	6.1E-06	yes	chr3	intron	internal intron	NA	0.90	no	down	0.75	no	0.91	no	down	5.9E-09	yes
MSTRG.11405.1 : Pde4dip : A0A0G2JW66	6.94	yes	up	5.0E-08	yes	chr2	exon	internal exon	NA	2.31	yes	up	0.01	yes	1.06	no	up	5.1E-04	yes
MSTRG.11985.2 : Vars : Q04462	4.99	yes	up	5.5E-05	yes	chr20	intron	internal intron	NA	1.34	no	up	0.25	no	1.01	no	up	2.1E-04	yes
MSTRG.13080.1 : Lnpk : A0JN29	3.42	yes	up	1.2E-11	yes	chr3	intron	internal intron	NA	1.00	no	down	1.00	no	0.96	no	down	2.6E-04	yes
MSTRG.8552.10 : Ddx46 : Q62780	0.56	yes	down	3.4E-04	yes	chr17	intron	internal intron	NA	1.07	no	up	0.81	no	0.98	no	down	1.3E-01	no
ENSRNOT00000031365 : Creb5 : A0A0G2K5F0	0.10	yes	down	1.8E-23	yes	chr4	intron	internal intron	NA	0.98	no	down	0.86	no	1.01	no	up	7.4E-01	no
MSTRG.15089.4 : Csgalnact2 : D4A5Z0	2.74	yes	up	6.9E-07	yes	chr4	intron	internal intron	NA	0.86	no	down	0.45	no	0.94	no	down	1.2E-02	yes
ENSRNOT00000085259 : Ppp1r18 : A0A0G2K8B3	0.32	yes	down	2.0E-04	yes	chr20	promoter	Distal	LCP	1.11	no	up	0.74	no	1.10	no	up	1.1E-05	yes
MSTRG.4117.1 : Nol11 : A0A0G2K1A9	0.21	yes	down	9.6E-05	yes	chr10	intron	internal intron	NA	1.08	no	up	0.60	no	1.06	no	up	1.9E-03	yes
MSTRG.19472.5 : Dnm2 : A0A0A0MY48	0.11	yes	down	8.9E-11	yes	chr8	intron	first intron	NA	1.09	no	up	0.64	no	1.05	no	up	2.0E-08	yes
MSTRG.11265.7 : Ubap2l : E9PTR4	6.62	yes	up	4.0E-04	yes	chr2	intron	internal intron	NA	2.09	yes	up	0.18	no	1.13	no	up	3.8E-12	yes
MSTRG.10878.1 : Anxa5 : P14668	3.34	yes	up	1.2E-08	yes	chr2	promoter	Distal	LCP	0.47	yes	down	0.36	no	0.88	no	down	2.2E-14	yes
ENSRNOT00000093270 : Nup214 : D4ACK1	0.13	yes	down	4.7E-04	yes	chr3	intron	internal intron	NA	0.99	no	down	0.21	no	0.99	no	down	6.9E-01	no
ENSRNOT00000000733 : Fyn : Q62844	0.38	yes	down	2.4E-05	yes	chr20	exon	last exon	NA	1.06	no	up	0.82	no	1.01	no	up	5.2E-01	no
MSTRG.3379.1 : Dlg4 : P31016	2.15	yes	up	8.6E-07	yes	chr10	intron	last intron	NA	1.39	no	up	0.09	no	0.97	no	down	9.1E-02	no
ENSRNOT00000026643 : Vwf : F5XVC1	0.45	yes	down	3.0E-05	yes	chr4	intron	internal intron	NA	0.53	no	down	0.03	yes	0.74	yes	down	1.0E-18	yes
MSTRG.5509.5 : Pitpnm2 : A0A0G2JW50	2.70	yes	up	3.9E-04	yes	chr12	promoter	Distal	LCP	0.70	no	down	0.40	no	0.89	no	down	1.9E-08	yes
ENSRNOT00000010127 : C1qtnf5 : Q5FVH0	0.32	yes	down	9.8E-07	yes	chr8	exon	last exon	NA	1.10	no	up	0.89	no	0.96	no	down	2.5E-05	yes
ENSRNOT00000050980 : Fgfr1 : F1LM54	0.22	yes	down	3.0E-05	yes	chr16	exon	last exon	NA	1.36	no	up	0.29	no	1.05	no	up	1.3E-02	yes
ENSRNOT00000093297 : Mycbp2 : A0A1W2Q6I3	0.31	yes	down	9.2E-06	yes	chr15	intron	internal intron	NA	1.09	no	up	0.59	no	1.00	no	up	8.8E-01	no
MSTRG.1668.4 : Prkcb : A0A0G2K5Q0	3.82	yes	up	4.6E-04	yes	chr1	intron	internal intron	NA	0.92	no	down	0.84	no	1.10	no	up	1.7E-07	yes
MSTRG.19538.5 : Nfrkb : D4A421	0.58	yes	down	1.3E-05	yes	chr8	intron	internal intron	NA	0.82	no	down	0.57	no	0.89	no	down	2.5E-03	yes
ENSRNOT00000023302 : Myh6 : G3V885	2.81	yes	up	4.8E-05	yes	chr15	intron	internal intron	NA	0.96	no	down	0.31	no	0.68	yes	down	6.8E-16	yes
MSTRG.2768.3 : Usp7 : F1LM09	inf	yes	up	5.0E-04	yes	chr10	intron	internal intron	NA	0.60	no	down	0.34	no	1.00	no	down	7.8E-01	no
MSTRG.2579.5 : Sfxn3 : Q9JHY2	0.39	yes	down	7.8E-05	yes	chr1	intron	internal intron	NA	0.48	yes	down	0.08	no	0.98	no	down	7.5E-03	yes
MSTRG.12201.1 : Gstt3 : D3Z8I7	0.44	yes	down	5.4E-04	yes	chr20	promoter	Intermediate	LCP	0.83	no	down	0.69	no	0.92	no	down	4.5E-04	yes
ENSRNOT00000088624 : Nedd4l : F1LRN8	2.03	yes	up	3.6E-05	yes	chr18	intron	internal intron	NA	0.98	no	down	0.36	no	0.95	no	down	3.9E-02	yes
MSTRG.12896.7 : Rabgap1 : D3ZX42	3.82	yes	up	8.1E-06	yes	chr3	intron	internal intron	NA	1.10	no	up	0.86	no	1.01	no	up	1.9E-01	no
MSTRG.21367.1 : Uba1 : Q5U300	0	yes	down	1.9E-08	yes	chrX	exon	last exon	NA	1.28	no	up	0.29	no	0.93	no	down	2.8E-14	yes
ENSRNOT00000010118 : Akap9 : F1LPB4	2.74	yes	up	2.9E-04	yes	chr4	intron	internal intron	NA	1.01	no	up	0.74	no	1.05	no	up	2.3E-05	yes
MSTRG.5280.2 : Taf6 : Q498R0	0.74	yes	down	2.2E-06	yes	chr12	intron	internal intron	NA	0.75	no	down	0.09	no	0.91	no	down	1.8E-03	yes
MSTRG.18264.1 : Notch3 : F1LQX7	0.61	yes	down	2.1E-04	yes	chr7	exon	internal exon	NA	1.57	no	up	0.11	no	0.96	no	down	9.5E-04	yes
ENSRNOT00000091230 : Exoc1 : A0A0G2K2V5	inf	yes	up	6.1E-05	yes	chr14	intron	first intron	NA	0.83	no	down	0.23	no	0.90	no	down	2.2E-04	yes
MSTRG.21176.7 : Inpp5d : F1M981	0.08	yes	down	9.9E-05	yes	chr9	intron	internal intron	NA	0.81	no	down	0.33	no	1.08	no	up	3.1E-05	yes
MSTRG.16309.1 : Zmpste24 : D4A5K6	2.42	yes	up	2.9E-04	yes	chr5	promoter	Proximal	LCP	0.99	no	down	0.96	no	0.98	no	down	1.5E-05	yes
MSTRG.3024.1 : Sgcd : F1LYS7	0.12	yes	down	5.4E-11	yes	chr10	intron	internal intron	NA	1.06	no	up	0.58	no	0.81	yes	down	2.9E-17	yes
ENSRNOT00000021648 : Cirbp : P60825	0.73	yes	down	1.3E-04	yes	chr7	exon	first exon	NA	0.77	no	down	0.65	no	0.85	no	down	3.4E-16	yes
MSTRG.479.2 : Epn1 : O88339	2.09	yes	up	1.9E-04	yes	chr1	intron	internal intron	NA	0.93	no	down	0.58	no	0.99	no	down	3.7E-01	no
MSTRG.17567.1 : Elmsan1 : D4ACA6	0.27	yes	down	2.9E-04	yes	chr6	exon	last exon	NA	0.50	yes	down	0.41	no	0.96	no	down	1.9E-01	no
MSTRG.659.5 : Cic : D4A853	4.32	yes	up	3.8E-07	yes	chr1	exon	internal exon	NA	1.18	no	up	0.74	no	1.06	no	up	4.3E-02	yes
MSTRG.22038.1 : Fmr1 : Q80WE1	inf	yes	up	3.0E-04	yes	chrX	intron	internal intron	NA	0.84	no	down	0.29	no	1.09	no	up	7.3E-06	yes
MSTRG.18368.2 : Nt5dc3 : D3ZAI6	1.55	yes	up	4.2E-07	yes	chr7	intron	internal intron	NA	2.53	yes	up	0.09	no	1.05	no	up	2.5E-05	yes
MSTRG.15110.4 : Wnk1 : Q9JIH7	18.77	yes	up	1.6E-38	yes	chr4	exon	internal exon	NA	0.82	no	down	0.51	no	1.04	no	up	1.7E-03	yes
MSTRG.7226.1 : Sec24c : A0A0G2JZF0	3.50	yes	up	1.3E-09	yes	chr15	intron	internal intron	NA	0.61	no	down	0.49	no	1.01	no	up	2.6E-01	no
MSTRG.19194.8 : Hdac7 : A0A0G2K6B1	1.57	yes	up	6.8E-05	yes	chr7	intron	internal intron	NA	0.88	no	down	0.48	no	0.99	no	down	6.7E-01	no
MSTRG.4991.4 : Ppm1f : Q9WVR7	1.80	yes	up	3.8E-11	yes	chr11	intron	internal intron	NA	1.89	no	up	0.48	no	0.91	no	down	2.5E-06	yes
ENSRNOT00000071215 : Gnas : P63095	0.77	yes	down	3.7E-04	yes	chr3	promoter	Distal	HCP	0.96	no	down	0.53	no	0.90	no	down	5.6E-15	yes
MSTRG.13265.1 : Tcp11l1 : F1M9Y7	1.13	yes	up	1.2E-06	yes	chr3	intron	last intron	NA	1.57	no	up	0.04	yes	0.99	no	down	8.5E-01	no
ENSRNOT00000000904 : Chmp2b : F1M8B7	5.91	yes	up	3.7E-05	yes	chr11	intron	internal intron	NA	0.93	no	down	0.91	no	1.20	no	up	2.6E-07	yes
MSTRG.14053.1 : Dido1 : D3ZWL9	2.83	yes	up	7.0E-07	yes	chr3	intron	internal intron	NA	1.20	no	up	0.60	no	1.01	no	up	2.0E-01	no
MSTRG.16606.6 : Alpl : P08289	0.52	yes	down	2.4E-04	yes	chr5	promoter	Intermediate	LCP	0.29	yes	down	0.00	yes	1.00	no	down	6.2E-01	no
MSTRG.21649.2 : Apoo : M0R7G4	22.85	yes	up	2.9E-05	yes	chrX	intron	first intron	NA	1.19	no	up	0.80	no	0.95	no	down	2.3E-02	yes
ENSRNOT00000048980 : Usp9x : D3ZC84	21.85	yes	up	4.1E-05	yes	chrX	exon	last exon	NA	1.00	no	down	0.77	no	1.00	no	up	7.5E-01	no
MSTRG.14960.8 : Eogt : Q5NDL0	6.71	yes	up	1.7E-04	yes	chr4	intron	internal intron	NA	0.85	no	down	0.65	no	1.10	no	up	2.0E-05	yes
MSTRG.4725.17 : Kalrn : P97924	1.54	yes	up	9.1E-07	yes	chr11	exon	last exon	NA	1.21	no	up	0.35	no	1.04	no	up	1.6E-04	yes
ENSRNOT00000090038 : Tnpo3 : D4AAM0	6.44	yes	up	4.2E-12	yes	chr4	promoter	Distal	LCP	0.97	no	down	0.73	no	1.04	no	up	4.3E-04	yes
ENSRNOT00000050191 : Lrrfip1 : Q66HF9	6.49	yes	up	4.9E-10	yes	chr9	exon	last exon	NA	1.04	no	up	0.82	no	1.15	no	up	2.1E-11	yes
MSTRG.21023.4 : Fn1 : F1LST1	0.11	yes	down	7.7E-11	yes	chr9	intron	internal intron	NA	1.68	no	up	0.61	no	1.27	yes	up	4.1E-20	yes
ENSRNOT00000002428 : Osbpl11 : B5DF74	0.37	yes	down	2.7E-06	yes	chr11	exon	first exon	NA	1.03	no	up	0.89	no	1.07	no	up	2.3E-05	yes
ENSRNOT00000091470 : Picalm : A0A1B0GWW9	8.74	yes	up	2.1E-13	yes	chr1	intron	internal intron	NA	1.31	no	up	0.64	no	1.04	no	up	1.0E-01	no
MSTRG.17313.1 : Snx6 : B5DEY8	21.45	yes	up	3.0E-04	yes	chr6	exon	internal exon	NA	0.79	no	down	0.31	no	0.97	no	down	1.8E-09	yes
ENSRNOT00000010385 : Golga5 : G3V6Z7	4.67	yes	up	4.2E-17	yes	chr6	intron	last intron	NA	1.94	no	up	0.07	no	1.07	no	up	2.3E-12	yes
ENSRNOT00000019047 : Hint3 : A0A0G2K7W8	0.41	yes	down	1.7E-04	yes	chr1	intron	internal intron	NA	1.02	no	up	0.96	no	1.02	no	up	2.1E-01	no
ENSRNOT00000015210 : Rftn1 : G3V7I2	3.70	yes	up	2.3E-04	yes	chr9	intron	internal intron	NA	1.55	no	up	0.31	no	1.12	no	up	1.5E-09	yes
ENSRNOT00000005260 : Commd10 : Q68FS9	0.39	yes	down	4.0E-04	yes	chr18	intron	internal intron	NA	1.16	no	up	0.65	no	1.00	no	up	7.8E-01	no
MSTRG.4727.1 : Kalrn : P97924	1.54	yes	up	9.1E-07	yes	chr11	exon	last exon	NA	0.99	no	down	0.97	no	1.04	no	up	1.6E-04	yes

ENSRNOT00000092524 : Washc2c : F1LPG9	0.05	yes	down	2.7E-19	yes	chr4	exon	internal exon	NA	1.11	no	up	0.32	no	1.05	no	up	2.1E-05	yes
MSTRG.21085.2 : Chpf : Q5XIQ8	1.40	yes	up	4.1E-04	yes	chr9	intron	internal intron	NA	2.05	yes	up	0.06	no	1.00	no	down	8.4E-01	no
ENSRNOT00000088288 : Sptbn2 : F1MA36	0.33	yes	down	1.2E-08	yes	chr1	intron	internal intron	NA	0.95	no	down	0.22	no	0.96	no	down	3.4E-01	no
MSTRG.19538.3 : Nfrkb : D4A421	0.58	yes	down	1.3E-05	yes	chr8	intron	internal intron	NA	1.12	no	up	0.52	no	0.89	no	down	2.5E-03	yes
ENSRNOT00000009104 : Gcfc2 : D4A3Z4	0.38	yes	down	6.2E-05	yes	chr4	intron	first intron	NA	0.89	no	down	0.47	no	0.96	no	down	1.9E-01	no
MSTRG.2010.6 : Ppp6r3 : D3ZBT9	3.21	yes	up	8.1E-07	yes	chr1	intron	internal intron	NA	0.74	no	down	0.07	no	0.98	no	down	7.0E-03	yes
ENSRNOT00000093260 : Mycbp2 : D4A2D3	0.31	yes	down	9.2E-06	yes	chr15	intron	internal intron	NA	0.97	no	down	0.34	no	1.00	no	up	6.8E-01	no
MSTRG.8233.2 : Fat1 : G3V9W9	0.12	yes	down	1.5E-10	yes	chr16	exon	last exon	NA	0.48	yes	down	0.51	no	1.12	no	up	2.9E-12	yes
MSTRG.11265.33 : Ubap2l : E9PTR4	6.62	yes	up	4.0E-04	yes	chr2	intron	internal intron	NA	0.97	no	down	0.93	no	1.13	no	up	3.8E-12	yes
ENSRNOT00000023648 : Ppme1 : Q4FZT2	16.52	yes	up	4.2E-04	yes	chr1	intron	internal intron	NA	0.74	no	down	0.12	no	1.02	no	up	3.9E-05	yes
MSTRG.16606.1 : Alpl : P08289	0.52	yes	down	2.4E-04	yes	chr5	promoter	Intermediate	LCP	1.22	no	up	0.71	no	1.00	no	down	6.2E-01	no
MSTRG.21083.3 : Speg : Q63638	0.17	yes	down	2.6E-06	yes	chr9	intron	internal intron	NA	2.02	yes	up	0.34	no	0.88	no	down	2.3E-07	yes
ENSRNOT00000064495 : C1qtnf7 : B2RYB7	3.60	yes	up	9.8E-06	yes	chr14	exon	last exon	NA	0.60	no	down	0.13	no	0.87	no	down	2.3E-09	yes
MSTRG.20879.4 : Stat1 : F1M9D6	0.50	yes	down	1.5E-10	yes	chr9	intron	first intron	NA	1.22	no	up	0.58	no	1.10	no	up	1.4E-13	yes
MSTRG.20879.2 : Stat1 : F1M9D6	0.50	yes	down	1.5E-10	yes	chr9	intron	first intron	NA	2.03	yes	up	0.12	no	1.10	no	up	1.4E-13	yes
MSTRG.14960.4 : Eogt : Q5NDL0	6.71	yes	up	1.7E-04	yes	chr4	intron	internal intron	NA	0.77	no	down	0.71	no	1.10	no	up	2.0E-05	yes
MSTRG.15855.2 : RGD1306148 : F1M446	0.22	yes	down	4.0E-05	yes	chr5	intron	internal intron	NA	0.48	yes	down	0.45	no	1.01	no	up	7.4E-02	no
MSTRG.11888.7 : Ppp1r18 : A0A0G2K8B3	0.32	yes	down	2.0E-04	yes	chr20	promoter	Distal	LCP	1.26	no	up	0.64	no	1.10	no	up	1.1E-05	yes
ENSRNOT00000009173 : Arid1a : D4A3E3	0.22	yes	down	1.1E-04	yes	chr5	intron	first intron	NA	1.20	no	up	0.74	no	1.00	no	down	2.1E-01	no
MSTRG.1731.1 : Tbc1d10b : D3ZSY8	1.59	yes	up	2.7E-04	yes	chr1	intron	internal intron	NA	0.93	no	down	0.89	no	0.99	no	down	3.1E-01	no
MSTRG.6812.1 : Pcdh7 : Q68HB8	0	yes	down	4.2E-04	yes	chr14	exon	last exon	NA	0.84	no	down	0.65	no	0.94	no	down	1.0E-07	yes
ENSRNOT00000091702 : Lima1 : F1LR10	0.35	yes	down	4.4E-04	yes	chr7	intron	internal intron	NA	0.32	yes	down	0.09	no	1.16	no	up	2.2E-10	yes
MSTRG.21205.1 : Ube2f : Q5U203	7.18	yes	up	7.2E-05	yes	chr9	intron	internal intron	NA	1.07	no	up	0.87	no	1.01	no	up	5.6E-01	no
ENSRNOT00000076699 : Yipf6 : A0A096MJG6	inf	yes	up	3.8E-06	yes	chrX	intron	first intron	NA	1.00	no	down	0.73	no	1.03	no	up	1.9E-01	no
MSTRG.3511.5 : Cpd : A0A0G2JY30	0.15	yes	down	4.3E-04	yes	chr10	intron	internal intron	NA	0.73	no	down	0.51	no	1.13	no	up	1.1E-08	yes
ENSRNOT00000011401 : Tex10 : D4A401	5.97	yes	up	4.0E-04	yes	chr5	intron	internal intron	NA	0.84	no	down	0.45	no	0.97	no	down	1.1E-01	no
MSTRG.3521.1 : Inpp5k : A0A0G2K2Z2	8.85	yes	up	4.8E-08	yes	chr10	promoter	Distal	ICP	0.93	no	down	0.26	no	0.94	no	down	3.3E-03	yes
ENSRNOT00000068334 : Dcun1d1 : D3ZRV0	1.60	yes	up	3.7E-04	yes	chr2	exon	internal exon	NA	0.99	no	down	0.98	no	0.97	no	down	2.6E-02	yes
MSTRG.15938.3 : Tnc : A0A0G2K1L0	0	yes	down	7.1E-07	yes	chr5	intron	internal intron	NA	1.23	no	up	0.46	no	1.49	yes	up	1.5E-12	yes
MSTRG.18268.5 : Akap8 : Q63014	0.08	yes	down	2.7E-04	yes	chr7	intron	internal intron	NA	0.98	no	down	0.86	no	0.99	no	down	2.8E-01	no
ENSRNOT00000003748 : Serpinc1 : Q5M7T5	11.92	yes	up	5.6E-26	yes	chr13	intron	first intron	NA	0.97	no	down	0.21	no	0.89	no	down	1.2E-15	yes
MSTRG.8665.1 : Kif13a : D3ZM20	0	yes	down	7.8E-05	yes	chr17	intron	internal intron	NA	0.77	no	down	0.81	no	1.00	no	up	9.5E-01	no
MSTRG.123.7 : Epb41l2 : D3ZM69	0.25	yes	down	1.1E-12	yes	chr1	intron	internal intron	NA	0.84	no	down	0.55	no	0.91	no	down	8.9E-11	yes
MSTRG.21926.4 : Pls3 : F1LPK7	0	yes	down	1.5E-04	yes	chrX	intron	internal intron	NA	1.96	no	up	0.07	no	1.11	no	up	9.8E-14	yes
MSTRG.7830.5 : Dock9 : F1LSM8	0.10	yes	down	2.7E-05	yes	chr15	intron	internal intron	NA	1.01	no	up	0.97	no	0.97	no	down	2.3E-04	yes
ENSRNOT00000012415 : Prkar2b : P12369	0.61	yes	down	1.6E-04	yes	chr6	intron	internal intron	NA	0.73	no	down	0.51	no	0.71	yes	down	2.8E-15	yes
MSTRG.17232.1 : Lamb1 : D3ZQN7	3.86	yes	up	8.9E-05	yes	chr6	intron	internal intron	NA	1.22	no	up	0.71	no	1.31	yes	up	5.4E-17	yes
MSTRG.14118.3 : Kmt2c : A0A0G2K426	0.22	yes	down	1.1E-11	yes	chr4	promoter	Distal	LCP	0.73	no	down	0.27	no	0.69	yes	down	1.4E-11	yes
MSTRG.12153.1 : Trappc10 : F1MAQ4	0.63	yes	down	1.1E-04	yes	chr20	exon	last exon	NA	0.54	no	down	0.40	no	0.99	no	down	5.9E-01	no
MSTRG.5942.4 : Ppp1r12b : D3ZIC4	2.69	yes	up	3.9E-04	yes	chr13	intron	internal intron	NA	0.91	no	down	0.29	no	0.95	no	down	7.9E-06	yes
MSTRG.5509.4 : Pitpnm2 : A0A0G2JW50	2.70	yes	up	3.9E-04	yes	chr12	promoter	Distal	LCP	0.46	yes	down	0.02	yes	0.89	no	down	1.9E-08	yes
MSTRG.19956.5 : Myo1e : A0A0G2K9E8	3.15	yes	up	9.4E-05	yes	chr8	intron	first intron	NA	0.37	yes	down	0.20	no	0.76	yes	down	2.6E-20	yes
MSTRG.6685.1 : Exoc1 : A0A0G2K2V5	inf	yes	up	6.1E-05	yes	chr14	intron	first intron	NA	1.28	no	up	0.02	yes	0.90	no	down	2.2E-04	yes
ENSRNOT00000023117 : Hydin : D3ZBX6	3.95	yes	up	2.0E-07	yes	chr19	intron	internal intron	NA	0.54	no	down	0.24	no	0.80	yes	down	2.3E-10	yes
ENSRNOT00000091117 : Pi4ka : A0A140TAJ5	7.84	yes	up	1.4E-07	yes	chr11	intron	internal intron	NA	3.61	yes	up	0.15	no	0.97	no	down	1.2E-03	yes
MSTRG.22041.3 : Dkc1 : P40615	0.28	yes	down	2.4E-05	yes	chrX	intron	internal intron	NA	0.65	no	down	0.45	no	0.99	no	down	3.6E-01	no
ENSRNOT00000086335 : Ss18 : Q5XI66	6.27	yes	up	1.6E-10	yes	chr18	exon	internal exon	NA	1.13	no	up	0.03	yes	1.04	no	up	6.9E-02	no
MSTRG.4508.8 : Dyrk1a : Q63470	0.26	yes	down	2.2E-05	yes	chr11	intron	internal intron	NA	0.54	no	down	0.45	no	1.02	no	up	1.6E-01	no
MSTRG.2622.1 : Nt5c2 : D3ZMY7	17.59	yes	up	5.0E-22	yes	chr1	exon	last exon	NA	1.01	no	up	0.93	no	0.92	no	down	2.5E-12	yes
MSTRG.21083.5 : Speg : Q63638	0.17	yes	down	2.6E-06	yes	chr9	intron	internal intron	NA	1.89	no	up	0.18	no	0.88	no	down	2.3E-07	yes
MSTRG.8278.23 : Rbpms : F2Z3S5	0.02	yes	down	2.6E-05	yes	chr16	promoter	Proximal	ICP	1.05	no	up	0.71	no	1.06	no	up	4.9E-05	yes
MSTRG.2881.2 : Pkd1 : Q9ERV0	inf	yes	up	6.6E-05	yes	chr10	intron	internal intron	NA	1.27	no	up	0.60	no	1.19	no	up	9.8E-07	yes
ENSRNOT00000089068 : Ppp1r12b : D3ZIC4	2.69	yes	up	3.9E-04	yes	chr13	intron	internal intron	NA	0.82	no	down	0.21	no	0.95	no	down	7.9E-06	yes
MSTRG.19963.1 : Sltm : A0A0G2K904	0.34	yes	down	6.4E-06	yes	chr8	intron	internal intron	NA	1.06	no	up	0.89	no	0.94	no	down	2.8E-02	yes
MSTRG.19819.4 : Neo1 : P97603	0	yes	down	3.5E-04	yes	chr8	promoter	Intermediate	LCP	1.06	no	up	0.78	no	0.98	no	down	1.8E-02	yes
ENSRNOT00000059565 : Ankrd33b : A0A0G2JZI6	0	yes	down	3.3E-04	yes	chr2	exon	last exon	NA	1.13	no	up	0.39	no	1.18	no	up	2.0E-04	yes
ENSRNOT00000047805 : Asph : A0A096MKE0	2.56	yes	up	9.8E-06	yes	chr5	intron	internal intron	NA	1.23	no	up	0.56	no	0.99	no	down	1.3E-01	no
ENSRNOT00000068682 : Ankrd17 : D4A0B4	3.39	yes	up	8.9E-05	yes	chr14	intron	internal intron	NA	0.97	no	down	0.39	no	1.11	no	up	1.6E-06	yes
ENSRNOT00000050792 : Pan2 : R9PXX6	2.14	yes	up	4.2E-04	yes	chr7	exon	internal exon	NA	1.16	no	up	0.03	yes	0.84	no	down	1.2E-03	yes
MSTRG.14776.1 : Gcfc2 : D4A3Z4	0.38	yes	down	6.2E-05	yes	chr4	intron	first intron	NA	1.03	no	up	0.82	no	0.96	no	down	1.9E-01	no
MSTRG.20748.3 : Prim2 : Q4V8C0	4.01	yes	up	2.8E-05	yes	chr9	intron	internal intron	NA	1.00	no	down	0.99	no	1.03	no	up	2.8E-01	no
MSTRG.15318.2 : Ddx47 : G3V727	0.37	yes	down	4.0E-04	yes	chr4	intron	internal intron	NA	1.05	no	up	0.77	no	1.05	no	up	9.1E-04	yes
MSTRG.8713.2 : Sirt5 : Q68FX9	6.16	yes	up	1.6E-04	yes	chr17	intron	internal intron	NA	1.14	no	up	0.22	no	1.01	no	up	5.1E-01	no
MSTRG.549.4 : Zc3h4 : D3ZVW3	2.19	yes	up	2.6E-04	yes	chr1	intron	internal intron	NA	0.73	no	down	0.17	no	0.98	no	down	3.2E-01	no
ENSRNOT00000064323 : Ddx31 : B1H297	0.21	yes	down	8.0E-05	yes	chr3	intron	first intron	NA	1.25	no	up	0.13	no	0.95	no	down	4.7E-02	yes
MSTRG.19777.4 : Sin3a : A0A0G2K3H5	0.53	yes	down	4.1E-06	yes	chr8	intron	last intron	NA	0.39	yes	down	0.05	yes	0.96	no	down	6.2E-05	yes
ENSRNOT00000089584 : Nfatc4 : A0A0G2K0L1	0	yes	down	3.3E-04	yes	chr15	intron	internal intron	NA	1.11	no	up	0.67	no	1.13	no	up	1.3E-11	yes
ENSRNOT00000002727 : Atp6v1a : D4A133	0.36	yes	down	7.3E-06	yes	chr11	intron	internal intron	NA	1.18	no	up	0.46	no	1.08	no	up	1.1E-08	yes
MSTRG.10426.4 : Scamp1 : A0A0G2K1I6	11.34	yes	up	2.9E-04	yes	chr2	intron	first intron	NA	0.90	no	down	0.50	no	1.05	no	up	3.2E-06	yes
MSTRG.17811.4 : Mark3 : F1M836	0.30	yes	down	2.6E-04	yes	chr6	exon	last exon	NA	1.21	no	up	0.35	no	1.10	no	up	4.1E-07	yes
MSTRG.3783.2 : Acsf2 : Q499N5	0.31	yes	down	5.4E-07	yes	chr10	intron	internal intron	NA	0.80	no	down	0.10	no	0.87	no	down	2.4E-13	yes
MSTRG.540.1 : Slc8a2 : A0A0G2JZK7	5.43	yes	up	2.7E-05	yes	chr1	exon	last exon	NA	1.03	no	up	0.80	no	0.78	yes	down	9.3E-16	yes
ENSRNOT00000078073 : Gls : P13264	0.05	yes	down	1.5E-13	yes	chr9	intron	first intron	NA	0.34	yes	down	0.01	yes	1.08	no	up	4.1E-02	yes
ENSRNOT00000023567 : Tpm3 : Q63610	0.37	yes	down	8.0E-05	yes	chr2	intron	internal intron	NA	1.07	no	up	0.64	no	1.11	no	up	3.2E-12	yes
MSTRG.16452.1 : Tinagl1 : Q4V8N0	0.10	yes	down	7.3E-25	yes	chr5	promoter	Distal	LCP	0.81	no	down	0.70	no	0.89	no	down	4.1E-08	yes
ENSRNOT00000028098 : Rnls : Q5U2W9	0.31	yes	down	2.4E-05	yes	chr1	promoter	Distal	LCP	0.93	no	down	0.64	no	1.02	no	up	3.0E-01	no
MSTRG.4335.1 : Pcyt2 : O88637	4.82	yes	up	3.0E-05	yes	chr10	intron	last intron	NA	1.77	no	up	0.06	no	0.94	no	down	1.8E-03	yes
MSTRG.19538.9 : Nfrkb : D4A421	0.58	yes	down	1.3E-05	yes	chr8	intron	internal intron	NA	0.76	no	down	0.50	no	0.89	no	down	2.5E-03	yes
MSTRG.20764.6 : Uggt1 : Q9JLA3	0.22	yes	down	3.9E-04	yes	chr9	intron	internal intron	NA	1.03	no	up	0.49	no	1.11	no	up	9.8E-15	yes
MSTRG.19472.8 : Dnm2 : A0A0A0MY48	0.11	yes	down	8.9E-11	yes	chr8	intron	first intron	NA	1.10	no	up	0.23	no	1.05	no	up	2.0E-08	yes
MSTRG.19407.2 : Med17 : D4ABU0	3.31	yes	up	2.5E-04	yes	chr8	intron	last intron	NA	0.64	no	down	0.50	no	0.89	no	down	7.9E-05	yes
MSTRG.21122.7 : Agfg1 : F1M9N7	3.59	yes	up	1.8E-08	yes	chr9	exon	internal exon	NA	1.13	no	up	0.61	no	1.16	no	up	6.0E-06	yes
ENSRNOT00000064257 : Bmi1 : B4F7B6	2.46	yes	up	2.3E-04	yes	chr17	exon	last exon	NA	0.36	yes	down	0.46	no	0.98	no	down	3.6E-01	no
MSTRG.21604.1 : Tbl1x : B2RZA6	0	yes	down	6.0E-05	yes	chrX	intron	internal intron	NA	1.92	no	up	0.06	no	1.12	no	up	7.8E-09	yes
ENSRNOT00000052131 : Fam98a : Q5FWT1	0.57	yes	down	6.5E-05	yes	chr6	exon	last exon	NA	0.98	no	down	0.97	no	0.98	no	down	9.6E-02	no
ENSRNOT00000013621 : Wnk1 : Q9JIH7	18.77	yes	up	1.6E-38	yes	chr4	exon	internal exon	NA	1.00	no	down	0.48	no	1.04	no	up	1.7E-03	yes
MSTRG.18268.13 : Akap8 : Q63014	0.08	yes	down	2.7E-04	yes	chr7	intron	internal intron	NA	1.05	no	up	0.58	no	0.99	no	down	2.8E-01	no
MSTRG.16609.7 : Ece1 : Q6IN10	0.58	yes	down	9.3E-05	yes	chr5	intron	internal intron	NA	0.55	no	down	0.16	no	1.14	no	up	8.3E-15	yes
MSTRG.6127.2 : Fmo2 : G3V6F6	2.66	yes	up	2.1E-11	yes	chr13	intron	internal intron	NA	0.49	yes	down	0.10	no	1.21	yes	up	2.2E-15	yes
MSTRG.14075.8 : Dnajc5 : A0A0G2JX56	0.30	yes	down	1.1E-06	yes	chr3	promoter	Intermediate	HCP	0.99	no	down	0.48	no	0.89	no	down	4.0E-08	yes
MSTRG.4304.2 : Cep131 : D4AEL8	3.69	yes	up	1.2E-04	yes	chr10	intron	last intron	NA	0.98	no	down	0.93	no	0.92	no	down	1.5E-02	yes
ENSRNOT00000092956 : Gtf2i : Q5U2Y1	5.31	yes	up	4.9E-06	yes	chr12	promoter	Distal	LCP	1.00	no	down	0.95	no	0.95	no	down	1.5E-07	yes
MSTRG.1326.1 : Picalm : Q66SY1	8.74	yes	up	2.1E-13	yes	chr1	intron	internal intron	NA	3.98	yes	up	0.01	yes	1.13	no	up	1.1E-06	yes
MSTRG.2579.1 : Sfxn3 : Q9JHY2	0.39	yes	down	7.8E-05	yes	chr1	intron	internal intron	NA	2.21	yes	up	0.05	yes	0.98	no	down	7.5E-03	yes
MSTRG.9433.4 : Rbm27 : F1M1R4	0.45	yes	down	2.5E-04	yes	chr18	intron	internal intron	NA	0.65	no	down	0.20	no	1.01	no	up	5.2E-01	no
ENSRNOT00000086272 : Ndufv3 : Q6PCU8	0	yes	down	1.9E-04	yes	chr20	intron	first intron	NA	0.74	no	down	0.22	no	0.93	no	down	3.2E-03	yes
MSTRG.16636.1 : Ubr4 : A0A0G2JU89	2.84	yes	up	3.7E-05	yes	chr5	intron	internal intron	NA	0.69	no	down	0.17	no	1.02	no	up	4.6E-05	yes
MSTRG.20094.1 : Plscr1 : A0A0G2K7Q1	0.33	yes	down	1.3E-05	yes	chr8	promoter	Distal	LCP	0.91	no	down	0.62	no	1.08	no	up	5.2E-06	yes
ENSRNOT00000020669 : Col4a3 : Q63122	0.33	yes	down	1.8E-08	yes	chr9	intron	internal intron	NA	0.75	no	down	0.23	no	0.93	no	down	2.1E-06	yes
MSTRG.19764.3 : Pstpip1 : B0BNK4	0.47	yes	down	3.6E-05	yes	chr8	intron	internal intron	NA	0.80	no	down	0.20	no	1.20	yes	up	1.1E-06	yes

MSTRG.18072.3 : Dgka : P51556	0.72	yes	down	1.5E-04	yes	chr7	exon	internal exon	NA	1.64	no	up	0.22	no	0.95	no	down	8.8E-04	yes
MSTRG.3949.3 : Dhx58 : D3ZD46	5.00	yes	up	2.1E-04	yes	chr10	exon	internal exon	NA	0.76	no	down	0.01	yes	1.22	yes	up	6.3E-08	yes
MSTRG.19188.2 : Rpap3 : Q68FQ7	0.29	yes	down	4.9E-05	yes	chr7	intron	internal intron	NA	0.99	no	down	0.95	no	1.04	no	up	2.9E-04	yes
ENSRNOT00000090288 : Tpm1 : A0A0G2JSQ4	9.41	yes	up	5.1E-14	yes	chr8	intron	internal intron	NA	0.97	no	down	0.82	no	0.81	yes	down	2.5E-10	yes
MSTRG.16317.4 : Cap1 : Q08163	0.21	yes	down	1.6E-07	yes	chr5	intron	internal intron	NA	2.23	yes	up	0.00	yes	0.94	no	down	1.0E-08	yes
MSTRG.8094.2 : Atp13a1 : B5DEX7	3.83	yes	up	1.1E-13	yes	chr16	intron	internal intron	NA	2.38	yes	up	0.10	no	1.09	no	up	4.2E-12	yes
ENSRNOT00000066109 : Aldh3a2 : G3V9W6	0.68	yes	down	5.9E-04	yes	chr10	intron	internal intron	NA	1.02	no	up	0.95	no	0.89	no	down	4.4E-14	yes
MSTRG.16032.1 : Tek : D3ZCD0	inf	yes	up	4.6E-07	yes	chr5	intron	internal intron	NA	0.80	no	down	0.10	no	0.98	no	down	7.9E-02	no
MSTRG.16327.12 : Macf1 : A0A0G2K9T4	0.36	yes	down	1.3E-05	yes	chr5	exon	last exon	NA	1.05	no	up	0.91	no	0.97	no	down	9.0E-11	yes
ENSRNOT00000003901 : Ddr2 : B1WC09	0.49	yes	down	3.3E-05	yes	chr13	exon	internal exon	NA	1.18	no	up	0.35	no	0.95	no	down	3.2E-01	no
MSTRG.20044.1 : Phip : F1M3B3	4.05	yes	up	4.4E-04	yes	chr8	intron	internal intron	NA	1.14	no	up	0.90	no	0.95	no	down	6.3E-02	no
MSTRG.14384.1 : Atp6v1f : P50408	3.83	yes	up	8.5E-07	yes	chr4	exon	last exon	NA	0.87	no	down	0.85	no	1.10	no	up	1.4E-09	yes
ENSRNOT00000006053 : Sec14l4 : A0A0G2JWI8	0.38	yes	down	1.3E-04	yes	chr14	intron	internal intron	NA	1.29	no	up	0.31	no	0.91	no	down	1.1E-08	yes
MSTRG.10291.1 : Egln1 : A0A140TAD9	0.32	yes	down	1.1E-04	yes	chr19	exon	last exon	NA	0.91	no	down	0.81	no	1.28	yes	up	2.1E-09	yes
MSTRG.20154.1 : Armc8 : B4F7A2	0.25	yes	down	7.8E-05	yes	chr8	exon	first exon	NA	1.04	no	up	0.91	no	0.97	no	down	2.3E-03	yes
ENSRNOT00000066137 : Mxra7 : F1M1U0	inf	yes	up	4.6E-04	yes	chr10	intron	internal intron	NA	1.15	no	up	0.31	no	1.16	no	up	7.7E-10	yes
ENSRNOT00000015365 : Filip1 : F1LM79	4.15	yes	up	2.1E-10	yes	chr8	exon	first exon	NA	0.61	no	down	0.06	no	1.01	no	up	3.0E-01	no
ENSRNOT00000009496 : Neo1 : P97603	0	yes	down	3.5E-04	yes	chr8	promoter	Intermediate	LCP	1.16	no	up	0.48	no	0.98	no	down	1.8E-02	yes
MSTRG.15720.3 : Pigo : D3ZTP8	0.61	yes	down	2.7E-05	yes	chr5	exon	internal exon	NA	1.00	no	down	0.45	no	0.80	yes	down	9.0E-03	yes
MSTRG.3981.2 : Becn1 : Q91XJ1	0.15	yes	down	1.2E-04	yes	chr10	exon	first exon	NA	1.25	no	up	0.57	no	0.97	no	down	9.2E-02	no
MSTRG.1783.9 : Tgfb1i1 : Q99PD6	3.08	yes	up	7.2E-05	yes	chr1	intron	internal intron	NA	0.88	no	down	0.15	no	1.06	no	up	1.6E-04	yes
MSTRG.16048.2 : Fggy : Q5FVC3	18.59	yes	up	3.3E-04	yes	chr5	intron	internal intron	NA	1.74	no	up	0.17	no	0.84	no	down	2.3E-07	yes
MSTRG.8345.4 : Fgfr1 : F1LM54	0.22	yes	down	3.0E-05	yes	chr16	exon	last exon	NA	0.77	no	down	0.58	no	1.05	no	up	1.3E-02	yes
MSTRG.6158.4 : Dpt : B2RZ77	6.14	yes	up	2.8E-04	yes	chr13	exon	last exon	NA	0.22	yes	down	0.45	no	0.85	no	down	3.5E-10	yes
MSTRG.3468.3 : Pafah1b1 : P63004	0.19	yes	down	5.9E-04	yes	chr10	intron	internal intron	NA	0.77	no	down	0.52	no	0.96	no	down	1.4E-04	yes
ENSRNOT00000073948 : Ltn1 : F1M9Q3	0.38	yes	down	4.2E-04	yes	chr11	intron	internal intron	NA	0.88	no	down	0.78	no	1.08	no	up	1.4E-11	yes
MSTRG.15557.2 : Ints8 : A0A0G2K0V1	0.11	yes	down	2.1E-05	yes	chr5	intron	last intron	NA	1.03	no	up	0.50	no	1.07	no	up	1.6E-05	yes
ENSRNOT00000084725 : Exosc7 : Q5EB65	0.07	yes	down	4.3E-04	yes	chr8	intron	internal intron	NA	0.94	no	down	0.21	no	1.01	no	up	6.0E-01	no
ENSRNOT00000057950 : Ctnna3 : B2RYN9	0	yes	down	1.3E-06	yes	chr20	promoter	Distal	LCP	1.14	no	up	0.59	no	1.03	no	up	1.7E-01	no
MSTRG.18056.1 : Smarcc2 : D4A510	6.20	yes	up	2.1E-04	yes	chr7	intron	last intron	NA	1.01	no	up	0.99	no	0.96	no	down	6.4E-05	yes
ENSRNOT00000009028 : Sptb : Q6XDA0	0.47	yes	down	3.3E-04	yes	chr6	intron	internal intron	NA	0.97	no	down	0.16	no	0.94	no	down	2.4E-06	yes
MSTRG.18793.1 : Ndrg1 : Q6JE36	0.37	yes	down	7.6E-09	yes	chr7	intron	internal intron	NA	0.57	no	down	0.24	no	0.92	no	down	4.9E-08	yes
ENSRNOT00000009115 : Ubr5 : H9KVE3	4.26	yes	up	2.2E-04	yes	chr7	intron	first intron	NA	1.01	no	up	0.85	no	0.94	no	down	3.0E-02	yes
MSTRG.8717.1 : Mcur1 : D3ZEJ2	0.10	yes	down	2.2E-05	yes	chr17	promoter	Distal	LCP	0.57	no	down	0.56	no	0.92	no	down	1.0E-11	yes
MSTRG.8278.8 : Rbpms : F2Z3S5	0.02	yes	down	2.6E-05	yes	chr16	promoter	Proximal	ICP	0.96	no	down	0.85	no	1.06	no	up	4.9E-05	yes
ENSRNOT00000065335 : Trim16 : D3ZW47	5.28	yes	up	1.3E-04	yes	chr10	exon	last exon	NA	0.68	no	down	0.47	no	0.88	no	down	3.3E-06	yes
ENSRNOT00000082288 : Myo5a : A0A0G2K9S4	1.92	yes	up	1.3E-04	yes	chr8	exon	internal exon	NA	1.00	no	down	0.45	no	1.12	no	up	2.4E-10	yes
MSTRG.1683.3 : Il4r : Q63257	9.80	yes	up	2.9E-08	yes	chr1	intron	internal intron	NA	1.93	no	up	0.33	no	1.11	no	up	2.1E-05	yes
ENSRNOT00000027526 : Calhm2 : Q5RJQ8	0.13	yes	down	1.1E-04	yes	chr1	intron	first intron	NA	1.66	no	up	0.01	yes	1.02	no	up	9.8E-02	no
MSTRG.16748.4 : Tardbp : I6L9G6	2.86	yes	up	4.5E-10	yes	chr5	intron	internal intron	NA	0.65	no	down	0.01	yes	1.05	no	up	5.8E-11	yes
MSTRG.12896.3 : Rabgap1 : D3ZX42	3.82	yes	up	8.1E-06	yes	chr3	intron	internal intron	NA	0.91	no	down	0.40	no	1.01	no	up	1.9E-01	no
ENSRNOT00000087906 : Cast : F1LPH1	0.36	yes	down	1.6E-06	yes	chr2	intron	internal intron	NA	0.97	no	down	0.70	no	0.95	no	down	2.4E-10	yes
MSTRG.14603.2 : Tax1bp1 : Q66HA4	8.48	yes	up	1.1E-06	yes	chr4	exon	last exon	NA	0.93	no	down	0.92	no	1.04	no	up	2.0E-04	yes
MSTRG.18183.1 : Abhd17a : Q5XIJ5	3.11	yes	up	7.4E-06	yes	chr7	exon	internal exon	NA	1.13	no	up	0.30	no	0.84	no	down	6.6E-10	yes
ENSRNOT00000040356 : Gtf2i : Q5U2Y1	5.31	yes	up	4.9E-06	yes	chr12	promoter	Distal	LCP	0.90	no	down	0.76	no	0.95	no	down	1.5E-07	yes
ENSRNOT00000084471 : Lmo7 : A0A0G2K174	3.35	yes	up	1.2E-04	yes	chr15	intron	internal intron	NA	1.00	no	down	0.81	no	0.96	no	down	4.2E-06	yes
ENSRNOT00000081482 : Macf1 : A0A0G2K9T4	0.36	yes	down	1.3E-05	yes	chr5	exon	last exon	NA	0.97	no	down	0.52	no	0.97	no	down	9.0E-11	yes
MSTRG.3295.1 : Sco1 : B0BNM7	0.18	yes	down	3.1E-08	yes	chr10	promoter	Intermediate	ICP	0.80	no	down	0.51	no	1.00	no	up	8.2E-01	no
ENSRNOT00000026567 : Lsm4 : D4A2C6	0.48	yes	down	3.2E-04	yes	chr16	exon	last exon	NA	1.36	no	up	0.10	no	0.96	no	down	2.1E-01	no
ENSRNOT00000084420 : Asf1a : A0A0G2JTI3	0.41	yes	down	1.4E-04	yes	chr20	intron	last intron	NA	1.00	no	down	0.47	no	0.88	no	down	4.8E-08	yes
MSTRG.20561.3 : Rftn1 : G3V7I2	3.70	yes	up	2.3E-04	yes	chr9	intron	internal intron	NA	0.68	no	down	0.40	no	1.12	no	up	1.5E-09	yes
ENSRNOT00000024959 : Mitd1 : Q5I0J5	0.26	yes	down	9.9E-05	yes	chr9	promoter	Intermediate	LCP	1.24	no	up	0.19	no	1.14	no	up	9.0E-08	yes
MSTRG.9425.2 : Nr3c1 : P06536	0.27	yes	down	1.2E-04	yes	chr18	exon	first exon	NA	1.11	no	up	0.85	no	0.91	no	down	1.6E-10	yes
MSTRG.7830.4 : Dock9 : F1LSM8	0.10	yes	down	2.7E-05	yes	chr15	intron	internal intron	NA	0.57	no	down	0.31	no	0.97	no	down	2.3E-04	yes
ENSRNOT00000029255 : Vps8 : D4A5F7	7.39	yes	up	3.4E-05	yes	chr11	intron	internal intron	NA	0.65	no	down	0.38	no	0.95	no	down	3.2E-03	yes
MSTRG.17299.2 : Heatr5a : F1LSK5	0.05	yes	down	7.6E-05	yes	chr6	intron	internal intron	NA	1.45	no	up	0.10	no	0.98	no	down	2.4E-01	no
ENSRNOT00000010895 : Tmem43 : Q5XIP9	5.31	yes	up	2.6E-04	yes	chr4	exon	internal exon	NA	1.13	no	up	0.80	no	0.99	no	down	3.0E-01	no
MSTRG.8772.1 : Serpinb6b : Q68FX2	5.33	yes	up	5.8E-08	yes	chr17	promoter	Distal	LCP	1.30	no	up	0.27	no	0.95	no	down	6.3E-06	yes
ENSRNOT00000017832 : Cwc27 : A0A0G2JXR7	2.71	yes	up	6.8E-05	yes	chr2	intron	internal intron	NA	1.30	no	up	0.05	yes	1.19	no	up	1.4E-06	yes
ENSRNOT00000019601 : Anln : M0RDG0	0.78	yes	down	4.3E-04	yes	chr8	intron	internal intron	NA	1.15	no	up	0.10	no	0.97	no	down	9.1E-01	no
MSTRG.16636.9 : Ubr4 : A0A0G2JU89	2.84	yes	up	3.7E-05	yes	chr5	intron	internal intron	NA	0.68	no	down	0.63	no	1.02	no	up	4.6E-05	yes
MSTRG.11379.2 : Txnip : Q5M7W1	6.38	yes	up	4.0E-07	yes	chr2	exon	internal exon	NA	1.00	no	down	1.00	no	0.85	no	down	1.0E-06	yes
ENSRNOT00000001036 : RT1-S3 : Q9R0V1	0.16	yes	down	4.2E-07	yes	chr20	exon	last exon	NA	0.91	no	down	0.62	no	1.05	no	up	2.0E-01	no
MSTRG.12403.1 : Msl3l2 : Q6AYG1	0.29	yes	down	7.4E-06	yes	chr20	exon	first exon	NA	2.03	yes	up	0.09	no	0.89	no	down	2.3E-05	yes
MSTRG.9649.4 : Hdhd2 : Q6QI86	0.20	yes	down	5.1E-06	yes	chr18	intron	first intron	NA	0.71	no	down	0.51	no	1.04	no	up	3.3E-03	yes
MSTRG.4716.5 : Adcy5 : G3V9G1	0.16	yes	down	2.3E-10	yes	chr11	intron	internal intron	NA	0.94	no	down	0.52	no	1.00	no	down	7.6E-01	no
MSTRG.9752.5 : Coq9 : Q68FT1	0.32	yes	down	5.1E-11	yes	chr19	promoter	Distal	LCP	1.01	no	up	0.92	no	0.93	no	down	1.8E-05	yes
ENSRNOT00000083340 : Cdk5rap2 : F1M4B7	0	yes	down	1.4E-04	yes	chr5	intron	internal intron	NA	0.90	no	down	0.76	no	0.98	no	down	2.6E-01	no
ENSRNOT00000025415 : Picalm : Q66SY1	8.74	yes	up	2.1E-13	yes	chr1	intron	internal intron	NA	0.69	no	down	0.22	no	1.13	no	up	1.1E-06	yes
ENSRNOT00000082592 : Prom1 : Q9JI49	8.32	yes	up	2.4E-09	yes	chr14	intron	internal intron	NA	1.03	no	up	0.85	no	0.87	no	down	1.5E-05	yes
ENSRNOT00000066084 : Cox7a2 : P35171	0.27	yes	down	2.3E-04	yes	chr8	promoter	Proximal	LCP	0.63	no	down	0.20	no	1.01	no	up	4.7E-01	no
MSTRG.8278.2 : Rbpms : F2Z3S5	0.02	yes	down	2.6E-05	yes	chr16	promoter	Proximal	ICP	0.91	no	down	0.66	no	1.06	no	up	4.9E-05	yes
MSTRG.6590.1 : Scarb2 : P27615	6.44	yes	up	3.7E-05	yes	chr14	intron	internal intron	NA	0.69	no	down	0.08	no	0.99	no	down	3.4E-01	no
ENSRNOT00000077461 : Aldh3a2 : G3V9W6	0.68	yes	down	5.9E-04	yes	chr10	intron	internal intron	NA	0.98	no	down	0.84	no	0.89	no	down	4.4E-14	yes
MSTRG.18268.20 : Akap8 : Q63014	0.08	yes	down	2.7E-04	yes	chr7	intron	internal intron	NA	2.36	yes	up	0.26	no	0.99	no	down	2.8E-01	no
MSTRG.3249.4 : Specc1 : A0A0G2K5D7	5.53	yes	up	1.9E-10	yes	chr10	exon	last exon	NA	1.15	no	up	0.59	no	0.93	no	down	4.4E-09	yes
ENSRNOT00000084736 : ST7 : A0A0G2KBB5	4.88	yes	up	1.5E-16	yes	chr4	promoter	Distal	LCP	1.03	no	up	0.04	yes	1.14	no	up	1.4E-03	yes
MSTRG.14075.7 : Dnajc5 : A0A0G2JX56	0.30	yes	down	1.1E-06	yes	chr3	promoter	Intermediate	HCP	1.10	no	up	0.77	no	0.89	no	down	4.0E-08	yes
MSTRG.18475.2 : Pawr : G3V6S1	4.33	yes	up	2.3E-06	yes	chr7	intron	last intron	NA	2.85	yes	up	0.23	no	1.06	no	up	9.6E-07	yes
MSTRG.3849.2 : Pnpo : O88794	2.62	yes	up	1.2E-06	yes	chr10	intron	internal intron	NA	1.00	no	down	0.99	no	0.99	no	down	6.7E-01	no
ENSRNOT00000077809 : Ddx39b : Q63413	0.02	yes	down	4.7E-11	yes	chr20	exon	last exon	NA	0.79	no	down	0.74	no	1.01	no	up	2.0E-01	no
MSTRG.18880.1 : Plec : Q6S3A0	0.33	yes	down	4.2E-06	yes	chr7	exon	last exon	NA	0.61	no	down	0.10	no	1.11	no	up	6.0E-08	yes
MSTRG.4375.1 : Tbcd : F1M1D5	10.07	yes	up	4.7E-25	yes	chr10	intron	internal intron	NA	1.13	no	up	0.55	no	0.94	no	down	2.3E-10	yes
ENSRNOT00000004231 : Prom1 : Q9JI49	8.32	yes	up	2.4E-09	yes	chr14	intron	internal intron	NA	0.43	yes	down	0.29	no	0.87	no	down	1.5E-05	yes
ENSRNOT00000080049 : Ube3b : A0A0G2K0E7	0.36	yes	down	3.1E-06	yes	chr12	intron	internal intron	NA	1.13	no	up	0.21	no	1.15	no	up	1.8E-12	yes
MSTRG.6568.4 : Antxr2 : Q00IM8	1.10	no	up	9.8E-05	yes	chr14	intron	internal intron	NA	1.92	no	up	0.47	no	1.01	no	up	3.8E-01	no
ENSRNOT00000016340 : Kalrn : P97924	1.54	yes	up	9.1E-07	yes	chr11	exon	last exon	NA	1.98	no	up	0.26	no	1.04	no	up	1.6E-04	yes
MSTRG.8278.36 : Rbpms : F2Z3S5	0.02	yes	down	2.6E-05	yes	chr16	promoter	Proximal	ICP	1.09	no	up	0.69	no	1.06	no	up	4.9E-05	yes
MSTRG.5417.13 : Auts2 : F1M388	0.04	yes	down	4.8E-14	yes	chr12	intron	internal intron	NA	1.26	no	up	0.14	no	1.01	no	up	5.6E-01	no
MSTRG.15855.1 : RGD1306148 : F1M446	0.22	yes	down	4.0E-05	yes	chr5	intron	internal intron	NA	0.98	no	down	0.98	no	1.01	no	up	7.4E-02	no
ENSRNOT00000027789 : Nfatc4 : A0A0G2K0L1	0	yes	down	3.3E-04	yes	chr15	intron	internal intron	NA	1.10	no	up	0.50	no	1.13	no	up	1.3E-11	yes
MSTRG.11953.62 : RT1-CE7 : D3ZLE6	inf	yes	up	7.3E-07	yes	chr20	exon	internal exon	NA	1.80	no	up	0.04	yes	1.22	yes	up	7.3E-16	yes
MSTRG.16324.5 : Pabpc4 : G3V9N0	15.58	yes	up	1.8E-05	yes	chr5	intron	first intron	NA	1.09	no	up	0.75	no	1.10	no	up	6.8E-12	yes
MSTRG.12186.1 : S100b : P04631	0.27	yes	down	2.6E-04	yes	chr20	exon	last exon	NA	0.92	no	down	0.63	no	0.76	yes	down	2.0E-16	yes
MSTRG.18876.1 : Puf60 : A0A0H2UHZ6	0.25	yes	down	1.3E-05	yes	chr7	exon	internal exon	NA	1.12	no	up	0.59	no	1.00	no	down	7.4E-02	no
ENSRNOT00000044726 : Grb10 : D3ZEA3	0.35	yes	down	3.4E-07	yes	chr14	intron	internal intron	NA	1.00	no	down	0.95	no	1.09	no	up	4.5E-06	yes
MSTRG.4717.3 : Adcy5 : G3V9G1	0.16	yes	down	2.3E-10	yes	chr11	intron	internal intron	NA	1.24	no	up	0.48	no	1.00	no	down	7.6E-01	no
MSTRG.2596.3 : Gbf1 : A0A0G2K3N1	0.28	yes	down	1.1E-05	yes	chr1	intron	internal intron	NA	0.95	no	down	0.79	no	1.05	no	up	6.1E-10	yes
ENSRNOT00000016410 : Zmpste24 : D4A5K6	2.42	yes	up	2.9E-04	yes	chr5	promoter	Proximal	LCP	1.06	no	up	0.80	no	0.98	no	down	1.5E-05	yes
MSTRG.21168.2 : Gigyf2 : A0A096MKC0	0.59	yes	down	1.9E-05	yes	chr9	intron	internal intron	NA	0.86	no	down	0.28	no	1.01	no	up	7.0E-01	no

ENSRNOT00000022017 : Prrc1 : G3V834	0.26	yes	down	1.1E-04	yes	chr18	exon	first exon	NA	1.22	no	up	0.16	no	1.12	no	up	3.2E-14	yes
MSTRG.9268.3 : Lims2 : A0A0G2KAE1	0.67	yes	down	6.6E-13	yes	chr18	intron	internal intron	NA	1.05	no	up	0.91	no	0.82	yes	down	4.4E-10	yes
MSTRG.3280.1 : Arhgap44 : A0A0H2UHC0	7.94	yes	up	4.2E-19	yes	chr10	intron	internal intron	NA	1.84	no	up	0.17	no	1.04	no	up	6.6E-02	no
ENSRNOT00000080660 : Hdhd2 : Q6QI86	0.20	yes	down	5.1E-06	yes	chr18	intron	first intron	NA	1.09	no	up	0.76	no	1.04	no	up	3.3E-03	yes
ENSRNOT00000067448 : Insr : F1LPL6	0.36	yes	down	9.6E-05	yes	chr12	intron	internal intron	NA	1.05	no	up	0.09	no	1.09	no	up	1.4E-04	yes
MSTRG.22038.7 : Fmr1 : Q80WE1	inf	yes	up	3.0E-04	yes	chrX	intron	internal intron	NA	0.77	no	down	0.62	no	1.09	no	up	7.3E-06	yes
ENSRNOT00000021321 : Ube4a : F1M9N5	4.51	yes	up	1.3E-04	yes	chr8	intron	internal intron	NA	1.11	no	up	0.67	no	0.97	no	down	2.4E-03	yes
MSTRG.12896.1 : Rabgap1 : D3ZX42	3.82	yes	up	8.1E-06	yes	chr3	intron	internal intron	NA	1.01	no	up	0.98	no	1.01	no	up	1.9E-01	no
ENSRNOT00000006835 : Crip1 : P63255	8.66	yes	up	6.1E-05	yes	chr6	intron	first intron	NA	4.77	yes	up	0.33	no	1.20	yes	up	1.6E-08	yes
MSTRG.20878.3 : Gls : P13264	0.05	yes	down	1.5E-13	yes	chr9	intron	first intron	NA	1.28	no	up	0.55	no	1.08	no	up	4.1E-02	yes
ENSRNOT00000092795 : Clasp1 : F1LNR1	0.36	yes	down	6.7E-07	yes	chr13	intron	internal intron	NA	0.98	no	down	0.69	no	1.00	no	down	7.0E-01	no
ENSRNOT00000071280 : Prdm16 : M0RDL0	0.37	yes	down	2.7E-04	yes	chr5	exon	internal exon	NA	0.93	no	down	0.60	no	0.83	yes	down	1.1E-06	yes
ENSRNOT00000012247 : Jam3 : Q68FQ2	0.30	yes	down	4.0E-04	yes	chr8	exon	last exon	NA	1.11	no	up	0.25	no	0.97	no	down	8.5E-02	no
ENSRNOT00000066928 : Kcnma1 : A0A0G2K104	4.06	yes	up	2.6E-13	yes	chr15	intron	internal intron	NA	1.01	no	up	0.50	no	0.89	no	down	7.4E-06	yes
ENSRNOT00000065127 : Sgpl1 : Q8CHN6	0.20	yes	down	6.8E-07	yes	chr20	intron	internal intron	NA	1.23	no	up	0.07	no	1.12	no	up	5.6E-16	yes
ENSRNOT00000041891 : Apoe : P02650	0.23	yes	down	3.2E-04	yes	chr1	exon	last exon	NA	1.09	no	up	0.67	no	0.86	no	down	6.2E-12	yes
MSTRG.10830.3 : Phc3 : D3ZS50	0.36	yes	down	4.0E-04	yes	chr2	intron	internal intron	NA	0.55	no	down	0.48	no	0.86	no	down	4.2E-04	yes
ENSRNOT00000015351 : S100a9 : P50116	5.80	yes	up	1.2E-04	yes	chr2	intron	first intron	NA	2.43	yes	up	0.05	yes	1.60	yes	up	6.6E-11	yes
ENSRNOT00000020002 : Anpep : G3V7W7	0.25	yes	down	1.9E-09	yes	chr1	intron	internal intron	NA	1.04	no	up	0.91	no	1.05	no	up	3.0E-08	yes
MSTRG.4992.10 : Top3b : D4A9Z2	11.41	yes	up	1.2E-04	yes	chr11	intron	last intron	NA	1.87	no	up	0.10	no	0.99	no	down	6.1E-01	no
ENSRNOT00000068185 : Ybx3 : D4A0L4	0.20	yes	down	8.8E-05	yes	chr4	intron	internal intron	NA	1.00	no	down	0.99	no	1.24	yes	up	8.2E-11	yes
MSTRG.9610.1 : Rab27b : Q99P74	0	yes	down	1.4E-04	yes	chr18	promoter	Distal	LCP	0.82	no	down	0.10	no	1.04	no	up	7.7E-02	no
MSTRG.8665.4 : Kif13a : D3ZM20	0	yes	down	7.8E-05	yes	chr17	intron	internal intron	NA	1.29	no	up	0.48	no	1.00	no	up	9.5E-01	no
ENSRNOT00000034983 : Rrp7a : D4AE65	1.73	yes	up	1.3E-04	yes	chr7	intron	internal intron	NA	0.94	no	down	0.87	no	0.94	no	down	1.5E-02	yes
MSTRG.8877.1 : Hist1h2bd : D3ZWM5	0.90	no	down	5.8E-05	yes	chr17	promoter	Distal	LCP	0.75	no	down	0.28	no	0.74	yes	down	5.5E-11	yes
MSTRG.16938.2 : Lrpprc : F1LM33	0.61	yes	down	7.5E-05	yes	chr6	intron	internal intron	NA	1.00	no	down	0.89	no	0.97	no	down	4.6E-07	yes
MSTRG.1783.10 : Tgfb1i1 : Q99PD6	3.08	yes	up	7.2E-05	yes	chr1	intron	internal intron	NA	0.36	yes	down	0.29	no	1.06	no	up	1.6E-04	yes
MSTRG.6568.2 : Antxr2 : Q00IM8	1.10	no	up	9.8E-05	yes	chr14	intron	internal intron	NA	1.96	no	up	0.61	no	1.01	no	up	3.8E-01	no
MSTRG.15119.1 : Bcl2l13 : D3ZT71	0.35	yes	down	1.0E-05	yes	chr4	exon	last exon	NA	1.05	no	up	0.50	no	1.11	no	up	6.6E-10	yes
ENSRNOT00000060292 : Ptprc : P04157	2.98	yes	up	6.9E-14	yes	chr13	intron	internal intron	NA	1.44	no	up	0.50	no	1.12	no	up	6.1E-15	yes
MSTRG.15276.1 : Chtopl1 : M0RDD7	0.48	yes	down	9.7E-06	yes	chr4	exon	last exon	NA	0.97	no	down	0.33	no	1.25	yes	up	3.9E-09	yes
ENSRNOT00000085601 : Acaa2 : G3V9U2	0.52	yes	down	2.2E-04	yes	chr18	exon	last exon	NA	1.01	no	up	0.07	no	1.00	no	up	7.4E-01	no
MSTRG.18214.1 : Arhgap45 : D4AAI2	5.87	yes	up	2.5E-04	yes	chr7	exon	internal exon	NA	1.47	no	up	0.29	no	1.20	no	up	4.5E-05	yes
MSTRG.11028.2 : Ptx3 : D3ZT94	0.07	yes	down	2.2E-04	yes	chr2	intron	last intron	NA	1.40	no	up	0.25	no	1.08	no	up	3.1E-07	yes
MSTRG.9752.3 : Coq9 : Q68FT1	0.32	yes	down	5.1E-11	yes	chr19	promoter	Distal	LCP	1.30	no	up	0.15	no	0.93	no	down	1.8E-05	yes
MSTRG.8278.39 : Rbpms : F2Z3S5	0.02	yes	down	2.6E-05	yes	chr16	promoter	Proximal	ICP	0.72	no	down	0.41	no	1.06	no	up	4.9E-05	yes
ENSRNOT00000065965 : Mapk10 : A0A0U1RRS7	2.61	yes	up	3.6E-06	yes	chr14	intron	first intron	NA	0.99	no	down	0.41	no	0.56	yes	down	6.7E-04	yes
ENSRNOT00000037164 : Nup214 : M0RBV9	0.13	yes	down	4.7E-04	yes	chr3	intron	internal intron	NA	0.89	no	down	0.45	no	1.01	no	up	5.4E-01	no
MSTRG.13103.1 : Itga4 : D3ZMQ3	0.24	yes	down	2.1E-07	yes	chr3	intron	internal intron	NA	0.70	no	down	0.24	no	0.99	no	down	7.5E-03	yes
ENSRNOT00000086550 : Plec : F7F9U6	0.33	yes	down	4.2E-06	yes	chr7	exon	last exon	NA	1.00	no	down	0.70	no	0.90	no	down	2.5E-03	yes
ENSRNOT00000017900 : Ireb2 : Q62751	0.22	yes	down	5.8E-05	yes	chr8	exon	internal exon	NA	1.14	no	up	0.51	no	1.28	yes	up	1.3E-04	yes
MSTRG.5704.4 : RGD1306556 : A0A0G2JZ88	0.24	yes	down	1.7E-04	yes	chr12	exon	internal exon	NA	1.03	no	up	0.97	no	1.28	yes	up	1.1E-07	yes
MSTRG.4508.7 : Dyrk1a : Q63470	0.26	yes	down	2.2E-05	yes	chr11	intron	internal intron	NA	0.50	yes	down	0.45	no	1.02	no	up	1.6E-01	no
MSTRG.10325.4 : Cast : F1LPH1	0.36	yes	down	1.6E-06	yes	chr2	intron	internal intron	NA	1.04	no	up	0.60	no	0.95	no	down	2.4E-10	yes
MSTRG.20000.4 : Myo5a : A0A0G2K9S4	1.92	yes	up	1.3E-04	yes	chr8	exon	internal exon	NA	0.99	no	down	0.95	no	1.12	no	up	2.4E-10	yes
MSTRG.4725.2 : Kalrn : P97924	1.54	yes	up	9.1E-07	yes	chr11	exon	last exon	NA	1.11	no	up	0.61	no	1.04	no	up	1.6E-04	yes
ENSRNOT00000033917 : Fdps : F1LND7	inf	yes	up	2.2E-05	yes	chr2	exon	last exon	NA	1.04	no	up	0.69	no	1.07	no	up	1.2E-03	yes
MSTRG.11263.3 : Atp8b2 : D4A509	0.14	yes	down	3.1E-04	yes	chr2	exon	first exon	NA	1.35	no	up	0.60	no	0.92	no	down	5.0E-03	yes
MSTRG.17010.3 : Ttc27 : D3ZTG2	0.43	yes	down	8.1E-09	yes	chr6	exon	internal exon	NA	1.44	no	up	0.49	no	1.02	no	up	1.3E-01	no
ENSRNOT00000082486 : Thop1 : P24155	0.11	yes	down	2.2E-07	yes	chr7	exon	last exon	NA	1.02	no	up	0.77	no	0.92	no	down	9.5E-13	yes
MSTRG.8992.2 : Dip2c : D3ZZB0	0.67	yes	down	4.5E-04	yes	chr17	intron	internal intron	NA	0.38	yes	down	0.19	no	0.88	no	down	3.3E-04	yes
MSTRG.1974.3 : Nap1l4 : Q5U2Z3	0.36	yes	down	7.2E-05	yes	chr1	intron	internal intron	NA	3.62	yes	up	0.21	no	0.98	no	down	3.1E-02	yes
ENSRNOT00000058906 : Tle1 : A0A0G2K324	0.11	yes	down	4.0E-05	yes	chr5	exon	last exon	NA	0.96	no	down	0.71	no	0.95	no	down	1.3E-05	yes
ENSRNOT00000080694 : Hydin : D3ZBX6	3.95	yes	up	2.0E-07	yes	chr19	intron	internal intron	NA	0.99	no	down	0.43	no	0.80	yes	down	2.3E-10	yes
ENSRNOT00000084729 : Scamp1 : A0A0G2K1I6	11.34	yes	up	2.9E-04	yes	chr2	intron	first intron	NA	1.06	no	up	0.47	no	1.05	no	up	3.2E-06	yes
ENSRNOT00000078271 : Sec14l4 : A0A0G2JWI8	0.38	yes	down	1.3E-04	yes	chr14	intron	internal intron	NA	0.70	no	down	0.37	no	0.91	no	down	1.1E-08	yes
ENSRNOT00000006350 : Zeb2 : A0A0G2K8T6	0.34	yes	down	8.0E-09	yes	chr3	intron	internal intron	NA	1.30	no	up	0.74	no	1.01	no	up	2.4E-01	no
ENSRNOT00000078861 : Clu : G3V836	0.38	yes	down	6.6E-05	yes	chr15	intron	first intron	NA	0.64	no	down	0.16	no	1.26	yes	up	5.8E-16	yes
MSTRG.16610.2 : Ece1 : Q6IN10	0.58	yes	down	9.3E-05	yes	chr5	intron	internal intron	NA	0.83	no	down	0.23	no	1.14	no	up	8.3E-15	yes
MSTRG.20817.11 : Map4k4 : A0A0G2K7W4	0.36	yes	down	7.1E-05	yes	chr9	intron	internal intron	NA	0.98	no	down	0.95	no	1.10	no	up	7.1E-06	yes
MSTRG.4725.13 : Kalrn : P97924	1.54	yes	up	9.1E-07	yes	chr11	exon	last exon	NA	0.89	no	down	0.35	no	1.04	no	up	1.6E-04	yes
MSTRG.1683.12 : Il4r : Q63257	9.80	yes	up	2.9E-08	yes	chr1	intron	internal intron	NA	0.81	no	down	0.42	no	1.11	no	up	2.1E-05	yes
ENSRNOT00000071906 : Rpl30l1 : M0RD99	4.10	yes	up	1.5E-08	yes	chr9	intron	last intron	NA	1.00	no	down	0.74	no	0.97	no	down	6.7E-01	no
ENSRNOT00000015620 : Washc2c : F1LPG9	0.05	yes	down	2.7E-19	yes	chr4	exon	internal exon	NA	1.02	no	up	0.83	no	1.05	no	up	2.1E-05	yes
ENSRNOT00000076623 : Smndc1 : Q4QQU6	inf	yes	up	1.3E-04	yes	chr1	promoter	Distal	LCP	1.06	no	up	0.83	no	1.06	no	up	2.2E-04	yes
MSTRG.19472.1 : Dnm2 : A0A0A0MY48	0.11	yes	down	8.9E-11	yes	chr8	intron	first intron	NA	1.21	no	up	0.20	no	1.05	no	up	2.0E-08	yes
ENSRNOT00000090816 : Rock2 : F1LQT3	0.10	yes	down	2.0E-24	yes	chr6	intron	internal intron	NA	1.62	no	up	0.54	no	0.97	no	down	1.9E-04	yes
ENSRNOT00000084506 : Clasp1 : F1LNR1	0.36	yes	down	6.7E-07	yes	chr13	intron	internal intron	NA	1.04	no	up	0.46	no	1.00	no	down	7.0E-01	no
ENSRNOT00000017592 : Tram2 : F1LUA2	3	yes	up	2.1E-05	yes	chr9	intron	first intron	NA	1.62	no	up	0.11	no	1.02	no	up	1.2E-01	no
MSTRG.8552.6 : Ddx46 : Q62780	0.56	yes	down	3.4E-04	yes	chr17	intron	internal intron	NA	1.00	no	down	0.86	no	0.98	no	down	1.3E-01	no
ENSRNOT00000016563 : Cnot1 : G3V7M0	0.08	yes	down	2.6E-04	yes	chr19	intron	internal intron	NA	0.83	no	down	0.76	no	1.01	no	up	1.2E-01	no
MSTRG.3515.3 : Slc43a2 : D3ZDC2	17.66	yes	up	2.2E-19	yes	chr10	intron	internal intron	NA	1.67	no	up	0.35	no	1.39	yes	up	5.1E-08	yes
ENSRNOT00000003914 : Luc7l3 : D3ZFB2	0.31	yes	down	4.3E-04	yes	chr10	intron	internal intron	NA	1.00	no	down	0.97	no	0.98	no	down	9.6E-04	yes
ENSRNOT00000049247 : Gtpbp1 : D2XV59	2.18	yes	up	7.2E-06	yes	chr7	exon	last exon	NA	0.93	no	down	0.18	no	0.97	no	down	3.4E-03	yes
MSTRG.16327.2 : Macf1 : A0A0G2K9T4	0.36	yes	down	1.3E-05	yes	chr5	exon	last exon	NA	1.53	no	up	0.62	no	0.97	no	down	9.0E-11	yes
MSTRG.877.1 : Lsm14a : A0A0G2JUK2	0.31	yes	down	3.8E-04	yes	chr1	intron	last intron	NA	1.07	no	up	0.87	no	1.02	no	up	2.8E-01	no
MSTRG.13167.3 : Nup160 : D3ZBL6	14.66	yes	up	4.1E-04	yes	chr3	intron	internal intron	NA	0.92	no	down	0.84	no	1.00	no	down	6.2E-01	no
MSTRG.4207.1 : Wbp2 : G3V721	4.13	yes	up	1.7E-04	yes	chr10	promoter	Intermediate	LCP	1.13	no	up	0.38	no	0.89	no	down	6.1E-15	yes
MSTRG.162.3 : Tpd52l1 : Q499Q2	2.69	yes	up	6.5E-28	yes	chr1	promoter	Distal	LCP	1.25	no	up	0.47	no	0.97	no	down	5.0E-01	no
MSTRG.15715.3 : Dnajb5 : D3ZB76	0.05	yes	down	2.8E-05	yes	chr5	promoter	Distal	ICP	1.12	no	up	0.87	no	1.02	no	up	2.3E-01	no
ENSRNOT00000054775 : Ptar1 : D3ZWG9	2.31	yes	up	2.3E-04	yes	chr1	exon	internal exon	NA	0.91	no	down	0.29	no	0.95	no	down	1.2E-02	yes
ENSRNOT00000016317 : Masp2 : A2VCV7	0.27	yes	down	4.0E-08	yes	chr5	intron	internal intron	NA	0.94	no	down	0.55	no	1.00	no	down	8.1E-01	no
ENSRNOT00000076833 : Znfx1 : F1LMA9	20.79	yes	up	3.0E-04	yes	chr3	intron	internal intron	NA	0.98	no	down	0.35	no	0.92	no	down	1.0E-03	yes
MSTRG.16636.8 : Ubr4 : A0A0G2JU89	2.84	yes	up	3.7E-05	yes	chr5	intron	internal intron	NA	2.23	yes	up	0.28	no	1.02	no	up	4.6E-05	yes
MSTRG.3538.1 : Abr : A0A0G2JZZ7	inf	yes	up	3.7E-04	yes	chr10	intron	internal intron	NA	0.95	no	down	0.92	no	1.05	no	up	1.0E-03	yes
ENSRNOT00000083363 : Nedd4l : F1LRN8	2.03	yes	up	3.6E-05	yes	chr18	intron	internal intron	NA	1.13	no	up	0.86	no	0.95	no	down	3.9E-02	yes
ENSRNOT00000010007 : Fhl3 : D3ZPF0	0.20	yes	down	2.5E-09	yes	chr5	exon	last exon	NA	0.89	no	down	0.48	no	1.28	yes	up	1.8E-14	yes
ENSRNOT00000077189 : Gtf2i : Q5U2Y1	5.31	yes	up	4.9E-06	yes	chr12	promoter	Distal	LCP	1.28	no	up	0.13	no	0.95	no	down	1.5E-07	yes
MSTRG.16317.6 : Cap1 : Q08163	0.21	yes	down	1.6E-07	yes	chr5	intron	internal intron	NA	0.94	no	down	0.86	no	0.94	no	down	1.0E-08	yes
MSTRG.2839.6 : Srrm2 : A0A0G2K2M9	1.72	yes	up	9.0E-05	yes	chr10	exon	internal exon	NA	0.76	no	down	0.55	no	1.00	no	up	9.0E-01	no
ENSRNOT00000006505 : Ttc27 : D3ZTG2	0.43	yes	down	8.1E-09	yes	chr6	exon	internal exon	NA	1.02	no	up	0.89	no	1.02	no	up	1.3E-01	no
MSTRG.11265.3 : Ubap2l : E9PTR4	6.62	yes	up	4.0E-04	yes	chr2	intron	internal intron	NA	0.64	no	down	0.20	no	1.13	no	up	3.8E-12	yes
ENSRNOT00000072915 : Mef2a : A0A0G2JSZ4	0.04	yes	down	2.4E-04	yes	chr1	exon	last exon	NA	1.57	no	up	0.21	no	0.78	yes	down	4.1E-07	yes
MSTRG.5511.1 : Pitpnm2 : A0A0G2JW50	2.70	yes	up	3.9E-04	yes	chr12	promoter	Distal	LCP	0.83	no	down	0.30	no	0.89	no	down	1.9E-08	yes
MSTRG.2440.1 : Lipa : Q64194	35.26	yes	up	5.1E-18	yes	chr1	intron	internal intron	NA	1.24	no	up	0.70	no	1.02	no	up	6.9E-02	no
ENSRNOT00000060977 : Rapgef1 : F1M8L9	inf	yes	up	4.7E-05	yes	chr3	intron	internal intron	NA	0.63	no	down	0.19	no	0.88	no	down	4.1E-04	yes
MSTRG.21039.1 : Tns1 : F1LN42	0.09	yes	down	2.0E-04	yes	chr9	intron	last intron	NA	0.68	no	down	0.19	no	0.86	no	down	7.8E-19	yes
ENSRNOT00000030782 : Chuk : B5DF32	24.13	yes	up	2.0E-05	yes	chr1	intron	internal intron	NA	1.23	no	up	0.27	no	0.90	no	down	8.1E-05	yes
MSTRG.21039.10 : Tns1 : F1LN42	0.09	yes	down	2.0E-04	yes	chr9	intron	last intron	NA	0.45	yes	down	0.40	no	0.86	no	down	7.8E-19	yes
MSTRG.9206.1 : Ss18 : Q5XI66	6.27	yes	up	1.6E-10	yes	chr18	exon	internal exon	NA	1.06	no	up	0.80	no	1.04	no	up	6.9E-02	no

MSTRG.12646.6 : Rapgef1 : F1M8L9	inf	yes	up	4.7E-05	yes	chr3	intron	internal intron	NA	1.19	no	up	0.85	no	0.88	no	down	4.1E-04	yes
ENSRNOT00000078285 : Kpna1 : P83953	32	yes	up	7.2E-05	yes	chr11	promoter	Distal	LCP	0.93	no	down	0.35	no	1.11	no	up	5.7E-08	yes
MSTRG.20352.1 : Ubp1 : D4A030	0.44	yes	down	2.3E-04	yes	chr8	intron	internal intron	NA	0.86	no	down	0.77	no	0.98	no	down	1.9E-01	no
ENSRNOT00000020886 : Ppp6r3 : D3ZBT9	3.21	yes	up	8.1E-07	yes	chr1	intron	internal intron	NA	1.00	no	down	0.41	no	0.98	no	down	7.0E-03	yes
MSTRG.17520.3 : Srsf5 : Q09167	0.10	yes	down	3.0E-09	yes	chr6	promoter	Proximal	ICP	1.85	no	up	0.37	no	1.03	no	up	4.2E-02	yes
MSTRG.8094.5 : Atp13a1 : B5DEX7	3.83	yes	up	1.1E-13	yes	chr16	intron	internal intron	NA	0.91	no	down	0.81	no	1.09	no	up	4.2E-12	yes
MSTRG.10871.10 : Dcun1d1 : D3ZRV0	1.60	yes	up	3.7E-04	yes	chr2	exon	internal exon	NA	1.01	no	up	0.21	no	0.97	no	down	2.6E-02	yes
ENSRNOT00000057585 : Fn1 : F1LST1	0.11	yes	down	7.7E-11	yes	chr9	intron	internal intron	NA	1.31	no	up	0.79	no	1.27	yes	up	4.1E-20	yes
MSTRG.14930.4 : Grip2 : A0A0H2UHH8	2.18	yes	up	2.2E-05	yes	chr4	intron	internal intron	NA	1.06	no	up	0.48	no	1.01	no	up	5.2E-01	no
MSTRG.6746.4 : Pds5a : A4L9P7	1.26	yes	up	1.3E-13	yes	chr14	exon	last exon	NA	0.91	no	down	0.46	no	1.01	no	up	2.7E-01	no
MSTRG.8351.7 : Adam9 : E9PTA4	0.15	yes	down	9.7E-05	yes	chr16	intron	internal intron	NA	0.60	no	down	0.35	no	1.06	no	up	3.4E-02	yes
ENSRNOT00000090644 : Fgl1 : A0A0G2KA83	5.57	yes	up	1.8E-24	yes	chr16	intron	first intron	NA	1.06	no	up	0.34	no	1.15	no	up	6.9E-07	yes
MSTRG.4105.2 : Smurf2 : F1M3F2	3.77	yes	up	9.3E-06	yes	chr10	intron	internal intron	NA	1.06	no	up	0.49	no	1.03	no	up	6.3E-02	no
ENSRNOT00000088885 : Hint3 : A0A0G2K7W8	0.41	yes	down	1.7E-04	yes	chr1	intron	internal intron	NA	1.06	no	up	0.43	no	1.02	no	up	2.1E-01	no
MSTRG.14930.7 : Grip2 : A0A0H2UHH8	2.18	yes	up	2.2E-05	yes	chr4	intron	internal intron	NA	0.81	no	down	0.34	no	1.01	no	up	5.2E-01	no
MSTRG.11953.30 : RT1-CE7 : D3ZLE6	inf	yes	up	7.3E-07	yes	chr20	exon	internal exon	NA	1.03	no	up	0.84	no	1.22	yes	up	7.3E-16	yes
MSTRG.8345.12 : Fgfr1 : F1LM54	0.22	yes	down	3.0E-05	yes	chr16	exon	last exon	NA	1.83	no	up	0.48	no	1.05	no	up	1.3E-02	yes
ENSRNOT00000013933 : Map2k1 : Q01986	3.88	yes	up	3.2E-04	yes	chr8	exon	last exon	NA	1.15	no	up	0.24	no	1.06	no	up	2.7E-09	yes
ENSRNOT00000003837 : Cdc42bpa : G3V6C9	8.92	yes	up	2.1E-05	yes	chr13	intron	internal intron	NA	0.68	no	down	0.49	no	0.97	no	down	2.5E-04	yes
ENSRNOT00000021925 : Ptdss2 : B2GV22	0.17	yes	down	6.9E-07	yes	chr1	intron	internal intron	NA	1.10	no	up	0.83	no	0.94	no	down	7.1E-04	yes
MSTRG.4751.1 : Pcyt1a : P19836	0.29	yes	down	1.2E-05	yes	chr11	exon	first exon	NA	0.82	no	down	0.25	no	0.94	no	down	3.3E-07	yes
MSTRG.10716.2 : Trio : F1M0Z1	0.35	yes	down	1.6E-06	yes	chr2	exon	first exon	NA	1.14	no	up	0.90	no	1.02	no	up	2.1E-02	yes
ENSRNOT00000046262 : Nf1 : F1LM28	2.12	yes	up	4.3E-05	yes	chr10	intron	internal intron	NA	1.02	no	up	0.73	no	0.98	no	down	1.9E-01	no
ENSRNOT00000068633 : Aox1 : F1LRQ1	0.36	yes	down	1.5E-07	yes	chr9	intron	internal intron	NA	1.13	no	up	0.80	no	0.73	yes	down	3.5E-17	yes
ENSRNOT00000087061 : Bax : Q9JKL3	8.53	yes	up	7.6E-07	yes	chr1	promoter	Distal	LCP	1.16	no	up	0.42	no	1.07	no	up	5.8E-08	yes
MSTRG.21205.2 : Ube2f : Q5U203	7.18	yes	up	7.2E-05	yes	chr9	intron	internal intron	NA	0.98	no	down	0.47	no	1.01	no	up	5.6E-01	no
MSTRG.10517.2 : Erbin : M0R9T2	3.86	yes	up	2.1E-05	yes	chr2	intron	last intron	NA	0.94	no	down	0.68	no	0.94	no	down	3.9E-05	yes
MSTRG.6713.7 : Fryl : D3ZQY4	2.76	yes	up	1.7E-04	yes	chr14	intron	internal intron	NA	1.34	no	up	0.47	no	1.00	no	down	4.6E-01	no
ENSRNOT00000005100 : Slc43a2 : D3ZDC2	17.66	yes	up	2.2E-19	yes	chr10	intron	internal intron	NA	0.95	no	down	0.91	no	1.39	yes	up	5.1E-08	yes
ENSRNOT00000054699 : Cnnm2 : Q5U2P1	1.24	yes	up	9.1E-07	yes	chr1	intron	first intron	NA	1.39	no	up	0.34	no	1.05	no	up	1.5E-03	yes
MSTRG.19189.1 : Rpap3 : Q68FQ7	0.29	yes	down	4.9E-05	yes	chr7	intron	internal intron	NA	0.92	no	down	0.65	no	1.04	no	up	2.9E-04	yes
ENSRNOT00000011552 : Tbx3 : A0A0G2K8D7	2.83	yes	up	2.0E-10	yes	chr12	intron	internal intron	NA	0.90	no	down	0.85	no	0.99	no	down	7.3E-01	no
ENSRNOT00000023854 : Sf3b3 : E9PT66	0.34	yes	down	3.6E-04	yes	chr19	exon	last exon	NA	1.09	no	up	0.71	no	0.97	no	down	3.1E-07	yes
MSTRG.19928.5 : Herc1 : A0A0G2JTT6	10.78	yes	up	2.7E-04	yes	chr8	intron	internal intron	NA	2.25	yes	up	0.28	no	1.04	no	up	5.4E-02	no
MSTRG.9889.5 : Nf1x : F2Z3R4	0.58	yes	down	1.9E-06	yes	chr19	intron	internal intron	NA	0.63	no	down	0.50	no	0.77	yes	down	2.4E-14	yes
ENSRNOT00000028646 : Rasgrp2 : P0C643	0.26	yes	down	2.6E-04	yes	chr1	exon	internal exon	NA	1.33	no	up	0.31	no	1.04	no	up	7.0E-02	no
MSTRG.11953.34 : RT1-CE7 : D3ZLE6	inf	yes	up	7.3E-07	yes	chr20	exon	internal exon	NA	1.02	no	up	0.91	no	1.22	yes	up	7.3E-16	yes
MSTRG.3861.1 : Kpnb1 : F2Z3Q8	17.89	yes	up	1.8E-04	yes	chr10	intron	internal intron	NA	0.62	no	down	0.47	no	0.97	no	down	1.9E-07	yes
ENSRNOT00000014709 : Cast : F1LPH1	0.36	yes	down	1.6E-06	yes	chr2	intron	internal intron	NA	0.99	no	down	0.50	no	0.95	no	down	2.4E-10	yes
MSTRG.13130.2 : Calcrl : Q63118	2.81	yes	up	1.2E-04	yes	chr3	intron	internal intron	NA	0.56	no	down	0.50	no	1.09	no	up	1.2E-04	yes
ENSRNOT00000087439 : Rbms1 : A0A0G2K4R7	0	yes	down	3.3E-05	yes	chr3	intron	internal intron	NA	1.01	no	up	0.48	no	0.97	no	down	1.2E-01	no
ENSRNOT00000090727 : Lmo7 : A0A0G2K174	3.35	yes	up	1.2E-04	yes	chr15	intron	internal intron	NA	2.54	yes	up	0.00	yes	0.96	no	down	4.2E-06	yes
ENSRNOT00000086226 : Kif1b : A0A0G2KA12	3.65	yes	up	7.4E-05	yes	chr5	intron	internal intron	NA	0.86	no	down	0.77	no	1.05	no	up	6.5E-02	no
MSTRG.2057.1 : Sptbn2 : F1MA36	0.33	yes	down	1.2E-08	yes	chr1	intron	internal intron	NA	0.92	no	down	0.04	yes	0.96	no	down	3.4E-01	no
ENSRNOT00000066546 : Flnb : D4A8D5	1.70	yes	up	8.8E-05	yes	chr15	intron	last intron	NA	0.61	no	down	0.02	yes	1.00	no	up	2.4E-01	no
ENSRNOT00000076460 : Parp4 : A0A096MJR6	1.23	yes	up	1.1E-06	yes	chr15	intron	last intron	NA	0.92	no	down	0.11	no	0.95	no	down	6.6E-05	yes
ENSRNOT00000092452 : Upf2 : D3ZT03	0.21	yes	down	9.8E-07	yes	chr17	intron	internal intron	NA	1.09	no	up	0.56	no	1.03	no	up	3.0E-03	yes
MSTRG.14960.5 : Eogt : Q5NDL0	6.71	yes	up	1.7E-04	yes	chr4	intron	internal intron	NA	2.18	yes	up	0.10	no	1.10	no	up	2.0E-05	yes
MSTRG.4508.3 : Dyrk1a : Q63470	0.26	yes	down	2.2E-05	yes	chr11	intron	internal intron	NA	0.77	no	down	0.79	no	1.02	no	up	1.6E-01	no
MSTRG.8434.1 : Lamp1 : P14562	0.27	yes	down	5.9E-08	yes	chr16	promoter	Distal	LCP	1.16	no	up	0.62	no	1.10	no	up	5.4E-06	yes
ENSRNOT00000048044 : Tpm1 : Q923Z2	9.41	yes	up	5.1E-14	yes	chr8	intron	internal intron	NA	0.70	no	down	0.01	yes	0.75	yes	down	6.0E-18	yes
MSTRG.20157.4 : Dzip1l : Q5XIA0	0.67	yes	down	4.1E-05	yes	chr8	intron	internal intron	NA	0.63	no	down	0.15	no	1.09	no	up	1.8E-03	yes
MSTRG.1443.4 : Arap1 : F1LM60	2.71	yes	up	3.3E-04	yes	chr1	intron	first intron	NA	1.23	no	up	0.33	no	0.99	no	down	2.5E-02	yes
MSTRG.21039.15 : Tns1 : F1LN42	0.09	yes	down	2.0E-04	yes	chr9	intron	last intron	NA	1.46	no	up	0.38	no	0.86	no	down	7.8E-19	yes
ENSRNOT00000093334 : Eftud2 : F1LM66	4.66	yes	up	6.0E-04	yes	chr10	exon	internal exon	NA	1.31	no	up	0.14	no	1.02	no	up	4.9E-02	yes
MSTRG.19219.2 : Fkbp11 : G3V7V5	inf	yes	up	4.7E-06	yes	chr7	intron	internal intron	NA	0.94	no	down	0.90	no	1.06	no	up	1.6E-07	yes
MSTRG.4992.6 : Top3b : D4A9Z2	11.41	yes	up	1.2E-04	yes	chr11	intron	last intron	NA	0.52	no	down	0.03	yes	0.99	no	down	6.1E-01	no
MSTRG.19735.4 : Atm : A0A0G2K3I0	0.65	yes	down	2.4E-04	yes	chr8	intron	internal intron	NA	0.28	yes	down	0.20	no	1.01	no	up	4.6E-01	no
MSTRG.6746.3 : Pds5a : A4L9P7	1.26	yes	up	1.3E-13	yes	chr14	exon	last exon	NA	0.75	no	down	0.47	no	1.01	no	up	2.7E-01	no
MSTRG.12922.2 : Zeb2 : A0A0G2K8T6	0.34	yes	down	8.0E-09	yes	chr3	intron	internal intron	NA	0.71	no	down	0.59	no	1.01	no	up	2.4E-01	no
ENSRNOT00000041915 : Hacd3 : D4ABI7	0.11	yes	down	1.7E-06	yes	chr8	intron	internal intron	NA	2.02	yes	up	0.55	no	1.02	no	up	6.2E-05	yes
ENSRNOT00000023890 : Nedd4l : F1LRN8	2.03	yes	up	3.6E-05	yes	chr18	intron	internal intron	NA	1.01	no	up	0.09	no	0.95	no	down	3.9E-02	yes
MSTRG.11268.3 : Tpm3 : Q63610	0.37	yes	down	8.0E-05	yes	chr2	intron	internal intron	NA	0.97	no	down	0.91	no	1.11	no	up	3.2E-12	yes
MSTRG.10153.4 : Cmip : A0A0G2JYB9	0.58	yes	down	9.9E-05	yes	chr19	intron	internal intron	NA	0.40	yes	down	0.35	no	0.89	no	down	2.1E-04	yes
MSTRG.11187.1 : Sema4a : A0A1B0GWV9	3.09	yes	up	1.9E-04	yes	chr2	promoter	Intermediate	LCP	0.93	no	down	0.44	no	1.07	no	up	2.4E-05	yes
MSTRG.12584.2 : Pmpca : Q68FX8	0.39	yes	down	1.4E-04	yes	chr3	intron	internal intron	NA	0.77	no	down	0.35	no	1.03	no	up	5.9E-03	yes
MSTRG.20501.1 : Trip10 : P97531	2.19	yes	up	4.1E-04	yes	chr9	intron	last intron	NA	1.00	no	down	0.99	no	0.91	no	down	4.8E-08	yes
MSTRG.5410.5 : Gtf2i : Q5U2Y1	5.31	yes	up	4.9E-06	yes	chr12	promoter	Distal	LCP	0.90	no	down	0.72	no	0.95	no	down	1.5E-07	yes
ENSRNOT00000008150 : Ncstn : Q8CGU6	0.30	yes	down	6.8E-05	yes	chr13	intron	first intron	NA	1.05	no	up	0.67	no	1.02	no	up	4.0E-02	yes
MSTRG.5906.6 : Atp2b4 : Q64542	2.31	yes	up	1.3E-05	yes	chr13	intron	internal intron	NA	1.12	no	up	0.52	no	0.68	yes	down	2.6E-19	yes
MSTRG.256.1 : Syne1 : Q8VHJ9	0.04	yes	down	1.5E-19	yes	chr1	exon	first exon	NA	1.32	no	up	0.77	no	0.89	no	down	8.9E-14	yes
MSTRG.13870.5 : Plcg1 : G3V845	0.24	yes	down	1.1E-04	yes	chr3	intron	first intron	NA	0.78	no	down	0.43	no	0.94	no	down	1.8E-09	yes
ENSRNOT00000057641 : Tpm1 : Q923Z2	9.41	yes	up	5.1E-14	yes	chr8	intron	internal intron	NA	0.59	no	down	0.35	no	0.75	yes	down	6.0E-18	yes
ENSRNOT00000014435 : Med17 : D4ABU0	3.31	yes	up	2.5E-04	yes	chr8	intron	last intron	NA	1.22	no	up	0.08	no	0.89	no	down	7.9E-05	yes
ENSRNOT00000011513 : Myo1f : D4A7X9	0.24	yes	down	1.1E-14	yes	chr7	intron	internal intron	NA	1.45	no	up	0.12	no	1.28	yes	up	9.7E-15	yes
MSTRG.8278.6 : Rbpms : F2Z3S5	0.02	yes	down	2.6E-05	yes	chr16	promoter	Proximal	ICP	0.54	no	down	0.40	no	1.06	no	up	4.9E-05	yes
ENSRNOT00000030327 : Tpd52l1 : Q499Q2	2.69	yes	up	6.5E-28	yes	chr1	promoter	Distal	LCP	0.95	no	down	0.56	no	0.97	no	down	5.0E-01	no
MSTRG.13265.2 : Tcp11l1 : F1M9Y7	3.13	yes	up	1.2E-06	yes	chr3	intron	last intron	NA	0.90	no	down	0.69	no	0.99	no	down	8.5E-01	no
MSTRG.14995.2 : Itpr1 : A0A0A0MY31	3.16	yes	up	1.1E-05	yes	chr4	intron	internal intron	NA	1.21	no	up	0.84	no	0.87	no	down	1.1E-14	yes
ENSRNOT00000082152 : Syne3 : D3ZD24	0.24	yes	down	2.1E-14	yes	chr6	intron	internal intron	NA	0.98	no	down	0.88	no	1.11	no	up	3.8E-04	yes
MSTRG.11953.54 : RT1-CE7 : D3ZLE6	inf	yes	up	7.3E-07	yes	chr20	exon	internal exon	NA	1.15	no	up	0.72	no	1.22	yes	up	7.3E-16	yes
ENSRNOT00000038480 : Krt8 : Q10758	0.19	yes	down	1.4E-04	yes	chr7	exon	last exon	NA	0.56	no	down	0.07	no	0.83	yes	down	4.5E-14	yes
MSTRG.1443.12 : Arap1 : F1LM60	2.71	yes	up	3.3E-04	yes	chr1	intron	first intron	NA	1.31	no	up	0.38	no	0.99	no	down	2.5E-02	yes
MSTRG.4725.8 : Kalrn : P97924	1.54	yes	up	9.1E-07	yes	chr11	exon	last exon	NA	1.22	no	up	0.11	no	1.04	no	up	1.6E-04	yes
MSTRG.17232.2 : Lamb1 : D3ZQN7	3.86	yes	up	8.9E-05	yes	chr6	intron	internal intron	NA	3.85	yes	up	0.02	yes	1.31	yes	up	5.4E-17	yes
MSTRG.6192.1 : Uap1 : D3ZF39	0.41	yes	down	2.7E-06	yes	chr13	intron	internal intron	NA	0.81	no	down	0.22	no	1.13	no	up	4.6E-12	yes
MSTRG.12973.4 : Rbms1 : A0A0G2K4R7	0	yes	down	3.3E-05	yes	chr3	intron	internal intron	NA	1.17	no	up	0.56	no	0.97	no	down	1.2E-01	no
ENSRNOT00000054952 : Pcyt2 : O88637	4.82	yes	up	3.0E-05	yes	chr10	intron	last intron	NA	0.55	no	down	0.23	no	0.94	no	down	1.8E-03	yes
MSTRG.17715.1 : Syne3 : D3ZD24	0.24	yes	down	2.1E-14	yes	chr6	intron	internal intron	NA	0.86	no	down	0.10	no	1.11	no	up	3.8E-04	yes
MSTRG.21083.10 : Speg : Q63638	0.17	yes	down	2.6E-06	yes	chr9	intron	internal intron	NA	1.07	no	up	0.86	no	0.88	no	down	2.3E-07	yes
MSTRG.12174.4 : Col18a1 : F1LR02	0.22	yes	down	4.1E-11	yes	chr20	exon	internal exon	NA	2.42	yes	up	0.47	no	1.03	no	up	3.6E-07	yes
MSTRG.14387.1 : Tnpo3 : D4AAM0	6.44	yes	up	4.2E-12	yes	chr4	promoter	Distal	LCP	1.46	no	up	0.17	no	1.04	no	up	4.3E-04	yes
MSTRG.19538.6 : Nfrkb : D4A421	0.58	yes	down	1.3E-05	yes	chr8	intron	internal intron	NA	1.17	no	up	0.43	no	0.89	no	down	2.5E-03	yes
MSTRG.3849.3 : Pnpo : O88794	2.62	yes	up	1.2E-06	yes	chr10	intron	internal intron	NA	1.04	no	up	0.89	no	0.99	no	down	6.7E-01	no
MSTRG.20352.10 : Ubp1 : D4A030	0.44	yes	down	2.3E-04	yes	chr8	intron	internal intron	NA	0.98	no	down	0.96	no	0.98	no	down	1.9E-01	no
MSTRG.21023.2 : Fn1 : F1LST1	0.11	yes	down	7.7E-11	yes	chr9	intron	internal intron	NA	1.20	no	up	0.84	no	1.27	yes	up	4.1E-20	yes
ENSRNOT00000047234 : Fbln1 : B1WC21	0.23	yes	down	7.2E-09	yes	chr7	intron	internal intron	NA	1.22	no	up	0.68	no	1.15	no	up	5.3E-06	yes
MSTRG.8278.30 : Rbpms : F2Z3S5	0.02	yes	down	2.6E-05	yes	chr16	promoter	Proximal	ICP	1.08	no	up	0.14	no	1.06	no	up	4.9E-05	yes
ENSRNOT00000083427 : Rpl14 : Q63507	6.79	yes	up	7.4E-07	yes	chr8	intron	internal intron	NA	0.89	no	down	0.24	no	1.09	no	up	1.1E-04	yes
ENSRNOT00000038703 : Fat4 : D3ZEH1	10.57	yes	up	1.5E-05	yes	chr2	intron	internal intron	NA	0.96	no	down	0.32	no	0.96	no	down	1.0E-02	yes

MSTRG.659.1 : Cic : D4A853	4.32	yes	up	3.8E-07	yes	chr1	exon	internal exon	NA	1.13	no	up	0.59	no	1.06	no	up	4.3E-02	yes
MSTRG.19928.4 : Herc1 : A0A0G2JTT6	10.78	yes	up	2.7E-04	yes	chr8	intron	internal intron	NA	0.71	no	down	0.38	no	1.04	no	up	5.4E-02	no
MSTRG.18971.2 : Triobp : A2TIS7	0.29	yes	down	2.0E-06	yes	chr7	intron	internal intron	NA	1.23	no	up	0.06	no	0.97	no	down	5.1E-06	yes
MSTRG.18451.1 : Atp2b1 : P11505	4.09	yes	up	4.6E-11	yes	chr7	exon	last exon	NA	2.12	yes	up	0.26	no	1.04	no	up	4.5E-06	yes
ENSRNOT00000093370 : Mycbp2 : D4A2D3	0.31	yes	down	9.2E-06	yes	chr15	intron	internal intron	NA	0.94	no	down	0.81	no	1.00	no	up	6.8E-01	no
MSTRG.20752.3 : Ptpn18 : Q4KM54	0	yes	down	1.9E-04	yes	chr9	intron	first intron	NA	0.98	no	down	0.94	no	1.20	no	up	1.2E-08	yes
MSTRG.1683.9 : Il4r : Q63257	9.80	yes	up	2.9E-08	yes	chr1	intron	internal intron	NA	0.50	yes	down	0.18	no	1.11	no	up	2.1E-05	yes
MSTRG.21023.3 : Fn1 : F1LST1	0.11	yes	down	7.7E-11	yes	chr9	intron	internal intron	NA	1.42	no	up	0.73	no	1.27	yes	up	4.1E-20	yes
MSTRG.22041.2 : Dkc1 : P40615	0.28	yes	down	2.4E-05	yes	chrX	intron	internal intron	NA	1.21	no	up	0.60	no	0.99	no	down	3.6E-01	no
ENSRNOT00000088416 : Gsta3 : P04904	2.74	yes	up	2.3E-04	yes	chr9	exon	last exon	NA	0.76	no	down	0.31	no	0.64	yes	down	2.9E-15	yes
ENSRNOT00000006946 : Gnptab : D3ZKE0	0.07	yes	down	6.5E-07	yes	chr7	intron	first intron	NA	1.09	no	up	0.35	no	0.80	yes	down	3.3E-05	yes
MSTRG.19735.2 : Atm : A0A0G2K3I0	0.65	yes	down	2.4E-04	yes	chr8	intron	internal intron	NA	1.62	no	up	0.47	no	1.01	no	up	4.6E-01	no
ENSRNOT00000001182 : Auts2 : F1M388	0.04	yes	down	4.8E-14	yes	chr12	intron	internal intron	NA	1.02	no	up	0.70	no	1.01	no	up	5.6E-01	no
MSTRG.13766.3 : Ncoa6 : G3V8C9	7.63	yes	up	1.0E-10	yes	chr3	exon	internal exon	NA	0.52	no	down	0.46	no	1.03	no	up	4.4E-03	yes
ENSRNOT00000020573 : Kmt2a : F1M0L3	0.53	yes	down	1.7E-05	yes	chr8	exon	internal exon	NA	1.08	no	up	0.71	no	0.99	no	down	9.3E-01	no
MSTRG.4262.1 : Afmid : M0RC77	4.72	yes	up	9.0E-06	yes	chr10	promoter	Intermediate	LCP	0.96	no	down	0.87	no	0.81	yes	down	7.4E-05	yes
MSTRG.11265.6 : Ubap2l : E9PTR4	6.62	yes	up	4.0E-04	yes	chr2	intron	internal intron	NA	1.33	no	up	0.10	no	1.13	no	up	3.8E-12	yes
MSTRG.5906.7 : Atp2b4 : Q64542	2.31	yes	up	1.3E-05	yes	chr13	intron	internal intron	NA	0.92	no	down	0.26	no	0.68	yes	down	2.6E-19	yes
ENSRNOT00000077164 : Hmox2 : P23711	inf	yes	up	5.3E-04	yes	chr10	exon	last exon	NA	0.95	no	down	0.48	no	1.09	no	up	4.1E-06	yes
MSTRG.12626.2 : Col5a1 : G3V763	0.33	yes	down	4.5E-04	yes	chr3	intron	internal intron	NA	0.83	no	down	0.80	no	1.08	no	up	2.2E-06	yes
MSTRG.18072.1 : Dgka : P51556	0.72	yes	down	1.5E-04	yes	chr7	exon	internal exon	NA	1.44	no	up	0.33	no	0.95	no	down	8.8E-04	yes
MSTRG.16640.2 : Arhgef10l : M0R7W2	2.87	yes	up	9.9E-07	yes	chr5	intron	internal intron	NA	1.00	no	down	0.98	no	1.13	no	up	8.4E-04	yes
ENSRNOT00000020558 : Uggt1 : Q9JLA3	0.22	yes	down	3.9E-04	yes	chr9	intron	internal intron	NA	1.12	no	up	0.93	no	1.11	no	up	9.8E-15	yes
MSTRG.12945.1 : Fmnl2 : A0A0G2K132	0.84	no	down	6.1E-07	yes	chr3	intron	internal intron	NA	0.75	no	down	0.40	no	1.00	no	down	9.6E-01	no
MSTRG.1443.9 : Arap1 : F1LM60	2.71	yes	up	3.3E-04	yes	chr1	intron	first intron	NA	1.37	no	up	0.28	no	0.99	no	down	2.5E-02	yes
ENSRNOT00000081437 : Filip1 : F1LM79	4.15	yes	up	2.1E-10	yes	chr8	exon	first exon	NA	0.76	no	down	0.02	yes	1.01	no	up	3.0E-01	no
MSTRG.12240.11 : Bicc1 : A0A0G2K0Y0	5.19	yes	up	5.0E-04	yes	chr20	intron	internal intron	NA	0.90	no	down	0.67	no	1.52	yes	up	1.2E-05	yes
MSTRG.19070.2 : Rrp7a : D4AE65	1.73	yes	up	1.3E-04	yes	chr7	intron	internal intron	NA	0.83	no	down	0.52	no	0.94	no	down	1.5E-02	yes
MSTRG.4525.3 : Mx1 : Q499S4	3.65	yes	up	1.1E-13	yes	chr11	intron	internal intron	NA	4.84	yes	up	0.18	no	1.36	yes	up	1.2E-13	yes
MSTRG.8278.4 : Rbpms : F2Z3S5	0.02	yes	down	2.6E-05	yes	chr16	promoter	Proximal	ICP	1.07	no	up	0.74	no	1.06	no	up	4.9E-05	yes
MSTRG.1035.1 : Rasip1 : B5DF05	6.95	yes	up	1.3E-05	yes	chr1	intron	first intron	NA	0.63	no	down	0.23	no	0.96	no	down	1.3E-02	yes
MSTRG.15557.1 : Ints8 : A0A0G2K0V1	0.11	yes	down	2.1E-05	yes	chr5	intron	last intron	NA	1.05	no	up	0.89	no	1.07	no	up	1.6E-05	yes
MSTRG.8345.9 : Fgfr1 : F1LM54	0.22	yes	down	3.0E-05	yes	chr16	exon	last exon	NA	1.11	no	up	0.88	no	1.05	no	up	1.3E-02	yes
ENSRNOT00000005722 : Deptor : F1M8Y4	2.15	yes	up	9.9E-15	yes	chr7	intron	internal intron	NA	0.79	no	down	0.45	no	0.97	no	down	2.0E-01	no
MSTRG.20817.9 : Map4k4 : A0A0G2K7W4	0.36	yes	down	7.1E-05	yes	chr9	intron	internal intron	NA	0.72	no	down	0.22	no	1.10	no	up	7.1E-06	yes
ENSRNOT00000027414 : Tie1 : B5DFD6	1.35	yes	up	2.1E-04	yes	chr5	intron	first intron	NA	2.40	yes	up	0.52	no	0.76	yes	down	4.3E-08	yes
MSTRG.1443.3 : Arap1 : F1LM60	2.71	yes	up	3.3E-04	yes	chr1	intron	first intron	NA	1.07	no	up	0.80	no	0.99	no	down	2.5E-02	yes
ENSRNOT00000090751 : Akap9 : F1LPB4	2.74	yes	up	2.9E-04	yes	chr4	intron	internal intron	NA	1.00	no	down	0.82	no	1.05	no	up	2.3E-05	yes
MSTRG.20963.1 : Nbeal1 : F1M6V0	4.79	yes	up	6.8E-08	yes	chr9	intron	internal intron	NA	1.18	no	up	0.59	no	1.00	no	up	5.1E-01	no
ENSRNOT00000093684 : Nup214 : D4ACK1	0.13	yes	down	4.7E-04	yes	chr3	intron	internal intron	NA	1.05	no	up	0.50	no	0.99	no	down	6.9E-01	no
ENSRNOT00000004787 : Utp3 : Q6AXX4	0.25	yes	down	1.4E-04	yes	chr14	exon	first exon	NA	4.23	yes	up	0.09	no	1.02	no	up	3.8E-01	no
ENSRNOT00000093420 : Nr2f1 : A0A1W2Q6N8	1.84	yes	up	2.6E-07	yes	chr2	intron	last intron	NA	1.01	no	up	0.48	no	1.00	no	up	7.8E-01	no
MSTRG.8772.3 : Serpinb6b : Q68FX2	5.33	yes	up	5.8E-08	yes	chr17	promoter	Distal	LCP	1.27	no	up	0.48	no	0.95	no	down	6.3E-06	yes
MSTRG.8948.4 : Mtpap : D3ZPN5	1.99	yes	up	1.4E-05	yes	chr17	intron	internal intron	NA	1.04	no	up	0.88	no	1.03	no	up	1.2E-01	no
ENSRNOT00000092387 : Tsr1 : D3ZEM8	45.59	yes	up	3.4E-04	yes	chr10	intron	internal intron	NA	1.00	no	down	0.98	no	1.00	no	down	9.7E-01	no
ENSRNOT00000080988 : Aldoa : P05065	0.17	yes	down	1.4E-04	yes	chr1	exon	last exon	NA	0.92	no	down	0.77	no	0.98	no	down	4.2E-08	yes
MSTRG.17811.1 : Mark3 : F1M836	0.30	yes	down	2.6E-04	yes	chr6	exon	last exon	NA	1.03	no	up	0.94	no	1.10	no	up	4.1E-07	yes
MSTRG.19220.14 : Kmt2d : A0A0G2JVD6	6.53	yes	up	2.1E-09	yes	chr7	exon	last exon	NA	1.07	no	up	0.70	no	1.03	no	up	3.5E-02	yes
ENSRNOT00000017453 : Postn : A0A097BW25	0.29	yes	down	1.4E-05	yes	chr2	intron	internal intron	NA	4.85	yes	up	0.11	no	1.23	yes	up	1.4E-16	yes
MSTRG.12934.2 : Nmi : Q498S7	0.14	yes	down	8.2E-08	yes	chr3	exon	last exon	NA	1.58	no	up	0.09	no	1.02	no	up	4.1E-02	yes
MSTRG.15557.4 : Ints8 : A0A0G2K0V1	0.11	yes	down	2.1E-05	yes	chr5	intron	last intron	NA	0.75	no	down	0.50	no	1.07	no	up	1.6E-05	yes
ENSRNOT00000000471 : Asf1a : A0A0G2JTI3	0.41	yes	down	1.4E-04	yes	chr20	intron	last intron	NA	1.00	no	down	0.99	no	0.88	no	down	4.8E-08	yes
MSTRG.3249.8 : Specc1 : A0A0G2K5D7	5.53	yes	up	1.9E-10	yes	chr10	exon	last exon	NA	0.92	no	down	0.46	no	0.93	no	down	4.4E-09	yes
ENSRNOT00000092663 : Srsf3 : A0A0U1RRV7	4.40	yes	up	1.5E-08	yes	chr20	intron	internal intron	NA	1.17	no	up	0.36	no	0.94	no	down	5.5E-08	yes
MSTRG.15963.6 : Ptprd : M0RB22	3.73	yes	up	1.3E-08	yes	chr5	exon	internal exon	NA	1.69	no	up	0.17	no	1.01	no	up	5.3E-01	no
MSTRG.10871.4 : Dcun1d1 : D3ZRV0	1.60	yes	up	3.7E-04	yes	chr2	exon	internal exon	NA	1.13	no	up	0.72	no	0.97	no	down	2.6E-02	yes
ENSRNOT00000074378 : Arhgef10l : M0R7W2	2.87	yes	up	9.9E-07	yes	chr5	intron	internal intron	NA	1.00	no	down	0.56	no	1.13	no	up	8.4E-04	yes
ENSRNOT00000093376 : Gpx4 : A0A0G2K398	0.42	yes	down	9.7E-05	yes	chr7	intron	internal intron	NA	0.94	no	down	0.74	no	0.91	no	down	1.6E-05	yes
ENSRNOT00000001160 : Vars : Q04462	4.99	yes	up	5.5E-05	yes	chr20	intron	internal intron	NA	0.77	no	down	0.62	no	1.01	no	up	2.1E-04	yes
ENSRNOT00000091675 : Klhl22 : D3ZZC3	2.85	yes	up	3.5E-06	yes	chr11	intron	internal intron	NA	0.84	no	down	0.19	no	0.98	no	down	1.1E-01	no
MSTRG.3447.1 : Atp2a3 : G3V9U7	inf	yes	up	2.1E-05	yes	chr10	intron	internal intron	NA	0.43	yes	down	0.48	no	0.77	yes	down	1.7E-16	yes
MSTRG.19895.5 : Hacd3 : D4ABI7	0.11	yes	down	1.7E-06	yes	chr8	intron	internal intron	NA	0.57	no	down	0.27	no	1.02	no	up	6.2E-05	yes
MSTRG.7835.3 : Tm9sf2 : Q66HG5	0.39	yes	down	9.3E-05	yes	chr15	intron	last intron	NA	0.48	yes	down	0.57	no	1.03	no	up	6.1E-04	yes
MSTRG.4030.1 : Eftud2 : F1LM66	4.66	yes	up	6.0E-04	yes	chr10	exon	internal exon	NA	1.97	no	up	0.48	no	1.02	no	up	4.9E-02	yes
MSTRG.15065.3 : Plxnd1 : D4AA77	0.14	yes	down	1.5E-08	yes	chr4	intron	internal intron	NA	0.84	no	down	0.71	no	0.94	no	down	1.4E-01	no
MSTRG.1283.5 : Tm6sf1 : D4A3E7	0.12	yes	down	9.3E-08	yes	chr1	intron	first intron	NA	1.08	no	up	0.64	no	1.05	no	up	5.5E-03	yes
MSTRG.19956.4 : Myo1e : A0A0G2K9E8	3.15	yes	up	9.4E-05	yes	chr8	intron	first intron	NA	1.64	no	up	0.48	no	0.76	yes	down	2.6E-20	yes
MSTRG.22064.10 : Arhgap4 : A0A0G2JVF0	0.34	yes	down	1.4E-05	yes	chrX	intron	internal intron	NA	1.74	no	up	0.19	no	1.07	no	up	7.1E-04	yes
MSTRG.2580.1 : Sfxn3 : Q9JHY2	0.39	yes	down	7.8E-05	yes	chr1	intron	internal intron	NA	1.28	no	up	0.08	no	0.98	no	down	7.5E-03	yes
ENSRNOT00000080703 : Abr : A0A0G2JZZ7	inf	yes	up	3.7E-04	yes	chr10	intron	internal intron	NA	0.81	no	down	0.66	no	1.05	no	up	1.0E-03	yes
MSTRG.17733.1 : Atg2b : Q5EBA2	0.42	yes	down	4.5E-04	yes	chr6	exon	internal exon	NA	0.96	no	down	0.93	no	1.03	no	up	1.2E-01	no
ENSRNOT00000082962 : Med12l : A0A0G2JV69	6.48	yes	up	1.3E-08	yes	chr2	promoter	Distal	LCP	1.09	no	up	0.30	no	0.61	yes	down	2.2E-04	yes
ENSRNOT00000070864 : Mef2a : A0A0G2JSZ4	0.04	yes	down	2.4E-04	yes	chr1	exon	last exon	NA	1.33	no	up	0.18	no	0.78	yes	down	4.1E-07	yes
ENSRNOT00000022650 : Use1 : B0BNG6	3.16	yes	up	2.8E-15	yes	chr16	intron	internal intron	NA	0.75	no	down	0.29	no	1.02	no	up	5.2E-02	no
ENSRNOT00000020307 : Npr1 : P18910	0.37	yes	down	1.2E-04	yes	chr2	intron	internal intron	NA	1.34	no	up	0.24	no	1.02	no	up	1.6E-02	yes
MSTRG.7804.6 : Mycbp2 : D4A2D3	0.31	yes	down	9.2E-06	yes	chr15	intron	internal intron	NA	2.39	yes	up	0.06	no	1.00	no	up	6.8E-01	no
MSTRG.21176.2 : Inpp5d : F1M981	0.08	yes	down	9.9E-05	yes	chr9	intron	internal intron	NA	0.76	no	down	0.29	no	1.08	no	up	3.1E-05	yes
MSTRG.97.16 : Bclaf1 : B1WC16	0.67	yes	down	1.3E-07	yes	chr1	intron	internal intron	NA	0.74	no	down	0.19	no	1.03	no	up	6.8E-02	no
MSTRG.15397.2 : Stk38l : A4GW50	0.61	yes	down	1.5E-06	yes	chr4	intron	internal intron	NA	2.65	yes	up	0.24	no	1.16	no	up	1.1E-11	yes
ENSRNOT00000077742 : Ece1 : Q6IN10	0.58	yes	down	9.3E-05	yes	chr5	intron	internal intron	NA	0.36	yes	down	0.50	no	1.14	no	up	8.3E-15	yes
ENSRNOT00000041645 : Gusb : F1LQQ8	29.04	yes	up	6.5E-05	yes	chr12	intron	internal intron	NA	1.02	no	up	0.29	no	0.97	no	down	1.8E-03	yes
MSTRG.4263.1 : Tnrc6c : D3ZRA6	0.39	yes	down	2.3E-06	yes	chr10	intron	internal intron	NA	3.14	yes	up	0.15	no	1.07	no	up	1.9E-01	no
MSTRG.20218.1 : Rpl29 : P25886	3.86	yes	up	3.0E-04	yes	chr8	intron	first intron	NA	5.99	yes	up	0.01	yes	0.98	no	down	6.0E-01	no
MSTRG.986.5 : Akt1s1 : D3ZH75	2.93	yes	up	2.4E-05	yes	chr1	intron	internal intron	NA	1.23	no	up	0.04	yes	0.97	no	down	7.2E-01	no
MSTRG.5482.16 : Ncor2 : A0A0G2JU91	2.16	yes	up	1.6E-06	yes	chr12	intron	internal intron	NA	1.17	no	up	0.63	no	1.01	no	up	7.1E-01	no
MSTRG.17422.4 : Ppm1a : P20650	2.35	yes	up	3.9E-04	yes	chr6	intron	last intron	NA	1.31	no	up	0.47	no	0.96	no	down	5.3E-04	yes
MSTRG.8514.9 : Zcchc6 : D3ZKR9	0.14	yes	down	1.2E-08	yes	chr17	exon	internal exon	NA	0.81	no	down	0.54	no	1.12	no	up	2.3E-06	yes
MSTRG.14695.1 : Ptcd3 : D3ZGM1	3.88	yes	up	4.1E-35	yes	chr4	intron	internal intron	NA	1.77	no	up	0.45	no	0.98	no	down	3.1E-01	no
MSTRG.8278.7 : Rbpms : F2Z3S5	0.02	yes	down	2.6E-05	yes	chr16	promoter	Proximal	ICP	1.19	no	up	0.68	no	1.06	no	up	4.9E-05	yes
MSTRG.19220.11 : Kmt2d : A0A0G2JVD6	6.53	yes	up	2.1E-09	yes	chr7	exon	last exon	NA	1.29	no	up	0.17	no	1.03	no	up	3.5E-02	yes
ENSRNOT00000016828 : Prim2 : Q4V8C0	4.01	yes	up	2.8E-05	yes	chr9	intron	internal intron	NA	1.23	no	up	0.41	no	1.03	no	up	2.8E-01	no
ENSRNOT00000000640 : Cmtr1 : Q5U2Z5	4.09	yes	up	4.3E-15	yes	chr20	intron	internal intron	NA	1.16	no	up	0.49	no	1.00	no	down	9.7E-01	no
ENSRNOT00000092086 : Picalm : A0A1B0GWW9	8.74	yes	up	2.1E-13	yes	chr1	intron	internal intron	NA	0.60	no	down	0.06	no	1.04	no	up	1.0E-01	no
MSTRG.3949.2 : Dhx58 : D3ZD46	5.00	yes	up	2.1E-04	yes	chr10	exon	internal exon	NA	1.40	no	up	0.66	no	1.22	yes	up	6.3E-08	yes
MSTRG.19119.3 : Gramd4 : F1LYJ8	0.57	yes	down	9.4E-05	yes	chr7	intron	internal intron	NA	0.72	no	down	0.51	no	1.32	yes	up	2.0E-09	yes
MSTRG.9728.5 : Cnot1 : G3V7M0	0.08	yes	down	2.6E-04	yes	chr19	intron	internal intron	NA	0.96	no	down	0.94	no	1.01	no	up	1.2E-01	no
ENSRNOT00000019497 : Aamp : B0K024	0.09	yes	down	2.6E-04	yes	chr9	promoter	Intermediate	LCP	1.01	no	up	0.89	no	1.06	no	up	1.5E-05	yes
ENSRNOT00000016561 : Bcl2l13 : D3ZT71	0.35	yes	down	1.0E-05	yes	chr4	exon	last exon	NA	0.99	no	down	0.96	no	1.11	no	up	6.6E-10	yes
ENSRNOT00000032808 : Cryz : Q6AYT0	3.72	yes	up	1.8E-04	yes	chr2	intron	first intron	NA	0.89	no	down	0.32	no	0.91	no	down	9.1E-09	yes
ENSRNOT00000062052 : Cast : F1LPH1	0.36	yes	down	1.6E-06	yes	chr2	intron	internal intron	NA	1.16	no	up	0.76	no	0.95	no	down	2.4E-10	yes

MSTRG.7803.1 : Mycbp2 : D4A2D3	0.31	yes	down	9.2E-06	yes	chr15	intron	internal intron	NA	0.93	no	down	0.50	no	1.00	no	up	6.8E-01	no
MSTRG.16523.3 : Arid1a : D4A3E3	0.22	yes	down	1.1E-04	yes	chr5	intron	first intron	NA	2.21	yes	up	0.27	no	1.00	no	down	2.1E-01	no
MSTRG.2839.14 : Srrm2 : A0A0G2K2M9	1.72	yes	up	9.0E-05	yes	chr10	exon	internal exon	NA	1.19	no	up	0.71	no	1.00	no	up	9.0E-01	no
MSTRG.9889.12 : Nf1x : F2Z3R4	0.58	yes	down	1.9E-06	yes	chr19	intron	internal intron	NA	0.62	no	down	0.45	no	0.77	yes	down	2.4E-14	yes
MSTRG.7561.10 : Phf11b : M0RB46	3.03	yes	up	2.9E-04	yes	chr15	promoter	Intermediate	LCP	1.42	no	up	0.41	no	1.15	no	up	2.3E-06	yes
MSTRG.9721.5 : Cdh11 : F1MAH6	1.94	yes	up	2.2E-05	yes	chr19	exon	last exon	NA	1.10	no	up	0.09	no	0.91	no	down	6.5E-11	yes
MSTRG.123.8 : Epb41l2 : D3ZM69	0.25	yes	down	1.1E-12	yes	chr1	intron	internal intron	NA	0.72	no	down	0.42	no	0.91	no	down	8.9E-11	yes
ENSRNOT00000077053 : Cdh13 : Q8R490	2.07	yes	up	3.3E-06	yes	chr19	promoter	Intermediate	LCP	1.01	no	up	0.92	no	0.83	yes	down	2.3E-16	yes
MSTRG.20352.2 : Ubp1 : D4A030	0.44	yes	down	2.3E-04	yes	chr8	intron	internal intron	NA	1.15	no	up	0.84	no	0.98	no	down	1.9E-01	no
ENSRNOT00000044116 : Sco1 : B0BNM7	0.18	yes	down	3.1E-08	yes	chr10	promoter	Intermediate	ICP	0.92	no	down	0.46	no	1.00	no	up	8.2E-01	no
ENSRNOT00000073289 : Was : G3V9K5	0.60	yes	down	2.3E-04	yes	chrX	intron	internal intron	NA	1.63	no	up	0.06	no	1.19	no	up	3.0E-08	yes
MSTRG.6812.5 : Pcdh7 : Q68HB8	0	yes	down	4.2E-04	yes	chr14	exon	last exon	NA	1.58	no	up	0.18	no	0.94	no	down	1.0E-07	yes
MSTRG.7226.7 : Sec24c : A0A0G2JZF0	3.50	yes	up	1.3E-09	yes	chr15	intron	internal intron	NA	0.44	yes	down	0.52	no	1.01	no	up	2.6E-01	no
MSTRG.19522.1 : Vps26b : B1WBS4	3.89	yes	up	3.2E-04	yes	chr8	intron	internal intron	NA	0.93	no	down	0.50	no	0.94	no	down	1.6E-09	yes
ENSRNOT00000086473 : Creb5 : A0A0G2K5F0	0.10	yes	down	1.8E-23	yes	chr4	intron	internal intron	NA	0.91	no	down	0.69	no	1.01	no	up	7.4E-01	no
MSTRG.11379.1 : Txnip : Q5M7W1	6.38	yes	up	4.0E-07	yes	chr2	exon	internal exon	NA	0.87	no	down	0.83	no	0.85	no	down	1.0E-06	yes
ENSRNOT00000005996 : Pes1 : Q3B8N8	4.53	yes	up	2.5E-04	yes	chr14	exon	internal exon	NA	1.02	no	up	0.79	no	1.08	no	up	1.0E-03	yes
MSTRG.11955.3 : RT1-CE7 : D3ZLE6	inf	yes	up	7.3E-07	yes	chr20	exon	internal exon	NA	0.73	no	down	0.36	no	1.22	yes	up	7.3E-16	yes
MSTRG.21083.17 : Speg : Q63638	0.17	yes	down	2.6E-06	yes	chr9	intron	internal intron	NA	1.10	no	up	0.63	no	0.88	no	down	2.3E-07	yes
MSTRG.5482.19 : Ncor2 : A0A0G2JU91	2.16	yes	up	1.6E-06	yes	chr12	intron	internal intron	NA	0.93	no	down	0.83	no	1.01	no	up	7.1E-01	no
MSTRG.3515.1 : Slc43a2 : D3ZDC2	17.66	yes	up	2.2E-19	yes	chr10	intron	internal intron	NA	1.01	no	up	0.19	no	1.39	yes	up	5.1E-08	yes
MSTRG.12710.1 : Nup214 : D4ACK1	0.13	yes	down	4.7E-04	yes	chr3	intron	internal intron	NA	1.02	no	up	0.85	no	0.99	no	down	6.9E-01	no
MSTRG.6855.1 : Prom1 : Q9JI49	8.32	yes	up	2.4E-09	yes	chr14	intron	internal intron	NA	0.45	yes	down	0.20	no	0.87	no	down	1.5E-05	yes
MSTRG.307.3 : Igf2r : Q63002	0.32	yes	down	2.3E-06	yes	chr1	promoter	Distal	ICP	1.00	no	down	0.87	no	1.15	no	up	2.6E-11	yes
MSTRG.5482.18 : Ncor2 : A0A0G2JU91	2.16	yes	up	1.6E-06	yes	chr12	intron	internal intron	NA	0.72	no	down	0.16	no	1.01	no	up	7.1E-01	no
ENSRNOT00000090882 : Arhgap26 : A0A0G2K5D5	0.31	yes	down	2.9E-05	yes	chr18	intron	internal intron	NA	1.09	no	up	0.58	no	1.14	no	up	8.4E-08	yes
ENSRNOT00000030186 : Lsm14a : A0A0G2JUK2	0.31	yes	down	3.8E-04	yes	chr1	intron	last intron	NA	2.15	yes	up	0.13	no	1.02	no	up	2.8E-01	no
MSTRG.8278.3 : Rbpms : F2Z3S5	0.02	yes	down	2.6E-05	yes	chr16	promoter	Proximal	ICP	1.61	no	up	0.44	no	1.06	no	up	4.9E-05	yes
ENSRNOT00000040830 : Pds5b : D3ZU56	0.26	yes	down	2.1E-04	yes	chr12	promoter	Intermediate	LCP	1.20	no	up	0.15	no	0.96	no	down	4.7E-06	yes
MSTRG.16794.3 : Dnajc11 : B1WBY5	2.59	yes	up	1.9E-14	yes	chr5	promoter	Intermediate	ICP	0.74	no	down	0.06	no	0.88	no	down	4.2E-12	yes
MSTRG.5482.1 : Ncor2 : A0A0G2JU91	2.16	yes	up	1.6E-06	yes	chr12	intron	internal intron	NA	0.77	no	down	0.49	no	1.01	no	up	7.1E-01	no
MSTRG.9728.1 : Cnot1 : G3V7M0	0.08	yes	down	2.6E-04	yes	chr19	intron	internal intron	NA	2.43	yes	up	0.26	no	1.01	no	up	1.2E-01	no
MSTRG.19258.1 : Lima1 : F1LR10	0.35	yes	down	4.4E-04	yes	chr7	intron	internal intron	NA	1.00	no	down	1.00	no	1.16	no	up	2.2E-10	yes
MSTRG.1545.2 : Sbf2 : B5DEJ9	4.77	yes	up	8.9E-06	yes	chr1	intron	internal intron	NA	0.44	yes	down	0.31	no	1.03	no	up	9.5E-02	no
MSTRG.20927.2 : Aox1 : F1LRQ1	0.36	yes	down	1.5E-07	yes	chr9	intron	internal intron	NA	0.98	no	down	0.92	no	0.73	yes	down	3.5E-17	yes
MSTRG.19524.1 : Jam3 : Q68FQ2	0.30	yes	down	4.0E-04	yes	chr8	exon	last exon	NA	1.19	no	up	0.09	no	0.97	no	down	8.5E-02	no
ENSRNOT00000068493 : Dlg4 : P31016	2.15	yes	up	8.6E-07	yes	chr10	intron	last intron	NA	0.79	no	down	0.31	no	0.97	no	down	9.1E-02	no
MSTRG.17422.2 : Ppm1a : P20650	2.35	yes	up	3.9E-04	yes	chr6	intron	last intron	NA	0.44	yes	down	0.45	no	0.96	no	down	5.3E-04	yes
ENSRNOT00000080304 : Mark3 : F1M836	0.30	yes	down	2.6E-04	yes	chr6	exon	last exon	NA	0.99	no	down	0.57	no	1.10	no	up	4.1E-07	yes
MSTRG.10871.2 : Dcun1d1 : D3ZRV0	1.60	yes	up	3.7E-04	yes	chr2	exon	internal exon	NA	0.66	no	down	0.46	no	0.97	no	down	2.6E-02	yes
ENSRNOT00000085545 : Fmnl2 : A0A0G2K132	0.84	no	down	6.1E-07	yes	chr3	intron	internal intron	NA	1.05	no	up	0.78	no	1.00	no	down	9.6E-01	no
MSTRG.18268.3 : Akap8 : Q63014	0.08	yes	down	2.7E-04	yes	chr7	intron	internal intron	NA	1.08	no	up	0.42	no	0.99	no	down	2.8E-01	no
ENSRNOT00000085158 : Aldh1a3 : A9EEP5	0.08	yes	down	2.9E-04	yes	chr1	promoter	Distal	ICP	0.96	no	down	0.94	no	0.84	no	down	1.5E-18	yes
MSTRG.16327.4 : Macf1 : A0A0G2K9T4	0.36	yes	down	1.3E-05	yes	chr5	exon	last exon	NA	1.10	no	up	0.90	no	0.97	no	down	9.0E-11	yes
MSTRG.10589.3 : Itga1 : P18614	0.09	yes	down	9.6E-08	yes	chr2	intron	internal intron	NA	1.34	no	up	0.70	no	0.81	yes	down	3.6E-17	yes
MSTRG.4117.4 : Nol11 : A0A0G2K1A9	0.21	yes	down	9.6E-05	yes	chr10	intron	internal intron	NA	0.91	no	down	0.71	no	1.06	no	up	1.9E-03	yes
MSTRG.3783.1 : Acsf2 : Q499N5	0.31	yes	down	5.4E-07	yes	chr10	intron	internal intron	NA	1.12	no	up	0.56	no	0.87	no	down	2.4E-13	yes
MSTRG.2839.1 : Srrm2 : A0A0G2K2M9	1.72	yes	up	9.0E-05	yes	chr10	exon	internal exon	NA	0.92	no	down	0.96	no	1.00	no	up	9.0E-01	no
ENSRNOT00000064840 : Atp2a3 : G3V9U7	inf	yes	up	2.1E-05	yes	chr10	intron	internal intron	NA	0.77	no	down	0.62	no	0.77	yes	down	1.7E-16	yes
MSTRG.15068.5 : Washc2c : F1LPG9	0.05	yes	down	2.7E-19	yes	chr4	exon	internal exon	NA	2.24	yes	up	0.11	no	1.05	no	up	2.1E-05	yes
MSTRG.3781.2 : Luc7l3 : D3ZFB2	0.31	yes	down	4.3E-04	yes	chr10	intron	internal intron	NA	0.94	no	down	0.50	no	0.98	no	down	9.6E-04	yes
ENSRNOT00000089847 : Dkc1 : P40615	0.28	yes	down	2.4E-05	yes	chrX	intron	internal intron	NA	0.81	no	down	0.21	no	0.99	no	down	3.6E-01	no
ENSRNOT00000079196 : Ensa : P60841	2.35	yes	up	1.3E-05	yes	chr2	exon	last exon	NA	1.14	no	up	0.13	no	1.04	no	up	5.7E-02	no
MSTRG.11265.31 : Ubap2l : E9PTR4	6.62	yes	up	4.0E-04	yes	chr2	intron	internal intron	NA	0.94	no	down	0.83	no	1.13	no	up	3.8E-12	yes
ENSRNOT00000041234 : Aplf : F6Q5G6	0.12	yes	down	8.1E-06	yes	chr4	exon	last exon	NA	1.13	no	up	0.58	no	0.99	no	down	2.8E-01	no
ENSRNOT00000011490 : Xpc : D4A3D8	20.37	yes	up	1.0E-05	yes	chr4	exon	internal exon	NA	1.02	no	up	0.77	no	0.95	no	down	3.2E-02	yes
ENSRNOT00000093066 : Clasp1 : F1LNR1	0.36	yes	down	6.7E-07	yes	chr13	intron	internal intron	NA	1.31	no	up	0.38	no	1.00	no	down	7.0E-01	no
MSTRG.3351.2 : Polr2a : D4A5A6	2.68	yes	up	1.4E-05	yes	chr10	exon	internal exon	NA	0.71	no	down	0.37	no	1.00	no	down	6.0E-01	no
ENSRNOT00000066071 : Aff1 : D3ZBU5	1.49	yes	up	9.8E-06	yes	chr14	intron	internal intron	NA	0.99	no	down	0.54	no	1.07	no	up	4.0E-02	yes
MSTRG.6635.2 : Rufy3 : A0A0G2K6A9	2.37	yes	up	3.9E-04	yes	chr14	promoter	Distal	LCP	1.44	no	up	0.13	no	1.11	no	up	1.3E-05	yes
MSTRG.19472.4 : Dnm2 : A0A0A0MY48	0.11	yes	down	8.9E-11	yes	chr8	intron	first intron	NA	1.00	no	down	0.99	no	1.05	no	up	2.0E-08	yes
MSTRG.18268.1 : Akap8 : Q63014	0.08	yes	down	2.7E-04	yes	chr7	intron	internal intron	NA	1.02	no	up	0.92	no	0.99	no	down	2.8E-01	no
MSTRG.20920.1 : Maip1 : Q6AY04	27.81	yes	up	1.4E-21	yes	chr9	promoter	Intermediate	ICP	0.50	yes	down	0.24	no	0.87	no	down	5.5E-04	yes
MSTRG.162.2 : Tpd52l1 : Q499Q2	2.69	yes	up	6.5E-28	yes	chr1	promoter	Distal	LCP	1.59	no	up	0.37	no	0.97	no	down	5.0E-01	no
MSTRG.21040.1 : Tns1 : F1LN42	0.09	yes	down	2.0E-04	yes	chr9	intron	last intron	NA	0.93	no	down	0.94	no	0.86	no	down	7.8E-19	yes
MSTRG.862.1 : Lgi4 : Q6P2A4	inf	yes	up	6.8E-05	yes	chr1	promoter	Proximal	LCP	0.97	no	down	0.83	no	0.77	yes	down	1.0E-07	yes
MSTRG.19472.2 : Dnm2 : A0A0A0MY48	0.11	yes	down	8.9E-11	yes	chr8	intron	first intron	NA	1.05	no	up	0.81	no	1.05	no	up	2.0E-08	yes
MSTRG.15948.3 : Cdk5rap2 : F1M4B7	0	yes	down	1.4E-04	yes	chr5	intron	internal intron	NA	1.11	no	up	0.52	no	0.98	no	down	2.6E-01	no
ENSRNOT00000019640 : Lcmt1 : G3V7V9	0.40	yes	down	4.3E-07	yes	chr1	promoter	Distal	LCP	0.99	no	down	0.94	no	0.98	no	down	7.9E-02	no
MSTRG.17804.2 : Cdc42bpb : A0A0G2KB58	0.39	yes	down	3.8E-04	yes	chr6	intron	internal intron	NA	1.09	no	up	0.76	no	0.97	no	down	3.6E-07	yes
MSTRG.21039.4 : Tns1 : F1LN42	0.09	yes	down	2.0E-04	yes	chr9	intron	last intron	NA	0.48	yes	down	0.51	no	0.86	no	down	7.8E-19	yes
MSTRG.13195.6 : Arhgap1 : D4A6C5	3.73	yes	up	1.8E-11	yes	chr3	intron	internal intron	NA	1.58	no	up	0.44	no	1.04	no	up	1.7E-04	yes
ENSRNOT00000026057 : Acsl4 : O35547	0.19	yes	down	1.2E-04	yes	chrX	intron	internal intron	NA	1.40	no	up	0.46	no	1.22	yes	up	2.0E-10	yes
MSTRG.1443.15 : Arap1 : F1LM60	2.71	yes	up	3.3E-04	yes	chr1	intron	first intron	NA	1.11	no	up	0.78	no	0.99	no	down	2.5E-02	yes
MSTRG.12912.2 : Scai : F1M3P6	0.24	yes	down	1.2E-04	yes	chr3	intron	internal intron	NA	0.99	no	down	0.77	no	0.86	no	down	1.0E-11	yes
MSTRG.13654.1 : Xrn2 : D4A914	0.32	yes	down	3.4E-05	yes	chr3	intron	internal intron	NA	0.78	no	down	0.39	no	1.03	no	up	8.8E-06	yes
MSTRG.20878.1 : Gls : P13264	0.05	yes	down	1.5E-13	yes	chr9	intron	first intron	NA	2.11	yes	up	0.05	yes	1.08	no	up	4.1E-02	yes
ENSRNOT00000060452 : Prim2 : Q4V8C0	4.01	yes	up	2.8E-05	yes	chr9	intron	internal intron	NA	1.08	no	up	0.09	no	1.03	no	up	2.8E-01	no
MSTRG.2596.5 : Gbf1 : A0A0G2K3N1	0.28	yes	down	1.1E-05	yes	chr1	intron	internal intron	NA	1.29	no	up	0.09	no	1.05	no	up	6.1E-10	yes
MSTRG.332.4 : Fgfr1op : Q4V7C1	7.97	yes	up	2.8E-17	yes	chr1	intron	internal intron	NA	0.79	no	down	0.50	no	1.07	no	up	7.3E-04	yes
ENSRNOT00000093297 : Mycbp2 : D4A2D3	0.31	yes	down	9.2E-06	yes	chr15	intron	internal intron	NA	1.09	no	up	0.59	no	1.00	no	up	6.8E-01	no
ENSRNOT00000091840 : Plec : Q6S395	0.33	yes	down	4.2E-06	yes	chr7	exon	last exon	NA	1.01	no	up	0.04	yes	1.03	no	up	2.2E-01	no
ENSRNOT00000025550 : Rbm17 : Q6AY02	inf	yes	up	1.9E-04	yes	chr17	intron	internal intron	NA	1.09	no	up	0.22	no	1.01	no	up	4.0E-01	no
ENSRNOT00000072511 : Mmab : M0R959	0.58	yes	down	6.7E-04	yes	chr12	intron	last intron	NA	1.06	no	up	0.71	no	0.97	no	down	1.6E-01	no
ENSRNOT00000002293 : Arl6 : B1WC73	5.24	yes	up	2.5E-08	yes	chr11	exon	first exon	NA	0.95	no	down	0.67	no	0.98	no	down	1.6E-01	no
MSTRG.9206.2 : Ss18 : Q5XI66	6.27	yes	up	1.6E-10	yes	chr18	exon	internal exon	NA	0.97	no	down	0.91	no	1.04	no	up	6.9E-02	no
MSTRG.10871.3 : Dcun1d1 : D3ZRV0	1.60	yes	up	3.7E-04	yes	chr2	exon	internal exon	NA	1.43	no	up	0.56	no	0.97	no	down	2.6E-02	yes
ENSRNOT00000092712 : Pafah1b1 : P63004	0.19	yes	down	5.9E-04	yes	chr10	intron	internal intron	NA	1.67	no	up	0.40	no	0.96	no	down	1.4E-04	yes
ENSRNOT00000076269 : Slfn13 : A0A096MKD0	2.27	yes	up	1.5E-04	yes	chr10	promoter	Intermediate	LCP	1.00	no	down	0.04	yes	0.97	no	down	2.7E-02	yes
MSTRG.21144.1 : Cab39 : A0A0G2JZH0	39.86	yes	up	1.2E-27	yes	chr9	promoter	Intermediate	LCP	0.90	no	down	0.40	no	0.99	no	down	5.6E-01	no
MSTRG.2021.2 : Unc93b1 : D3ZDJ4	3.27	yes	up	6.0E-05	yes	chr1	intron	internal intron	NA	1.73	no	up	0.07	no	1.19	no	up	9.2E-05	yes
ENSRNOT00000047527 : RT1-CE7 : D3ZLE6	inf	yes	up	7.3E-07	yes	chr20	exon	internal exon	NA	0.75	no	down	0.73	no	1.22	yes	up	7.3E-16	yes
MSTRG.21039.14 : Tns1 : F1LN42	0.09	yes	down	2.0E-04	yes	chr9	intron	last intron	NA	0.74	no	down	0.16	no	0.86	no	down	7.8E-19	yes
ENSRNOT00000093649 : Tor1a : Q68G38	3.04	yes	up	5.0E-05	yes	chr3	exon	last exon	NA	1.35	no	up	0.00	yes	1.02	no	up	1.6E-01	no
ENSRNOT00000026134 : Rnf185 : Q568Y3	7.04	yes	up	1.7E-09	yes	chr14	intron	internal intron	NA	0.75	no	down	0.61	no	0.86	no	down	3.3E-04	yes
MSTRG.21600.2 : Acot9 : Q5U2X8	0.09	yes	down	5.0E-06	yes	chrX	promoter	Distal	LCP	0.99	no	down	0.98	no	0.99	no	down	5.1E-02	no
MSTRG.11265.5 : Ubap2l : E9PTR4	6.62	yes	up	4.0E-04	yes	chr2	intron	internal intron	NA	1.18	no	up	0.60	no	1.13	no	up	3.8E-12	yes
MSTRG.97.9 : Bclaf1 : B1WC16	0.67	yes	down	1.3E-07	yes	chr1	intron	internal intron	NA	1.24	no	up	0.64	no	1.03	no	up	6.8E-02	no
ENSRNOT00000013238 : Acadm : G3V796	0.46	yes	down	4.0E-04	yes	chr2	intron	internal intron	NA	1.19	no	up	0.41	no	1.00	no	down	9.4E-01	no
MSTRG.5417.9 : Auts2 : F1M388	0.04	yes	down	4.8E-14	yes	chr12	intron	internal intron	NA	1.08	no	up	0.82	no	1.01	no	up	5.6E-01	no

MSTRG.19257.2 : Lima1 : F1LR10	0.35	yes	down	4.4E-04	yes	chr7	intron	internal intron	NA	3.53	yes	up	0.09	no	1.16	no	up	2.2E-10	yes
ENSRNOT00000089743 : Fmr1 : Q80WE1	inf	yes	up	3.0E-04	yes	chrX	intron	internal intron	NA	0.77	no	down	0.14	no	1.09	no	up	7.3E-06	yes
ENSRNOT00000058206 : Tsc22d1 : P62501	3.00	yes	up	3.0E-04	yes	chr15	promoter	Intermediate	ICP	0.78	no	down	0.81	no	0.77	yes	down	5.9E-09	yes
MSTRG.7470.9 : Acin1 : E9PST5	0.30	yes	down	1.1E-04	yes	chr15	promoter	Distal	LCP	0.71	no	down	0.04	yes	0.98	no	down	5.1E-04	yes
MSTRG.20817.4 : Map4k4 : A0A0G2K7W4	0.36	yes	down	7.1E-05	yes	chr9	intron	internal intron	NA	1.14	no	up	0.79	no	1.10	no	up	7.1E-06	yes
MSTRG.19188.1 : Rpap3 : Q68FQ7	0.29	yes	down	4.9E-05	yes	chr7	intron	internal intron	NA	1.06	no	up	0.76	no	1.04	no	up	2.9E-04	yes
ENSRNOT00000006880 : Itga4 : D3ZMQ3	0.24	yes	down	2.1E-07	yes	chr3	intron	internal intron	NA	1.79	no	up	0.09	no	0.99	no	down	7.5E-03	yes
ENSRNOT00000076255 : Lsm14a : A0A0G2JUK2	0.31	yes	down	3.8E-04	yes	chr1	intron	last intron	NA	0.99	no	down	0.99	no	1.02	no	up	2.8E-01	no
MSTRG.7056.1 : Myo1g : A0A0G2K6E3	0.66	yes	down	3.2E-12	yes	chr14	intron	internal intron	NA	1.41	no	up	0.10	no	1.04	no	up	3.4E-02	yes
MSTRG.12240.6 : Bicc1 : A0A0G2K0Y0	5.19	yes	up	5.0E-04	yes	chr20	intron	internal intron	NA	0.79	no	down	0.38	no	1.52	yes	up	1.2E-05	yes
MSTRG.8511.3 : Dapk1 : F1LNN8	0.35	yes	down	1.5E-04	yes	chr17	intron	internal intron	NA	1.19	no	up	0.47	no	0.96	no	down	1.0E-01	no
MSTRG.19538.16 : Nfrkb : D4A421	0.58	yes	down	1.3E-05	yes	chr8	intron	internal intron	NA	0.64	no	down	0.32	no	0.89	no	down	2.5E-03	yes
MSTRG.19306.1 : Krt8 : Q10758	0.19	yes	down	1.4E-04	yes	chr7	exon	last exon	NA	0.95	no	down	0.55	no	0.83	yes	down	4.5E-14	yes
MSTRG.17785.5 : Dync1h1 : M0R9X8	0.19	yes	down	1.5E-06	yes	chr6	intron	internal intron	NA	2.08	yes	up	0.48	no	0.99	no	down	3.4E-03	yes
MSTRG.15720.4 : Pigo : D3ZTP8	0.61	yes	down	2.7E-05	yes	chr5	exon	internal exon	NA	0.71	no	down	0.44	no	0.80	yes	down	9.0E-03	yes
MSTRG.10916.3 : Naa15 : D3ZD89	0.53	yes	down	3.9E-04	yes	chr2	exon	last exon	NA	1.18	no	up	0.27	no	1.05	no	up	5.6E-06	yes
MSTRG.2579.6 : Sfxn3 : Q9JHY2	0.39	yes	down	7.8E-05	yes	chr1	intron	internal intron	NA	0.62	no	down	0.36	no	0.98	no	down	7.5E-03	yes
ENSRNOT00000077406 : Podxl : A0A0G2K2L1	0.31	yes	down	1.5E-14	yes	chr4	intron	internal intron	NA	1.05	no	up	0.85	no	0.91	no	down	6.5E-07	yes
MSTRG.18880.4 : Plec : Q6S395	0.33	yes	down	4.2E-06	yes	chr7	exon	last exon	NA	1.31	no	up	0.08	no	1.03	no	up	2.2E-01	no
MSTRG.549.3 : Zc3h4 : D3ZVW3	2.19	yes	up	2.6E-04	yes	chr1	intron	internal intron	NA	0.86	no	down	0.78	no	0.98	no	down	3.2E-01	no
ENSRNOT00000016863 : Ppwd1 : D3ZVP6	0.23	yes	down	1.8E-04	yes	chr2	intron	internal intron	NA	1.18	no	up	0.18	no	1.06	no	up	3.7E-03	yes
ENSRNOT00000076676 : Parp4 : A0A096MJ98	1.23	yes	up	1.1E-06	yes	chr15	intron	last intron	NA	1.05	no	up	0.37	no	1.00	no	down	9.9E-01	no
MSTRG.14995.9 : Itpr1 : A0A0A0MY31	3.16	yes	up	1.1E-05	yes	chr4	intron	internal intron	NA	1.74	no	up	0.23	no	0.87	no	down	1.1E-14	yes
ENSRNOT00000076204 : Asph : A0A096MKE0	2.56	yes	up	9.8E-06	yes	chr5	intron	internal intron	NA	0.95	no	down	0.50	no	0.99	no	down	1.3E-01	no
ENSRNOT00000025758 : Slc25a17 : B2GUY8	3.05	yes	up	2.3E-04	yes	chr7	intron	internal intron	NA	1.34	no	up	0.21	no	0.95	no	down	3.0E-02	yes
ENSRNOT00000011049 : Slc9a1 : Q8R4H8	0.61	yes	down	7.2E-05	yes	chr5	intron	internal intron	NA	0.65	no	down	0.11	no	1.05	no	up	2.8E-01	no
MSTRG.3481.6 : Tsr1 : D3ZEM8	45.59	yes	up	3.4E-04	yes	chr10	intron	internal intron	NA	1.15	no	up	0.40	no	1.00	no	down	9.7E-01	no
ENSRNOT00000068011 : Rbpms : F2Z3S5	0.02	yes	down	2.6E-05	yes	chr16	promoter	Proximal	ICP	1.04	no	up	0.78	no	1.06	no	up	4.9E-05	yes
MSTRG.4725.14 : Kalrn : P97924	1.54	yes	up	9.1E-07	yes	chr11	exon	last exon	NA	1.08	no	up	0.70	no	1.04	no	up	1.6E-04	yes
MSTRG.16755.11 : Kif1b : A0A0G2KA12	3.65	yes	up	7.4E-05	yes	chr5	intron	internal intron	NA	0.69	no	down	0.42	no	1.05	no	up	6.5E-02	no
MSTRG.9198.2 : Osbpl1a : A0A0G2K327	0.28	yes	down	2.2E-05	yes	chr18	intron	internal intron	NA	1.23	no	up	0.03	yes	0.94	no	down	2.8E-02	yes
MSTRG.1675.1 : Arhgap17 : D4AAV2	0.83	yes	down	2.6E-07	yes	chr1	intron	internal intron	NA	0.83	no	down	0.73	no	1.01	no	up	3.1E-01	no
ENSRNOT00000050511 : Tst : P24329	0.37	yes	down	4.2E-05	yes	chr7	intron	last intron	NA	0.47	yes	down	0.56	no	0.77	yes	down	1.8E-17	yes
ENSRNOT00000080750 : Dapk1 : F1LNN8	0.35	yes	down	1.5E-04	yes	chr17	intron	internal intron	NA	0.91	no	down	0.52	no	0.96	no	down	1.0E-01	no
ENSRNOT00000076099 : Gigyf2 : A0A096MKC0	0.59	yes	down	1.9E-05	yes	chr9	intron	internal intron	NA	0.83	no	down	0.06	no	1.01	no	up	7.0E-01	no
MSTRG.5417.10 : Auts2 : F1M388	0.04	yes	down	4.8E-14	yes	chr12	intron	internal intron	NA	1.43	no	up	0.48	no	1.01	no	up	5.6E-01	no
MSTRG.16606.9 : Alpl : P08289	0.52	yes	down	2.4E-04	yes	chr5	promoter	Intermediate	LCP	1.00	no	down	0.47	no	1.00	no	down	6.2E-01	no
MSTRG.13466.4 : Myef2 : A0A0G2K402	2.63	yes	up	2.7E-04	yes	chr3	promoter	Intermediate	LCP	1.47	no	up	0.16	no	0.93	no	down	4.5E-09	yes
ENSRNOT00000092125 : Anpep : G3V7W7	0.25	yes	down	1.9E-09	yes	chr1	intron	internal intron	NA	1.33	no	up	0.34	no	1.05	no	up	3.0E-08	yes
MSTRG.17857.2 : Crip1 : P63255	8.66	yes	up	6.1E-05	yes	chr6	intron	first intron	NA	0.07	yes	down	0.16	no	1.20	yes	up	1.6E-08	yes
MSTRG.17520.9 : Srsf5 : Q09167	0.10	yes	down	3.0E-09	yes	chr6	promoter	Proximal	ICP	1.66	no	up	0.00	yes	1.03	no	up	4.2E-02	yes
ENSRNOT00000011054 : Bsdc1 : D4A3M7	0	yes	down	2.4E-04	yes	chr5	intron	last intron	NA	0.99	no	down	0.97	no	0.92	no	down	4.2E-04	yes
ENSRNOT00000032569 : Mb21d2 : D4ACS3	2.29	yes	up	3.8E-07	yes	chr11	exon	last exon	NA	0.47	yes	down	0.08	no	0.98	no	down	6.7E-02	no
MSTRG.15855.8 : RGD1306148 : F1M446	0.22	yes	down	4.0E-05	yes	chr5	intron	internal intron	NA	1.09	no	up	0.46	no	1.01	no	up	7.4E-02	no
MSTRG.6746.1 : Pds5a : A4L9P7	1.26	yes	up	1.3E-13	yes	chr14	exon	last exon	NA	0.88	no	down	0.83	no	1.01	no	up	2.7E-01	no
MSTRG.4508.2 : Dyrk1a : Q63470	0.26	yes	down	2.2E-05	yes	chr11	intron	internal intron	NA	1.83	no	up	0.09	no	1.02	no	up	1.6E-01	no
ENSRNOT00000032780 : Eln : A0A0G2JST5	3.55	yes	up	3.0E-04	yes	chr12	intron	internal intron	NA	0.89	no	down	0.81	no	0.91	no	down	1.2E-01	no
MSTRG.12973.11 : Rbms1 : A0A0G2K4R7	0	yes	down	3.3E-05	yes	chr3	intron	internal intron	NA	1.22	no	up	0.17	no	0.97	no	down	1.2E-01	no
MSTRG.20350.1 : Clasp2 : A0A0G2JZM8	0.25	yes	down	1.3E-05	yes	chr8	intron	internal intron	NA	1.30	no	up	0.71	no	0.98	no	down	1.8E-01	no
MSTRG.9568.3 : Nedd4l : F1LRN8	2.03	yes	up	3.6E-05	yes	chr18	intron	internal intron	NA	1.32	no	up	0.66	no	0.95	no	down	3.9E-02	yes
MSTRG.7804.3 : Mycbp2 : A0A1W2Q6I3	0.31	yes	down	9.2E-06	yes	chr15	intron	internal intron	NA	0.66	no	down	0.10	no	1.00	no	up	8.8E-01	no
MSTRG.12646.2 : Rapgef1 : F1M8L9	inf	yes	up	4.7E-05	yes	chr3	intron	internal intron	NA	0.46	yes	down	0.30	no	0.88	no	down	4.1E-04	yes
ENSRNOT00000067000 : Man1a2 : D3ZR49	1.09	no	up	3.3E-05	yes	chr2	exon	last exon	NA	1.28	no	up	0.05	yes	1.05	no	up	1.6E-05	yes
MSTRG.3421.3 : Nup88 : O08658	0.25	yes	down	1.2E-04	yes	chr10	intron	internal intron	NA	2.12	yes	up	0.23	no	0.95	no	down	3.4E-05	yes
MSTRG.9598.5 : Cep192 : D4A3X0	4.22	yes	up	1.3E-12	yes	chr18	intron	internal intron	NA	0.93	no	down	0.53	no	0.95	no	down	9.1E-04	yes
MSTRG.16327.5 : Macf1 : A0A0G2K9T4	0.36	yes	down	1.3E-05	yes	chr5	exon	last exon	NA	1.48	no	up	0.63	no	0.97	no	down	9.0E-11	yes
MSTRG.2527.1 : Pi4k2a : Q99M64	0.48	yes	down	2.9E-06	yes	chr1	intron	internal intron	NA	1.14	no	up	0.53	no	1.02	no	up	1.6E-01	no
MSTRG.13195.5 : Arhgap1 : D4A6C5	3.73	yes	up	1.8E-11	yes	chr3	intron	internal intron	NA	0.78	no	down	0.71	no	1.04	no	up	1.7E-04	yes
MSTRG.7804.3 : Mycbp2 : D4A2D3	0.31	yes	down	9.2E-06	yes	chr15	intron	internal intron	NA	0.66	no	down	0.10	no	1.00	no	up	6.8E-01	no
MSTRG.9889.7 : Nf1x : F2Z3R4	0.58	yes	down	1.9E-06	yes	chr19	intron	internal intron	NA	0.77	no	down	0.45	no	0.77	yes	down	2.4E-14	yes
MSTRG.1974.2 : Nap1l4 : Q5U2Z3	0.36	yes	down	7.2E-05	yes	chr1	intron	internal intron	NA	2.54	yes	up	0.31	no	0.98	no	down	3.1E-02	yes
ENSRNOT00000075874 : Smndc1 : Q4QQU6	inf	yes	up	1.3E-04	yes	chr1	promoter	Distal	LCP	0.92	no	down	0.57	no	1.06	no	up	2.2E-04	yes
MSTRG.5050.1 : Elavl1 : B5DF91	0.21	yes	down	5.3E-09	yes	chr12	intron	internal intron	NA	1.41	no	up	0.01	yes	1.05	no	up	2.2E-07	yes
MSTRG.11953.44 : RT1-CE7 : D3ZLE6	inf	yes	up	7.3E-07	yes	chr20	exon	internal exon	NA	1.82	no	up	0.43	no	1.22	yes	up	7.3E-16	yes
MSTRG.5958.4 : Csrp1 : P47875	1.98	yes	up	3.9E-04	yes	chr13	promoter	Intermediate	LCP	0.26	yes	down	0.30	no	1.11	no	up	3.1E-08	yes
ENSRNOT00000086959 : Rap2a : A0A0G2JTW1	4.31	yes	up	1.6E-04	yes	chr15	intron	last intron	NA	1.03	no	up	0.73	no	0.76	yes	down	2.5E-10	yes
MSTRG.7500.4 : Tm9sf1 : Q66HF2	5.15	yes	up	5.3E-11	yes	chr15	intron	last intron	NA	1.38	no	up	0.30	no	1.02	no	up	4.7E-02	yes
MSTRG.3511.2 : Cpd : A0A0G2JY30	0.15	yes	down	4.3E-04	yes	chr10	intron	internal intron	NA	1.63	no	up	0.10	no	1.13	no	up	1.1E-08	yes
MSTRG.10475.1 : Tnpo1 : F1LQP9	24.87	yes	up	1.6E-05	yes	chr2	exon	internal exon	NA	1.35	no	up	0.49	no	0.98	no	down	5.8E-02	no
MSTRG.21202.4 : Lrrfip1 : Q66HF9	6.49	yes	up	4.9E-10	yes	chr9	exon	last exon	NA	0.89	no	down	0.79	no	1.15	no	up	2.1E-11	yes
ENSRNOT00000080345 : Aamp : B0K024	0.09	yes	down	2.6E-04	yes	chr9	promoter	Intermediate	LCP	1.01	no	up	0.81	no	1.06	no	up	1.5E-05	yes
MSTRG.14695.7 : Ptcd3 : D3ZGM1	3.88	yes	up	4.1E-35	yes	chr4	intron	internal intron	NA	0.34	yes	down	0.05	yes	0.98	no	down	3.1E-01	no
MSTRG.20698.1 : Tram2 : F1LUA2	3	yes	up	2.1E-05	yes	chr9	intron	first intron	NA	0.76	no	down	0.50	no	1.02	no	up	1.2E-01	no
MSTRG.8552.8 : Ddx46 : Q62780	0.56	yes	down	3.4E-04	yes	chr17	intron	internal intron	NA	1.41	no	up	0.47	no	0.98	no	down	1.3E-01	no
MSTRG.6624.8 : Ankrd17 : D4A0B4	3.39	yes	up	8.9E-05	yes	chr14	intron	internal intron	NA	1.27	no	up	0.09	no	1.11	no	up	1.6E-06	yes
MSTRG.16640.10 : Arhgef10l : M0R7W2	2.87	yes	up	9.9E-07	yes	chr5	intron	internal intron	NA	0.74	no	down	0.45	no	1.13	no	up	8.4E-04	yes
MSTRG.5482.11 : Ncor2 : A0A0G2JU91	2.16	yes	up	1.6E-06	yes	chr12	intron	internal intron	NA	0.58	no	down	0.08	no	1.01	no	up	7.1E-01	no
MSTRG.9435.9 : Tcerg1 : B5DEZ4	0.26	yes	down	2.8E-04	yes	chr18	promoter	Intermediate	ICP	1.16	no	up	0.64	no	1.02	no	up	4.4E-02	yes
ENSRNOT00000077884 : Fkbp11 : G3V7V5	inf	yes	up	4.7E-06	yes	chr7	intron	internal intron	NA	1.00	no	down	0.95	no	1.06	no	up	1.6E-07	yes
MSTRG.18876.4 : Puf60 : A0A0H2UHZ6	0.25	yes	down	1.3E-05	yes	chr7	exon	internal exon	NA	1.35	no	up	0.57	no	1.00	no	down	7.4E-02	no
MSTRG.7965.2 : Bmpr1a : Q78EA7	0.29	yes	down	2.1E-04	yes	chr16	intron	internal intron	NA	0.97	no	down	0.45	no	0.90	no	down	9.8E-10	yes
MSTRG.4699.3 : Kpna1 : P83953	32	yes	up	7.2E-05	yes	chr11	promoter	Distal	LCP	0.67	no	down	0.42	no	1.11	no	up	5.7E-08	yes
ENSRNOT00000089859 : Cys1 : D3ZCZ3	7.20	yes	up	2.2E-05	yes	chr6	exon	last exon	NA	1.05	no	up	0.91	no	0.98	no	down	4.3E-01	no
MSTRG.10153.3 : Cmip : A0A0G2JYB9	0.58	yes	down	9.9E-05	yes	chr19	intron	internal intron	NA	0.60	no	down	0.50	no	0.89	no	down	2.1E-04	yes
MSTRG.9889.9 : Nf1x : F2Z3R4	0.58	yes	down	1.9E-06	yes	chr19	intron	internal intron	NA	0.56	no	down	0.40	no	0.77	yes	down	2.4E-14	yes
MSTRG.6713.8 : Fryl : D3ZQY4	2.76	yes	up	1.7E-04	yes	chr14	intron	internal intron	NA	0.85	no	down	0.82	no	1.00	no	down	4.6E-01	no
MSTRG.1151.2 : Aldh1a3 : A9EEP5	0.08	yes	down	2.9E-04	yes	chr1	promoter	Distal	ICP	1.47	no	up	0.33	no	0.84	no	down	1.5E-18	yes
ENSRNOT00000004228 : Sdhc : Q641Z9	5.32	yes	up	1.1E-04	yes	chr13	intron	internal intron	NA	1.13	no	up	0.39	no	0.93	no	down	3.6E-02	yes
MSTRG.97.17 : Bclaf1 : B1WC16	0.67	yes	down	1.3E-07	yes	chr1	intron	internal intron	NA	0.97	no	down	0.80	no	1.03	no	up	6.8E-02	no
MSTRG.8326.1 : Hook3 : Q7TQ77	0.22	yes	down	2.1E-04	yes	chr16	intron	internal intron	NA	1.19	no	up	0.47	no	1.01	no	up	5.0E-03	yes
MSTRG.12318.1 : Dnajb12 : Q5FVC4	32.15	yes	up	1.5E-05	yes	chr20	exon	internal exon	NA	1.27	no	up	0.02	yes	1.07	no	up	2.0E-04	yes
MSTRG.3441.2 : Ankfy1 : D4A1J6	0.33	yes	down	7.9E-05	yes	chr10	intron	internal intron	NA	0.83	no	down	0.82	no	0.97	no	down	1.1E-04	yes
MSTRG.7551.1 : Xpo4 : D3ZQI6	5.75	yes	up	1.3E-06	yes	chr15	intron	internal intron	NA	0.76	no	down	0.48	no	0.88	no	down	7.2E-03	yes
ENSRNOT00000093112 : Rrm1 : Q5U2Q5	8.63	yes	up	2.6E-22	yes	chr1	intron	last intron	NA	1.00	no	down	0.96	no	1.04	no	up	2.0E-04	yes
MSTRG.14583.1 : Skap2 : Q920G0	0.26	yes	down	1.3E-08	yes	chr4	intron	internal intron	NA	1.20	no	up	0.29	no	1.23	yes	up	9.4E-12	yes
MSTRG.11953.29 : RT1-CE7 : D3ZLE6	inf	yes	up	7.3E-07	yes	chr20	exon	internal exon	NA	0.97	no	down	0.92	no	1.22	yes	up	7.3E-16	yes
ENSRNOT00000089359 : Arhgef17 : A0A0G2JXT9	0.45	yes	down	6.0E-05	yes	chr1	exon	last exon	NA	0.93	no	down	0.72	no	0.93	no	down	3.1E-10	yes
ENSRNOT00000083005 : Map1a : P34926	3.41	yes	up	1.8E-05	yes	chr3	intron	last intron	NA	0.96	no	down	0.55	no	1.03	no	up	8.9E-11	yes
MSTRG.8713.5 : Sirt5 : Q68FX9	6.16	yes	up	1.6E-04	yes	chr17	intron	internal intron	NA	1.98	no	up	0.38	no	1.01	no	up	5.1E-01	no
ENSRNOT00000078279 : Lipa : Q64194	35.26	yes	up	5.1E-18	yes	chr1	intron	internal intron	NA	1.07	no	up	0.82	no	1.02	no	up	6.9E-02	no

MSTRG.19777.8 : Sin3a : A0A0G2K3H5	0.53	yes	down	4.1E-06	yes	chr8	intron	last intron	NA	1.25	no	up	0.48	no	0.96	no	down	6.2E-05	yes
ENSRNOT00000072628 : Nup214 : M0RBV9	0.13	yes	down	4.7E-04	yes	chr3	intron	internal intron	NA	0.98	no	down	0.86	no	1.01	no	up	5.4E-01	no
ENSRNOT00000024947 : Tubb6 : Q4QQV0	0.26	yes	down	2.6E-06	yes	chr18	intron	last intron	NA	1.10	no	up	0.27	no	1.03	no	up	1.0E-02	yes
MSTRG.20350.4 : Clasp2 : A0A0G2JZM8	0.25	yes	down	1.3E-05	yes	chr8	intron	internal intron	NA	0.96	no	down	0.40	no	0.98	no	down	1.8E-01	no
ENSRNOT00000023567 : Tpm3 : A0A140TAF0	0.37	yes	down	8.0E-05	yes	chr2	intron	internal intron	NA	1.07	no	up	0.64	no	1.03	no	up	4.4E-04	yes
MSTRG.8278.45 : Rbpms : F2Z3S5	0.02	yes	down	2.6E-05	yes	chr16	promoter	Proximal	ICP	0.92	no	down	0.73	no	1.06	no	up	4.9E-05	yes
MSTRG.11167.1 : Iqgap3 : D3ZCS4	6.61	yes	up	2.3E-04	yes	chr2	intron	internal intron	NA	1.06	no	up	0.84	no	1.06	no	up	2.0E-01	no
MSTRG.21046.1 : Aamp : B0K024	0.09	yes	down	2.6E-04	yes	chr9	promoter	Intermediate	LCP	0.96	no	down	0.53	no	1.06	no	up	1.5E-05	yes
MSTRG.18268.18 : Akap8 : Q63014	0.08	yes	down	2.7E-04	yes	chr7	intron	internal intron	NA	1.12	no	up	0.29	no	0.99	no	down	2.8E-01	no
ENSRNOT00000087723 : Atp2a3 : G3V9U7	inf	yes	up	2.1E-05	yes	chr10	intron	internal intron	NA	0.83	no	down	0.48	no	0.77	yes	down	1.7E-16	yes
MSTRG.8345.2 : Fgfr1 : F1LM54	0.22	yes	down	3.0E-05	yes	chr16	exon	last exon	NA	1.07	no	up	0.85	no	1.05	no	up	1.3E-02	yes
ENSRNOT00000018259 : Dhfr : Q920D2	4.07	yes	up	3.2E-04	yes	chr2	exon	last exon	NA	0.94	no	down	0.72	no	0.91	no	down	9.0E-10	yes
MSTRG.6191.7 : Ddr2 : B1WC09	0.49	yes	down	3.3E-05	yes	chr13	exon	internal exon	NA	0.88	no	down	0.70	no	0.95	no	down	3.2E-01	no
MSTRG.7229.3 : Usp54 : Q6IE24	0.36	yes	down	1.9E-04	yes	chr15	intron	internal intron	NA	0.62	no	down	0.48	no	0.83	yes	down	7.0E-09	yes
MSTRG.5280.7 : Taf6 : Q498R0	0.74	yes	down	2.2E-06	yes	chr12	intron	internal intron	NA	1.46	no	up	0.18	no	0.91	no	down	1.8E-03	yes
ENSRNOT00000001566 : Pknox1 : Q5BJP1	0.26	yes	down	3.4E-07	yes	chr20	promoter	Proximal	LCP	1.45	no	up	0.03	yes	0.89	no	down	7.5E-04	yes
MSTRG.97.6 : Bclaf1 : B1WC16	0.67	yes	down	1.3E-07	yes	chr1	intron	internal intron	NA	0.59	no	down	0.05	yes	1.03	no	up	6.8E-02	no
MSTRG.2665.4 : Acsl5 : O88813	6.59	yes	up	1.3E-04	yes	chr1	intron	last intron	NA	1.03	no	up	0.58	no	1.08	no	up	2.2E-08	yes
MSTRG.1158.3 : Mef2a : A0A0G2JSZ4	0.04	yes	down	2.4E-04	yes	chr1	exon	last exon	NA	0.61	no	down	0.27	no	0.78	yes	down	4.1E-07	yes
ENSRNOT00000022095 : Clu : G3V836	0.38	yes	down	6.6E-05	yes	chr15	intron	first intron	NA	0.82	no	down	0.43	no	1.26	yes	up	5.8E-16	yes
ENSRNOT00000016541 : Ptx3 : D3ZT94	0.07	yes	down	2.2E-04	yes	chr2	intron	last intron	NA	1.80	no	up	0.26	no	1.08	no	up	3.1E-07	yes
ENSRNOT00000064613 : Tln2 : D3ZA84	3.91	yes	up	1.0E-11	yes	chr8	intron	internal intron	NA	1.11	no	up	0.52	no	0.87	no	down	7.8E-17	yes
ENSRNOT00000016991 : Podxl : A0A0G2K2L1	0.31	yes	down	1.5E-14	yes	chr4	intron	internal intron	NA	0.67	no	down	0.07	no	0.91	no	down	6.5E-07	yes
ENSRNOT00000009779 : Nbas : F1M0U5	5.28	yes	up	7.6E-38	yes	chr6	intron	internal intron	NA	1.14	no	up	0.01	yes	1.02	no	up	1.1E-03	yes
ENSRNOT00000084700 : Golt1b : B0BNB0	0.74	yes	down	1.2E-04	yes	chr4	intron	first intron	NA	0.62	no	down	0.50	no	0.99	no	down	5.4E-01	no
ENSRNOT00000091084 : Pitpnm2 : A0A0G2JW50	2.70	yes	up	3.9E-04	yes	chr12	promoter	Distal	LCP	1.08	no	up	0.64	no	0.89	no	down	1.9E-08	yes
MSTRG.8713.1 : Sirt5 : Q68FX9	6.16	yes	up	1.6E-04	yes	chr17	intron	internal intron	NA	0.95	no	down	0.90	no	1.01	no	up	5.1E-01	no
MSTRG.2839.3 : Srrm2 : A0A0G2K2M9	1.72	yes	up	9.0E-05	yes	chr10	exon	internal exon	NA	0.99	no	down	1.00	no	1.00	no	up	9.0E-01	no
ENSRNOT00000046201 : Mt-atp8 : P11608	1.03	no	up	5.1E-02	no	chrMT	promoter	Distal	ICP	0.44	yes	down	0.36	no	0.92	no	down	1.1E-09	yes
MSTRG.8772.2 : Serpinb6b : Q68FX2	5.33	yes	up	5.8E-08	yes	chr17	promoter	Distal	LCP	1.25	no	up	0.35	no	0.95	no	down	6.3E-06	yes
MSTRG.18046.4 : Pan2 : R9PXX6	2.14	yes	up	4.2E-04	yes	chr7	exon	internal exon	NA	1.78	no	up	0.14	no	0.84	no	down	1.2E-03	yes
MSTRG.10153.8 : Cmip : A0A0G2JYB9	0.58	yes	down	9.9E-05	yes	chr19	intron	internal intron	NA	0.71	no	down	0.31	no	0.89	no	down	2.1E-04	yes
MSTRG.4030.5 : Eftud2 : F1LM66	4.66	yes	up	6.0E-04	yes	chr10	exon	internal exon	NA	1.02	no	up	0.28	no	1.02	no	up	4.9E-02	yes
MSTRG.6707.1 : Spata18 : Q6AYL6	1.61	yes	up	6.3E-07	yes	chr14	intron	internal intron	NA	0.32	yes	down	0.22	no	0.76	yes	down	1.3E-15	yes
ENSRNOT00000067486 : Fat1 : G3V9W9	0.12	yes	down	1.5E-10	yes	chr16	exon	last exon	NA	1.13	no	up	0.78	no	1.12	no	up	2.9E-12	yes
ENSRNOT00000092613 : Washc2c : F1LPG9	0.05	yes	down	2.7E-19	yes	chr4	exon	internal exon	NA	0.94	no	down	0.32	no	1.05	no	up	2.1E-05	yes
ENSRNOT00000088945 : Plec : Q6S3A0	0.33	yes	down	4.2E-06	yes	chr7	exon	last exon	NA	1.11	no	up	0.50	no	1.11	no	up	6.0E-08	yes
MSTRG.256.3 : Syne1 : Q8VHJ9	0.04	yes	down	1.5E-19	yes	chr1	exon	first exon	NA	5.56	yes	up	0.14	no	0.89	no	down	8.9E-14	yes
ENSRNOT00000036035 : Vrk1 : Q6AYA2	0.22	yes	down	2.5E-07	yes	chr6	intron	internal intron	NA	0.94	no	down	0.91	no	1.01	no	up	2.7E-01	no
ENSRNOT00000019910 : Prpf4 : D4A7J8	0.23	yes	down	2.8E-04	yes	chr5	exon	last exon	NA	0.98	no	down	0.86	no	1.03	no	up	3.4E-04	yes
MSTRG.1158.2 : Mef2a : A0A0G2JSZ4	0.04	yes	down	2.4E-04	yes	chr1	exon	last exon	NA	0.82	no	down	0.58	no	0.78	yes	down	4.1E-07	yes
ENSRNOT00000066098 : Plpp1 : O08564	0.47	yes	down	8.3E-05	yes	chr2	intron	internal intron	NA	0.79	no	down	0.21	no	1.04	no	up	2.9E-03	yes
MSTRG.17520.5 : Srsf5 : Q09167	0.10	yes	down	3.0E-09	yes	chr6	promoter	Proximal	ICP	0.40	yes	down	0.41	no	1.03	no	up	4.2E-02	yes
MSTRG.6211.8 : Fcgr2b : A3RLA8	0.44	yes	down	2.8E-04	yes	chr13	promoter	Distal	LCP	1.30	no	up	0.64	no	1.04	no	up	4.3E-03	yes
ENSRNOT00000005977 : Cep131 : D4AEL8	3.69	yes	up	1.2E-04	yes	chr10	intron	last intron	NA	0.88	no	down	0.10	no	0.92	no	down	1.5E-02	yes
MSTRG.18880.6 : Plec : F7F9U6	0.33	yes	down	4.2E-06	yes	chr7	exon	last exon	NA	0.59	no	down	0.48	no	0.90	no	down	2.5E-03	yes
MSTRG.15479.1 : Prex2 : A0A0G2KA11	5.57	yes	up	1.6E-04	yes	chr5	intron	internal intron	NA	1.68	no	up	0.17	no	1.02	no	up	2.3E-01	no
ENSRNOT00000014810 : Bet1 : Q62896	0.17	yes	down	3.0E-07	yes	chr4	intron	first intron	NA	1.13	no	up	0.20	no	1.04	no	up	9.9E-04	yes
MSTRG.14695.3 : Ptcd3 : D3ZGM1	3.88	yes	up	4.1E-35	yes	chr4	intron	internal intron	NA	2.41	yes	up	0.09	no	0.98	no	down	3.1E-01	no
ENSRNOT00000010481 : Tram1 : Q5XI41	3.29	yes	up	2.9E-05	yes	chr5	exon	last exon	NA	1.19	no	up	0.19	no	1.13	no	up	1.2E-09	yes
MSTRG.7470.13 : Acin1 : E9PST5	0.30	yes	down	1.1E-04	yes	chr15	promoter	Distal	LCP	1.02	no	up	0.97	no	0.98	no	down	5.1E-04	yes
MSTRG.10799.1 : Gyg1 : F8WFR6	0.52	yes	down	3.8E-05	yes	chr2	intron	internal intron	NA	1.44	no	up	0.52	no	1.17	no	up	1.9E-09	yes
ENSRNOT00000075425 : Ahsa1 : B0BN63	0.11	yes	down	3.3E-06	yes	chr6	exon	last exon	NA	1.21	no	up	0.37	no	1.00	no	up	4.2E-01	no
ENSRNOT00000022373 : Zcchc6 : D3ZKR9	0.14	yes	down	1.2E-08	yes	chr17	exon	internal exon	NA	0.99	no	down	0.32	no	1.12	no	up	2.3E-06	yes
ENSRNOT00000092945 : Picalm : A0A1B0GWW9	8.74	yes	up	2.1E-13	yes	chr1	intron	internal intron	NA	0.63	no	down	0.04	yes	1.04	no	up	1.0E-01	no
ENSRNOT00000088549 : Itgav : F1LZX9	24.74	yes	up	1.9E-17	yes	chr3	intron	first intron	NA	1.06	no	up	0.76	no	0.92	no	down	1.7E-15	yes
MSTRG.10007.4 : Ctcf : Q9R1D1	2.29	yes	up	1.4E-04	yes	chr19	intron	internal intron	NA	1.79	no	up	0.11	no	0.94	no	down	4.5E-07	yes
MSTRG.14924.1 : Xpc : D4A3D8	20.37	yes	up	1.0E-05	yes	chr4	exon	internal exon	NA	1.11	no	up	0.35	no	0.95	no	down	3.2E-02	yes
MSTRG.7503.2 : Gmpr2 : A0A0G2JX25	0.33	yes	down	2.2E-06	yes	chr15	promoter	Distal	LCP	0.91	no	down	0.09	no	0.95	no	down	1.0E-01	no
MSTRG.9568.8 : Nedd4l : F1LRN8	2.03	yes	up	3.6E-05	yes	chr18	intron	internal intron	NA	0.60	no	down	0.19	no	0.95	no	down	3.9E-02	yes
ENSRNOT00000066808 : Ctnnbl1 : Q4V8K2	5.63	yes	up	4.6E-05	yes	chr3	intron	first intron	NA	1.04	no	up	0.57	no	1.01	no	up	3.8E-01	no
ENSRNOT00000005033 : Acot9 : Q5U2X8	0.09	yes	down	5.0E-06	yes	chrX	promoter	Distal	LCP	1.00	no	down	0.97	no	0.99	no	down	5.1E-02	no
MSTRG.7470.7 : Acin1 : E9PST5	0.30	yes	down	1.1E-04	yes	chr15	promoter	Distal	LCP	1.56	no	up	0.22	no	0.98	no	down	5.1E-04	yes
ENSRNOT00000086424 : Tmem38a : A6ZIQ8	9.90	yes	up	7.7E-05	yes	chr16	promoter	Intermediate	LCP	0.98	no	down	0.92	no	0.79	yes	down	1.0E-01	no
ENSRNOT00000084350 : Sec24c : A0A0G2JZF0	3.50	yes	up	1.3E-09	yes	chr15	intron	internal intron	NA	1.36	no	up	0.51	no	1.01	no	up	2.6E-01	no
ENSRNOT00000085530 : LOC361016 : A0A0G2JXI	0.05	yes	down	2.2E-18	yes	chr15	intron	first intron	NA	0.88	no	down	0.07	no	1.05	no	up	2.5E-01	no
MSTRG.8345.13 : Fgfr1 : F1LM54	0.22	yes	down	3.0E-05	yes	chr16	exon	last exon	NA	0.73	no	down	0.28	no	1.05	no	up	1.3E-02	yes
ENSRNOT00000079407 : Masp2 : A2VCV7	0.27	yes	down	4.0E-08	yes	chr5	intron	internal intron	NA	1.14	no	up	0.22	no	1.00	no	down	8.1E-01	no
ENSRNOT00000035400 : Fcgr2b : A3RLA8	0.44	yes	down	2.8E-04	yes	chr13	promoter	Distal	LCP	1.18	no	up	0.64	no	1.04	no	up	4.3E-03	yes
ENSRNOT00000021716 : Coq9 : Q68FT1	0.32	yes	down	5.1E-11	yes	chr19	promoter	Distal	LCP	1.42	no	up	0.59	no	0.93	no	down	1.8E-05	yes
MSTRG.14118.4 : Kmt2c : A0A0G2K426	0.22	yes	down	1.1E-11	yes	chr4	promoter	Distal	LCP	1.13	no	up	0.75	no	0.69	yes	down	1.4E-11	yes
ENSRNOT00000023385 : Ephx2 : P80299	0.14	yes	down	5.5E-05	yes	chr15	exon	internal exon	NA	0.89	no	down	0.57	no	0.95	no	down	6.7E-02	no
MSTRG.3481.2 : Tsr1 : D3ZEM8	45.59	yes	up	3.4E-04	yes	chr10	intron	internal intron	NA	0.97	no	down	0.39	no	1.00	no	down	9.7E-01	no
MSTRG.1443.8 : Arap1 : F1LM60	2.71	yes	up	3.3E-04	yes	chr1	intron	first intron	NA	0.71	no	down	0.41	no	0.99	no	down	2.5E-02	yes
MSTRG.13405.1 : Tmem62 : D3ZIW4	0.44	yes	down	9.9E-05	yes	chr3	exon	last exon	NA	0.49	yes	down	0.40	no	1.03	no	up	1.0E-01	no
MSTRG.20927.5 : Aox1 : F1LRQ1	0.36	yes	down	1.5E-07	yes	chr9	intron	internal intron	NA	0.66	no	down	0.37	no	0.73	yes	down	3.5E-17	yes
ENSRNOT00000031607 : Pmpca : Q68FX8	0.39	yes	down	1.4E-04	yes	chr3	intron	internal intron	NA	1.16	no	up	0.38	no	1.03	no	up	5.9E-03	yes
MSTRG.16523.4 : Arid1a : D4A3E3	0.22	yes	down	1.1E-04	yes	chr5	intron	first intron	NA	1.00	no	down	0.99	no	1.00	no	down	2.1E-01	no
ENSRNOT00000034450 : Krt1 : A0A0G2JST3	21.78	yes	up	3.6E-04	yes	chr7	promoter	Distal	LCP	0.73	no	down	0.02	yes	0.36	yes	down	4.2E-17	yes
MSTRG.4726.1 : Kalrn : P97924	1.54	yes	up	9.1E-07	yes	chr11	exon	last exon	NA	0.55	no	down	0.16	no	1.04	no	up	1.6E-04	yes
ENSRNOT00000079513 : Wnk1 : Q9JIH7	18.77	yes	up	1.6E-38	yes	chr4	exon	internal exon	NA	1.00	no	down	0.96	no	1.04	no	up	1.7E-03	yes
MSTRG.2854.3 : Abca3 : A0A0G2K1Q8	0.49	yes	down	3.5E-07	yes	chr10	intron	internal intron	NA	0.42	yes	down	0.05	yes	1.05	no	up	1.5E-04	yes
ENSRNOT00000084882 : Mark4 : A0A0G2JTD8	0.28	yes	down	3.4E-05	yes	chr1	intron	internal intron	NA	1.06	no	up	0.90	no	0.99	no	down	7.5E-01	no
ENSRNOT00000075382 : Arhgef10l : M0R7W2	2.87	yes	up	9.9E-07	yes	chr5	intron	internal intron	NA	1.00	no	down	0.23	no	1.13	no	up	8.4E-04	yes
ENSRNOT00000087604 : Mtch2 : A0A0G2K459	2.54	yes	up	4.3E-04	yes	chr3	intron	internal intron	NA	1.27	no	up	0.23	no	1.00	no	up	8.3E-01	no
MSTRG.16748.1 : Tardbp : I6L9G6	2.86	yes	up	4.5E-10	yes	chr5	intron	internal intron	NA	1.02	no	up	0.93	no	1.05	no	up	5.8E-11	yes
MSTRG.11539.1 : RGD1310209 : F1MAB2	0.28	yes	down	3.0E-04	yes	chr2	exon	last exon	NA	0.73	no	down	0.47	no	1.30	yes	up	8.2E-07	yes
ENSRNOT00000076795 : Yipf6 : A0A096MJG6	inf	yes	up	3.8E-06	yes	chrX	intron	first intron	NA	0.96	no	down	0.50	no	1.03	no	up	1.9E-01	no
ENSRNOT00000091909 : Osbpl1a : A0A0G2K327	0.28	yes	down	2.2E-05	yes	chr18	intron	internal intron	NA	0.69	no	down	0.17	no	0.94	no	down	2.8E-02	yes
ENSRNOT00000075511 : Ensa : P60841	2.35	yes	up	1.3E-05	yes	chr2	exon	last exon	NA	0.93	no	down	0.81	no	1.04	no	up	5.7E-02	no
MSTRG.8598.1 : Sfxn1 : Q63965	0.56	yes	down	3.6E-04	yes	chr17	intron	internal intron	NA	3.09	yes	up	0.01	yes	1.22	yes	up	2.3E-08	yes
ENSRNOT00000082037 : Srrm1 : B2RYB3	2.05	yes	up	2.0E-04	yes	chr5	intron	internal intron	NA	1.25	no	up	0.28	no	1.10	no	up	2.8E-06	yes
MSTRG.7561.7 : Phf11b : M0RB46	3.03	yes	up	2.9E-04	yes	chr15	promoter	Intermediate	LCP	1.69	no	up	0.29	no	1.15	no	up	2.3E-06	yes
MSTRG.14118.5 : Kmt2c : A0A0G2K426	0.22	yes	down	1.1E-11	yes	chr4	promoter	Distal	LCP	1.66	no	up	0.23	no	0.69	yes	down	1.4E-11	yes
MSTRG.9900.4 : Rnaseh2a : Q5U209	14.16	yes	up	1.1E-05	yes	chr19	intron	last intron	NA	1.17	no	up	0.54	no	1.08	no	up	2.6E-05	yes
ENSRNOT00000090381 : Cd63 : Q9QZV0	0.47	yes	down	3.6E-06	yes	chr7	promoter	Intermediate	ICP	1.31	no	up	0.47	no	0.56	yes	down	4.8E-03	yes
ENSRNOT00000080712 : Zw10 : Q4V8C2	7.96	yes	up	7.6E-05	yes	chr8	intron	last intron	NA	1.03	no	up	0.78	no	1.01	no	up	2.9E-01	no
MSTRG.11953.17 : RT1-CE7 : D3ZLE6	inf	yes	up	7.3E-07	yes	chr20	exon	internal exon	NA	0.99	no	down	0.96	no	1.22	yes	up	7.3E-16	yes
ENSRNOT00000002020 : Gtf2i : Q5U2Y1	5.31	yes	up	4.9E-06	yes	chr12	promoter	Distal	LCP	0.94	no	down	0.67	no	0.95	no	down	1.5E-07	yes
ENSRNOT00000023326 : Cspg4 : Q00657	3.02	yes	up	6.9E-06	yes	chr8	exon	internal exon	NA	1.23	no	up	0.16	no	1.08	no	up	7.3E-14	yes

MSTRG.18129.4 : Nfic : O70188	1.68	yes	up	1.4E-06	yes	chr7	intron	internal intron	NA	1.00	no	down	0.97	no	0.85	no	down	3.8E-12	yes
MSTRG.323.12 : LOC108348175 : A0A0G2JUS0	0.05	yes	down	4.0E-19	yes	chr1	promoter	Intermediate	LCP	0.86	no	down	0.39	no	1.01	no	up	4.0E-01	no
ENSRNOT00000058387 : Focad : F1LU27	5.61	yes	up	3.5E-06	yes	chr5	intron	internal intron	NA	0.94	no	down	0.51	no	1.10	no	up	1.2E-08	yes
MSTRG.3349.2 : Senp3 : Q3MID8	0.16	yes	down	5.8E-06	yes	chr10	exon	internal exon	NA	1.33	no	up	0.08	no	0.90	no	down	1.2E-11	yes
ENSRNOT00000081023 : Fat1 : G3V9W9	0.12	yes	down	1.5E-10	yes	chr16	exon	last exon	NA	2.39	yes	up	0.24	no	1.12	no	up	2.9E-12	yes
MSTRG.13466.3 : Myef2 : A0A0G2K402	2.63	yes	up	2.7E-04	yes	chr3	promoter	Intermediate	LCP	2.62	yes	up	0.09	no	0.93	no	down	4.5E-09	yes
MSTRG.20698.4 : Tram2 : F1LUA2	3	yes	up	2.1E-05	yes	chr9	intron	first intron	NA	0.62	no	down	0.55	no	1.02	no	up	1.2E-01	no
ENSRNOT00000061515 : Clasp1 : F1LNR1	0.36	yes	down	6.7E-07	yes	chr13	intron	internal intron	NA	1.02	no	up	0.47	no	1.00	no	down	7.0E-01	no
MSTRG.12111.3 : Cmtr1 : Q5U2Z5	4.09	yes	up	4.3E-15	yes	chr20	intron	internal intron	NA	1.12	no	up	0.12	no	1.00	no	down	9.7E-01	no
MSTRG.1283.4 : Tm6sf1 : D4A3E7	0.12	yes	down	9.3E-08	yes	chr1	intron	first intron	NA	1.06	no	up	0.70	no	1.05	no	up	5.5E-03	yes
MSTRG.2257.3 : Ddb1 : G3V8T4	inf	yes	up	1.9E-05	yes	chr1	intron	internal intron	NA	1.65	no	up	0.48	no	0.97	no	down	4.7E-09	yes
ENSRNOT00000010180 : Cd63 : Q9QZV0	0.47	yes	down	3.6E-06	yes	chr7	promoter	Intermediate	ICP	1.02	no	up	0.92	no	0.56	yes	down	4.8E-03	yes
ENSRNOT00000092754 : Gtf2i : Q5U2Y1	5.31	yes	up	4.9E-06	yes	chr12	promoter	Distal	LCP	1.02	no	up	0.75	no	0.95	no	down	1.5E-07	yes
ENSRNOT00000078500 : Tm6sf1 : D4A3E7	0.12	yes	down	9.3E-08	yes	chr1	intron	first intron	NA	0.98	no	down	0.91	no	1.05	no	up	5.5E-03	yes
ENSRNOT00000093625 : Kmt2c : A0A0G2K426	0.22	yes	down	1.1E-11	yes	chr4	promoter	Distal	LCP	1.22	no	up	0.60	no	0.69	yes	down	1.4E-11	yes
MSTRG.18206.7 : Cirbp : P60825	0.73	yes	down	1.3E-04	yes	chr7	exon	first exon	NA	0.82	no	down	0.59	no	0.85	no	down	3.4E-16	yes
ENSRNOT00000049245 : Pcdh7 : Q68HB8	0	yes	down	4.2E-04	yes	chr14	exon	last exon	NA	1.16	no	up	0.57	no	0.94	no	down	1.0E-07	yes
ENSRNOT00000092700 : Tecpr1 : Q3ZBA0	0.43	yes	down	3.1E-04	yes	chr12	promoter	Intermediate	LCP	1.02	no	up	0.73	no	1.17	no	up	7.7E-08	yes
MSTRG.12973.6 : Rbms1 : A0A0G2K4R7	0	yes	down	3.3E-05	yes	chr3	intron	internal intron	NA	1.00	no	down	0.47	no	0.97	no	down	1.2E-01	no
MSTRG.4866.3 : Vps8 : D4A5F7	7.39	yes	up	3.4E-05	yes	chr11	intron	internal intron	NA	1.28	no	up	0.08	no	0.95	no	down	3.2E-03	yes
MSTRG.7965.3 : Bmpr1a : Q78EA7	0.29	yes	down	2.1E-04	yes	chr16	intron	internal intron	NA	0.78	no	down	0.59	no	0.90	no	down	9.8E-10	yes
ENSRNOT00000043902 : Tnpo3 : D4AAM0	6.44	yes	up	4.2E-12	yes	chr4	promoter	Distal	LCP	0.98	no	down	0.82	no	1.04	no	up	4.3E-04	yes
MSTRG.5482.12 : Ncor2 : A0A0G2JU91	2.16	yes	up	1.6E-06	yes	chr12	intron	internal intron	NA	1.21	no	up	0.59	no	1.01	no	up	7.1E-01	no
MSTRG.2257.1 : Ddb1 : G3V8T4	inf	yes	up	1.9E-05	yes	chr1	intron	internal intron	NA	1.78	no	up	0.46	no	0.97	no	down	4.7E-09	yes
MSTRG.97.12 : Bclaf1 : B1WC16	0.67	yes	down	1.3E-07	yes	chr1	intron	internal intron	NA	0.70	no	down	0.24	no	1.03	no	up	6.8E-02	no
ENSRNOT00000049180 : Adcy5 : G3V9G1	0.16	yes	down	2.3E-10	yes	chr11	intron	internal intron	NA	1.13	no	up	0.75	no	1.00	no	down	7.6E-01	no
MSTRG.18495.2 : Tbc1d15 : F7EZ89	0	yes	down	1.6E-05	yes	chr7	exon	internal exon	NA	1.08	no	up	0.73	no	0.98	no	down	2.3E-02	yes
MSTRG.17586.4 : Ylpm1 : A0A0G2K678	0.14	yes	down	2.3E-07	yes	chr6	intron	internal intron	NA	1.04	no	up	0.85	no	1.02	no	up	1.8E-03	yes
MSTRG.12226.2 : Bcr : F1LXF1	2.12	yes	up	1.3E-04	yes	chr20	intron	first intron	NA	0.82	no	down	0.40	no	0.96	no	down	8.7E-05	yes
MSTRG.20963.2 : Nbeal1 : F1M6V0	4.79	yes	up	6.8E-08	yes	chr9	intron	internal intron	NA	0.86	no	down	0.54	no	1.00	no	up	5.1E-01	no
MSTRG.10325.3 : Cast : F1LPH1	0.36	yes	down	1.6E-06	yes	chr2	intron	internal intron	NA	1.09	no	up	0.72	no	0.95	no	down	2.4E-10	yes
MSTRG.276.6 : Arid1b : F1LNP1	1.53	yes	up	6.5E-08	yes	chr1	promoter	Proximal	LCP	0.32	yes	down	0.04	yes	0.98	no	down	1.8E-01	no
MSTRG.21122.5 : Agfg1 : F1M9N7	3.59	yes	up	1.8E-08	yes	chr9	exon	internal exon	NA	0.91	no	down	0.75	no	1.16	no	up	6.0E-06	yes
MSTRG.12626.1 : Col5a1 : A0A0G2JX47	0.33	yes	down	4.5E-04	yes	chr3	intron	internal intron	NA	1.00	no	down	1.00	no	1.01	no	up	4.0E-01	no
ENSRNOT00000018670 : Senp3 : Q3MID8	0.16	yes	down	5.8E-06	yes	chr10	exon	internal exon	NA	1.04	no	up	0.28	no	0.90	no	down	1.2E-11	yes
MSTRG.6203.1 : Olfml2b : D4A0J7	0.13	yes	down	2.7E-04	yes	chr13	exon	internal exon	NA	1.03	no	up	0.96	no	1.06	no	up	4.0E-07	yes
ENSRNOT00000016751 : Uqcrh : Q5M9I5	15.45	yes	up	1.6E-15	yes	chr5	exon	last exon	NA	0.78	no	down	0.04	yes	0.98	no	down	3.9E-01	no
ENSRNOT00000024575 : Tpm1 : A0A0G2JSQ4	9.41	yes	up	5.1E-14	yes	chr8	intron	internal intron	NA	0.69	no	down	0.37	no	0.81	yes	down	2.5E-10	yes
MSTRG.9106.2 : Nebl : F1LVX3	0	yes	down	3.8E-04	yes	chr17	intron	first intron	NA	1.00	no	down	0.04	yes	1.02	no	up	2.0E-01	no
MSTRG.15110.5 : Wnk1 : Q9JIH7	18.77	yes	up	1.6E-38	yes	chr4	exon	internal exon	NA	2.38	yes	up	0.09	no	1.04	no	up	1.7E-03	yes
ENSRNOT00000017838 : Myl1 : P02600	2.29	yes	up	4.3E-05	yes	chr9	promoter	Distal	LCP	1.05	no	up	0.19	no	1.33	yes	up	6.1E-13	yes
ENSRNOT00000050321 : Erlec1 : D3ZF97	0.05	yes	down	1.8E-18	yes	chr14	intron	internal intron	NA	0.94	no	down	0.17	no	1.05	no	up	1.1E-04	yes
ENSRNOT00000093657 : Timm50 : D3ZJX5	0.54	yes	down	3.3E-07	yes	chr1	exon	last exon	NA	1.04	no	up	0.72	no	0.98	no	down	4.2E-02	yes
ENSRNOT00000026580 : Lamp1 : P14562	0.27	yes	down	5.9E-08	yes	chr16	promoter	Distal	LCP	1.12	no	up	0.47	no	1.10	no	up	5.4E-06	yes
ENSRNOT00000035628 : Ywhaz : P63102	3.68	yes	up	2.7E-04	yes	chr7	intron	last intron	NA	1.08	no	up	0.74	no	0.87	no	down	1.2E-13	yes
MSTRG.3379.2 : Dlg4 : P31016	2.15	yes	up	8.6E-07	yes	chr10	intron	last intron	NA	1.23	no	up	0.42	no	0.97	no	down	9.1E-02	no
MSTRG.13466.2 : Myef2 : A0A0G2K402	2.63	yes	up	2.7E-04	yes	chr3	promoter	Intermediate	LCP	0.89	no	down	0.75	no	0.93	no	down	4.5E-09	yes
MSTRG.9433.3 : Rbm27 : F1M1R4	0.45	yes	down	2.5E-04	yes	chr18	intron	internal intron	NA	0.76	no	down	0.15	no	1.01	no	up	5.2E-01	no
MSTRG.7470.12 : Acin1 : E9PST5	0.30	yes	down	1.1E-04	yes	chr15	promoter	Distal	LCP	0.64	no	down	0.12	no	0.98	no	down	5.1E-04	yes
ENSRNOT00000051857 : Hdhd2 : Q6QI86	0.20	yes	down	5.1E-06	yes	chr18	intron	first intron	NA	1.03	no	up	0.38	no	1.04	no	up	3.3E-03	yes
MSTRG.5025.7 : Pds5b : D3ZU56	0.26	yes	down	2.1E-04	yes	chr12	promoter	Intermediate	LCP	1.04	no	up	0.90	no	0.96	no	down	4.7E-06	yes
MSTRG.9435.1 : Tcerg1 : B5DEZ4	0.26	yes	down	2.8E-04	yes	chr18	promoter	Intermediate	ICP	0.99	no	down	0.99	no	1.02	no	up	4.4E-02	yes
MSTRG.10007.5 : Ctcf : Q9R1D1	2.29	yes	up	1.4E-04	yes	chr19	intron	internal intron	NA	0.86	no	down	0.56	no	0.94	no	down	4.5E-07	yes
ENSRNOT00000080453 : Apoe : P02650	0.23	yes	down	3.2E-04	yes	chr1	exon	last exon	NA	0.99	no	down	0.95	no	0.86	no	down	6.2E-12	yes
MSTRG.14075.5 : Dnajc5 : A0A0G2JX56	0.30	yes	down	1.1E-06	yes	chr3	promoter	Intermediate	HCP	0.57	no	down	0.16	no	0.89	no	down	4.0E-08	yes
MSTRG.5340.1 : Serpine1 : F1LM16	2.28	yes	up	2.6E-04	yes	chr12	exon	last exon	NA	0.49	yes	down	0.32	no	1.00	no	down	7.7E-01	no
ENSRNOT00000014354 : Cachd1 : D4ADK4	1.98	yes	up	2.7E-04	yes	chr5	intron	internal intron	NA	0.98	no	down	0.90	no	0.99	no	down	6.0E-01	no
MSTRG.21273.2 : Fer : A0A140TAC4	3.31	yes	up	2.7E-04	yes	chr9	promoter	Proximal	LCP	1.33	no	up	0.05	yes	0.92	no	down	7.3E-07	yes
MSTRG.9598.1 : Cep192 : D4A3X0	4.22	yes	up	1.3E-12	yes	chr18	intron	internal intron	NA	1.11	no	up	0.20	no	0.95	no	down	9.1E-04	yes
MSTRG.9598.3 : Cep192 : D4A3X0	4.22	yes	up	1.3E-12	yes	chr18	intron	internal intron	NA	0.93	no	down	0.43	no	0.95	no	down	9.1E-04	yes
MSTRG.11642.3 : Tifa : Q5XIB9	0.42	yes	down	9.5E-05	yes	chr2	exon	internal exon	NA	1.51	no	up	0.08	no	1.16	no	up	2.8E-04	yes
ENSRNOT00000092388 : Shank3 : A0A0U1RS13	3.23	yes	up	4.3E-05	yes	chr7	exon	first exon	NA	1.06	no	up	0.15	no	0.98	no	down	1.0E-02	yes
MSTRG.20878.2 : Gls : P13264	0.05	yes	down	1.5E-13	yes	chr9	intron	first intron	NA	0.96	no	down	0.95	no	1.08	no	up	4.1E-02	yes
MSTRG.18557.7 : Mon2 : D3ZCG3	0.07	yes	down	1.5E-08	yes	chr7	intron	internal intron	NA	2.62	yes	up	0.21	no	1.05	no	up	7.1E-07	yes
MSTRG.7470.11 : Acin1 : E9PST5	0.30	yes	down	1.1E-04	yes	chr15	promoter	Distal	LCP	1.12	no	up	0.76	no	0.98	no	down	5.1E-04	yes
MSTRG.16080.1 : Cachd1 : D4ADK4	1.98	yes	up	2.7E-04	yes	chr5	intron	internal intron	NA	0.86	no	down	0.26	no	0.99	no	down	6.0E-01	no
MSTRG.18495.1 : Tbc1d15 : F7EZ89	0	yes	down	1.6E-05	yes	chr7	exon	internal exon	NA	1.07	no	up	0.73	no	0.98	no	down	2.3E-02	yes
MSTRG.21604.2 : Tbl1x : B2RZA6	0	yes	down	6.0E-05	yes	chrX	intron	internal intron	NA	0.84	no	down	0.45	no	1.12	no	up	7.8E-09	yes
MSTRG.16636.4 : Ubr4 : A0A0G2JU89	2.84	yes	up	3.7E-05	yes	chr5	intron	internal intron	NA	0.58	no	down	0.41	no	1.02	no	up	4.6E-05	yes
MSTRG.2433.1 : Rnls : Q5U2W9	0.31	yes	down	2.4E-05	yes	chr1	promoter	Distal	LCP	0.73	no	down	0.32	no	1.02	no	up	3.0E-01	no
MSTRG.4525.4 : Mx1 : Q499S4	3.65	yes	up	1.1E-13	yes	chr11	intron	internal intron	NA	0.90	no	down	0.42	no	1.36	yes	up	1.2E-13	yes
ENSRNOT00000086694 : Aff1 : D3ZBU5	1.49	yes	up	9.8E-06	yes	chr14	intron	internal intron	NA	1.35	no	up	0.59	no	1.07	no	up	4.0E-02	yes
ENSRNOT00000023543 : H6pd : D4A7D7	0.22	yes	down	2.6E-06	yes	chr5	intron	last intron	NA	1.20	no	up	0.39	no	1.14	no	up	9.6E-15	yes
MSTRG.8665.3 : Kif13a : D3ZM20	0	yes	down	7.8E-05	yes	chr17	intron	internal intron	NA	0.64	no	down	0.45	no	1.00	no	up	9.5E-01	no
ENSRNOT00000089477 : Phc3 : D3ZS50	0.36	yes	down	4.0E-04	yes	chr2	intron	internal intron	NA	1.21	no	up	0.69	no	0.86	no	down	4.2E-04	yes
MSTRG.7470.3 : Acin1 : E9PST5	0.30	yes	down	1.1E-04	yes	chr15	promoter	Distal	LCP	1.22	no	up	0.74	no	0.98	no	down	5.1E-04	yes
ENSRNOT00000063930 : Pstpip1 : B0BNK4	0.47	yes	down	3.6E-05	yes	chr8	intron	internal intron	NA	0.98	no	down	0.91	no	1.20	yes	up	1.1E-06	yes
ENSRNOT00000089279 : Krt8 : Q10758	0.19	yes	down	1.4E-04	yes	chr7	exon	last exon	NA	0.80	no	down	0.26	no	0.83	yes	down	4.5E-14	yes
MSTRG.19148.4 : Shank3 : A0A0U1RS13	3.23	yes	up	4.3E-05	yes	chr7	exon	first exon	NA	1.28	no	up	0.05	yes	0.98	no	down	1.0E-02	yes
MSTRG.3447.2 : Atp2a3 : G3V9U7	inf	yes	up	2.1E-05	yes	chr10	intron	internal intron	NA	0.92	no	down	0.27	no	0.77	yes	down	1.7E-16	yes
ENSRNOT00000017379 : Naa15 : D3ZD89	0.53	yes	down	3.9E-04	yes	chr2	exon	last exon	NA	1.52	no	up	0.41	no	1.05	no	up	5.6E-06	yes
MSTRG.6191.8 : Ddr2 : B1WC09	0.49	yes	down	3.3E-05	yes	chr13	exon	internal exon	NA	1.36	no	up	0.47	no	0.95	no	down	3.2E-01	no
MSTRG.19824.1 : Arih1 : D3ZXL1	0.23	yes	down	8.6E-06	yes	chr8	intron	first intron	NA	1.57	no	up	0.24	no	0.93	no	down	1.4E-04	yes
ENSRNOT00000023368 : Plg : Q01177	7.88	yes	up	1.6E-04	yes	chr1	intron	internal intron	NA	1.03	no	up	0.25	no	1.05	no	up	7.8E-06	yes
ENSRNOT00000089538 : Nf1 : F1LM28	2.12	yes	up	4.3E-05	yes	chr10	intron	internal intron	NA	1.07	no	up	0.55	no	0.98	no	down	1.9E-01	no
ENSRNOT00000085843 : Myzap : Q5EB94	0.16	yes	down	3.6E-06	yes	chr8	exon	last exon	NA	1.02	no	up	0.78	no	0.84	no	down	4.4E-10	yes
MSTRG.3572.6 : RGD1307929 : F1LSX1	7.34	yes	up	1.1E-12	yes	chr10	intron	internal intron	NA	1.13	no	up	0.09	no	0.97	no	down	1.8E-04	yes
MSTRG.17299.1 : Heatr5a : F1LSK5	0.05	yes	down	7.6E-05	yes	chr6	intron	internal intron	NA	2.30	yes	up	0.03	yes	0.98	no	down	2.4E-01	no
MSTRG.21446.3 : Tfe3 : D3ZAW6	0.07	yes	down	2.9E-11	yes	chrX	exon	last exon	NA	2.23	yes	up	0.09	no	1.04	no	up	1.6E-03	yes
ENSRNOT00000064060 : Tmem38a : A6ZIQ8	9.90	yes	up	7.7E-05	yes	chr16	promoter	Intermediate	LCP	0.87	no	down	0.64	no	0.79	yes	down	1.0E-01	no
MSTRG.9317.1 : Ctnna1 : Q5U302	2.58	yes	up	1.7E-05	yes	chr18	intron	internal intron	NA	0.78	no	down	0.81	no	0.96	no	down	3.1E-09	yes
MSTRG.11511.1 : Rbm15 : M0R3Z8	4.02	yes	up	1.9E-06	yes	chr2	exon	last exon	NA	1.00	no	down	0.99	no	0.94	no	down	2.6E-06	yes
MSTRG.256.7 : Syne1 : Q8VHJ9	0.04	yes	down	1.5E-19	yes	chr1	exon	first exon	NA	0.31	yes	down	0.40	no	0.89	no	down	8.9E-14	yes
ENSRNOT00000025415 : Picalm : Q66WT9	8.74	yes	up	2.1E-13	yes	chr1	intron	internal intron	NA	0.69	no	down	0.22	no	1.45	yes	up	1.6E-10	yes
ENSRNOT00000079923 : Snrpb : B0BN51	0.11	yes	down	3.8E-04	yes	chr3	intron	last intron	NA	1.06	no	up	0.67	no	0.98	no	down	4.0E-02	yes
ENSRNOT00000093632 : Gpx4 : A0A0G2K398	0.42	yes	down	9.7E-05	yes	chr7	intron	internal intron	NA	0.98	no	down	0.82	no	0.91	no	down	1.6E-05	yes
MSTRG.17785.4 : Dync1h1 : M0R9X8	0.19	yes	down	1.5E-06	yes	chr6	intron	internal intron	NA	1.08	no	up	0.65	no	0.99	no	down	3.4E-03	yes
ENSRNOT00000092248 : Lamp1 : P14562	0.27	yes	down	5.9E-08	yes	chr16	promoter	Distal	LCP	1.02	no	up	0.82	no	1.10	no	up	5.4E-06	yes
ENSRNOT00000025392 : Jph3 : D3ZWH2	0.51	yes	down	5.7E-04	yes	chr19	intron	internal intron	NA	0.91	no	down	0.15	no	1.14	no	up	2.7E-07	yes
MSTRG.3645.7 : Slfn13 : A0A096MKD0	2.27	yes	up	1.5E-04	yes	chr10	promoter	Intermediate	LCP	0.63	no	down	0.40	no	0.97	no	down	2.7E-02	yes

MSTRG.19963.6 : Sltm : A0A0G2K904	0.34	yes	down	6.4E-06	yes	chr8	intron	internal intron	NA	0.83	no	down	0.62	no	0.94	no	down	2.8E-02	yes
ENSRNOT00000092936 : Clasp1 : F1LNR1	0.36	yes	down	6.7E-07	yes	chr13	intron	internal intron	NA	1.00	no	down	0.87	no	1.00	no	down	7.0E-01	no
MSTRG.19125.4 : Brd1 : D3ZUW8	7.17	yes	up	3.3E-04	yes	chr7	exon	internal exon	NA	0.81	no	down	0.83	no	1.21	yes	up	6.4E-02	no
ENSRNOT00000082138 : Ubr4 : A0A0G2JU89	2.84	yes	up	3.7E-05	yes	chr5	intron	internal intron	NA	2.69	yes	up	0.14	no	1.02	no	up	4.6E-05	yes
MSTRG.14024.1 : Gnas : P63095	0.77	yes	down	3.7E-04	yes	chr3	promoter	Distal	HCP	1.12	no	up	0.20	no	0.90	no	down	5.6E-15	yes
MSTRG.16753.1 : Pex14 : Q642G4	2.11	yes	up	2.4E-04	yes	chr5	intron	internal intron	NA	1.95	no	up	0.10	no	1.04	no	up	2.1E-03	yes
MSTRG.3895.7 : Cdk12 : A0A0G2K5U7	5.94	yes	up	1.1E-04	yes	chr10	exon	first exon	NA	1.22	no	up	0.41	no	0.97	no	down	5.5E-04	yes
MSTRG.13067.2 : Scrn3 : A0A0G2K189	9.14	yes	up	1.0E-04	yes	chr3	intron	first intron	NA	1.65	no	up	0.29	no	0.92	no	down	8.4E-04	yes
MSTRG.7470.18 : Acin1 : E9PST5	0.30	yes	down	1.1E-04	yes	chr15	promoter	Distal	LCP	0.78	no	down	0.28	no	0.98	no	down	5.1E-04	yes
MSTRG.21176.12 : Inpp5d : F1M981	0.08	yes	down	9.9E-05	yes	chr9	intron	internal intron	NA	0.77	no	down	0.27	no	1.08	no	up	3.1E-05	yes
MSTRG.11263.6 : Atp8b2 : D4A509	0.14	yes	down	3.1E-04	yes	chr2	exon	first exon	NA	0.95	no	down	0.81	no	0.92	no	down	5.0E-03	yes
ENSRNOT00000002180 : Ltn1 : F1M9Q3	0.38	yes	down	4.2E-04	yes	chr11	intron	internal intron	NA	2.64	yes	up	0.12	no	1.08	no	up	1.4E-11	yes
MSTRG.7229.2 : Usp54 : Q6IE24	0.36	yes	down	1.9E-04	yes	chr15	intron	internal intron	NA	0.40	yes	down	0.50	no	0.83	yes	down	7.0E-09	yes
ENSRNOT00000076558 : Nebl : F1LVX3	0	yes	down	3.8E-04	yes	chr17	intron	first intron	NA	1.01	no	up	0.48	no	1.02	no	up	2.0E-01	no
MSTRG.19220.7 : Kmt2d : A0A0G2JVD6	6.53	yes	up	2.1E-09	yes	chr7	exon	last exon	NA	1.25	no	up	0.80	no	1.03	no	up	3.5E-02	yes
ENSRNOT00000083855 : RGD1310507 : A0A0G2K	10.49	yes	up	4.2E-04	yes	chr8	intron	internal intron	NA	0.72	no	down	0.21	no	1.03	no	up	1.5E-02	yes
MSTRG.13391.1 : Lrrc57 : Q5FVI3	0.39	yes	down	6.1E-06	yes	chr3	intron	internal intron	NA	1.04	no	up	0.92	no	0.91	no	down	5.9E-09	yes
ENSRNOT00000065950 : Ppp6r3 : D3ZBT9	3.21	yes	up	8.1E-07	yes	chr1	intron	internal intron	NA	1.07	no	up	0.11	no	0.98	no	down	7.0E-03	yes
MSTRG.6713.2 : Fryl : D3ZQY4	2.76	yes	up	1.7E-04	yes	chr14	intron	internal intron	NA	0.69	no	down	0.09	no	1.00	no	down	4.6E-01	no
ENSRNOT00000045628 : Tf : P12346	5.10	yes	up	5.5E-06	yes	chr8	intron	first intron	NA	1.34	no	up	0.46	no	1.33	yes	up	1.6E-08	yes
MSTRG.20764.1 : Uggt1 : Q9JLA3	0.22	yes	down	3.9E-04	yes	chr9	intron	internal intron	NA	2.04	yes	up	0.45	no	1.11	no	up	9.8E-15	yes
ENSRNOT00000045180 : Cc2d1a : Q66HA5	0.21	yes	down	1.7E-05	yes	chr19	promoter	Distal	LCP	1.01	no	up	0.85	no	0.96	no	down	4.1E-02	yes
MSTRG.123.4 : Epb41l2 : D3ZM69	0.25	yes	down	1.1E-12	yes	chr1	intron	internal intron	NA	2.19	yes	up	0.07	no	0.91	no	down	8.9E-11	yes
ENSRNOT00000017749 : Cfh : F1M983	0.20	yes	down	2.4E-04	yes	chr13	intron	internal intron	NA	1.46	no	up	0.09	no	1.20	no	up	2.7E-15	yes
MSTRG.11607.2 : Bcar3 : D3ZAZ5	3.50	yes	up	1.2E-08	yes	chr2	intron	internal intron	NA	0.76	no	down	0.19	no	1.06	no	up	1.2E-02	yes
ENSRNOT00000013232 : Grip2 : A0A0H2UHH8	2.18	yes	up	2.2E-05	yes	chr4	intron	internal intron	NA	0.91	no	down	0.74	no	1.01	no	up	5.2E-01	no
ENSRNOT00000092872 : Aox1 : F1LRQ1	0.36	yes	down	1.5E-07	yes	chr9	intron	internal intron	NA	1.00	no	down	0.60	no	0.73	yes	down	3.5E-17	yes
ENSRNOT00000050832 : Synm : G3V9G5	1.68	yes	up	3.0E-04	yes	chr1	exon	last exon	NA	1.24	no	up	0.44	no	0.82	yes	down	8.8E-13	yes
MSTRG.6480.2 : Evi5 : D3ZJN9	4.04	yes	up	4.0E-04	yes	chr14	intron	internal intron	NA	0.28	yes	down	0.20	no	0.98	no	down	2.5E-01	no
MSTRG.3249.11 : Specc1 : A0A0G2K5D7	5.53	yes	up	1.9E-10	yes	chr10	exon	last exon	NA	0.69	no	down	0.28	no	0.93	no	down	4.4E-09	yes
ENSRNOT00000008337 : Aco1 : G3V6S2	4.49	yes	up	2.7E-06	yes	chr5	intron	internal intron	NA	1.44	no	up	0.25	no	0.93	no	down	1.1E-11	yes
ENSRNOT00000008035 : Oxsm : G3V6R7	7.88	yes	up	2.7E-09	yes	chr15	exon	last exon	NA	1.01	no	up	0.94	no	1.01	no	up	5.6E-01	no
MSTRG.7098.1 : Egfr : E7CXR8	0.25	yes	down	9.1E-09	yes	chr14	exon	internal exon	NA	0.94	no	down	0.80	no	0.99	no	down	2.7E-01	no
ENSRNOT00000044896 : Ephb3 : D3ZH39	1.63	yes	up	6.2E-06	yes	chr11	intron	internal intron	NA	0.95	no	down	0.25	no	0.86	no	down	1.2E-07	yes
MSTRG.10603.4 : Nnt : Q5BJZ3	3.00	yes	up	1.8E-04	yes	chr2	intron	internal intron	NA	1.04	no	up	0.85	no	0.96	no	down	1.2E-07	yes
ENSRNOT00000024933 : Ampd3 : O09178	0.39	yes	down	3.1E-04	yes	chr1	intron	internal intron	NA	0.88	no	down	0.74	no	0.94	no	down	8.5E-03	yes
MSTRG.20655.1 : Cdc5l : O08837	4.40	yes	up	1.7E-04	yes	chr9	intron	internal intron	NA	1.32	no	up	0.23	no	1.05	no	up	3.5E-05	yes
MSTRG.4937.2 : Gp1bb : Q9JJM7	0	yes	down	2.2E-04	yes	chr11	intron	internal intron	NA	1.18	no	up	0.40	no	0.93	no	down	7.7E-08	yes
MSTRG.12240.1 : Bicc1 : A0A0G2K0Y0	5.19	yes	up	5.0E-04	yes	chr20	intron	internal intron	NA	3.74	yes	up	0.13	no	1.52	yes	up	1.2E-05	yes
MSTRG.4866.1 : Vps8 : D4A5F7	7.39	yes	up	3.4E-05	yes	chr11	intron	internal intron	NA	1.17	no	up	0.50	no	0.95	no	down	3.2E-03	yes
ENSRNOT00000043646 : Itpr1 : A0A0A0MY31	3.16	yes	up	1.1E-05	yes	chr4	intron	internal intron	NA	2.46	yes	up	0.37	no	0.87	no	down	1.1E-14	yes
MSTRG.8598.1 : Sfxn1 : Q6AYS2	0.56	yes	down	3.6E-04	yes	chr17	intron	internal intron	NA	3.09	yes	up	0.01	yes	1.09	no	up	2.5E-03	yes
MSTRG.21367.2 : Uba1 : Q5U300	0	yes	down	1.9E-08	yes	chrX	exon	last exon	NA	1.05	no	up	0.40	no	0.93	no	down	2.8E-14	yes
MSTRG.16557.3 : Srrm1 : B2RYB3	2.05	yes	up	2.0E-04	yes	chr5	intron	internal intron	NA	2.30	yes	up	0.04	yes	1.10	no	up	2.8E-06	yes
MSTRG.17811.2 : Mark3 : F1M836	0.30	yes	down	2.6E-04	yes	chr6	exon	last exon	NA	0.90	no	down	0.68	no	1.10	no	up	4.1E-07	yes
MSTRG.5467.2 : Stx2 : Q7TS57	4.45	yes	up	6.0E-04	yes	chr12	exon	last exon	NA	1.40	no	up	0.07	no	0.91	no	down	2.6E-04	yes
MSTRG.6568.3 : Antxr2 : Q00IM8	1.10	no	up	9.8E-05	yes	chr14	intron	internal intron	NA	0.72	no	down	0.39	no	1.01	no	up	3.8E-01	no
ENSRNOT00000076113 : Asph : A0A096MKE0	2.56	yes	up	9.8E-06	yes	chr5	intron	internal intron	NA	1.30	no	up	0.02	yes	0.99	no	down	1.3E-01	no
MSTRG.8278.33 : Rbpms : F2Z3S5	0.02	yes	down	2.6E-05	yes	chr16	promoter	Proximal	ICP	0.99	no	down	0.35	no	1.06	no	up	4.9E-05	yes
MSTRG.13954.2 : Znfx1 : F1LMA9	20.79	yes	up	3.0E-04	yes	chr3	intron	internal intron	NA	1.62	no	up	0.48	no	0.92	no	down	1.0E-03	yes
MSTRG.15546.3 : Asph : A0A096MKE0	2.56	yes	up	9.8E-06	yes	chr5	intron	internal intron	NA	0.65	no	down	0.36	no	0.99	no	down	1.3E-01	no
ENSRNOT00000035590 : Hydin : D3ZBX6	3.95	yes	up	2.0E-07	yes	chr19	intron	internal intron	NA	0.95	no	down	0.51	no	0.80	yes	down	2.3E-10	yes
MSTRG.16327.14 : Macf1 : A0A0G2K9T4	0.36	yes	down	1.3E-05	yes	chr5	exon	last exon	NA	1.16	no	up	0.82	no	0.97	no	down	9.0E-11	yes
MSTRG.323.6 : LOC108348175 : A0A0G2JUS0	0.05	yes	down	4.0E-19	yes	chr1	promoter	Intermediate	LCP	1.18	no	up	0.67	no	1.01	no	up	4.0E-01	no
ENSRNOT00000090923 : Lims2 : A0A0G2KAE1	0.67	yes	down	6.6E-13	yes	chr18	intron	internal intron	NA	0.60	no	down	0.17	no	0.82	yes	down	4.4E-10	yes
MSTRG.7482.1 : Pabpn1 : G3V7Z8	0.52	yes	down	3.9E-04	yes	chr15	intron	last intron	NA	0.91	no	down	0.42	no	1.02	no	up	3.4E-02	yes
ENSRNOT00000016084 : RGD1307929 : F1LSX1	7.34	yes	up	1.1E-12	yes	chr10	intron	internal intron	NA	2.08	yes	up	0.02	yes	0.97	no	down	1.8E-04	yes
MSTRG.611.1 : Apoe : P02650	0.23	yes	down	3.2E-04	yes	chr1	exon	last exon	NA	1.22	no	up	0.70	no	0.86	no	down	6.2E-12	yes
ENSRNOT00000005987 : Cltc : F1M779	0.27	yes	down	1.4E-04	yes	chr10	intron	internal intron	NA	1.30	no	up	0.04	yes	1.04	no	up	1.3E-11	yes
MSTRG.8278.24 : Rbpms : F2Z3S5	0.02	yes	down	2.6E-05	yes	chr16	promoter	Proximal	ICP	1.07	no	up	0.72	no	1.06	no	up	4.9E-05	yes
MSTRG.4725.10 : Kalrn : P97924	1.54	yes	up	9.1E-07	yes	chr11	exon	last exon	NA	0.69	no	down	0.03	yes	1.04	no	up	1.6E-04	yes
MSTRG.6211.2 : Fcgr2b : A3RLA8	0.44	yes	down	2.8E-04	yes	chr13	promoter	Distal	LCP	1.26	no	up	0.46	no	1.04	no	up	4.3E-03	yes
MSTRG.7179.1 : Ccdc88a : D3ZYD7	6.58	yes	up	8.2E-05	yes	chr14	promoter	Distal	LCP	1.02	no	up	0.98	no	1.03	no	up	5.3E-03	yes
MSTRG.15361.2 : Recql : Q6AYJ1	0.74	yes	down	1.2E-04	yes	chr4	exon	last exon	NA	1.07	no	up	0.85	no	0.97	no	down	6.1E-03	yes
ENSRNOT00000000717 : Rpf2 : B0BN82	0.50	yes	down	5.1E-06	yes	chr20	intron	internal intron	NA	1.19	no	up	0.13	no	0.96	no	down	2.2E-02	yes
ENSRNOT00000005262 : Cpd : A0A0G2JY30	0.15	yes	down	4.3E-04	yes	chr10	intron	internal intron	NA	1.16	no	up	0.36	no	1.13	no	up	1.1E-08	yes
MSTRG.10322.1 : Cast : F1LPH1	0.36	yes	down	1.6E-06	yes	chr2	intron	internal intron	NA	4.41	yes	up	0.17	no	0.95	no	down	2.4E-10	yes
MSTRG.20817.7 : Map4k4 : A0A0G2K7W4	0.36	yes	down	7.1E-05	yes	chr9	intron	internal intron	NA	1.30	no	up	0.46	no	1.10	no	up	7.1E-06	yes
ENSRNOT00000024576 : Abhd17a : Q5XIJ5	3.11	yes	up	7.4E-06	yes	chr7	exon	internal exon	NA	0.93	no	down	0.58	no	0.84	no	down	6.6E-10	yes
ENSRNOT00000064043 : Tmem245 : D3ZXD8	5.92	yes	up	3.2E-05	yes	chr5	intron	internal intron	NA	0.88	no	down	0.69	no	0.88	no	down	4.0E-07	yes
MSTRG.2881.6 : Pkd1 : Q9ERV0	inf	yes	up	6.6E-05	yes	chr10	intron	internal intron	NA	2.03	yes	up	0.21	no	1.19	no	up	9.8E-07	yes
ENSRNOT00000060695 : Skiv2l2 : D4AE49	5.78	yes	up	1.8E-10	yes	chr2	intron	internal intron	NA	0.99	no	down	0.89	no	0.99	no	down	1.0E-01	no
MSTRG.9334.2 : Tmem173 : F1M391	inf	yes	up	7.6E-06	yes	chr18	intron	last intron	NA	1.27	no	up	0.07	no	1.18	no	up	2.5E-10	yes
ENSRNOT00000018232 : Dok3 : B2RYG7	9.95	yes	up	1.6E-09	yes	chr17	promoter	Distal	LCP	0.84	no	down	0.62	no	1.15	no	up	1.1E-05	yes
MSTRG.7561.9 : Phf11b : M0RB46	3.03	yes	up	2.9E-04	yes	chr15	promoter	Intermediate	LCP	1.44	no	up	0.48	no	1.15	no	up	2.3E-06	yes
ENSRNOT00000024707 : Sfxn1 : Q6AYS2	0.56	yes	down	3.6E-04	yes	chr17	intron	internal intron	NA	1.20	no	up	0.78	no	1.09	no	up	2.5E-03	yes
MSTRG.18661.2 : Ywhaz : P63102	3.68	yes	up	2.7E-04	yes	chr7	intron	last intron	NA	0.31	yes	down	0.20	no	0.87	no	down	1.2E-13	yes
ENSRNOT00000092998 : Clasp1 : F1LNR1	0.36	yes	down	6.7E-07	yes	chr13	intron	internal intron	NA	1.09	no	up	0.82	no	1.00	no	down	7.0E-01	no
MSTRG.14881.1 : Aplf : F6Q5G6	0.12	yes	down	8.1E-06	yes	chr4	exon	last exon	NA	1.24	no	up	0.37	no	0.99	no	down	2.8E-01	no
ENSRNOT00000031352 : Cnot11 : B0BNA9	15.80	yes	up	2.2E-05	yes	chr9	promoter	Distal	ICP	1.20	no	up	0.03	yes	0.98	no	down	1.2E-01	no
MSTRG.18268.11 : Akap8 : Q63014	0.08	yes	down	2.7E-04	yes	chr7	intron	internal intron	NA	1.28	no	up	0.06	no	0.99	no	down	2.8E-01	no
MSTRG.1545.3 : Sbf2 : B5DEJ9	4.77	yes	up	8.9E-06	yes	chr1	intron	internal intron	NA	1.66	no	up	0.48	no	1.03	no	up	9.5E-02	no
ENSRNOT00000091448 : Ddx3x : A0A0G2K719	3.18	yes	up	1.4E-05	yes	chrX	exon	first exon	NA	1.03	no	up	0.83	no	1.11	no	up	3.1E-07	yes
MSTRG.21083.13 : Speg : Q63638	0.17	yes	down	2.6E-06	yes	chr9	intron	internal intron	NA	1.34	no	up	0.06	no	0.88	no	down	2.3E-07	yes
MSTRG.11285.7 : Npr1 : P18910	0.37	yes	down	1.2E-04	yes	chr2	intron	internal intron	NA	1.74	no	up	0.09	no	1.02	no	up	1.6E-02	yes
ENSRNOT00000089243 : Grb14 : O88900	1.86	yes	up	4.5E-07	yes	chr3	intron	internal intron	NA	1.00	no	down	0.99	no	1.11	no	up	6.5E-07	yes
MSTRG.13963.5 : Ptgis : Q62969	0.55	yes	down	6.0E-05	yes	chr3	intron	internal intron	NA	0.84	no	down	0.83	no	0.99	no	down	7.4E-02	no
MSTRG.12337.2 : Sgpl1 : Q8CHN6	0.20	yes	down	6.8E-07	yes	chr20	intron	internal intron	NA	1.14	no	up	0.79	no	1.12	no	up	5.6E-16	yes
ENSRNOT00000073746 : Pnpo : O88794	2.62	yes	up	1.2E-06	yes	chr10	intron	internal intron	NA	1.09	no	up	0.43	no	0.99	no	down	6.7E-01	no
MSTRG.11265.14 : Ubap2l : E9PTR4	6.62	yes	up	4.0E-04	yes	chr2	intron	internal intron	NA	1.16	no	up	0.02	yes	1.13	no	up	3.8E-12	yes
MSTRG.8915.2 : Rala : P63322	0.27	yes	down	4.5E-04	yes	chr17	promoter	Intermediate	LCP	1.02	no	up	0.96	no	0.88	no	down	5.2E-09	yes
ENSRNOT00000018982 : Acin1 : E9PST5	0.30	yes	down	1.1E-04	yes	chr15	promoter	Distal	LCP	1.31	no	up	0.28	no	0.98	no	down	5.1E-04	yes
MSTRG.15557.6 : Ints8 : A0A0G2K0V1	0.11	yes	down	2.1E-05	yes	chr5	intron	last intron	NA	1.09	no	up	0.70	no	1.07	no	up	1.6E-05	yes
MSTRG.1333.2 : Dlg2 : F1M907	2.35	yes	up	1.4E-05	yes	chr1	intron	internal intron	NA	0.70	no	down	0.30	no	0.92	no	down	2.8E-06	yes
ENSRNOT00000072855 : Gas7 : M0R4R4	4.60	yes	up	4.0E-11	yes	chr10	intron	first intron	NA	0.71	no	down	0.29	no	1.22	yes	up	2.5E-15	yes
MSTRG.5906.8 : Atp2b4 : Q64542	2.31	yes	up	1.3E-05	yes	chr13	intron	internal intron	NA	1.02	no	up	0.94	no	0.68	yes	down	2.6E-19	yes
MSTRG.1158.1 : Mef2a : A0A0G2JSZ4	0.04	yes	down	2.4E-04	yes	chr1	exon	last exon	NA	1.44	no	up	0.35	no	0.78	yes	down	4.1E-07	yes
MSTRG.6572.2 : Bmp2k : F1M7M4	11.81	yes	up	5.6E-07	yes	chr14	intron	internal intron	NA	1.19	no	up	0.47	no	1.09	no	up	3.4E-07	yes
MSTRG.15392.1 : Itpr2 : P29995	2.12	yes	up	2.5E-04	yes	chr4	intron	internal intron	NA	0.98	no	down	0.87	no	0.79	yes	down	4.4E-05	yes
MSTRG.8345.5 : Fgfr1 : F1LM54	0.22	yes	down	3.0E-05	yes	chr16	exon	last exon	NA	0.66	no	down	0.49	no	1.05	no	up	1.3E-02	yes

MSTRG.15720.2 : Pigo : D3ZTP8	0.61	yes	down	2.7E-05	yes	chr5	exon	internal exon	NA	0.55	no	down	0.31	no	0.80	yes	down	9.0E-03	yes
MSTRG.3379.4 : Dlg4 : P31016	2.15	yes	up	8.6E-07	yes	chr10	intron	last intron	NA	1.18	no	up	0.49	no	0.97	no	down	9.1E-02	no
MSTRG.11405.7 : Pde4dip : A0A0G2JW66	6.94	yes	up	5.0E-08	yes	chr2	exon	internal exon	NA	0.95	no	down	0.26	no	1.06	no	up	5.1E-04	yes
MSTRG.323.2 : LOC108348175 : A0A0G2JUS0	0.05	yes	down	4.0E-19	yes	chr1	promoter	Intermediate	LCP	1.00	no	down	0.35	no	1.01	no	up	4.0E-01	no
MSTRG.4508.5 : Dyrk1a : Q63470	0.26	yes	down	2.2E-05	yes	chr11	intron	internal intron	NA	1.51	no	up	0.48	no	1.02	no	up	1.6E-01	no
MSTRG.19538.12 : Nfrkb : D4A421	0.58	yes	down	1.3E-05	yes	chr8	intron	internal intron	NA	1.31	no	up	0.09	no	0.89	no	down	2.5E-03	yes
MSTRG.6320.6 : Cdc42bpa : G3V6C9	8.92	yes	up	2.1E-05	yes	chr13	intron	internal intron	NA	0.57	no	down	0.50	no	0.97	no	down	2.5E-04	yes
MSTRG.4992.5 : Top3b : D4A9Z2	11.41	yes	up	1.2E-04	yes	chr11	intron	last intron	NA	0.71	no	down	0.38	no	0.99	no	down	6.1E-01	no
MSTRG.3353.3 : Zbtb4 : D4A8X0	inf	yes	up	2.4E-05	yes	chr10	intron	last intron	NA	0.53	no	down	0.21	no	0.97	no	down	6.9E-02	no
MSTRG.21083.14 : Speg : Q63638	0.17	yes	down	2.6E-06	yes	chr9	intron	internal intron	NA	0.75	no	down	0.00	yes	0.88	no	down	2.3E-07	yes
ENSRNOT00000060311 : Tcp11l1 : F1M9Y7	3.13	yes	up	1.2E-06	yes	chr3	intron	last intron	NA	1.00	no	down	0.47	no	0.99	no	down	8.5E-01	no
ENSRNOT00000031812 : Dmxl1 : D4AA13	0.19	yes	down	1.3E-04	yes	chr18	intron	internal intron	NA	0.95	no	down	0.88	no	0.95	no	down	3.8E-03	yes
MSTRG.21491.3 : Phf8 : D4AD31	0	yes	down	2.1E-07	yes	chrX	exon	first exon	NA	1.00	no	down	0.47	no	1.13	no	up	3.6E-02	yes
MSTRG.12274.1 : Ctnna3 : B2RYN9	0	yes	down	1.3E-06	yes	chr20	promoter	Distal	LCP	0.77	no	down	0.44	no	1.03	no	up	1.7E-01	no
MSTRG.7804.4 : Mycbp2 : D4A2D3	0.31	yes	down	9.2E-06	yes	chr15	intron	internal intron	NA	1.12	no	up	0.47	no	1.00	no	up	6.8E-01	no
MSTRG.18268.26 : Akap8 : Q63014	0.08	yes	down	2.7E-04	yes	chr7	intron	internal intron	NA	0.75	no	down	0.72	no	0.99	no	down	2.8E-01	no
ENSRNOT00000029615 : Usp54 : Q6IE24	0.36	yes	down	1.9E-04	yes	chr15	intron	internal intron	NA	1.55	no	up	0.61	no	0.83	yes	down	7.0E-09	yes
MSTRG.16327.6 : Macf1 : A0A0G2K9T4	0.36	yes	down	1.3E-05	yes	chr5	exon	last exon	NA	1.19	no	up	0.78	no	0.97	no	down	9.0E-11	yes
ENSRNOT00000078909 : Plcg1 : G3V845	0.24	yes	down	1.1E-04	yes	chr3	intron	first intron	NA	1.20	no	up	0.44	no	0.94	no	down	1.8E-09	yes
MSTRG.5614.1 : Tbx3 : A0A0G2K8D7	2.83	yes	up	2.0E-10	yes	chr12	intron	internal intron	NA	1.27	no	up	0.24	no	0.99	no	down	7.3E-01	no
ENSRNOT00000037164 : Nup214 : D4ACK1	0.13	yes	down	4.7E-04	yes	chr3	intron	internal intron	NA	0.89	no	down	0.45	no	0.99	no	down	6.9E-01	no
ENSRNOT00000013126 : Sbf2 : B5DEJ9	4.77	yes	up	8.9E-06	yes	chr1	intron	internal intron	NA	0.94	no	down	0.42	no	1.03	no	up	9.5E-02	no
ENSRNOT00000084660 : Ctnna3 : B2RYN9	0	yes	down	1.3E-06	yes	chr20	promoter	Distal	LCP	1.09	no	up	0.69	no	1.03	no	up	1.7E-01	no
ENSRNOT00000092857 : Trim21 : D4ACF2	0.20	yes	down	5.7E-10	yes	chr1	promoter	Distal	LCP	1.00	no	down	0.68	no	0.96	no	down	1.3E-04	yes
MSTRG.7780.4 : Lmo7 : A0A0G2K174	3.35	yes	up	1.2E-04	yes	chr15	intron	internal intron	NA	0.44	yes	down	0.29	no	0.96	no	down	4.2E-06	yes
MSTRG.6327.2 : Acbd3 : G3V6E4	2.42	yes	up	1.4E-04	yes	chr13	intron	internal intron	NA	0.86	no	down	0.69	no	1.07	no	up	2.4E-14	yes
ENSRNOT00000010947 : Dnm2 : A0A0A0MY48	0.11	yes	down	8.9E-11	yes	chr8	intron	first intron	NA	1.05	no	up	0.54	no	1.05	no	up	2.0E-08	yes
MSTRG.16755.4 : Kif1b : A0A0G2KA12	3.65	yes	up	7.4E-05	yes	chr5	intron	internal intron	NA	0.86	no	down	0.87	no	1.05	no	up	6.5E-02	no
MSTRG.6480.6 : Evi5 : D3ZJN9	4.04	yes	up	4.0E-04	yes	chr14	intron	internal intron	NA	1.53	no	up	0.09	no	0.98	no	down	2.5E-01	no
ENSRNOT00000092975 : Dnajc11 : B1WBY5	2.59	yes	up	1.9E-14	yes	chr5	promoter	Intermediate	ICP	1.49	no	up	0.03	yes	0.88	no	down	4.2E-12	yes
MSTRG.20422.1 : Rpl14 : Q63507	6.79	yes	up	7.4E-07	yes	chr8	intron	internal intron	NA	0.90	no	down	0.59	no	1.09	no	up	1.1E-04	yes
MSTRG.123.3 : Epb41l2 : D3ZM69	0.25	yes	down	1.1E-12	yes	chr1	intron	internal intron	NA	1.05	no	up	0.32	no	0.91	no	down	8.9E-11	yes
MSTRG.13952.3 : Stau1 : Q9ESY8	0.08	yes	down	6.3E-05	yes	chr3	intron	internal intron	NA	0.99	no	down	0.97	no	1.05	no	up	2.2E-05	yes
ENSRNOT00000067907 : Tbc1d15 : F7EZ89	0	yes	down	1.6E-05	yes	chr7	exon	internal exon	NA	1.12	no	up	0.69	no	0.98	no	down	2.3E-02	yes
ENSRNOT00000006884 : Rtfdc1 : Q3T1J8	0	yes	down	2.5E-04	yes	chr3	exon	internal exon	NA	1.00	no	down	0.92	no	1.01	no	up	3.6E-01	no
MSTRG.13559.4 : Hspa12b : D3ZVM5	0.65	yes	down	3.5E-04	yes	chr3	intron	last intron	NA	0.75	no	down	0.15	no	0.86	no	down	5.9E-13	yes
ENSRNOT00000048528 : Pitpnm2 : A0A0G2JW50	2.70	yes	up	3.9E-04	yes	chr12	promoter	Distal	LCP	1.06	no	up	0.35	no	0.89	no	down	1.9E-08	yes
MSTRG.15309.1 : Mansc1 : D4AE21	0.45	yes	down	1.5E-04	yes	chr4	exon	first exon	NA	1.28	no	up	0.57	no	0.85	no	down	5.3E-06	yes
ENSRNOT00000090870 : Mitd1 : Q5I0J5	0.26	yes	down	9.9E-05	yes	chr9	promoter	Intermediate	LCP	1.12	no	up	0.12	no	1.14	no	up	9.0E-08	yes
MSTRG.10569.4 : Plpp1 : O08564	0.47	yes	down	8.3E-05	yes	chr2	intron	internal intron	NA	1.04	no	up	0.90	no	1.04	no	up	2.9E-03	yes
MSTRG.7804.2 : Mycbp2 : A0A1W2Q6I3	0.31	yes	down	9.2E-06	yes	chr15	intron	internal intron	NA	1.07	no	up	0.82	no	1.00	no	up	8.8E-01	no
MSTRG.14349.1 : Cped1 : A0A0G2QC10	1.61	yes	up	4.5E-04	yes	chr4	intron	internal intron	NA	1.24	no	up	0.72	no	1.07	no	up	1.5E-03	yes
ENSRNOT00000051846 : Cacna1c : F1MA84	4.75	yes	up	4.3E-04	yes	chr4	intron	internal intron	NA	1.00	no	down	0.88	no	0.97	no	down	2.7E-02	yes
ENSRNOT00000068018 : Lnpk : A0JN29	3.42	yes	up	1.2E-11	yes	chr3	intron	internal intron	NA	1.19	no	up	0.36	no	0.96	no	down	2.6E-04	yes
MSTRG.7186.3 : Sptbn1 : A0A0G2K8W9	5.72	yes	up	4.0E-04	yes	chr14	intron	internal intron	NA	2.74	yes	up	0.49	no	0.87	no	down	1.1E-18	yes
MSTRG.10322.2 : Cast : F1LPH1	0.36	yes	down	1.6E-06	yes	chr2	intron	internal intron	NA	1.18	no	up	0.50	no	0.95	no	down	2.4E-10	yes
MSTRG.13195.2 : Arhgap1 : D4A6C5	3.73	yes	up	1.8E-11	yes	chr3	intron	internal intron	NA	1.11	no	up	0.84	no	1.04	no	up	1.7E-04	yes
ENSRNOT00000092945 : Picalm : Q66SY1	8.74	yes	up	2.1E-13	yes	chr1	intron	internal intron	NA	0.63	no	down	0.04	yes	1.13	no	up	1.1E-06	yes
ENSRNOT00000076880 : Slc33a1 : Q6AYY8	0.10	yes	down	8.7E-08	yes	chr2	exon	last exon	NA	1.08	no	up	0.63	no	1.05	no	up	1.4E-05	yes
MSTRG.5034.1 : Insr : F1LPL6	0.36	yes	down	9.6E-05	yes	chr12	intron	internal intron	NA	3.63	yes	up	0.00	yes	1.09	no	up	1.4E-04	yes
MSTRG.19194.3 : Hdac7 : A0A0G2K6B1	1.57	yes	up	6.8E-05	yes	chr7	intron	internal intron	NA	1.53	no	up	0.18	no	0.99	no	down	6.7E-01	no
MSTRG.18268.27 : Akap8 : Q63014	0.08	yes	down	2.7E-04	yes	chr7	intron	internal intron	NA	0.62	no	down	0.53	no	0.99	no	down	2.8E-01	no
MSTRG.22089.1 : Htatsf1 : D4A997	6.87	yes	up	2.7E-04	yes	chrX	promoter	Intermediate	HCP	1.13	no	up	0.84	no	0.95	no	down	9.0E-07	yes
MSTRG.9889.10 : Nf1x : F2Z3R4	0.58	yes	down	1.9E-06	yes	chr19	intron	internal intron	NA	1.53	no	up	0.20	no	0.77	yes	down	2.4E-14	yes
MSTRG.20831.5 : Tgfbrap1 : D3ZXT8	0.12	yes	down	2.5E-04	yes	chr9	intron	internal intron	NA	0.75	no	down	0.50	no	0.98	no	down	2.9E-01	no
ENSRNOT00000086041 : Gbf1 : A0A0G2K3N1	0.28	yes	down	1.1E-05	yes	chr1	intron	internal intron	NA	1.45	no	up	0.47	no	1.05	no	up	6.1E-10	yes
ENSRNOT00000093422 : Phf8 : D4AD31	0	yes	down	2.1E-07	yes	chrX	exon	first exon	NA	0.96	no	down	0.40	no	1.13	no	up	3.6E-02	yes
MSTRG.9847.1 : Pkn1 : Q63433	1.53	yes	up	6.1E-04	yes	chr19	intron	internal intron	NA	0.60	no	down	0.08	no	1.01	no	up	5.4E-01	no
ENSRNOT00000055401 : Dlg2 : F1M907	2.35	yes	up	1.4E-05	yes	chr1	intron	internal intron	NA	1.05	no	up	0.56	no	0.92	no	down	2.8E-06	yes
ENSRNOT00000040762 : Plec : Q6S395	0.33	yes	down	4.2E-06	yes	chr7	exon	last exon	NA	1.05	no	up	0.87	no	1.03	no	up	2.2E-01	no
ENSRNOT00000040762 : Plec : F7F9U6	0.33	yes	down	4.2E-06	yes	chr7	exon	last exon	NA	1.05	no	up	0.87	no	0.90	no	down	2.5E-03	yes
MSTRG.7804.7 : Mycbp2 : A0A1W2Q6I3	0.31	yes	down	9.2E-06	yes	chr15	intron	internal intron	NA	0.48	yes	down	0.23	no	1.00	no	up	8.8E-01	no
MSTRG.14146.2 : Fam126a : A0A0G2K3C7	0.38	yes	down	2.9E-04	yes	chr4	promoter	Intermediate	LCP	1.06	no	up	0.66	no	1.07	no	up	1.8E-06	yes
MSTRG.1466.2 : Trim21 : D4ACF2	0.20	yes	down	5.7E-10	yes	chr1	promoter	Distal	LCP	0.75	no	down	0.45	no	0.96	no	down	1.3E-04	yes
MSTRG.21427.1 : Rbm3 : Q925G0	5.50	yes	up	1.2E-04	yes	chrX	intron	first intron	NA	0.87	no	down	0.69	no	1.28	yes	up	1.7E-10	yes
MSTRG.14948.2 : Magi1 : M0R8T1	0.38	yes	down	2.0E-04	yes	chr4	intron	internal intron	NA	1.26	no	up	0.27	no	0.99	no	down	6.0E-01	no
ENSRNOT00000036518 : Gstt3 : D3Z8I7	0.44	yes	down	5.4E-04	yes	chr20	promoter	Intermediate	LCP	0.83	no	down	0.63	no	0.92	no	down	4.5E-04	yes
MSTRG.7470.1 : Acin1 : E9PST5	0.30	yes	down	1.1E-04	yes	chr15	promoter	Distal	LCP	1.08	no	up	0.46	no	0.98	no	down	5.1E-04	yes
ENSRNOT00000085714 : Mcc : F1LZS6	43.12	yes	up	1.3E-14	yes	chr1	exon	last exon	NA	1.39	no	up	0.19	no	0.85	no	down	3.7E-04	yes
MSTRG.21085.1 : Chpf : Q5XIQ8	1.40	yes	up	4.1E-04	yes	chr9	intron	internal intron	NA	0.96	no	down	0.94	no	1.00	no	down	8.4E-01	no
MSTRG.21938.3 : Klhl13 : F1LM44	0.22	yes	down	7.1E-07	yes	chrX	intron	last intron	NA	0.87	no	down	0.69	no	1.03	no	up	6.8E-03	yes
ENSRNOT00000083045 : Lnpk : A0JN29	3.42	yes	up	1.2E-11	yes	chr3	intron	internal intron	NA	1.00	no	down	0.75	no	0.96	no	down	2.6E-04	yes
MSTRG.4012.1 : Ubtf : P25977	1.48	yes	up	1.5E-06	yes	chr10	intron	internal intron	NA	0.64	no	down	0.38	no	0.96	no	down	1.4E-06	yes
MSTRG.1731.2 : Tbc1d10b : D3ZSY8	1.59	yes	up	2.7E-04	yes	chr1	intron	internal intron	NA	1.15	no	up	0.46	no	0.99	no	down	3.1E-01	no
ENSRNOT00000007367 : Rbm3 : Q925G0	5.50	yes	up	1.2E-04	yes	chrX	intron	first intron	NA	0.51	no	down	0.21	no	1.28	yes	up	1.7E-10	yes
MSTRG.18129.1 : Nfic : O70188	1.68	yes	up	1.4E-06	yes	chr7	intron	internal intron	NA	0.76	no	down	0.51	no	0.85	no	down	3.8E-12	yes
MSTRG.19538.7 : Nfrkb : D4A421	0.58	yes	down	1.3E-05	yes	chr8	intron	internal intron	NA	1.07	no	up	0.52	no	0.89	no	down	2.5E-03	yes
MSTRG.2210.1 : Stx5 : Q08851	0	yes	down	9.9E-06	yes	chr1	exon	first exon	NA	1.13	no	up	0.33	no	1.01	no	up	5.9E-01	no
MSTRG.9889.4 : Nf1x : F2Z3R4	0.58	yes	down	1.9E-06	yes	chr19	intron	internal intron	NA	0.62	no	down	0.21	no	0.77	yes	down	2.4E-14	yes
MSTRG.7309.3 : Flnb : D4A8D5	1.70	yes	up	8.8E-05	yes	chr15	intron	last intron	NA	0.93	no	down	0.83	no	1.00	no	up	2.4E-01	no
ENSRNOT00000067327 : Trappc9 : B1H266	4.58	yes	up	1.4E-09	yes	chr7	intron	internal intron	NA	1.09	no	up	0.25	no	0.94	no	down	3.8E-02	yes
ENSRNOT00000077070 : Dpt : B2RZ77	6.14	yes	up	2.8E-04	yes	chr13	exon	last exon	NA	0.63	no	down	0.30	no	0.85	no	down	3.5E-10	yes
ENSRNOT00000003770 : Gfpt2 : Q4KMC4	0.77	yes	down	3.9E-05	yes	chr10	intron	internal intron	NA	1.51	no	up	0.10	no	1.43	yes	up	1.7E-17	yes
MSTRG.7350.7 : Ktn1 : D4A4Z9	2.49	yes	up	1.2E-04	yes	chr15	intron	internal intron	NA	1.06	no	up	0.09	no	1.04	no	up	2.2E-11	yes
MSTRG.986.7 : Akt1s1 : D3ZH75	2.93	yes	up	2.4E-05	yes	chr1	intron	internal intron	NA	0.99	no	down	0.93	no	0.97	no	down	7.2E-01	no
MSTRG.11964.1 : Ddx39b : Q63413	0.02	yes	down	4.7E-11	yes	chr20	exon	last exon	NA	1.43	no	up	0.37	no	1.01	no	up	2.0E-01	no
ENSRNOT00000072789 : Abca3 : A0A0G2K1Q8	0.49	yes	down	3.5E-07	yes	chr10	intron	internal intron	NA	0.90	no	down	0.28	no	1.05	no	up	1.5E-04	yes
MSTRG.13544.4 : Ptpra : Q03348	0.34	yes	down	1.6E-04	yes	chr3	exon	internal exon	NA	1.12	no	up	0.46	no	0.89	no	down	6.4E-12	yes
ENSRNOT00000004117 : Olfml2b : D4A0J7	0.13	yes	down	2.7E-04	yes	chr13	exon	internal exon	NA	0.82	no	down	0.51	no	1.06	no	up	4.0E-07	yes
MSTRG.6320.1 : Cdc42bpa : G3V6C9	8.92	yes	up	2.1E-05	yes	chr13	intron	internal intron	NA	1.25	no	up	0.38	no	0.97	no	down	2.5E-04	yes
ENSRNOT00000025255 : Scrn3 : A0A0G2K189	9.14	yes	up	1.0E-04	yes	chr3	intron	first intron	NA	0.95	no	down	0.40	no	0.92	no	down	8.4E-04	yes
ENSRNOT00000078336 : Eml2 : Q6P6T4	0.17	yes	down	4.6E-06	yes	chr1	intron	internal intron	NA	1.07	no	up	0.36	no	0.81	yes	down	6.9E-13	yes
ENSRNOT00000080100 : Dcun1d1 : D3ZRV0	1.60	yes	up	3.7E-04	yes	chr2	exon	internal exon	NA	1.00	no	down	0.89	no	0.97	no	down	2.6E-02	yes
ENSRNOT00000013378 : Rabgap1 : D3ZX42	3.82	yes	up	8.1E-06	yes	chr3	intron	internal intron	NA	1.23	no	up	0.79	no	1.01	no	up	1.9E-01	no
MSTRG.21083.11 : Speg : Q63638	0.17	yes	down	2.6E-06	yes	chr9	intron	internal intron	NA	0.84	no	down	0.50	no	0.88	no	down	2.3E-07	yes
MSTRG.9846.4 : Pkn1 : Q63433	1.53	yes	up	6.1E-04	yes	chr19	intron	internal intron	NA	1.23	no	up	0.07	no	1.01	no	up	5.4E-01	no
MSTRG.3572.2 : RGD1307929 : F1LSX1	7.34	yes	up	1.1E-12	yes	chr10	intron	internal intron	NA	0.53	no	down	0.44	no	0.97	no	down	1.8E-04	yes
ENSRNOT00000017624 : Tenm3 : F1LV44	3.57	yes	up	5.2E-08	yes	chr16	exon	internal exon	NA	0.74	no	down	0.40	no	1.03	no	up	3.0E-03	yes
ENSRNOT00000086979 : Tcerg1 : B5DEZ4	0.26	yes	down	2.8E-04	yes	chr18	promoter	Intermediate	ICP	1.28	no	up	0.36	no	1.02	no	up	4.4E-02	yes
MSTRG.12303.1 : Lims1 : C0KUC5	12.34	yes	up	5.9E-05	yes	chr20	intron	internal intron	NA	0.34	yes	down	0.12	no	0.77	yes	down	1.9E-12	yes

MSTRG.2354.1 : Ptar1 : D3ZWG9	2.31	yes	up	2.3E-04	yes	chr1	exon	internal exon	NA	1.09	no	up	0.23	no	0.95	no	down	1.2E-02	yes
MSTRG.21176.4 : Inpp5d : F1M981	0.08	yes	down	9.9E-05	yes	chr9	intron	internal intron	NA	1.05	no	up	0.29	no	1.08	no	up	3.1E-05	yes
ENSRNOT00000065576 : Recql : Q6AYJ1	0.74	yes	down	1.2E-04	yes	chr4	exon	last exon	NA	0.67	no	down	0.69	no	0.97	no	down	6.1E-03	yes
ENSRNOT00000030373 : Nmi : Q498S7	0.14	yes	down	8.2E-08	yes	chr3	exon	last exon	NA	0.65	no	down	0.28	no	1.02	no	up	4.1E-02	yes
MSTRG.16755.2 : Kif1b : A0A0G2KA12	3.65	yes	up	7.4E-05	yes	chr5	intron	internal intron	NA	1.04	no	up	0.88	no	1.05	no	up	6.5E-02	no
ENSRNOT00000083322 : Map4k4 : A0A0G2K7W4	0.36	yes	down	7.1E-05	yes	chr9	intron	internal intron	NA	1.40	no	up	0.55	no	1.10	no	up	7.1E-06	yes
MSTRG.1670.1 : Rbbp6 : G3V953	inf	yes	up	4.6E-05	yes	chr1	intron	internal intron	NA	0.92	no	down	0.44	no	1.09	no	up	2.1E-04	yes
MSTRG.4992.2 : Top3b : D4A9Z2	11.41	yes	up	1.2E-04	yes	chr11	intron	last intron	NA	1.08	no	up	0.94	no	0.99	no	down	6.1E-01	no
MSTRG.21039.8 : Tns1 : F1LN42	0.09	yes	down	2.0E-04	yes	chr9	intron	last intron	NA	2.19	yes	up	0.24	no	0.86	no	up	7.8E-19	yes
MSTRG.1443.7 : Arap1 : F1LM60	2.71	yes	up	3.3E-04	yes	chr1	intron	first intron	NA	1.25	no	up	0.67	no	0.99	no	down	2.5E-02	yes
ENSRNOT00000025467 : Tcerg1 : B5DEZ4	0.26	yes	down	2.8E-04	yes	chr18	promoter	Intermediate	ICP	1.01	no	up	0.98	no	1.02	no	up	4.4E-02	yes
MSTRG.20561.1 : Rftn1 : G3V7I2	3.70	yes	up	2.3E-04	yes	chr9	intron	internal intron	NA	0.66	no	down	0.40	no	1.12	no	up	1.5E-09	yes
ENSRNOT00000092781 : Filip1 : F1LM79	4.15	yes	up	2.1E-10	yes	chr8	exon	first exon	NA	1.00	no	down	0.48	no	1.01	no	up	3.0E-01	no
MSTRG.19538.15 : Nfrkb : D4A421	0.58	yes	down	1.3E-05	yes	chr8	intron	internal intron	NA	0.67	no	down	0.07	no	0.89	no	down	2.5E-03	yes
ENSRNOT00000083926 : Rpap3 : Q68FQ7	0.29	yes	down	4.9E-05	yes	chr7	intron	internal intron	NA	0.98	no	down	0.91	no	1.04	no	up	2.9E-04	yes
ENSRNOT00000021171 : Sfxn3 : Q6P6T0	0.39	yes	down	7.8E-05	yes	chr1	intron	internal intron	NA	0.92	no	down	0.57	no	1.12	no	up	6.4E-07	yes
ENSRNOT00000071929 : Rbpj : M0R7Q3	0.34	yes	down	7.0E-05	yes	chr14	intron	last intron	NA	0.99	no	down	0.76	no	1.08	no	up	1.1E-04	yes
MSTRG.751.3 : Timm50 : D3ZJX5	0.54	yes	down	3.3E-07	yes	chr1	exon	last exon	NA	0.98	no	down	0.97	no	0.98	no	down	4.2E-02	yes
ENSRNOT00000081786 : Btaf1 : A0A0G2K1N4	2.34	yes	up	6.1E-05	yes	chr1	intron	internal intron	NA	1.87	no	up	0.55	no	1.00	no	down	9.8E-01	no
MSTRG.10218.2 : Zc3h18 : Q6TQE1	0.03	yes	down	1.2E-11	yes	chr19	promoter	Intermediate	LCP	1.00	no	down	0.99	no	1.02	no	up	2.3E-01	no
MSTRG.7830.1 : Dock9 : F1LSM8	0.10	yes	down	2.7E-05	yes	chr15	intron	internal intron	NA	1.00	no	down	0.45	no	0.97	no	down	2.3E-04	yes
ENSRNOT00000087026 : Pde4dip : A0A0G2JW66	6.94	yes	up	5.0E-08	yes	chr2	exon	internal exon	NA	0.68	no	down	0.13	no	1.06	no	up	5.1E-04	yes
MSTRG.21682.3 : Yipf6 : A0A096MJG6	inf	yes	up	3.8E-06	yes	chrX	intron	first intron	NA	0.87	no	down	0.70	no	1.03	no	up	1.9E-01	no
ENSRNOT00000058497 : Fcgr2b : A3RLA8	0.44	yes	down	2.8E-04	yes	chr13	promoter	Distal	LCP	1.18	no	up	0.65	no	1.04	no	up	4.3E-03	yes
MSTRG.4335.3 : Pcyt2 : O88637	4.82	yes	up	3.0E-05	yes	chr10	intron	last intron	NA	1.50	no	up	0.25	no	0.94	no	down	1.8E-03	yes
MSTRG.12668.6 : Nup188 : F1LRC6	0.22	yes	down	2.3E-05	yes	chr3	exon	internal exon	NA	0.90	no	down	0.82	no	1.02	no	up	1.9E-01	no
ENSRNOT00000012270 : Sec24c : A0A0G2JZF0	3.50	yes	up	1.3E-09	yes	chr15	intron	internal intron	NA	1.92	no	up	0.45	no	1.01	no	up	2.6E-01	no
ENSRNOT00000012334 : Col5a1 : G3V763	0.33	yes	down	4.5E-04	yes	chr3	intron	internal intron	NA	0.89	no	down	0.53	no	1.08	no	up	2.2E-06	yes
ENSRNOT00000061516 : Clasp1 : F1LNR1	0.36	yes	down	6.7E-07	yes	chr13	intron	internal intron	NA	1.04	no	up	0.48	no	1.00	no	down	7.0E-01	no
MSTRG.3538.2 : Abr : A0A0G2JZZ7	inf	yes	up	3.7E-04	yes	chr10	intron	internal intron	NA	0.78	no	down	0.51	no	1.05	no	up	1.0E-03	yes
MSTRG.15356.1 : Pde3a : Q62865	0.30	yes	down	3.1E-07	yes	chr4	intron	internal intron	NA	1.86	no	up	0.43	no	1.00	no	up	9.7E-01	no
ENSRNOT00000085894 : Tpm1 : A0A0G2JSQ4	9.41	yes	up	5.1E-14	yes	chr8	intron	internal intron	NA	0.26	yes	down	0.16	no	0.81	yes	down	2.5E-10	yes
MSTRG.8364.1 : Ap3m2 : V9GZ82	2.61	yes	up	1.6E-06	yes	chr16	intron	internal intron	NA	0.98	no	down	0.72	no	0.97	no	down	9.9E-02	no
ENSRNOT00000045130 : Mon2 : D3ZCG3	0.07	yes	down	1.5E-08	yes	chr7	intron	internal intron	NA	0.73	no	down	0.66	no	1.05	no	up	7.1E-07	yes
MSTRG.11797.3 : Cryz : Q6AYT0	3.72	yes	up	1.8E-04	yes	chr2	intron	first intron	NA	0.99	no	down	0.77	no	0.91	no	down	9.1E-09	yes
ENSRNOT00000026040 : Ola1 : A0JPJ7	3.58	yes	up	9.2E-08	yes	chr3	promoter	Proximal	LCP	1.08	no	up	0.60	no	1.04	no	up	2.3E-07	yes
ENSRNOT00000013355 : Wnk1 : Q9JIH7	18.77	yes	up	1.6E-38	yes	chr4	exon	internal exon	NA	1.00	no	down	0.77	no	1.04	no	up	1.7E-03	yes
ENSRNOT00000009485 : Mien1 : D3ZSU7	0	yes	down	1.5E-07	yes	chr10	exon	last exon	NA	1.24	no	up	0.62	no	1.01	no	up	3.2E-01	no
MSTRG.2510.1 : Tm9sf3 : D3ZUD8	4.98	yes	up	1.2E-05	yes	chr1	promoter	Intermediate	LCP	1.35	no	up	0.29	no	1.06	no	up	1.3E-07	yes
MSTRG.20352.9 : Ubp1 : D4A030	0.44	yes	down	2.3E-04	yes	chr8	intron	internal intron	NA	1.12	no	up	0.57	no	0.98	no	down	1.9E-01	no
MSTRG.17010.1 : Ttc27 : D3ZTG2	0.43	yes	down	8.1E-09	yes	chr6	exon	internal exon	NA	1.22	no	up	0.47	no	1.02	no	up	1.3E-01	no
MSTRG.9568.6 : Nedd4l : F1LRN8	2.03	yes	up	3.6E-05	yes	chr18	intron	internal intron	NA	1.01	no	up	0.48	no	0.95	no	down	3.9E-02	yes
MSTRG.16557.4 : Srrm1 : B2RYB3	2.05	yes	up	2.0E-04	yes	chr5	intron	internal intron	NA	0.49	yes	down	0.13	no	1.10	no	up	2.8E-06	yes
MSTRG.14242.2 : Cdk14 : D3ZSZ0	0.30	yes	down	1.6E-17	yes	chr4	intron	internal intron	NA	1.45	no	up	0.20	no	1.02	no	up	4.9E-01	no
ENSRNOT00000088245 : P2rx1 : B7U2F3	5.09	yes	up	1.5E-10	yes	chr10	promoter	Intermediate	LCP	3.72	yes	up	0.03	yes	0.82	yes	down	1.7E-12	yes
MSTRG.19819.3 : Neo1 : P97603	0	yes	down	3.5E-04	yes	chr8	promoter	Intermediate	LCP	1.00	no	down	0.99	no	0.98	no	down	1.8E-02	yes
MSTRG.15096.6 : Cacna1c : F1MA84	4.75	yes	up	4.3E-04	yes	chr4	intron	internal intron	NA	0.35	yes	down	0.06	no	0.97	no	down	2.7E-02	yes
MSTRG.12184.3 : Pcnt : D3ZMY8	0.14	yes	down	8.5E-07	yes	chr20	promoter	Distal	ICP	1.08	no	up	0.89	no	0.84	no	down	9.2E-06	yes
MSTRG.1362.1 : Rsf1 : D3ZGQ8	2.47	yes	up	3.7E-29	yes	chr1	exon	last exon	NA	1.04	no	up	0.49	no	0.95	no	down	6.9E-05	yes
MSTRG.3645.1 : Slfn13 : A0A096MKD0	2.27	yes	up	1.5E-04	yes	chr10	promoter	Intermediate	LCP	0.64	no	down	0.41	no	0.97	no	down	2.7E-02	yes
MSTRG.6137.1 : Gorab : B1H222	0.41	yes	down	1.4E-04	yes	chr13	intron	first intron	NA	0.79	no	down	0.45	no	1.08	no	up	1.7E-02	yes
MSTRG.4375.2 : Tbcd : F1M1D5	10.07	yes	up	4.7E-25	yes	chr10	intron	internal intron	NA	0.99	no	down	0.97	no	0.94	no	down	2.3E-10	yes
ENSRNOT00000041140 : Acaca : P11497	0.15	yes	down	3.0E-04	yes	chr10	intron	internal intron	NA	1.24	no	up	0.27	no	0.97	no	down	3.1E-04	yes
ENSRNOT00000075193 : Dock4 : M0R6K4	0.43	yes	down	2.9E-04	yes	chr6	exon	last exon	NA	1.00	no	down	0.90	no	0.94	no	down	8.4E-03	yes
MSTRG.8192.2 : Tenm3 : F1LV44	3.57	yes	up	5.2E-08	yes	chr16	exon	internal exon	NA	1.35	no	up	0.47	no	1.03	no	up	3.0E-03	yes
MSTRG.13195.13 : Arhgap1 : D4A6C5	3.73	yes	up	1.8E-11	yes	chr3	intron	internal intron	NA	0.71	no	down	0.45	no	1.04	no	up	1.7E-04	yes
MSTRG.16557.1 : Srrm1 : B2RYB3	2.05	yes	up	2.0E-04	yes	chr5	intron	internal intron	NA	0.88	no	down	0.64	no	1.10	no	up	2.8E-06	yes
ENSRNOT00000079680 : Tbl1x : B2RZA6	0	yes	down	6.0E-05	yes	chrX	intron	internal intron	NA	0.91	no	down	0.73	no	1.12	no	up	7.8E-09	yes
MSTRG.20817.5 : Map4k4 : A0A0G2K7W4	0.36	yes	down	7.1E-05	yes	chr9	intron	internal intron	NA	0.97	no	down	0.93	no	1.10	no	up	7.1E-06	yes
MSTRG.19219.6 : Fkbp11 : G3V7V5	inf	yes	up	4.7E-06	yes	chr7	intron	internal intron	NA	0.53	no	down	0.38	no	1.06	no	up	1.6E-07	yes
MSTRG.4716.6 : Adcy5 : G3V9G1	0.16	yes	down	2.3E-10	yes	chr11	intron	internal intron	NA	2.40	yes	up	0.09	no	1.00	no	down	7.6E-01	no
ENSRNOT00000019409 : Nr3c1 : P06536	0.27	yes	down	1.2E-04	yes	chr18	exon	first exon	NA	1.00	no	down	0.40	no	0.91	no	down	1.6E-10	yes
ENSRNOT00000008827 : Akap8 : Q63014	0.08	yes	down	2.7E-04	yes	chr7	intron	internal intron	NA	0.95	no	down	0.80	no	0.99	no	down	2.8E-01	no
MSTRG.3136.1 : Anxa6 : Q6IMZ3	2.83	yes	up	1.2E-05	yes	chr10	intron	internal intron	NA	0.93	no	down	0.25	no	0.84	no	down	6.6E-16	yes
ENSRNOT00000030037 : Arap1 : F1LM60	2.71	yes	up	3.3E-04	yes	chr1	intron	first intron	NA	1.08	no	up	0.73	no	0.99	no	down	2.5E-02	yes
ENSRNOT00000016060 : Ndufb3 : D4A4P3	0.10	yes	down	1.1E-16	yes	chr9	intron	first intron	NA	1.55	no	up	0.14	no	1.01	no	up	2.1E-01	no
MSTRG.9868.2 : Cc2d1a : Q66HA5	0.21	yes	down	1.7E-05	yes	chr19	promoter	Distal	LCP	0.84	no	down	0.57	no	0.96	no	down	4.1E-02	yes
MSTRG.2161.1 : Map4k2 : D3ZXB1	4.89	yes	up	3.4E-06	yes	chr1	intron	internal intron	NA	0.74	no	down	0.25	no	0.65	yes	down	2.4E-14	yes
ENSRNOT00000040069 : Eln : A0A0G2JST5	3.55	yes	up	3.0E-04	yes	chr12	intron	internal intron	NA	0.67	no	down	0.24	no	0.91	no	down	1.2E-01	no
ENSRNOT00000080522 : Stat1 : F1M9D6	0.50	yes	down	1.5E-10	yes	chr9	intron	first intron	NA	0.97	no	down	0.73	no	1.10	no	up	1.4E-13	yes
ENSRNOT00000017417 : Epb41l2 : D3ZM69	0.25	yes	down	1.1E-12	yes	chr1	intron	internal intron	NA	0.94	no	down	0.33	no	0.91	no	down	8.9E-11	yes
MSTRG.21008.2 : Myl1 : P02600	2.29	yes	up	4.3E-05	yes	chr9	promoter	Distal	LCP	0.99	no	down	0.97	no	1.33	yes	up	6.1E-13	yes
ENSRNOT00000093070 : Rrm1 : Q5U2Q5	8.63	yes	up	2.6E-22	yes	chr1	intron	last intron	NA	1.01	no	up	0.68	no	1.04	no	up	2.0E-04	yes
ENSRNOT00000086524 : Plscr1 : A0A0G2K7Q1	0.33	yes	down	1.3E-05	yes	chr8	promoter	Distal	LCP	1.00	no	down	0.06	no	1.08	no	up	5.2E-06	yes
ENSRNOT00000081450 : Tln2 : D3ZA84	3.91	yes	up	1.0E-11	yes	chr8	intron	internal intron	NA	0.85	no	down	0.40	no	0.87	no	down	7.8E-17	yes
MSTRG.21083.6 : Speg : Q63638	0.17	yes	down	2.6E-06	yes	chr9	intron	internal intron	NA	0.75	no	down	0.47	no	0.88	no	down	2.3E-07	yes
MSTRG.7505.2 : Rabggta : Q08602	0.13	yes	down	1.1E-04	yes	chr15	exon	internal exon	NA	0.83	no	down	0.14	no	0.95	no	down	1.4E-05	yes
MSTRG.9425.1 : Nr3c1 : P06536	0.27	yes	down	1.2E-04	yes	chr18	exon	first exon	NA	1.43	no	up	0.02	yes	0.91	no	down	1.6E-10	yes
MSTRG.7350.4 : Ktn1 : D4A4Z9	2.49	yes	up	1.2E-04	yes	chr15	intron	internal intron	NA	0.92	no	down	0.67	no	1.04	no	up	2.2E-11	yes
ENSRNOT00000078222 : D2hgdh : P84850	0.09	yes	down	4.1E-04	yes	chr9	exon	first exon	NA	1.22	no	up	0.20	no	0.95	no	down	7.7E-05	yes
MSTRG.2839.9 : Srrm2 : A0A0G2K2M9	1.72	yes	up	9.0E-05	yes	chr10	exon	internal exon	NA	1.36	no	up	0.24	no	1.00	no	up	9.0E-01	no
MSTRG.18557.11 : Mon2 : D3ZCG3	0.07	yes	down	1.5E-08	yes	chr7	intron	internal intron	NA	0.66	no	down	0.56	no	1.05	no	up	7.1E-07	yes
ENSRNOT00000000651 : Dnah8 : F1MAM6	5.44	yes	up	2.2E-09	yes	chr20	intron	internal intron	NA	0.99	no	down	0.21	no	0.94	no	down	6.6E-04	yes
ENSRNOT00000024465 : Btaf1 : A0A0G2K1N4	2.34	yes	up	6.1E-05	yes	chr1	intron	internal intron	NA	0.54	no	down	0.55	no	1.00	no	down	9.8E-01	no
MSTRG.18880.2 : Plec : Q6S395	0.33	yes	down	4.2E-06	yes	chr7	exon	last exon	NA	1.39	no	up	0.52	no	1.03	no	up	2.2E-01	no
ENSRNOT00000090925 : Itpr3 : C7E1V1	5.60	yes	up	1.8E-04	yes	chr20	intron	internal intron	NA	0.82	no	down	0.16	no	0.95	no	down	1.5E-04	yes
ENSRNOT00000024885 : Tln2 : D3ZA84	3.91	yes	up	1.0E-11	yes	chr8	intron	internal intron	NA	1.04	no	up	0.75	no	0.87	no	down	7.8E-17	yes
ENSRNOT00000023446 : Pter : Q63530	5.96	yes	up	4.9E-04	yes	chr17	promoter	Distal	LCP	0.98	no	down	0.95	no	1.15	no	up	4.7E-10	yes
MSTRG.3136.2 : Anxa6 : Q6IMZ3	2.83	yes	up	1.2E-05	yes	chr10	intron	internal intron	NA	0.91	no	down	0.94	no	0.84	no	down	6.6E-16	yes
MSTRG.16748.3 : Tardbp : I6L9G6	2.86	yes	up	4.5E-10	yes	chr5	intron	internal intron	NA	1.16	no	up	0.61	no	1.05	no	up	5.8E-11	yes
MSTRG.1326.2 : Picalm : Q66SY1	8.74	yes	up	2.1E-13	yes	chr1	intron	internal intron	NA	1.00	no	down	0.99	no	1.13	no	up	1.1E-06	yes
MSTRG.18043.3 : Stat2 : Q5XI26	0.06	yes	down	1.0E-04	yes	chr7	promoter	Distal	LCP	1.52	no	up	0.34	no	1.14	no	up	1.9E-09	yes
MSTRG.20698.2 : Tram2 : F1LUA2	3	yes	up	2.1E-05	yes	chr9	intron	first intron	NA	0.64	no	down	0.45	no	1.02	no	up	1.2E-01	no
MSTRG.6191.6 : Ddr2 : B1WC09	0.49	yes	down	3.3E-05	yes	chr13	exon	internal exon	NA	1.22	no	up	0.78	no	0.95	no	down	3.2E-01	no
ENSRNOT00000086550 : Plec : Q6S3A0	0.33	yes	down	4.2E-06	yes	chr7	exon	last exon	NA	1.00	no	down	0.70	no	1.11	no	up	6.0E-08	yes
MSTRG.4725.7 : Kalrn : P97924	1.54	yes	up	9.1E-07	yes	chr11	exon	last exon	NA	0.96	no	down	0.40	no	1.04	no	up	1.6E-04	yes
ENSRNOT00000037094 : Tgfbrap1 : D3ZXT8	0.12	yes	down	2.5E-04	yes	chr9	intron	internal intron	NA	1.57	no	up	0.48	no	0.98	no	down	2.9E-01	no
ENSRNOT00000027478 : Akt1s1 : D3ZH75	2.93	yes	up	2.4E-05	yes	chr1	intron	internal intron	NA	1.35	no	up	0.37	no	0.97	no	down	7.2E-01	no
ENSRNOT00000014583 : Mycbp2 : D4A2D3	0.31	yes	down	9.2E-06	yes	chr15	intron	internal intron	NA	1.00	no	down	0.84	no	1.00	no	down	6.8E-01	no
ENSRNOT00000051917 : Hist2h2ac : D4ACV3	0.18	yes	down	9.0E-08	yes	chr2	promoter	Intermediate	ICP	1.13	no	up	0.51	no	0.65	yes	down	8.6E-07	yes

MSTRG.21202.3 : Lrrfip1 : Q66HF9	6.49	yes	up	4.9E-10	yes	chr9	exon	last exon	NA	0.98	no	down	0.97	no	1.15	no	up	2.1E-11	yes
ENSRNOT00000000106 : Gpr89b : F1LTD0	0.21	yes	down	2.8E-04	yes	chr2	intron	internal intron	NA	0.98	no	down	0.88	no	0.93	no	down	2.3E-02	yes
ENSRNOT00000041910 : Fmn1 : D4A7C2	4.93	yes	up	1.3E-26	yes	chr3	intron	internal intron	NA	1.02	no	up	0.80	no	1.33	yes	up	4.7E-13	yes
MSTRG.18268.16 : Akap8 : Q63014	0.08	yes	down	2.7E-04	yes	chr7	intron	internal intron	NA	0.93	no	down	0.66	no	0.99	no	down	2.8E-01	no
MSTRG.21170.1 : Gigyf2 : A0A096MKC0	0.59	yes	down	1.9E-05	yes	chr9	intron	internal intron	NA	0.88	no	down	0.38	no	1.01	no	up	7.0E-01	no
ENSRNOT00000093424 : Eftud2 : F1LM66	4.66	yes	up	6.0E-04	yes	chr10	exon	internal exon	NA	0.94	no	down	0.54	no	1.02	no	up	4.9E-02	yes
MSTRG.12973.8 : Rbms1 : A0A0G2K4R7	0	yes	down	3.3E-05	yes	chr3	intron	internal intron	NA	0.82	no	down	0.43	no	0.97	no	down	1.2E-01	no
MSTRG.350.1 : Thbs2 : D4A2G6	1.43	yes	up	1.4E-19	yes	chr1	intron	internal intron	NA	0.93	no	down	0.68	no	1.17	no	up	2.2E-05	yes
MSTRG.8278.31 : Rbpms : F2Z3S5	0.02	yes	down	2.6E-05	yes	chr16	promoter	Proximal	ICP	0.79	no	down	0.05	yes	1.06	no	up	4.9E-05	yes
MSTRG.18268.24 : Akap8 : Q63014	0.08	yes	down	2.7E-04	yes	chr7	intron	internal intron	NA	0.93	no	down	0.89	no	0.99	no	down	2.8E-01	no
MSTRG.12896.8 : Rabgap1 : D3ZX42	3.82	yes	up	8.1E-06	yes	chr3	intron	internal intron	NA	1.73	no	up	0.09	no	1.01	no	up	1.9E-01	no
ENSRNOT00000019004 : Alpl : P08289	0.52	yes	down	2.4E-04	yes	chr5	promoter	Intermediate	LCP	0.98	no	down	0.98	no	1.00	no	down	6.2E-01	no
MSTRG.14948.1 : Magi1 : M0R8T1	0.38	yes	down	2.0E-04	yes	chr4	intron	internal intron	NA	0.98	no	down	0.76	no	0.99	no	down	6.0E-01	no
ENSRNOT00000048218 : Pcnt : D3ZMY8	0.14	yes	down	8.5E-07	yes	chr20	promoter	Distal	ICP	1.00	no	down	0.49	no	0.84	no	down	9.2E-06	yes
ENSRNOT00000067150 : Gmpr2 : A0A0G2JX25	0.33	yes	down	2.2E-06	yes	chr15	promoter	Distal	LCP	0.97	no	down	0.44	no	0.95	no	down	1.0E-01	no
MSTRG.8517.2 : Naa35 : Q6DKG0	inf	yes	up	1.7E-04	yes	chr17	promoter	Proximal	LCP	1.61	no	up	0.20	no	1.04	no	up	3.3E-02	yes
MSTRG.3511.3 : Cpd : A0A0G2JY30	0.15	yes	down	4.3E-04	yes	chr10	intron	internal intron	NA	1.63	no	up	0.48	no	1.13	no	up	1.1E-08	yes
MSTRG.8325.3 : Hook3 : Q7TQ77	0.22	yes	down	2.1E-04	yes	chr16	intron	internal intron	NA	1.00	no	down	0.94	no	1.01	no	up	5.0E-03	yes
MSTRG.14995.1 : Itpr1 : A0A0A0MY31	3.16	yes	up	1.1E-05	yes	chr4	intron	internal intron	NA	0.57	no	down	0.58	no	0.87	no	down	1.1E-14	yes
MSTRG.4991.2 : Ppm1f : Q9WVR7	1.80	yes	up	3.8E-11	yes	chr11	intron	internal intron	NA	1.14	no	up	0.55	no	0.91	no	down	2.5E-06	yes
MSTRG.11953.26 : RT1-CE7 : D3ZLE6	inf	yes	up	7.3E-07	yes	chr20	exon	internal exon	NA	0.82	no	down	0.40	no	1.22	yes	up	7.3E-16	yes
MSTRG.11405.8 : Pde4dip : A0A0G2JW66	6.94	yes	up	5.0E-08	yes	chr2	exon	internal exon	NA	0.63	no	down	0.31	no	1.06	no	up	5.1E-04	yes
MSTRG.7199.1 : Kcnma1 : A0A0G2K104	4.06	yes	up	2.6E-13	yes	chr15	intron	internal intron	NA	1.07	no	up	0.57	no	0.89	no	down	7.4E-06	yes
MSTRG.11265.4 : Ubap2l : E9PTR4	6.62	yes	up	4.0E-04	yes	chr2	intron	internal intron	NA	1.01	no	up	0.98	no	1.13	no	up	3.8E-12	yes
MSTRG.6480.7 : Evi5 : D3ZJN9	4.04	yes	up	4.0E-04	yes	chr14	intron	internal intron	NA	1.58	no	up	0.09	no	0.98	no	down	2.5E-01	no
ENSRNOT00000064007 : Rbm28 : D4A5K7	7.60	yes	up	4.6E-06	yes	chr4	intron	internal intron	NA	1.13	no	up	0.21	no	1.18	no	up	6.9E-05	yes
MSTRG.14711.2 : Mat2a : F1LRB8	0.10	yes	down	1.4E-04	yes	chr4	exon	last exon	NA	1.30	no	up	0.11	no	0.99	no	down	1.5E-01	no
MSTRG.17733.3 : Atg2b : Q5EBA2	0.42	yes	down	4.5E-04	yes	chr6	exon	internal exon	NA	1.57	no	up	0.44	no	1.03	no	up	1.2E-01	no
MSTRG.10799.2 : Gyg1 : F8WFR6	0.52	yes	down	3.8E-05	yes	chr2	intron	internal intron	NA	2.47	yes	up	0.13	no	1.17	no	up	1.9E-09	yes
ENSRNOT00000027774 : Pafah1b3 : O35263	4.32	yes	up	3.8E-07	yes	chr1	promoter	Distal	LCP	1.06	no	up	0.57	no	0.99	no	down	2.6E-01	no
MSTRG.19764.4 : Pstpip1 : B0BNK4	0.47	yes	down	3.6E-05	yes	chr8	intron	internal intron	NA	0.85	no	down	0.36	no	1.20	yes	up	1.1E-06	yes
MSTRG.6213.1 : Fcgr2b : A3RLA8	0.44	yes	down	2.8E-04	yes	chr13	promoter	Distal	LCP	1.03	no	up	0.69	no	1.04	no	up	4.3E-03	yes
ENSRNOT00000001242 : Stx2 : Q7TS57	4.45	yes	up	6.0E-04	yes	chr12	exon	last exon	NA	1.39	no	up	0.41	no	0.91	no	down	2.6E-04	yes
MSTRG.11223.4 : Fdps : F1LND7	inf	yes	up	2.2E-05	yes	chr2	exon	last exon	NA	0.83	no	down	0.39	no	1.07	no	up	1.2E-03	yes
ENSRNOT00000080013 : Eml4 : A0A0G2K2Z0	0.79	yes	down	2.3E-04	yes	chr6	exon	internal exon	NA	0.96	no	down	0.82	no	1.03	no	up	6.8E-04	yes
ENSRNOT00000078670 : Smndc1 : Q4QQU6	inf	yes	up	1.3E-04	yes	chr1	promoter	Distal	LCP	1.39	no	up	0.39	no	1.06	no	up	2.2E-04	yes
ENSRNOT00000001415 : Elavl1 : B5DF91	0.21	yes	down	5.3E-09	yes	chr12	intron	internal intron	NA	1.02	no	up	0.84	no	1.05	no	up	2.2E-07	yes
ENSRNOT00000077530 : Eogt : Q5NDL0	6.71	yes	up	1.7E-04	yes	chr4	intron	internal intron	NA	0.81	no	down	0.76	no	1.10	no	up	2.0E-05	yes
MSTRG.659.7 : Cic : D4A853	4.32	yes	up	3.8E-07	yes	chr1	exon	internal exon	NA	0.96	no	down	0.86	no	1.06	no	up	4.3E-02	yes
MSTRG.4335.5 : Pcyt2 : O88637	4.82	yes	up	3.0E-05	yes	chr10	intron	last intron	NA	0.67	no	down	0.04	yes	0.94	no	down	1.8E-03	yes
MSTRG.7470.4 : Acin1 : E9PST5	0.30	yes	down	1.1E-04	yes	chr15	promoter	Distal	LCP	1.44	no	up	0.38	no	0.98	no	down	5.1E-04	yes
MSTRG.12910.2 : Golga1 : D4A6K4	0.04	yes	down	1.1E-04	yes	chr3	intron	internal intron	NA	1.27	no	up	0.20	no	1.12	no	up	8.2E-05	yes
MSTRG.4727.2 : Kalrn : P97924	1.54	yes	up	9.1E-07	yes	chr11	exon	last exon	NA	0.95	no	down	0.95	no	1.04	no	up	1.6E-04	yes
MSTRG.2366.3 : Pgm5 : D3ZVR9	2.44	yes	up	4.5E-04	yes	chr1	intron	last intron	NA	0.76	no	down	0.60	no	1.01	no	up	3.2E-01	no
ENSRNOT00000084670 : Abr : A0A0G2JZZ7	inf	yes	up	3.7E-04	yes	chr10	intron	internal intron	NA	1.05	no	up	0.34	no	1.05	no	up	1.0E-03	yes
ENSRNOT00000078249 : Oxsm : G3V6R7	7.88	yes	up	2.7E-09	yes	chr15	exon	last exon	NA	1.35	no	up	0.19	no	1.01	no	up	5.6E-01	no
ENSRNOT00000007484 : Derl1 : F7FNS3	0.48	yes	down	3.9E-05	yes	chr7	exon	last exon	NA	0.88	no	down	0.80	no	1.04	no	up	2.4E-03	yes
MSTRG.16461.5 : Pum1 : D3Z8L5	0.44	yes	down	3.1E-04	yes	chr5	intron	internal intron	NA	1.74	no	up	0.01	yes	1.03	no	up	7.0E-02	no
MSTRG.6624.7 : Ankrd17 : D4A0B4	3.39	yes	up	8.9E-05	yes	chr14	intron	internal intron	NA	0.96	no	down	0.40	no	1.11	no	up	1.6E-06	yes
MSTRG.549.1 : Zc3h4 : D3ZVW3	2.19	yes	up	2.6E-04	yes	chr1	intron	internal intron	NA	1.42	no	up	0.25	no	0.98	no	down	3.2E-01	no
MSTRG.3600.2 : Nf1 : F1LM28	2.12	yes	up	4.3E-05	yes	chr10	intron	internal intron	NA	0.16	yes	down	0.23	no	0.98	no	down	1.9E-01	no
MSTRG.6327.1 : Acbd3 : G3V6E4	2.42	yes	up	1.4E-04	yes	chr13	intron	internal intron	NA	1.29	no	up	0.32	no	1.07	no	up	2.4E-14	yes
MSTRG.5906.2 : Atp2b4 : Q64542	2.31	yes	up	1.3E-05	yes	chr13	intron	internal intron	NA	1.38	no	up	0.42	no	0.68	yes	down	2.6E-19	yes
MSTRG.20352.5 : Ubp1 : D4A030	0.44	yes	down	2.3E-04	yes	chr8	intron	internal intron	NA	0.89	no	down	0.47	no	0.98	no	down	1.9E-01	no
ENSRNOT00000067228 : Ddost : Q641Y0	2.98	yes	up	4.3E-04	yes	chr5	promoter	Distal	ICP	1.12	no	up	0.45	no	1.04	no	up	7.4E-04	yes
ENSRNOT00000080421 : Morf4l1 : Q6AYU1	0.22	yes	down	4.4E-05	yes	chr8	intron	internal intron	NA	1.04	no	up	0.45	no	1.07	no	up	1.5E-07	yes
ENSRNOT00000061630 : Evi5 : D3ZJN9	4.04	yes	up	4.0E-04	yes	chr14	intron	internal intron	NA	0.48	yes	down	0.31	no	0.98	no	down	2.5E-01	no
ENSRNOT00000035129 : Dennd1b : F1M3B0	0.50	yes	down	5.1E-04	yes	chr13	promoter	Distal	HCP	0.97	no	down	0.45	no	0.99	no	down	8.0E-01	no
MSTRG.1035.3 : Rasip1 : B5DF05	6.95	yes	up	1.3E-05	yes	chr1	intron	first intron	NA	0.76	no	down	0.63	no	0.96	no	down	1.3E-02	yes
MSTRG.18752.1 : Tatdn1 : M0R6T1	0.49	yes	down	2.8E-04	yes	chr7	promoter	Distal	LCP	0.91	no	down	0.57	no	0.91	no	down	4.5E-09	yes
MSTRG.2527.3 : Pi4k2a : Q99M64	0.48	yes	down	2.9E-06	yes	chr1	intron	internal intron	NA	0.81	no	down	0.30	no	1.02	no	up	1.6E-01	no
ENSRNOT00000039410 : Scai : F1M3P6	0.24	yes	down	1.2E-04	yes	chr3	intron	internal intron	NA	0.95	no	down	0.61	no	0.86	no	down	1.0E-11	yes
MSTRG.16756.1 : Kif1b : A0A0G2KA12	3.65	yes	up	7.4E-05	yes	chr5	intron	internal intron	NA	0.79	no	down	0.26	no	1.02	no	up	6.5E-02	no
MSTRG.6248.2 : Ncstn : Q8CGU6	0.30	yes	down	6.8E-05	yes	chr13	intron	first intron	NA	0.83	no	down	0.64	no	1.02	no	up	4.0E-02	yes
MSTRG.4725.16 : Kalrn : P97924	1.54	yes	up	9.1E-07	yes	chr11	exon	last exon	NA	1.10	no	up	0.52	no	1.04	no	up	1.6E-04	yes
ENSRNOT00000005148 : Gorab : B1H222	0.41	yes	down	1.4E-04	yes	chr13	intron	first intron	NA	1.60	no	up	0.26	no	1.08	no	up	1.7E-02	yes
ENSRNOT00000090267 : Aamp : B0K024	0.09	yes	down	2.6E-04	yes	chr9	promoter	Intermediate	LCP	0.94	no	down	0.01	yes	1.06	no	up	1.5E-05	yes
ENSRNOT00000020608 : Rufy3 : A0A0G2K6A9	2.37	yes	up	3.9E-04	yes	chr14	promoter	Distal	LCP	1.02	no	up	0.47	no	1.11	no	up	1.3E-05	yes
ENSRNOT00000012005 : Mpp5 : B4F7E7	0.37	yes	down	1.0E-04	yes	chr6	exon	internal exon	NA	1.14	no	up	0.12	no	0.99	no	down	5.9E-01	no
ENSRNOT00000085987 : Fyn : Q62844	0.38	yes	down	2.4E-05	yes	chr20	exon	last exon	NA	1.05	no	up	0.76	no	1.01	no	up	5.2E-01	no
ENSRNOT00000084268 : Picalm : A0A1B0GWW9	8.74	yes	up	2.1E-13	yes	chr1	intron	internal intron	NA	0.50	yes	down	0.04	yes	1.04	no	up	1.0E-01	no
MSTRG.15546.6 : Asph : A0A096MKE0	2.56	yes	up	9.8E-06	yes	chr5	intron	internal intron	NA	0.77	no	down	0.09	no	0.99	no	down	1.3E-01	no
ENSRNOT00000020634 : Dip2c : D3ZZB0	0.67	yes	down	4.5E-04	yes	chr17	intron	internal intron	NA	2.31	yes	up	0.19	no	0.88	no	down	3.3E-04	yes
ENSRNOT00000089966 : Bclaf1 : B1WC16	0.67	yes	down	1.3E-07	yes	chr1	intron	internal intron	NA	1.00	no	down	0.07	no	1.03	no	up	6.8E-02	no
MSTRG.17520.4 : Srsf5 : Q09167	0.10	yes	down	3.0E-09	yes	chr6	promoter	Proximal	ICP	2.27	yes	up	0.06	no	1.03	no	up	4.2E-02	yes
MSTRG.8278.32 : Rbpms : F2Z3S5	0.02	yes	down	2.6E-05	yes	chr16	promoter	Proximal	ICP	1.00	no	down	0.09	no	1.06	no	up	4.9E-05	yes
MSTRG.986.3 : Akt1s1 : D3ZH75	2.93	yes	up	2.4E-05	yes	chr1	intron	internal intron	NA	1.13	no	up	0.30	no	0.97	no	down	7.2E-01	no
MSTRG.21176.10 : Inpp5d : F1M981	0.08	yes	down	9.9E-05	yes	chr9	intron	internal intron	NA	0.95	no	down	0.75	no	1.08	no	up	3.1E-05	yes
ENSRNOT00000004673 : Acsf2 : Q499N5	0.31	yes	down	5.4E-07	yes	chr10	intron	internal intron	NA	1.09	no	up	0.82	no	0.87	no	down	2.4E-13	yes
ENSRNOT00000064588 : Golim4 : D3ZM57	0.34	yes	down	3.2E-04	yes	chr2	intron	internal intron	NA	1.17	no	up	0.48	no	0.55	yes	down	1.1E-01	no
MSTRG.7470.19 : Acin1 : E9PST5	0.30	yes	down	1.1E-04	yes	chr15	promoter	Distal	LCP	1.00	no	down	0.99	no	0.98	no	down	5.1E-04	yes
MSTRG.3379.3 : Dlg4 : P31016	2.15	yes	up	8.6E-07	yes	chr10	intron	last intron	NA	1.45	no	up	0.27	no	0.97	no	down	9.1E-02	no
ENSRNOT00000018043 : Tm9sf3 : D3ZUD8	4.98	yes	up	1.2E-05	yes	chr1	promoter	Intermediate	LCP	1.13	no	up	0.85	no	1.06	no	up	1.3E-07	yes
ENSRNOT00000084527 : Postn : A0A097BW25	0.29	yes	down	1.4E-05	yes	chr2	intron	internal intron	NA	1.95	no	up	0.04	yes	1.23	yes	up	1.4E-16	yes
ENSRNOT00000026458 : Wars2 : F1M8H2	0.03	yes	down	1.4E-06	yes	chr2	promoter	Proximal	ICP	1.05	no	up	0.68	no	0.90	no	down	1.3E-05	yes
MSTRG.6575.1 : Fras1 : F1M3H3	2.21	yes	up	1.3E-04	yes	chr14	intron	internal intron	NA	1.29	no	up	0.36	no	0.81	yes	down	2.2E-05	yes
MSTRG.18268.7 : Akap8 : Q63014	0.08	yes	down	2.7E-04	yes	chr7	intron	internal intron	NA	0.78	no	down	0.36	no	0.99	no	down	2.8E-01	no
ENSRNOT00000016288 : Supt16h : D4A4J0	3.93	yes	up	5.1E-06	yes	chr15	intron	internal intron	NA	1.05	no	up	0.71	no	1.01	no	up	1.6E-03	yes
MSTRG.18948.1 : Tst : P24329	0.37	yes	down	4.2E-05	yes	chr7	intron	last intron	NA	0.70	no	down	0.23	no	0.77	yes	down	1.8E-17	yes
ENSRNOT00000049437 : Ctnna3 : F1M4I1	0	yes	down	1.3E-06	yes	chr20	promoter	Distal	LCP	0.92	no	down	0.55	no	0.92	no	down	3.6E-04	yes
MSTRG.10153.6 : Cmip : A0A0G2JYB9	0.58	yes	down	9.9E-05	yes	chr19	intron	internal intron	NA	1.61	no	up	0.48	no	0.89	no	down	2.1E-04	yes
MSTRG.6568.5 : Antxr2 : Q00IM8	1.10	no	up	9.8E-05	yes	chr14	intron	internal intron	NA	0.42	yes	down	0.45	no	1.01	no	up	3.8E-01	no
ENSRNOT00000085463 : Skp1 : Q6PEC4	0.41	yes	down	2.1E-04	yes	chr10	exon	last exon	NA	0.99	no	down	0.95	no	0.93	no	down	2.8E-11	yes
MSTRG.18072.4 : Dgka : P51556	0.72	yes	down	1.5E-04	yes	chr7	exon	internal exon	NA	1.12	no	up	0.64	no	0.95	no	down	8.8E-04	yes
MSTRG.256.6 : Syne1 : Q8VHJ9	0.04	yes	down	1.5E-19	yes	chr1	exon	first exon	NA	0.77	no	down	0.59	no	0.89	no	down	8.9E-14	yes
MSTRG.17673.2 : Ppp4r3a : D4ABC4	3.37	yes	up	4.5E-04	yes	chr6	intron	internal intron	NA	0.74	no	down	0.46	no	1.03	no	up	5.4E-03	yes
MSTRG.12277.2 : Ctnna3 : B2RYN9	0	yes	down	1.3E-06	yes	chr20	promoter	Distal	LCP	1.37	no	up	0.48	no	1.03	no	up	1.7E-01	no
MSTRG.5467.7 : Stx2 : Q7TS57	4.45	yes	up	6.0E-04	yes	chr12	exon	last exon	NA	1.00	no	down	0.98	no	0.91	no	down	2.6E-04	yes
ENSRNOT00000082116 : RT1-S3 : Q9R0V1	0.16	yes	down	4.2E-07	yes	chr20	exon	last exon	NA	1.14	no	up	0.48	no	1.05	no	up	2.0E-01	no
MSTRG.18880.3 : Plec : Q6S3A0	0.33	yes	down	4.2E-06	yes	chr7	exon	last exon	NA	1.02	no	up	0.96	no	1.11	no	up	6.0E-08	yes

MSTRG.1974.1 : Nap1l4 : Q5U2Z3	0.36	yes	down	7.2E-05	yes	chr1	intron	internal intron	NA	0.57	no	down	0.00	yes	0.98	no	down	3.1E-02	yes
MSTRG.22089.3 : Htatsf1 : D4A997	6.87	yes	up	2.7E-04	yes	chrX	promoter	Intermediate	HCP	0.85	no	down	0.47	no	0.95	no	down	9.0E-07	yes
ENSRNOT00000075922 : Fyn : Q62844	0.38	yes	down	2.4E-05	yes	chr20	exon	last exon	NA	0.97	no	down	0.76	no	1.01	no	up	5.2E-01	no
ENSRNOT00000027050 : Ube2f : Q5U203	7.18	yes	up	7.2E-05	yes	chr9	intron	internal intron	NA	0.90	no	down	0.41	no	1.01	no	up	5.6E-01	no
MSTRG.10104.1 : Sf3b3 : E9PT66	0.34	yes	down	3.6E-04	yes	chr19	exon	last exon	NA	1.08	no	up	0.89	no	0.97	no	down	3.1E-07	yes
ENSRNOT00000027868 : Becn1 : Q91XJ1	0.15	yes	down	1.2E-04	yes	chr10	exon	first exon	NA	0.88	no	down	0.55	no	0.97	no	down	9.2E-02	no
MSTRG.19042.4 : Aco2 : Q9ER34	0	yes	down	2.0E-04	yes	chr7	promoter	Distal	ICP	0.71	no	down	0.43	no	0.90	no	down	5.3E-13	yes
MSTRG.4972.2 : Klhl22 : D3ZZC3	2.85	yes	up	3.5E-06	yes	chr11	intron	internal intron	NA	0.34	yes	down	0.04	yes	0.98	no	down	1.1E-01	no
MSTRG.13265.5 : Tcp11l1 : F1M9Y7	3.13	yes	up	1.2E-06	yes	chr3	intron	last intron	NA	1.03	no	up	0.65	no	0.99	no	down	8.5E-01	no
ENSRNOT00000089082 : Palmd : Q4KM62	0.16	yes	down	7.2E-05	yes	chr2	promoter	Distal	LCP	0.97	no	down	0.25	no	0.77	yes	down	4.3E-06	yes
ENSRNOT00000071590 : Apoo : M0R7G4	22.85	yes	up	2.9E-05	yes	chrX	intron	first intron	NA	1.38	no	up	0.25	no	0.95	no	down	2.3E-02	yes
MSTRG.3895.4 : Cdk12 : A0A0G2K5U7	5.94	yes	up	1.1E-04	yes	chr10	exon	first exon	NA	1.28	no	up	0.14	no	0.97	no	down	5.5E-04	yes
ENSRNOT00000064070 : Ank1 : D3Z9Z0	1.38	yes	up	4.3E-04	yes	chr16	intron	internal intron	NA	1.00	no	down	0.90	no	0.90	no	down	2.2E-09	yes
ENSRNOT00000085727 : Myo1e : Q63356	3.15	yes	up	9.4E-05	yes	chr8	intron	first intron	NA	1.00	no	down	0.48	no	1.03	no	up	1.7E-02	yes
ENSRNOT00000001360 : Tsc22d1 : P62501	3.00	yes	up	3.0E-04	yes	chr15	promoter	Intermediate	ICP	5.81	yes	up	0.24	no	0.77	yes	down	5.9E-09	yes
MSTRG.19528.2 : Opcml : F1M2I5	2.41	yes	up	1.6E-04	yes	chr8	intron	last intron	NA	1.64	no	up	0.38	no	0.91	no	down	2.1E-15	yes
MSTRG.2839.8 : Srrm2 : A0A0G2K2M9	1.72	yes	up	9.0E-05	yes	chr10	exon	internal exon	NA	1.28	no	up	0.73	no	1.00	no	up	9.0E-01	no
MSTRG.8278.44 : Rbpms : F2Z3S5	0.02	yes	down	2.6E-05	yes	chr16	promoter	Proximal	ICP	0.88	no	down	0.33	no	1.06	no	up	4.9E-05	yes
MSTRG.5958.1 : Csrp1 : P47875	1.98	yes	up	3.9E-04	yes	chr13	promoter	Intermediate	LCP	0.13	yes	down	0.26	no	1.11	no	up	3.1E-08	yes
ENSRNOT00000077372 : Gsta3 : P04904	2.74	yes	up	2.3E-04	yes	chr9	exon	last exon	NA	1.01	no	up	0.84	no	0.64	yes	down	2.9E-15	yes
ENSRNOT00000051496 : Map3k5 : D3ZW27	0.78	yes	down	2.6E-04	yes	chr1	exon	internal exon	NA	1.43	no	up	0.05	yes	1.15	no	up	1.2E-09	yes
MSTRG.10104.4 : Sf3b3 : E9PT66	0.34	yes	down	3.6E-04	yes	chr19	exon	last exon	NA	0.81	no	down	0.45	no	0.97	no	down	3.1E-07	yes
MSTRG.16048.3 : Fggy : Q5FVC3	18.59	yes	up	3.3E-04	yes	chr5	intron	internal intron	NA	1.26	no	up	0.13	no	0.84	no	down	2.3E-07	yes
MSTRG.4276.1 : Timp2 : P30121	3.20	yes	up	1.5E-04	yes	chr10	exon	first exon	NA	3.47	yes	up	0.08	no	1.01	no	up	5.4E-01	no
MSTRG.16748.2 : Tardbp : I6L9G6	2.86	yes	up	4.5E-10	yes	chr5	intron	internal intron	NA	1.01	no	up	0.96	no	1.05	no	up	5.8E-11	yes
ENSRNOT00000014660 : Terf2ip : Q5EAN7	6.13	yes	up	7.3E-09	yes	chr19	intron	first intron	NA	1.25	no	up	0.78	no	1.01	no	up	4.8E-01	no
ENSRNOT00000016987 : Fam89b : Q566R4	inf	yes	up	8.3E-05	yes	chr1	promoter	Distal	LCP	0.79	no	down	0.72	no	0.95	no	down	1.3E-01	no
ENSRNOT00000076044 : Prdm16 : M0RDL0	0.37	yes	down	2.7E-04	yes	chr5	exon	internal exon	NA	1.16	no	up	0.63	no	0.83	yes	down	1.1E-06	yes
ENSRNOT00000044452 : Dst : D3ZC56	0.35	yes	down	1.3E-05	yes	chr9	intron	internal intron	NA	1.57	no	up	0.17	no	1.03	no	up	7.3E-04	yes
ENSRNOT00000013901 : Xpo4 : D3ZQI6	5.75	yes	up	1.3E-06	yes	chr15	intron	internal intron	NA	1.09	no	up	0.24	no	0.88	no	down	7.2E-03	yes
MSTRG.15855.3 : RGD1306148 : F1M446	0.22	yes	down	4.0E-05	yes	chr5	intron	internal intron	NA	1.00	no	down	0.77	no	1.01	no	up	7.4E-02	no
MSTRG.15110.1 : Wnk1 : Q9JIH7	18.77	yes	up	1.6E-38	yes	chr4	exon	internal exon	NA	0.86	no	down	0.50	no	1.04	no	up	1.7E-03	yes
MSTRG.11265.32 : Ubap2l : E9PTR4	6.62	yes	up	4.0E-04	yes	chr2	intron	internal intron	NA	0.56	no	down	0.18	no	1.13	no	up	3.8E-12	yes
MSTRG.5341.1 : Serpine1 : F1LM16	2.28	yes	up	2.6E-04	yes	chr12	exon	last exon	NA	1.21	no	up	0.47	no	1.00	no	down	7.7E-01	no
MSTRG.21938.4 : Klhl13 : F1LM44	0.22	yes	down	7.1E-07	yes	chrX	intron	last intron	NA	0.63	no	down	0.28	no	1.03	no	up	6.8E-03	yes
MSTRG.5439.2 : Gusb : F1LQQ8	29.04	yes	up	6.5E-05	yes	chr12	intron	internal intron	NA	1.01	no	up	0.97	no	0.97	no	down	1.8E-03	yes
ENSRNOT00000080352 : Plg : Q01177	7.88	yes	up	1.6E-04	yes	chr1	intron	internal intron	NA	1.00	no	down	0.09	no	1.05	no	up	7.8E-06	yes
MSTRG.16606.7 : Alpl : P08289	0.52	yes	down	2.4E-04	yes	chr5	promoter	Intermediate	LCP	1.36	no	up	0.09	no	1.00	no	down	6.2E-01	no
MSTRG.8915.1 : Rala : P63322	0.27	yes	down	4.5E-04	yes	chr17	promoter	Intermediate	LCP	0.90	no	down	0.24	no	0.88	no	down	5.2E-09	yes
MSTRG.19220.6 : Kmt2d : A0A0G2JVD6	6.53	yes	up	2.1E-09	yes	chr7	exon	last exon	NA	0.78	no	down	0.76	no	1.03	no	up	3.5E-02	yes
ENSRNOT00000085301 : Papss1 : A0A0G2K0L0	15.84	yes	up	7.3E-05	yes	chr2	promoter	Intermediate	LCP	0.89	no	down	0.82	no	1.04	no	up	5.2E-04	yes
MSTRG.4117.3 : Nol11 : A0A0G2K1A9	0.21	yes	down	9.6E-05	yes	chr10	intron	internal intron	NA	1.49	no	up	0.40	no	1.06	no	up	1.9E-03	yes
MSTRG.21144.6 : Cab39 : A0A0G2JZH0	39.86	yes	up	1.2E-27	yes	chr9	promoter	Intermediate	LCP	3.69	yes	up	0.03	yes	0.99	no	down	5.6E-01	no
ENSRNOT00000032865 : Lyplal1 : D3ZFS7	inf	yes	up	4.0E-04	yes	chr13	exon	first exon	NA	0.94	no	down	0.77	no	1.08	no	up	2.3E-02	yes
MSTRG.21202.1 : Lrrfip1 : Q66HF9	6.49	yes	up	4.9E-10	yes	chr9	exon	last exon	NA	0.89	no	down	0.83	no	1.15	no	up	2.1E-11	yes
ENSRNOT00000090942 : Yipf6 : A0A096MJG6	inf	yes	up	3.8E-06	yes	chrX	intron	first intron	NA	1.01	no	up	0.70	no	1.03	no	up	1.9E-01	no
ENSRNOT00000093189 : Ndrg1 : Q6JE36	0.37	yes	down	7.6E-09	yes	chr7	intron	internal intron	NA	0.94	no	down	0.34	no	0.92	no	down	4.9E-08	yes
MSTRG.4725.5 : Kalrn : P97924	1.54	yes	up	9.1E-07	yes	chr11	exon	last exon	NA	0.91	no	down	0.83	no	1.04	no	up	1.6E-04	yes
ENSRNOT00000026767 : Egln1 : A0A140TAD9	0.32	yes	down	1.1E-04	yes	chr19	exon	last exon	NA	0.88	no	down	0.36	no	1.28	yes	up	2.1E-09	yes
ENSRNOT00000040954 : Gp1bb : Q9JJM7	0	yes	down	2.2E-04	yes	chr11	intron	internal intron	NA	1.00	no	down	0.50	no	0.93	no	down	7.7E-08	yes
MSTRG.8345.8 : Fgfr1 : F1LM54	0.22	yes	down	3.0E-05	yes	chr16	exon	last exon	NA	0.71	no	down	0.29	no	1.05	no	up	1.3E-02	yes
MSTRG.19472.3 : Dnm2 : A0A0A0MY48	0.11	yes	down	8.9E-11	yes	chr8	intron	first intron	NA	1.17	no	up	0.44	no	1.05	no	up	2.0E-08	yes
MSTRG.19194.5 : Hdac7 : A0A0G2K6B1	1.57	yes	up	6.8E-05	yes	chr7	intron	internal intron	NA	0.84	no	down	0.34	no	0.99	no	down	6.7E-01	no
MSTRG.15231.5 : Tspan9 : D4AAV9	0.41	yes	down	1.4E-09	yes	chr4	exon	last exon	NA	1.11	no	up	0.85	no	0.96	no	down	2.4E-05	yes
MSTRG.8514.1 : Zcchc6 : D3ZKR9	0.14	yes	down	1.2E-08	yes	chr17	exon	internal exon	NA	0.81	no	down	0.36	no	1.12	no	up	2.3E-06	yes
MSTRG.11265.9 : Ubap2l : E9PTR4	6.62	yes	up	4.0E-04	yes	chr2	intron	internal intron	NA	1.16	no	up	0.47	no	1.13	no	up	3.8E-12	yes
ENSRNOT00000091574 : Apoe : P02650	0.23	yes	down	3.2E-04	yes	chr1	exon	last exon	NA	1.02	no	up	0.93	no	0.86	no	down	6.2E-12	yes
MSTRG.6480.5 : Evi5 : D3ZJN9	4.04	yes	up	4.0E-04	yes	chr14	intron	internal intron	NA	1.65	no	up	0.49	no	0.98	no	down	2.5E-01	no
ENSRNOT00000017190 : Gucy1a3 : P19686	0.26	yes	down	3.9E-06	yes	chr2	intron	internal intron	NA	2.02	yes	up	0.18	no	0.85	no	down	1.4E-12	yes
ENSRNOT00000048455 : Rabggta : Q08602	0.13	yes	down	1.1E-04	yes	chr15	exon	internal exon	NA	1.69	no	up	0.18	no	0.95	no	down	1.4E-05	yes
ENSRNOT00000060607 : C2 : Q6MG73	2.91	yes	up	9.4E-08	yes	chr20	intron	internal intron	NA	0.96	no	down	0.93	no	1.20	yes	up	2.9E-12	yes
MSTRG.6572.1 : Bmp2k : Q3SYQ0	11.81	yes	up	5.6E-07	yes	chr14	intron	internal intron	NA	1.04	no	up	0.49	no	1.19	no	up	6.4E-09	yes
ENSRNOT00000010826 : Wbp2 : G3V721	4.13	yes	up	1.7E-04	yes	chr10	promoter	Intermediate	LCP	0.99	no	down	0.87	no	0.89	no	down	6.1E-15	yes
ENSRNOT00000017813 : Nab1 : Q62722	3.78	yes	up	5.6E-07	yes	chr9	exon	internal exon	NA	1.14	no	up	0.23	no	0.97	no	down	6.0E-01	no
MSTRG.12277.2 : Ctnna3 : F1M4I1	0	yes	down	1.3E-06	yes	chr20	promoter	Distal	LCP	1.37	no	up	0.48	no	0.92	no	down	3.6E-04	yes
MSTRG.15469.1 : Tram1 : Q5XI41	3.29	yes	up	2.9E-05	yes	chr5	exon	last exon	NA	2.41	yes	up	0.00	yes	1.13	no	up	1.2E-09	yes
MSTRG.4335.7 : Pcyt2 : O88637	4.82	yes	up	3.0E-05	yes	chr10	intron	last intron	NA	0.58	no	down	0.11	no	0.94	no	down	1.8E-03	yes
MSTRG.256.4 : Syne1 : Q8VHJ9	0.04	yes	down	1.5E-19	yes	chr1	exon	first exon	NA	0.23	yes	down	0.23	no	0.89	no	down	8.9E-14	yes
ENSRNOT00000071020 : Rbm14 : M0R9Q1	0.55	yes	down	9.3E-08	yes	chr1	intron	first intron	NA	1.00	no	down	0.83	no	1.03	no	up	3.8E-06	yes
MSTRG.12945.3 : Fmnl2 : A0A0G2K132	0.84	no	down	6.1E-07	yes	chr3	intron	internal intron	NA	1.21	no	up	0.24	no	1.00	no	down	9.6E-01	no
ENSRNOT00000076337 : Dync1li2 : Q5D023	0.17	yes	down	2.9E-04	yes	chr19	intron	last intron	NA	1.02	no	up	0.48	no	0.99	no	down	1.1E-01	no
MSTRG.10602.1 : Nnt : Q5BJZ3	3.00	yes	up	1.8E-04	yes	chr2	intron	internal intron	NA	0.84	no	down	0.59	no	0.96	no	down	1.2E-07	yes
MSTRG.14960.7 : Eogt : Q5NDL0	6.71	yes	up	1.7E-04	yes	chr4	intron	internal intron	NA	0.96	no	down	0.91	no	1.10	no	up	2.0E-05	yes
ENSRNOT00000083531 : Thbs4 : F1LMS5	0.15	yes	down	4.0E-04	yes	chr2	intron	internal intron	NA	2.15	yes	up	0.10	no	1.25	yes	up	3.7E-10	yes
ENSRNOT00000064588 : Golim4 : Q5BJK8	0.34	yes	down	3.2E-04	yes	chr2	intron	internal intron	NA	1.17	no	up	0.48	no	1.06	no	up	1.6E-03	yes
ENSRNOT00000089895 : Ano6 : A0A0G2K1M7	3.87	yes	up	9.8E-09	yes	chr7	intron	internal intron	NA	1.06	no	up	0.52	no	0.91	no	down	2.1E-09	yes
MSTRG.1675.2 : Arhgap17 : D4AAV2	0.83	yes	down	2.6E-07	yes	chr1	intron	internal intron	NA	1.10	no	up	0.41	no	1.01	no	up	3.1E-01	no
MSTRG.5034.2 : Insr : F1LPL6	0.36	yes	down	9.6E-05	yes	chr12	intron	internal intron	NA	0.20	yes	down	0.00	yes	1.09	no	up	1.4E-04	yes
ENSRNOT00000000627 : Srsf3 : A0A0U1RRV7	4.40	yes	up	1.5E-08	yes	chr20	intron	internal intron	NA	0.68	no	down	0.18	no	0.94	no	down	5.5E-08	yes
ENSRNOT00000078841 : Tpd52l1 : Q499Q2	2.69	yes	up	6.5E-28	yes	chr1	promoter	Distal	LCP	1.00	no	down	0.43	no	0.97	no	down	5.0E-01	no
ENSRNOT00000091213 : Agr2 : D3ZIA7	0.34	yes	down	2.0E-08	yes	chr6	intron	first intron	NA	1.00	no	down	0.94	no	0.85	no	down	2.5E-12	yes
MSTRG.19619.1 : C1qtnf5 : Q5FVH0	0.32	yes	down	9.8E-07	yes	chr8	exon	last exon	NA	1.06	no	up	0.80	no	0.96	no	down	2.5E-05	yes
MSTRG.4871.3 : Ephb3 : D3ZH39	1.63	yes	up	6.2E-06	yes	chr11	intron	internal intron	NA	1.00	no	down	0.97	no	0.86	no	down	1.2E-07	yes
ENSRNOT00000023459 : Rnf214 : D4A3V4	0.59	yes	down	3.7E-05	yes	chr8	intron	internal intron	NA	1.44	no	up	0.31	no	1.00	no	down	9.9E-01	no
MSTRG.20218.3 : Rpl29 : P25886	3.86	yes	up	3.0E-04	yes	chr8	intron	first intron	NA	0.49	yes	down	0.56	no	0.98	no	down	6.0E-01	no
MSTRG.11268.1 : Tpm3 : A0A140TAF0	0.37	yes	down	8.0E-05	yes	chr2	intron	internal intron	NA	0.85	no	down	0.26	no	1.03	no	up	4.4E-04	yes
ENSRNOT00000016351 : Xrn2 : D4A914	0.32	yes	down	3.4E-05	yes	chr3	intron	internal intron	NA	1.21	no	up	0.17	no	1.03	no	up	8.8E-06	yes
ENSRNOT00000076353 : Dync1li2 : Q5D023	0.17	yes	down	2.9E-04	yes	chr19	intron	last intron	NA	1.03	no	up	0.30	no	0.99	no	down	1.1E-01	no
ENSRNOT00000087176 : Fkbp11 : G3V7V5	inf	yes	up	4.7E-06	yes	chr7	intron	internal intron	NA	0.98	no	down	0.82	no	1.06	no	up	1.6E-07	yes
MSTRG.21202.2 : Lrrfip1 : Q66HF9	6.49	yes	up	4.9E-10	yes	chr9	exon	last exon	NA	1.04	no	up	0.92	no	1.15	no	up	2.1E-11	yes
ENSRNOT00000093143 : Eftud2 : F1LM66	4.66	yes	up	6.0E-04	yes	chr10	exon	internal exon	NA	0.88	no	down	0.14	no	1.02	no	up	4.9E-02	yes
MSTRG.20352.11 : Ubp1 : D4A030	0.44	yes	down	2.3E-04	yes	chr8	intron	internal intron	NA	1.18	no	up	0.06	no	0.98	no	down	1.9E-01	no
MSTRG.13195.7 : Arhgap1 : D4A6C5	3.73	yes	up	1.8E-11	yes	chr3	intron	internal intron	NA	0.71	no	down	0.67	no	1.04	no	up	1.7E-04	yes
ENSRNOT00000009649 : Psmc6 : Q32PW9	2.30	yes	up	1.2E-05	yes	chr15	intron	internal intron	NA	0.99	no	down	0.81	no	1.02	no	up	3.7E-03	yes
MSTRG.10127.2 : Kars : Q5XIM7	16.06	yes	up	5.9E-09	yes	chr19	exon	last exon	NA	0.59	no	down	0.28	no	0.95	no	down	1.9E-07	yes
MSTRG.1283.3 : Tm6sf1 : D4A3E7	0.12	yes	down	9.3E-08	yes	chr1	intron	first intron	NA	0.91	no	down	0.50	no	1.05	no	up	5.5E-03	yes
MSTRG.18268.8 : Akap8 : Q63014	0.08	yes	down	2.7E-04	yes	chr7	intron	internal intron	NA	0.92	no	down	0.41	no	0.99	no	down	2.8E-01	no
MSTRG.12973.2 : Rbms1 : A0A0G2K4R7	0	yes	down	3.3E-05	yes	chr3	intron	internal intron	NA	1.09	no	up	0.81	no	0.97	no	down	1.2E-01	no
MSTRG.21039.7 : Tns1 : F1LN42	0.09	yes	down	2.0E-04	yes	chr9	intron	last intron	NA	0.63	no	down	0.26	no	0.86	no	down	7.8E-19	yes
MSTRG.19538.17 : Nfrkb : D4A421	0.58	yes	down	1.3E-05	yes	chr8	intron	internal intron	NA	1.83	no	up	0.09	no	0.89	no	down	2.5E-03	yes

ENSRNOT00000049536 : Stat2 : Q5XI26	0.06	yes	down	1.0E-04	yes	chr7	promoter	Distal	LCP	1.26	no	up	0.43	no	1.14	no	up	1.9E-09	yes
MSTRG.12973.7 : Rbms1 : A0A0G2K4R7	0	yes	down	3.3E-05	yes	chr3	intron	internal intron	NA	0.85	no	down	0.53	no	0.97	no	down	1.2E-01	no
ENSRNOT00000018535 : Cdh11 : F1MAH6	1.94	yes	up	2.2E-05	yes	chr19	exon	last exon	NA	2.28	yes	up	0.48	no	0.91	no	down	6.5E-11	yes
MSTRG.7350.5 : Ktn1 : D4A4Z9	2.49	yes	up	1.2E-04	yes	chr15	intron	internal intron	NA	0.95	no	down	0.93	no	1.04	no	up	2.2E-11	yes
ENSRNOT00000080174 : Ddx39b : Q63413	0.02	yes	down	4.7E-11	yes	chr20	exon	last exon	NA	0.62	no	down	0.48	no	1.01	no	up	2.0E-01	no
MSTRG.1229.2 : Anpep : G3V7W7	0.25	yes	down	1.9E-09	yes	chr1	intron	internal intron	NA	1.35	no	up	0.55	no	1.05	no	up	3.0E-08	yes
ENSRNOT00000003061 : Fryl : D3ZQY4	2.76	yes	up	1.7E-04	yes	chr14	intron	internal intron	NA	0.95	no	down	0.91	no	1.00	no	down	4.6E-01	no
MSTRG.8278.20 : Rbpms : F2Z3S5	0.02	yes	down	2.6E-05	yes	chr16	promoter	Proximal	ICP	1.19	no	up	0.36	no	1.06	no	up	4.9E-05	yes
MSTRG.5482.2 : Ncor2 : A0A0G2JU91	2.16	yes	up	1.6E-06	yes	chr12	intron	internal intron	NA	1.07	no	up	0.43	no	1.01	no	up	7.1E-01	no
MSTRG.4751.2 : Pcyt1a : P19836	0.29	yes	down	1.2E-05	yes	chr11	exon	first exon	NA	1.53	no	up	0.05	yes	0.94	no	down	3.3E-07	yes
MSTRG.13870.6 : Plcg1 : G3V845	0.24	yes	down	1.1E-04	yes	chr3	intron	first intron	NA	0.90	no	down	0.43	no	0.94	no	down	1.8E-09	yes
ENSRNOT00000065759 : Plin1 : F1LSF5	2.99	yes	up	9.0E-13	yes	chr1	intron	internal intron	NA	1.09	no	up	0.52	no	1.08	no	up	8.1E-05	yes
ENSRNOT00000025232 : Bud13 : G3V8F3	2.30	yes	up	8.0E-06	yes	chr8	promoter	Distal	LCP	0.92	no	down	0.69	no	1.02	no	up	2.3E-01	no
ENSRNOT00000017412 : Epb41l2 : D3ZM69	0.25	yes	down	1.1E-12	yes	chr1	intron	internal intron	NA	0.99	no	down	0.53	no	0.91	no	down	8.9E-11	yes
ENSRNOT00000086813 : Stk38l : A4GW50	0.61	yes	down	1.5E-06	yes	chr4	intron	internal intron	NA	1.55	no	up	0.17	no	1.16	no	up	1.1E-11	yes
ENSRNOT00000012545 : Clasp2 : A0A0G2JZM8	0.25	yes	down	1.3E-05	yes	chr8	intron	internal intron	NA	1.01	no	up	0.09	no	0.98	no	down	1.8E-01	no
ENSRNOT00000085501 : Fgfr1op : Q4V7C1	7.97	yes	up	2.8E-17	yes	chr1	intron	internal intron	NA	1.17	no	up	0.39	no	1.07	no	up	7.3E-04	yes
MSTRG.11888.3 : Ppp1r18 : A0A0G2K8B3	0.32	yes	down	2.0E-04	yes	chr20	promoter	Distal	LCP	1.58	no	up	0.49	no	1.10	no	up	1.1E-05	yes
MSTRG.18880.1 : Plec : Q6S395	0.33	yes	down	4.2E-06	yes	chr7	exon	last exon	NA	0.61	no	down	0.10	no	1.03	no	up	2.2E-01	no
ENSRNOT00000024737 : Hectd3 : F1LVZ9	3.28	yes	up	3.1E-04	yes	chr5	intron	internal intron	NA	1.07	no	up	0.81	no	0.99	no	down	6.3E-01	no
ENSRNOT00000060868 : Bmp2k : F1M7M4	11.81	yes	up	5.6E-07	yes	chr14	intron	internal intron	NA	0.91	no	down	0.28	no	1.09	no	up	3.4E-07	yes
MSTRG.3600.1 : Nf1 : F1LM28	2.12	yes	up	4.3E-05	yes	chr10	intron	internal intron	NA	0.17	yes	down	0.09	no	0.98	no	down	1.9E-01	no
MSTRG.13559.1 : Hspa12b : D3ZVM5	0.65	yes	down	3.5E-04	yes	chr3	intron	last intron	NA	0.97	no	down	0.90	no	0.86	no	down	5.9E-13	yes
MSTRG.7470.2 : Acin1 : E9PST5	0.30	yes	down	1.1E-04	yes	chr15	promoter	Distal	LCP	0.88	no	down	0.65	no	0.98	no	down	5.1E-04	yes
MSTRG.6624.5 : Ankrd17 : D4A0B4	3.39	yes	up	8.9E-05	yes	chr14	intron	internal intron	NA	2.67	yes	up	0.04	yes	1.11	no	up	1.6E-06	yes
MSTRG.15356.2 : Pde3a : Q62865	0.30	yes	down	3.1E-07	yes	chr4	intron	internal intron	NA	1.42	no	up	0.47	no	1.00	no	up	9.7E-01	no
MSTRG.16327.13 : Macf1 : A0A0G2K9T4	0.36	yes	down	1.3E-05	yes	chr5	exon	last exon	NA	0.42	yes	down	0.45	no	0.97	no	down	9.0E-11	yes
ENSRNOT00000092722 : Lamp1 : P14562	0.27	yes	down	5.9E-08	yes	chr16	promoter	Distal	LCP	1.01	no	up	0.93	no	1.10	no	up	5.4E-06	yes
ENSRNOT00000040808 : Tpm1 : Q923Z2	9.41	yes	up	5.1E-14	yes	chr8	intron	internal intron	NA	0.56	no	down	0.42	no	0.75	yes	down	6.0E-18	yes
MSTRG.15231.12 : Tspan9 : D4AAV9	0.41	yes	down	1.4E-09	yes	chr4	exon	last exon	NA	0.51	no	down	0.46	no	0.96	no	down	2.4E-05	yes
MSTRG.2881.3 : Pkd1 : Q9ERV0	inf	yes	up	6.6E-05	yes	chr10	intron	internal intron	NA	1.03	no	up	0.28	no	1.19	no	up	9.8E-07	yes
ENSRNOT00000019761 : Pigt : D4A604	29.04	yes	up	1.4E-06	yes	chr3	intron	internal intron	NA	0.96	no	down	0.59	no	1.02	no	up	5.9E-02	no
MSTRG.18995.2 : Gtpbp1 : D2XV59	2.18	yes	up	7.2E-06	yes	chr7	exon	last exon	NA	1.09	no	up	0.78	no	0.97	no	down	3.4E-03	yes
MSTRG.12174.3 : Col18a1 : F1LR02	0.22	yes	down	4.1E-11	yes	chr20	exon	internal exon	NA	1.20	no	up	0.75	no	1.03	no	up	3.6E-07	yes
ENSRNOT00000067636 : Vps41 : D3ZVH6	0.30	yes	down	1.3E-06	yes	chr17	intron	internal intron	NA	1.06	no	up	0.34	no	1.02	no	up	4.2E-02	yes
MSTRG.15089.3 : Csgalnact2 : D4A5Z0	2.74	yes	up	6.9E-07	yes	chr4	intron	internal intron	NA	0.86	no	down	0.45	no	0.94	no	down	1.2E-02	yes
MSTRG.8278.46 : Rbpms : F2Z3S5	0.02	yes	down	2.6E-05	yes	chr16	promoter	Proximal	ICP	0.92	no	down	0.67	no	1.06	no	up	4.9E-05	yes
MSTRG.7309.2 : Flnb : D4A8D5	1.70	yes	up	8.8E-05	yes	chr15	intron	last intron	NA	0.74	no	down	0.17	no	1.00	no	up	2.4E-01	no
ENSRNOT00000065636 : Htatsf1 : D4A997	6.87	yes	up	2.7E-04	yes	chrX	promoter	Intermediate	HCP	2.28	yes	up	0.02	yes	0.95	no	down	9.0E-07	yes
MSTRG.18072.8 : Dgka : P51556	0.72	yes	down	1.5E-04	yes	chr7	exon	internal exon	NA	1.46	no	up	0.19	no	0.95	no	down	8.8E-04	yes
MSTRG.15778.1 : Ncbp1 : Q56A27	0.31	yes	down	1.1E-07	yes	chr5	intron	internal intron	NA	0.99	no	down	0.97	no	1.00	no	up	9.4E-01	no
ENSRNOT00000018942 : Mccc1 : F1LP30	0.34	yes	down	1.3E-04	yes	chr2	intron	internal intron	NA	1.14	no	up	0.27	no	0.96	no	down	1.3E-08	yes
ENSRNOT00000003106 : Hnrnpdl : A0A0G2KAZ7	inf	yes	up	7.6E-05	yes	chr14	intron	first intron	NA	0.94	no	down	0.83	no	0.99	no	down	4.6E-01	no
MSTRG.20742.3 : Dst : D3ZC56	0.35	yes	down	1.3E-05	yes	chr9	intron	internal intron	NA	0.95	no	down	0.55	no	1.03	no	up	7.3E-04	yes
ENSRNOT00000016285 : Kdelc1 : B5DFA5	5.54	yes	up	1.1E-06	yes	chr9	exon	last exon	NA	1.21	no	up	0.36	no	0.94	no	down	8.4E-02	no
MSTRG.13167.1 : Nup160 : D3ZBL6	14.66	yes	up	4.1E-04	yes	chr3	intron	internal intron	NA	1.19	no	up	0.19	no	1.00	no	down	6.2E-01	no
MSTRG.20352.7 : Ubp1 : D4A030	0.44	yes	down	2.3E-04	yes	chr8	intron	internal intron	NA	1.71	no	up	0.08	no	0.98	no	down	1.9E-01	no
MSTRG.6624.9 : Ankrd17 : D4A0B4	3.39	yes	up	8.9E-05	yes	chr14	intron	internal intron	NA	0.65	no	down	0.61	no	1.11	no	up	1.6E-06	yes
ENSRNOT00000025845 : Lipa : Q64194	35.26	yes	up	5.1E-18	yes	chr1	intron	internal intron	NA	2.12	yes	up	0.20	no	1.02	no	up	6.9E-02	no
MSTRG.21926.3 : Pls3 : F1LPK7	0	yes	down	1.5E-04	yes	chrX	intron	internal intron	NA	1.36	no	up	0.75	no	1.11	no	up	9.8E-14	yes
MSTRG.6480.3 : Evi5 : D3ZJN9	4.04	yes	up	4.0E-04	yes	chr14	intron	internal intron	NA	1.45	no	up	0.74	no	0.98	no	down	2.5E-01	no
MSTRG.2839.4 : Srrm2 : A0A0G2K2M9	1.72	yes	up	9.0E-05	yes	chr10	exon	internal exon	NA	0.80	no	down	0.83	no	1.00	no	up	9.0E-01	no
MSTRG.12240.9 : Bicc1 : A0A0G2K0Y0	5.19	yes	up	5.0E-04	yes	chr20	intron	internal intron	NA	0.92	no	down	0.51	no	1.52	yes	up	1.2E-05	yes
MSTRG.18880.2 : Plec : Q6S3A0	0.33	yes	down	4.2E-06	yes	chr7	exon	last exon	NA	1.39	no	up	0.52	no	1.11	no	up	6.0E-08	yes
MSTRG.1443.5 : Arap1 : F1LM60	2.71	yes	up	3.3E-04	yes	chr1	intron	first intron	NA	1.33	no	up	0.28	no	0.99	no	down	2.5E-02	yes
MSTRG.19088.4 : Scube1 : F1M987	2.13	yes	up	1.3E-04	yes	chr7	intron	internal intron	NA	1.08	no	up	0.10	no	0.80	yes	down	1.2E-14	yes
MSTRG.10916.2 : Naa15 : D3ZD89	0.53	yes	down	3.9E-04	yes	chr2	exon	last exon	NA	0.99	no	down	0.00	yes	1.05	no	up	5.6E-06	yes
MSTRG.4992.4 : Top3b : D4A9Z2	11.41	yes	up	1.2E-04	yes	chr11	intron	last intron	NA	0.60	no	down	0.31	no	0.99	no	down	6.1E-01	no
MSTRG.8278.38 : Rbpms : F2Z3S5	0.02	yes	down	2.6E-05	yes	chr16	promoter	Proximal	ICP	0.96	no	down	0.82	no	1.06	no	up	4.9E-05	yes
ENSRNOT00000019703 : Golga1 : D4A6K4	0.04	yes	down	1.1E-04	yes	chr3	intron	internal intron	NA	1.18	no	up	0.06	no	1.12	no	up	8.2E-05	yes
MSTRG.11405.9 : Pde4dip : A0A0G2JW66	6.94	yes	up	5.0E-08	yes	chr2	exon	internal exon	NA	1.63	no	up	0.16	no	1.06	no	up	5.1E-04	yes
MSTRG.8018.2 : Use1 : B0BNG6	3.16	yes	up	2.8E-15	yes	chr16	intron	internal intron	NA	0.63	no	down	0.09	no	1.02	no	up	5.2E-02	no
ENSRNOT00000084571 : Syne1 : Q8VHJ9	0.04	yes	down	1.5E-19	yes	chr1	exon	first exon	NA	1.00	no	down	0.06	no	0.89	no	down	8.9E-14	yes
ENSRNOT00000046126 : Kars : Q5XIM7	16.06	yes	up	5.9E-09	yes	chr19	exon	last exon	NA	0.86	no	down	0.63	no	0.95	no	down	1.9E-07	yes
MSTRG.7470.6 : Acin1 : E9PST5	0.30	yes	down	1.1E-04	yes	chr15	promoter	Distal	LCP	1.22	no	up	0.28	no	0.98	no	down	5.1E-04	yes
ENSRNOT00000056430 : Fer : A0A140TAC4	3.31	yes	up	2.7E-04	yes	chr9	promoter	Proximal	LCP	0.98	no	down	0.48	no	0.92	no	down	7.3E-07	yes
ENSRNOT00000078532 : Klf6 : G3V880	14.58	yes	up	4.1E-04	yes	chr17	intron	internal intron	NA	0.91	no	down	0.69	no	0.81	yes	down	4.1E-06	yes
MSTRG.20753.1 : Ptpn18 : Q4KM54	0	yes	down	1.9E-04	yes	chr9	intron	first intron	NA	1.59	no	up	0.04	yes	1.20	no	up	1.2E-08	yes
MSTRG.3949.4 : Dhx58 : D3ZD46	5.00	yes	up	2.1E-04	yes	chr10	exon	internal exon	NA	1.11	no	up	0.72	no	1.22	yes	up	6.3E-08	yes
MSTRG.8278.42 : Rbpms : F2Z3S5	0.02	yes	down	2.6E-05	yes	chr16	promoter	Proximal	ICP	0.24	yes	down	0.05	yes	1.06	no	up	4.9E-05	yes
ENSRNOT00000093322 : Ndrg1 : Q6JE36	0.37	yes	down	7.6E-09	yes	chr7	intron	internal intron	NA	0.96	no	down	0.31	no	0.92	no	down	4.9E-08	yes
MSTRG.12945.4 : Fmnl2 : A0A0G2K132	0.84	no	down	6.1E-07	yes	chr3	intron	internal intron	NA	0.87	no	down	0.69	no	1.00	no	down	9.6E-01	no
MSTRG.19945.1 : Tpm1 : A0A0G2JSQ4	9.41	yes	up	5.1E-14	yes	chr8	intron	internal intron	NA	0.98	no	down	0.92	no	0.81	yes	down	2.5E-10	yes
MSTRG.20303.2 : Trex1 : Q5BK16	0.35	yes	down	1.1E-04	yes	chr8	promoter	Distal	LCP	0.81	no	down	0.68	no	1.21	yes	up	5.1E-11	yes
MSTRG.17263.3 : Ankmy2 : D3ZC34	2.16	yes	up	2.9E-04	yes	chr6	promoter	Distal	ICP	0.81	no	down	0.55	no	0.93	no	down	2.5E-09	yes
MSTRG.21023.5 : Fn1 : F1LST1	0.11	yes	down	7.7E-11	yes	chr9	intron	internal intron	NA	8.58	yes	up	0.48	no	1.27	yes	up	4.1E-20	yes
MSTRG.11265.29 : Ubap2l : E9PTR4	6.62	yes	up	4.0E-04	yes	chr2	intron	internal intron	NA	1.28	no	up	0.04	yes	1.13	no	up	3.8E-12	yes
MSTRG.19020.4 : Tnrc6b : A0A0G2K6R0	2.14	yes	up	4.3E-13	yes	chr7	exon	last exon	NA	0.42	yes	down	0.07	no	1.01	no	up	7.4E-01	no
ENSRNOT00000079767 : Hnrnpdl : A0A0G2KAZ7	inf	yes	up	7.6E-05	yes	chr14	intron	first intron	NA	0.99	no	down	0.93	no	0.99	no	down	4.6E-01	no
MSTRG.18208.2 : Atp5d : G3V7Y3	0.12	yes	down	5.2E-08	yes	chr7	promoter	Distal	LCP	1.04	no	up	0.08	no	0.90	no	down	7.4E-12	yes
ENSRNOT00000092762 : Sema4a : A0A1B0GWV9	3.09	yes	up	1.9E-04	yes	chr2	promoter	Intermediate	LCP	1.00	no	down	0.80	no	1.07	no	up	2.4E-05	yes
MSTRG.8278.28 : Rbpms : F2Z3S5	0.02	yes	down	2.6E-05	yes	chr16	promoter	Proximal	ICP	0.95	no	down	0.68	no	1.06	no	up	4.9E-05	yes
MSTRG.7780.3 : Lmo7 : A0A0G2K174	3.35	yes	up	1.2E-04	yes	chr15	intron	internal intron	NA	0.61	no	down	0.11	no	0.96	no	down	4.2E-06	yes
ENSRNOT00000008355 : Sf3a1 : D3ZQM0	2.34	yes	up	9.2E-07	yes	chr14	intron	internal intron	NA	1.16	no	up	0.18	no	1.04	no	up	2.6E-10	yes
ENSRNOT00000003696 : Pafah1b1 : P63004	0.19	yes	down	5.9E-04	yes	chr10	intron	internal intron	NA	0.99	no	down	0.97	no	0.96	no	down	1.4E-04	yes
MSTRG.14695.9 : Ptcd3 : D3ZGM1	3.88	yes	up	4.1E-35	yes	chr4	intron	internal intron	NA	1.10	no	up	0.84	no	0.98	no	down	3.1E-01	no
MSTRG.1283.2 : Tm6sf1 : D4A3E7	0.12	yes	down	9.3E-08	yes	chr1	intron	first intron	NA	1.00	no	down	0.48	no	1.05	no	up	5.5E-03	yes
ENSRNOT00000085355 : Rhoc : B2RYP0	0.09	yes	down	2.2E-12	yes	chr2	intron	first intron	NA	0.87	no	down	0.17	no	0.96	no	down	1.7E-03	yes
MSTRG.6094.4 : Serpinc1 : Q5M7T5	11.92	yes	up	5.6E-26	yes	chr13	intron	first intron	NA	1.34	no	up	0.11	no	0.89	no	down	1.2E-15	yes
ENSRNOT00000084470 : Ptpn18 : Q4KM54	0	yes	down	1.9E-04	yes	chr9	intron	first intron	NA	1.20	no	up	0.08	no	1.20	no	up	1.2E-08	yes
MSTRG.4012.4 : Ubtf : P25977	1.48	yes	up	1.5E-06	yes	chr10	intron	internal intron	NA	1.18	no	up	0.69	no	0.96	no	down	1.4E-06	yes
MSTRG.4890.4 : Dvl3 : D4ADV8	0.50	yes	down	1.6E-05	yes	chr11	intron	first intron	NA	1.16	no	up	0.57	no	0.98	no	down	2.0E-01	no
MSTRG.6192.2 : Uap1 : D3ZF39	0.41	yes	down	2.7E-06	yes	chr13	intron	internal intron	NA	1.08	no	up	0.82	no	1.13	no	up	4.6E-12	yes
MSTRG.13515.2 : Anapc1 : F1M801	0.25	yes	down	6.5E-11	yes	chr3	intron	internal intron	NA	0.44	yes	down	0.40	no	1.00	no	down	5.4E-01	no
ENSRNOT00000079239 : Tgfbrap1 : D3ZXT8	0.12	yes	down	2.5E-04	yes	chr9	intron	internal intron	NA	1.48	no	up	0.67	no	0.98	no	down	2.9E-01	no
ENSRNOT00000014158 : Elmsan1 : D4ACA6	0.27	yes	down	2.9E-04	yes	chr6	exon	last exon	NA	1.05	no	up	0.92	no	0.96	no	down	1.9E-01	no
MSTRG.16461.2 : Pum1 : D3Z8L5	0.44	yes	down	3.1E-04	yes	chr5	intron	internal intron	NA	0.72	no	down	0.24	no	1.03	no	up	7.0E-02	no
ENSRNOT00000046232 : Usp7 : F1LM09	inf	yes	up	5.0E-04	yes	chr10	intron	internal intron	NA	0.63	no	down	0.07	no	1.00	no	down	7.8E-01	no
ENSRNOT00000082341 : Trip10 : P97531	2.19	yes	up	4.1E-04	yes	chr9	intron	last intron	NA	1.08	no	up	0.74	no	0.91	no	down	4.8E-08	yes
MSTRG.21083.9 : Speg : Q63638	0.17	yes	down	2.6E-06	yes	chr9	intron	internal intron	NA	0.54	no	down	0.32	no	0.88	no	down	2.3E-07	yes

MSTRG.12240.10 : Bicc1 : A0A0G2K0Y0	5.19	yes	up	5.0E-04	yes	chr20	intron	internal intron	NA	1.01	no	up	0.93	no	1.52	yes	up	1.2E-05	yes
MSTRG.7470.14 : Acin1 : E9PST5	0.30	yes	down	1.1E-04	yes	chr15	promoter	Distal	LCP	1.22	no	up	0.66	no	0.98	no	down	5.1E-04	yes
MSTRG.14695.2 : Ptcd3 : D3ZGM1	3.88	yes	up	4.1E-35	yes	chr4	intron	internal intron	NA	1.05	no	up	0.88	no	0.98	no	down	3.1E-01	no
MSTRG.1443.10 : Arap1 : F1LM60	2.71	yes	up	3.3E-04	yes	chr1	intron	first intron	NA	1.69	no	up	0.06	no	0.99	no	down	2.5E-02	yes
MSTRG.18129.2 : Nfic : O70188	1.68	yes	up	1.4E-06	yes	chr7	intron	internal intron	NA	1.38	no	up	0.42	no	0.85	no	down	3.8E-12	yes
MSTRG.1333.3 : Dlg2 : F1M907	2.35	yes	up	1.4E-05	yes	chr1	intron	internal intron	NA	1.39	no	up	0.64	no	0.92	no	down	2.8E-06	yes
MSTRG.18046.2 : Pan2 : R9PXX6	2.14	yes	up	4.2E-04	yes	chr7	exon	internal exon	NA	1.24	no	up	0.65	no	0.84	no	down	1.2E-03	yes
MSTRG.13128.2 : Itgav : F1LZX9	24.74	yes	up	1.9E-17	yes	chr3	intron	first intron	NA	1.29	no	up	0.38	no	0.92	no	down	1.7E-15	yes
ENSRNOT00000078585 : Lsm14a : A0A0G2JUK2	0.31	yes	down	3.8E-04	yes	chr1	intron	last intron	NA	2.34	yes	up	0.23	no	1.02	no	up	2.8E-01	no
MSTRG.7835.1 : Tm9sf2 : Q66HG5	0.39	yes	down	9.3E-05	yes	chr15	intron	last intron	NA	1.02	no	up	0.94	no	1.03	no	up	6.1E-04	yes
MSTRG.8514.6 : Zcchc6 : D3ZKR9	0.14	yes	down	1.2E-08	yes	chr17	exon	internal exon	NA	1.15	no	up	0.32	no	1.12	no	up	2.3E-06	yes
ENSRNOT00000040762 : Plec : Q6S3A0	0.33	yes	down	4.2E-06	yes	chr7	exon	last exon	NA	1.05	no	up	0.87	no	1.11	no	up	6.0E-08	yes
ENSRNOT00000004290 : Timp2 : P30121	3.20	yes	up	1.5E-04	yes	chr10	exon	first exon	NA	1.25	no	up	0.14	no	1.01	no	up	5.4E-01	no
ENSRNOT00000025415 : Picalm : A0A1B0GWW9	8.74	yes	up	2.1E-13	yes	chr1	intron	internal intron	NA	0.69	no	down	0.22	no	1.04	no	up	1.0E-01	no
ENSRNOT00000088073 : Il4r : Q63257	9.80	yes	up	2.9E-08	yes	chr1	intron	internal intron	NA	1.00	no	down	0.83	no	1.11	no	up	2.1E-05	yes
MSTRG.20817.13 : Map4k4 : A0A0G2K7W4	0.36	yes	down	7.1E-05	yes	chr9	intron	internal intron	NA	0.74	no	down	0.56	no	1.10	no	up	7.1E-06	yes
MSTRG.14053.2 : Dido1 : D3ZWL9	2.83	yes	up	7.0E-07	yes	chr3	intron	internal intron	NA	0.67	no	down	0.51	no	1.01	no	up	2.0E-01	no
MSTRG.12668.2 : Nup188 : F1LRC6	0.22	yes	down	2.3E-05	yes	chr3	exon	internal exon	NA	1.42	no	up	0.31	no	1.02	no	up	1.9E-01	no
ENSRNOT00000083528 : Sptbn1 : A0A0G2K8W9	5.72	yes	up	4.0E-04	yes	chr14	intron	internal intron	NA	1.05	no	up	0.57	no	0.87	no	down	1.1E-18	yes
ENSRNOT00000026941 : Speg : Q63638	0.17	yes	down	2.6E-06	yes	chr9	intron	internal intron	NA	1.03	no	up	0.94	no	0.88	no	down	2.3E-07	yes
ENSRNOT00000013189 : Dido1 : D3ZWL9	2.83	yes	up	7.0E-07	yes	chr3	intron	internal intron	NA	0.96	no	down	0.20	no	1.01	no	up	2.0E-01	no
MSTRG.16897.2 : Atl2 : F1LQ09	2.32	yes	up	2.9E-04	yes	chr6	intron	last intron	NA	1.29	no	up	0.06	no	0.95	no	down	8.9E-05	yes
MSTRG.19305.1 : Krt8 : Q10758	0.19	yes	down	1.4E-04	yes	chr7	exon	last exon	NA	0.89	no	down	0.17	no	0.83	yes	down	4.5E-14	yes
ENSRNOT00000024068 : P2rx1 : B7U2F3	5.09	yes	up	1.5E-10	yes	chr10	promoter	Intermediate	LCP	0.81	no	down	0.48	no	0.82	yes	down	1.7E-12	yes
MSTRG.18557.6 : Mon2 : D3ZCG3	0.07	yes	down	1.5E-08	yes	chr7	intron	internal intron	NA	0.78	no	down	0.45	no	1.05	no	up	7.1E-07	yes
ENSRNOT00000076604 : Dync1li2 : Q5D023	0.17	yes	down	2.9E-04	yes	chr19	intron	last intron	NA	1.20	no	up	0.30	no	0.99	no	down	1.1E-01	no
ENSRNOT00000027045 : Thop1 : P24155	0.11	yes	down	2.2E-07	yes	chr7	exon	last exon	NA	1.02	no	up	0.82	no	0.92	no	down	9.5E-13	yes
MSTRG.2579.2 : Sfxn3 : Q6P6T0	0.39	yes	down	7.8E-05	yes	chr1	intron	internal intron	NA	0.37	yes	down	0.20	no	1.12	no	up	6.4E-07	yes
MSTRG.11223.3 : Fdps : F1LND7	inf	yes	up	2.2E-05	yes	chr2	exon	last exon	NA	0.68	no	down	0.08	no	1.07	no	up	1.2E-03	yes
ENSRNOT00000042103 : Fmn1 : D4A7C2	4.93	yes	up	1.3E-26	yes	chr3	intron	internal intron	NA	1.00	no	down	0.76	no	1.33	yes	up	4.7E-13	yes
MSTRG.20352.13 : Ubp1 : D4A030	0.44	yes	down	2.3E-04	yes	chr8	intron	internal intron	NA	1.08	no	up	0.57	no	0.98	no	down	1.9E-01	no
ENSRNOT00000050733 : Pds5b : D3ZU56	0.26	yes	down	2.1E-04	yes	chr12	promoter	Intermediate	LCP	1.02	no	up	0.05	yes	0.96	no	down	4.7E-06	yes
MSTRG.8948.1 : Mtpap : D3ZPN5	1.99	yes	up	1.4E-05	yes	chr17	intron	internal intron	NA	0.98	no	down	0.90	no	1.03	no	up	1.2E-01	no
MSTRG.16327.16 : Macf1 : A0A0G2K9T4	0.36	yes	down	1.3E-05	yes	chr5	exon	last exon	NA	0.73	no	down	0.81	no	0.97	no	down	9.0E-11	yes
MSTRG.22089.5 : Htatsf1 : D4A997	6.87	yes	up	2.7E-04	yes	chrX	promoter	Intermediate	HCP	0.91	no	down	0.68	no	0.95	no	down	9.0E-07	yes
MSTRG.8992.3 : Dip2c : D3ZZB0	0.67	yes	down	4.5E-04	yes	chr17	intron	internal intron	NA	0.90	no	down	0.70	no	0.88	no	down	3.3E-04	yes
MSTRG.20878.7 : Gls : P13264	0.05	yes	down	1.5E-13	yes	chr9	intron	first intron	NA	1.00	no	down	0.99	no	1.08	no	up	4.1E-02	yes
MSTRG.2768.2 : Usp7 : F1LM09	inf	yes	up	5.0E-04	yes	chr10	intron	internal intron	NA	0.96	no	down	0.96	no	1.00	no	down	7.8E-01	no
MSTRG.19194.2 : Hdac7 : A0A0G2K6B1	1.57	yes	up	6.8E-05	yes	chr7	intron	internal intron	NA	1.00	no	down	1.00	no	0.99	no	down	6.7E-01	no
ENSRNOT00000025711 : D2hgdh : P84850	0.09	yes	down	4.1E-04	yes	chr9	exon	first exon	NA	0.90	no	down	0.75	no	0.95	no	down	7.7E-05	yes
MSTRG.11091.5 : Gucy1a3 : P19686	0.26	yes	down	3.9E-06	yes	chr2	intron	internal intron	NA	0.69	no	down	0.54	no	0.85	no	down	1.4E-12	yes
MSTRG.21170.2 : Gigyf2 : A0A096MKC0	0.59	yes	down	1.9E-05	yes	chr9	intron	internal intron	NA	0.96	no	down	0.71	no	1.01	no	up	7.0E-01	no
ENSRNOT00000074829 : Pdia6 : Q63081	0.09	yes	down	1.3E-04	yes	chr6	intron	internal intron	NA	1.30	no	up	0.09	no	1.12	no	up	2.7E-12	yes
MSTRG.5906.5 : Atp2b4 : Q64542	2.31	yes	up	1.3E-05	yes	chr13	intron	internal intron	NA	1.10	no	up	0.50	no	0.68	yes	down	2.6E-19	yes
ENSRNOT00000075978 : Dync1li2 : Q5D023	0.17	yes	down	2.9E-04	yes	chr19	intron	last intron	NA	0.99	no	down	0.94	no	0.99	no	down	1.1E-01	no
ENSRNOT00000012725 : Tf : A0A0G2QC06	5.10	yes	up	5.5E-06	yes	chr8	intron	first intron	NA	1.07	no	up	0.37	no	1.08	no	up	1.0E-13	yes
MSTRG.5410.6 : Gtf2i : Q5U2Y1	5.31	yes	up	4.9E-06	yes	chr12	promoter	Distal	LCP	0.93	no	down	0.51	no	0.95	no	down	1.5E-07	yes
ENSRNOT00000088370 : Stim1 : P84903	2.61	yes	up	1.3E-04	yes	chr1	exon	last exon	NA	0.79	no	down	0.56	no	0.92	no	down	3.4E-08	yes
ENSRNOT00000001201 : Hsph1 : Q66HA8	inf	yes	up	3.0E-05	yes	chr12	intron	internal intron	NA	0.98	no	down	0.96	no	1.12	no	up	9.8E-13	yes
ENSRNOT00000067616 : Ece1 : Q6IN10	0.58	yes	down	9.3E-05	yes	chr5	intron	internal intron	NA	17.01	yes	up	0.11	no	1.14	no	up	8.3E-15	yes
MSTRG.1466.1 : Trim21 : D4ACF2	0.20	yes	down	5.7E-10	yes	chr1	promoter	Distal	LCP	1.24	no	up	0.32	no	0.96	no	down	1.3E-04	yes
ENSRNOT00000023605 : Tpm3 : Q63610	0.37	yes	down	8.0E-05	yes	chr2	intron	internal intron	NA	0.91	no	down	0.63	no	1.11	no	up	3.2E-12	yes
MSTRG.20035.1 : Filip1 : F1LM79	4.15	yes	up	2.1E-10	yes	chr8	exon	first exon	NA	0.91	no	down	0.61	no	1.01	no	up	3.0E-01	no
ENSRNOT00000093012 : Stim1 : P84903	2.61	yes	up	1.3E-04	yes	chr1	exon	last exon	NA	1.05	no	up	0.09	no	0.92	no	down	3.4E-08	yes
MSTRG.16517.4 : Slc9a1 : Q8R4H8	0.61	yes	down	7.2E-05	yes	chr5	intron	internal intron	NA	2.17	yes	up	0.08	no	1.05	no	up	2.8E-01	no
ENSRNOT00000008222 : Pawr : G3V6S1	4.33	yes	up	2.3E-06	yes	chr7	intron	last intron	NA	1.26	no	up	0.47	no	1.06	no	up	9.6E-07	yes
MSTRG.21039.5 : Tns1 : F1LN42	0.09	yes	down	2.0E-04	yes	chr9	intron	last intron	NA	1.04	no	up	0.95	no	0.86	no	down	7.8E-19	yes
MSTRG.19088.6 : Scube1 : F1M987	2.13	yes	up	1.3E-04	yes	chr7	intron	internal intron	NA	1.19	no	up	0.22	no	0.80	yes	down	1.2E-14	yes
ENSRNOT00000092870 : Gtf2i : Q5U2Y1	5.31	yes	up	4.9E-06	yes	chr12	promoter	Distal	LCP	1.06	no	up	0.75	no	0.95	no	down	1.5E-07	yes
ENSRNOT00000079508 : Wdr72 : A0A0G2K3H7	1.90	yes	up	4.4E-07	yes	chr8	promoter	Distal	LCP	0.98	no	down	0.19	no	1.14	no	up	5.6E-05	yes
MSTRG.8517.1 : Naa35 : Q6DKG0	inf	yes	up	1.7E-04	yes	chr17	promoter	Proximal	LCP	0.81	no	down	0.27	no	1.04	no	up	3.3E-02	yes
MSTRG.16771.2 : H6pd : D4A7D7	0.22	yes	down	2.6E-06	yes	chr5	intron	last intron	NA	0.65	no	down	0.40	no	1.14	no	up	9.6E-15	yes
ENSRNOT00000067018 : Acaa2 : G3V9U2	0.52	yes	down	2.2E-04	yes	chr18	exon	last exon	NA	0.82	no	down	0.15	no	1.00	no	up	7.4E-01	no
MSTRG.6635.1 : Rufy3 : A0A0G2K6A9	2.37	yes	up	3.9E-04	yes	chr14	promoter	Distal	LCP	0.85	no	down	0.67	no	1.11	no	up	1.3E-05	yes
MSTRG.3468.1 : Pafah1b1 : P63004	0.19	yes	down	5.9E-04	yes	chr10	intron	internal intron	NA	1.00	no	down	0.48	no	0.96	no	down	1.4E-04	yes
MSTRG.8345.11 : Fgfr1 : F1LM54	0.22	yes	down	3.0E-05	yes	chr16	exon	last exon	NA	0.69	no	down	0.25	no	1.05	no	up	1.3E-02	yes
ENSRNOT00000064288 : Lims1 : C0KUC5	12.34	yes	up	5.9E-05	yes	chr20	intron	internal intron	NA	1.20	no	up	0.33	no	0.77	yes	down	1.9E-12	yes
ENSRNOT00000019536 : Armc8 : B4F7A2	0.25	yes	down	7.8E-05	yes	chr8	exon	first exon	NA	1.05	no	up	0.73	no	0.97	no	down	2.3E-03	yes
MSTRG.17811.5 : Mark3 : F1M836	0.30	yes	down	2.6E-04	yes	chr6	exon	last exon	NA	1.03	no	up	0.66	no	1.10	no	up	4.1E-07	yes
MSTRG.7226.6 : Sec24c : A0A0G2JZF0	3.50	yes	up	1.3E-09	yes	chr15	intron	internal intron	NA	1.06	no	up	0.77	no	1.01	no	up	2.6E-01	no
ENSRNOT00000077261 : Slfn13 : A0A096MKD0	2.27	yes	up	1.5E-04	yes	chr10	promoter	Intermediate	LCP	1.42	no	up	0.13	no	0.97	no	down	2.7E-02	yes
MSTRG.13368.1 : Rtf1 : D3ZLH8	0.17	yes	down	2.6E-07	yes	chr3	intron	first intron	NA	0.89	no	down	0.11	no	1.04	no	up	2.7E-04	yes
MSTRG.19220.2 : Kmt2d : A0A0G2JVD6	6.53	yes	up	2.1E-09	yes	chr7	exon	last exon	NA	1.36	no	up	0.68	no	1.03	no	up	3.5E-02	yes
ENSRNOT00000048044 : Tpm1 : A0A0G2JSQ4	9.41	yes	up	5.1E-14	yes	chr8	intron	internal intron	NA	0.70	no	down	0.01	yes	0.81	yes	down	2.5E-10	yes
MSTRG.6992.1 : Sec14l4 : A0A0G2JWI8	0.38	yes	down	1.3E-04	yes	chr14	intron	internal intron	NA	0.91	no	down	0.53	no	0.91	no	down	1.1E-08	yes
MSTRG.13130.3 : Calcrl : Q63118	2.81	yes	up	1.2E-04	yes	chr3	intron	internal intron	NA	0.79	no	down	0.39	no	1.09	no	up	1.2E-04	yes
MSTRG.12710.1 : Nup214 : M0RBV9	0.13	yes	down	4.7E-04	yes	chr3	intron	internal intron	NA	1.02	no	up	0.85	no	1.01	no	up	5.4E-01	no
ENSRNOT00000089544 : Kmt2d : A0A0G2JVD6	6.53	yes	up	2.1E-09	yes	chr7	exon	last exon	NA	1.01	no	up	0.29	no	1.03	no	up	3.5E-02	yes
MSTRG.659.6 : Cic : D4A853	4.32	yes	up	3.8E-07	yes	chr1	exon	internal exon	NA	0.88	no	down	0.62	no	1.06	no	up	4.3E-02	yes
MSTRG.16640.3 : Arhgef10l : M0R7W2	2.87	yes	up	9.9E-07	yes	chr5	intron	internal intron	NA	0.82	no	down	0.22	no	1.13	no	up	8.4E-04	yes
ENSRNOT00000030996 : Nup188 : F1LRC6	0.22	yes	down	2.3E-05	yes	chr3	exon	internal exon	NA	0.78	no	down	0.41	no	1.02	no	up	1.9E-01	no
MSTRG.479.6 : Epn1 : O88339	2.09	yes	up	1.9E-04	yes	chr1	intron	internal intron	NA	0.83	no	down	0.61	no	0.99	no	down	3.7E-01	no
MSTRG.5280.5 : Taf6 : Q498R0	0.74	yes	down	2.2E-06	yes	chr12	intron	internal intron	NA	1.33	no	up	0.10	no	0.91	no	down	1.8E-03	yes
MSTRG.17520.7 : Srsf5 : Q09167	0.10	yes	down	3.0E-09	yes	chr6	promoter	Proximal	ICP	0.79	no	down	0.52	no	1.03	no	up	4.2E-02	yes
MSTRG.3249.6 : Specc1 : A0A0G2K5D7	5.53	yes	up	1.9E-10	yes	chr10	exon	last exon	NA	1.70	no	up	0.09	no	0.93	no	down	4.4E-09	yes
MSTRG.3349.1 : Senp3 : Q3MID8	0.16	yes	down	5.8E-06	yes	chr10	exon	internal exon	NA	1.07	no	up	0.47	no	0.90	no	down	1.2E-11	yes
MSTRG.18206.1 : Cirbp : P60825	0.73	yes	down	1.3E-04	yes	chr7	exon	first exon	NA	0.97	no	down	0.95	no	0.85	no	down	3.4E-16	yes
MSTRG.16327.8 : Macf1 : A0A0G2K9T4	0.36	yes	down	1.3E-05	yes	chr5	exon	last exon	NA	1.00	no	down	0.40	no	0.97	no	down	9.0E-11	yes
ENSRNOT00000000749 : Cisd1 : B0K020	15.21	yes	up	2.4E-04	yes	chr20	promoter	Distal	ICP	1.14	no	up	0.66	no	0.97	no	down	6.1E-02	no
ENSRNOT00000077289 : Kmt2c : A0A0G2K426	0.22	yes	down	1.1E-11	yes	chr4	promoter	Distal	LCP	0.99	no	down	0.78	no	0.69	yes	down	1.4E-11	yes
ENSRNOT00000041130 : Itpr1 : A0A0A0MY31	3.16	yes	up	1.1E-05	yes	chr4	intron	internal intron	NA	0.96	no	down	0.40	no	0.87	no	down	1.1E-14	yes
ENSRNOT00000074644 : Larp7 : M0R7D1	0.06	yes	down	3.4E-04	yes	chr2	exon	last exon	NA	1.23	no	up	0.82	no	1.07	no	up	2.0E-06	yes
ENSRNOT00000090572 : Dock9 : F1LSM8	0.10	yes	down	2.7E-05	yes	chr15	intron	internal intron	NA	1.11	no	up	0.26	no	0.97	no	down	2.3E-04	yes
MSTRG.5035.4 : Arhgef18 : F1LT94	0.62	yes	down	4.6E-05	yes	chr12	exon	internal exon	NA	0.98	no	down	0.96	no	0.93	no	down	5.9E-05	yes
ENSRNOT00000018003 : Hykk : D3ZUX1	25.31	yes	up	2.9E-04	yes	chr8	promoter	Distal	LCP	1.04	no	up	0.81	no	0.93	no	down	2.9E-02	yes
MSTRG.21176.6 : Inpp5d : F1M981	0.08	yes	down	9.9E-05	yes	chr9	intron	internal intron	NA	0.46	yes	down	0.02	yes	1.08	no	up	3.1E-05	yes
MSTRG.18368.5 : Nt5dc3 : D3ZAI6	1.55	yes	up	4.2E-07	yes	chr7	intron	internal intron	NA	0.96	no	down	0.45	no	1.05	no	up	2.5E-05	yes
ENSRNOT00000079077 : Atad2b : A0A096MKA5	3.51	yes	up	9.0E-07	yes	chr6	intron	internal intron	NA	1.46	no	up	0.06	no	1.15	no	up	9.1E-06	yes
MSTRG.18268.15 : Akap8 : Q63014	0.08	yes	down	2.7E-04	yes	chr7	intron	internal intron	NA	1.01	no	up	0.02	yes	0.99	no	down	2.8E-01	no
MSTRG.5035.2 : Arhgef18 : F1LT94	0.62	yes	down	4.6E-05	yes	chr12	exon	internal exon	NA	2.50	yes	up	0.08	no	0.93	no	down	5.9E-05	yes

MSTRG.19219.3 : Fkbp11 : G3V7V5	inf	yes	up	4.7E-06	yes	chr7	intron	internal intron	NA	0.86	no	down	0.61	no	1.06	no	up	1.6E-07	yes
MSTRG.2839.5 : Srrm2 : A0A0G2K2M9	1.72	yes	up	9.0E-05	yes	chr10	exon	internal exon	NA	1.20	no	up	0.82	no	1.00	no	up	9.0E-01	no
MSTRG.5417.7 : Auts2 : F1M388	0.04	yes	down	4.8E-14	yes	chr12	intron	internal intron	NA	0.80	no	down	0.38	no	1.01	no	up	5.6E-01	no
ENSRNOT00000025067 : Hspa5 : P06761	0.56	yes	down	2.4E-04	yes	chr3	intron	internal intron	NA	1.09	no	up	0.87	no	1.06	no	up	6.4E-09	yes
MSTRG.19777.1 : Sin3a : A0A0G2K3H5	0.53	yes	down	4.1E-06	yes	chr8	intron	last intron	NA	1.15	no	up	0.47	no	0.96	no	down	6.2E-05	yes
MSTRG.5723.3 : Ttc28 : D3ZXP1	4.41	yes	up	1.9E-04	yes	chr12	promoter	Intermediate	LCP	2.06	yes	up	0.05	yes	0.86	no	down	6.9E-05	yes
ENSRNOT00000012334 : Col5a1 : A0A0G2JX47	0.33	yes	down	4.5E-04	yes	chr3	intron	internal intron	NA	0.89	no	down	0.53	no	1.01	no	up	4.0E-01	no
MSTRG.5897.1 : Cntn2 : G3V758	2.37	yes	up	8.3E-06	yes	chr13	exon	internal exon	NA	1.00	no	down	0.50	no	1.00	no	down	9.1E-01	no
ENSRNOT00000001115 : Ddx39b : Q63413	0.02	yes	down	4.7E-11	yes	chr20	exon	last exon	NA	2.53	yes	up	0.10	no	1.01	no	up	2.0E-01	no
MSTRG.4525.6 : Mx1 : Q499S4	3.65	yes	up	1.1E-13	yes	chr11	intron	internal intron	NA	2.23	yes	up	0.51	no	1.36	yes	up	1.2E-13	yes
MSTRG.12274.1 : Ctnna3 : F1M4I1	0	yes	down	1.3E-06	yes	chr20	promoter	Distal	LCP	0.77	no	down	0.44	no	0.92	no	down	3.6E-04	yes
MSTRG.11964.6 : Ddx39b : Q63413	0.02	yes	down	4.7E-11	yes	chr20	exon	last exon	NA	0.33	yes	down	0.28	no	1.01	no	up	2.0E-01	no
ENSRNOT00000020910 : Rftn2 : D3ZII1	34.41	yes	up	1.8E-14	yes	chr9	exon	last exon	NA	0.98	no	down	0.93	no	0.99	no	down	8.1E-01	no
MSTRG.10716.3 : Trio : F1M0Z1	0.35	yes	down	1.6E-06	yes	chr2	exon	first exon	NA	0.97	no	down	0.86	no	1.02	no	up	2.1E-02	yes
ENSRNOT00000010101 : Lgmn : Q5PPG2	2.26	yes	up	3.7E-04	yes	chr6	intron	internal intron	NA	1.42	no	up	0.28	no	1.15	no	up	3.3E-08	yes
MSTRG.15231.4 : Tspan9 : D4AAV9	0.41	yes	down	1.4E-09	yes	chr4	exon	last exon	NA	0.74	no	down	0.50	no	0.96	no	down	2.4E-05	yes
ENSRNOT00000076460 : Parp4 : A0A096MJ98	1.23	yes	up	1.1E-06	yes	chr15	intron	last intron	NA	0.92	no	down	0.11	no	1.00	no	down	9.9E-01	no
ENSRNOT00000082549 : Nebl : F1LVX3	0	yes	down	3.8E-04	yes	chr17	intron	first intron	NA	0.89	no	down	0.40	no	1.02	no	up	2.0E-01	no
ENSRNOT00000039353 : Eogt : Q5NDL0	6.71	yes	up	1.7E-04	yes	chr4	intron	internal intron	NA	1.08	no	up	0.10	no	1.10	no	up	2.0E-05	yes
MSTRG.8552.9 : Ddx46 : Q62780	0.56	yes	down	3.4E-04	yes	chr17	intron	internal intron	NA	0.82	no	down	0.28	no	0.98	no	down	1.3E-01	no
ENSRNOT00000029510 : Mansc1 : D4AE21	0.45	yes	down	1.5E-04	yes	chr4	exon	first exon	NA	0.71	no	down	0.05	yes	0.85	no	down	5.3E-06	yes
ENSRNOT00000023290 : LOC108348175 : A0A0G20.05		yes	down	4.0E-19	yes	chr1	promoter	Intermediate	LCP	1.00	no	down	0.97	no	1.01	no	up	4.0E-01	no
ENSRNOT00000067796 : Smc5 : D4A9F0	4.35	yes	up	2.4E-09	yes	chr1	promoter	Distal	LCP	1.01	no	up	0.09	no	0.86	no	down	3.1E-03	yes
MSTRG.14252.1 : Akap9 : F1LPB4	2.74	yes	up	2.9E-04	yes	chr4	intron	internal intron	NA	1.00	no	down	0.99	no	1.05	no	up	2.3E-05	yes
MSTRG.19819.2 : Neo1 : P97603	0	yes	down	3.5E-04	yes	chr8	promoter	Intermediate	LCP	1.54	no	up	0.34	no	0.98	no	down	1.8E-02	yes
MSTRG.10569.3 : Plpp1 : O08564	0.47	yes	down	8.3E-05	yes	chr2	intron	internal intron	NA	1.21	no	up	0.54	no	1.04	no	up	2.9E-03	yes
MSTRG.18043.2 : Stat2 : Q5XI26	0.06	yes	down	1.0E-04	yes	chr7	promoter	Distal	LCP	0.69	no	down	0.15	no	1.14	no	up	1.9E-09	yes
MSTRG.16517.1 : Slc9a1 : Q8R4H8	0.61	yes	down	7.2E-05	yes	chr5	intron	internal intron	NA	0.70	no	down	0.48	no	1.05	no	up	2.8E-01	no
MSTRG.21176.1 : Inpp5d : F1M981	0.08	yes	down	9.9E-05	yes	chr9	intron	internal intron	NA	0.38	yes	down	0.04	yes	1.08	no	up	3.1E-05	yes
MSTRG.8033.1 : Slc27a1 : Q6GMM8	0.26	yes	down	5.6E-15	yes	chr16	intron	internal intron	NA	1.05	no	up	0.91	no	0.99	no	down	2.5E-01	no
ENSRNOT00000087961 : Nap1l4 : Q5U2Z3	0.36	yes	down	7.2E-05	yes	chr1	intron	internal intron	NA	0.62	no	down	0.62	no	0.98	no	down	3.1E-02	yes
ENSRNOT00000077015 : Prdm16 : M0RDL0	0.37	yes	down	2.7E-04	yes	chr5	exon	internal exon	NA	0.76	no	down	0.02	yes	0.83	yes	down	1.1E-06	yes
MSTRG.21122.3 : Agfg1 : F1M9N7	3.59	yes	up	1.8E-08	yes	chr9	exon	internal exon	NA	0.87	no	down	0.46	no	1.16	no	up	6.0E-06	yes
MSTRG.18208.1 : Atp5d : G3V7Y3	0.12	yes	down	5.2E-08	yes	chr7	promoter	Distal	LCP	0.25	yes	down	0.40	no	0.90	no	down	7.4E-12	yes
ENSRNOT00000010349 : Kmt2c : A0A0G2K426	0.22	yes	down	1.1E-11	yes	chr4	promoter	Distal	LCP	1.22	no	up	0.42	no	0.69	yes	down	1.4E-11	yes
MSTRG.18072.2 : Dgka : P51556	0.72	yes	down	1.5E-04	yes	chr7	exon	internal exon	NA	1.45	no	up	0.21	no	0.95	no	down	8.8E-04	yes
MSTRG.11222.1 : Fdps : F1LND7	inf	yes	up	2.2E-05	yes	chr2	exon	last exon	NA	1.01	no	up	0.98	no	1.07	no	up	1.2E-03	yes
MSTRG.11265.13 : Ubap2l : E9PTR4	6.62	yes	up	4.0E-04	yes	chr2	intron	internal intron	NA	1.21	no	up	0.38	no	1.13	no	up	3.8E-12	yes
MSTRG.2881.9 : Pkd1 : Q9ERV0	inf	yes	up	6.6E-05	yes	chr10	intron	internal intron	NA	0.97	no	down	0.93	no	1.19	no	up	9.8E-07	yes
MSTRG.8278.27 : Rbpms : F2Z3S5	0.02	yes	down	2.6E-05	yes	chr16	promoter	Proximal	ICP	1.07	no	up	0.50	no	1.06	no	up	4.9E-05	yes
MSTRG.15231.3 : Tspan9 : D4AAV9	0.41	yes	down	1.4E-09	yes	chr4	exon	last exon	NA	1.70	no	up	0.42	no	0.96	no	down	2.4E-05	yes
MSTRG.332.3 : Fgfr1op : Q4V7C1	7.97	yes	up	2.8E-17	yes	chr1	intron	internal intron	NA	0.64	no	down	0.46	no	1.07	no	up	7.3E-04	yes
MSTRG.16327.11 : Macf1 : A0A0G2K9T4	0.36	yes	down	1.3E-05	yes	chr5	exon	last exon	NA	0.35	yes	down	0.40	no	0.97	no	down	9.0E-11	yes
MSTRG.10569.2 : Plpp1 : O08564	0.47	yes	down	8.3E-05	yes	chr2	intron	internal intron	NA	0.67	no	down	0.11	no	1.04	no	up	2.9E-03	yes
MSTRG.6818.3 : Rbpj : M0R7Q3	0.34	yes	down	7.0E-05	yes	chr14	intron	last intron	NA	0.77	no	down	0.69	no	1.08	no	up	1.1E-04	yes
MSTRG.8325.2 : Hook3 : Q7TQ77	0.22	yes	down	2.1E-04	yes	chr16	intron	internal intron	NA	2.60	yes	up	0.36	no	1.01	no	up	5.0E-03	yes
MSTRG.14930.2 : Grip2 : A0A0H2UHH8	2.18	yes	up	2.2E-05	yes	chr4	intron	internal intron	NA	0.87	no	down	0.42	no	1.01	no	up	5.2E-01	no
MSTRG.12280.3 : Herc4 : Q5PQN1	2.70	yes	up	6.2E-07	yes	chr20	intron	internal intron	NA	0.77	no	down	0.50	no	1.00	no	down	9.1E-01	no
MSTRG.19963.5 : Sltm : A0A0G2K904	0.34	yes	down	6.4E-06	yes	chr8	intron	internal intron	NA	0.85	no	down	0.73	no	0.94	no	down	2.8E-02	yes
ENSRNOT00000076511 : Synm : G3V9G5	1.68	yes	up	3.0E-04	yes	chr1	exon	last exon	NA	0.43	yes	down	0.01	yes	0.82	yes	down	8.8E-13	yes
ENSRNOT00000011969 : Slc35a1 : D3ZZ48	0.47	yes	down	4.9E-04	yes	chr5	exon	internal exon	NA	1.75	no	up	0.03	yes	1.00	no	up	9.2E-01	no
MSTRG.11405.3 : Pde4dip : A0A0G2JW66	6.94	yes	up	5.0E-08	yes	chr2	exon	internal exon	NA	1.04	no	up	0.47	no	1.06	no	up	5.1E-04	yes
ENSRNOT00000041571 : Cacna1c : F1MA84	4.75	yes	up	4.3E-04	yes	chr4	intron	internal intron	NA	0.89	no	down	0.62	no	0.97	no	down	2.7E-02	yes
ENSRNOT00000091287 : Srrm2 : A0A0G2K2M9	1.72	yes	up	9.0E-05	yes	chr10	exon	internal exon	NA	1.10	no	up	0.86	no	1.00	no	up	9.0E-01	no
MSTRG.16771.3 : H6pd : D4A7D7	0.22	yes	down	2.6E-06	yes	chr5	intron	last intron	NA	0.58	no	down	0.50	no	1.14	no	up	9.6E-15	yes
ENSRNOT00000028445 : Slc4a1 : Q5U329	0.42	yes	down	3.2E-08	yes	chr10	intron	internal intron	NA	1.00	no	down	0.48	no	0.87	no	down	1.3E-09	yes
MSTRG.11263.2 : Atp8b2 : D4A509	0.14	yes	down	3.1E-04	yes	chr2	exon	first exon	NA	0.68	no	down	0.47	no	0.92	no	down	5.0E-03	yes
MSTRG.10603.2 : Nnt : Q5BJZ3	3.00	yes	up	1.8E-04	yes	chr2	intron	internal intron	NA	1.18	no	up	0.67	no	0.96	no	down	1.2E-07	yes
MSTRG.5399.3 : Eln : A0A0G2JST5	3.55	yes	up	3.0E-04	yes	chr12	intron	internal intron	NA	0.82	no	down	0.64	no	0.91	no	down	1.2E-01	no
ENSRNOT00000091569 : Zfp592 : D3ZJG8	0.22	yes	down	8.1E-08	yes	chr1	exon	internal exon	NA	0.96	no	down	0.46	no	1.06	no	up	1.8E-05	yes
MSTRG.20044.2 : Phip : F1M3B3	4.05	yes	up	4.4E-04	yes	chr8	intron	internal intron	NA	1.22	no	up	0.86	no	0.95	no	down	6.3E-02	no
MSTRG.12137.3 : Pknox1 : Q5BJP1	0.26	yes	down	3.4E-07	yes	chr20	promoter	Proximal	LCP	1.22	no	up	0.60	no	0.89	no	down	7.5E-04	yes
MSTRG.20878.6 : Gls : P13264	0.05	yes	down	1.5E-13	yes	chr9	intron	first intron	NA	1.14	no	up	0.89	no	1.08	no	up	4.1E-02	yes
ENSRNOT00000007583 : Srsf5 : Q09167	0.10	yes	down	3.0E-09	yes	chr6	promoter	Proximal	ICP	1.12	no	up	0.72	no	1.03	no	up	4.2E-02	yes
ENSRNOT00000041168 : Gp1bb : Q9JJM7	0	yes	down	2.2E-04	yes	chr11	intron	internal intron	NA	0.62	no	down	0.42	no	0.93	no	down	7.7E-08	yes
ENSRNOT00000014207 : Cand2 : G3V7E8	0.39	yes	down	2.9E-04	yes	chr4	exon	internal exon	NA	1.01	no	up	0.23	no	0.97	no	down	1.3E-05	yes
ENSRNOT00000061332 : Epb41l2 : D3ZM69	0.25	yes	down	1.1E-12	yes	chr1	intron	internal intron	NA	0.99	no	down	0.14	no	0.91	no	down	8.9E-11	yes
ENSRNOT00000016449 : Ktn1 : D4A4Z9	2.49	yes	up	1.2E-04	yes	chr15	intron	internal intron	NA	1.22	no	up	0.22	no	1.04	no	up	2.2E-11	yes
ENSRNOT00000085022 : Ube4a : F1M9N5	4.51	yes	up	1.3E-04	yes	chr8	intron	internal intron	NA	1.37	no	up	0.24	no	0.97	no	down	2.4E-03	yes
MSTRG.7470.8 : Acin1 : E9PST5	0.30	yes	down	1.1E-04	yes	chr15	promoter	Distal	LCP	1.00	no	down	0.99	no	0.98	no	down	5.1E-04	yes
MSTRG.11265.12 : Ubap2l : E9PTR4	6.62	yes	up	4.0E-04	yes	chr2	intron	internal intron	NA	0.88	no	down	0.52	no	1.13	no	up	3.8E-12	yes
MSTRG.14606.3 : Creb5 : A0A0G2K5F0	0.10	yes	down	1.8E-23	yes	chr4	intron	internal intron	NA	0.85	no	down	0.72	no	1.01	no	up	7.4E-01	no
ENSRNOT00000075621 : Exoc4 : M0R649	0.23	yes	down	6.6E-05	yes	chr4	exon	internal exon	NA	1.12	no	up	0.13	no	0.99	no	down	3.0E-01	no
MSTRG.19895.6 : Hacd3 : D4ABI7	0.11	yes	down	1.7E-06	yes	chr8	intron	internal intron	NA	1.26	no	up	0.47	no	1.02	no	up	6.2E-05	yes
ENSRNOT00000017727 : Scube1 : F1M987	2.13	yes	up	1.3E-04	yes	chr7	intron	internal intron	NA	1.48	no	up	0.39	no	0.80	yes	down	1.2E-14	yes
ENSRNOT00000024066 : Sirt5 : Q68FX9	6.16	yes	up	1.6E-04	yes	chr17	intron	internal intron	NA	0.49	yes	down	0.44	no	1.01	no	up	5.1E-01	no
MSTRG.22038.3 : Fmr1 : Q80WE1	inf	yes	up	3.0E-04	yes	chrX	intron	internal intron	NA	0.82	no	down	0.27	no	1.09	no	up	7.3E-06	yes
MSTRG.12235.1 : Cisd1 : B0K020	15.21	yes	up	2.4E-04	yes	chr20	promoter	Distal	ICP	0.96	no	down	0.69	no	0.97	no	down	6.1E-02	no
ENSRNOT00000011991 : Csrp1 : P47875	1.98	yes	up	3.9E-04	yes	chr13	promoter	Intermediate	LCP	5.17	yes	up	0.53	no	1.11	no	up	3.1E-08	yes
ENSRNOT00000018422 : Ercc3 : Q4G005	0.04	yes	down	1.2E-04	yes	chr18	promoter	Intermediate	ICP	0.67	no	down	0.39	no	0.97	no	down	5.7E-03	yes
MSTRG.1651.2 : Mettl9 : G0Z7P9	inf	yes	up	1.6E-04	yes	chr1	intron	first intron	NA	0.86	no	down	0.72	no	0.99	no	down	7.9E-01	no
ENSRNOT00000049822 : Tardbp : I6L9G6	2.86	yes	up	4.5E-10	yes	chr5	intron	internal intron	NA	1.90	no	up	0.02	yes	1.05	no	up	5.8E-11	yes
MSTRG.15110.3 : Wnk1 : Q9JIH7	18.77	yes	up	1.6E-38	yes	chr4	exon	internal exon	NA	2.50	yes	up	0.08	no	1.04	no	up	1.7E-03	yes
MSTRG.10127.1 : Kars : Q5XIM7	16.06	yes	up	5.9E-09	yes	chr19	exon	last exon	NA	1.42	no	up	0.61	no	0.95	no	down	1.9E-07	yes
ENSRNOT00000021200 : Qpctl : B5DFI7	20.24	yes	up	4.4E-10	yes	chr1	intron	first intron	NA	1.03	no	up	0.96	no	1.00	no	down	6.3E-01	no
ENSRNOT00000016784 : Trio : F1M0Z1	0.35	yes	down	1.6E-06	yes	chr2	exon	first exon	NA	1.39	no	up	0.47	no	1.02	no	up	2.1E-02	yes
ENSRNOT00000055572 : Ube4b : F1M8V2	0.02	yes	down	2.8E-12	yes	chr5	exon	last exon	NA	1.39	no	up	0.05	yes	1.06	no	up	1.6E-06	yes
MSTRG.5280.1 : Taf6 : Q498R0	0.74	yes	down	2.2E-06	yes	chr12	intron	internal intron	NA	0.89	no	down	0.57	no	0.91	no	down	1.8E-03	yes
ENSRNOT00000023741 : Ctr9 : G3V897	12.42	yes	up	1.5E-04	yes	chr1	intron	internal intron	NA	1.71	no	up	0.47	no	1.02	no	up	9.9E-02	no
MSTRG.20352.14 : Ubp1 : D4A030	0.44	yes	down	2.3E-04	yes	chr8	intron	internal intron	NA	0.87	no	down	0.40	no	0.98	no	down	1.9E-01	no
ENSRNOT00000072373 : Rbm14 : M0R9Q1	0.55	yes	down	9.3E-08	yes	chr1	intron	first intron	NA	1.05	no	up	0.91	no	1.03	no	up	3.8E-06	yes
ENSRNOT00000056481 : Puf60 : A0A0H2UHZ6	0.25	yes	down	1.3E-05	yes	chr7	exon	internal exon	NA	1.15	no	up	0.46	no	1.00	no	down	7.4E-02	no
MSTRG.9502.1 : Prrc1 : G3V834	0.26	yes	down	1.1E-04	yes	chr18	exon	first exon	NA	0.96	no	down	0.91	no	1.12	no	up	3.2E-14	yes
ENSRNOT00000065065 : Fdps : F1LND7	inf	yes	up	2.2E-05	yes	chr2	exon	last exon	NA	1.11	no	up	0.50	no	1.07	no	up	1.2E-03	yes
MSTRG.19173.7 : Ano6 : A0A0G2K1M7	3.87	yes	up	9.8E-09	yes	chr7	intron	internal intron	NA	0.71	no	down	0.50	no	0.91	no	down	2.1E-09	yes
MSTRG.18734.2 : Derl1 : F7FNS3	0.48	yes	down	3.9E-05	yes	chr7	exon	last exon	NA	1.18	no	up	0.58	no	1.04	no	up	2.4E-03	yes
ENSRNOT00000076460 : Parp4 : A0A096MK99	1.23	yes	up	1.1E-06	yes	chr15	intron	last intron	NA	0.92	no	down	0.11	no	0.94	no	down	4.1E-06	yes
MSTRG.7171.2 : Efemp1 : Q6AXN2	0.06	yes	down	2.6E-05	yes	chr14	exon	last exon	NA	1.11	no	up	0.64	no	0.94	no	down	1.1E-08	yes
ENSRNOT00000087033 : Rbm3 : G3V6P6	5.50	yes	up	1.2E-04	yes	chrX	intron	first intron	NA	0.84	no	down	0.23	no	0.93	no	down	8.6E-07	yes
MSTRG.8278.9 : Rbpms : F2Z3S5	0.02	yes	down	2.6E-05	yes	chr16	promoter	Proximal	ICP	1.03	no	up	0.94	no	1.06	no	up	4.9E-05	yes

MSTRG.7804.1 : Mycbp2 : A0A1W2Q6I3	0.31	yes	down	9.2E-06	yes	chr15	intron	internal intron	NA	1.03	no	up	0.49	no	1.00	no	up	8.8E-01	no
MSTRG.13558.3 : Siglec1 : A0A0G2K320	0.24	yes	down	6.2E-06	yes	chr3	promoter	Proximal	LCP	1.35	no	up	0.09	no	1.25	yes	up	7.1E-13	yes
ENSRNOT00000027220 : Scly : Q68FT9	0.19	yes	down	5.0E-08	yes	chr9	intron	internal intron	NA	0.96	no	down	0.80	no	0.92	no	down	4.2E-10	yes
ENSRNOT00000087739 : Kif1b : A0A0G2KA12	3.65	yes	up	7.4E-05	yes	chr5	intron	internal intron	NA	1.00	no	down	0.47	no	1.05	no	up	6.5E-02	no
MSTRG.1546.2 : Ampd3 : O09178	0.39	yes	down	3.1E-04	yes	chr1	intron	internal intron	NA	0.91	no	down	0.85	no	0.94	no	down	8.5E-03	yes
MSTRG.15356.4 : Pde3a : Q62865	0.30	yes	down	3.1E-07	yes	chr4	intron	internal intron	NA	0.85	no	down	0.76	no	1.00	no	up	9.7E-01	no
MSTRG.5804.9 : Clasp1 : F1LNR1	0.36	yes	down	6.7E-07	yes	chr13	intron	internal intron	NA	0.47	yes	down	0.05	yes	1.00	no	down	7.0E-01	no
MSTRG.21401.3 : Usp9x : D3ZC84	21.85	yes	up	4.1E-05	yes	chrX	exon	last exon	NA	1.30	no	up	0.28	no	1.00	no	up	7.5E-01	no
MSTRG.3895.6 : Cdk12 : A0A0G2K5U7	5.94	yes	up	1.1E-04	yes	chr10	exon	first exon	NA	1.24	no	up	0.49	no	0.97	no	down	5.5E-04	yes
MSTRG.20764.3 : Uggt1 : Q9JLA3	0.22	yes	down	3.9E-04	yes	chr9	intron	internal intron	NA	0.91	no	down	0.79	no	1.11	no	up	9.8E-15	yes
ENSRNOT00000083779 : Asph : A0A096MKE0	2.56	yes	up	9.8E-06	yes	chr5	intron	internal intron	NA	1.00	no	down	0.76	no	0.99	no	down	1.3E-01	no
ENSRNOT00000012190 : Cntn2 : G3V758	2.37	yes	up	8.3E-06	yes	chr13	exon	internal exon	NA	1.02	no	up	0.96	no	1.00	no	down	9.1E-01	no
MSTRG.12279.1 : Ctnna3 : F1M4I1	0	yes	down	1.3E-06	yes	chr20	promoter	Distal	LCP	0.68	no	down	0.54	no	0.92	no	down	3.6E-04	yes
ENSRNOT00000020752 : Pign : E9PTA5	0.23	yes	down	2.2E-04	yes	chr13	intron	internal intron	NA	1.81	no	up	0.19	no	1.05	no	up	3.6E-03	yes
ENSRNOT00000078980 : Morc2 : D4A2C4	6.36	yes	up	1.5E-04	yes	chr14	promoter	Proximal	HCP	1.20	no	up	0.40	no	0.97	no	down	3.2E-01	no
MSTRG.11964.2 : Ddx39b : Q63413	0.02	yes	down	4.7E-11	yes	chr20	exon	last exon	NA	0.69	no	down	0.68	no	1.01	no	up	2.0E-01	no
MSTRG.7830.3 : Dock9 : F1LSM8	0.10	yes	down	2.7E-05	yes	chr15	intron	internal intron	NA	1.51	no	up	0.38	no	0.97	no	down	2.3E-04	yes
MSTRG.16557.9 : Srrm1 : B2RYB3	2.05	yes	up	2.0E-04	yes	chr5	intron	internal intron	NA	1.64	no	up	0.17	no	1.10	no	up	2.8E-06	yes
MSTRG.17588.1 : Ylpm1 : A0A0G2K678	0.14	yes	down	2.3E-07	yes	chr6	intron	internal intron	NA	0.75	no	down	0.15	no	1.02	no	up	1.8E-03	yes
MSTRG.11888.1 : Ppp1r18 : A0A0G2K8B3	0.32	yes	down	2.0E-04	yes	chr20	promoter	Distal	LCP	1.88	no	up	0.53	no	1.10	no	up	1.1E-05	yes
ENSRNOT00000045315 : Col18a1 : F1LR02	0.22	yes	down	4.1E-11	yes	chr20	exon	internal exon	NA	0.86	no	down	0.58	no	1.03	no	up	3.6E-07	yes
MSTRG.18210.2 : Stk11 : A0A0H2UI02	0.18	yes	down	9.1E-05	yes	chr7	exon	last exon	NA	1.03	no	up	0.74	no	0.88	no	down	1.8E-03	yes
MSTRG.19088.5 : Scube1 : F1M987	2.13	yes	up	1.3E-04	yes	chr7	intron	internal intron	NA	1.62	no	up	0.13	no	0.80	yes	down	1.2E-14	yes
MSTRG.20764.3 : Uggt1 : Q9JLA3	0.22	yes	down	3.9E-04	yes	chr9	intron	internal intron	NA	0.56	no	down	0.44	no	1.11	no	up	9.8E-15	yes
MSTRG.6480.8 : Evi5 : D3ZJN9	4.04	yes	up	4.0E-04	yes	chr14	intron	internal intron	NA	0.98	no	down	0.98	no	0.98	no	down	2.5E-01	no
ENSRNOT00000014023 : Fam126a : A0A0G2K3C7	0.38	yes	down	2.9E-04	yes	chr4	promoter	Intermediate	LCP	1.31	no	up	0.19	no	1.07	no	up	1.8E-06	yes
MSTRG.20352.3 : Ubp1 : D4A030	0.44	yes	down	2.3E-04	yes	chr8	intron	internal intron	NA	0.98	no	down	0.98	no	0.98	no	down	1.9E-01	no
ENSRNOT00000006116 : Sec14l4 : A0A0G2JWI8	0.38	yes	down	1.3E-04	yes	chr14	intron	internal intron	NA	0.65	no	down	0.30	no	0.91	no	down	1.1E-08	yes
MSTRG.19710.3 : Alg9 : D3ZCW5	0.10	yes	down	9.3E-08	yes	chr8	intron	first intron	NA	1.39	no	up	0.09	no	1.11	no	up	6.0E-03	yes
MSTRG.8948.3 : Mtpap : D3ZPN5	1.99	yes	up	1.4E-05	yes	chr17	intron	internal intron	NA	0.68	no	down	0.04	yes	1.03	no	up	1.2E-01	no
MSTRG.6713.5 : Fryl : D3ZQY4	2.76	yes	up	1.7E-04	yes	chr14	intron	internal intron	NA	0.67	no	down	0.36	no	1.00	no	down	4.6E-01	no
MSTRG.22064.11 : Arhgap4 : A0A0G2JVF0	0.34	yes	down	1.4E-05	yes	chrX	intron	internal intron	NA	1.65	no	up	0.16	no	1.07	no	up	7.1E-04	yes
MSTRG.18043.7 : Stat2 : Q5XI26	0.06	yes	down	1.0E-04	yes	chr7	promoter	Distal	LCP	0.93	no	down	0.72	no	1.14	no	up	1.9E-09	yes
ENSRNOT00000010891 : Ptgis : Q62969	0.55	yes	down	6.0E-05	yes	chr3	intron	internal intron	NA	0.65	no	down	0.03	yes	0.99	no	down	7.4E-02	no
MSTRG.7061.1 : Tbrg4 : Q5M9G9	0.25	yes	down	2.7E-04	yes	chr14	promoter	Distal	LCP	0.96	no	down	0.89	no	0.99	no	down	1.9E-01	no
MSTRG.16517.2 : Slc9a1 : Q8R4H8	0.61	yes	down	7.2E-05	yes	chr5	intron	internal intron	NA	1.34	no	up	0.44	no	1.05	no	up	2.8E-01	no
MSTRG.7822.1 : Rap2a : A0A0G2JTW1	4.31	yes	up	1.6E-04	yes	chr15	intron	last intron	NA	1.12	no	up	0.14	no	0.76	yes	down	2.5E-10	yes
ENSRNOT00000078589 : Bclaf1 : B1WC16	0.67	yes	down	1.3E-07	yes	chr1	intron	internal intron	NA	1.39	no	up	0.68	no	1.03	no	up	6.8E-02	no
ENSRNOT00000086853 : Triobp : A2TIS7	0.29	yes	down	2.0E-06	yes	chr7	intron	internal intron	NA	0.63	no	down	0.32	no	0.97	no	down	5.1E-06	yes
MSTRG.5280.4 : Taf6 : Q498R0	0.74	yes	down	2.2E-06	yes	chr12	intron	internal intron	NA	0.87	no	down	0.39	no	0.91	no	down	1.8E-03	yes
ENSRNOT00000068013 : Polr2a : D4A5A6	2.68	yes	up	1.4E-05	yes	chr10	exon	internal exon	NA	1.22	no	up	0.33	no	1.00	no	down	6.0E-01	no
MSTRG.5082.2 : Hsph1 : Q66HA8	inf	yes	up	3.0E-05	yes	chr12	intron	internal intron	NA	0.71	no	down	0.52	no	1.12	no	up	9.8E-13	yes
ENSRNOT00000080253 : Spg11 : D3Z9Z3	3.32	yes	up	5.0E-08	yes	chr3	exon	internal exon	NA	1.13	no	up	0.02	yes	0.97	no	down	1.4E-02	yes
MSTRG.3572.8 : RGD1307929 : F1LSX1	7.34	yes	up	1.1E-12	yes	chr10	intron	internal intron	NA	1.64	no	up	0.59	no	0.97	no	down	1.8E-04	yes
ENSRNOT00000031770 : Arhgap17 : D4AAV2	0.83	yes	down	2.6E-07	yes	chr1	intron	internal intron	NA	0.91	no	down	0.82	no	1.01	no	up	3.1E-01	no
ENSRNOT00000038246 : Thada : D3ZVT2	inf	yes	up	4.7E-04	yes	chr6	intron	internal intron	NA	1.02	no	up	0.94	no	0.88	no	down	1.3E-03	yes
MSTRG.20998.1 : Pikfyve : D3ZYT8	0.33	yes	down	2.0E-04	yes	chr9	intron	internal intron	NA	0.49	yes	down	0.14	no	1.22	yes	up	6.5E-08	yes
ENSRNOT00000057950 : Ctnna3 : F1M4I1	0	yes	down	1.3E-06	yes	chr20	promoter	Distal	LCP	1.14	no	up	0.59	no	0.92	no	down	3.6E-04	yes
MSTRG.19895.1 : Hacd3 : D4ABI7	0.11	yes	down	1.7E-06	yes	chr8	intron	internal intron	NA	1.36	no	up	0.46	no	1.02	no	up	6.2E-05	yes
ENSRNOT00000038652 : Atp8b1 : D4AA47	0.41	yes	down	3.3E-04	yes	chr18	promoter	Distal	LCP	0.99	no	down	0.93	no	1.06	no	up	7.8E-04	yes
MSTRG.2839.11 : Srrm2 : A0A0G2K2M9	1.72	yes	up	9.0E-05	yes	chr10	exon	internal exon	NA	0.69	no	down	0.81	no	1.00	no	up	9.0E-01	no
MSTRG.4716.3 : Adcy5 : G3V9G1	0.16	yes	down	2.3E-10	yes	chr11	intron	internal intron	NA	0.68	no	down	0.43	no	1.00	no	down	7.6E-01	no
MSTRG.11268.3 : Tpm3 : A0A140TAF0	0.37	yes	down	8.0E-05	yes	chr2	intron	internal intron	NA	0.97	no	down	0.91	no	1.03	no	up	4.4E-04	yes
MSTRG.12668.1 : Nup188 : F1LRC6	0.22	yes	down	2.3E-05	yes	chr3	exon	internal exon	NA	2.25	yes	up	0.09	no	1.02	no	up	1.9E-01	no
ENSRNOT00000021171 : Sfxn3 : Q9JHY2	0.39	yes	down	7.8E-05	yes	chr1	intron	internal intron	NA	0.92	no	down	0.57	no	0.98	no	down	7.5E-03	yes
ENSRNOT00000025536 : Ntrk3 : Q68G04	7.03	yes	up	1.5E-06	yes	chr1	intron	internal intron	NA	0.90	no	down	0.20	no	0.87	no	down	1.8E-07	yes
ENSRNOT00000033975 : Tnrc6b : A0A0G2K6R0	2.14	yes	up	4.3E-13	yes	chr7	exon	last exon	NA	0.69	no	down	0.52	no	1.01	no	up	7.4E-01	no
ENSRNOT00000080382 : Zeb2 : A0A0G2K8T6	0.34	yes	down	8.0E-09	yes	chr3	intron	internal intron	NA	0.87	no	down	0.47	no	1.01	no	up	2.4E-01	no
MSTRG.877.3 : Lsm14a : A0A0G2JUK2	0.31	yes	down	3.8E-04	yes	chr1	intron	last intron	NA	1.25	no	up	0.55	no	1.02	no	up	2.8E-01	no
ENSRNOT00000006168 : Nfic : O70188	1.68	yes	up	1.4E-06	yes	chr7	intron	internal intron	NA	0.94	no	down	0.59	no	0.85	no	down	3.8E-12	yes
MSTRG.15840.1 : Tmem245 : D3ZXD8	5.92	yes	up	3.2E-05	yes	chr5	intron	internal intron	NA	2.00	yes	up	0.38	no	0.88	no	down	4.0E-07	yes
MSTRG.8511.2 : Dapk1 : F1LNN8	0.35	yes	down	1.5E-04	yes	chr17	intron	internal intron	NA	1.44	no	up	0.09	no	0.96	no	down	1.0E-01	no
ENSRNOT00000087241 : Podxl : A0A0G2K2L1	0.31	yes	down	1.5E-14	yes	chr4	intron	internal intron	NA	1.36	no	up	0.09	no	0.91	no	down	6.5E-07	yes
ENSRNOT00000008211 : Cpq : Q6IRK9	0.21	yes	down	1.1E-08	yes	chr7	intron	internal intron	NA	1.44	no	up	0.05	yes	1.02	no	up	4.8E-04	yes
MSTRG.2630.1 : Pdcd11 : D3ZNI3	0.34	yes	down	5.3E-07	yes	chr1	exon	internal exon	NA	1.19	no	up	0.29	no	1.08	no	up	5.5E-06	yes
ENSRNOT00000092987 : Stim1 : P84903	2.61	yes	up	1.3E-04	yes	chr1	exon	last exon	NA	0.99	no	down	0.13	no	0.92	no	down	3.4E-08	yes
MSTRG.3645.6 : Slfn13 : A0A096MKD0	2.27	yes	up	1.5E-04	yes	chr10	promoter	Intermediate	LCP	1.20	no	up	0.84	no	0.97	no	down	2.7E-02	yes
MSTRG.4978.1 : Pi4ka : A0A140TAJ5	7.84	yes	up	1.4E-07	yes	chr11	intron	internal intron	NA	0.45	yes	down	0.01	yes	0.97	no	down	1.2E-03	yes
MSTRG.19125.3 : Brd1 : D3ZUW8	7.17	yes	up	3.3E-04	yes	chr7	exon	internal exon	NA	1.44	no	up	0.75	no	1.21	yes	up	6.4E-02	no
MSTRG.11962.2 : Ddx39b : Q63413	0.02	yes	down	4.7E-11	yes	chr20	exon	last exon	NA	1.34	no	up	0.57	no	1.01	no	up	2.0E-01	no
MSTRG.13128.1 : Itgav : F1LZX9	24.74	yes	up	1.9E-17	yes	chr3	intron	first intron	NA	1.72	no	up	0.21	no	0.92	no	down	1.7E-15	yes
MSTRG.15068.1 : Washc2c : F1LPG9	0.05	yes	down	2.7E-19	yes	chr4	exon	internal exon	NA	1.12	no	up	0.80	no	1.05	no	up	2.1E-05	yes
ENSRNOT00000024617 : Tpm1 : A0A0G2JSQ4	9.41	yes	up	5.1E-14	yes	chr8	intron	internal intron	NA	0.67	no	down	0.44	no	0.81	yes	down	2.5E-10	yes
ENSRNOT00000084971 : RT1-CE7 : D3ZLE6	inf	yes	up	7.3E-07	yes	chr20	exon	internal exon	NA	1.06	no	up	0.53	no	1.22	yes	up	7.3E-16	yes
MSTRG.5417.6 : Auts2 : F1M388	0.04	yes	down	4.8E-14	yes	chr12	intron	internal intron	NA	1.15	no	up	0.57	no	1.01	no	up	5.6E-01	no
MSTRG.18037.1 : Rbms2 : Q4QR81	0.04	yes	down	2.6E-23	yes	chr7	exon	last exon	NA	1.14	no	up	0.75	no	1.14	no	up	2.7E-10	yes
MSTRG.15231.8 : Tspan9 : D4AAV9	0.41	yes	down	1.4E-09	yes	chr4	exon	last exon	NA	1.23	no	up	0.69	no	0.96	no	down	2.4E-05	yes
ENSRNOT00000014583 : Mycbp2 : A0A1W2Q6I3	0.31	yes	down	9.2E-06	yes	chr15	intron	internal intron	NA	1.00	no	down	0.84	no	1.00	no	up	8.8E-01	no
MSTRG.6560.3 : Hnrnpdl : A0A0G2KAZ7	inf	yes	up	7.6E-05	yes	chr14	intron	first intron	NA	1.00	no	down	0.99	no	0.99	no	down	4.6E-01	no
ENSRNOT00000077671 : Kcnma1 : A0A0G2K104	4.06	yes	up	2.6E-13	yes	chr15	intron	internal intron	NA	1.01	no	up	0.53	no	0.89	no	down	7.4E-06	yes
MSTRG.4991.1 : Ppm1f : Q9WVR7	1.80	yes	up	3.8E-11	yes	chr11	intron	internal intron	NA	0.65	no	down	0.15	no	0.91	no	down	2.5E-06	yes
MSTRG.3981.1 : Becn1 : Q91XJ1	0.15	yes	down	1.2E-04	yes	chr10	exon	first exon	NA	0.79	no	down	0.72	no	0.97	no	down	9.2E-02	no
MSTRG.10830.2 : Phc3 : D3ZS50	0.36	yes	down	4.0E-04	yes	chr2	intron	internal intron	NA	0.74	no	down	0.52	no	0.86	no	down	4.2E-04	yes
ENSRNOT00000093592 : Eftud2 : F1LM66	4.66	yes	up	6.0E-04	yes	chr10	exon	internal exon	NA	0.98	no	down	0.64	no	1.02	no	up	4.9E-02	yes
MSTRG.2839.7 : Srrm2 : A0A0G2K2M9	1.72	yes	up	9.0E-05	yes	chr10	exon	internal exon	NA	1.00	no	down	1.00	no	1.00	no	up	9.0E-01	no
MSTRG.1463.1 : Stim1 : P84903	2.61	yes	up	1.3E-04	yes	chr1	exon	last exon	NA	1.28	no	up	0.37	no	0.92	no	down	3.4E-08	yes
MSTRG.5778.3 : Pign : E9PTA5	0.23	yes	down	2.2E-04	yes	chr13	intron	internal intron	NA	1.22	no	up	0.50	no	1.05	no	up	3.6E-03	yes
ENSRNOT00000084782 : Mat2a : F1LRB8	0.10	yes	down	1.4E-04	yes	chr4	exon	last exon	NA	1.16	no	up	0.14	no	0.99	no	down	1.5E-01	no
MSTRG.11285.4 : Npr1 : P18910	0.37	yes	down	1.2E-04	yes	chr2	intron	internal intron	NA	0.79	no	down	0.17	no	1.02	no	up	1.6E-02	yes
MSTRG.4716.2 : Adcy5 : G3V9G1	0.16	yes	down	2.3E-10	yes	chr11	intron	internal intron	NA	1.81	no	up	0.34	no	1.00	no	down	7.6E-01	no
MSTRG.10605.1 : Nnt : Q5BJZ3	3.00	yes	up	1.8E-04	yes	chr2	intron	internal intron	NA	0.81	no	down	0.50	no	0.96	no	down	1.2E-07	yes
MSTRG.8033.4 : Slc27a1 : Q6GMM8	0.26	yes	down	5.6E-15	yes	chr16	intron	internal intron	NA	1.44	no	up	0.47	no	0.99	no	down	2.5E-01	no
MSTRG.10871.1 : Dcun1d1 : D3ZRV0	1.60	yes	up	3.7E-04	yes	chr2	exon	internal exon	NA	0.80	no	down	0.57	no	0.97	no	down	2.6E-02	yes
MSTRG.1731.3 : Tbc1d10b : D3ZSY8	1.59	yes	up	2.7E-04	yes	chr1	intron	internal intron	NA	2.29	yes	up	0.08	no	0.99	no	down	3.1E-01	no
ENSRNOT00000037456 : Notch3 : F1LQX7	0.61	yes	down	2.1E-04	yes	chr7	exon	internal exon	NA	1.01	no	up	0.91	no	0.96	no	down	9.5E-04	yes
ENSRNOT00000022462 : Mtpap : D3ZPN5	1.99	yes	up	1.4E-05	yes	chr17	intron	internal intron	NA	1.16	no	up	0.09	no	1.03	no	up	1.2E-01	no
MSTRG.6855.5 : Prom1 : Q9JI49	8.32	yes	up	2.4E-09	yes	chr14	intron	internal intron	NA	0.87	no	down	0.74	no	0.87	no	down	1.5E-05	yes
ENSRNOT00000089149 : LOC108348175 : A0A0G2	0.05	yes	down	4.0E-19	yes	chr1	promoter	Intermediate	LCP	1.24	no	up	0.22	no	1.01	no	up	4.0E-01	no
ENSRNOT00000084651 : P3h1 : Q9R1J8	0.24	yes	down	1.5E-05	yes	chr5	intron	internal intron	NA	1.27	no	up	0.09	no	1.16	no	up	6.3E-15	yes
MSTRG.11265.28 : Ubap2l : E9PTR4	6.62	yes	up	4.0E-04	yes	chr2	intron	internal intron	NA	0.92	no	down	0.39	no	1.13	no	up	3.8E-12	yes

MSTRG.21039.12 : Tns1 : F1LN42	0.09	yes	down	2.0E-04	yes	chr9	intron	last intron	NA	0.58	no	down	0.40	no	0.86	no	down	7.8E-19	yes
MSTRG.12137.2 : Pknox1 : Q5BJP1	0.26	yes	down	3.4E-07	yes	chr20	promoter	Proximal	LCP	1.25	no	up	0.72	no	0.89	no	down	7.5E-04	yes
MSTRG.21083.7 : Speg : Q63638	0.17	yes	down	2.6E-06	yes	chr9	intron	internal intron	NA	0.70	no	down	0.58	no	0.88	no	down	2.3E-07	yes
MSTRG.11265.16 : Ubap2l : E9PTR4	6.62	yes	up	4.0E-04	yes	chr2	intron	internal intron	NA	2.25	yes	up	0.02	yes	1.13	no	up	3.8E-12	yes
MSTRG.9268.4 : Lims2 : A0A0G2KAE1	0.67	yes	down	6.6E-13	yes	chr18	intron	internal intron	NA	0.60	no	down	0.08	no	0.82	yes	down	4.4E-10	yes
ENSRNOT00000078073 : Gls : A0A0G2KAN7	0.05	yes	down	1.5E-13	yes	chr9	intron	first intron	NA	0.34	yes	down	0.01	yes	1.09	no	up	8.3E-03	yes
MSTRG.16461.10 : Pum1 : D3Z8L5	0.44	yes	down	3.1E-04	yes	chr5	intron	internal intron	NA	0.55	no	down	0.04	yes	1.03	no	up	7.0E-02	no
MSTRG.21039.13 : Tns1 : F1LN42	0.09	yes	down	2.0E-04	yes	chr9	intron	last intron	NA	1.11	no	up	0.86	no	0.86	no	down	7.8E-19	yes
MSTRG.9111.1 : Bmi1 : B4F7B6	2.46	yes	up	2.3E-04	yes	chr17	exon	last exon	NA	4.45	yes	up	0.33	no	0.98	no	down	3.6E-01	no
ENSRNOT00000003077 : Pdgfra : G3V6A0	0.22	yes	down	1.6E-04	yes	chr14	intron	last intron	NA	1.19	no	up	0.38	no	1.02	no	up	4.1E-02	yes
ENSRNOT00000092769 : Sema4a : A0A1B0GWV9	3.09	yes	up	1.9E-04	yes	chr2	promoter	Intermediate	LCP	1.11	no	up	0.34	no	1.07	no	up	2.4E-05	yes
ENSRNOT00000028856 : Hspa12b : D3ZVM5	0.65	yes	down	3.5E-04	yes	chr3	intron	last intron	NA	0.86	no	down	0.67	no	0.86	no	down	5.9E-13	yes
ENSRNOT00000092395 : Mapk10 : A0A0U1RRS7	2.61	yes	up	3.6E-06	yes	chr14	intron	first intron	NA	0.99	no	down	0.45	no	0.56	yes	down	6.7E-04	yes
MSTRG.11369.2 : Vps45 : O08700	3.11	yes	up	8.5E-06	yes	chr2	intron	internal intron	NA	1.09	no	up	0.72	no	0.99	no	down	3.7E-01	no
MSTRG.6560.1 : Hnrnpdl : A0A0G2KAZ7	inf	yes	up	7.6E-05	yes	chr14	intron	first intron	NA	0.92	no	down	0.64	no	0.99	no	down	4.6E-01	no
ENSRNOT00000045400 : Macf1 : A0A0G2K9T4	0.36	yes	down	1.3E-05	yes	chr5	exon	last exon	NA	1.17	no	up	0.41	no	0.97	no	down	9.0E-11	yes
ENSRNOT00000025281 : Tomm40 : G3V8F5	0.23	yes	down	3.2E-04	yes	chr1	promoter	Distal	LCP	1.34	no	up	0.77	no	1.06	no	up	1.1E-05	yes
ENSRNOT00000079069 : Bclaf1 : B1WC16	0.67	yes	down	1.3E-07	yes	chr1	intron	internal intron	NA	1.41	no	up	0.06	no	1.03	no	up	6.8E-02	no
ENSRNOT00000089338 : Lrba : A0A0G2JYI0	10.95	yes	up	4.8E-12	yes	chr2	intron	internal intron	NA	0.99	no	down	0.94	no	0.94	no	down	4.5E-07	yes
ENSRNOT00000004293 : Acbd3 : G3V6E4	2.42	yes	up	1.4E-04	yes	chr13	intron	internal intron	NA	1.09	no	up	0.40	no	1.07	no	up	2.4E-14	yes
MSTRG.19070.1 : Rrp7a : D4AE65	1.73	yes	up	1.3E-04	yes	chr7	intron	internal intron	NA	0.70	no	down	0.46	no	0.94	no	down	1.5E-02	yes
ENSRNOT00000054980 : Tgfb1i1 : Q99PD6	3.08	yes	up	7.2E-05	yes	chr1	intron	internal intron	NA	0.80	no	down	0.67	no	1.06	no	up	1.6E-04	yes
ENSRNOT00000084109 : Macf1 : A0A0G2K9T4	0.36	yes	down	1.3E-05	yes	chr5	exon	last exon	NA	0.99	no	down	0.95	no	0.97	no	down	9.0E-11	yes
ENSRNOT00000093459 : Ndrg1 : Q6JE36	0.37	yes	down	7.6E-09	yes	chr7	intron	internal intron	NA	0.98	no	down	0.40	no	0.92	no	down	4.9E-08	yes
ENSRNOT00000008988 : Atl2 : F1LQ09	2.32	yes	up	2.9E-04	yes	chr6	intron	last intron	NA	0.99	no	down	0.74	no	0.95	no	down	8.9E-05	yes
ENSRNOT00000080166 : Tinagl1 : Q4V8N0	0.10	yes	down	7.3E-25	yes	chr5	promoter	Distal	LCP	1.76	no	up	0.34	no	0.89	no	down	4.1E-08	yes
MSTRG.16557.6 : Srrm1 : B2RYB3	2.05	yes	up	2.0E-04	yes	chr5	intron	internal intron	NA	0.85	no	down	0.63	no	1.10	no	up	2.8E-06	yes
MSTRG.21176.11 : Inpp5d : F1M981	0.08	yes	down	9.9E-05	yes	chr9	intron	internal intron	NA	0.53	no	down	0.01	yes	1.08	no	up	3.1E-05	yes
MSTRG.5482.22 : Ncor2 : A0A0G2JU91	2.16	yes	up	1.6E-06	yes	chr12	intron	internal intron	NA	0.93	no	down	0.45	no	1.01	no	up	7.1E-01	no
MSTRG.15963.2 : Ptprd : M0RB22	3.73	yes	up	1.3E-08	yes	chr5	exon	internal exon	NA	0.76	no	down	0.68	no	1.01	no	up	5.3E-01	no
ENSRNOT00000010811 : Ndrg1 : Q6JE36	0.37	yes	down	7.6E-09	yes	chr7	intron	internal intron	NA	1.43	no	up	0.54	no	0.92	no	down	4.9E-08	yes
MSTRG.4263.2 : Tnrc6c : D3ZRA6	0.39	yes	down	2.3E-06	yes	chr10	intron	internal intron	NA	1.23	no	up	0.67	no	1.07	no	up	1.9E-01	no
MSTRG.5897.2 : Cntn2 : G3V758	2.37	yes	up	8.3E-06	yes	chr13	exon	internal exon	NA	0.96	no	down	0.91	no	1.00	no	down	9.1E-01	no
MSTRG.19148.6 : Shank3 : A0A0U1RS13	3.23	yes	up	4.3E-05	yes	chr7	exon	first exon	NA	0.86	no	down	0.49	no	0.98	no	down	1.0E-02	yes
MSTRG.16755.6 : Kif1b : A0A0G2KA12	3.65	yes	up	7.4E-05	yes	chr5	intron	internal intron	NA	0.59	no	down	0.43	no	1.05	no	up	6.5E-02	no
ENSRNOT00000029076 : Tpm3 : A0A140TAF0	0.37	yes	down	8.0E-05	yes	chr2	intron	internal intron	NA	1.07	no	up	0.55	no	1.03	no	up	4.4E-04	yes
ENSRNOT00000027351 : Nt5c2 : D3ZMY7	17.59	yes	up	5.0E-22	yes	chr1	exon	last exon	NA	0.76	no	down	0.20	no	0.92	no	down	2.5E-12	yes
MSTRG.2579.6 : Sfxn3 : Q6P6T0	0.39	yes	down	7.8E-05	yes	chr1	intron	internal intron	NA	0.62	no	down	0.36	no	1.12	no	up	6.4E-07	yes
MSTRG.18268.19 : Akap8 : Q63014	0.08	yes	down	2.7E-04	yes	chr7	intron	internal intron	NA	1.07	no	up	0.79	no	0.99	no	down	2.8E-01	no
ENSRNOT00000039663 : Ubr4 : A0A0G2JU89	2.84	yes	up	3.7E-05	yes	chr5	intron	internal intron	NA	0.94	no	down	0.40	no	1.02	no	up	4.6E-05	yes
MSTRG.8552.4 : Ddx46 : Q62780	0.56	yes	down	3.4E-04	yes	chr17	intron	internal intron	NA	0.93	no	down	0.50	no	0.98	no	down	1.3E-01	no
MSTRG.16008.1 : Dennd4c : F1LTD7	0.31	yes	down	2.0E-04	yes	chr5	intron	internal intron	NA	1.64	no	up	0.65	no	0.97	no	down	9.0E-02	no
MSTRG.16592.1 : Kdm1a : A0A0G2K736	0.26	yes	down	3.7E-04	yes	chr5	intron	internal intron	NA	1.16	no	up	0.56	no	1.01	no	up	5.0E-01	no
ENSRNOT00000014533 : Tifa : Q5XIB9	0.42	yes	down	9.5E-05	yes	chr2	exon	internal exon	NA	1.38	no	up	0.12	no	1.16	no	up	2.8E-04	yes
MSTRG.2622.2 : Nt5c2 : D3ZMY7	17.59	yes	up	5.0E-22	yes	chr1	exon	last exon	NA	0.88	no	down	0.32	no	0.92	no	down	2.5E-12	yes
MSTRG.8278.5 : Rbpms : F2Z3S5	0.02	yes	down	2.6E-05	yes	chr16	promoter	Proximal	ICP	1.30	no	up	0.57	no	1.06	no	up	4.9E-05	yes
MSTRG.1783.7 : Tgfb1i1 : Q99PD6	3.08	yes	up	7.2E-05	yes	chr1	intron	internal intron	NA	1.06	no	up	0.82	no	1.06	no	up	1.6E-04	yes
ENSRNOT00000000814 : Ptprc : P04157	2.98	yes	up	6.9E-14	yes	chr13	intron	internal intron	NA	0.86	no	down	0.83	no	1.12	no	up	6.1E-15	yes
MSTRG.21175.3 : Inpp5d : F1M981	0.08	yes	down	9.9E-05	yes	chr9	intron	internal intron	NA	0.86	no	down	0.53	no	1.08	no	up	3.1E-05	yes
MSTRG.19188.4 : Rpap3 : Q68FQ7	0.29	yes	down	4.9E-05	yes	chr7	intron	internal intron	NA	0.97	no	down	0.90	no	1.04	no	up	2.9E-04	yes
MSTRG.2881.1 : Pkd1 : Q9ERV0	inf	yes	up	6.6E-05	yes	chr10	intron	internal intron	NA	0.87	no	down	0.84	no	1.19	no	up	9.8E-07	yes
MSTRG.14695.6 : Ptcd3 : D3ZGM1	3.88	yes	up	4.1E-35	yes	chr4	intron	internal intron	NA	1.04	no	up	0.94	no	0.98	no	down	3.1E-01	no
MSTRG.14881.3 : Aplf : F6Q5G6	0.12	yes	down	8.1E-06	yes	chr4	exon	last exon	NA	1.05	no	up	0.76	no	0.99	no	down	2.8E-01	no
MSTRG.10689.1 : Cdh6 : F1LQP8	3.77	yes	up	4.7E-05	yes	chr2	intron	internal intron	NA	1.44	no	up	0.14	no	1.08	no	up	1.9E-09	yes
MSTRG.16771.4 : H6pd : D4A7D7	0.22	yes	down	2.6E-06	yes	chr5	intron	last intron	NA	0.58	no	down	0.59	no	1.14	no	up	9.6E-15	yes
MSTRG.20266.1 : Dag1 : F1M8K0	2.95	yes	up	1.4E-04	yes	chr8	exon	first exon	NA	1.01	no	up	0.20	no	0.91	no	down	7.5E-12	yes
MSTRG.5958.3 : Csrp1 : P47875	1.98	yes	up	3.9E-04	yes	chr13	promoter	Intermediate	LCP	8.12	yes	up	0.12	no	1.11	no	up	3.1E-08	yes
MSTRG.20927.7 : Aox1 : F1LRQ1	0.36	yes	down	1.5E-07	yes	chr9	intron	internal intron	NA	1.37	no	up	0.03	yes	0.73	yes	down	3.5E-17	yes
MSTRG.4030.3 : Eftud2 : F1LM66	4.66	yes	up	6.0E-04	yes	chr10	exon	internal exon	NA	0.76	no	down	0.23	no	1.02	no	up	4.9E-02	yes
MSTRG.3421.1 : Nup88 : O08658	0.25	yes	down	1.2E-04	yes	chr10	intron	internal intron	NA	0.79	no	down	0.51	no	0.95	no	down	3.4E-05	yes
MSTRG.21427.1 : Rbm3 : G3V6P6	5.50	yes	up	1.2E-04	yes	chrX	intron	first intron	NA	0.87	no	down	0.69	no	0.93	no	down	8.6E-07	yes
MSTRG.17687.1 : Lgmn : Q5PPG2	2.26	yes	up	3.7E-04	yes	chr6	intron	internal intron	NA	1.13	no	up	0.87	no	1.15	no	up	3.3E-08	yes
MSTRG.19968.1 : Myzap : Q5EB94	0.16	yes	down	3.6E-06	yes	chr8	exon	last exon	NA	1.08	no	up	0.68	no	0.84	no	down	4.4E-10	yes
ENSRNOT00000043907 : Dennd1b : F1M3B0	0.50	yes	down	5.1E-04	yes	chr13	promoter	Distal	HCP	1.07	no	up	0.58	no	0.99	no	down	8.0E-01	no
MSTRG.19125.1 : Brd1 : D3ZUW8	7.17	yes	up	3.3E-04	yes	chr7	exon	internal exon	NA	0.94	no	down	0.96	no	1.21	yes	up	6.4E-02	no
ENSRNOT00000003224 : Sgcd : F1LYS7	0.12	yes	down	5.4E-11	yes	chr10	intron	internal intron	NA	1.27	no	up	0.08	no	0.81	yes	down	2.9E-17	yes
ENSRNOT00000017265 : Rprd1b : B5DEK0	0.05	yes	down	1.1E-04	yes	chr3	intron	last intron	NA	1.02	no	up	0.94	no	0.98	no	down	1.8E-03	yes
MSTRG.6211.6 : Fcgr2b : A3RLA8	0.44	yes	down	2.8E-04	yes	chr13	promoter	Distal	LCP	0.95	no	down	0.89	no	1.04	no	up	4.3E-03	yes
ENSRNOT00000042890 : Opcml : F1M2I5	2.41	yes	up	1.6E-04	yes	chr8	intron	last intron	NA	1.02	no	up	0.91	no	0.91	no	down	2.1E-15	yes
MSTRG.10872.2 : Mccc1 : F1LP30	0.34	yes	down	1.3E-04	yes	chr2	intron	internal intron	NA	1.03	no	up	0.47	no	0.96	no	down	1.3E-08	yes
ENSRNOT00000083942 : Unc93b1 : D3ZDJ4	3.27	yes	up	6.0E-05	yes	chr1	intron	internal intron	NA	1.00	no	down	0.99	no	1.19	no	up	9.2E-05	yes
MSTRG.16609.6 : Ece1 : Q6IN10	0.58	yes	down	9.3E-05	yes	chr5	intron	internal intron	NA	0.19	yes	down	0.30	no	1.14	no	up	8.3E-15	yes
MSTRG.1714.1 : Aldoa : P05065	0.17	yes	down	1.4E-04	yes	chr1	exon	last exon	NA	2.81	yes	up	0.32	no	0.98	no	down	4.2E-08	yes
MSTRG.20817.8 : Map4k4 : A0A0G2K7W4	0.36	yes	down	7.1E-05	yes	chr9	intron	internal intron	NA	1.02	no	up	0.95	no	1.10	no	up	7.1E-06	yes
MSTRG.12668.3 : Nup188 : F1LRC6	0.22	yes	down	2.3E-05	yes	chr3	exon	internal exon	NA	0.61	no	down	0.50	no	1.02	no	up	1.9E-01	no
MSTRG.19928.3 : Herc1 : A0A0G2JTT6	10.78	yes	up	2.7E-04	yes	chr8	intron	internal intron	NA	0.66	no	down	0.50	no	1.04	no	up	5.4E-02	no
ENSRNOT00000015077 : Papss1 : A0A0G2K0L0	15.84	yes	up	7.3E-05	yes	chr2	promoter	Intermediate	LCP	1.03	no	up	0.81	no	1.04	no	up	5.2E-04	yes
ENSRNOT00000087615 : Ddx39b : Q63413	0.02	yes	down	4.7E-11	yes	chr20	exon	last exon	NA	1.01	no	up	0.99	no	1.01	no	up	2.0E-01	no
ENSRNOT00000079655 : Prmt3 : O70467	1.85	yes	up	1.1E-04	yes	chr1	intron	internal intron	NA	0.98	no	down	0.91	no	1.06	no	up	2.2E-04	yes
MSTRG.17473.1 : Sptb : Q6XDA0	0.47	yes	down	3.3E-04	yes	chr6	intron	internal intron	NA	0.81	no	down	0.45	no	0.94	no	down	2.4E-06	yes
MSTRG.13515.1 : Anapc1 : F1M801	0.25	yes	down	6.5E-11	yes	chr3	intron	internal intron	NA	1.28	no	up	0.11	no	1.00	no	down	5.4E-01	no
MSTRG.9889.3 : Nf1x : F2Z3R4	0.58	yes	down	1.9E-06	yes	chr19	intron	internal intron	NA	0.93	no	down	0.83	no	0.77	yes	down	2.4E-14	yes
MSTRG.9889.6 : Nf1x : F2Z3R4	0.58	yes	down	1.9E-06	yes	chr19	intron	internal intron	NA	0.81	no	down	0.50	no	0.77	yes	down	2.4E-14	yes
MSTRG.11953.36 : RT1-CE7 : D3ZLE6	inf	yes	up	7.3E-07	yes	chr20	exon	internal exon	NA	0.96	no	down	0.94	no	1.22	yes	up	7.3E-16	yes
ENSRNOT00000030518 : Snx17 : Q6AYS6	0.27	yes	down	1.2E-11	yes	chr6	intron	internal intron	NA	0.88	no	down	0.30	no	0.99	no	down	1.5E-01	no
MSTRG.4725.12 : Kalrn : P97924	1.54	yes	up	9.1E-07	yes	chr11	exon	last exon	NA	1.32	no	up	0.44	no	1.04	no	up	1.6E-04	yes
MSTRG.15397.4 : Stk38l : A4GW50	0.61	yes	down	1.5E-06	yes	chr4	intron	internal intron	NA	1.00	no	down	0.47	no	1.16	no	up	1.1E-11	yes
ENSRNOT00000031115 : Uba1 : Q5U300	0	yes	down	1.9E-08	yes	chrX	exon	last exon	NA	1.07	no	up	0.43	no	0.93	no	down	2.8E-14	yes
MSTRG.19538.14 : Nfrkb : D4A421	0.58	yes	down	1.3E-05	yes	chr8	intron	internal intron	NA	1.05	no	up	0.80	no	0.89	no	down	2.5E-03	yes
MSTRG.986.4 : Akt1s1 : D3ZH75	2.93	yes	up	2.4E-05	yes	chr1	intron	internal intron	NA	0.99	no	down	0.97	no	0.97	no	down	7.2E-01	no
MSTRG.10878.2 : Anxa5 : P14668	3.34	yes	up	1.2E-08	yes	chr2	promoter	Distal	LCP	0.82	no	down	0.91	no	0.88	no	down	2.2E-14	yes
ENSRNOT00000086046 : Dnajc5 : A0A0G2JX56	0.30	yes	down	1.1E-06	yes	chr3	promoter	Intermediate	HCP	1.22	no	up	0.30	no	0.89	no	down	4.0E-08	yes
MSTRG.13870.1 : Plcg1 : G3V845	0.24	yes	down	1.1E-04	yes	chr3	intron	first intron	NA	0.78	no	down	0.55	no	0.94	no	down	1.8E-09	yes
ENSRNOT00000088264 : Flnb : D4A8D5	1.70	yes	up	8.8E-05	yes	chr15	intron	last intron	NA	0.85	no	down	0.35	no	1.00	no	up	2.4E-01	no
MSTRG.7319.2 : Psmc6 : Q32PW9	2.30	yes	up	1.2E-05	yes	chr15	intron	internal intron	NA	1.26	no	up	0.21	no	1.02	no	up	3.7E-03	yes
MSTRG.10428.1 : Scamp1 : A0A0G2K1I6	11.34	yes	up	2.9E-04	yes	chr2	intron	first intron	NA	0.93	no	down	0.79	no	1.05	no	up	3.2E-06	yes
ENSRNOT00000088188 : Lrba : A0A0G2JYI0	10.95	yes	up	4.8E-12	yes	chr2	intron	internal intron	NA	0.98	no	down	0.89	no	0.94	no	down	4.5E-07	yes
MSTRG.5035.6 : Arhgef18 : F1LT94	0.62	yes	down	4.6E-05	yes	chr12	exon	internal exon	NA	1.26	no	up	0.09	no	0.93	no	down	5.9E-05	yes
MSTRG.14960.6 : Eogt : Q5NDL0	6.71	yes	up	1.7E-04	yes	chr4	intron	internal intron	NA	1.33	no	up	0.29	no	1.10	no	up	2.0E-05	yes
MSTRG.986.2 : Akt1s1 : D3ZH75	2.93	yes	up	2.4E-05	yes	chr1	intron	internal intron	NA	1.10	no	up	0.76	no	0.97	no	down	7.2E-01	no

ENSRNOT00000092043 : Unkl : D4A3S7	2.08	yes	up	2.1E-04	yes	chr10	intron	internal intron	NA	0.93	no	down	0.32	no	0.84	no	down	1.1E-15	yes
MSTRG.3645.12 : Slfn13 : A0A096MKD0	2.27	yes	up	1.5E-04	yes	chr10	promoter	Intermediate	LCP	1.30	no	up	0.53	no	0.97	no	down	2.7E-02	yes
ENSRNOT00000083484 : B3glct : A0A0G2JUT7	0.28	yes	down	9.0E-07	yes	chr12	intron	internal intron	NA	0.44	yes	down	0.31	no	0.86	no	down	1.3E-06	yes
MSTRG.14075.1 : Dnajc5 : A0A0G2JX56	0.30	yes	down	1.1E-06	yes	chr3	promoter	Intermediate	HCP	0.46	yes	down	0.18	no	0.89	no	down	4.0E-08	yes
ENSRNOT00000029166 : Alg9 : D3ZCW5	0.10	yes	down	9.3E-08	yes	chr8	intron	first intron	NA	1.16	no	up	0.42	no	1.11	no	up	6.0E-03	yes
MSTRG.20284.1 : Qrich1 : F1M4M7	3.68	yes	up	3.4E-07	yes	chr8	exon	internal exon	NA	1.71	no	up	0.24	no	1.03	no	up	2.0E-01	no
ENSRNOT00000045373 : Tram1 : Q5XI41	3.29	yes	up	2.9E-05	yes	chr5	exon	last exon	NA	0.97	no	down	0.47	no	1.13	no	up	1.2E-09	yes
ENSRNOT00000038566 : Cped1 : A0A0G2QC10	1.61	yes	up	4.5E-04	yes	chr4	intron	internal intron	NA	1.46	no	up	0.54	no	1.07	no	up	1.5E-03	yes
ENSRNOT00000035309 : Heatr5a : F1LSK5	0.05	yes	down	7.6E-05	yes	chr6	intron	internal intron	NA	0.64	no	down	0.04	yes	0.98	no	down	2.4E-01	no
MSTRG.8351.10 : Adam9 : E9PTA4	0.15	yes	down	9.7E-05	yes	chr16	intron	internal intron	NA	1.14	no	up	0.75	no	1.06	no	up	3.4E-02	yes
MSTRG.12896.4 : Rabgap1 : D3ZX42	3.82	yes	up	8.1E-06	yes	chr3	intron	internal intron	NA	1.37	no	up	0.39	no	1.01	no	up	1.9E-01	no
ENSRNOT00000085126 : Vcl : P85972	0.35	yes	down	3.5E-07	yes	chr15	intron	internal intron	NA	0.84	no	down	0.51	no	0.88	no	down	2.8E-16	yes
MSTRG.9889.11 : Nf1x : F2Z3R4	0.58	yes	down	1.9E-06	yes	chr19	intron	internal intron	NA	0.68	no	down	0.45	no	0.77	yes	down	2.4E-14	yes
ENSRNOT00000085390 : Acbd3 : G3V6E4	2.42	yes	up	1.4E-04	yes	chr13	intron	internal intron	NA	1.04	no	up	0.16	no	1.07	no	up	2.4E-14	yes
MSTRG.20323.2 : Ptpn23 : F1M951	1.59	yes	up	2.2E-04	yes	chr8	exon	internal exon	NA	0.66	no	down	0.09	no	0.98	no	down	1.3E-03	yes
MSTRG.11953.33 : RT1-CE7 : D3ZLE6	inf	yes	up	7.3E-07	yes	chr20	exon	internal exon	NA	1.83	no	up	0.06	no	1.22	yes	up	7.3E-16	yes
MSTRG.13870.10 : Plcg1 : G3V845	0.24	yes	down	1.1E-04	yes	chr3	intron	first intron	NA	1.00	no	down	0.93	no	0.94	no	down	1.8E-09	yes
ENSRNOT00000080788 : Wnk1 : Q9JIH7	18.77	yes	up	1.6E-38	yes	chr4	exon	internal exon	NA	0.83	no	down	0.60	no	1.04	no	up	1.7E-03	yes
ENSRNOT00000076030 : Parp4 : A0A096MJ98	1.23	yes	up	1.1E-06	yes	chr15	intron	last intron	NA	0.62	no	down	0.14	no	1.00	no	down	9.9E-01	no
ENSRNOT00000019359 : Smchd1 : D4AAG8	0.20	yes	down	3.0E-04	yes	chr9	intron	internal intron	NA	1.12	no	up	0.25	no	0.97	no	down	2.5E-04	yes
MSTRG.4894.1 : Yeats2 : D3ZBV9	4.61	yes	up	5.1E-05	yes	chr11	intron	internal intron	NA	0.95	no	down	0.64	no	1.11	no	up	2.4E-05	yes
MSTRG.1201.7 : Ntrk3 : Q68G04	7.03	yes	up	1.5E-06	yes	chr1	intron	internal intron	NA	1.06	no	up	0.94	no	0.87	no	down	1.8E-07	yes
ENSRNOT00000077301 : Atp5d : G3V7Y3	0.12	yes	down	5.2E-08	yes	chr7	promoter	Distal	LCP	1.06	no	up	0.83	no	0.90	no	down	7.4E-12	yes
ENSRNOT00000082271 : Plec : Q6S395	0.33	yes	down	4.2E-06	yes	chr7	exon	last exon	NA	1.04	no	up	0.53	no	1.03	no	up	2.2E-01	no
MSTRG.19148.11 : Shank3 : A0A0U1RS13	3.23	yes	up	4.3E-05	yes	chr7	exon	first exon	NA	0.54	no	down	0.30	no	0.98	no	down	1.0E-02	yes
ENSRNOT00000007367 : Rbm3 : G3V6P6	5.50	yes	up	1.2E-04	yes	chrX	intron	first intron	NA	0.51	no	down	0.21	no	0.93	no	down	8.6E-07	yes
ENSRNOT00000083354 : Kmt2d : A0A0G2JVD6	6.53	yes	up	2.1E-09	yes	chr7	exon	last exon	NA	0.86	no	down	0.91	no	1.03	no	up	3.5E-02	yes
MSTRG.16609.2 : Ece1 : Q6IN10	0.58	yes	down	9.3E-05	yes	chr5	intron	internal intron	NA	0.89	no	down	0.95	no	1.14	no	up	8.3E-15	yes
ENSRNOT00000087181 : Fkbp11 : G3V7V5	inf	yes	up	4.7E-06	yes	chr7	intron	internal intron	NA	0.73	no	down	0.23	no	1.06	no	up	1.6E-07	yes
MSTRG.11091.6 : Gucy1a3 : P19686	0.26	yes	down	3.9E-06	yes	chr2	intron	internal intron	NA	2.33	yes	up	0.05	yes	0.85	no	down	1.4E-12	yes
MSTRG.2579.5 : Sfxn3 : Q6P6T0	0.39	yes	down	7.8E-05	yes	chr1	intron	internal intron	NA	0.48	yes	down	0.08	no	1.12	no	up	6.4E-07	yes
ENSRNOT00000093605 : Eftud2 : F1LM66	4.66	yes	up	6.0E-04	yes	chr10	exon	internal exon	NA	0.86	no	down	0.16	no	1.02	no	up	4.9E-02	yes
MSTRG.12973.10 : Rbms1 : A0A0G2K4R7	0	yes	down	3.3E-05	yes	chr3	intron	internal intron	NA	1.27	no	up	0.14	no	0.97	no	down	1.2E-01	no
MSTRG.12240.2 : Bicc1 : A0A0G2K0Y0	5.19	yes	up	5.0E-04	yes	chr20	intron	internal intron	NA	0.69	no	down	0.68	no	1.52	yes	up	1.2E-05	yes
MSTRG.3645.3 : Slfn13 : A0A096MKD0	2.27	yes	up	1.5E-04	yes	chr10	promoter	Intermediate	LCP	0.81	no	down	0.72	no	0.97	no	down	2.7E-02	yes
ENSRNOT00000092881 : Gtf2i : Q5U2Y1	5.31	yes	up	4.9E-06	yes	chr12	promoter	Distal	LCP	0.99	no	down	0.72	no	0.95	no	down	1.5E-07	yes
MSTRG.15361.1 : Recql : Q6AYJ1	0.74	yes	down	1.2E-04	yes	chr4	exon	last exon	NA	0.78	no	down	0.44	no	0.97	no	down	6.1E-03	yes
MSTRG.7731.4 : Tsc22d1 : P62501	3.00	yes	up	3.0E-04	yes	chr15	promoter	Intermediate	ICP	1.01	no	up	0.97	no	0.77	yes	down	5.9E-09	yes
MSTRG.11265.11 : Ubap2l : E9PTR4	6.62	yes	up	4.0E-04	yes	chr2	intron	internal intron	NA	1.27	no	up	0.34	no	1.13	no	up	3.8E-12	yes
MSTRG.5159.1 : Tecpr1 : Q3ZBA0	0.43	yes	down	3.1E-04	yes	chr12	promoter	Intermediate	LCP	1.32	no	up	0.21	no	1.17	no	up	7.7E-08	yes
MSTRG.17567.3 : Elmsan1 : D4ACA6	0.27	yes	down	2.9E-04	yes	chr6	exon	last exon	NA	1.35	no	up	0.09	no	0.96	no	down	1.9E-01	no
MSTRG.4485.1 : Runx1 : Q63046	0.62	yes	down	5.4E-05	yes	chr11	intron	first intron	NA	1.48	no	up	0.30	no	1.47	yes	up	3.2E-10	yes
ENSRNOT00000046920 : Ttc28 : D3ZXP1	4.41	yes	up	1.9E-04	yes	chr12	promoter	Intermediate	LCP	1.22	no	up	0.02	yes	0.86	no	down	6.9E-05	yes
ENSRNOT00000022594 : Pltp : E9PSP1	0.31	yes	down	3.6E-05	yes	chr3	intron	internal intron	NA	0.98	no	down	0.91	no	0.93	no	down	1.9E-11	yes
ENSRNOT00000024493 : Tpm1 : Q923Z2	9.41	yes	up	5.1E-14	yes	chr8	intron	internal intron	NA	0.72	no	down	0.44	no	0.75	yes	down	6.0E-18	yes
MSTRG.21176.5 : Inpp5d : F1M981	0.08	yes	down	9.9E-05	yes	chr9	intron	internal intron	NA	0.75	no	down	0.22	no	1.08	no	up	3.1E-05	yes
MSTRG.19819.1 : Neo1 : P97603	0	yes	down	3.5E-04	yes	chr8	promoter	Intermediate	LCP	0.50	yes	down	0.50	no	0.98	no	down	1.8E-02	yes
ENSRNOT00000024718 : Dhx58 : D3ZD46	5.00	yes	up	2.1E-04	yes	chr10	exon	internal exon	NA	1.14	no	up	0.73	no	1.22	yes	up	6.3E-08	yes
ENSRNOT00000093467 : Atp6v1a : D4A133	0.36	yes	down	7.3E-06	yes	chr11	intron	internal intron	NA	1.31	no	up	0.48	no	1.08	no	up	1.1E-08	yes
ENSRNOT00000084560 : Atad2b : A0A096MKA5	3.51	yes	up	9.0E-07	yes	chr6	intron	internal intron	NA	0.95	no	down	0.86	no	1.15	no	up	9.1E-06	yes
MSTRG.17520.1 : Srsf5 : Q09167	0.10	yes	down	3.0E-09	yes	chr6	promoter	Proximal	ICP	2.16	yes	up	0.37	no	1.03	no	up	4.2E-02	yes
ENSRNOT00000015247 : Atp13a1 : B5DEX7	3.83	yes	up	1.1E-13	yes	chr16	intron	internal intron	NA	0.46	yes	down	0.12	no	1.09	no	up	4.2E-12	yes
ENSRNOT00000092978 : Sema4a : A0A1B0GWV9	3.09	yes	up	1.9E-04	yes	chr2	promoter	Intermediate	LCP	1.03	no	up	0.34	no	1.07	no	up	2.4E-05	yes
MSTRG.276.7 : Arid1b : F1LNP1	1.53	yes	up	6.5E-08	yes	chr1	promoter	Proximal	LCP	1.36	no	up	0.57	no	0.98	no	down	1.8E-01	no
MSTRG.12059.5 : Srsf3 : A0A0U1RRV7	4.40	yes	up	1.5E-08	yes	chr20	intron	internal intron	NA	1.69	no	up	0.36	no	0.94	no	down	5.5E-08	yes
MSTRG.5280.3 : Taf6 : Q498R0	0.74	yes	down	2.2E-06	yes	chr12	intron	internal intron	NA	0.79	no	down	0.35	no	0.91	no	down	1.8E-03	yes
MSTRG.3572.3 : RGD1307929 : F1LSX1	7.34	yes	up	1.1E-12	yes	chr10	intron	internal intron	NA	3.07	yes	up	0.03	yes	0.97	no	down	1.8E-04	yes
MSTRG.20501.3 : Trip10 : P97531	2.19	yes	up	4.1E-04	yes	chr9	intron	last intron	NA	0.67	no	down	0.67	no	0.91	no	down	4.8E-08	yes
ENSRNOT00000091470 : Picalm : Q66WT9	8.74	yes	up	2.1E-13	yes	chr1	intron	internal intron	NA	1.31	no	up	0.64	no	1.45	yes	up	1.6E-10	yes
MSTRG.323.7 : LOC108348175 : A0A0G2JUS0	0.05	yes	down	4.0E-19	yes	chr1	promoter	Intermediate	LCP	1.55	no	up	0.17	no	1.01	no	up	4.0E-01	no
MSTRG.1651.1 : Mettl9 : G0Z7P9	inf	yes	up	1.6E-04	yes	chr1	intron	first intron	NA	0.93	no	down	0.63	no	0.99	no	down	7.9E-01	no
ENSRNOT00000092995 : Gtf2i : Q5U2Y1	5.31	yes	up	4.9E-06	yes	chr12	promoter	Distal	LCP	0.92	no	down	0.22	no	0.95	no	down	1.5E-07	yes
MSTRG.20839.7 : Kdelc1 : B5DFA5	5.54	yes	up	1.1E-06	yes	chr9	exon	last exon	NA	0.91	no	down	0.18	no	0.94	no	down	8.4E-02	no
ENSRNOT00000022514 : Efs : B1WBZ1	inf	yes	up	1.8E-04	yes	chr15	promoter	Distal	LCP	1.07	no	up	0.82	no	1.07	no	up	2.0E-01	no
ENSRNOT00000076329 : Synm : G3V9G5	1.68	yes	up	3.0E-04	yes	chr1	exon	last exon	NA	0.97	no	down	0.93	no	0.82	yes	down	8.8E-13	yes
MSTRG.11090.1 : Gucy1a3 : P19686	0.26	yes	down	3.9E-06	yes	chr2	intron	internal intron	NA	0.93	no	down	0.53	no	0.85	no	down	1.4E-12	yes
MSTRG.2788.1 : Hmox2 : P23711	inf	yes	up	5.3E-04	yes	chr10	exon	last exon	NA	1.69	no	up	0.44	no	1.09	no	up	4.1E-06	yes
MSTRG.22041.5 : Dkc1 : P40615	0.28	yes	down	2.4E-05	yes	chrX	intron	internal intron	NA	1.15	no	up	0.20	no	0.99	no	down	3.6E-01	no
ENSRNOT00000056727 : Plscr1 : A0A0G2K7Q1	0.33	yes	down	1.3E-05	yes	chr8	promoter	Distal	LCP	1.28	no	up	0.37	no	1.08	no	up	5.2E-06	yes
MSTRG.6812.7 : Pcdh7 : Q68HB8	0	yes	down	4.2E-04	yes	chr14	exon	last exon	NA	0.99	no	down	0.72	no	0.94	no	down	1.0E-07	yes
MSTRG.15096.2 : Cacna1c : F1MA84	4.75	yes	up	4.3E-04	yes	chr4	intron	internal intron	NA	2.50	yes	up	0.15	no	0.97	no	down	2.7E-02	yes
ENSRNOT00000000134 : Ak2 : A0A0G2JSG6	inf	yes	up	6.9E-05	yes	chr5	exon	first exon	NA	0.90	no	down	0.76	no	0.98	no	down	1.2E-04	yes
MSTRG.7350.1 : Ktn1 : D4A4Z9	2.49	yes	up	1.2E-04	yes	chr15	intron	internal intron	NA	1.62	no	up	0.50	no	1.04	no	up	2.2E-11	yes
ENSRNOT00000017941 : Cmip : A0A0G2JYB9	0.58	yes	down	9.9E-05	yes	chr19	intron	internal intron	NA	0.91	no	down	0.71	no	0.89	no	down	2.1E-04	yes
ENSRNOT00000013050 : Ubp1 : D4A030	0.44	yes	down	2.3E-04	yes	chr8	intron	internal intron	NA	1.86	no	up	0.51	no	0.98	no	down	1.9E-01	no
MSTRG.11265.30 : Ubap2l : E9PTR4	6.62	yes	up	4.0E-04	yes	chr2	intron	internal intron	NA	1.01	no	up	0.97	no	1.13	no	up	3.8E-12	yes
MSTRG.5083.1 : B3glct : A0A0G2JUT7	0.28	yes	down	9.0E-07	yes	chr12	intron	internal intron	NA	3.06	yes	up	0.30	no	0.86	no	down	1.3E-06	yes
MSTRG.332.2 : Fgfr1op : Q4V7C1	7.97	yes	up	2.8E-17	yes	chr1	intron	internal intron	NA	1.40	no	up	0.48	no	1.07	no	up	7.3E-04	yes
MSTRG.5417.1 : Auts2 : F1M388	0.04	yes	down	4.8E-14	yes	chr12	intron	internal intron	NA	1.03	no	up	0.75	no	1.01	no	up	5.6E-01	no
MSTRG.20157.5 : Dzip1l : Q5XIA0	0.67	yes	down	4.1E-05	yes	chr8	intron	internal intron	NA	1.03	no	up	0.83	no	1.09	no	up	1.8E-03	yes
MSTRG.13558.2 : Siglec1 : A0A0G2K320	0.24	yes	down	6.2E-06	yes	chr3	promoter	Proximal	LCP	0.98	no	down	0.92	no	1.25	yes	up	7.1E-13	yes
MSTRG.11964.7 : Ddx39b : Q63413	0.02	yes	down	4.7E-11	yes	chr20	exon	last exon	NA	0.66	no	down	0.58	no	1.01	no	up	2.0E-01	no
ENSRNOT00000080699 : Gyg1 : F8WFR6	0.52	yes	down	3.8E-05	yes	chr2	intron	internal intron	NA	3.13	yes	up	0.02	yes	1.17	no	up	1.9E-09	yes
MSTRG.18214.3 : Arhgap45 : D4AAI2	5.87	yes	up	2.5E-04	yes	chr7	exon	internal exon	NA	1.53	no	up	0.58	no	1.20	no	up	4.5E-05	yes
ENSRNOT00000077681 : Gpx4 : A0A0G2K398	0.42	yes	down	9.7E-05	yes	chr7	intron	internal intron	NA	0.92	no	down	0.49	no	0.91	no	down	1.6E-05	yes
MSTRG.19538.11 : Nfrkb : D4A421	0.58	yes	down	1.3E-05	yes	chr8	intron	internal intron	NA	1.01	no	up	0.98	no	0.89	no	down	2.5E-03	yes
ENSRNOT00000072611 : Rpl30l1 : M0RD99	4.10	yes	up	1.5E-08	yes	chr9	intron	last intron	NA	0.94	no	down	0.81	no	0.97	no	down	6.7E-01	no
ENSRNOT00000060648 : Arhgap45 : D4AAI2	5.87	yes	up	2.5E-04	yes	chr7	exon	internal exon	NA	2.14	yes	up	0.06	no	1.20	no	up	4.5E-05	yes
ENSRNOT00000079727 : Slc12a6 : A0A0G2K6T0	0.22	yes	down	4.0E-04	yes	chr3	intron	internal intron	NA	0.94	no	down	0.84	no	1.10	no	up	9.9E-02	no
ENSRNOT00000009417 : Cdk14 : D3ZSZ0	0.30	yes	down	1.6E-17	yes	chr4	intron	internal intron	NA	0.40	yes	down	0.11	no	1.02	no	up	4.9E-01	no
MSTRG.2353.1 : Ptar1 : D3ZWG9	2.31	yes	up	2.3E-04	yes	chr1	exon	internal exon	NA	1.11	no	up	0.50	no	0.95	no	down	1.2E-02	yes
MSTRG.13469.2 : Fbn1 : G3V9M6	0.29	yes	down	5.0E-07	yes	chr3	intron	internal intron	NA	3.86	yes	up	0.13	no	0.99	no	down	4.3E-01	no
ENSRNOT00000082271 : Plec : F7F9U6	0.33	yes	down	4.2E-06	yes	chr7	exon	last exon	NA	1.04	no	up	0.53	no	0.90	no	down	2.5E-03	yes
ENSRNOT00000089346 : Rbfox3 : A0A0G2K2J4	0.25	yes	down	5.8E-04	yes	chr10	exon	internal exon	NA	0.96	no	down	0.41	no	0.94	no	down	8.2E-05	yes
MSTRG.15392.1 : Itpr2 : Q6W3E4	2.12	yes	up	2.5E-04	yes	chr4	intron	internal intron	NA	0.98	no	down	0.87	no	0.97	no	down	7.9E-01	no
MSTRG.18072.9 : Dgka : P51556	0.72	yes	down	1.5E-04	yes	chr7	exon	internal exon	NA	0.94	no	down	0.84	no	0.95	no	down	8.8E-04	yes
ENSRNOT00000079118 : Arhgap1 : D4A6C5	3.73	yes	up	1.8E-11	yes	chr3	intron	internal intron	NA	1.10	no	up	0.87	no	1.04	no	up	1.7E-04	yes
ENSRNOT00000088473 : Aldoa : P05065	0.17	yes	down	1.4E-04	yes	chr1	exon	last exon	NA	0.79	no	down	0.81	no	0.98	no	down	4.2E-08	yes
MSTRG.11985.1 : Vars : Q04462	4.99	yes	up	5.5E-05	yes	chr20	intron	internal intron	NA	1.44	no	up	0.62	no	1.01	no	up	2.1E-04	yes
ENSRNOT00000017322 : Epb41l2 : D3ZM69	0.25	yes	down	1.1E-12	yes	chr1	intron	internal intron	NA	0.98	no	down	0.19	no	0.91	no	down	8.9E-11	yes

MSTRG.5399.2 : Eln : A0A0G2JST5	3.55	yes	up	3.0E-04	yes	chr12	intron	internal intron	NA	0.75	no	down	0.06	no	0.91	no	down	1.2E-01	no
ENSRNOT00000085410 : Arhgap4 : A0A0G2JVF0	0.34	yes	down	1.4E-05	yes	chrX	intron	internal intron	NA	1.62	no	up	0.21	no	1.07	no	up	7.1E-04	yes
MSTRG.9752.1 : Coq9 : Q68FT1	0.32	yes	down	5.1E-11	yes	chr19	promoter	Distal	LCP	0.92	no	down	0.67	no	0.93	no	down	1.8E-05	yes
MSTRG.9721.4 : Cdh11 : F1MAH6	1.94	yes	up	2.2E-05	yes	chr19	exon	last exon	NA	1.15	no	up	0.92	no	0.91	no	down	6.5E-11	yes
MSTRG.7186.4 : Sptbn1 : A0A0G2K8W9	5.72	yes	up	4.0E-04	yes	chr14	intron	internal intron	NA	0.33	yes	down	0.45	no	0.87	no	down	1.1E-18	yes
MSTRG.19088.1 : Scube1 : F1M987	2.13	yes	up	1.3E-04	yes	chr7	intron	internal intron	NA	0.37	yes	down	0.20	no	0.80	yes	down	1.2E-14	yes
ENSRNOT00000035916 : Gnas : P63095	0.77	yes	down	3.7E-04	yes	chr3	promoter	Distal	HCP	0.80	no	down	0.43	no	0.90	no	down	5.6E-15	yes
MSTRG.21023.10 : Fn1 : F1LST1	0.11	yes	down	7.7E-11	yes	chr9	intron	internal intron	NA	9.31	yes	up	0.44	no	1.27	yes	up	4.1E-20	yes
MSTRG.9721.7 : Cdh11 : F1MAH6	1.94	yes	up	2.2E-05	yes	chr19	exon	last exon	NA	1.15	no	up	0.21	no	0.91	no	down	6.5E-11	yes
ENSRNOT00000076030 : Parp4 : A0A096MJR6	1.23	yes	up	1.1E-06	yes	chr15	intron	last intron	NA	0.62	no	down	0.14	no	0.95	no	down	6.6E-05	yes
ENSRNOT00000087893 : Fmr1 : Q80WE1	inf	yes	up	3.0E-04	yes	chrX	intron	internal intron	NA	1.02	no	up	0.94	no	1.09	no	up	7.3E-06	yes
ENSRNOT00000016608 : Skap2 : Q920G0	0.26	yes	down	1.3E-08	yes	chr4	intron	internal intron	NA	1.28	no	up	0.14	no	1.23	yes	up	9.4E-12	yes
MSTRG.19692.2 : Zw10 : Q4V8C2	7.96	yes	up	7.6E-05	yes	chr8	intron	last intron	NA	1.20	no	up	0.43	no	1.01	no	up	2.9E-01	no
ENSRNOT00000077784 : Kif1b : A0A0G2KA12	3.65	yes	up	7.4E-05	yes	chr5	intron	internal intron	NA	0.70	no	down	0.13	no	1.05	no	up	6.5E-02	no
ENSRNOT00000015179 : Vcl : P85972	0.35	yes	down	3.5E-07	yes	chr15	intron	internal intron	NA	0.80	no	down	0.16	no	0.88	no	down	2.8E-16	yes
MSTRG.11582.1 : Palmd : Q4KM62	0.16	yes	down	7.2E-05	yes	chr2	promoter	Distal	LCP	0.73	no	down	0.03	yes	0.77	yes	down	4.3E-06	yes
ENSRNOT00000084018 : Tbx3 : A0A0G2K8D7	2.83	yes	up	2.0E-10	yes	chr12	intron	internal intron	NA	0.62	no	down	0.45	no	0.99	no	down	7.3E-01	no
MSTRG.16324.4 : Pabpc4 : G3V9N0	15.58	yes	up	1.8E-05	yes	chr5	intron	first intron	NA	1.06	no	up	0.84	no	1.10	no	up	6.8E-12	yes
MSTRG.4716.7 : Adcy5 : G3V9G1	0.16	yes	down	2.3E-10	yes	chr11	intron	internal intron	NA	1.36	no	up	0.21	no	1.00	no	down	7.6E-01	no
MSTRG.10104.2 : Sf3b3 : E9PT66	0.34	yes	down	3.6E-04	yes	chr19	exon	last exon	NA	0.94	no	down	0.84	no	0.97	no	down	3.1E-07	yes
ENSRNOT00000076193 : Ophn1 : P0CAX5	0.26	yes	down	5.2E-06	yes	chrX	exon	last exon	NA	0.97	no	down	0.72	no	0.94	no	down	1.5E-02	yes
MSTRG.4443.3 : Ltn1 : F1M9Q3	0.38	yes	down	4.2E-04	yes	chr11	intron	internal intron	NA	0.56	no	down	0.46	no	1.08	no	up	1.4E-11	yes
MSTRG.16324.3 : Pabpc4 : G3V9N0	15.58	yes	up	1.8E-05	yes	chr5	intron	first intron	NA	0.98	no	down	0.93	no	1.10	no	up	6.8E-12	yes
MSTRG.5280.6 : Taf6 : Q498R0	0.74	yes	down	2.2E-06	yes	chr12	intron	internal intron	NA	1.14	no	up	0.66	no	0.91	no	down	1.8E-03	yes
ENSRNOT00000018691 : Gpx4 : A0A0G2K398	0.42	yes	down	9.7E-05	yes	chr7	intron	internal intron	NA	0.71	no	down	0.26	no	0.91	no	down	1.6E-05	yes
ENSRNOT00000074070 : Ces1f : M0R7R1	0.26	yes	down	2.8E-08	yes	chr19	intron	internal intron	NA	0.79	no	down	0.42	no	0.86	no	down	3.2E-11	yes
MSTRG.11265.23 : Ubap2l : E9PTR4	6.62	yes	up	4.0E-04	yes	chr2	intron	internal intron	NA	0.50	yes	down	0.21	no	1.13	no	up	3.8E-12	yes
ENSRNOT00000079919 : Kif1b : A0A0G2KA12	3.65	yes	up	7.4E-05	yes	chr5	intron	internal intron	NA	1.68	no	up	0.34	no	1.05	no	up	6.5E-02	no
MSTRG.19173.9 : Ano6 : A0A0G2K1M7	3.87	yes	up	9.8E-09	yes	chr7	intron	internal intron	NA	0.92	no	down	0.45	no	0.91	no	down	2.1E-09	yes
MSTRG.15855.4 : RGD1306148 : F1M446	0.22	yes	down	4.0E-05	yes	chr5	intron	internal intron	NA	1.31	no	up	0.48	no	1.01	no	up	7.4E-02	no
MSTRG.6691.2 : Pdgfra : G3V6A0	0.22	yes	down	1.6E-04	yes	chr14	intron	last intron	NA	1.12	no	up	0.62	no	1.02	no	up	4.1E-02	yes
ENSRNOT00000056022 : Fbn1 : G3V9M6	0.29	yes	down	5.0E-07	yes	chr3	intron	internal intron	NA	0.89	no	down	0.24	no	0.99	no	down	4.3E-01	no
ENSRNOT00000076197 : Smndc1 : Q4QQU6	inf	yes	up	1.3E-04	yes	chr1	promoter	Distal	LCP	0.94	no	down	0.55	no	1.06	no	up	2.2E-04	yes
MSTRG.12655.4 : Gle1 : Q4KLN4	0.16	yes	down	1.6E-04	yes	chr3	promoter	Proximal	LCP	0.76	no	down	0.26	no	0.95	no	down	1.3E-02	yes
ENSRNOT00000064660 : Tor1a : Q68G38	3.04	yes	up	5.0E-05	yes	chr3	exon	last exon	NA	1.14	no	up	0.16	no	1.02	no	up	1.6E-01	no
ENSRNOT00000008200 : Lrpprc : F1LM33	0.61	yes	down	7.5E-05	yes	chr6	intron	internal intron	NA	1.04	no	up	0.59	no	0.97	no	down	4.6E-07	yes
MSTRG.3296.4 : Gas7 : M0R4R4	4.60	yes	up	4.0E-11	yes	chr10	intron	first intron	NA	0.97	no	down	0.88	no	1.22	yes	up	2.5E-15	yes
ENSRNOT00000021286 : Epn1 : O88339	2.09	yes	up	1.9E-04	yes	chr1	intron	internal intron	NA	1.17	no	up	0.49	no	0.99	no	down	3.7E-01	no
MSTRG.7171.3 : Efemp1 : Q6AXN2	0.06	yes	down	2.6E-05	yes	chr14	exon	last exon	NA	0.36	yes	down	0.02	yes	0.94	no	down	1.1E-08	yes
ENSRNOT00000020763 : Pgm5 : D3ZVR9	2.44	yes	up	4.5E-04	yes	chr1	intron	last intron	NA	0.56	no	down	0.59	no	1.01	no	up	3.2E-01	no
MSTRG.7780.5 : Lmo7 : A0A0G2K174	3.35	yes	up	1.2E-04	yes	chr15	intron	internal intron	NA	0.87	no	down	0.78	no	0.96	no	down	4.2E-06	yes
MSTRG.16606.2 : Alpl : P08289	0.52	yes	down	2.4E-04	yes	chr5	promoter	Intermediate	LCP	2.61	yes	up	0.20	no	1.00	no	down	6.2E-01	no
MSTRG.6164.1 : Mpc2 : P38718	0.25	yes	down	5.7E-05	yes	chr13	promoter	Intermediate	ICP	1.20	no	up	0.35	no	0.90	no	down	1.6E-06	yes
ENSRNOT00000016442 : Prkcb : A0A0G2K5Q0	3.82	yes	up	4.6E-04	yes	chr1	intron	internal intron	NA	1.53	no	up	0.19	no	1.10	no	up	1.7E-07	yes
ENSRNOT00000009737 : Atp6v1f : P50408	3.83	yes	up	8.5E-07	yes	chr4	exon	last exon	NA	0.22	yes	down	0.24	no	1.10	no	up	1.4E-09	yes
MSTRG.21909.3 : Acsl4 : O35547	0.19	yes	down	1.2E-04	yes	chrX	intron	internal intron	NA	0.89	no	down	0.39	no	1.22	yes	up	2.0E-10	yes
ENSRNOT00000093052 : Picalm : Q66SY1	8.74	yes	up	2.1E-13	yes	chr1	intron	internal intron	NA	1.05	no	up	0.84	no	1.13	no	up	1.1E-06	yes
MSTRG.1907.1 : Ptdss2 : B2GV22	0.17	yes	down	6.9E-07	yes	chr1	intron	internal intron	NA	1.01	no	up	0.97	no	0.94	no	down	7.1E-04	yes
MSTRG.15948.2 : Cdk5rap2 : F1M4B7	0	yes	down	1.4E-04	yes	chr5	intron	internal intron	NA	1.01	no	up	0.98	no	0.98	no	down	2.6E-01	no
ENSRNOT00000081534 : Ncstn : Q8CGU6	0.30	yes	down	6.8E-05	yes	chr13	intron	first intron	NA	0.95	no	down	0.90	no	1.02	no	up	4.0E-02	yes
ENSRNOT00000024732 : Chd5 : D3ZR50	0.12	yes	down	1.1E-04	yes	chr5	intron	internal intron	NA	0.93	no	down	0.31	no	0.87	no	down	3.8E-03	yes
MSTRG.20352.16 : Ubp1 : D4A030	0.44	yes	down	2.3E-04	yes	chr8	intron	internal intron	NA	0.94	no	down	0.22	no	0.98	no	down	1.9E-01	no
ENSRNOT00000086352 : Col5a1 : G3V763	0.33	yes	down	4.5E-04	yes	chr3	intron	internal intron	NA	0.97	no	down	0.76	no	1.08	no	up	2.2E-06	yes
MSTRG.3645.5 : Slfn13 : A0A096MKD0	2.27	yes	up	1.5E-04	yes	chr10	promoter	Intermediate	LCP	0.89	no	down	0.50	no	0.97	no	down	2.7E-02	yes
MSTRG.8727.2 : Dsp : F1LMV6	inf	yes	up	5.3E-04	yes	chr17	intron	internal intron	NA	1.03	no	up	0.93	no	1.01	no	up	5.1E-01	no
ENSRNOT00000003759 : Selp : A0A096MK10	inf	yes	up	7.4E-05	yes	chr13	promoter	Distal	LCP	4.13	yes	up	0.23	no	0.93	no	down	6.5E-03	yes
ENSRNOT00000066281 : Arhgef18 : F1LT94	0.62	yes	down	4.6E-05	yes	chr12	exon	internal exon	NA	1.01	no	up	0.09	no	0.93	no	down	5.9E-05	yes
MSTRG.110.5 : Lama2 : F1M614	0.44	yes	down	4.4E-04	yes	chr1	intron	internal intron	NA	1.02	no	up	0.49	no	1.06	no	up	1.3E-07	yes
MSTRG.11139.4 : Lrba : A0A0G2JYI0	10.95	yes	up	4.8E-12	yes	chr2	intron	internal intron	NA	0.91	no	down	0.74	no	0.94	no	down	4.5E-07	yes
ENSRNOT00000079695 : Ipo8 : A0A0G2K6J6	3.66	yes	up	4.4E-04	yes	chr4	intron	internal intron	NA	1.07	no	up	0.78	no	1.07	no	up	4.0E-07	yes
ENSRNOT00000032152 : Dlat : P08461	4.32	yes	up	2.7E-04	yes	chr8	intron	internal intron	NA	1.11	no	up	0.39	no	0.96	no	down	3.7E-05	yes
MSTRG.12277.3 : Ctnna3 : B2RYN9	0	yes	down	1.3E-06	yes	chr20	promoter	Distal	LCP	0.95	no	down	0.84	no	1.03	no	up	1.7E-01	no
MSTRG.20350.2 : Clasp2 : A0A0G2JZM8	0.25	yes	down	1.3E-05	yes	chr8	intron	internal intron	NA	0.77	no	down	0.40	no	0.98	no	down	1.8E-01	no
ENSRNOT00000056145 : Ptpn23 : F1M951	1.59	yes	up	2.2E-04	yes	chr8	exon	internal exon	NA	0.91	no	down	0.51	no	0.98	no	down	1.3E-03	yes
ENSRNOT00000003713 : Npl : Q66H59	2.35	yes	up	1.9E-05	yes	chr13	intron	internal intron	NA	0.80	no	down	0.55	no	1.08	no	up	5.3E-06	yes
MSTRG.18557.4 : Mon2 : D3ZCG3	0.07	yes	down	1.5E-08	yes	chr7	intron	internal intron	NA	0.96	no	down	0.83	no	1.05	no	up	7.1E-07	yes
MSTRG.10603.3 : Nnt : Q5BJZ3	3.00	yes	up	1.8E-04	yes	chr2	intron	internal intron	NA	0.57	no	down	0.19	no	0.96	no	down	1.2E-07	yes
MSTRG.10154.1 : Cmip : A0A0G2JYB9	0.58	yes	down	9.9E-05	yes	chr19	intron	internal intron	NA	0.91	no	down	0.71	no	0.89	no	down	2.1E-04	yes
ENSRNOT00000079432 : Asph : A0A096MKE0	2.56	yes	up	9.8E-06	yes	chr5	intron	internal intron	NA	0.86	no	down	0.50	no	0.99	no	down	1.3E-01	no
ENSRNOT00000019453 : RGD1563354 : F1M4P5	0.18	yes	down	5.6E-06	yes	chr3	promoter	Distal	LCP	1.69	no	up	0.06	no	0.66	yes	down	1.6E-11	yes
MSTRG.19538.10 : Nfrkb : D4A421	0.58	yes	down	1.3E-05	yes	chr8	intron	internal intron	NA	0.96	no	down	0.83	no	0.89	no	down	2.5E-03	yes
MSTRG.19538.2 : Nfrkb : D4A421	0.58	yes	down	1.3E-05	yes	chr8	intron	internal intron	NA	1.05	no	up	0.47	no	0.89	no	down	2.5E-03	yes
MSTRG.21144.5 : Cab39 : A0A0G2JZH0	39.86	yes	up	1.2E-27	yes	chr9	promoter	Intermediate	LCP	1.38	no	up	0.47	no	0.99	no	down	5.6E-01	no
ENSRNOT00000027773 : Actn4 : Q9QXQ0	0.43	yes	down	2.1E-04	yes	chr1	intron	internal intron	NA	0.90	no	down	0.63	no	1.01	no	up	3.9E-03	yes
MSTRG.11953.32 : RT1-CE7 : D3ZLE6	inf	yes	up	7.3E-07	yes	chr20	exon	internal exon	NA	1.16	no	up	0.39	no	1.22	yes	up	7.3E-16	yes
MSTRG.2881.10 : Pkd1 : Q9ERV0	inf	yes	up	6.6E-05	yes	chr10	intron	internal intron	NA	0.85	no	down	0.53	no	1.19	no	up	9.8E-07	yes
MSTRG.5510.1 : Pitpnm2 : A0A0G2JW50	2.70	yes	up	3.9E-04	yes	chr12	promoter	Distal	LCP	0.96	no	down	0.88	no	0.89	no	down	1.9E-08	yes
ENSRNOT00000040069 : Eln : Q99372	3.55	yes	up	3.0E-04	yes	chr12	intron	internal intron	NA	0.67	no	down	0.24	no	0.82	yes	down	3.9E-08	yes
ENSRNOT00000084946 : Mef2a : A0A0G2JSZ4	0.04	yes	down	2.4E-04	yes	chr1	exon	last exon	NA	0.99	no	down	0.51	no	0.78	yes	down	4.1E-07	yes
MSTRG.8351.9 : Adam9 : E9PTA4	0.15	yes	down	9.7E-05	yes	chr16	intron	internal intron	NA	1.23	no	up	0.30	no	1.06	no	up	3.4E-02	yes
MSTRG.10153.5 : Cmip : A0A0G2JYB9	0.58	yes	down	9.9E-05	yes	chr19	intron	internal intron	NA	0.67	no	down	0.69	no	0.89	no	down	2.1E-04	yes
MSTRG.19672.1 : Rnf214 : D4A3V4	0.59	yes	down	3.7E-05	yes	chr8	intron	internal intron	NA	0.79	no	down	0.39	no	1.00	no	down	9.9E-01	no
ENSRNOT00000039059 : Cdc42bpb : A0A0G2KB58	0.39	yes	down	3.8E-04	yes	chr6	intron	internal intron	NA	0.86	no	down	0.29	no	0.97	no	down	3.6E-07	yes
MSTRG.3351.1 : Polr2a : D4A5A6	2.68	yes	up	1.4E-05	yes	chr10	exon	internal exon	NA	1.09	no	up	0.86	no	1.00	no	down	6.0E-01	no
ENSRNOT00000019687 : Zbtb4 : D4A8X0	inf	yes	up	2.4E-05	yes	chr10	intron	last intron	NA	0.55	no	down	0.25	no	0.97	no	down	6.9E-02	no
MSTRG.11427.1 : Man1a2 : D3ZR49	1.09	no	up	3.3E-05	yes	chr2	exon	last exon	NA	0.62	no	down	0.15	no	1.05	no	up	1.6E-05	yes
MSTRG.7561.6 : Phf11b : M0RB46	3.03	yes	up	2.9E-04	yes	chr15	promoter	Intermediate	LCP	1.11	no	up	0.81	no	1.15	no	up	2.3E-06	yes
MSTRG.4263.3 : Tnrc6c : D3ZRA6	0.39	yes	down	2.3E-06	yes	chr10	intron	internal intron	NA	0.87	no	down	0.26	no	1.07	no	up	1.9E-01	no
ENSRNOT00000063787 : Rpl14 : Q63507	6.79	yes	up	7.4E-07	yes	chr8	intron	internal intron	NA	1.09	no	up	0.79	no	1.09	no	up	1.1E-04	yes
MSTRG.12646.5 : Rapgef1 : F1M8L9	inf	yes	up	4.7E-05	yes	chr3	intron	internal intron	NA	2.08	yes	up	0.02	yes	0.88	no	down	4.1E-04	yes
MSTRG.11265.10 : Ubap2l : E9PTR4	6.62	yes	up	4.0E-04	yes	chr2	intron	internal intron	NA	0.91	no	down	0.65	no	1.13	no	up	3.8E-12	yes
MSTRG.18268.4 : Akap8 : Q63014	0.08	yes	down	2.7E-04	yes	chr7	intron	internal intron	NA	1.13	no	up	0.53	no	0.99	no	down	2.8E-01	no
ENSRNOT00000081881 : Scrn3 : A0A0G2K189	9.14	yes	up	1.0E-04	yes	chr3	intron	first intron	NA	0.95	no	down	0.84	no	0.92	no	down	8.4E-04	yes
MSTRG.6732.1 : Limch1 : F1M392	1.09	no	up	3.3E-04	yes	chr14	intron	last intron	NA	1.40	no	up	0.63	no	0.85	no	down	1.1E-13	yes
MSTRG.5400.1 : Eln : Q99372	3.55	yes	up	3.0E-04	yes	chr12	intron	internal intron	NA	0.18	yes	down	0.17	no	0.82	yes	down	3.9E-08	yes
MSTRG.19710.1 : Alg9 : D3ZCW5	0.10	yes	down	9.3E-08	yes	chr8	intron	first intron	NA	1.02	no	up	0.82	no	1.11	no	up	6.0E-03	yes
MSTRG.5942.3 : Ppp1r12b : D3ZIC4	2.69	yes	up	3.9E-04	yes	chr13	intron	internal intron	NA	1.10	no	up	0.56	no	0.95	no	down	7.9E-06	yes
MSTRG.21168.2 : Gigyf2 : A0A096MJI4	0.59	yes	down	1.9E-05	yes	chr9	intron	internal intron	NA	0.86	no	down	0.28	no	1.31	yes	up	2.3E-10	yes
ENSRNOT00000057965 : Nova2 : F1M4H5	0.12	yes	down	3.3E-07	yes	chr1	exon	last exon	NA	0.73	no	down	0.05	yes	0.92	no	down	2.8E-08	yes
MSTRG.3051.1 : Gfpt2 : Q4KMC4	0.77	yes	down	3.9E-05	yes	chr10	intron	internal intron	NA	0.96	no	down	0.74	no	1.43	yes	up	1.7E-17	yes

MSTRG.18723.3 : Deptor : F1M8Y4	2.15	yes	up	9.9E-15	yes	chr7	intron	internal intron	NA	0.56	no	down	0.40	no	0.97	no	down	2.0E-01	no
MSTRG.19538.8 : Nfrkb : D4A421	0.58	yes	down	1.3E-05	yes	chr8	intron	internal intron	NA	1.27	no	up	0.39	no	0.89	no	down	2.5E-03	yes
MSTRG.15089.2 : Csgalnact2 : D4A5Z0	2.74	yes	up	6.9E-07	yes	chr4	intron	internal intron	NA	0.58	no	down	0.19	no	0.94	no	down	1.2E-02	yes
MSTRG.19220.1 : Kmt2d : A0A0G2JVD6	6.53	yes	up	2.1E-09	yes	chr7	exon	last exon	NA	0.98	no	down	0.97	no	1.03	no	up	3.5E-02	yes
ENSRNOT00000092086 : Picalm : Q66SY1	8.74	yes	up	2.1E-13	yes	chr1	intron	internal intron	NA	0.60	no	down	0.06	no	1.13	no	up	1.1E-06	yes
MSTRG.16609.1 : Ece1 : Q6IN10	0.58	yes	down	9.3E-05	yes	chr5	intron	internal intron	NA	1.87	no	up	0.74	no	1.14	no	up	8.3E-15	yes
ENSRNOT00000076489 : Becn1 : Q91XJ1	0.15	yes	down	1.2E-04	yes	chr10	exon	first exon	NA	1.88	no	up	0.18	no	0.97	no	down	9.2E-02	no
ENSRNOT00000083611 : Naa10 : D3ZUQ2	0.09	yes	down	2.8E-11	yes	chrX	intron	internal intron	NA	0.33	yes	down	0.11	no	1.04	no	up	2.6E-02	yes
ENSRNOT00000007103 : Myef2 : A0A0G2K402	2.63	yes	up	2.7E-04	yes	chr3	promoter	Intermediate	LCP	0.77	no	down	0.28	no	0.93	no	down	4.5E-09	yes
ENSRNOT00000062055 : Cast : F1LPH1	0.36	yes	down	1.6E-06	yes	chr2	intron	internal intron	NA	1.07	no	up	0.82	no	0.95	no	down	2.4E-10	yes
ENSRNOT00000090288 : Tpm1 : Q923Z2	9.41	yes	up	5.1E-14	yes	chr8	intron	internal intron	NA	0.97	no	down	0.82	no	0.75	yes	down	6.0E-18	yes
MSTRG.4525.1 : Mx1 : Q499S4	3.65	yes	up	1.1E-13	yes	chr11	intron	internal intron	NA	0.58	no	down	0.36	no	1.36	yes	up	1.2E-13	yes
MSTRG.9435.3 : Tcerg1 : B5DEZ4	0.26	yes	down	2.8E-04	yes	chr18	promoter	Intermediate	ICP	0.92	no	down	0.71	no	1.02	no	up	4.4E-02	yes
MSTRG.1783.1 : Tgfb1i1 : Q99PD6	3.08	yes	up	7.2E-05	yes	chr1	intron	internal intron	NA	0.71	no	down	0.24	no	1.06	no	up	1.6E-04	yes
MSTRG.13653.2 : Xrn2 : D4A914	0.32	yes	down	3.4E-05	yes	chr3	intron	internal intron	NA	0.75	no	down	0.54	no	1.03	no	up	8.8E-06	yes
ENSRNOT00000026327 : Dag1 : F1M8K0	2.95	yes	up	1.4E-04	yes	chr8	exon	first exon	NA	1.14	no	up	0.22	no	0.91	no	down	7.5E-12	yes
MSTRG.2010.4 : Ppp6r3 : D3ZBT9	3.21	yes	up	8.1E-07	yes	chr1	intron	internal intron	NA	1.12	no	up	0.32	no	0.98	no	down	7.0E-03	yes
ENSRNOT00000083957 : Rab27a : P23640	0.72	yes	down	7.7E-07	yes	chr8	exon	internal exon	NA	0.97	no	down	0.97	no	1.02	no	up	2.0E-01	no
ENSRNOT00000076266 : Parp4 : A0A096MJ98	1.23	yes	up	1.1E-06	yes	chr15	intron	last intron	NA	0.97	no	down	0.67	no	1.00	no	down	9.9E-01	no
MSTRG.4335.2 : Pcyt2 : O88637	4.82	yes	up	3.0E-05	yes	chr10	intron	last intron	NA	1.54	no	up	0.03	yes	0.94	no	down	1.8E-03	yes
MSTRG.19253.2 : Smarcd1 : D3ZBS9	0.24	yes	down	1.4E-04	yes	chr7	intron	internal intron	NA	0.93	no	down	0.71	no	0.95	no	down	2.9E-07	yes
ENSRNOT00000041678 : Tyk2 : D3ZD03	0.17	yes	down	1.5E-06	yes	chr8	intron	internal intron	NA	1.39	no	up	0.21	no	1.01	no	up	4.7E-01	no
ENSRNOT00000086272 : Ndufv3 : G3V644	0	yes	down	1.9E-04	yes	chr20	intron	first intron	NA	0.74	no	down	0.22	no	1.00	no	up	9.0E-01	no
MSTRG.8325.5 : Hook3 : Q7TQ77	0.22	yes	down	2.1E-04	yes	chr16	intron	internal intron	NA	0.25	yes	down	0.21	no	1.01	no	up	5.0E-03	yes
ENSRNOT00000090470 : Exoc1 : A0A0G2K2V5	inf	yes	up	6.1E-05	yes	chr14	intron	first intron	NA	1.00	no	down	0.98	no	0.90	no	down	2.2E-04	yes
MSTRG.10871.9 : Dcun1d1 : D3ZRV0	1.60	yes	up	3.7E-04	yes	chr2	exon	internal exon	NA	1.09	no	up	0.73	no	0.97	no	down	2.6E-02	yes
