# Supplementary material for: Revealing the pathogenic changes of PAH based on multiomics characteristics
Source: J Transl Med. 2019 Jul 22;17:231. doi: 10.1186/s12967-019-1981-5 (PMC6647123; doi:10.1186/s12967-019-1981-5)
Supplement: Supplementary file 3 — Additional file 3: Table S3. GO enrichment analysis ( p-value < 0.05). [file 12967_2019_1981_MOESM3_ESM.docx]

**Table S3.** GO enrichment analysis ( *p*-value < 0.05 ). 90 Go terms are identified using the filter *p* < 0.05. The Gene Ontology are divided into three parts: molecular function, biological process and cellular component. The category, *p*-value and the FDR of Go terms are described in the table.

**Table S3.** **GO enrichment analysis ( *p*-value < 0.05 )**

| **Category** | **Go term** | ***p*-value** | **FDR** |
| --- | --- | --- | --- |
| CELLULAR COMPONENT | GO:0016529~sarcoplasmic reticulum | 3.7E-09 | 5.0E-06 |
| CELLULAR COMPONENT | GO:0005604~basement membrane | 3.1E-08 | 4.2E-05 |
| BIOLOGICAL PROCESS | GO:0010951~negative regulation of endopeptidase activity | 8.8E-08 | 1.5E-04 |
| BIOLOGICAL PROCESS | GO:0006958~complement activation, classical pathway | 1.5E-07 | 2.6E-04 |
| BIOLOGICAL PROCESS | GO:0045087~innate immune response | 1.2E-06 | 2.1E-03 |
| MOLECULAR FUNCTION | GO:0005198~structural molecule activity | 2.5E-06 | 3.6E-03 |
| CELLULAR COMPONENT | GO:0005581~collagen trimer | 9.5E-06 | 1.3E-02 |
| BIOLOGICAL PROCESS | GO:0007596~blood coagulation | 2.5E-05 | 4.3E-02 |
| CELLULAR COMPONENT | GO:0005578~proteinaceous extracellular matrix | 2.8E-05 | 3.9E-02 |
| BIOLOGICAL PROCESS | GO:0006749~glutathione metabolic process | 2.9E-05 | 4.9E-02 |
| BIOLOGICAL PROCESS | GO:0098869~cellular oxidant detoxification | 3.6E-05 | 6.1E-02 |
| BIOLOGICAL PROCESS | GO:0051258~protein polymerization | 9.1E-05 | 1.6E-01 |
| BIOLOGICAL PROCESS | GO:0042554~superoxide anion generation | 9.1E-05 | 1.6E-01 |
| BIOLOGICAL PROCESS | GO:0042554~superoxide anion generation | 9.1E-05 | 1.6E-01 |
| BIOLOGICAL PROCESS | GO:0072378~blood coagulation, fibrin clot formation | 1.3E-04 | 2.2E-01 |
| BIOLOGICAL PROCESS | GO:0006911~phagocytosis, engulfment | 1.6E-04 | 2.7E-01 |
| MOLECULAR FUNCTION | GO:0004867~serine-type endopeptidase inhibitor activity | 1.8E-04 | 2.7E-01 |
| CELLULAR COMPONENT | GO:0000786~nucleosome | 2.1E-04 | 2.8E-01 |
| CELLULAR COMPONENT | GO:0000786~nucleosome | 2.1E-04 | 2.8E-01 |
| CELLULAR COMPONENT | GO:0005577~fibrinogen complex | 4.1E-04 | 5.6E-01 |
| MOLECULAR FUNCTION | GO:0004364~glutathione transferase activity | 4.8E-04 | 7.0E-01 |
| MOLECULAR FUNCTION | GO:0003823~antigen binding | 5.4E-04 | 8.0E-01 |
| MOLECULAR FUNCTION | GO:0016209~antioxidant activity | 5.6E-04 | 8.3E-01 |
| MOLECULAR FUNCTION | GO:0043295~glutathione binding | 5.6E-04 | 8.3E-01 |
| MOLECULAR FUNCTION | GO:0005201~extracellular matrix structural constituent | 6.9E-04 | 1.0E+00 |
| MOLECULAR FUNCTION | GO:0005201~extracellular matrix structural constituent | 6.9E-04 | 1.0E+00 |
| MOLECULAR FUNCTION | GO:0005201~extracellular matrix structural constituent | 6.9E-04 | 1.0E+00 |
| MOLECULAR FUNCTION | GO:0030674~protein binding, bridging | 7.4E-04 | 1.1E+00 |
| BIOLOGICAL PROCESS | GO:0050665~hydrogen peroxide biosynthetic process | 1.0E-03 | 1.7E+00 |
| BIOLOGICAL PROCESS | GO:0010880~regulation of release of sequestered calcium ion into cytosol by sarcoplasmic reticulum | 1.1E-03 | 1.9E+00 |
| BIOLOGICAL PROCESS | GO:0010880~regulation of release of sequestered calcium ion into cytosol by sarcoplasmic reticulum | 1.1E-03 | 1.9E+00 |
| CELLULAR COMPONENT | GO:0016459~myosin complex | 1.1E-03 | 1.6E+00 |
| MOLECULAR FUNCTION | GO:0050786~RAGE receptor binding | 1.4E-03 | 2.0E+00 |
| BIOLOGICAL PROCESS | GO:0034116~positive regulation of heterotypic cell-cell adhesion | 1.4E-03 | 2.4E+00 |
| BIOLOGICAL PROCESS | GO:0046314~phosphocreatine biosynthetic process | 1.7E-03 | 2.8E+00 |
| BIOLOGICAL PROCESS | GO:0007160~cell-matrix adhesion | 2.2E-03 | 3.7E+00 |
| BIOLOGICAL PROCESS | GO:0042178~xenobiotic catabolic process | 2.5E-03 | 4.2E+00 |
| BIOLOGICAL PROCESS | GO:0006910~phagocytosis, recognition | 3.1E-03 | 5.2E+00 |
| BIOLOGICAL PROCESS | GO:0070458~cellular detoxification of nitrogen compound | 3.3E-03 | 5.5E+00 |
| CELLULAR COMPONENT | GO:0031091~platelet alpha granule | 4.6E-03 | 6.2E+00 |
| MOLECULAR FUNCTION | GO:0004866~endopeptidase inhibitor activity | 5.1E-03 | 7.3E+00 |
| MOLECULAR FUNCTION | GO:0019825~oxygen binding | 5.1E-03 | 7.3E+00 |
| MOLECULAR FUNCTION | GO:0004111~creatine kinase activity | 5.3E-03 | 7.5E+00 |
| MOLECULAR FUNCTION | GO:0004854~xanthine dehydrogenase activity | 5.3E-03 | 7.5E+00 |
| MOLECULAR FUNCTION | GO:0055056~D-glucose transmembrane transporter activity | 5.3E-03 | 7.5E+00 |
| CELLULAR COMPONENT | GO:0001725~stress fiber | 5.3E-03 | 7.1E+00 |
| BIOLOGICAL PROCESS | GO:0018916~nitrobenzene metabolic process | 5.4E-03 | 8.8E+00 |
| BIOLOGICAL PROCESS | GO:0009115~xanthine catabolic process | 5.4E-03 | 8.8E+00 |
| MOLECULAR FUNCTION | GO:0030247~polysacellular componentharide binding | 5.8E-03 | 8.2E+00 |
| BIOLOGICAL PROCESS | GO:0006936~muscle contraction | 6.5E-03 | 1.1E+01 |
| BIOLOGICAL PROCESS | GO:0006801~superoxide metabolic process | 7.1E-03 | 1.1E+01 |
| CELLULAR COMPONENT | GO:0005587~collagen type IV trimer | 7.6E-03 | 1.0E+01 |
| CELLULAR COMPONENT | GO:0005887~integral component of plasma membrane | 7.7E-03 | 1.0E+01 |
| BIOLOGICAL PROCESS | GO:0006957~complement activation, alternative pathway | 7.9E-03 | 1.3E+01 |
| BIOLOGICAL PROCESS | GO:0006874~cellular calcium ion homeostasis | 8.4E-03 | 1.3E+01 |
| MOLECULAR FUNCTION | GO:0034987~immunoglobulin receptor binding | 9.5E-03 | 1.3E+01 |
| MOLECULAR FUNCTION | GO:0009055~electron carrier activity | 1.0E-02 | 1.4E+01 |
| MOLECULAR FUNCTION | GO:0004029~aldehyde dehydrogenase (NAD) activity | 1.1E-02 | 1.5E+01 |
| BIOLOGICAL PROCESS | GO:0045109~intermediate filament organization | 1.1E-02 | 1.8E+01 |
| MOLECULAR FUNCTION | GO:0004252~serine-type endopeptidase activity | 1.2E-02 | 1.6E+01 |
| MOLECULAR FUNCTION | GO:0016175~superoxide-generating NADPH oxidase activity | 1.4E-02 | 1.9E+01 |
| BIOLOGICAL PROCESS | GO:0006068~ethanol catabolic process | 1.4E-02 | 2.2E+01 |
| BIOLOGICAL PROCESS | GO:1902042~negative regulation of extrinsic apoptotic signaling pathway via death domain receptors | 1.5E-02 | 2.2E+01 |
| MOLECULAR FUNCTION | GO:0051537~2 iron, 2 sulfur cluster binding | 1.6E-02 | 2.1E+01 |
| CELLULAR COMPONENT | GO:0043020~NADPH oxidase complex | 1.7E-02 | 2.1E+01 |
| MOLECULAR FUNCTION | GO:0004028~3-chloroallyl aldehyde dehydrogenase activity | 1.8E-02 | 2.3E+01 |
| BIOLOGICAL PROCESS | GO:0090277~positive regulation of peptide hormone secretion | 1.8E-02 | 2.7E+01 |
| BIOLOGICAL PROCESS | GO:0060315~negative regulation of ryanodine-sensitive calcium-release channel activity | 1.8E-02 | 2.7E+01 |
| BIOLOGICAL PROCESS | GO:0006342~chromatin silencing | 1.9E-02 | 2.8E+01 |
| BIOLOGICAL PROCESS | GO:0008152~metabolic process | 2.0E-02 | 2.9E+01 |
| BIOLOGICAL PROCESS | GO:0045907~positive regulation of vasoconstriction | 2.2E-02 | 3.2E+01 |
| MOLECULAR FUNCTION | GO:0016614~oxidoreductase activity, acting on CH-OH group of donors | 2.2E-02 | 2.8E+01 |
| MOLECULAR FUNCTION | GO:0005388~calcium-transporting ATPase activity | 2.2E-02 | 2.8E+01 |
| CELLULAR COMPONENT | GO:0005882~intermediate filament | 2.2E-02 | 2.7E+01 |
| BIOLOGICAL PROCESS | GO:0031639~plasminogen activation | 2.2E-02 | 3.2E+01 |
| BIOLOGICAL PROCESS | GO:0071313~cellular response to caffeine | 2.2E-02 | 3.2E+01 |
| BIOLOGICAL PROCESS | GO:0006816~calcium ion transport | 2.2E-02 | 3.2E+01 |
| BIOLOGICAL PROCESS | GO:0015758~glucose transport | 2.3E-02 | 3.3E+01 |
| BIOLOGICAL PROCESS | GO:0050853~B cell receptor signaling pathway | 2.3E-02 | 3.3E+01 |
| CELLULAR COMPONENT | GO:0033017~sarcoplasmic reticulum membrane | 2.4E-02 | 2.9E+01 |
| MOLECULAR FUNCTION | GO:0005044~scavenger receptor activity | 2.6E-02 | 3.2E+01 |
| BIOLOGICAL PROCESS | GO:0006898~receptor-mediated endocytosis | 2.9E-02 | 4.0E+01 |
| BIOLOGICAL PROCESS | GO:2000352~negative regulation of endothelial cell apoptotic process | 3.4E-02 | 4.5E+01 |
| MOLECULAR FUNCTION | GO:0003779~actin binding | 3.4E-02 | 4.0E+01 |
| CELLULAR COMPONENT | GO:0045095~keratin filament | 3.7E-02 | 4.0E+01 |
| CELLULAR COMPONENT | GO:0070469~respiratory chain | 4.1E-02 | 4.4E+01 |
| MOLECULAR FUNCTION | GO:0020037~heme binding | 4.3E-02 | 4.8E+01 |
| BIOLOGICAL PROCESS | GO:0042572~retinol metabolic process | 4.3E-02 | 5.3E+01 |
| CELLULAR COMPONENT | GO:0042571~immunoglobulin complex, circulating | 4.6E-02 | 4.8E+01 |
| BIOLOGICAL PROCESS | GO:0042730~fibrinolysis | 4.8E-02 | 5.7E+01 |
