# Supplementary material for: Revealing the pathogenic changes of PAH based on multiomics characteristics
Source: J Transl Med. 2019 Jul 22;17:231. doi: 10.1186/s12967-019-1981-5 (PMC6647123; doi:10.1186/s12967-019-1981-5)
Supplement: Supplementary file 4 — Additional file 4: Table S4. The detailed information of the significant KEGG pathways. [file 12967_2019_1981_MOESM4_ESM.pdf]

Table S4. The detailed information of the significant KEGG pathways. Three omics correlated information of genes in the selected key KEGG pathways. The fold change (FC), the direction (dir) and p value (*p*) of three omics information are shown. Meanwhile, the chromosomal localization (Chr), the strand of methylation (strand) and methylated positions (Gene_Info) are also shown in methylation information.

Table S4. The detailed information of the significant KEGG pathways.

KEGG pathway	Gene	Gene name	mRNA information				protein information				Methylation information					
			mRNA_name	FC	dir	*p* value	protein	FC	dir	*p* value	FC	dir	chr	strand	Gene_Info	*p* value
Complement and coagulation cascades	C5	complement C5	ENSRNOT00000092736	0.92	down	0.44	A0A096P6L9	1.29	up	6.09E-17						
	C5	complement C5	ENSRNOT00000025534	1.01	up	0.34	A0A096P6L9	1.29	up	6.09E-17						
	Kng1	kininogen 1	ENSRNOT00000080562	1.02	up	0.47	A0A0G2KAY3	3.00	up	1.12E-18						
	Kng1	kininogen 1	ENSRNOT00000080949	0.9	down	0.34	A0A0G2KAY3	3.00	up	1.12E-18						
	Kng1	kininogen 1	ENSRNOT00000084867	1.52	up	0.11	A0A0G2KAY3	3.00	up	1.12E-18						
	C4bpb	complement component 4 binding protein, beta	ENSRNOT00000005505	0.97	down	0.86	A0A5C5	1.90	up	6.50E-16						
	C6	complement component 6	MSTRG.10628.1	2.8	up	0	F1M7F7	1.31	up	3.07E-17						
	C6	complement component 6	MSTRG.10628.4	2.28	up	0.06	F1M7F7	1.31	up	3.07E-17						
	C6	complement component 6	MSTRG.10628.2	1.04	up	0.04	F1M7F7	1.31	up	3.07E-17						
	C6	complement component 6	MSTRG.10628.3	1.33	up	0.08	F1M7F7	1.31	up	3.07E-17						
	C6	complement component 6	MSTRG.10628.6	2.15	up	0	F1M7F7	1.31	up	3.07E-17						
	C1qb	complement component 1, q subcomponent, B chain	ENSRNOT00000017060	1.2	up	0.7	G3V7N9	1.25	up	9.74E-13						
	C1qb	complement component 1, q subcomponent, B chain	MSTRG.16594.1	1.06	up	0.85	G3V7N9	1.25	up	9.74E-13						
	F13a1	coagulation factor XIII, A1 polypeptide	ENSRNOT00000021568	1.91	up	0.06	G3V811	1.27	up	1.99E-17						
	C3	complement component 3	ENSRNOT00000075494	1.68	up	0.25	M0RBJ7	1.26	up	7.42E-19						
	C3	complement component 3	MSTRG.20503.3	1.41	up	0.05	M0RBJ7	1.26	up	7.42E-19						
	C3	complement component 3	MSTRG.20503.1	1.22	up	0.46	M0RBJ7	1.26	up	7.42E-19						
	C3	complement component 3	ENSRNOT00000073042	1.69	up	0.24	M0RBJ7	1.26	up	7.42E-19						
	Thbd	thrombomodulin	ENSRNOT00000006200	0.57	down	0.75	O35370	0.60	down	5.77E-06						
	Fgg	fibrinogen gamma chain	ENSRNOT00000034521	1.27	up	0.41	P02680	1.57	up	3.22E-17						
	A2m	alpha-2-macroglobulin	ENSRNOT00000019346	2.78	up	0.26	P06238	1.93	up	1.94E-20						
	Fgb	fibrinogen beta chain	ENSRNOT00000009813	0.96	down	0.4	P14480	1.49	up	2.02E-21						
	Proc	protein C	ENSRNOT00000019596	0.94	down	0.4	P31394	0.79	down	7.53E-11						
	F3	coagulation factor III (thromboplastin, tissue factor)	ENSRNOT00000015836	2.53	up	0.48	P42533	0.71	down	4.47E-19						
	F3	coagulation factor III (thromboplastin, tissue factor)	MSTRG.11600.1	0.23	down	0.45	P42533	0.71	down	4.47E-19						
	F3	coagulation factor III (thromboplastin, tissue factor)	MSTRG.11600.3	0.67	down	0.46	P42533	0.71	down	4.47E-19						
	C5ar1	complement component 5a receptor 1	MSTRG.545.1	1.72	up	0.49	P97520	1.47	up	9.88E-13						
	C5ar1	complement component 5a receptor 1	ENSRNOT00000073034	1.05	up	0.6	P97520	1.47	up	9.88E-13						
	C9	complement component 9	ENSRNOT00000018545	0.97	down	0.59	Q62930	1.44	up	6.93E-18						
	F10	coagulation factor X	ENSRNOT00000026677	1.26	up	0.42	Q63207	1.32	up	1.70E-14						
	C4bpa	complement component 4 binding protein, alpha	ENSRNOT00000005461	1.46	up	0.07	Q63514	1.64	up	6.23E-17						
Drug metabolism - cytochrome P450	Mgst2	microsomal glutathione S-transferase 2	MSTRG.10921.1	0.97	down	0.2	A0A0G2JU12	1.49	up	4.54E-11						
	Mgst2	microsomal glutathione S-transferase 2	MSTRG.10921.2	1.07	up	0.88	A0A0G2JU12	1.49	up	4.54E-11						
	Mgst2	microsomal glutathione S-transferase 2	ENSRNOT00000088846	1.09	up	0.66	A0A0G2JU12	1.49	up	4.54E-11						
	Aox3	aldehyde oxidase 3	ENSRNOT00000081146	1.28	up	0.44	A0A0G2K1Z5	0.73	down	6.67E-16						
	Aox3	aldehyde oxidase 3	ENSRNOT00000093062	1	down	0.47	A0A0G2K1Z5	0.73	down	6.67E-16						
	Aox3	aldehyde oxidase 3	ENSRNOT00000092841	1.07	up	0.17	A0A0G2K1Z5	0.73	down	6.67E-16						
	Aox3	aldehyde oxidase 3	ENSRNOT00000092786	1.11	up	0.7	A0A0G2K1Z5	0.73	down	6.67E-16						
	Aox3	aldehyde oxidase 3	ENSRNOT00000093120	0.8	down	0.48	A0A0G2K1Z5	0.73	down	6.67E-16						
	Aox1	aldehyde oxidase 1	MSTRG.20927.3	0.76	down	0.15	F1LRQ1	0.73	down	3.54E-17	0.36	hypo-methylated	9	"+"	intron	1.54E-07
	Aox1	aldehyde oxidase 1	ENSRNOT00000092805	0.98	down	0.5	F1LRQ1	0.73	down	3.54E-17	0.36	hypo-methylated	9	"+"	intron	1.54E-07
	Aox1	aldehyde oxidase 1	MSTRG.20927.1	0.71	down	0.43	F1LRQ1	0.73	down	3.54E-17	0.36	hypo-methylated	9	"+"	intron	1.54E-07
	Aox1	aldehyde oxidase 1	MSTRG.20927.4	0.9	down	0.81	F1LRQ1	0.73	down	3.54E-17	0.36	hypo-methylated	9	"+"	intron	1.54E-07
	Aox1	aldehyde oxidase 1	ENSRNOT00000068633	1.13	up	0.8	F1LRQ1	0.73	down	3.54E-17	0.36	hypo-methylated	9	"+"	intron	1.54E-07
	Aox1	aldehyde oxidase 1	MSTRG.20927.2	0.98	down	0.92	F1LRQ1	0.73	down	3.54E-17	0.36	hypo-methylated	9	"+"	intron	1.54E-07
	Aox1	aldehyde oxidase 1	MSTRG.20927.5	0.66	down	0.37	F1LRQ1	0.73	down	3.54E-17	0.36	hypo-methylated	9	"+"	intron	1.54E-07
	Aox1	aldehyde oxidase 1	ENSRNOT00000092872	1	down	0.6	F1LRQ1	0.73	down	3.54E-17	0.36	hypo-methylated	9	"+"	intron	1.54E-07
	Aox1	aldehyde oxidase 1	MSTRG.20927.7	1.37	up	0.03	F1LRQ1	0.73	down	3.54E-17	0.36	hypo-methylated	9	"+"	intron	1.54E-07
	Gsta3	glutathione S-transferase alpha 3	ENSRNOT00000088416	0.76	down	0.31	P04904	0.64	down	2.87E-15	2.74	hyper-methylated	9	"+"	exon	2.28E-04
	Gsta3	glutathione S-transferase alpha 3	ENSRNOT00000077372	1.01	up	0.84	P04904	0.64	down	2.87E-15	2.74	hyper-methylated	9	"+"	exon	2.28E-04
	Adh1	alcohol dehydrogenase 1 (class I)	ENSRNOT00000083682	0.89	down	0.64	P06757	0.78	down	2.84E-16						
	Adh1	alcohol dehydrogenase 1 (class I)	MSTRG.11708.1	1	down	0.97	P06757	0.78	down	2.84E-16						
	Adh1	alcohol dehydrogenase 1 (class I)	ENSRNOT00000085067	0.99	down	0.97	P06757	0.78	down	2.84E-16						
	Gstm3	glutathione S-transferase mu 3	ENSRNOT00000025800	1.01	up	0.91	P08009	0.59	down	2.02E-20						
	Aldh3a1	aldehyde dehydrogenase 3 family, member A1	ENSRNOT00000003182	0.45	down	0.25	P11883	0.72	down	6.70E-23						
	Gsta4	glutathione S-transferase alpha 4	MSTRG.20020.1	0.78	down	0.48	P14942	0.76	down	4.34E-13						
	Gsta4	glutathione S-transferase alpha 4	ENSRNOT00000090146	0.88	down	0.55	P14942	0.76	down	4.34E-13						
	Gsta4	glutathione S-transferase alpha 4	ENSRNOT00000012348	1.17	up	0.76	P14942	0.76	down	4.34E-13						
	Gstt1	glutathione S-transferase theta 1	ENSRNOT00000001669	0.92	down	0.8	Q01579	0.80	down	1.36E-15	0.44	hypo-methylated	20	"+"	exon	5.43E-04
	Gstt1	glutathione S-transferase theta 1	MSTRG.12200.2	0.67	down	0.23	Q01579	0.80	down	1.36E-15	0.44	hypo-methylated	20	"+"	exon	5.43E-04
	Fmo5	flavin containing monooxygenase 5	ENSRNOT00000024423	1.49	up	0.37	Q8K4C0	1.26	up	1.37E-07						
Metabolism of xenobiotics by cytochrome P450	Mgst2	microsomal glutathione S-transferase 2	MSTRG.10921.1	0.97	down	0.2	A0A0G2JU12	1.49	up	4.54E-11						
	Mgst2	microsomal glutathione S-transferase 2	MSTRG.10921.2	1.07	up	0.88	A0A0G2JU12	1.49	up	4.54E-11						
	Gsta3	glutathione S-transferase alpha 3	ENSRNOT00000088416	0.76	down	0.31	P04904	0.64	down	2.87E-15	2.74	hyper-methylated	9	"+"	exon	2.28E-04
	Gsta3	glutathione S-transferase alpha 3	ENSRNOT00000077372	1.01	up	0.84	P04904	0.64	down	2.87E-15	2.74	hyper-methylated	9	"+"	exon	2.28E-04
	Adh1	alcohol dehydrogenase 1 (class I)	ENSRNOT00000083682	0.89	down	0.64	P06757	0.78	down	2.84E-16						
	Adh1	alcohol dehydrogenase 1 (class I)	MSTRG.11708.1	1	down	0.97	P06757	0.78	down	2.84E-16						
	Adh1	alcohol dehydrogenase 1 (class I)	ENSRNOT00000085067	0.99	down	0.97	P06757	0.78	down	2.84E-16						
	Ephx1	epoxide hydrolase 1, microsomal (xenobiotic)	ENSRNOT00000085279	0.89	down	0.77	P07687	0.69	down	5.24E-14	0.19	hypo-methylated	13	"-"	promoter	2.92E-07
	Ephx1	epoxide hydrolase 1, microsomal (xenobiotic)	ENSRNOT00000004780	0.92	down	0.69	P07687	0.69	down	5.24E-14	0.19	hypo-methylated	13	"-"	promoter	2.92E-07
	Gstm3	glutathione S-transferase mu 3	ENSRNOT00000025800	1.01	up	0.91	P08009	0.59	down	2.02E-20						
	Aldh3a1	aldehyde dehydrogenase 3 family, member A1	ENSRNOT00000003182	0.45	down	0.25	P11883	0.72	down	6.70E-23						
	Gsta4	glutathione S-transferase alpha 4	MSTRG.20020.1	0.78	down	0.48	P14942	0.76	down	4.34E-13						
	Gsta4	glutathione S-transferase alpha 4	ENSRNOT00000090146	0.88	down	0.55	P14942	0.76	down	4.34E-13						
	Gsta4	glutathione S-transferase alpha 4	ENSRNOT00000012348	1.17	up	0.76	P14942	0.76	down	4.34E-13						
	Gstt1	glutathione S-transferase theta 1	ENSRNOT00000001669	0.92	down	0.8	Q01579	0.80	down	1.36E-15	0.44	hypo-methylated	20	"+"	exon	5.43E-04
	Gstt1	glutathione S-transferase theta 1	MSTRG.12200.2	0.67	down	0.23	Q01579	0.80	down	1.36E-15	0.44	hypo-methylated	20	"+"	exon	5.43E-04
ECM-receptor interaction	Col6a5	collagen, type VI, alpha 5	ENSRNOT00000038160	0.84	down	0.15	A0A0G2JTX7	0.69	down	9.07E-21						
	Col6a5	collagen, type VI, alpha 5	MSTRG.20206.1	0.66	down	0.12	A0A0G2JTX7	0.69	down	9.07E-21						
	Col6a5	collagen, type VI, alpha 5	ENSRNOT00000082992	1.08	up	0.44	A0A0G2JTX7	0.69	down	9.07E-21						
	Itga5	integrin subunit alpha 5	MSTRG.19340.1	0.75	down	0.58	A0A0G2K1E2	0.79	down	6.51E-16						
	Itga5	integrin subunit alpha 5	ENSRNOT00000087748	0.52	down	0.18	A0A0G2K1E2	0.79	down	6.51E-16						
	Tnc	tenascin C	ENSRNOT00000084563	2.22	up	0.36	A0A0G2K1L0	1.49	up	1.46E-12	0.41	hypo-methylated	5	"-"	intron	2.15E-05
	Tnc	tenascin C	MSTRG.15938.2	1.15	up	0.84	A0A0G2K1L0	1.49	up	1.46E-12	0.41	hypo-methylated	5	"-"	intron	2.15E-05
	Tnc	tenascin C	MSTRG.15938.3	1.23	up	0.46	A0A0G2K1L0	1.49	up	1.46E-12	0.41	hypo-methylated	5	"-"	intron	2.15E-05
	Col4a6	collagen, type IV, alpha 6	MSTRG.21902.4	1.56	up	0.05	A0A0G2K601	0.78	down	3.69E-15						
	Col4a6	collagen, type IV, alpha 6	MSTRG.21902.1	1.32	up	0.42	A0A0G2K601	0.78	down	3.69E-15						
	Col4a6	collagen, type IV, alpha 6	MSTRG.21902.3	0.56	down	0.6	A0A0G2K601	0.78	down	3.69E-15						
	Col4a6	collagen, type IV, alpha 6	ENSRNOT00000079180	0.56	down	0.46	A0A0G2K601	0.78	down	3.69E-15						
	Col6a6	collagen, type VI, alpha 6	ENSRNOT00000056414	0.54	down	0.06	D3ZL10	0.72	down	2.01E-20						
	Lamb1	laminin, beta 1	ENSRNOT00000008321	2.02	up	0.49	D3ZQN7	1.31	up	5.36E-17	3.86	hyper-methylated	6	"+"	intron	8.86E-05
	Lamb1	laminin, beta 1	MSTRG.17232.1	1.22	up	0.71	D3ZQN7	1.31	up	5.36E-17	3.86	hyper-methylated	6	"+"	intron	8.86E-05
	Lamb1	laminin, beta 1	MSTRG.17232.2	3.85	up	0.02	D3ZQN7	1.31	up	5.36E-17	3.86	hyper-methylated	6	"+"	intron	8.86E-05
	Thbs4	thrombospondin 4	MSTRG.10411.3	1.07	up	0.47	F1LMS5	1.25	up	3.66E-10	0.15	hypo-methylated	2	"-"	intron	3.98E-04
	Thbs4	thrombospondin 4	MSTRG.10411.2	0.99	down	0.45	F1LMS5	1.25	up	3.66E-10	0.15	hypo-methylated	2	"-"	intron	3.98E-04
	Thbs4	thrombospondin 4	ENSRNOT00000065224	2.16	up	0.08	F1LMS5	1.25	up	3.66E-10	0.15	hypo-methylated	2	"-"	intron	3.98E-04
	Thbs4	thrombospondin 4	ENSRNOT00000083531	2.15	up	0.1	F1LMS5	1.25	up	3.66E-10	0.15	hypo-methylated	2	"-"	intron	3.98E-04
	Col2a1	collagen, type II, alpha 1	ENSRNOT00000086062	0.98	down	0.33	F1LRM7	0.58	down	9.54E-17						
	Fn1	fibronectin 1	MSTRG.21023.11	8.43	up	0.01	F1LST1	1.27	up	4.07E-20	0.11	hypo-methylated	9	"-"	intron	2.24E-04
	Fn1	fibronectin 1	MSTRG.21023.6	7.56	up	0.49	F1LST1	1.27	up	4.07E-20	0.11	hypo-methylated	9	"-"	intron	2.24E-04
	Fn1	fibronectin 1	MSTRG.21023.9	0.17	down	0.09	F1LST1	1.27	up	4.07E-20	0.11	hypo-methylated	9	"-"	intron	2.24E-04
	Fn1	fibronectin 1	ENSRNOT00000019772	0.52	down	0.62	F1LST1	1.27	up	4.07E-20	0.11	hypo-methylated	9	"-"	intron	2.24E-04
	Fn1	fibronectin 1	MSTRG.21023.8	9.19	up	0.42	F1LST1	1.27	up	4.07E-20	0.11	hypo-methylated	9	"-"	intron	2.24E-04
	Fn1	fibronectin 1	MSTRG.21023.4	1.68	up	0.61	F1LST1	1.27	up	4.07E-20	0.11	hypo-methylated	9	"-"	intron	2.24E-04
	Fn1	fibronectin 1	ENSRNOT00000057585	1.31	up	0.79	F1LST1	1.27	up	4.07E-20	0.11	hypo-methylated	9	"-"	intron	2.24E-04
	Fn1	fibronectin 1	MSTRG.21023.2	1.2	up	0.84	F1LST1	1.27	up	4.07E-20	0.11	hypo-methylated	9	"-"	intron	2.24E-04
	Fn1	fibronectin 1	MSTRG.21023.3	1.42	up	0.73	F1LST1	1.27	up	4.07E-20	0.11	hypo-methylated	9	"-"	intron	2.24E-04
	Fn1	fibronectin 1	MSTRG.21023.5	8.58	up	0.48	F1LST1	1.27	up	4.07E-20	0.11	hypo-methylated	9	"-"	intron	2.24E-04
	Fn1	fibronectin 1	MSTRG.21023.10	9.31	up	0.44	F1LST1	1.27	up	4.07E-20	0.11	hypo-methylated	9	"-"	intron	2.24E-04
	Spp1	secreted phosphoprotein 1	ENSRNOT00000075989	5.34	up	0.17	P08721	1.32	up	5.17E-14	29.54	hyper-methylated	14	"+"	exon	1.81E-44
	Spp1	secreted phosphoprotein 1	MSTRG.6515.2	4.8	up	0.11	P08721	1.32	up	5.17E-14	29.54	hyper-methylated	14	"+"	exon	1.81E-44
	Spp1	secreted phosphoprotein 1	ENSRNOT00000067875	7.36	up	0.06	P08721	1.32	up	5.17E-14	29.54	hyper-methylated	14	"+"	exon	1.81E-44
	Vtn	vitronectin	ENSRNOT00000078897	1.01	up	0.76	Q3KR94	1.27	up	2.27E-07						
	Vtn	vitronectin	ENSRNOT00000039954	1.2	up	0.41	Q3KR94	1.27	up	2.27E-07						
	Col4a1	collagen, type IV, alpha 1	MSTRG.8454.1	2.88	up	0.52	Q5FWY9	1.87	up	1.30E-15						
	Col4a1	collagen, type IV, alpha 1	ENSRNOT00000057386	1.58	up	0.25	Q5FWY9	1.87	up	1.30E-15						
	Cav2	caveolin 2	MSTRG.14336.1	0.75	down	0.46	A0A0A0MXU8	0.76	down	5.70E-15						
	Cav2	caveolin 2	ENSRNOT00000080271	0.74	down	0.1	A0A0A0MXU8	0.76	down	5.70E-15						
	Col6a5	collagen, type VI, alpha 5	ENSRNOT00000038160	0.84	down	0.15	A0A0G2JTX7	0.69	down	9.07E-21						
	Col6a5	collagen, type VI, alpha 5	MSTRG.20206.1	0.66	down	0.12	A0A0G2JTX7	0.69	down	9.07E-21						
	Col6a5	collagen, type VI, alpha 5	ENSRNOT00000082992	1.08	up	0.44	A0A0G2JTX7	0.69	down	9.07E-21						
	Itga5	integrin subunit alpha 5	MSTRG.19340.1	0.75	down	0.58	A0A0G2K1E2	0.79	down	6.51E-16						
	Itga5	integrin subunit alpha 5	ENSRNOT00000087748	0.52	down	0.18	A0A0G2K1E2	0.79	down	6.51E-16						
	Tnc	tenascin C	ENSRNOT00000084563	2.22	up	0.36	A0A0G2K1L0	1.49	up	1.46E-12	0.41	hypo-methylated	5	"-"	intron	2.15E-05
	Tnc	tenascin C	MSTRG.15938.2	1.15	up	0.84	A0A0G2K1L0	1.49	up	1.46E-12	0.41	hypo-methylated	5	"-"	intron	2.15E-05
	Tnc	tenascin C	MSTRG.15938.3	1.23	up	0.46	A0A0G2K1L0	1.49	up	1.46E-12	0.41	hypo-methylated	5	"-"	intron	2.15E-05
	Col4a6	collagen, type IV, alpha 6	MSTRG.21902.4	1.56	up	0.05	A0A0G2K601	0.78	down	3.69E-15						
	Col4a6	collagen, type IV, alpha 6	MSTRG.21902.1	1.32	up	0.42	A0A0G2K601	0.78	down	3.69E-15						
	Col4a6	collagen, type IV, alpha 6	MSTRG.21902.3	0.56	down	0.6	A0A0G2K601	0.78	down	3.69E-15						
	Col4a6	collagen, type IV, alpha 6	ENSRNOT00000079180	0.56	down	0.46	A0A0G2K601	0.78	down	3.69E-15						

	Flnc	filamin C, gamma	ENSRNOT00000027237	1.11	up	0.35	A0A0H2UHR7	0.75	down	1.10E-19						
	Flnc	filamin C, gamma	ENSRNOT00000088149	0.84	down	0.49	A0A0H2UHR7	0.75	down	1.10E-19						
	Mapk10	mitogen activated protein kinase 10	ENSRNOT00000092542	1	down	0.4	A0A0U1RRS7	0.56	down	6.75E-04	6.77	hyper-methylated	14	"+"	intron	5.13E-18
	Mapk10	mitogen activated protein kinase 10	ENSRNOT00000065965	0.99	down	0.41	A0A0U1RRS7	0.56	down	6.75E-04	6.77	hyper-methylated	14	"+"	intron	5.13E-18
	Mapk10	mitogen activated protein kinase 10	ENSRNOT00000092395	0.99	down	0.45	A0A0U1RRS7	0.56	down	6.75E-04	6.77	hyper-methylated	14	"+"	intron	5.13E-18
	Cav1	caveolin 1	ENSRNOT00000078250	0.54	down	0.07	B1WBN8	0.73	down	8.89E-14						
	Cav1	caveolin 1	MSTRG.14338.1	0.99	down	0.53	B1WBN8	0.73	down	8.89E-14						
	Myl10	myosin, light chain 10, regulatory	ENSRNOT00000041654	1.05	up	0.09	D3ZCI9	0.76	down	1.15E-16						
	Col6a6	collagen, type VI, alpha 6	ENSRNOT00000056414	0.54	down	0.06	D3ZL10	0.72	down	2.01E-20						
	Lamb1	laminin, beta 1	ENSRNOT00000008321	2.02	up	0.49	D3ZQN7	1.31	up	5.36E-17	3.86	hyper-methylated	6	"+"	intron	8.86E-05
	Lamb1	laminin, beta 1	MSTRG.17232.1	1.22	up	0.71	D3ZQN7	1.31	up	5.36E-17	3.86	hyper-methylated	6	"+"	intron	8.86E-05
	Lamb1	laminin, beta 1	MSTRG.17232.2	3.85	up	0.02	D3ZQN7	1.31	up	5.36E-17	3.86	hyper-methylated	6	"+"	intron	8.86E-05
**Focal adhesion**	Thbs4	thrombospondin 4	MSTRG.10411.3	1.07	up	0.47	F1LMS5	1.25	up	3.66E-10	0.15	hypo-methylated	2	"-"	intron	3.98E-04
	Thbs4	thrombospondin 4	MSTRG.10411.2	0.99	down	0.45	F1LMS5	1.25	up	3.66E-10	0.15	hypo-methylated	2	"-"	intron	3.98E-04
	Thbs4	thrombospondin 4	ENSRNOT00000065224	2.16	up	0.08	F1LMS5	1.25	up	3.66E-10	0.15	hypo-methylated	2	"-"	intron	3.98E-04
	Thbs4	thrombospondin 4	ENSRNOT00000083531	2.15	up	0.1	F1LMS5	1.25	up	3.66E-10	0.15	hypo-methylated	2	"-"	intron	3.98E-04
	Col2a1	collagen, type II, alpha 1	ENSRNOT00000086062	0.98	down	0.33	F1LRM7	0.58	down	9.54E-17						
	Fn1	fibronectin 1	MSTRG.21023.11	8.43	up	0.01	F1LST1	1.27	up	4.07E-20	0.11	hypo-methylated	9	"-"	intron	2.24E-04
	Fn1	fibronectin 1	MSTRG.21023.6	7.56	up	0.49	F1LST1	1.27	up	4.07E-20	0.11	hypo-methylated	9	"-"	intron	2.24E-04
	Fn1	fibronectin 1	MSTRG.21023.9	0.17	down	0.09	F1LST1	1.27	up	4.07E-20	0.11	hypo-methylated	9	"-"	intron	2.24E-04
	Fn1	fibronectin 1	ENSRNOT00000019772	0.52	down	0.62	F1LST1	1.27	up	4.07E-20	0.11	hypo-methylated	9	"-"	intron	2.24E-04
	Fn1	fibronectin 1	MSTRG.21023.8	9.19	up	0.42	F1LST1	1.27	up	4.07E-20	0.11	hypo-methylated	9	"-"	intron	2.24E-04
	Fn1	fibronectin 1	MSTRG.21023.4	1.68	up	0.61	F1LST1	1.27	up	4.07E-20	0.11	hypo-methylated	9	"-"	intron	2.24E-04
	Fn1	fibronectin 1	ENSRNOT00000057585	1.31	up	0.79	F1LST1	1.27	up	4.07E-20	0.11	hypo-methylated	9	"-"	intron	2.24E-04
	Fn1	fibronectin 1	MSTRG.21023.2	1.2	up	0.84	F1LST1	1.27	up	4.07E-20	0.11	hypo-methylated	9	"-"	intron	2.24E-04
	Fn1	fibronectin 1	MSTRG.21023.3	1.42	up	0.73	F1LST1	1.27	up	4.07E-20	0.11	hypo-methylated	9	"-"	intron	2.24E-04
	Fn1	fibronectin 1	MSTRG.21023.5	8.58	up	0.48	F1LST1	1.27	up	4.07E-20	0.11	hypo-methylated	9	"-"	intron	2.24E-04
	Fn1	fibronectin 1	MSTRG.21023.10	9.31	up	0.44	F1LST1	1.27	up	4.07E-20	0.11	hypo-methylated	9	"-"	intron	2.24E-04
	Spp1	secreted phosphoprotein 1	ENSRNOT00000075989	5.34	up	0.17	P08721	1.32	up	5.17E-14	29.54	hyper-methylated	14	"+"	exon	1.81E-44
	Spp1	secreted phosphoprotein 1	MSTRG.6515.2	4.8	up	0.11	P08721	1.32	up	5.17E-14	29.54	hyper-methylated	14	"+"	exon	1.81E-44
	Spp1	secreted phosphoprotein 1	ENSRNOT00000067875	7.36	up	0.06	P08721	1.32	up	5.17E-14	29.54	hyper-methylated	14	"+"	exon	1.81E-44
	Ppp1cc	protein phosphatase 1 catalytic subunit gamma	ENSRNOT00000078761	1.05	up	0.47	P63088	0.69	down	9.22E-05						
	Ppp1cc	protein phosphatase 1 catalytic subunit gamma	MSTRG.5563.2	1	down	0.99	P63088	0.69	down	9.22E-05						
	Ppp1cc	protein phosphatase 1 catalytic subunit gamma	ENSRNOT00000048851	1.05	up	0.59	P63088	0.69	down	9.22E-05						
	Ppp1cc	protein phosphatase 1 catalytic subunit gamma	ENSRNOT00000083296	1.19	up	0.26	P63088	0.69	down	9.22E-05						
	Vtn	vitronectin	ENSRNOT00000078897	1.01	up	0.76	Q3KR94	1.27	up	2.27E-07						
	Vtn	vitronectin	ENSRNOT00000039954	1.2	up	0.41	Q3KR94	1.27	up	2.27E-07						
	Col4a1	collagen, type IV, alpha 1	MSTRG.8454.1	2.88	up	0.52	Q5FWY9	1.87	up	1.30E-15						
	Col4a1	collagen, type IV, alpha 1	ENSRNOT00000057386	1.58	up	0.25	Q5FWY9	1.87	up	1.30E-15						
